# Electro‐mediated PhotoRedox Catalysis for Selective C(sp^3^)–O Cleavages of Phosphinated Alcohols to Carbanions

**DOI:** 10.1002/anie.202105895

**Published:** 2021-08-16

**Authors:** Xianhai Tian, Tobias A. Karl, Sebastian Reiter, Shahboz Yakubov, Regina de Vivie‐Riedle, Burkhard König, Joshua P. Barham

**Affiliations:** ^1^ Institute of Organic Chemistry University of Regensburg Universitätsstr. 31 93053 Regensburg Germany; ^2^ Department of Chemistry LMU Munich 81377 Munich Germany

**Keywords:** deoxygenation, olefination, photoelectrochemistry, preassembly, radical anion

## Abstract

We report a novel example of electro‐mediated photoredox catalysis (e‐PRC) in the reductive cleavage of C(sp^3^)−O bonds of phosphinated alcohols to alkyl carbanions. As well as deoxygenations, olefinations are reported which are *E*‐selective and can be made *Z*‐selective in a tandem reduction/photosensitization process where both steps are photoelectrochemically promoted. Spectroscopy, computation, and catalyst structural variations reveal that our new naphthalene monoimide‐type catalyst allows for an intimate dispersive precomplexation of its radical anion form with the phosphinate substrate, facilitating a reactivity‐determining C(sp^3^)−O cleavage. Surprisingly and in contrast to previously reported photoexcited radical anion chemistries, our conditions tolerate aryl chlorides/bromides and do not give rise to Birch‐type reductions.

## Graphical abstract

Beyond C(sp^2^)–X cleavage: We report reductive cleavages of C(sp^3^)−O bonds of phosphinated alcohols to alkyl carbanions by electro‐mediated photoredox catalysis. Deoxygenations as well as *E*‐ or *Z*‐selective olefinations are reported. Surprisingly, reactions tolerate C(sp^2^)–X cleavages such as aryl halides. Radical ion catalyst‐substrate preassembly is key in enabling reactivity and overturning conventional redox chemoselectivity.

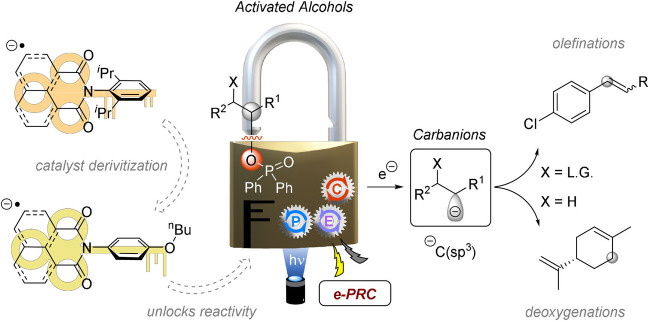

## Introduction

**Scheme 1:**
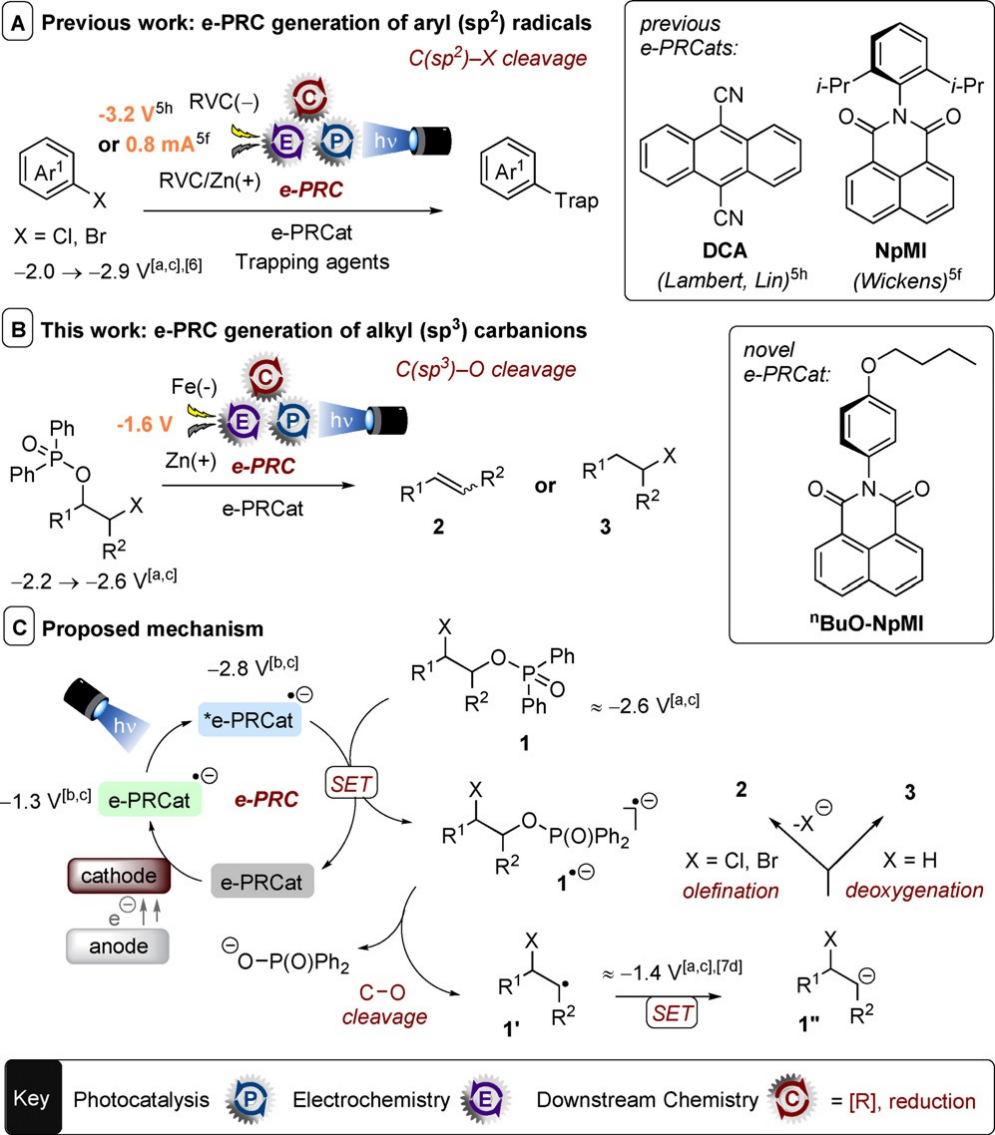
Previous reductive e‐PRC reports involving C(sp^2^)−X cleavages to afford aryl radicals vs. this work involving C(sp^3^)−O cleavages to afford alkyl radicals and carbanions. [a] *E* ^p^_red_. [b] *E*_1/2_. [c] Redox potential vs. SCE.

Synthetic methodologies involving single electron transfer (SET) are increasingly popular for the facile synthesis or modifications of important organic compounds. PhotoRedox Catalysis (PRC)^[1]^ and Synthetic Organic Electrochemistry (SOE)^[2]^ lead to easy SET processes, providing notable redox power for various organic transformations under mild conditions. Generally, visible‐light PRC generates radical intermediates with good functional group tolerance in a mild manner. However, synthetic applications of PRC in terms of transformations needing highly oxidizing or reducing potentials are limited by the energetic limitations of visible light photons. One solution is to generate photoexcitable radical ions by multi‐photon processes.^[3]^ Such photoexcited radical ions are highly oxidizing[^3a^, ^3b^] or reducing species,[^3c^, ^3d^, ^3e^, ^3f^, ^3g^, ^3h^] leading to a significantly expanded redox “window” for activating inert substrates. Sacrificial redox additives (e.g. DIPEA) are employed in stoichiometric excesses in consecutive Photoinduced Electron Transfer (conPET) processes to prime catalysts prior to excitation. Their excesses and organic by‐products can plague purification steps. In contrast, SOE allows direct access to high, user‐controlled redox energy without involving photocatalysts or sacrificial redox additives, offering advantages to net‐oxidative/reductive reactions. However, the applied constant current or voltage can cause uncontrollable over‐reductions/oxidations to afford by‐products. To address the aforementioned limitations in PRC and SOE, organic chemists have recently explored their combination (Scheme 1).^[4]^

Merging the advantages of these two important techniques has made photoelectrochemistry a tool for greener, more challenging and more selective molecular activations.^[5]^ Pioneering reports by Xu,[^5b^, ^5c^, ^5m^] Lambert,[^5g^, ^5h^, ^5i^, ^5k^] Lin[^5h^, ^5j^] and Wickens^[5f]^ have shown that introducing applied potential in photoredox catalysis is not only beneficial for accessing challenging redox reactions, but is also a green replacement for sacrificial redox additives.

Among the various strategies for combining photocatalysis and electrochemistry^[4a]^ the sub‐category coined electrochemically‐mediated photoredox catalysis (e‐PRC) is highly attractive. In addition to turning over “spent” closed‐shell photocatalysts, e‐PRC can also involve electrochemical generation of open‐shell (radical ion) photocatalysts, followed by their photoexcitation to species with ultra‐high redox potentials. A seminal report from the Lambert group demonstrated this strategy for super‐oxidations of highly electron‐poor arenes.^[5k]^ In the reductive direction, photoexcited radical anions of dicyanoanthracene (**DCA**)^[5h]^ and of 2,6‐diisopropylphenyl‐containing naphthalenemonoimide (**NpMI**)^[5f]^ are highly reducing species (*E*°_*red*_ < −3.0 V vs. SCE) that reduce challenging aryl chlorides to their aryl radicals. Even *p*‐chloroanisole was reduced, beyond reach of the photon energy limit of monophotonic PRC and where SOE inevitably leads to dehalogenation via subsequent aryl radical reduction (Scheme 1 A).^[6]^ Despite these elegant advances, reductive e‐PRC and biphotonic strategies^[3]^ are still heavily focused on the reductions of aryl halides/pseudohalides through C(sp^2^)−X bond cleavages to generate aryl C(sp^2^) radicals in an overall dehalogenation or functionalization with excesses of radical trapping agents.[^5f^, ^5h^]

Inspired by previous reports,^[5]^ we envisioned that phosphinates of aliphatic alcohols (*E* ^p^_red_=−2.2 → −2.6 V vs. SCE) could undergo e‐PRC reduction to give carbanions (Scheme 1 B). Thereby, an electroactivated‐PhotoRedox Catalyst (e‐PRCat) undergoes cathodic activation and photoexcitation to afford a potent reductant. SET reduction of **1** to its radical anion followed by C(sp^3^)−O bond cleavage delivers benzyl radical **1′**. Its further reduction^[7d]^ to carbanion intermediate **1”** would enable either an olefination (X=Cl, Br) or a deoxygenation (X=H) process by a mechanism that does not depend on hydrogen atom transfer agents or decarboxylation.^[7]^ Herein, we report the e‐PRC reduction of alkyl phosphinates to alkyl(sp^3^) carbanions for olefination and deoxygenation reactions that i) proceeds under exceedingly mild conditions, ii) tolerates aryl halides/pseudohalides with similar or more accessible redox potentials than the target alkyl phosphinate moiety.

## Results and Discussion

To assess the viability of our proposed e‐PRC alkyl phosphinate reduction, we employed 2‐chloro‐1,2‐diphenylphosphinate **1 a** as a model substrate for the olefination reaction (Table 1). By using **DCA** as an e‐PRCat and Zn(+)/RVC(−) as the electrodes in a divided H‐cell, we examined the reduction of **1 a** under blue light irradiation and with different applied constant potentials. A high constant voltage (*U*_cell_=−3.2 V) as used previously^[5h]^ for electron‐priming **DCA** to its radical anion for photoexcitation gave notable decomposition, desired product *E*‐stilbene (*E*‐**2 a**) in only 7 % yield and a 25 % yield of diphenylethane **3 a** ^[8]^ (Table 1, entry 1). A lower potential (*U*_cell_=−1.6 V) led to a remarkable improvement in the reaction profile and yield of *E*‐**2 a** to 70 % (Table 1, entry 2). The optimal yield of *E*‐**2 a** was obtained at an even lower potential (*U*_cell_=−1.0 V). Cyclic phosphate ester **4 a** was also a suitable substrate for preparing product *E*‐**2 a** (entry 4), offering an attractive Corey–Winter‐type olefination that avoids explosive/toxic trimethylphosphite, harsh activating reagents or high temperature. Control reactions omitting light, constant potential or e‐PRCat confirmed the photoelectrochemical nature of the olefination reaction (entries 5–7). In contrast to **DCA**, **NpMI** as catalyst delivered higher amounts of *Z*‐**2 a** (entry 8).^[9]^ Allowing the reaction to proceed for 48 h (entry 9) increased the *E*‐/*Z*‐ ratio to 1/10 (71 % of *Z*‐**2 a**). Detailed investigations (see Supporting Information (SI)) revealed that light, constant potential and **NpMI** are all advantageous to the isomerization, representing a novel photoelectroisomerism of alkenes.

**Table 1:** Optimization of the reaction conditions.^[a]^ 
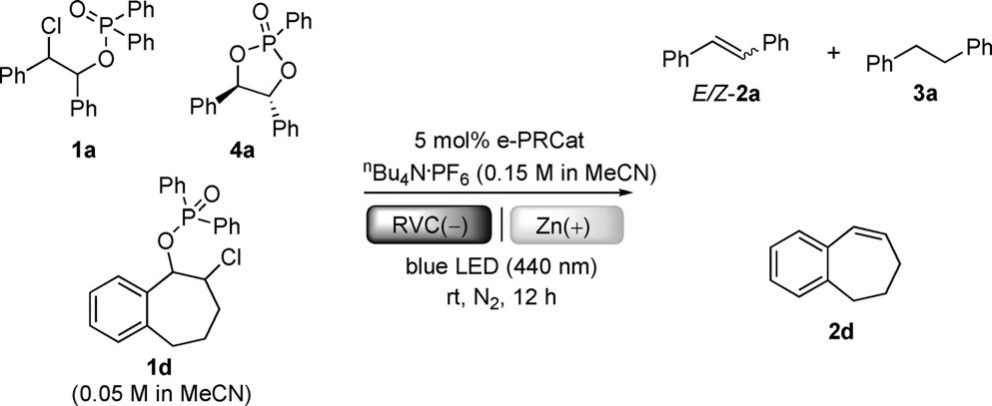

Entry	Substrate	e‐PRCat	*U*_cell_ [V]	*t* [h]	Product: Yield^[a]^
1	**1 a**	**DCA**	−3.2	12	**2 a**: 7 %, *E‐/Z‐*>20:1^[b]^ **3 a**: 25 %
2	**1 a**	**DCA**	−1.6	12	**2 a**: 70 %, *E‐/Z‐*>20:1^[b]^ **3 a**: trace
3	**1 a**	**DCA**	−1.0	12	**2 a**: 79 %, *E‐/Z‐*>20:1^[b]^ **3 a**: n.d.
4	**4 a**	**DCA**	−1.0	24	**2 a**: 79 %, *E‐/Z‐*>20:1^[b]^ **3 a**: n.d.
5^[c]^	**1 a**	**DCA**	−1.0	12	**2 a**: n.d. **3 a**: n.d.
6	**1 a**	**DCA**	–	12	**2 a**: n.d. **3 a**: n.d.
7	**1 a**	–	−1.0	12	**2 a**: trace **3 a**: n.d.
8	**1 a**	**NpMI**	−1.6	12	**2 a**: 80 %, *E‐/Z‐*=1:1.3^[b]^ **3 a**: n.d.
9	**1 a**	**NpMI**	−1.6	48	**2 a**: 78 %, *E‐/Z‐*=1:10^[b]^ **3 a**: n.d.
10^[d]^	**1 d**	**DCA**	−1.0	12	**2 d**: n.d.
11^[d]^	**1 d**	**NpMI**	−1.6	12	**2 d**: trace
12^[d]^	**1 d**	^**n**^ **BuO‐NpMI**	−1.6	12	**2 d**: 75 %
13^[d]^	**1 d**	^**n**^ **BuO‐NpMI**	–	12	**2 d**: n.d.
14^[c,d]^	**1 d**	^**n**^ **BuO‐NpMI**	−1.6	12	**2 d**: n.d.
15^[d]^	**1 d**	–	−1.6	12	**2 d**: <5 %

[a] n.d.=not detected; yields determined by ^1^H NMR spectroscopy with 1,3,5‐trimethoxybenzene as an internal standard. [b] *E‐*/*Z‐* ratios determined by ^1^H NMR spectroscopy. [c] In the dark. [d] Fe cathode.

**Scheme 2:**
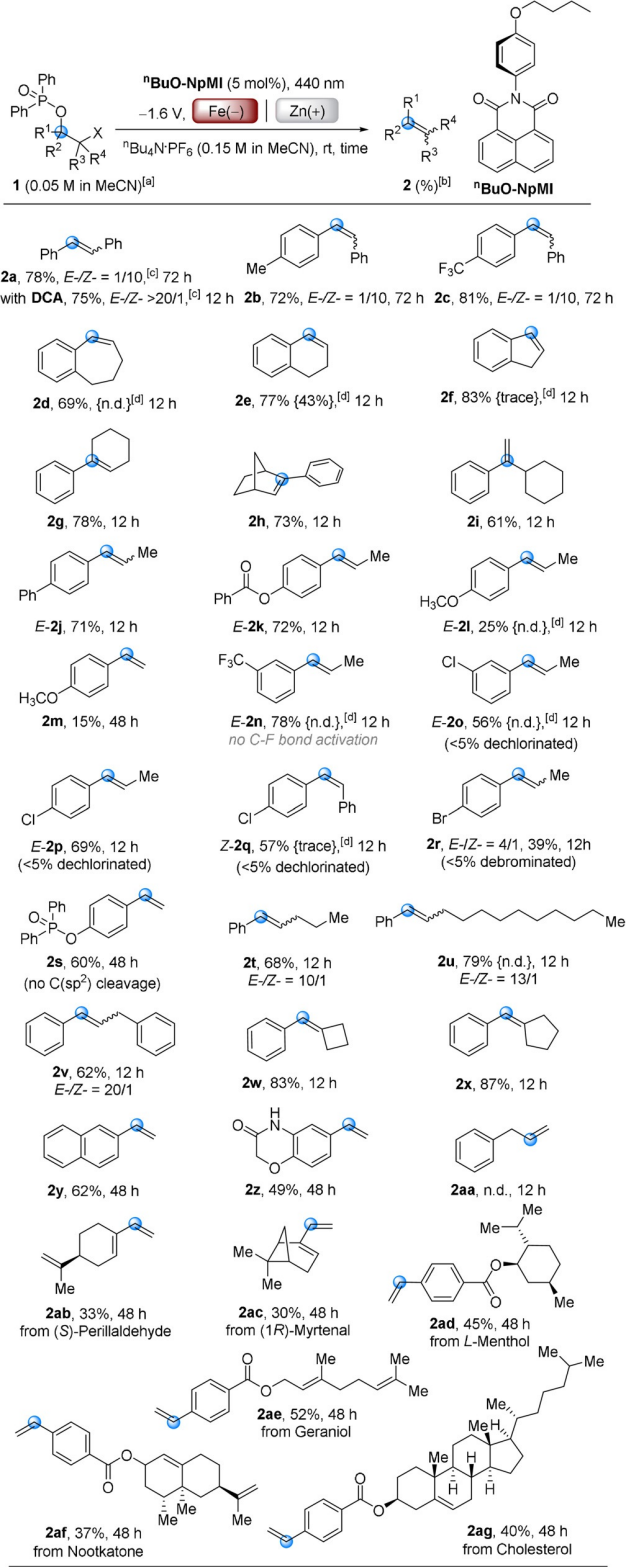
e‐PRC reductive olefination scope. [a] for compounds **2 a**–**2 q**, **2 t**–**2 x**, **2 aa**–**2 ad**, X=Cl; for compounds **2 r**–**2 s**, **2 y**–**2 z**, **2 ae**–**2 ag**, X=Br. [b] Isolated yields. [c] *E*‐/*Z*‐ ratios determined by ^1^H NMR spectroscopy. [d] Yields in parentheses {} are ^1^H NMR yields from **NpMI** as an e‐PRCat.

Reaction scope was expanded to other substrates including precursors to unsymmetrical stilbenes as well as cyclic, hindered and terminal olefins. Phosphinate precursors are readily synthesized from their ketones via α‐chlorination and one‐pot NaBH_4_ reduction/Cl‐P(O)Ph_2_ protection (see SI). Here we opted to use Fe instead of RVC as a cheaper, robust cathode material.^[10]^ However, it was quickly identified that **DCA** and **NpMI** were ineffective e‐PRCats for the majority of phosphinates. For example, cyclic substrate **1 d** underwent no reaction with these catalysts (entries 10–11). We synthesized ^**n**^ **BuO‐NpMI** as a novel e‐PRCat which afforded the desired product **2 d** in very good yield (entry 12). Control reactions confirmed operation of e‐PRC (entries 13–15), while cathode materials greatly impacted the reaction (for detailed optimizations, see SI).^[11]^ Optimal conditions were examined for a range of olefination reactions (Scheme 2). Unsymmetrical *Z*‐stilbenes **2 b**, **2 c** were prepared in high yields from the tandem e‐PRC reduction/photoelectroisomerism process. Cyclic olefins **2 d**–**2 h**, rarely synthesized by the Wittig reaction due to the inconvenience of substrate preparations, were prepared in good to excellent (69–83 %) yields. Terminal olefin **2 i** could not be prepared in high selectivity by dehydration of its corresponding tertiary alcohol as such a method inevitably leads to the most substituted olefin,^[12]^ in this case, a tetrasubstituted instead of a terminal olefin.

After the successful preparations of a series of *E*‐styrene derivatives (exclusive isomers) bearing divergent substituents including ‐Ph (**2 j**), ‐OBz(**2 k**), ‐OMe(**2 l**) and ‐CF_3_(**2 n**) at their arene rings, we questioned whether halogen substituents could be tolerated by our reaction. This is a highly challenging issue, since the reductions of aryl chlorides and bromides by photoexcited radical anions (either e‐PRC or conPET‐type) are highly efficient and heavily reported as discussed earlier (Scheme 1).[^3c^, ^3d^, ^3e^, ^3f^, ^3g^, ^5f^, ^5h^] With this aim, we tested phosphinates bearing either a chloro‐ or bromo‐ substituent on their arene. To our delight, aryl chlorides **1 o**–**1 q** and aryl bromide **1 r** underwent olefination in moderate to good (39–69 %) yields with high or exclusive selectivities for their *E*‐ or *Z*‐ isomers; only traces of dehalogenated styrenes were observed (>10:1 in favor of olefination for **2 p**). Compared with products **2 o**–**2 p**, *p*‐chlorostilbene **2 q** has a more conjugated π‐system and is easier to reduce, yet still gave only traces of dechlorinated product **2 a**. Substrate **1 s**, bearing both an alkyl and aryl phosphinate,^[13]^ selectively underwent e‐PRC reduction of the alkyl phosphinate leading only to C(sp^3^)−O cleavage to afford **2 s** in good yield. Our method retains reductively labile C(sp^2^)−O functionality, providing complementary selectivity to a recent report involving a phenothiazine photocatalyst.^[13]^

Styrene‐forming substrates containing longer‐chain aliphatic groups or a benzyl group retained high *E*‐isomer selectivity, affording **2 t**–**2 v** in good to high (62–79 %) yields and high selectivities (>10:1 in favor of their *E*‐isomers). Olefin geometry is not impacted by the diastereomeric ratio of phosphinate precursors, but by the reaction conditions. For example, although the diastereomeric ratios of phosphinate precursors to **2 r**, **2 t** and **2 v** were all >30:1, the *E*‐/*Z*‐ ratios were 4:1, 10:1 and 20:1 respectively. Hindered olefins derived from carbocycles **1 w**–**1 x** were formed in high (83–87 %) yields. In the synthesis of **2 x**, our conditions offer an alternative to i) ^n^BuLi or Grignard chemistry with expensive bromocyclobutane and ii) expensive Wittig reagents/cyclobutanone, instead starting from commercial, inexpensive cyclobutyl phenyl ketone. Our e‐PRC phosphinate reduction offers complementary selectivity to Birch‐type photochemical reports involving SET,^[14]^ or E_n_T.^[15]^ Naphthalene‐based substrate **1 y** was well‐tolerated, affording **2 y** in good (62 %) yield without Birch‐type reduction products. Amide **1 z** was also well‐tolerated, in spite of its free proton and labile heterocycle that would react with strong bases. Although an alkyl phosphinate derived from a non‐benzylic alcohol **1 aa** did not react, alkyl phosphinates derived from allylic alcohols were feasible. Allylic substrates **1 ab**, **1 ac** derived from naturally‐occurring terpenes were found to be sluggish, but afforded dienes **2 ab**, **2 ac** in satisfactory (30–33 %) yields in a complementary fashion to previous reports that require strong bases^[16]^ or transition metal catalysis.^[17]^

**Scheme 3:**
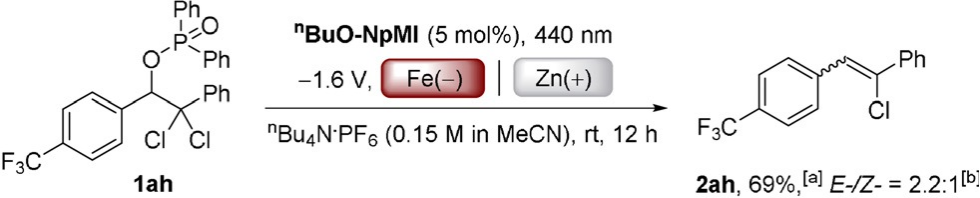
e‐PRC reduction of dichlorinated substrate **1 ah**. [a] Yield of isolated product. [b] *E*‐/*Z*‐ ratio was determined by ^1^H NMR spectroscopy.

**Scheme 4:**
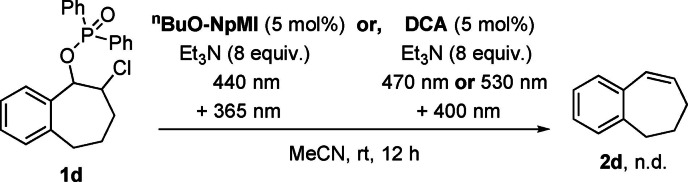
Attempted reduction of **1 d** under conPET conditions.

Demonstrating the utility of our base‐free approach, products **2 ad**–**2 ag** were synthesized from their alkyl *p*‐acetylbenzoate precursors. Given the properties of Geraniol and Nootkatone as fragrance oils and cholesteryl benzoate as a liquid crystal, our reaction is a useful entry to terpene‐loaded monomers for the synthesis of functional polymers.^[18]^ Strategies involving strong base (for example i) Wittig reaction of an aldehyde or ii) ketone reduction, mesylation and E_2_‐elimination) lead to hydrolysis or E_2_ elimination of the benzoate,^[19]^ while direct esterification suffers from the caveats that 4‐vinylbenzoic is thermally sensitive and formulated with BHT stabilizer. Further exemplifying utility, substrate **1 ah**, readily prepared from its α‐dichloroketone, underwent selective reduction to its unsymmetrical stilbene **2 ah** in good yield while leaving the olefinic Cl atom untouched (Scheme 3). This demonstrates the value of our method which retains reductively labile halides for further functionalizations. The method provides alternative access to unsymmetrical halogenated stilbenes that does not rely on transition metal catalysis.^[20]^ While conPET photocatalysis and e‐PRC are complementary approaches in the reductions of aryl halides/pseudohalides,[^3d^, ^3g^] conPET conditions did not effect the net‐reductive transformation herein (Scheme 4).

**Scheme 5:**
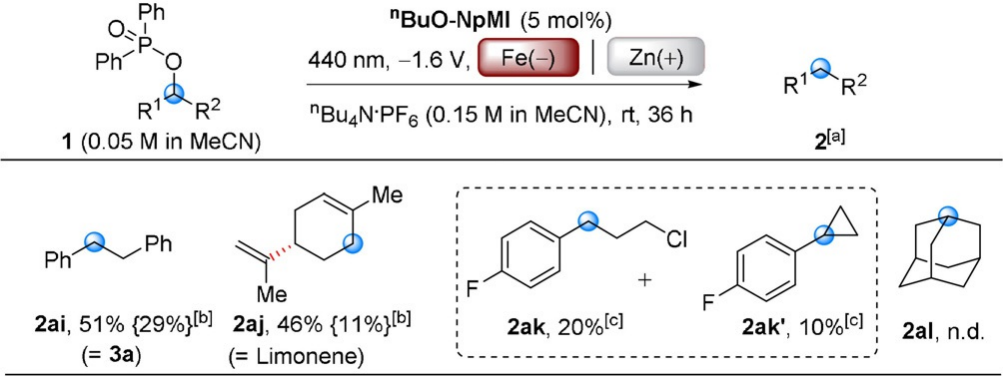
e‐PRCreductive deoxygenation. [a] Isolated yields of products **2 ai** and **2 aj**. [b] Yields in parentheses {} are ^1^H NMR yields from using **NpMI** as an e‐PRCat. [c] Yields of **2 ak** and **2 ak′** are determined by ^1^H NMR spectroscopy with 1,3,5‐trimethoxybenzene as an internal standard, identified by literature comparisons and GC‐MS traces.

At this juncture, we wondered if overall deoxygenation would be possible by removing the α‐Cl atom from **1 a** (**1 ai**) as the generated carbanion would be protonated. Photocatalytic deoxygenations of alcohols activated as their bis(trifluoromethyl)benzoates has been achieved with an iridium photocatalyst, but required stoichiometric Hantzsch ester, alkylamine and water at 40 °C.^[21]^ Direct electrolytic reduction of alkyl phosphinates is known, and required a constant current of 600 mA at 60–110 °C where a constant potential (*U*_cell_=−2.4 V vs. Ag/AgCl) was ineffective.^[22]^ Reductive functionality (styrenes, aryl halides, dienes, benzoates) would not tolerate these conditions. e‐PRC deoxygenation afforded desired product **1 ai** in good yield under standard conditions (*U*_cell_=−1.6 V) with extended time (Scheme 5). Allylic substrate **1 aj** smoothly deoxygenated to **2 aj** (Limonene). When a Cl atom was present β‐ to the phosphinate (**1 ak**), deoxygenation afforded **2 ak** and cyclopropane **2 ak′**, confirming the intermediacy of a benzylic carbanion (see **1 a′**, Scheme 1 c). An alkyl phosphinate derived from a non‐benzylic/allylic alcohol (**1 al**) did not react. We sought explanations as to two questions: 1) why e‐PRC conditions herein could not engage non‐benzylic substrates (**1 aa** and **1 al**, respectively) and 2) why ^**n**^ **BuO‐NpMI** was a superior e‐PRCat to **NpMI**; since **NpMI** as an e‐PRCat gave no conversion of various substrates (**1 f**, **1 n**, **1 o**, **1 q**, **1 u**) in olefinations (Scheme 2), and poor conversion of **1 ai** and **1 aj** in deoxygenations (Scheme 5).

Concerning the first question, measured reduction potentials (*E* ^p^_red_) of the alkyl phosphinates (in good agreement with those calculated by DFT) did not correlate with reactivity (Table 2). Instead, comparison of the C(sp^3^)−O bond‐dissociation free energies (BDFEs) of phosphinate radical anions correlated well with reactivity. This corroborated C(sp^3^)−O cleavage as the rate‐limiting step and rationalized i) the unique tolerance of our conditions to aryl halides due to their less exergonic C−X BDFEs (entries 4,5; 6,7) and ii) the lack of reactivity of phosphinates derived from non‐benzylic/allylic alcohols that require higher temperatures^[22]^ to assist C(sp^3^)−O cleavage (entries 9,10).

**Table 2:** Calculated properties of phosphinate radical anions vs. reactivity.

Entry	Radical anion	e‐PRCat	Product yield	BDFE	*E* ^p^_red_ [V]	
			[%]^[a]^	[kcal mol^−1^]^[b]^	Δ*E* ^calc.[c]^	Δ*E* ^exp.[d]^
1	**1 g**	**NpMI**	78 (**2 g**)	−39.8 _(C−O)_	−2.55	−2.47
2	**1 a**	**NpMI**	78 (**2 a**)	−39.2 _(C−O)_	−2.60	−2.23/ −2.34
3	**1 ai**	^**n**^ **BuO‐NpMI**	51 (**2 ai**)	−38.7 _(C−O)_	−2.62	ND
4	**1 o**	^**n**^ **BuO‐NpMI**	56 (**2 o**)	−38.1 _(C−O)_	−2.45	−2.60
5	**1 o**	^**n**^ **BuO‐NpMI**	5 (de‐Cl)	−26.9 _(C−Cl)_	–	−2.78^[f]^
6	**1 r**	^**n**^ **BuO‐NpMI**	39 (**2 r**)	−38.2 _(C−O)_	−2.44	−2.33/ −2.46
7	**1 r**	^**n**^ **BuO‐NpMI**	trace (de‐Br)	−30.6 _(C−Br)_	–	−2.44^[f]^
8	**1 d**	^**n**^ **BuO‐NpMI**	69 (**2 d**)	−34.5 _(C−O)_	−2.44	−2.41
9	**1 aa**	^**n**^ **BuO‐NpMI**	n.d. (**2 aa**)	−27.5 _(C−O)_	−2.40	−2.42
10	**1 al**	^**n**^ **BuO‐NpMI**	n.d. (**2 al**)	−22.1 _(C−O)_	−2.56	−2.68

[a] Product yields as defined in Scheme 2. [b] Bond dissociation free energies (Δ*G*) calculated at the ωB97X‐D/6–311+G*, IEFPCM(MeCN) theory level. [c] Calculated at the ωB97X‐D/6–311+G*, IEFPCM(MeCN) theory level and calibrated to an experimental set, see SI. [d] Measured at 10 mM [phosphinate] in 0.1 M ^n^Bu_4_N⋅PF_6_ in MeCN using Fc as an internal standard and calibrated vs. SCE, see SI. [f] Literature redox potentials of PhCl and PhBr are taken as surrogates.^[6]^

**Figure 1:**
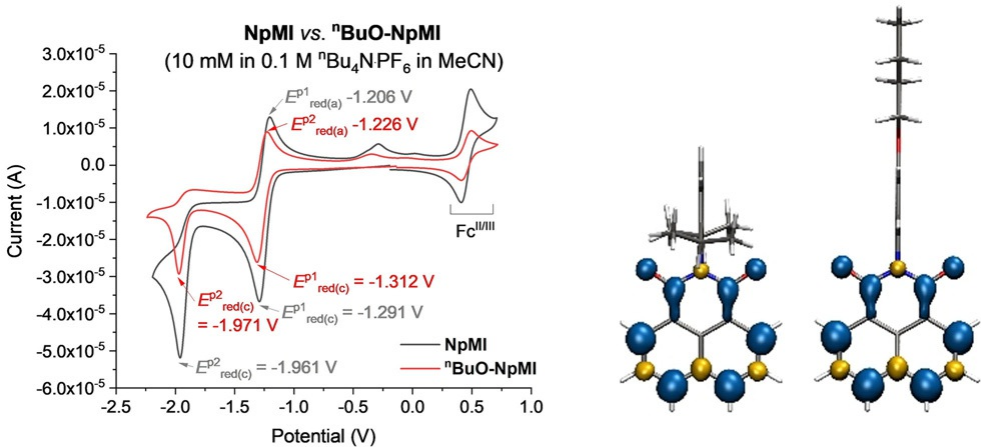
Cyclic voltammetry of e‐PRCats (10 mM [e‐PRCat] in 0.1 M ^n^Bu_4_N⋅PF_6_ in MeCN) vs. Ag/AgCl (left). DFT calculated spin densities (right) of **NpMI^.−^** and ^**n**^ **BuO‐NpMI^.−^**, see SI for details.

**Figure 2:**
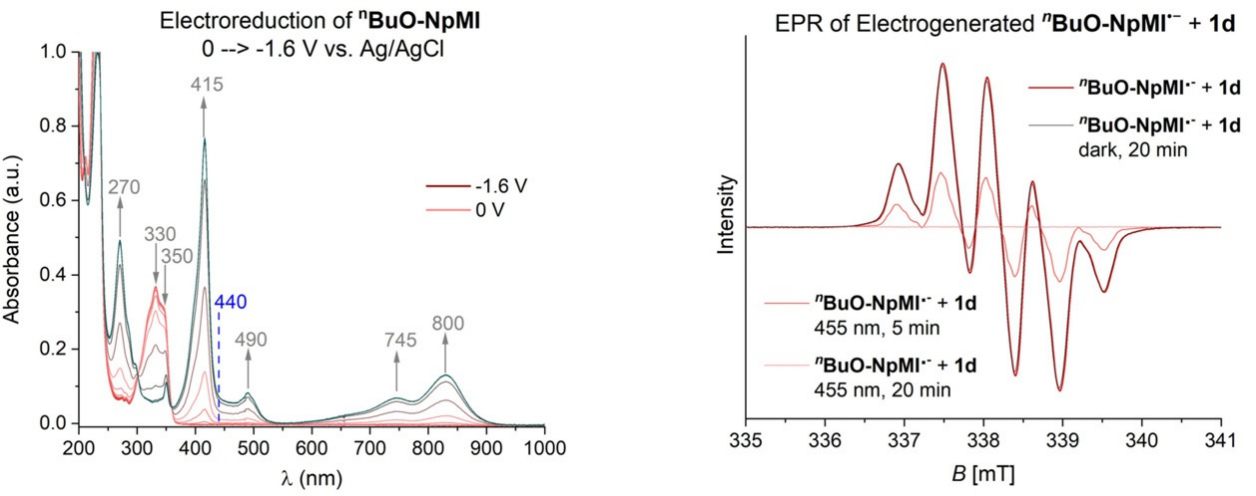
Spectroelectrochemistry of ^**n**^ **BuO‐NpMI** (2.5 mM in 0.1 M ^n^Bu_4_N⋅PF_6_ in MeCN) from 0 to −1.6 V vs. Ag/AgCl (left). EPR spectrum of electroreduced ^**n**^ **BuO‐NpMI** (2.5 mM in 0.1 M ^n^Bu_4_N⋅PF_6_ in MeCN at U_cell_=−1.6 V for 1 h) in the presence of **1 d** (10 equiv.) and signal quenching upon light irradiation (right).

As to the second question, **NpMI** and ^**n**^ **BuO‐NpMI** had identical redox potentials (*E*_1/2_=−1.3 V vs. SCE, Figure 1, left) by cyclic voltammetry. Their radical anions are electrogenerated with equal efficiency, which is entirely consistent with the spin densities of their radical anions (Figure 1, right) being localized on the naphthalene and being unaffected by substitution on the *N*‐aniline. Spectroelectrochemistry of both e‐PRCats gave identical UV‐vis bands for their radical anions (Figure 2, left and see SI). Taken together, these results indicate that their excited radical anions are equally potent reductants. To probe further, we electrochemically generated **NpMI^.−^** and ^**n**^ **BuO‐NpMI^.−^** under inert conditions for analysis by EPR (Figure 2, right).^[23]^ In both cases, a pentet was observed whose intensity was unchanged upon irradiation with blue LEDs. In both cases, in the presence of **1 d** (10 equiv.), the EPR signal was identical in the dark (see SI), but upon irradiation by blue LEDs the EPR signal quenched, corroborating successful SET from the doublet states (D_n_) of both catalysts ^2^[**NpMI^.−^** *] and ^2^[^**n**^ **BuO‐NpMI^.−^** *] to **1 d**. Given that the reaction of **1 d** is only successful with ^**n**^ **BuO‐NpMI^.−^** and taken together with the discussion of *E* ^p^_red_s and BDFEs in Table 2, this confirms SET is not the determining factor for the success of ^**n**^ **BuO‐NpMI^.−^**.

Neutral and electroreduced forms of **NpMI** and ^**n**^ **BuO‐NpMI** were probed by luminescence spectroscopy (Table 3). For neutral e‐PRCats, absorbance and emission (fluorescence) spectra corresponded with the literature.^[24]^ Measured lifetimes were *τ*≈3.0 ns in both cases. Although some *N*‐arylnaphthalimide derivatives have ultrashort‐lived singlet states, due to rapid intersystem crossing to triplet states,^[24]^ phosphorescence does not occur for the *N*‐aryl‐1,8‐naphthalimides where *N*‐aryl rotation becomes considerably hindered.^[24]^ Electroreduction for 1 h and selective excitation of the radical anions at 452 nm led to a new emission band (*λ*_max_ ca. 540 nm) and a longer‐lived species with biexponential decay (*τ*_1_≈7 ns and *τ*_2_≈20 ns) for both **NpMI^.−^** and ^**n**^ **BuO‐NpMI^.−^**. The doublet (D_1_) states of similar radical anions (naphthalene diimide radical anions, perylene diimide radical anions) are picosecond‐lived and do not luminesce,^[25]^ and we confirmed by excitation spectra (see SI) that this emission was not deriving from the initially‐formed excited state ^2^[^**n**^ **BuO‐NpMI^.−^** *] (Figure 2, left), but from a lower‐lying, longer lived excited state, termed “ES_1_”. Intersection of the longest wavelength excitation and shortest wavelength emission bands allows an estimation of *E* ^0–0^ for photoexcited states.^[26]^ For these emitting excited states, estimated *E* ^0–0^ values (*E* ^ES^) for both [**NpMI^.−^** *] and [^**n**^ **BuO‐NpMI^.−^** *] were (*E* ^ES^=56.6 kcal mol^−1^) almost identical to the triplet energies (*E* ^T^) of *Ir^III^ photosensitizers used in olefin photoisomerisms.[^9a^, ^9b^, ^9c^] It is therefore reasonable to propose *E*‐/*Z*‐ photoisomerism occurs via energy transfer (E_n_T) from ES_1_. E_n_T would be exergonic to *E*‐stilbene and less so to *Z*‐stilbene (*E* ^T^=51.0 vs. *E* ^T^=55.5 kcal mol^−1^, respectively), rationalizing high *Z*‐stilbene selectivity.[^9b^, ^9c^, ^27^] However, the lifetime of ES_1_ was unchanged in the presence of **1 d** (10 equiv.), confirming its catalytic inactivity in the initial SET step.

**Table 3:** Lifetimes of neutral and electroreduced^[a]^ e‐PRCats.

Entry	e‐PRCat	Conditions	*λ*_max_ (ex)/ *λ*_max_ (em)	*τ* [ns]	*E* ^S/ES1^ [kcal mol^−1^]
1	**NpMI**	–	375/412	*τ*=3.2	(S_1_) 75.4
2	**NpMI**	−1.6 V, 1 h^[a]^	452/535	*τ*_1_=5.4 *τ*_2_=21.7	(ES_1_) 56.6
3	^**n**^ **BuO‐NpMI**	–	375/412	*τ*=3.2	(S_1_) 75.6
4	^**n**^ **BuO‐NpMI**	−1.6 V, 1 h^[a]^	452/548	*τ*_1_=6.8 *τ*_2_=19.5	(ES_1_) 56.6
5	^**n**^ **BuO‐NpMI**	−1.6 V, 1 h^[a]^ +10 equiv. **1 d**	452/548	*τ*_1_=8.1 *τ*_2_=20.3	–

[a] Electroreduced e‐PRCat (2.5 mM in MeCN (0.1 M ^n^Bu_4_N⋅PF_6_), diluted 8×.

In their study of photoexcited benzo[*ghi*]perylenemonoamide (BPI) radical anions for Birch reductions, Miyake and co‐workers made similar observations.^[14]^ They assigned the long‐lived excited state as the lowest‐lying quartet excited state (^4^BPI**^.−^** *) arising from intersystem crossing (ISC) from the doublet state (^2^BPI**^.−^** *). Therefore, the lowest‐lying quartet state ^4^[^**n**^ **BuO‐NpMI^.−^** *] is a candidate for ES_1_, that allows E_n_T to be spin‐conserved. We calculated the vertical excitation energy of this lowest quartet state with CASSCF (see SI) and found a reasonable agreement with the observed *λ*_max_ of luminescence. It is energetically close to the doublet states underlying the 415 nm absorption band so that ISC is plausible.

Miyake similarly found that the putative ^4^BPI**^.−^** * was not catalytically active in the Birch SET step. They hypothesized SET from a higher lying excited doublet state ^2^BPI**^.−^** * (D_n_) in an anti‐Kasha fashion. Consistent with previously reported anti‐Kasha photochemistry of doublet excited state photocatalysts,[^5a^, ^14^] excitation of the broad absorption of ^2^[^**n**^ **BuO‐NpMI^.−^** *] between 650–900 nm (D_0_→D_1_) with 740 nm or 850 nm LEDs gave only traces of **2 d**.^[28]^ Ruling out participation of the first excited state (D_1_), “effective minimum” potentials (*E* ^0^_1/2_) of **NpMI^.−^** * (D_n_) at −3.7 V vs. SCE and ^**n**^ **BuO‐NpMI^.−^** *(D_n_) at −3.8 V vs. SCE can be calculated by previously described methods,^[29]^ easily reaching *E* ^p^_red_ of all phosphinates herein as well as aryl halides.[^30^, ^31^] Participation of a doublet excited state in SET is consistent with aforementioned quenching of the EPR signal (Figure 2).

**Figure 3:**
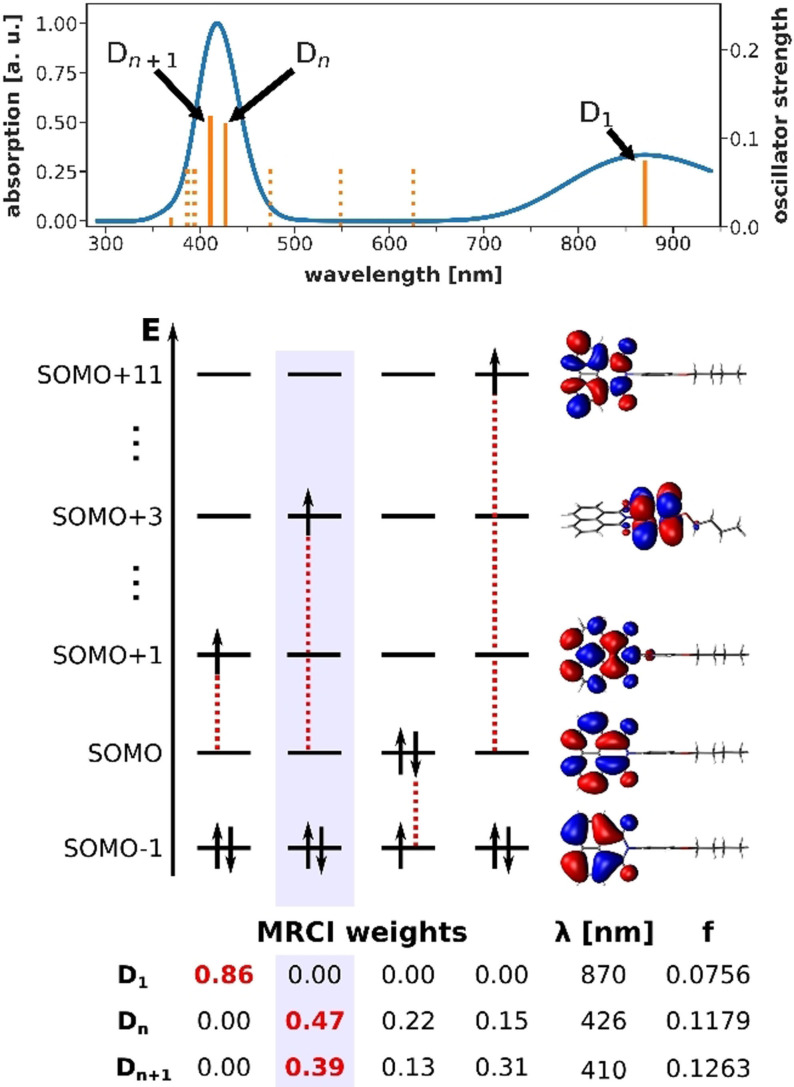
Calculated DFT/MRCI absorption spectrum for ^**n**^ **BuO‐NpMI^.−^** (top). Dark states with oscillator strengths *f*<0.01 are indicated by dotted orange lines. Leading electronic configurations for the bright excited states D_1_, D_*n*_ and D_*n*+1_ (bottom). Dotted red lines indicate single electron excitations from the ground state configuration.

High‐level DFT/MRCI calculations were carried out for ^**n**^ **BuO‐NpMI^.−^** to characterize this D_n_ state. The computed spectrum (Figure 3, top) is in excellent agreement with the experimental absorption spectrum, especially at the band with *λ*_max_=415 nm comprising two bright π–π* states (D_0_→D_n_ and D_0_→D_n+1_). Contrary to the D_0_→D_1_ transition around 870 nm, both these excitations transfer electron density from the naphthalene to the *N*‐aniline unit of ^**n**^ **BuO‐NpMI^.−^** (Figure 3, bottom). Preassembly of ground state radical anion and substrate could explain (i) photochemistry of ultrashort‐lived doublet states^[25]^ and (ii) faster than rates of diffusion.^[5a]^ Preassembly of ^**n**^ **BuO‐NpMI^.−^** with **1 d** being more favorable than that of **NpMI^.−^** may explain the reactivity differences of the e‐PRCats in effecting C(sp^3^)−O cleavage following SET, and may rationalize profound shift in the molecular site of reduction compared to previous reports.^[32]^ However, like Miyake and co‐workers, we were unable to find spectroscopic evidence of preassembly by UV‐vis or EPR (see SI). While the absence of spectroscopic perturbations does not rule out a preassociation,^[33]^ preassembly could occur at the *N*‐aniline that is spin‐disconnected from the naphthalene where the radical anion spin density is localized (Figure 1, right). Spin densities of favorable candidate preassemblies at the *N*‐aniline unit of ^**n**^ **BuO‐NpMI^.−^** found by computational geometry optimizations do not differ from that of ^**n**^ **BuO‐NpMI^.−^** alone, while a favorable candidate preassembly at the naphthalene unit of ^**n**^ **BuO‐NpMI^.−^** does differ (see SI). A preassembly at the *N*‐aniline could also rationalize anti‐Kasha photochemistry, since charge transfer to the *N*‐aniline in the D_n/*n*+1_ states is proximal to the bound substrate and promotes intermolecular SET upon photoexcitation (Figure 3). In contrast, the charge density of the lowest excited doublet state D_1_ remains localized on the naphthalene and is not close to the substrate.

**Scheme 6:**
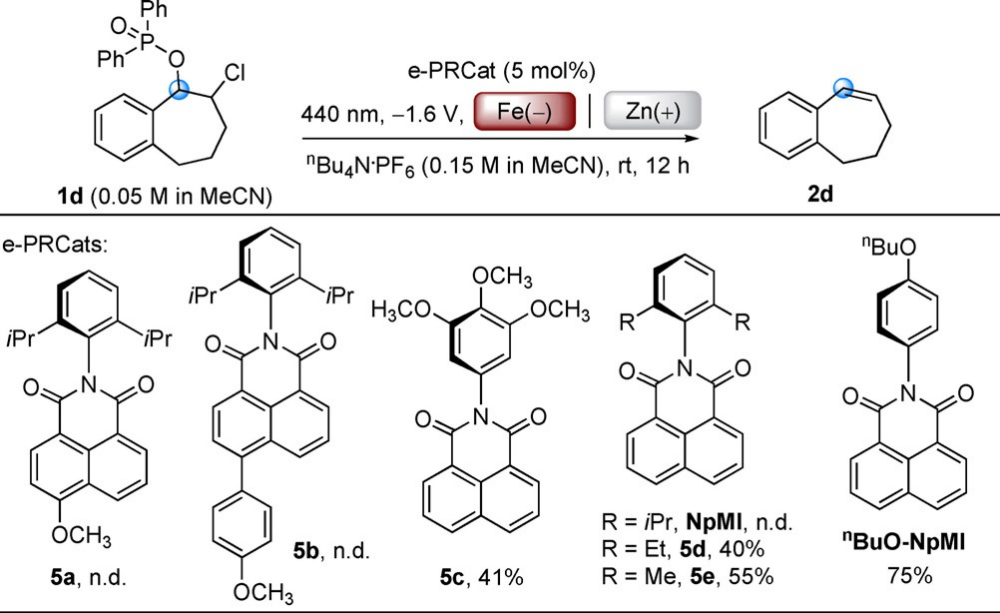
e‐PRC deoxygenation of **1 d** with various e‐PRCats. Yields of **2 d** determined by ^1^H NMR spectroscopy with 1,3,5‐trimethoxybenzene as an internal standard.

Where spectroscopy offers little insight, a top‐down approach varying catalyst structure and examining product yields has proven useful in investigating the mechanisms of reactions involving *in situ*‐formed organic electron donors.^[34]^ To probe the importance of a preassembly of **1 d** at the *N*‐aniline of the e‐PRCat, we explored the influence of a series of e‐PRCats with varying electronics and steric bulk (**5 a**–**f**, Scheme 6). Compared to **NpMI**, catalysts with electron donating alkoxy or *p*‐anisole substituents on the naphthalene unit (**5 a**, **5 b**) gave no reaction. Compared to ^**n**^ **BuO‐NpMI**, a catalyst with additional alkoxy substituents on the *N*‐aniline (**5 c**) gave a lower (41 %) yield of **2 d**. The yield of **2 d** increased with decreasing steric hindrance at the *ortho*‐positions of the *N*‐aniline (**NpMI**≪**5 d**<**5 e**).^[35]^ A decrease in “steric bulk” likely promotes preassociation of radical anion e‐PRCat and **1 d**. In our computational investigations we found multiple stable ground state preassemblies. Geometry optimizations (see SI) converged to pincer‐like conformations for all candidates, where two of the substrate's aryl groups coordinate to the *N*‐aniline of the e‐PRCat in a T−π and π−π orientation, respectively. The thermodynamics and kinetics of their formations (see SI) mirror reactivity trends in Scheme 6, corroborating a preassembly between e‐PRCat and substrate before photoexcitation.

## Conclusion

We report an electro‐mediated photoredox catalytic reductions of phosphinates derived from α‐chloroketones toward selective olefinations and deoxygenations. This study reports reductive formation of alkyl carbanions via photoexcited radical anions as super‐reductants. The selective reduction of C(sp^3^)−O bonds in the presence of C(sp^2^)−X bonds was achieved. Reactivity differences of various radical anion photocatalysts and anti‐Kasha photochemistry, backed by computational insights, suggest the importance of a close catalyst‐substrate interaction for an effective, selective reaction. In this context, our calculations indicate that intramolecular charge transfer in the catalyst radical anion upon photoexcitation promotes SET to the substrate. Photocatalyst‐substrate preassemblies such as EDA complexes,^[36]^ non‐covalent interactions,[^5a^, ^37^] hydrogen bonding^[38]^ and ordering of solvent^[39]^ are receiving increasing attention to unveil the next generation of photocatalytic transformations and offer new frontiers in selectivity and efficiency. Further studies into the nature of interactions and structure of preassemblies, as well as catalyst stability,^[40]^ are ongoing.

## Conflict of interest

The authors declare no conflict of interest.

## Supporting information

As a service to our authors and readers, this journal provides supporting information supplied by the authors. Such materials are peer reviewed and may be re‐organized for online delivery, but are not copy‐edited or typeset. Technical support issues arising from supporting information (other than missing files) should be addressed to the authors.

Supporting Information
