# Supplementary material for: Electro‐mediated PhotoRedox Catalysis for Selective C(sp3)–O Cleavages of Phosphinated Alcohols to Carbanions
Source: Angew Chem Int Ed Engl. 2021 Aug 16;60(38):20817–25. doi: 10.1002/anie.202105895 (PMC8518744; doi:10.1002/anie.202105895)
Supplement: Supplementary file 1 — Supporting Information [file ANIE-60-20817-s001.pdf]

Angewandte Chemie
Eine Zeitschrift der Gesellschaft Deutscher Chemiker

Supporting Information

Electro-mediated PhotoRedox Catalysis for Selective C(sp^3)–O Cleavages of Phosphinated Alcohols to Carbanions

Xianhai Tian, Tobias A. Karl, Sebastian Reiter, Shahboz Yakubov, Regina de Vivie-Riedle, Burkhard König, and Joshua P. Barham**

anie_202105895_sm_miscellaneous_information.pdf

SUPPORTING INFORMATION FILE

TABLE OF CONTENTS

1. General Experimental Information ... 2
2. Materials and Electrodes Preparation ... 3
3. Synthesis of Catalysts ... 5
4. Synthesis of Phosphinate Substrates ... 7
5. Optimization of Reaction Conditions ... 10
6. Photoelectrochemical Isomerism Control Experiments ... 11
7. Characterization Data: Catalysts ... 13
8. Characterization Data: Phosphinate Substrates ... 15
9. Characterization Data: e-PRC Phosphinate Reduction Products ... 31
10. Cyclic Voltammetry ... 41
11. Direct Electrolysis Experiments ... 44
12. Spectroelectrochemistry of Catalysts ... 44
13. UV-vis Spectroscopy of Electrogenerated Radical Anions ... 45
14. Luminescence Spectroscopy of Catalysts and Electroreduced Catalysts ... 47
   14.1. Steady-state Luminescence Emission Spectroscopy ... 47
   14.2. Lifetime measurements ... 49
15. Spectroscopic Investigations of Preassociation ... 52
   15.1. NMR spectroscopy ... 52
   15.2. FT-IR Spectroscopy ... 56
16. Emission spectra of LEDs ... 58
17. Electron Paramagnetic Spectroscopy Investigations ... 61
18. Computational Investigations ... 65
19. XYZ Co-ordinates of computed structures ... 78
20. ^{1}H NMR, ^{13}C NMR and ^{19}F NMR Spectra of The Compounds Prepared ... 138
21. X-ray Crystallography ... 216
22. References ... 220

1. General Experimental Information

Unless stated otherwise, reactions were carried out under an inert (N_2) atmosphere. Cryogenic conditions (-78 °C) were achieved using dry ice/acetone baths. Temperatures of 0 °C were obtained by means of an ice bath or ice/salt bath. 'Room temperature' (rt) indicates temperatures in the range of 20-25 °C. For purposes of thin layer chromatography (TLC), ALUGRAM® Xtra SIL G/UV_{254} silica plates were used, with UV light ($\lambda = 254$ nm), near-UV light ($\lambda = 366$ nm) and potassium permanganate used for visualization. Purification was achieved by column chromatography using Macherey-Nagel silica gel 60 (0.063-0.2 mm) or Merck silica gel 60 (0.040-0.063 mm, 230-440 mesh). Removal of solvents (in vacuo) was achieved using Heidolph rotary evaporators or Vacuubrand high vacuum pumps.

All NMR data were collected using a Bruker Avance 400 Ultrashield instrument (400 MHz, 376 MHz, 162 MHz and 101 MHz for ^{1}H, ^{19}F, ^{31}P and ^{13}C NMR), or a Bruker Avance 300 Ultrashield instrument (300 MHz, 282 MHz, 162 MHz and 75 MHz for ^{1}H, ^{19}F, ^{31}P and ^{13}C NMR) was used. ^{13}C NMR was run in ^{1}H-decoupled mode. Data were manipulated using MestReNova version 12.0.0. Multiplicities for coupled signals were denoted as: s = singlet, d = doublet, t = triplet, q = quartet, quint = quintet, sext = sextet, hept = heptet, dd = doublet of doublets, ddd = doublet of doublets of doublets, td = triplet of doublets, qd = quartet of doublets, m = multiplet, br. = broad, apt. = apparent. Coupling constants (*J*) are given in Hz and are uncorrected. Where appropriate, COSY, DEPT, HSQC and HMBC experiments were carried out to aid assignment.

Infra-red measurements of electrogenerated radical anions were recorded on a Bruker Tensor 27 FT-IR Spectrophotometer (courtesy of Prof. Dr. Patrick Nürnberger's group) as a thin film of solution between NaCl discs unless otherwise stated.

Preparation of electrogenerated radical anions was done either using a MBRAUN Unilab Plus glovebox (courtesy of Prof. Dr. Manfred Scheer's group) or an MBRAUN UNIlab glovebox (courtesy of Prof. Dr. Robert Wolf's group) under an N_2 atmosphere.

UV-visible absorption measurements were performed either using an Ottle Cell Chrono (optically transparent thin-layer electrochemical cell with a path length of 0.02 cm, see Section S12 for details on spectroelectrochemistry), or using a gastight quartz cuvette (path length of 1.0 cm), within an Agilent 8453 spectrometer unless otherwise stated. Online UV-vis measurements were performed within the same spectrometer, using an ISMATEC ISM930C dosing pump to continuously pump the catholyte (1 mL/min) through a quartz flow cell (path length of 1.0 cm).

EPR spectra of electrogenerated radical anions were measured on a Magnettech Miniscope MS 400 spectrometer (9.45 GHz) at 20 °C (see Section S17 for EPR studies).

High Resolution Mass spectral analyses were carried out in EI or ESI mode on a Finnigan MAT 95, Thermo Quest Finnigan TSQ 7000, Finnigan MATSSQ 710 A or an Agilent Q - TOF 6540 UHD instrument, masses observed are accurate to within ±5 ppm. Melting points are uncorrected and were

recorded using a Stuart melting point device up to 300 °C. All solvents and reagents were purchased from Sigma-Aldrich and used as supplied. All solvents and reagents were used as supplied or purified using standard techniques.[1]

As electrolyte in all preparative reactions and spectroscopic studies, *n*-tetrabutylammonium hexafluorophosphate ($^nBu_4N{\cdot}PF_6$ or 'TBAP' (98%+, TCI Chemicals)) was used as purchased (see Section S11 for cyclic voltammetry studies). Reactants and reagents were purchased at the highest commercial quality and used as received, from TCI, Sigma-Aldrich, Fischer Scientific, Fluorochem or ChemPur. All other solvents and reagents were used as supplied or purified using standard techniques.[1]

2. Materials and Electrodes Preparation

LEDs (Section S16 for characterization details):

365 nm: CCS (Creating Customer Satisfaction) Inc. (LDL-71X12UV12-365-N); 400 nm ("weak"): Edison EDEV-SLC1-03; 400 nm ("strong"): LED Engin (LZ440UB00-00U4); 440 nm: LED-TECH (OSRAM Oslon SSL 80: LT-1960); 530 nm ("strong"): CREE XP-E2 Q4 LT2496; 530 nm ("weak"): LED-TECH (OSRAM Oslon SSL 80: LT-1966); 740 nm: LED Engin (LZ4-00R308); 850 nm: LED Engin (LZ4-00R608).

Materials:

Eluteng (12 V, 1 A) USB cooling fan (purchased from Amazon); Faber-Castell 2.0 mm 2B pencil lead (purchased from Amazon); PeakTech® 6080A digital DC power supply; Carbon felt electrode purchased from Alfa Aesar (1.27 cm, thick, 99.0%); Glassy Carbon Foam electrode, thickness: 6.35 mm, porosity: 96.5% (Goodfellow, Product Code: 613-422-20); Fe Cathode was manufactured in house from DC01 CR1 (C 0.12, P 0.045, S 0.045, Mn 0.60, Ti 0.0) by Thyssen Krupp; Cu Cathode was manufactured in house from CW021A (Cu min 99.5, Bi 0.0005, P 0.002-0.007, Pb 0.005) by Batz + Burgel oHG; Zinc Anode (for electroplating) purchased from MARAWE (part01-74-00000) (200 x 170 x 0.6 mm).

Fabrication of divided H-cells was done by in-house by a glassblower. A built-in porous filter disc (Ø 1 cm, max. pore size 16 -40µm) was purchased from Duran®.

Set-up of divided cell for photoelectrochemical reactions followed a method previously reported[2] and is summarized in Figure S1.

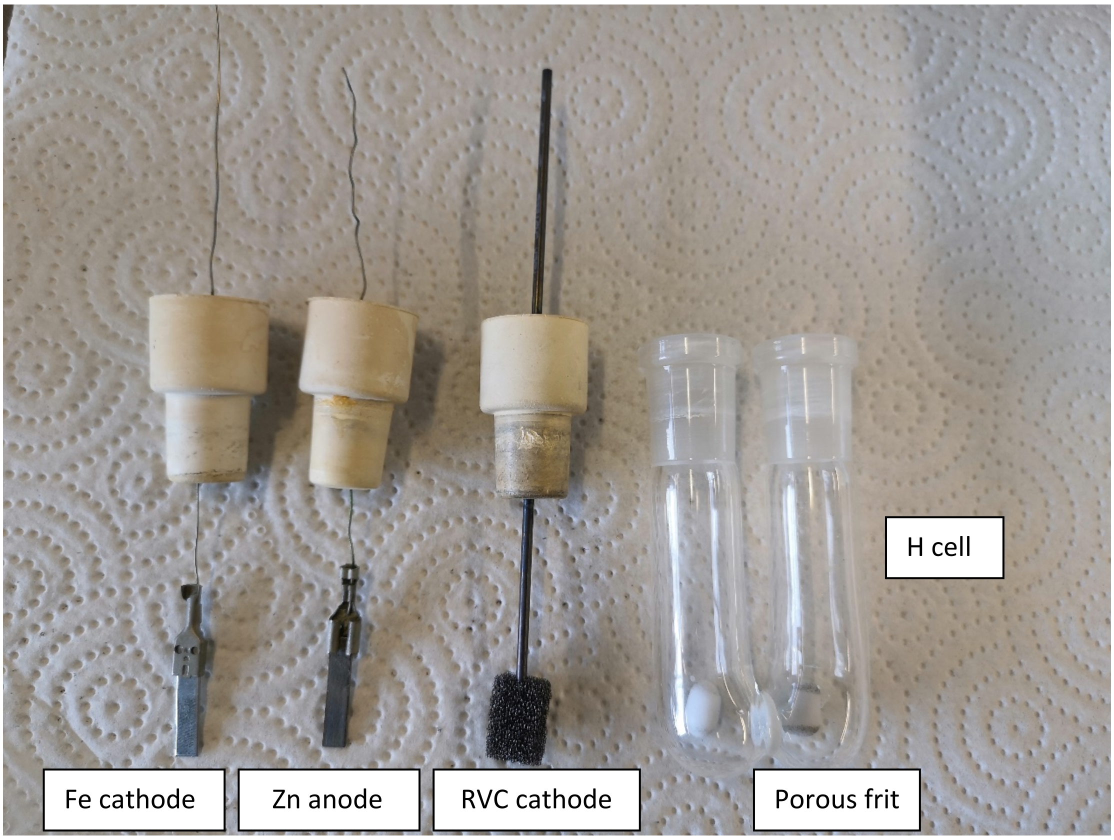

Figure S1. Electrode and H cell setup.

Cathode (RVC foam) set-up: A 2B pencil lead was inserted through a septum with the help of a needle. A small square (around 7 mm x 7 mm) of carbon foam was cut from the carbon foam plate, and the pencil lead was pierced through the resulting foam cube.

Cathode (Fe) set-up: A rectangular metal cathode (*ca.* 20 mm x 4 mm) was inserted into a conductive steel holder. With the help of a needle, the holder was inserted through a septum.

Anode (Zn) set-up: A rectangular metal cathode (*ca.* 20 mm x 4 mm) was inserted into a conductive steel holder. With the help of a needle, the holder was inserted through a septum.

The assembled H cell, after charging with reactants, reagents and solvent and degassing via N_2 bubbling for 10 min, was stirred at room temperature above a water-cooled cooling block under irradiation of a 440 nm LED from beneath the cathodic chamber (Figure S2). For the photoelectrochemical reaction procedure, see **Procedure 11**.

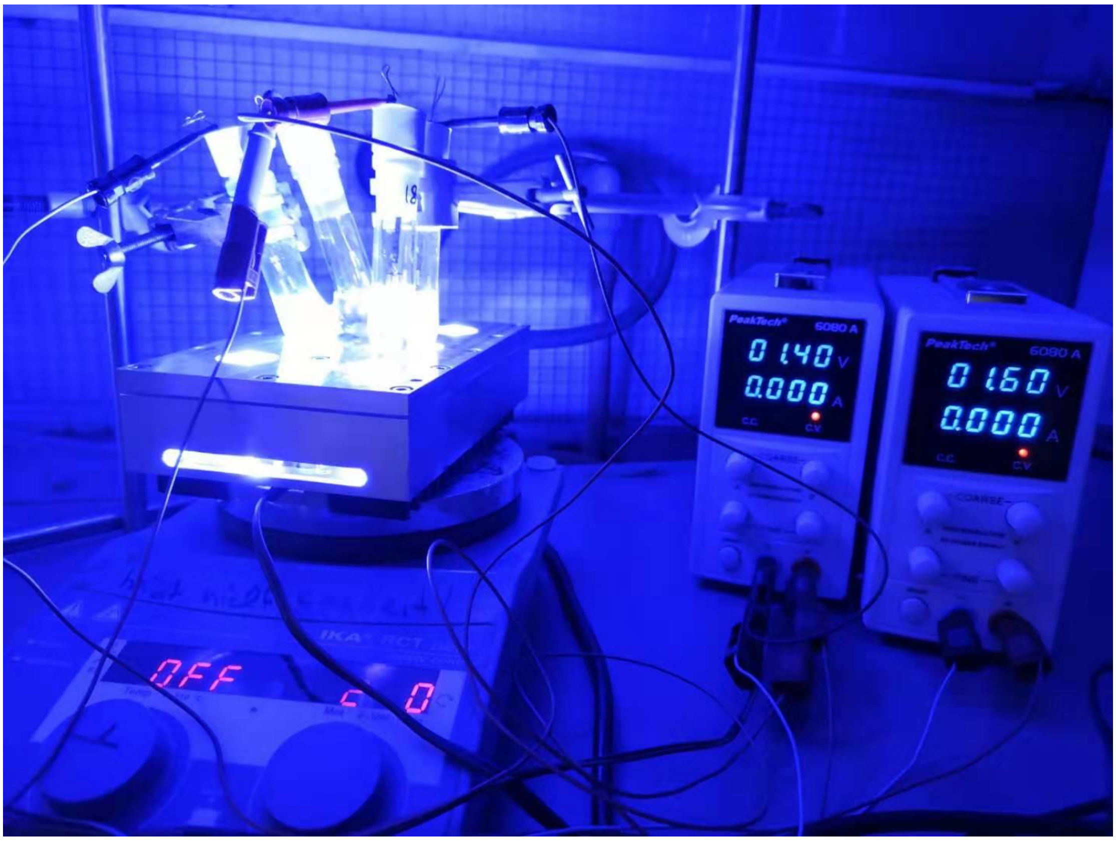

Figure S2. Photoelectrochemical reaction setup.

3. Synthesis of Catalysts

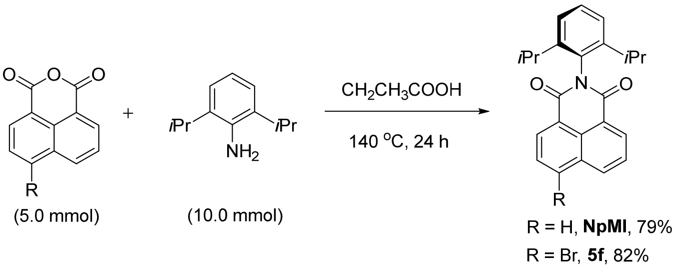

NpMI and **5f** were prepared according to a literature procedure.[3]

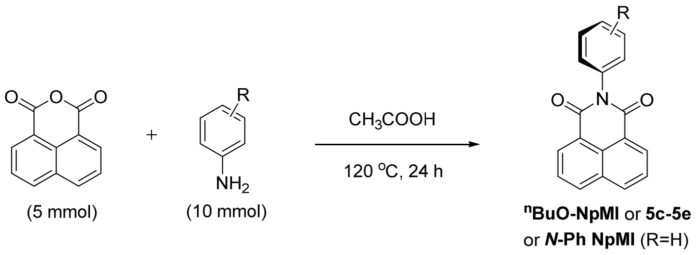

Procedure 1: ⁿBuO-NpMI, **5c-5e** and *N*-Ph **NpMI** were prepared according to a modified literature procedure.[3] A mixture of 1,8-naphthalic anhydride (5.0 mmol, 1.0 eq.) and substituted aniline (10.0 mmol, 2.0 eq.) in CH_3COOH (25 mL) was charged to a pressure tube (50 mL in volume) with a stirrer bar and was heated to 120 °C. After being stirred at this temperature for 24 h, the reaction mixture

was cooled down to rt and the precipitate collected by filtration. The solid was then washed with Et_2O (5 × 5 mL) and dried under vacuum to afford the pure product as a solid.

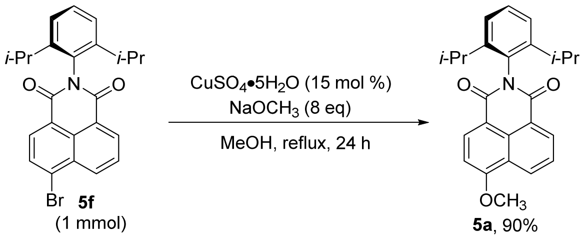

Procedure 2: 5a was prepared according to a modified literature procedure.[4] A mixture of **5f** (435 mg, 1.0 mmol), sodium methoxide (432 mg, 8.0 mmol) and copper sulfate (37.5 mg, 0.15 mmol) in anhydrous methanol (5 mL) was refluxed under N_2 for 24 h. After cooling, solvent was evaporated under vacuum and the residue was dissolved in DCM (20 mL). The resulting mixture was sequentially washed by H_2O (20 mL), sat. aq. NaCl solution (20 mL), then dried over Na_2SO_4. The organic layer was evaporated under vacuum and the residue was purified by column chromatography on silica gel using a mixture of pentane and EtOAc as eluent to afford pure product **5a** in 90% yield as a pale yellow solid.

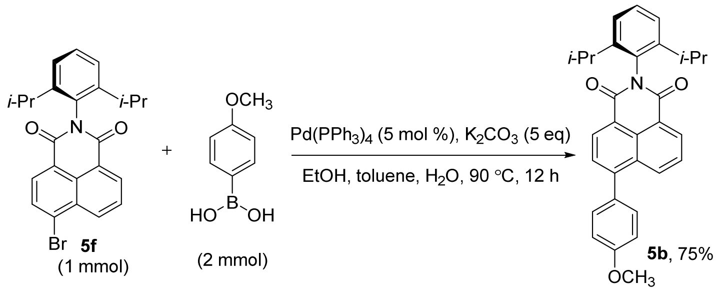

Procedure 3: 5b was prepared according to a modified literature procedure.[5] **5f** (435.0 mg, 1.0 mmol), 4-methoxyphenylboronic acid (304.0 mg, 2.0 mmol), $Pd(PPh_3)_4$ (58.0 mg, 0.05 mmol) and K_2CO_3 (690.0 mg, 5.0 mmol) were added to a mixture of toluene (12 mL), water (7 mL) and EtOH (5 mL). The resulting mixture was bubbled with N_2 for 10 min and then was heated at 90 °C for 12 h. After cooling, the organic solvents were removed under vacuum. DCM (20 mL) and H_2O (15 mL) were added to the residue. The organic layer was separated, and the aqueous layer was extracted with DCM (2 × 20 mL). The combined organic layers were dried over Na_2SO_4 and evaporated. The residue was purified by column chromatography on silica gel using a mixture of pentane and EtOAc as eluent to afford pure product **5b** in 75% yield as a yellow solid.

4. SYNTHESIS OF PHOSPHINATE SUBSTRATES

Preparation of phosphinate **1a**

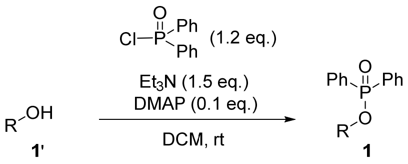

Procedure 4: To a mixture of alcohols (3.0 mmol, 1.0 eq.) and Et_3N (4.5 mmol, 1.5 eq.) in 20 mL anhydrous DCM was added diphenylphosphinic chloride (3.6 mmol, 1.2 eq.) dropwise followed by the addition of 4-dimethylamino pyridine (0.3 mmol, 0.1 eq.). The reaction mixture was stirred at rt overnight. The solvent was then removed under vacuum and the residue was purified by flash column chromatography using a mixture of pentane and EtOAc to give the desired phosphinates.

One-pot synthesis of phosphinates from α-halogenated ketones **1”**

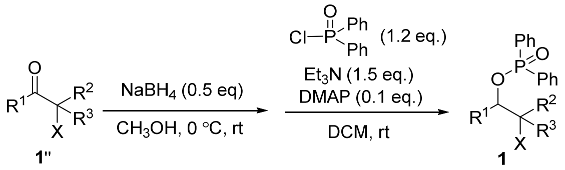

α-Halogenated ketones **1m”**, **1r”-1s”**, **1y”-1z”** are commercially available. Others were easily prepared using NXS (X = Cl or Br) according to literature procedures.[6]

Procedure 5: At 0 °C, to a solution of α-halogenated ketone (3.0 mmol, 1.0 eq.) in 20 mL MeOH was added $NaBH_4$ (57.0 mg, 0.5 eq.) in 2 portions. The reaction mixture was stirred at 0 °C for 10 min and at room temperature for 2 h. Solvent was removed under vacuum and 10 mL H_2O was added to the residue. The resulting mixture was then extracted with DCM (3 × 10 mL). The organic layers were dried over Na_2SO_4 and evaporated. At 0 °C, the residue was treated with 20 mL anhydrous DCM, Et_3N (4.5 mmol, 1.5 eq.), diphenylphosphinic chloride (3.6 mmol, 1.2 eq.) and 4-dimethylamino pyridine (0.3 mmol, 0.1 eq.). The reaction mixture was stirred at room temperature overnight. Solvent was removed under vacuum and the residue was purified by flash column chromatography using a mixture of pentane and EtOAc to give the desired phosphinates.

Preparation of adamantane-based phosphinate **1al**

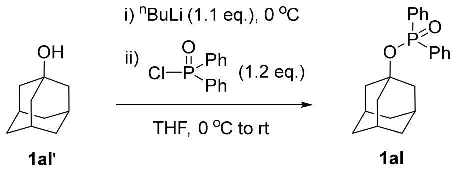

Procedure 6: Prepared according to a literature procedure.[7] At 0 °C, $^{n}BuLi$ (1.6 M in hexane, 5.5 mmol, 1.1 eq.) was added dropwise to a solution of alcohol (5.0 mmol, 1.0 eq.) in 20 mL anhydrous THF under N_2 atmosphere. The resulting mixture was stirred at 0 °C for 30 min, followed by the

addition of diphenylphosphinic chloride (6.0 mmol, 1.2 eq.) was added dropwise. Stirring continued for 1 h at 0 °C and then the reaction was allowed to warm to rt and stirred for 12 h. Afterwards, the mixture was quenched by sat. aq. NH_4Cl (10 mL). THF was removed under reduced pressure and the resulting mixture was extracted with DCM (3 × 10 mL). The combined organic layers were washed with water, dried over anhydrous sodium sulfate and the solvent was evaporated under reduced pressure. The residue was purified by column chromatography on silica gel using a mixture of pentane and EtOAc as eluent to afford pure product **1al** as a white solid.

One-pot preparation of phosphinates from α-chloro ketones

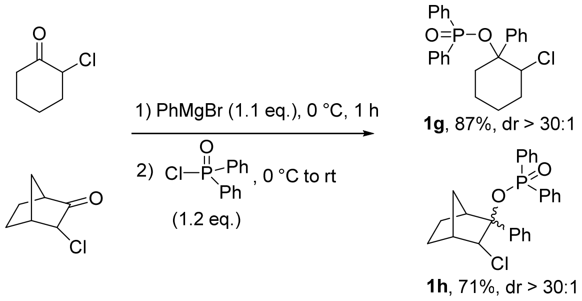

Procedure 7: At 0 °C, PhMgBr (1.0 M in THF, 5.5 mmol, 1.1 eq.) was added dropwise to a solution of α-chloro ketone (5.0 mmol, 1 eq.) in 20 mL anhydrous THF under N_2 atmosphere. The resulting mixture was stirred at 0 °C for 1 h, followed by the addition of diphenylphosphinic chloride (6.0 mmol, 1.2 eq.). Stirring continued for 1 h at 0 °C and then for 12 h at room temperature. Afterwards, the mixture was quenched by sat. aq. NH_4Cl (10 mL). THF was removed under reduced pressure and the resulting mixture was extracted with DCM (3 × 10 mL). The combined organic layers were washed with water, dried over anhydrous sodium sulfate and the solvent was evaporated under reduced pressure. The residue was purified by column chromatography on silica gel using a mixture of pentane and EtOAc as eluent.

One-pot preparation of phosphinate **1aa** from epichlorohydrine

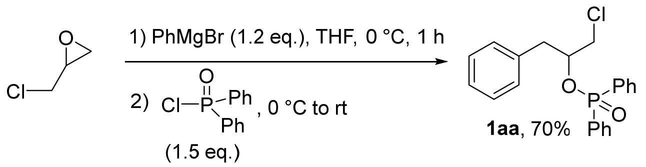

Procedure 8: At 0 °C, to a solution of PhMgBr (6.0 mmol in 20 mL anhydrous THF, 1.2 eq.) was added dropwise epichlorohydrine (5.0 mmol, 1 eq.) under N_2 atmosphere. The resulting mixture was stirred at 0 °C for 1 h, followed by the addition of diphenylphosphinic chloride (7.5 mmol, 1.5 eq.). Stirring continued for 1 h at 0 °C and then for 12 h at room temperature. The mixture was then quenched by sat. aq. NH_4Cl (10 mL). THF was removed under reduced pressure and the resulting mixture was extracted with DCM (3 × 10 mL). The combined organic layers were washed with water, dried over anhydrous sodium sulfate and the solvent was evaporated under reduced pressure. The residue was purified by column chromatography on silica gel using a mixture of pentane and EtOAc as eluent to afford product **1aa** as a white solid.

One-pot preparation of phosphinates **1ab-1ac** from chloroiodomethane

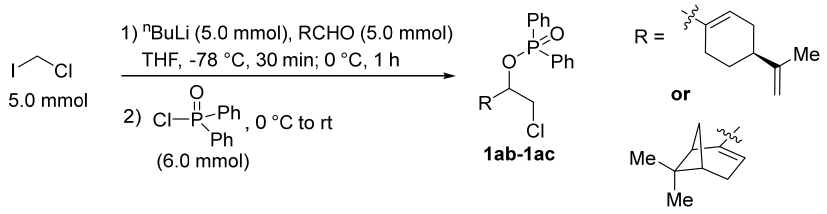

Procedure 9: At -78 °C, to a mixture of chloroiodomethane (5.0 mmol, 1 eq.) and aldehyde (5.0 mmol, 1 eq) in 20 mL anhydrous THF was added dropwise ^nBuLi (1.6 M in hexane, 5.0 mmol, 1.0 eq) under N_2 atmosphere. The resulting mixture was stirred at -78 °C for 30 min and then was allowed to warm to 0 °C for 1 h, followed by the addition of diphenylphosphinic chloride (6.0 mmol, 1.2 eq). Stirring continued for 1 h at 0 °C and then for 12 h at room temperature. The mixture was then quenched by sat. aq. NH_4Cl (10 mL). THF was removed *in vacuo* and the resulting mixture was extracted with DCM (3 × 10 mL). The combined organic layer was washed with water, dried over anhydrous sodium sulfate and the solvent was evaporated *in vacuo*. The residue was purified by column chromatography on silica gel using a mixture of pentane and EtOAc as eluent to afford the desired product.

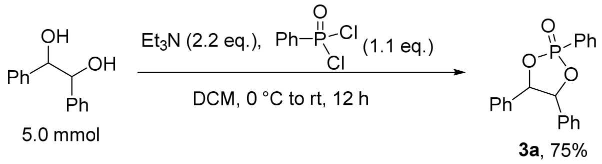

Procedure 10: At 0 °C, to a stirred mixture of diol (10.0 mmol, 1.0 eq.) and Et_3N (22.0 mmol, 2.2 eq.) in 50 mL anhydrous DCM was added dropwise phenylphosphonic dichloride (11.0 mmol, 1.1 eq.) under N_2 atmosphere. The resulting mixture was stirred at 0 °C for 10 min and then at room temperature for 12 h. The reaction mixture was washed with water (1 × 40 mL) and sat. aq. NaCl solution (1 × 40 mL), dried over anhydrous sodium sulphate. Solvent was evaporated under reduced pressure. The residue was purified by column chromatography on silica gel using a mixture of pentane and EtOAc as eluent to afford the desired product **3a** as a white solid.

Procedure 11: Electrochemically-mediated PhotoRedox Catalyzed Phosphinate Reductions

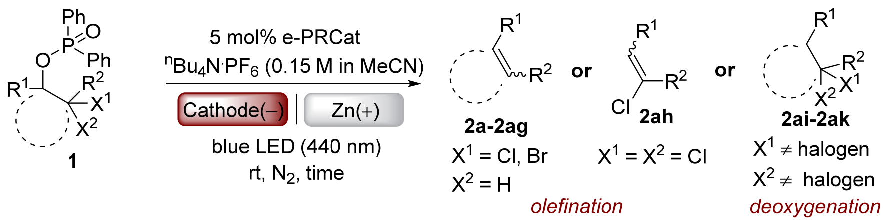

An oven-dried *H*-Cell was equipped with magnetic stirring bars in both chambers. To the cathodic chamber was added substrate **1** (0.2 mmol) and e-PRC catalyst (0.01 mmol), followed by the additions of $^nBu_4N{\cdot}PF_6$ (232.0 mg) and 4 mL MeCN in both chambers (resulting in 0.15 M $^nBu_4N{\cdot}PF_6$ in MeCN as solvent). Both chambers were sealed using rubber septa pierced with wire-connected electrodes then bubbled with N_2 for 10 min. The resulting mixture was stirred at room temperature above a water-cooled cooling block under irradiation of 440 nm LED from beneath the anodic

chamber (Figure S2). A constant potential of −1.6 V was applied across the cell. After the specified reaction time, the mixtures in both chambers were transferred into a flask and each chamber was washed with DCM (3 x 4 mL), the combined organics were evaporated under vacuum. After addition of Et_2O (20 mL) to the residue, $^nBu_4N{\cdot}PF_6$ was precipitated and removed by filtration. The filtrate was concentrated under vacuum and the residue was purified by column chromatography on silica gel using pentane or a mixture of pentane and EtOAc as eluent to afford pure product **2**.

5. Optimization of Reaction Conditions

Table S1. Optimization of light wavelength:

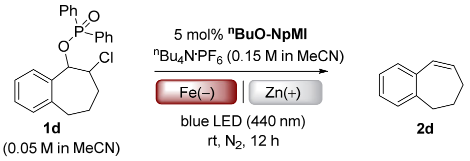

Entry	Wavelength / nm	NMR yield of **2d**[a]
1	400 (“weak”)	6%
2	400 (“strong”)	15%
3	440	75%
4	528 (“weak”)	trace
5	519 (“strong”)	9%
6	740	trace
7	850	trace

[a] Determined by ^1H NMR of the crude product using 1,3,5-trimethoxybenzene as an internal standard. See Table S6 for characterization data of LEDs employed.

Table S2. Optimization of cathodic material:

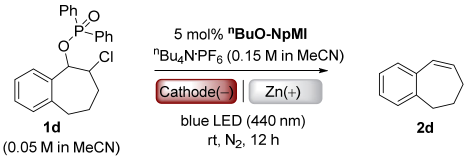

Entry	Cathode	NMR yield of **2d**[a]
1	RVC foam	trace
2	Carbon felt	trace
3	Cu	20%
4	Fe	75%

[a] Determined by ^1H NMR of the crude product using 1,3,5-trimethoxybenzene as an internal standard.

Table S3. Optimization of solvent:

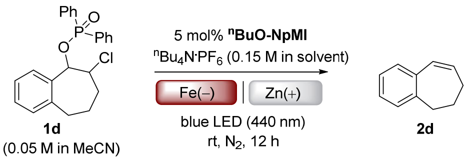

Entry	Solvent	NMR yield of **2d**[a]
1	MeCN	75%
2	DCM	n.d.
3	DMF	n.d.

[a] Determined by ^{1}H NMR of the crude product using 1,3,5-trimethoxybenzene as an internal standard. n.d., not detected.

Table S4. Optimization of catalyst:

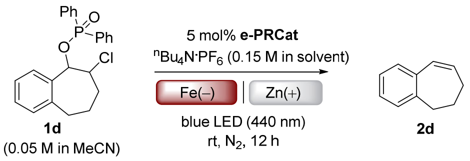

Entry	e-PRCat	NMR yield of **2d**[a]
1	**NpMI**	n.d.
2	**^{n}BuO-NpMI**	75%
3	**5a**	n.d.
4	**5b**	n.d.
5	**5c**	41%
6	**5d**	40%
7	**5e**	55%
8	***N*-Ph NpMI**	n.d.

[a] Determined by ^{1}H NMR of the crude product using 1,3,5-trimethoxybenzene as an internal standard. n.d., not detected.

6. Photoelectrochemical Isomerism Control Experiments

In order to confirm the photoelectrochemical nature of the stilbene isomerism reaction, we subjected commercially available *E*-**2a** to the reaction conditions (Table S5). **DCA** as a catalyst gave no isomerism (entry 1), while **NpMI** gave isomerism marginally favoring *Z*-**2a** (entry 2). Upon extending the reaction time of this reaction, a 1:10 ratio of *E*-**2a** : *Z*-**2a** was achieved. The absence of light gave no isomerism (entry 3), while absence of potential gave marginal isomerism (entry 4). The quartet energy of ^{4}[**NpMI**$^{•-}$]* (as a candidate for the emitting state termed 'ES$_1$') estimated from the excitation emission spectrum of **NpMI**$^{•-}$ and its steady-state emission spectra (56.6 kcal mol^{-1}, see Section S14), is well matched to the triplet energies of *E*-**2a** (51.0 kcal mol^{-1})[8] and *Z*-**2a** (55.5 kcal mol^{-1}).[8]

Interestingly, isomerism occurs in the absence of e-PRCat, marginally favoring *Z*-**2a** (entry 5). However, the ratio did not improve in favor of *Z*-**2a** upon running these conditions for a longer time and rather seemed to erode the selectivity (entry 6), in contrast to the clear enhancing effect of extending reaction time in the presence of **NpMI** (entry 3). The mechanism by which this catalyst-free photoelectrochemical isomerism occurs is currently elusive, but the addition of **DCA** catalyst clearly inhibits this mechanism (entry 1) and the reaction requires applied potential (entries 7-8). **^{n}BuO-NpMI** as an e-PRCat (entry 9) gave a similar result to **NpMI** (entry 3). Finally, a reaction was performed using 365 nm (entry 5). While this gave some isomerism, similar to entry 5, selectivity for the Z-isomer was lower than when 440 nm light was used.

Table S5. Photoelectrochemical isomerism:

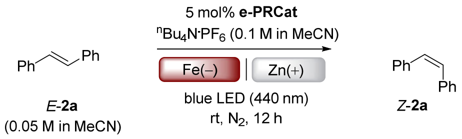

Entry	e-PRCat	λ (nm)	Electrolyte	Potential (V)	Time (h)	*E*-/*Z*- Ratio[a]	
						E-**2a**	*Z*-**2a**
1	**DCA**	440	$^nBu_4N{\cdot}PF_6$	-	10	100	n.d.
2	**NpMI**	440	$^nBu_4N{\cdot}PF_6$	−1.6	10	35	65
3	**NpMI**	440	$^nBu_4N{\cdot}PF_6$	−1.6	36	9	91
3	**NpMI**	-	$^nBu_4N{\cdot}PF_6$	−1.6	10	100	n.d.
4	**NpMI**	440	$^nBu_4N{\cdot}PF_6$	-	10	71	29
5	-	440	$^nBu_4N{\cdot}PF_6$	−1.6	10	33	67
6	-	440	$^nBu_4N{\cdot}PF_6$	−1.6	36	40	60
7	-	440	$^nBu_4N{\cdot}PF_6$	-	10	97	3
8	-	440	-	-	10	96	4
9	**^{n}BuO-NpMI**	440	$^nBu_4N{\cdot}PF_6$	−1.6	36	12	88
10	**^{n}BuO-NpMI**	365	$^nBu_4N{\cdot}PF_6$	-	36	34	66

[a]*E*-/*Z*- Ratio was determined by ^{1}H NMR of the crude product

7. Characterization Data: Catalysts

2-(4-butoxyphenyl)-1*H*-benzo[*de*]isoquinoline-1,3(2*H*)-dione (^{n}BuO-NpMI).

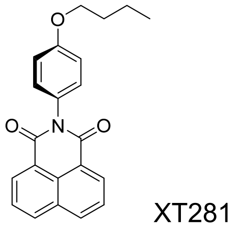

XT281

According to General **Procedure 1**. Yield: 1.54 g, 89%; pale grey solid, m.p. 191 °C; ^{1}H NMR (400 MHz, $CDCl_3$) δ 8.70 (dd, *J* = 0.8, 7.2 Hz, 2H), 8.31 (dd, *J* = 0.8, 8.4 Hz, 2H), 7.87-7.80 (m, 2H), 7.30-7.24 (m, 2H), 7.12-7.08 (m, 2H), 4.08 (t, *J* = 6.4 Hz, 2H), 1.90-1.82 (m, 2H), 1.63-1.52 (m, 2H), 1.05 (t, *J* = 7.2 Hz, 3H) ppm; ^{13}C NMR (100 MHz, $CDCl_3$) δ 164.6, 159.2, 134.2, 131.8, 131.6, 129.5, 128.5, 127.7, 127.0, 122.9, 115.3, 67.9, 31.3, 19.3, 13.9 ppm; HRMS (ESI) (*m/z*) $[M+H]^+$ $C_{22}H_{20}NO_3$ calcd. for 346.1438, found 346.1443.

2-(2,6-diisopropylphenyl)-6-methoxy-1*H*-benzo[*de*]isoquinoline-1,3(2*H*)-dione (5a).

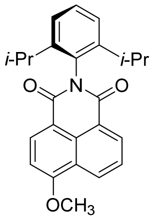

XT310

According to **Procedure 2**. Yield: 348 mg, 90%; pale yellow solid, m.p. 253-255 °C; ^{1}H NMR (400 MHz, $CDCl_3$) δ 8.70-8.61 (m, 3H), 7.79-7.73 (m, 1H), 7.49-7.43 (m, 1H), 7.33 (d, *J* = 7.6 Hz, 2H), 7.10 (d, *J* = 8.0 Hz, 1H), 4.16 (s, 3H), 2.76 (hept, *J* = 6.4 Hz, 2H), 1.16 (dd, *J* = 6.8, 1.2 Hz, 12H) ppm; ^{13}C NMR (100 MHz, $CDCl_3$) δ 164.6, 164.0, 161.1, 145.8, 134.0, 132.1, 131.2, 130.1, 129.4, 129.0, 126.1, 124.0, 123.8, 122.6, 115.2, 105.3, 56.4, 29.1, 24.0 ppm; HRMS (ESI) (*m/z*) $[M+H]^+$ $C_{25}H_{26}NO_3$ calcd. for 388.1907, found 388.1914.

2-(2,6-diisopropylphenyl)-6-(4-methoxyphenyl)-1*H*-benzo[*de*]isoquinoline-1,3(2*H*)-dione (5b).

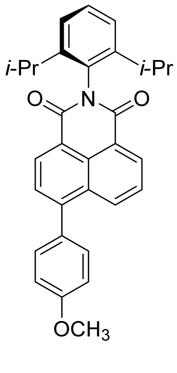

XT311

According to **Procedure 3**. Yield: 347 g, 75%; yellow solid, m.p. 216-217 °C; ^{1}H NMR (400 MHz, $CDCl_3$) δ 8.48 (d, *J* = 7.6 Hz, 2H), 8.17 (d, *J* = 8.4 Hz, 1H), 7.57-7.48 (m, 2H), 7.30-7.22 (m, 3H), 7.12 (d, *J* = 7.6 Hz, 2H), 6.89 (d, *J* = 8.4 Hz, 2H), 3.71 (s, 3H), 2.56 (hept, *J* = 6.4 Hz, 2H), 0.95 (d, *J* = 6.4, 12H) ppm; ^{13}C NMR (100 MHz, $CDCl_3$) δ 164.4, 164.2, 160.0, 147.1, 145.7, 133.1, 131.7, 131.4, 131.2 (one carbon was overlapped), 130.9, 130.5, 129.50, 129.45, 127.9, 126.8, 124.0, 123.0, 121.4, 114.3, 55.5, 29.2, 24.0 ppm; HRMS (ESI) (*m/z*) $[M+H]^+$ $C_{31}H_{30}NO_3$ calcd. for 464.2220, found 464.2224.

2-(3,4,5-trimethoxyphenyl)-1*H*-benzo[*de*]isoquinoline-1,3(2*H*)-dione (5c)

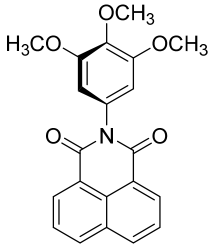

XT283

According to **Procedure 1**. Yield: 1.55 g, 85%; grey solid, m.p. > 300°C; ^{1}H NMR (400 MHz, $CDCl_3$) δ 8.66 (dd, *J* = 7.2, 0.8 Hz, 2H), 8.28 (dd, *J* = 8.0, 0.8 Hz, 2H), 7.80 (dd, *J* = 8.0, 7.6 Hz, 2H), 6.55 (s, 2H), 3.92 (s, 3H), 3.86 (s, 6H) ppm; ^{13}C NMR (100 MHz, $CDCl_3$) δ 164.5, 153.9, 138.1, 134.4, 131.8, 131.7, 131.1, 128.5, 127.1, 122.8, 105.9, 60.9, 56.1 ppm; HRMS (ESI) (*m/z*) $[M+H]^+$ $C_{21}H_{18}NO_5$ calcd. for 364.1179, found 364.1183. Data are consistent with the literature.[9]

2-(2,6-dimethylphenyl)-1*H*-benzo[*de*]isoquinoline-1,3(2*H*)-dione (5d)

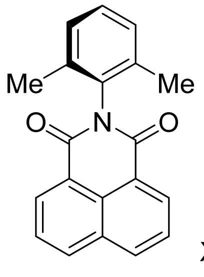

XT481

According to **Procedure 1**. Yield: 1.16 g, 77%; pale brown solid, m.p. 227-229 °C; ^{1}H NMR (400 MHz, $CDCl_3$) δ 8.68 (dd, *J* = 7.2, 0.8 Hz, 2H), 8.30 (dd, *J* = 8.4, 0.8 Hz, 2H) ppm, 7.81 (dd, *J* = 8.0, 7.2 Hz, 2H), 7.32-7.27 (m, 1H), 7.25-7.21 (m, 2H), 2.16 (s, 6H) ppm; ^{13}C NMR (100 MHz, $CDCl_3$) δ 163.5, 135.6, 134.4, 134.0, 131.9, 131.7, 128.9, 128.8, 128.6, 127.1, 122.8, 17.9 ppm; HRMS (ESI) (*m/z*) $[M+H]^+$ $C_{20}H_{16}NO_2$ calcd. for 302.1176, found 302.1182.

2-(2,6-diethylphenyl)-1*H*-benzo[*de*]isoquinoline-1,3(2*H*)-dione (5e)

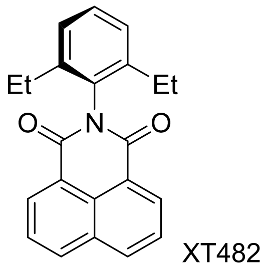

XT482

According to **Procedure 1**. Yield: 1.17 g, 71%; pale brown solid, m.p. 225-226 °C; ^{1}H NMR (400 MHz, $CDCl_3$) δ 8.67 (dd, *J* = 7.2, 0.8 Hz, 2H), 8.30 (dd, *J* = 8.4, 0.8 Hz, 2H), 7.82 (dd, *J* = 8.0, 7.2 Hz, 2H), 7.42 (t, *J* = 7.6 Hz, 1H), 7.29 (d, *J* = 7.6 Hz, 2H), 2.48 (q, *J* = 7.6 Hz, 4H), 1.15 (t, *J* = 7.6 Hz, 6H) ppm; ^{13}C NMR (100 MHz, $CDCl_3$) δ 164.1, 140.9, 134.3, 132.8, 131.9, 131.7, 129.2, 128.9, 127.1, 126.5, 122.8, 24.3, 14.0 ppm; HRMS (ESI) (*m/z*) $[M+H]^+$ $C_{22}H_{20}NO_2$ calcd. for 330.1489, found 330.1491. Data are consistent with the literature.[10]

2-phenyl-1*H*-benzo[*de*]isoquinoline-1,3(2*H*)-dione (*N*-Ph NpMI)

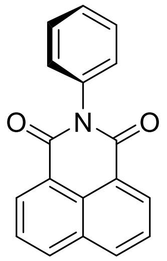

According to **Procedure 1**. Yield: 821 mg, 60%; pale brown solid, m.p. 199 ºC; ^{1}H NMR (400 MHz, $CDCl_3$) δ 8.65 (dd, J = 0.8, 7.2 Hz, 2H), 8.31 (dd, J = 0.8, 8.4 Hz, 2H), 7.82-7.75 (m, 2H), 7.59-7.53 (m, 2H), 7.52-7.46 (m, 1H), 7.36-7.30 (m, 2H) ppm; ^{13}C NMR (100 MHz, $CDCl_3$) δ 164.5, 135.6, 134.4, 131.9, 131.7, 129.5, 128.82, 128.76, 128.66, 127.2, 123.0 ppm. ^{1}H and ^{13}C data are consistent with the literature: H. J. Kim, J. Kim, S. H. Cho, S. Chang, *J. Am. Chem. Soc.* **2011**, *133*, 16382–16385.

8. Characterization Data: Phosphinate Substrates

2-chloro-1,2-diphenylethyl diphenylphosphinate (1a)

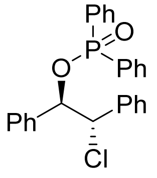

dr > 30:1 XT24

According to **Procedure 5**. Yield: 926.0 mg, 71%; dr > 30:1; white solid, m.p. 164-165 °C; ^{1}H NMR (400 MHz, $CDCl_3$) δ 7.50-7.39 (m, 5H), 7.35-7.23 (m, 8H), 7.22-7.11 (m, 7H), 5.70 (dd, *J* = 9.2, 6.8 Hz,

1H), 5.25 (d, J = 6.8 Hz, 1H) ppm; ^{13}C NMR (100 MHz, $CDCl_3$) δ 137.4, 136.3 (d, J_{C-P} = 2.1 Hz), 132.1 (d, J_{C-P} = 2.7 Hz), 132.0 (d, J_{C-P} = 2.8 Hz), 131.7 (d, J_{C-P} = 10.4 Hz), 131.44 (d, J_{C-P} = 10.5 Hz), 131.40 (d, J_{C-P} = 137.6 Hz), 131.0 (d, J_{C-P} = 133.0 Hz), 128.7, 128.6, 128.4, 128.3 (d, J_{C-P} = 13.4 Hz), 128.13, 128.09 (d, J_{C-P} = 13.2 Hz), 127.8, 80.1 (d, J_{C-P} = 5.7 Hz), 65.6 (d, J_{C-P} = 6.6 Hz) ppm; ^{31}P NMR (162 MHz, $CDCl_3$) δ 33.6 ppm; HRMS (ESI) (*m/z*) $[M+H]^+$ $C_{26}H_{23}{}^{35}ClO_2P$ calcd. for 433.1119, found 433.1122. Data are consistent with the literature.[11]

2-chloro-2-phenyl-1-(*p*-tolyl)ethyl diphenylphosphinate (1b)

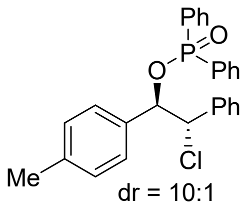

SY012

According to **Procedure 5**. Yield: 1.10 g, 82%; two diastereomers, dr = 10:1; white solid, m.p. 127-128 °C; ^{1}H NMR (400 MHz, $CDCl_3$) δ 7.92-7.76 (m, 2H), 7.62-7.42 (m, 5H), 7.39-7.33 (m, 1H), 7.27-7.17 (m, 3H), 7.14-7.03 (m, 3H), 7.02-6.93 (m, 5H), 5.76-5.72 (m, 0.1H, the minor isomer), 5.70-5.63 (m, 0.9H, the major isomer), 5.35-5.28 (m, 0.9H, the major isomer), 5.28-5.23 (m, 0.1H, the minor isomer), 2.28 (s, 0.27H, the minor isomer), 2.24 (s, 2.71H, the major isomer) ppm; ^{13}C NMR (100 MHz, $CDCl_3$) (not possible to assign every peak) δ 138.31, 136.22, 136.20, 133.60, 132.24, 132.22, 131.99, 131.96, 131.85, 131.79, 131.69, 128.77, 128.52, 128.48, 128.45, 128.39, 128.20, 128.17, 128.07, 127.94, 127.80, 80.74, 80.68, 77.46, 77.14, 76.82, 65.78, 65.72, 21.17 ppm; ^{31}P NMR (162 MHz, $CDCl_3$) δ 33.6 (0.1P, the minor isomer), 33.3 (0.9P, the major isomer) ppm; HRMS (ESI) (*m/z*) $[M+H]^+$ $C_{27}H_{25}{}^{35}ClO_2P$ calcd. for 447.1275, found 447.1281.

2,2-dichloro-2-phenyl-1-(4-(trifluoromethyl)phenyl)ethyl diphenylphosphinate (1c)

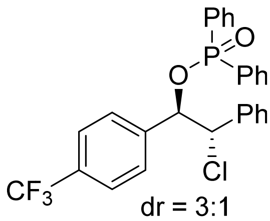

SY43-2

According to **Procedure 5**. Yield: 1.06 g, 65%; two diastereomers, dr = 3:1, white solid, m.p. 108-110 °C; ^{1}H NMR (400 MHz, $CDCl_3$) δ 7.88-7.57 (m, 4H), 7.55-7.30 (m, 7H), 7.29-6.98 (m, 8H), 5.83 (dd, J = 9.2, 6.8 Hz, 0.25H, the minor isomer), 5.71 (dd, J = 9.2, 6.8 Hz, 0.75H, the major isomer), 5.46 (d, J = 6.8 Hz, 0.75H, the major isomer), 5.33 (d, J = 6.8 Hz, 0.25H, the minor isomer) ppm; ^{13}C NMR (100 MHz, $CDCl_3$) (not possible to assign every peak) δ 141.39, 140.29, 136.05, 135.33, 135.30, 132.40, 132.38, 132.26, 132.23, 132.20, 132.17, 132.10, 132.06, 131.92, 131.82, 131.68, 131.57, 131.46, 131.34, 131.26, 131.16, 130.71, 130.38, 130.01, 129.19, 129.01, 128.98, 128.80, 128.61, 128.47, 128.39, 128.32, 128.26, 128.21, 128.19, 128.08, 128.03, 127.91, 127.83, 125.33, 125.29, 125.19, 124.93, 124.89, 124.86, 124.82, 122.49, 80.17, 80.11, 79.74, 79.69, 64.45, 64.40 ppm; ^{31}P

NMR (162 MHz, $CDCl_3$) δ 34.4 (0.33P, the minor isomer), 34.0 (1P, the major isomer) ppm; ^{19}F NMR (377 MHz, $CDCl_3$) δ -63.1 (0.33F, the minor isomer), -63.2 (1F, the minor isomer) ppm; HRMS (ESI) (*m/z*) $[M+H]^+$ $C_{27}H_{22}{}^{35}ClF_3O_2P$ calcd. for 501.0993, found 501.0998.

6-chloro-6,7,8,9-tetrahydro-5*H*-benzo[7]annulen-5-yl diphenylphosphinate (1d)

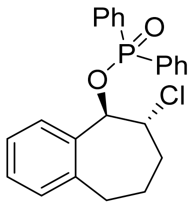

dr = 20:1 XT290

According to **Procedure 5**. Yield: 1.01 g, 85%; two diastereomers, dr = 20:1; white solid, m.p. 118-119 °C; ^1H NMR (400 MHz, $CDCl_3$) δ 7.75-7.63 (m, 2H), 7.52-7.38 (m, 2H), 7.37-7.30 (m, 1H), 7.30-7.23 (m, 2H), 7.22-7.15 (m, 1H), 7.13-6.98 (m, 3H), 6.96-6.86 (m, 2H), 6.82-6.75 (m, 1H), 5.60 (d, *J* = 8.4 Hz, 1H), 4.53-4.05 (m, 1H), 2.94-2.64 (m, 1H), 2.45-2.33 (m, 1H), 2.32-2.15 (m, 1H), 2.13-2.00 (m, 1H), 1.75-1.54 (m, 1H), 1.49-1.29 (m, 1H) ppm; ^{13}C NMR (100 MHz, $CDCl_3$) (not possible to assign every peak) δ 135.70, 132.52, 132.33, 132.30, 132.07, 131.98, 131.88, 131.72, 131.70, 131.61, 131.60, 131.44, 131.34, 131.14, 130.63, 129.86, 129.75, 128.69, 128.62, 128.49, 128.36, 128.22, 126.23, 81.18, 79.11, 77.47, 77.15, 76.83, 63.19, 36.55, 34.86 ppm; ^{31}P NMR (162 MHz, $CDCl_3$) δ 33.4 (1P, the major isomer), 31.9 (0.05P, the minor isomer) ppm; HRMS (ESI) (*m/z*) $[M+H]^+$ $C_{23}H_{23}{}^{35}ClO_2P$ calcd. for 397.1119, found 397.1123.

2-chloro-1,2,3,4-tetrahydronaphthalen-1-yl diphenylphosphinate (1e)

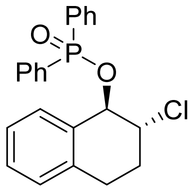

dr > 30:1 XT233

According to **Procedure 5**. Yield: 982.0 mg, 81%; dr > 30/1; white solid, m.p. 149-151 °C; ^1H NMR (400 MHz, $CDCl_3$) δ 7.80-7.66 (m, 2H), 7.64-7.53 (m, 2H), 7.36-7.22 (m, 4H), 7.22-7.12 (m, 3H), 7.01-6.93 (m, 1H), 6.92-6.82 (m, 2H), 5.54 (d, *J* = 6.0 Hz, 1H), 4.31-4.18 (m, 1H), 3.03-2.87 (m, 1H), 2.69-2.54 (m, 1H), 2.26-2.12 (m, 1H), 2.04-1.93 (m, 1H) ppm; ^{13}C NMR (100 MHz, $CDCl_3$) δ 135.2, 132.9 (d, J_{C-P} = 3.7 Hz), 132.03 (d, J_{C-P} = 136.2 Hz), 131.93 (d, J_{C-P} = 137.6 Hz), 132.24 (d, J_{C-P} = 13.0 Hz), 132.22 (d, J_{C-P} = 12.9 Hz), 131.89 (d, J_{C-P} = 10.6 Hz), 131.77 (d, J_{C-P} = 10.3 Hz), 129.1, 128.55, 128.52, 128.41, 128.38, 126.3, 73.6 (d, J_{C-P} = 5.7 Hz), 59.3 (d, J_{C-P} = 2.7 Hz), 27.9, 26.1 ppm; ^{31}P NMR (162 MHz, $CDCl_3$) δ 33.4 ppm; HRMS (ESI) (*m/z*) $[M+H]^+$ $C_{22}H_{20}{}^{35}ClNaO_2P$ calcd. for 405.0782, found 405.0779.

2-chloro-2,3-dihydro-1*H*-inden-1-yl diphenylphosphinate (1f)

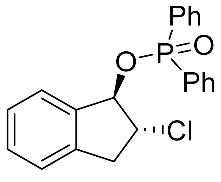

dr > 30:1 XT157

According to **Procedure 5**. Yield: 883.0 mg, 80%; dr > 30:1; white solid, m.p. 102-105 °C; ^{1}H NMR (400 MHz, $CDCl_3$) δ 8.02-7.91 (m, 2H), 7.90-7.81 (m, 2H), 7.57-7.40 (m, 6H), 7.37 (d, *J* = 7.6 Hz, 1H), 7.31-7.26 (m, 1H), 7.24-7.16 (m, 2H), 5.80 (dd, *J* = 8.8, 5.2 Hz, 1H), 4.64-4.58 (m, 1H), 3.32 (d, *J* = 5.2 Hz, 2H) ppm; ^{13}C NMR (100 MHz, $CDCl_3$) δ 139.4, 138.6 (d, J_{C-P} = 3.7 Hz), 132.4 (d, J_{C-P} = 2.8 Hz), 132.3 (d, J_{C-P} = 2.8 Hz), 132.1 (d, J_{C-P} = 10.4 Hz), 131.8 (d, J_{C-P} = 10.1 Hz), 131.7 (d, J_{C-P} = 137.8 Hz), 131.6 (d, J_{C-P} = 134.7 Hz), 129.4, 128.6 (d, J_{C-P} = 3.4 Hz), 128.5 (d, J_{C-P} = 3.4 Hz), 127.5, 125.9, 124.8, 77.8 (d, J_{C-P} = 5.7 Hz), 61.7 (d, J_{C-P} = 4.6 Hz), 40.2 ppm; ^{31}P NMR (162 MHz, $CDCl_3$) δ 33.5 ppm; HRMS (ESI) (*m/z*) $[M+H]^+$ $C_{21}H_{19}{}^{35}ClO_2P$ calcd. for 369.0806, found 369.0811.

2-chloro-1-phenylcyclohexyl diphenylphosphinate (1g)

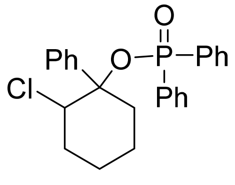

dr > 30:1 XT119-2

According to **Procedure 7**. Yield: 1.88 g, 87%; dr > 30:1; white solid, m.p. 180 °C; ^{1}H NMR (400 MHz, $CDCl_3$) δ 7.83-7.75 (m, 2H), 7.69-7.61 (m, 2H), 7.47-7.34 (m, 6H), 7.33-7.26 (m, 2H), 7.17-7.08 (m, 3H), 4.85-4.72 (m, 1H), 2.88-2.76 (m, 1H), 2.52-2.43 (m, 1H), 2.08-1.98 (m, 1H), 1.91-1.73 (m, 2H), 1.63-1.51 (m, 1H), 1.51-1.33 (m, 2H) ppm; ^{13}C NMR (100 MHz, $CDCl_3$) δ 139.4, 134.2 (d, J_{C-P} = 138.7 Hz), 134.0 (d, J_{C-P} = 137.8 Hz), 131.52, 131.51 (d, J_{C-P} = 2.8 Hz), 131.47 (d, J_{C-P} = 10.2 Hz), 131.37 (d, J_{C-P} = 2.8 Hz), 131.34 (d, J_{C-P} = 10.3 Hz), 128.25 (d, J_{C-P} = 10.0 Hz), 128.17, 128.11 (d, J_{C-P} = 10.2 Hz), 128.02, 127.28, 86.9 (d, J_{C-P} = 8.6 Hz), 66.9 (d, J_{C-P} = 6.8 Hz), 34.0, 32.0, 22.2, 22.0 ppm; ^{31}P NMR (162 MHz, $CDCl_3$) δ 28.0 ppm; HRMS (ESI) (*m/z*) $[M+H]^+$ $C_{24}H_{24}{}^{35}ClNaO_2P$ calcd. for 433.1095, found 433.1096.

(1*S*,2*R*,3*R*,4*R*)-3-chloro-2-phenylbicyclo[2.2.1]heptan-2-yl diphenylphosphinate (1h)

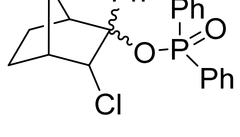

dr > 30:1 XT154

According to **Procedure 7**. Yield: 1.58 g, 71%; dr > 30:1; colorless oil; ^{1}H NMR (400 MHz, $CDCl_3$) δ 7.80-7.72 (m, 2H), 7.34-7.24 (m, 5H), 7.20-7.13 (m, 2H), 7.08-7.01 (m, 1H), 6.96-6.88 (m, 2H), 6.87-

6.78 (m, 3H), 4.18-4.08 (m, 1H), 3.68 (d, J = 3.2 Hz, 1H), 2.44-2.32 (m, 2H), 1.56-1.41(m, 2H), 1.35-1.27 (m, 1H), 1.25-1.17 (m, 1H), 1.06-0.96 (m, 1H) ppm; ^{13}C NMR (100 MHz, $CDCl_3$) δ 136.3 (d, J_{C-P} = 2.0 Hz), 134.6 (d, J_{C-P} = 179.9 Hz), 133.2 (d, J_{C-P} = 167.7 Hz), 131.4 (d, J_{C-P} = 2.8 Hz), 131.3 (d, J_{C-P} = 10.5 Hz), 131.0 (d, J_{C-P} = 10.0 Hz), 130.5 (d, J_{C-P} = 2.8 Hz), 130.0, 128.5, 128.3 (d, J_{C-P} = 13.3 Hz), 127.7, 127.4 (d, J_{C-P} = 13.3 Hz), 91.7 (d, J_{C-P} = 7.7 Hz), 71.6 (d, J_{C-P} = 7.2 Hz), 47.0 (d, J_{C-P} = 1.8 Hz), 34.8, 28.6, 22.8 ppm; ^{31}P NMR (162 MHz, $CDCl_3$) δ 26.4 ppm; HRMS (ESI) (*m/z*) $[M+H]^+$ $C_{25}H_{24}{}^{35}ClNaO_2P$ calcd. for 445.1095, found 445.1091.

1-([1,1'-biphenyl]-4-yl)-2-chloropropyl diphenylphosphinate (1j)

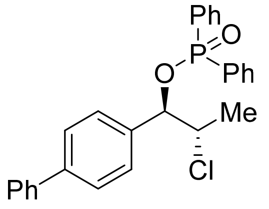

dr = 20:1 XT295

According to **Procedure 5**. Yield: 963.0 mg, 72%; two diastereomers, dr = 20:1 white solid, m.p. 116-117 °C; ^{1}H NMR (400 MHz, $CDCl_3$) δ 7.70 (dd, J = 7.6, 12.0 Hz, 2H), 7.42 (dd, J = 8.0, 12.4 Hz, 2H), 7.38-7.32 (m, 3H), 7.32-7.21 (m, 6H), 7.21-7.12 (m, 4H), 7.09-7.02 (m, 2H), 5.25 (dd, J = 6.4, 8.8 Hz, 1H), 4.32-4.21 (m, 1H), 1.38 (d, J = 6.4 Hz, 0.14H, the minor isomer), 1.24 (d, J = 6.4 Hz, 2.86H, the major isomer) ppm; ^{13}C NMR (100 MHz, $CDCl_3$) δ 141.5, 140.5, 135.6 (d, J_{C-P} = 2.2 Hz), 132.3 (d, J_{C-P} = 2.6 Hz), 132.02 (d, J_{C-P} = 2.6 Hz), 131.99 (d, J_{C-P} = 14.2 Hz), 131.7 (d, J_{C-P} = 10.3 Hz), 130.7 (d, J_{C-P} = 48.5 Hz), 128.8, 128.5 (d, J_{C-P} = 13.3 Hz), 128.2 (d, J_{C-P} = 13.2 Hz), 128.1, 127.54, 127.48 (d, J_{C-P} = 145.7 Hz), 127.1, 126.9, 80.4 (d, J_{C-P} = 5.6 Hz), 59.6 (d, J_{C-P} = 5.5 Hz), 20.7 ppm; ^{31}P NMR (162 MHz, $CDCl_3$) δ 33.2 ppm; HRMS (ESI) (*m/z*) $[M+H]^+$ $C_{27}H_{25}{}^{35}ClO_2P$ calcd. for 447.1275, found 447.1269.

(*E*)-4-(prop-1-en-1-yl)phenyl benzoate (1k)

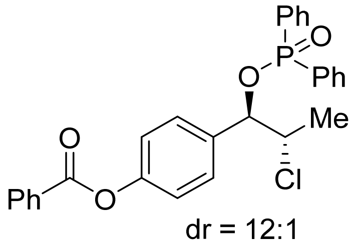

dr = 12:1 XT256

According to **Procedure 5**. Yield: 1.13 g, 77%; two diastereomers, dr = 12:1; white solid, m.p. 139-140 °C; ^{1}H NMR (400 MHz, $CDCl_3$) δ 8.17-8.07 (m, 2H), 7.85-7.75 (m, 2H), 7.60-7.50 (m, 3H), 7.50-7.32 (m, 6H), 7.30-7.20 (m, 4H), 7.06 (d, J = 8.8 Hz, 2H), 5.36 (d, J = 9.2, 5.6 Hz, 1H), 4.42-4.32 (m, 1H), 1.48 (d, J = 6.8 Hz, 0.23H, the minor isomer), 1.36 (d, J = 6.8 Hz, 2.77H, the major isomer) ppm; ^{13}C NMR (100 MHz, $CDCl_3$) δ 164.9, 151.1, 134.2 (d, J_{C-P} = 2.8 Hz), 133.7, 132.4 (d, J_{C-P} = 2.8 Hz), 132.23 (d, J_{C-P} = 2.7 Hz), 131.89 (d, J_{C-P} = 10.4 Hz), 131.61 (d, J_{C-P} = 10.3 Hz), 131.44 (d, J_{C-P} = 139.3 Hz), 130.77 (d, J_{C-P} = 133.1 Hz), 130.2, 129.4, 128.8, 128.65, 128.56 (d, J_{C-P} = 13.5 Hz), 128.33 (d, J_{C-}

P = 13.1 Hz), 121.5, 79.9 (d, J_{C-P} = 5.7 Hz), 59.4 (d, J_{C-P} = 5.2 Hz), 20.5 ppm; ^{31}P NMR (162 MHz, $CDCl_3$) δ 33.5 ppm; HRMS (ESI) (*m/z*) $[M+H]^+$ $C_{28}H_{25}ClO_4P$ calcd. for 491.1173, found 491.1171.

2-chloro-1-(4-methoxyphenyl)propyl diphenylphosphinate (1l)

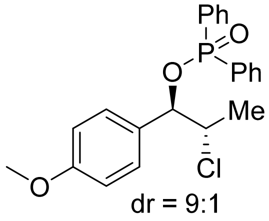

SY22

According to **Procedure 5**. Yield: 1.09 g, 86%; two diastereomers, dr = 9:1; colorless oil; ^{1}H NMR (400 MHz, $CDCl_3$) δ 7.88-7.81 (m, 2H), 7.62-7.56 (m, 2H), 7.54-7.51 (m, 1H), 7.49-7.43 (m, 2H), 7.42-7.37 (m, 1H), 7.33-7.22 (m, 3H), 7.22-7.12 (m, 2H), 6.80-6.75 (m, 1.8H, the major isomer), 6.73-6.70 (m, 0.2H, the minor isomer), 5.57 (dd, *J* = 9.6, 4.0 Hz, 0.1H, the minor isomer), 5.32 (dd, *J* = 9.2, 6.4 Hz, 0.9H, the major isomer), 4.45-4.35 (m, 1H), 3.86 (s, 0.3H, the minor isomer), 3.77 (s, 2.7H, the major isomer), 1.51 (d, *J* = 6.8 Hz, 0.27H), 1.35 (d, *J* = 6.4 Hz, 2.7H, the major isomer) ppm; ^{13}C NMR (100 MHz, $CDCl_3$) peaks of the major isomer δ 156.8, 132.2 (d, J_{C-P} = 2.8 Hz), 131.94 (d, J_{C-P} = 2.8 Hz), 131.89 (d, J_{C-P} = 10.3 Hz), 131.70 (d, J_{C-P} = 138.8 Hz), 131.65 (d, J_{C-P} = 10.3 Hz), 131.20 (d, J_{C-P} = 135.6 Hz), 128.9, 128.5 (d, J_{C-P} = 13.3 Hz), 128.1 (d, J_{C-P} = 13.3 Hz), 113.6, 80.5 (d, J_{C-P} = 5.8 Hz), 59.7 (d, J_{C-P} = 5.7 Hz), 55.3, 20.6 ppm; ^{31}P NMR (162 MHz, $CDCl_3$) δ 32.9 (0.1P, the minor isomer), 32.8 (1.0P, the major isomer) ppm; HRMS (ESI) (*m/z*) $[M+H]^+$ $C_{22}H_{22}{}^{35}ClNaO_3P$ calcd. for 423.0887, found 423.0887.

2-chloro-1-(4-methoxyphenyl)ethyl diphenylphosphinate (1m)

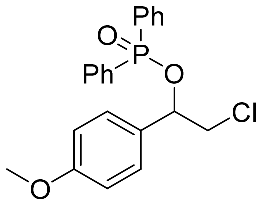

XT366

According to **Procedure 5**. Yield: 961.0 mg, 83%; colorless oil; ^{1}H NMR (300 MHz, $CDCl_3$) δ 7.94-7.86 (m, 2H), 7.75-7.67 (m, 2H), 7.62-7.56 (m, 1H), 7.54-7.47 (m, 3H), 7.42-7.35 (m, 2H), 7.30-7.24 (m, 2H), 6.91-6.84 (m, 2H), 5.51 (dt, *J* = 9.3, 3.3 Hz, 1H), 4.03 (dd, *J* = 8.4, 4.5 Hz, 1H), 3.85 (dd, *J* = 8.4, 4.5 Hz, 1H), 3.84 (s, 3H) ppm; ^{13}C NMR (100 MHz, $CDCl_3$) δ 159.9, 132.3 (d, J_{C-P} = 2.9 Hz), 132.14 (d, J_{C-P} = 2.8 Hz), 132.06 (d, J_{C-P} = 150.2 Hz), 131.9 (d, J_{C-P} = 10.3 Hz), 131.62 (d, J_{C-P} = 10.3 Hz), 130.7 (d, J_{C-P} = 146.8 Hz), 129.5 (d, J_{C-P} = 3.6 Hz), 128.5 (d, J_{C-P} = 13.3 Hz), 128.31 (d, J_{C-P} = 13.1 Hz), 128.27, 113.9, 76.6 (d, J_{C-P} = 5.6 Hz), 55.3, 47.8 (d, J_{C-P} = 5.0 Hz) ppm; ^{31}P NMR (162 MHz, $CDCl_3$) δ 33.1 ppm; HRMS (ESI) (*m/z*) $[M+Na]^+$ $C_{21}H_{20}{}^{35}ClO_3PNa$ calcd. For 409.0736, found 409.0727.

2-chloro-1-(3-(trifluoromethyl)phenyl)propyl diphenylphosphinate (1n)

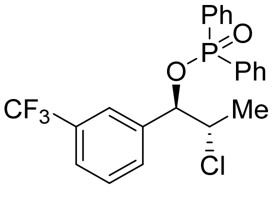

dr = 20:1 XT292

According to **Procedure 5**. Yield: 972.0 mg, 74%; two diastereomers, dr = 20:1; white solid, m.p. 72-74 °C; ^{1}H NMR (300 MHz, $CDCl_3$) δ 7.92-7.82 (m, 2H), 7.61-7.44 (m, 8H), 7.42-7.32 (m, 2H), 7.29-7.20 (m, 2H), 5.55-5.46 (m, 1H), 4.44-4.33 (m, 1H), 1.54 (d, *J* = 6.9 Hz, 0.14H), 1.42 (d, *J* = 6.9 Hz, 2.86H) ppm; ^{13}C NMR (100 MHz, $CDCl_3$) (not possible to assign every peak) δ 137.71, 137.69, 132.54, 132.51, 132.28, 132.26, 131.75, 131.65, 131.61, 131.51, 131.03, 128.33, 128.20, 127.87, 125.43, 125.39, 125.16, 124.35, 124.31, 124.27, 122.45, 119.75, 79.21, 79.16, 77.38, 77.07, 76.75, 59.18, 59.12, 20.56 ppm; ^{31}P NMR (121 MHz, $CDCl_3$) δ 33.8 (1P, the major isomer), 32.2 (0.05P, the minor isomer) ppm; ^{19}F NMR (282 MHz, $CDCl_3$) δ -63.2 ppm; HRMS (ESI) (*m/z*) [M+H]$^+$ $C_{22}H_{20}{}^{35}ClF_3O_2P$ calcd. For 439.0836, found 439.0841.

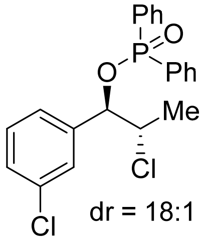

dr = 18:1 SY19

According to **Procedure 5**. Yield: 957.0 mg, 79%; two diastereomers, dr = 18:1; colorless oil; ^{1}H NMR (400 MHz, $CDCl_3$) δ 8.02-7.92 (m, 2H), 7.73-7.66 (m, 2H), 7.65-7.60 (m, 1H), 7.59-7.53 (m, 2H), 7.51-7.45 (m, 1H), 7.40-7.33 (m, 3H), 7.32-7.27 (m, 1H), 7.27-7.22 (m, 2H), 4.51-4.42 (m, 1H), 1.61 (d, *J* = 6.4 Hz, 0.16H, the minor isomer), 1.49 (d, *J* = 6.4 Hz, 2.86H, the major isomer) ppm; ^{13}C NMR (100 MHz, $CDCl_3$) peaks of the major isomer δ 138.6 (d, $J_{C\text{-}P}$ = 2.5 Hz), 134.1, 132.5 (d, $J_{C\text{-}P}$ = 2.7 Hz), 132.3 (d, $J_{C\text{-}P}$ = 2.8 Hz), 131.8 (d, $J_{C\text{-}P}$ = 10.5 Hz), 131.6 (d, $J_{C\text{-}P}$ = 10.4 Hz), 131.2 (d, $J_{C\text{-}P}$ = 139.0 Hz), 130.7 (d, $J_{C\text{-}P}$ = 133.1 Hz), 129.5, 128.8, 128.6 (d, $J_{C\text{-}P}$ = 13.3 Hz), 128.3 (d, $J_{C\text{-}P}$ = 13.2 Hz), 127.6, 125.8, 79.4 (d, $J_{C\text{-}P}$ = 5.6 Hz), 59.2 (d, $J_{C\text{-}P}$ = 5.6 Hz), 20.6 ppm; ^{31}P NMR (162 MHz, $CDCl_3$) δ 33.6 ppm; HRMS (ESI) (*m/z*) [M+H]$^+$ $C_{21}H_{20}{}^{35}Cl_2O_2P$ calcd. For 405.0572, found 405.0572.

2-chloro-1-(4-chlorophenyl)propyl diphenylphosphinate (1p)

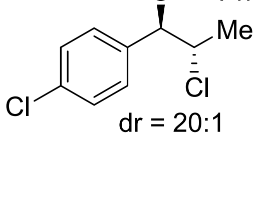

dr = 20:1 SY24

According to **Procedure 5**. Yield: 994.0 mg, 82%; two diastereomers, dr = 20:1; white solid, m.p. 96-98 °C; ^{1}H NMR (400 MHz, $CDCl_3$) δ 7.94-7.85 (m, 2H), 7.67-7.64 (m, 1H), 7.64-7.61 (m, 1H), 7.61-7.55 (m, 1H), 7.54-7.42 (m, 3H), 7.35-7.29 (m, 2H), 7.27-7.25 (m, 4H), 5.42 (dd, J = 9.2, 5.6 Hz, 1H), 4.46-4.38 (m, 1H), 1.55 (d, J = 6.8 Hz, 0.14H, the minor isomer), 1.42 (d, J = 6.8 Hz, 2.8H, the major isomer) ppm; ^{13}C NMR (100 MHz, $CDCl_3$) peaks of the major isomer δ 135.1 (d, J_{C-P} = 2.6 Hz), 134.6, 132.4 (d, J_{C-P} = 2.8 Hz), 132.2 (d, J_{C-P} = 2.7 Hz), 131.77 (d, J_{C-P} = 10.4 Hz), 131.59 (d, J_{C-P} = 10.3 Hz), 131.25 (d, J_{C-P} = 138.7 Hz), 130.83 (d, J_{C-P} = 133.5 Hz), 128.9, 128.6 (d, J_{C-P} = 13.4 Hz), 128.4, 128.3 (d, J_{C-P} = 14.0 Hz), 79.6 (d, J_{C-P} = 5.6 Hz), 59.3 (d, J_{C-P} = 5.5 Hz), 20.4 ppm; ^{31}P NMR (162 MHz, $CDCl_3$) δ 33.5 ppm; HRMS (ESI) (*m/z*) [M+H]$^+$ $C_{21}H_{20}{}^{35}Cl_2O_2P$ calcd. For 405.0572, found 405.0573.

2-chloro-1-(4-chlorophenyl)-2-phenylethyl diphenylphosphinate (1q)

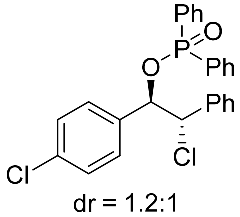

SY33

According to **Procedure 5**. Yield: 932.0 mg, 67%; two diastereomers, dr = 1.2:1; colorless oil; two diastereomers, dr = 1.2/1; ^{1}H NMR (400 MHz, $CDCl_3$) δ 7.85-7.74 (m, 1H), 7.60-7.41 (m, 5H), 7.39-7.31 (m, 2H), 7.29-7.20 (m, 5H), 7.16-7.10 (m, 2H), 7.09-7.04 (m, 2H), 7.03-6.98 (m, 1H), 6.89-6.85 (m, 1H), 5.68-5.58 (m, 1H), 5.28 (d, J = 7.6 Hz, 0.45H, the minor isomer), 5.23 (d, J = 6.4 Hz, 0.55H, the major isomer) ppm; ^{13}C NMR (100 MHz, $CDCl_3$) (difficult to assign every peak) δ 137.0 (the major isomer), 136.1 (the minor isomer), 134.83, 134.80, 134.60, 134.40, 132.42, 132.40, 132.29, 132.26, 132.21, 132.18, 132.15, 131.85, 131.75, 131.73, 131.72, 131.63, 131.62, 131.55, 131.50, 131.40, 130.69, 130.50, 130.38, 130.21, 129.93, 129.51, 129.24, 128.86, 128.70, 128.59, 128.49, 128.46, 128.36, 128.34, 128.30, 128.21, 128.17, 128.06, 128.01, 127.34, 79.9 (d, J_{C-P} = 5.8 Hz, the minor isomer), 79.3 (d, J_{C-P} = 5.7 Hz, the major isomer), 65.44 (d, J_{C-P} = 6.8 Hz, the minor isomer), 65.37 (d, J_{C-P} = 7.4 Hz, the major isomer) ppm; ^{31}P NMR (162 MHz, $CDCl_3$) δ 33.90 (1P, the major isomer), 33.70 (0.83P, the minor isomer) ppm; HRMS (ESI) (*m/z*) [M+H]$^+$ $C_{26}H_{22}{}^{35}Cl_2O_2P$ calcd. For 467.0729, found 467.0732.

2-bromo-1-(4-bromophenyl)propyl diphenylphosphinate (1r)

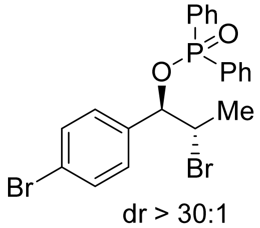

XT492

According to **Procedure 5**. Yield: 1.15 g, 78%; dr > 30:1; colorless oil; ^{1}H NMR (400 MHz, $CDCl_3$) δ 7.80-7.72 (m, 2H), 7.53-7.42 (m, 2H), 7.40-7.28 (m, 3H), 7.29-7.23 (m, 2H), 7.22-7.16 (m, 2H), 7.10-

7.00 (m, 2H), 5.29 (dd, *J* = 9.2, 6.0 Hz, 1H), 4.37-4.29 (m, 1H), 1.48 (d, *J* = 6.8, 3H) ppm; ^{13}C NMR (100 MHz, $CDCl_3$) δ 135.8 (d, J_{C-P} = 2.6 Hz), 132.4 (d, J_{C-P} = 2.9 Hz), 132.2 (d, J_{C-P} = 2.7 Hz), 131.8 (d, J_{C-P} = 10.4 Hz), 131.6 (d, J_{C-P} = 10.4 Hz), 131.3, 131.2 (d, J_{C-P} = 138.6 Hz), 130.8 (d, J_{C-P} = 133.5 Hz), 129.2, 128.6 (d, J_{C-P} = 13.4 Hz), 128.3 (d, J_{C-P} = 13.2 Hz), 122.8, 79.6 (d, J_{C-P} = 5.6 Hz), 51.0 (d, J_{C-P} = 5.7 Hz), 21.5 ppm; ^{31}P NMR (162 MHz, $CDCl_3$) δ 33.4 ppm; HRMS (ESI) (*m/z*) $[M+H]^+$ $C_{21}H_{20}{}^{79}Br_2O_2P$ calcd. For 492.9562, found 492.9564.

2-bromo-1-(4-((diphenylphosphoryl)oxy)phenyl)ethyl diphenylphosphinate (1s)

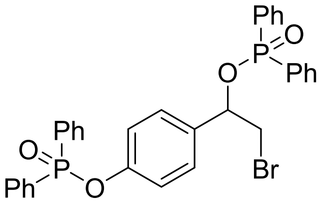

XT513

According to **Procedure 5**. Yield: 1.40 g, 76%; white solid, m.p. 155-157 °C; ^{1}H NMR (400 MHz, $CDCl_3$) δ 8.02-7.87 (m, 6H), 7.72-7.45 (m, 13H), 7.38-7.30 (m, 2H), 7.29-7.21 (m, 4H), 5.56-5.49 (m, 1H), 3.99 (dd, *J* = 7.6, 6.0 Hz, 1H), 3.85-3.79 (m, 1H) ppm; ^{13}C NMR (100 MHz, $CDCl_3$) δ 151.2 (d, J_{C-P} = 8.2 Hz), 133.7 (d, J_{C-P} = 2.9 Hz), 132.6 (d, J_{C-P} = 2.8 Hz), 132.4 (d, J_{C-P} = 2.8 Hz), 132.3 (d, J_{C-P} = 2.7 Hz), 131.82 (d, J_{C-P} = 10.4 Hz), 131.81 (d, J_{C-P} = 1.7 Hz), 131.7 (d, J_{C-P} = 1.6 Hz), 131.6 (d, J_{C-P} = 10.3 Hz), 131.13 (d, J_{C-P} = 138.4 Hz), 130.87 (d, J_{C-P} = 137.2 Hz), 130.74 (d, J_{C-P} = 133.6 Hz), 128.7 (d, J_{C-P} = 13.4 Hz), 128.5 (d, J_{C-P} = 13.4 Hz),128.33 (d, J_{C-P} = 13.2 Hz), 128.31, 120.8 (d, J_{C-P} = 4.9 Hz), 76.0 (d, J_{C-P} = 5.5 Hz), 47.7 (d, J_{C-P} = 4.7 Hz) ppm; ^{31}P NMR (162 MHz, $CDCl_3$) δ 33.6, 31.5 ppm; HRMS (ESI) (*m/z*) $[M+H]^+$ $C_{32}H_{28}{}^{79}BrO_4P_2$ calcd. For 617.0641, found 617.0639.

2-chloro-1-phenylpentyl diphenylphosphinate (1t)

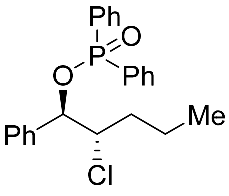

dr > 30:1 XT214

According to **Procedure 5**. Yield: 979.0 mg, 82%; dr > 30:1; white solid, m.p. 75-76 °C; ^{1}H NMR (400 MHz, $CDCl_3$) δ 7.93-7.83 (m, 2H), 7.63-7.52 (m, 3H), 7.51-7.45 (m, 2H), 7.42-7.36 (m, 1H), 7.31-7.22 (m, 7H), 5.47 (dd, *J* = 5.6, 9.2 Hz, 1H), 4.31-4.21 (m, 1H), 1.74-1.64 (m, 1H), 1.63-1.45 (m, 2H), 1.44-1.32 (m, 1H), 0.83 (t, *J* = 7.2 Hz, 3H) ppm; ^{13}C NMR (100 MHz, $CDCl_3$) δ 137.0 (d, J_{C-P} = 2.6 Hz), 132.2 (d, J_{C-P} = 2.7 Hz), 132.0 (d, J_{C-P} = 2.8 Hz), 131.9 (d, J_{C-P} = 10.5 Hz), 131.7 (d, J_{C-P} = 139.0 Hz), 131.6 (d, J_{C-P} = 10.3 Hz), 131.1 (d, J_{C-P} = 138.7 Hz), 128.53, 128.49 (d, J_{C-P} = 13.2 Hz), 128.126,

128.124 (d, J_{C-P} = 13.2 Hz), 127.5, 79.7 (d, J_{C-P} = 5.7 Hz), 65.1 (d, J_{C-P} = 5.3 Hz), 35.5, 19.5, 13.4 ppm; ^{31}P NMR (162 MHz, $CDCl_3$) δ 33.0 ppm; HRMS (ESI) (*m/z*) $[M+H]^+$ $C_{23}H_{25}{}^{35}ClO_2P$ calcd. for 399.1275, found 399.1279.

2-chloro-1-phenyldodecyl diphenylphosphinate (1u)

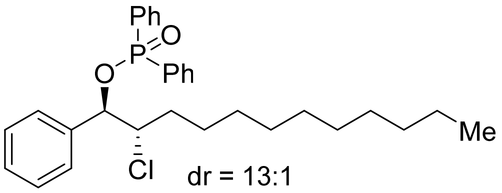

dr = 13:1

SY15

According to **Procedure 5**. Yield: 1.09 g, 73%; two diastereomers, dr = 13:1; colorless oil; ^{1}H NMR (400 MHz, $CDCl_3$) δ 7.91-7.82 (m, 2H), 7.62-7.50 (m, 3H), 7.49-7.42 (m, 2H), 7.40-7.33 (m, 1H), 7.30-7.20 (m, 2H), 7.46 (dd, *J* = 9.6, 6.0 Hz, 1H), 4.28-4.20 (m, 1H), 1.78-1.65 (m, 1H), 1.55-1.42 (m, 2H), 1.34-1.11 (m, 15H), 0.87 (t, *J* = 6.8, 3H) ppm; ^{13}C NMR (100 MHz, $CDCl_3$) (the major isomer) δ 137.1 (d, J_{C-P} = 2.5 Hz), 132.2 (d, J_{C-P} = 2.7 Hz), 132.0 (d, J_{C-P} = 2.7 Hz), 131.86 (d, J_{C-P} = 10.4 Hz), 131.76 (d, J_{C-P} = 138.9 Hz), 131.67 (d, J_{C-P} = 10.3 Hz), 131.2 (d, J_{C-P} = 133.7 Hz), 128.54, 128.49 (d, J_{C-P} = 13.2 Hz), 128.139, 128.136 (d, J_{C-P} = 13.2 Hz), 127.6, 79.7 (d, J_{C-P} = 5.8 Hz), 65.4 (d, J_{C-P} = 5.4 Hz), 33.5, 31.9, 29.57, 29.51, 29.38, 29.32, 28.9, 26.3, 22.7, 14.1 ppm; ^{31}P NMR (162 MHz, $CDCl_3$) δ 33.0 ppm; HRMS (ESI) (*m/z*) $[M+H]^+$ $C_{30}H_{39}{}^{35}ClO_2P$ calcd. for 497.2371, found 497.2372.

2-chloro-1,3-diphenylpropyl diphenylphosphinate (1v)

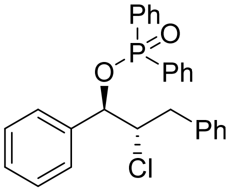

dr > 30:1

XT259

According to **Procedure 5**. Yield: 990.0 mg, 74%; dr > 30:1; white solid, m.p. 127-129 °C; ^{1}H NMR (400 MHz, $CDCl_3$) δ 7.95-7.85 (m, 2H), 7.64-7.43 (m, 5H), 7.42-7.16 (m, 12H), 7.15-7.07 (m, 2H), 5.62-5.22 (m, 1H), 4.48-4.42 (m, 1H), 3.30-3.18 (m, 1H), 2.75-2.65 (m, 1H) ppm; ^{13}C NMR (100 MHz, $CDCl_3$) (the major isomer) δ ppm; 137.4, 136.7 (d, J_{C-P} = 1.9 Hz), 132.4, 132.2, 131.9 (d, J_{C-P} = 10.2 Hz), 131.73 (d, J_{C-P} = 142.1 Hz) 131.67 (d, J_{C-P} = 10.0 Hz), 131.0 (d, J_{C-P} = 134.8 Hz), 129.4, 128.72, 128.66 (d, J_{C-P} = 12.0 Hz), 128.4, 128.29 (d, J_{C-P} = 13.2 Hz), 128.25, 127.6, 126.8, 79.1 (d, J_{C-P} = 4.9 Hz), 66.0 (d, J_{C-P} = 4.3 Hz), 39.9 ppm; ^{31}P NMR (162 MHz, $CDCl_3$) δ 33.1 ppm; HRMS (ESI) (*m/z*) $[M+H]^+$ $C_{27}H_{25}{}^{35}ClO_2P$ calcd. for 447.1275, found 447.1275.

(1-chlorocyclobutyl)(phenyl)methyl diphenylphosphinate (1w)

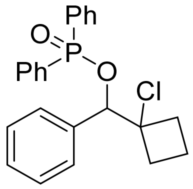

XT222

According to **Procedure 5**. Yield: 1.06 g, 89%; white solid, m.p. 124-125 °C; ^{1}H NMR (400 MHz, $CDCl_3$) δ 7.98-7.86 (m, 2H), 7.66-7.47 (m, 5H), 7.41-7.33 (m, 3H), 7.31-7.20 (m, 5H), 5.58 (d, *J* = 10.8 Hz, 1H), 3.00-2.89 (m, 1H), 2.76-2.66 (m, 1H), 2.46-2.37 (m, 1H), 2.35-2.26 (m, 1H), 2.26-2.15 (m, 1H), 1.93-1.81 (m, 1H) ppm; ^{13}C NMR (100 MHz, $CDCl_3$) δ 136.3 (d, $J_{C\text{-}P}$ = 2.1 Hz), 132.26 (d, $J_{C\text{-}P}$ = 2.8 Hz), 131.93 (d, $J_{C\text{-}P}$ = 2.5 Hz), 131.88 (d, $J_{C\text{-}P}$ = 10.7 Hz), 131.80 (d, $J_{C\text{-}P}$ = 140.4 Hz), 131.51 (d, $J_{C\text{-}P}$ = 10.1 Hz), 131.06 (d, $J_{C\text{-}P}$ = 142.2 Hz), 128.5 (d, $J_{C\text{-}P}$ = 13.3 Hz), 128.4, 128.3, 128.0 (d, $J_{C\text{-}P}$ = 13.2 Hz), 127.6, 80.4 (d, $J_{C\text{-}P}$ = 5.9 Hz), 72.7 (d, $J_{C\text{-}P}$ = 5.5 Hz), 36.0, 35.1, 15.0 ppm; ^{31}P NMR (162 MHz, $CDCl_3$) δ 33.2 ppm; HRMS (ESI) (*m/z*) $[M+H]^+$ $C_{23}H_{23}{}^{35}ClO_2P$ calcd. for 397.1119, found 397.1119.

(1-chlorocyclobutyl)(phenyl)methyl diphenylphosphinate (1x)

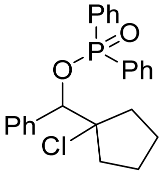

XT248

According to **Procedure 5**. Yield: 1.17 g, 95%; white solid, m.p. 92-93 °C; ^{1}H NMR (400 MHz, $CDCl_3$) δ 8.01-7.98 (m, 2H), 7.65-7.50 (m, 5H), 7.43-7.35 (m, 3H), 7.30-7.20 (m, 5H), 5.55 (d, *J* = 10.8 Hz, 1H), 2.31-2.20 (m, 2H), 2.17-2.07 (m, 1H), 2.06-1.92 (m, 2H), 1.89-1.73 (m, 3H) ppm; ^{13}C NMR (100 MHz, $CDCl_3$) δ 137.3 (d, $J_{C\text{-}P}$ = 1.7 Hz), 132.25 (d, $J_{C\text{-}P}$ = 2.8 Hz), 131.89 (d, $J_{C\text{-}P}$ = 10.4 Hz), 131.85 (d, $J_{C\text{-}P}$ = 2.7 Hz), 131.7 (d, $J_{C\text{-}P}$ = 140.8 Hz), 131.5 (d, $J_{C\text{-}P}$ = 10.1 Hz), 131.02 (d, $J_{C\text{-}P}$ = 132.0 Hz), 128.5 (d, $J_{C\text{-}P}$ = 13.3 Hz), 128.4, 128.3, 127.9 (d, $J_{C\text{-}P}$ = 13.2 Hz), 127.5, 83.2 (d, $J_{C\text{-}P}$ = 5.9 Hz), 81.6 (d, $J_{C\text{-}P}$ = 5.9 Hz), 40.5, 39.4, 22.9 ppm; ^{31}P NMR (162 MHz, $CDCl_3$) δ 32.7 ppm; HRMS (ESI) (*m/z*) $[M+H]^+$ $C_{24}H_{25}{}^{35}ClO_2P$ calcd. for 411.1275, found 411.1277.

2-bromo-1-(naphthalen-2-yl)ethyl diphenylphosphinate (1y)

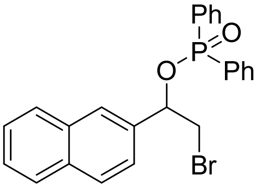

XT396

According to **Procedure 5**. Yield: 1.00 g, 71%; white solid, m.p. 94-95 °C; ^{1}H NMR (400 MHz, $CDCl_3$) δ 7.72-7.64 (m, 2H), 7.63-7.52 (m, 3H), 7.49-7.41 (m, 3H), 7.37-7.31 (m, 1H), 7.30-7.20 (m, 5H), 7.20-

7.13 (m, 1H), 7.07-6.99 (m, 2H), 5.55-5.44 (m, 1H), 3.73 (dd, J = 6.0, 10.8 Hz, 1H), 3.59 (dd, J = 6.4, 10.8 Hz, 1H) ppm; ^{13}C NMR (100 MHz, $CDCl_3$) δ 135.0 (d, J_{C-P} = 3.7 Hz), 133.4, 132.9, 132.4 (d, J_{C-P} = 2.8 Hz), 132.2 (d, J_{C-P} = 2.7 Hz), 131.9 (d, J_{C-P} = 10.3 Hz), 131.7 (d, J_{C-P} = 10.3 Hz), 131.5 (d, J_{C-P} = 139.0 Hz), 130.9 (d, J_{C-P} = 133.8 Hz), 128.57 (d, J_{C-P} = 13.4 Hz), 128.53, 128.3 (d, J_{C-P} = 13.2 Hz), 128.2, 127.7, 126.8, 126.6, 126.4, 123.8, 76.7 (d, J_{C-P} = 5.5 Hz), 35.8 (d, J_{C-P} = 4.7 Hz) ppm; ^{31}P NMR (162 MHz, $CDCl_3$) δ 33.5 ppm; HRMS (ESI) (*m/z*) $[M+H]^+$ $C_{24}H_{20}{}^{79}BrNaO_2P$ calcd. for 473.0277, found 473.0274.

2-chloro-1-(3-oxo-3,4-dihydro-2*H*-benzo[*b*][1,4]oxazin-6-yl)ethyl diphenylphosphinate (1z)

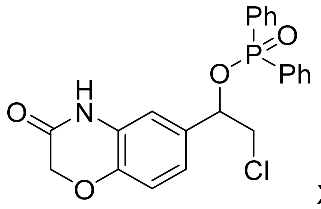

XT377

According to **Procedure 5**. Yield: 880.0 mg, 65%; pale yellow oil; ^{1}H NMR (400 MHz, Acetone-d_6) δ 9.84 (brs, 1H), 7.93-7.85 (m, 2H), 7.77-7.68 (m, 2H), 7.64-7.58 (m, 1H), 7.56-7.48 (m, 3H), 7.44-7.37 (m, 2H), 7.07-7.02 (m, 1H), 6.97 (dd, J = 8.4, 2.0 Hz, 1H), 6.84 (d, J = 8.4 Hz, 1H), 5.48-5.39 (m, 1H), 4.53 (s, 2H), 3.98 (dd, J = 6.4, 11.6 Hz, 1H), 3.89 (dd, J = 5.2, 11.6 Hz, 1H) ppm; ^{13}C NMR (100 MHz, Acetone-d_6) δ 169.6, 149.0, 137.6, 137.5, 137.24 (d, J_{C-P} = 3.0 Hz), 137.15 (d, J_{C-P} = 138.5 Hz), 137.0 (d, J_{C-P} = 135.5 Hz), 136.9 (d, J_{C-P} = 4.3 Hz), 136.8 (d, J_{C-P} = 3.4 Hz), 133.9 (d, J_{C-P} = 3.4 Hz), 133.6 (d, J_{C-P} = 3.2 Hz), 132.6, 127.0, 121.3, 120.0, 76.23, 76.18, 66.94, 47.95, 47.89 ppm; ^{31}P NMR (162 MHz, Acetone-d_6) δ 32.5 ppm; HRMS (ESI) (*m/z*) $[M+H]^+$ $C_{22}H_{19}{}^{35}ClNNaO_4P$ calcd. for 450.0632, found 450.0635.

1-chloro-3-phenylpropan-2-yl diphenylphosphinate (1aa)

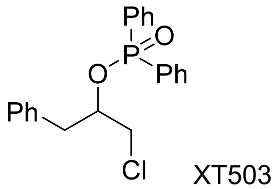

XT503

According to **Procedure 8**. Yield: 1.57 g, 85%; white solid, m.p. 109-111 °C; ^{1}H NMR (400 MHz, $CDCl_3$) δ 7.86-7.76 (m, 2H), 7.65-7.56 (m, 2H), 7.55-7.42 (m, 4H), 7.41-7.33 (m, 2H), 7.31-7.22 (m, 3H), 7.21-7.14 (m, 2H), 4.80-4.70 (m, 1H), 3.74-3.65 (m, 2H), 3.24-3.05 (m, 2H) ppm; ^{13}C NMR (100 MHz, $CDCl_3$) δ 136.1, 132.3 (d, J_{C-P} = 2.5 Hz), 132.2 (d, J_{C-P} = 2.5 Hz), 131.73 (d, J_{C-P} = 10.2 Hz), 131.66 (d, J_{C-P} = 135.2 Hz), 131.29 (d, J_{C-P} = 10.2 Hz), 131.15 (d, J_{C-P} = 134.5 Hz), 129.7, 128.6 (d, J_{C-P} = 13.0 Hz), 128.5 (d, J_{C-P} = 12.9 Hz), 127.0, 75.6 (d, J_{C-P} = 6.1 Hz), 46.2 (d, J_{C-P} = 2.7 Hz), 39.2 (d, J_{C-P} = 4.7 Hz) ppm; ^{31}P NMR (162 MHz, $CDCl_3$) δ 32.8 ppm; HRMS (ESI) (*m/z*) $[M+H]^+$ $C_{21}H_{21}{}^{35}ClO_2P$ calcd. for 371.0962, found 371.0969.

2-chloro-1-((*S*)-4-(prop-1-en-2-yl)cyclohex-1-en-1-yl)ethyl diphenylphosphinate (1ab)

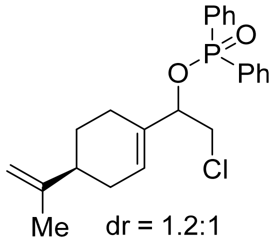

XT384

According to **Procedure 9**. Yield: 852.0 mg, 71%; two diastereomers, dr = 1.2/1; colorless oil; ^{1}H NMR (400 MHz, CDCl$_3$) δ 7.78-7.65 (m, 4H), 7.47-7.29 (m, 6H), 5.67-5.54 (m, 1H), 4.89-4.77 (m, 1H), 4.65-4.51 (m, 2H), 3.77-3.66 (m, 1H), 3.58-3.49 (m, 1H), 2.15-1.70 (m, 5H), 1.69-1.61 (m, 2H), 1.61 (s, 1.4H, the minor isomer), 1.59 (s, 1.68H, the major isomer) ppm; ^{13}C NMR (100 MHz, CDCl$_3$) δ (not possible to assign every peak) 149.4, 149.1, 132.68, 132.66, 132.56, 132.52, 132.49, 132.40, 132.37, 132.24, 132.20, 132.17, 132.15, 132.10, 132.08, 132.05, 132.00, 131.90, 131.64, 131.54, 131.44, 131.17, 131.02, 129.85, 128.61, 128.54, 128.53, 128.41, 128.39, 128.36, 128.25, 128.23, 108.91, 108.85, 79.21, 79.15, 79.01, 78.96, 45.21, 45.15, 44.65, 44.59, 40.73, 40.26, 30.51, 30.25, 26.89, 26.83, 23.54, 23.24, 20.78, 20.56 ppm; ^{31}P NMR (162 MHz, CDCl$_3$) δ 31.9 (0.82P, the minor isomer), 31.7 (1P, the major isomer) ppm; HRMS (ESI) (*m/z*) [M+H]$^+$ $C_{23}H_{27}{}^{35}ClO_2P$ calcd. for 401.1432, found 401.1427.

2-chloro-1-((1*R*,5*S*)-6,6-dimethylbicyclo[3.1.1]hept-2-en-2-yl)ethyl diphenylphosphinate (1ac)

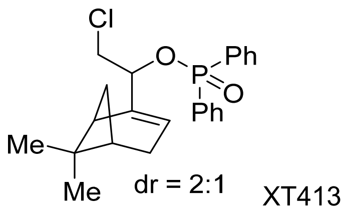

XT413

According to **Procedure 9**. Yield: 672.0 mg, 56%; two diastereomers, dr = 2/1; colorless oil; ^{1}H NMR (400 MHz, CDCl$_3$) δ 7.85-7.73 (m, 4H), 7.52-7.45 (m, 2H), 7.45-7.37 (m, 4H), 5.57-5.47 (m, 1H), 4.92-4.78 (m, 1H), 3.73 (dd, *J* = 5.2, 8.4 Hz, 0.66H, the major isomer), 3.68 (dd, *J* = 6.4, 11.6 Hz, 0.34H, the minor isomer), 3.62-3.52 (m, 1H), 2.39-2.29 (m, 1H), 2.26-2.11 (m, 3H), 2.09-1.98 (m, 1H), 1.26 (s, 2H, the major isomer), 1.23 (s, 1H, the minor isomer), 1.09 (d, *J* = 8.8 Hz, 0.66H, the major isomer), 0.94 (d, *J* = 8.8 Hz, 0.36H, the minor isomer), 0.81 (s, 2H, the major isomer), 0.73 (s, 1H, the minor isomer) ppm; ^{13}C NMR (100 MHz, CDCl$_3$) δ (not possible to assign every peak) 143.40, 143.37, 143.12, 143.07, 132.59, 132.26, 132.23, 132.22, 132.19, 132.16, 132.13, 132.11, 132.01, 131.98, 131.90, 131.88, 131.68, 131.60, 131.58, 131.50, 131.21, 130.77, 128.52, 128.50, 128.47, 128.42, 128.39, 128.37, 128.34, 128.29, 123.75, 123.70, 77.35, 77.29, 45.57, 45.52, 44.56, 44.53, 41.88, 41.68, 40.69, 40.52, 38.02, 37.83, 31.74, 31.42, 31.22, 26.18, 26.12, 21.42 ppm; ^{31}P NMR (162 MHz, CDCl$_3$) δ 32.5 (2P, the major isomer), 32.0 (1P, the major isomer) ppm; HRMS (ESI) (*m/z*) [M+H]$^+$ $C_{23}H_{27}{}^{35}ClO_2P$ calcd. for 401.1432, found 423.1250.

(1*R*,2*S*,4*S*)-2-isopropyl-4-methylcyclohexyl 4-(2-chloro-1-((diphenylphosphoryl)oxy)ethyl) benzoate (1ad)

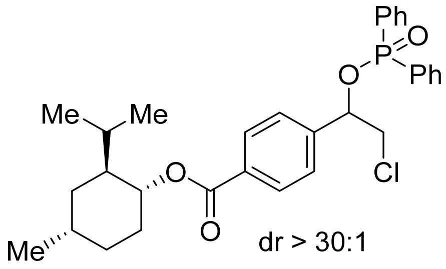

XT446

According to **Procedure 5**. Yield: 1.45 g, 90%; dr > 30:1; colorless oil; ^{1}H NMR (400 MHz, $CDCl_3$) δ 7.98 (d, *J* = 7.6 Hz, 2H), 7.90-7.81 (m, 2H), 7.71-7.62 (m, 2H), 7.58-7.51 (m, 1H), 7.50-7.42 (m, 3H), 7.38 (d, *J* = 8.4 Hz, 2H), 7.36-7.30 (m, 2H), 5.61-5.49 (m, 1H), 4.92 (dt, *J* = 4.4, 10.8 Hz, 1H), 4.00-3.91 (m, 1H), 3.87-3.76 (m, 1H), 2.14-2.05 (m, 1H), 1.99-1.87 (m, 1H), 1.78-1.67 (m, 2H), 1.62-1.48 (m, 2H), 1.20-1.03 (m, 2H), 0.99-0.94 (m, 1H), 0.92 (dd, *J* = 6.8, 2.4 Hz, 6H), 0.79 (d, *J* = 6.8 Hz, 3H) ppm; ^{13}C NMR (100 MHz, $CDCl_3$) δ 165.6, 142.0 (d, J_{C-P} = 3.4 Hz), 132.5 (d, J_{C-P} = 2.7 Hz), 132.4 (d, J_{C-P} = 1.7 Hz), 131.8 (d, J_{C-P} = 10.4 Hz), 131.6 (d, J_{C-P} = 10.3 Hz), 131.3, 131.2 (d, J_{C-P} = 137.7 Hz), 130.8 (d, J_{C-P} = 134.2 Hz), 129.8 (d, J_{C-P} = 0.8 Hz), 128.6 (d, J_{C-P} = 13.3 Hz), 128.5 (d, J_{C-P} = 13.2 Hz), 126.8, 76.0 (d, J_{C-P} = 5.4 Hz), 75.0, 47.7 (d, J_{C-P} = 4.5 Hz), 47.3, 41.0, 34.3, 31.5, 26.5, 23.6, 22.1, 20.8, 16.5 ppm; ^{31}P NMR (162 MHz, $CDCl_3$) δ 33.9 ppm; HRMS (EI) (*m/z*) $C_{31}H_{37}{}^{35}ClO_4P$ calcd. for 539.2113, found 539.2118.

2,2-dichloro-2-phenyl-1-(4-(trifluoromethyl)phenyl)ethyl diphenylphosphinate (1ah)

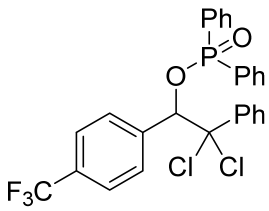

SY43-1

According to **Procedure 5**. Yield: 1.35 g, 84%; white solid, m.p. 118-119 °C; ^{1}H NMR (400 MHz, $CDCl_3$) δ 7.76-7.65 (m, 4H), 7.57-7.52 (m, 1H), 7.51-7.41 (m, 6H), 7.37-7.31 (m, 1H), 7.23-7.13 (m, 3H), 7.09-7.02 (m, 4H), 6.01 (d, *J* = 10.0 Hz, 1H) ppm; ^{13}C NMR (100 MHz, $CDCl_3$) peaks of the major isomer δ 142.6, 133.8 (d, J_{C-P} = 0.6 Hz), 132.4 (d, J_{C-P} = 2.8 Hz), 132.1 (d, J_{C-P} = 2.8 Hz), 131.55 (d, J_{C-P} = 10.6 Hz), 131.4 (q, J_{C-F} = 32.6 Hz), 131.00 (d, J_{C-P} = 134.4 Hz), 130.80 (d, J_{C-P} = 139.6 Hz), 129.5, 129.1, 128.9, 128.5 (d, J_{C-P} = 13.4 Hz), 128.1 (d, J_{C-P} = 13.4 Hz), 127.3, 124.7 (q, J_{C-F} = 3.6 Hz), 123.7 (q, J_{C-F} = 270.6 Hz), 92.3 (d, J_{C-P} = 7.8 Hz), 83.7 (d, J_{C-P} = 5.6 Hz) ppm; ^{31}P NMR (162 MHz, $CDCl_3$) δ 34.4 ppm; ^{19}F NMR (377 MHz, $CDCl_3$) δ -63.2 ppm; HRMS (ESI) (*m/z*) $[M+H]^+$ $C_{27}H_{21}{}^{35}Cl_2F_3O_2P$ calcd. for 535.0603, found 535.0608.

1,2-diphenylethyl diphenylphosphinate (1ai)

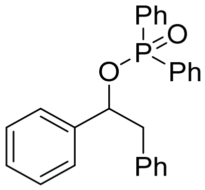

XT216

According to **Procedure 4**. Yield: 860 mg, 72%; white solid, m.p. 140-141 °C; ^{1}H NMR (400 MHz, $CDCl_3$) δ 7.80-7.66 (m, 4H), 7.62-7.55 (m, 1H), 7.53-7.44 (m, 3H), 7.41-7.34 (m, 2H), 7.33-7.29 (m, 3H), 7.29-7.22 (m, 5H), 7.14-7.03 (m, 2H), 5.68-5.56 (m, 1H), 3.49 (dd, *J* = 6.4, 13.2 Hz, 1H), 3.31 (dd, *J* = 6.8, 13.2 Hz, 1H) ppm; ^{13}C NMR (100 MHz, $CDCl_3$) δ 140.0 (d, $J_{C\text{-}P}$ = 3.4 Hz), 136.5, 132.05 (d, $J_{C\text{-}P}$ = 137.8 Hz), 131.95 (d, $J_{C\text{-}P}$ = 2.7 Hz), 131.88, 131.84 (d, $J_{C\text{-}P}$ = 10.3 Hz), 131.57 (d, $J_{C\text{-}P}$ = 134.3 Hz), 131.55 (d, $J_{C\text{-}P}$ = 10.2 Hz), 130.0, 128.4 (d, $J_{C\text{-}P}$ = 13.1 Hz), 128.19 (d, $J_{C\text{-}P}$ = 12.9 Hz), 128.15 (one carbon is overlapping), 128.0, 126.7, 126.6, 79.0 (d, $J_{C\text{-}P}$ = 6.0 Hz), 45.3 (d, $J_{C\text{-}P}$ = 4.6 Hz) ppm; ^{31}P NMR (162 MHz, $CDCl_3$) δ 32.1 ppm; HRMS (ESI) (*m/z*) $[M+H]^+$ $C_{26}H_{24}O_2P$ calcd. for 399.1508, found 399.1508.

(5*R*)-2-methyl-5-(prop-1-en-2-yl)cyclohex-2-en-1-yl diphenylphosphinate (1aj)

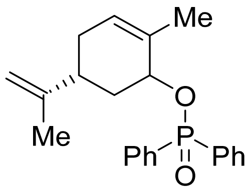

dr = 2:1

XT267

According to **Procedure 4**. Yield: 802.0 mg, 76%; two diastereomers, dr = 2:1; colorless oil; ^{1}H NMR (400 MHz, $CDCl_3$) δ 7.91-7.73 (m, 4H), 7.54-7.32 (m, 6H), 5.73-5.65 (m, 0.33H, the minor isomer), 5.61-5.53 (m, 0.67H, the major isomer), 5.10-4.99 (m, 0.67H, the major isomer), 4.89-4.83 (m, 0.33H, the minor isomer), 4.73-4.54 (m, 2H), 2.53-1.70 (m, 5H), 1.70 (s, 2H, the major isomer), 1.68 (s, 1H, the minor isomer), 1.62 (s, 3H) ppm; ^{13}C NMR (100 MHz, $CDCl_3$) (difficult to assign every peak) δ 148.4 (the minor isomer), 148.1 (the major isomer), 133.69, 133.63, 133.43, 133.30, 133.15, 132.80, 132.07, 132.04, 132.00, 131.97, 131.94, 131.80, 131.75, 131.70, 131.63, 131.61, 131.60, 131.53, 131.44, 128.49, 128.45, 128.36, 128.32, 128.02, 126.21, 109.38, 109.25, 75.63, 75.57, 73.25, 73.18, 40.37, 36.52, 35.40, 35.23, 30.74, 20.83, 20.40, 19.80 ppm; ^{31}P NMR (162 MHz, $CDCl_3$) δ 31.3 (1P, the major isomer), 31.1 (0.5P, the minor isomer) ppm; HRMS (ESI) (*m/z*) $[M+H]^+$ $C_{22}H_{26}O_2P$ calcd. for 353.1665, found 353.1664.

3-chloro-1-(4-fluorophenyl)propyl diphenylphosphinate (1ak)

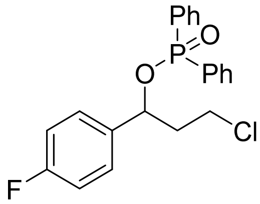

XT418

According to **Procedure 4**. Yield: 817.0 mg, 70%; colorless oil; ^{1}H NMR (400 MHz, $CDCl_3$) δ 7.91-7.81 (m, 2H), 7.67-7.54 (m, 3H), 7.54-7.42 (m, 3H), 7.37-7.29 (m, 2H), 7.29-7.22 (m, 2H), 7.05-6.93 (m, 2H), 5.60-5.48 (m, 1H), 3.73-3.60 (m, 1H), 3.53-3.42 (m, 1H), 2.67-2.53 (m, 1H), 2.32-2.21 (m, 1H) ppm; ^{13}C NMR (100 MHz, $CDCl_3$) δ 163.6 (d, $J_{C\text{-}F}$ = 245.6 Hz), 135.4 (dd, *J* = 3.1, 3.2 Hz), 132.3 (d, $J_{C\text{-}P}$ = 2.8 Hz), 132.1 (d, $J_{C\text{-}P}$ = 2.8 Hz), 131.9 (d, $J_{C\text{-}P}$ = 10.3 Hz), 131.7 (d, $J_{C\text{-}P}$ = 139.0 Hz), 131.4 (d, $J_{C\text{-}P}$ = 10.1 Hz), 131.0 (d, $J_{C\text{-}P}$ = 132.5 Hz), 128.6 (d, $J_{C\text{-}P}$ = 13.2 Hz), 128.4 (d, $J_{C\text{-}F}$ = 8.6 Hz), 128.2 (d, $J_{C\text{-}P}$ = 13.3 Hz), 115.5 (d, $J_{C\text{-}F}$ = 21.4 Hz), 74.8 (d, $J_{C\text{-}P}$ = 5.8 Hz), 41.1 (d, $J_{C\text{-}P}$ = 4.6 Hz), 40.5 ppm; ^{31}P NMR (162 MHz, $CDCl_3$) δ 32.5 ppm; ^{19}F NMR (377 MHz, $CDCl_3$) δ -113.8 ppm; HRMS (ESI) (*m/z*) $[M+H]^+$ $C_{21}H_{20}{}^{35}ClFO_2P$ calcd. for 389.0868, found 389.0868.

(3s,5s,7s)-adamantan-1-yl diphenylphosphinate (1al)

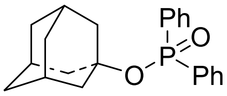

TK1227

According to **Procedure 6**. Yield: 1.32 g, 75%; white solid, m.p. 126-127 °C; ^{1}H NMR (400 MHz, $CDCl_3$) δ 7.82-7.76 (m, 4H), 7.47-7.37 (m, 6H), 2.15-2.05 (m, 9H), 1.59 (s, 6H) ppm; ^{13}C NMR (100 MHz, $CDCl_3$) δ 134.8 (d, $J_{C\text{-}P}$ = 137.5 Hz), 131.5 (d, $J_{C\text{-}P}$ = 2.8 Hz), 131.4 (d, $J_{C\text{-}P}$ = 10.2 Hz), 128.3 (d, $J_{C\text{-}P}$ = 13.1 Hz), 83.5 (d, $J_{C\text{-}P}$ = 8.7 Hz), 44.6 (d, $J_{C\text{-}P}$ = 3.9 Hz), 35.7, 31.2 ppm; ^{31}P NMR (162 MHz, $CDCl_3$) δ 26.5 ppm; HRMS (ESI) (*m/z*) $[M+H]^+$ $C_{22}H_{26}O_2P$ calcd. for 353.1665, found 353.1662.

2,4,5-triphenyl-1,3,2-dioxaphospholane 2-oxide (4a)

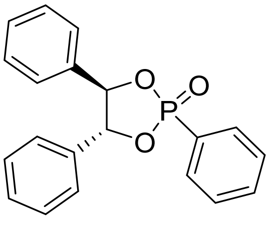

XT37

According to **Procedure 10**. Yield: 756.0 mg, 75%; white solid, m.p. 165-166 °C; ^{1}H NMR (400 MHz, $CDCl_3$) δ 8.08-7.98 (m, 2H), 7.70-7.64 (m, 1H), 7.62-7.54 (m, 2H), 7.44-7.33 (m, 8H), 7.25-7.17 (m, 2H), 5.55 (d, *J* = 9.2 Hz, 1H), 5.40 (dd, *J* = 1.6, 9.2 Hz, 1H) ppm; ^{13}C NMR (100 MHz, $CDCl_3$) δ 134.5 (d, $J_{C\text{-}P}$ = 10.0 Hz), 134.3 (d, $J_{C\text{-}P}$ = 7.4 Hz), 133.4 (d, $J_{C\text{-}P}$ = 3.2 Hz), 132.3, 132.2, 129.5, 129.4, 128.98, 128.92, 128.88, 128.82, 127.0, 126.9 (d, $J_{C\text{-}P}$ = 184.5 Hz), 126.5, 88.4 (d, $J_{C\text{-}P}$ = 1.7 Hz), 85.3 ppm; ^{31}P NMR (162 MHz, $CDCl_3$) δ 34.07 ppm; HRMS (ESI) (*m/z*) $[M+H]^+$ $C_{20}H_{18}O_3P$ calcd. for

337.0988, found 337.0994. Literature NMR data is not available in $CDCl_3$, but the compound has been previously reported.[12]

9. Characterization Data: e-PRC Phosphinate Reduction Products

(*E*)-1,2-diphenylethene (*E*-2a)

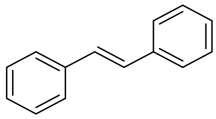

XT98

From the reaction of **1a** with **DCA** as e-PRCat according to **Procedure 11**, for 12 h.

Yield: 28.0 mg, 75%; colorless solid, m.p. 124 °C; ^{1}H NMR (400 MHz, $CDCl_3$) δ 7.66 (d, *J* = 7.6 Hz, 4H), 7.54-7.46 (m, 4H), 7.43-7.38 (m, 2H), 7.26 (s, 2H) ppm; ^{13}C NMR (100 MHz, $CDCl_3$) δ 137.4, 128.73, 128.71, 127.7, 126.5 ppm; HRMS (EI) (*m/z*) [M] $C_{14}H_{12}$ calcd. for 180.0939, found 180.0939. Data are consistent with the literature.[13]

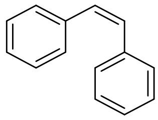

XT141

(*Z*)-1,2-diphenylethene (*Z*-2a)

From the reaction of **1a** with **^{n}BuO-NpMI** as e-PRCat according to **Procedure 11**, for 72 h.

Yield: 25.0 mg, 78%; as a mixture of isomers with *E*-/*Z*- = 1/10; colorless liquid; ^{1}H NMR (400 MHz, $CDCl_3$) δ 7.29-7.18 (m, 10H), 6.62 (s, 2H) ppm; ^{13}C NMR (75 MHz, $CDCl_3$) δ 137.3, 130.3, 128.9, 128.2, 127.1 ppm; HRMS (EI) (*m/z*) [M] $C_{14}H_{12}$ calcd. for 180.0939, found 180.0941. Data are consistent with the literature.[14]

(*Z*)-1-methyl-4-styrylbenzene (*Z*-2b)

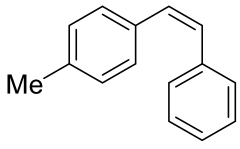

XT505A

According to Procedure **11**. Yield: 28.0 mg, 72%; as a mixture of isomers with *E*-/*Z*- = 1/10; colorless liquid; ^{1}H NMR (400 MHz, $CDCl_3$) (*Z*- isomer) δ 7.30-7.19 (m, 5H), 7.17-7.13 (m, 2H), 7.04 (d, *J* = 8.0 Hz, 2H), 6.57 (s, 2H), 2.32 (s, 3H) ppm; ^{13}C NMR (100 MHz, $CDCl_3$) (*Z*- isomer) δ 137.5, 136.9, 134.3, 130.2, 129.6, 128.92, 128.86, 128.81, 128.2, 127.0, 21.3; HRMS (ESI) (*m/z*) $[M+H]^+$ $C_{15}H_{14}$ calcd. for 194.1096, found 194.1092. Data are consistent with the literature.[15]

(Z)-1-styryl-4-(trifluoromethyl)benzene (*Z*-2c)

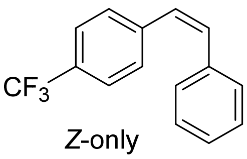

XT525A

According to Procedure **11**. Yield: 39.0 mg, 78%; colorless liquid; ^{1}H NMR (400 MHz, $CDCl_3$) δ 7.50 (d, *J* = 8.0 Hz, 2H), 7.37 (d, *J* = 8.0 Hz, 2H), 7.32-7.20 (m, 5H), 6.76 (d, *J* = 12.4 Hz, 1H), 6.63 (d, *J* = 12.4 Hz, 1H) ppm; ^{13}C NMR (100 MHz, $CDCl_3$) δ 140.9, 136.6, 132.3, 129.2, 129.0 (d, J_{C-P} = 139.6 Hz), 128.97 (d, J_{C-P} = 32.3 Hz), 128.84, 128.76, 128.4, 127.6, 125.2 (q, J_{C-F} = 3.7 Hz), 124.2 (q, J_{C-F} = 270.1 Hz) ppm; ^{31}P NMR (162 MHz, $CDCl_3$) δ 34.4 (0.33P, the minor isomer), 34.0 (1P, the major isomer) ppm; ^{19}F NMR (377 MHz, $CDCl_3$) -63.1 (0.33F, the minor isomer), -63.2 (1F, the minor isomer) ppm; HRMS (EI) (*m/z*) [M] $C_{15}H_{11}F_3$ calcd. for 248.0813, found 248.0810. Data are consistent with the literature.[16]

6,7-dihydro-5*H*-benzo[7]annulene (2d)

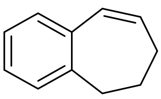

XT354

According to Procedure **11**. Yield: 20.0 mg, 69%; colorless liquid; ^{1}H NMR (400 MHz, $CDCl_3$) δ 7.21-7.05 (m, 4H), 6.42 (d, *J* = 12.4 Hz, 1H), 5.91 (dd, *J* = 12.4, 4.4 Hz, 1H), 2.89-2.83 (m, 2H), 2.48-2.39 (m, 2H), 2.03-1.93 (m, 2H) ppm; ^{13}C NMR (100 MHz, $CDCl_3$) δ 141.7, 136.3, 132.3, 130.9, 129.8, 129.0, 126.6, 125.9, 36.2, 32.5, 27.0 ppm; HRMS (EI) (*m/z*) [M] $C_{11}H_{12}$ calcd. for 144.0939, found 144.0937. Data are consistent with the literature.[17]

1,2-dihydronaphthalene (2e)

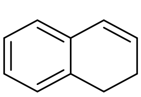

XT234

According to Procedure **11**. Yield: 20.0 mg, 77%; colorless liquid; ^{1}H NMR (400 MHz, $CDCl_3$) δ 7.20-7.09 (m, 3H), 7.06-7.01 (m, 1H), 6.48 (d, *J* = 10.0 Hz, 1H), 6.08-6.01 (m, 1H), 2.82 (t, *J* = 8.0 Hz, 2H), 2.38-2.30 (m, 2H) ppm; ^{13}C NMR (100 MHz, $CDCl_3$) δ 135.5, 134.2, 128.7, 127.8, 127.5, 126.9, 126.5, 125.9, 27.5, 23.2 ppm; HRMS (EI) (*m/z*) [M] $C_{10}H_{10}$ calcd. for 130.0783, found 130.0780. Data are consistent with the literature.[18]

1*H*-indene (2f)

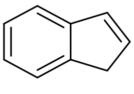

XT355

According to Procedure **11**. Yield: 19.0 mg, 83%; pale yellow liquid; ^{1}H NMR (400 MHz, $CDCl_3$) δ 7.34 (d, *J* = 7.6 Hz, 1H), 7.27 (d, *J* = 7.6 Hz, 1H), 7.16-7.11 (m, 1H), 7.08-7.02 (m, 1H), 6.78-6.72 (m, 1H), 6.42 (dt, *J* = 5.2, 2.0 Hz, 1H), 3.26 (q, *J* = 2.0 Hz, 2H) ppm; ^{13}C NMR (100 MHz, $CDCl_3$) δ 144.9, 143.7,

134.2, 132.1, 126.3, 124.6, 123.7, 121.0, 39.1 ppm; HRMS (EI) (*m/z*) [M] C_9H_8 calcd. for 116.0626, found 116.0624. Data are consistent with the literature.[18]

2,3,4,5-tetrahydro-1,1'-biphenyl (2g)

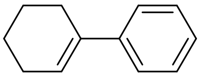

XT134

According to Procedure **11**. Yield: 25.0 mg, 78%; colorless liquid; ^{1}H NMR (300 MHz, $CDCl_3$) δ 7.43-7.37 (m, 2H), 7.35-7.28 (m, 2H), 7.26-7.19 (m, 1H), 6.17-6.09 (m, 1H), 2.47-2.37 (m, 2H), 2.27-2.16 (m, 2H), 1.85-1.75 (m, 2H), 1.73-1.63 (m, 2H) ppm; ^{13}C NMR (75 MHz, $CDCl_3$) δ 142.7, 136.6, 128.2, 126.5, 125.0, 124.8, 27.4, 25.9, 23.1, 22.2 ppm; HRMS (EI) (*m/z*) [M] $C_{12}H_{14}$ calcd. for 158.1096, found 158.1110. Data are consistent with the literature.[19]

(1*S*,4*R*)-2-phenylbicyclo[2.2.1]hept-2-ene (2h)

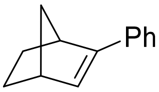

XT485

According to Procedure **11**. Yield: 25.0 mg, 73%; colorless liquid; ^{1}H NMR (400 MHz, $CDCl_3$) δ 7.40-7.34 (m, 2H), 7.29-7.23 (m, 2H), 7.20-7.11 (m, 1H), 6.25 (d, *J* = 3.2 Hz, 1H), 3.30-3.24 (m, 1H), 2.98-2.92 (m, 1H), 1.77-1.71 (m, 2H), 1.50-1.45 (m, 1H), 1.26-1.17 (m, 1H), 1.13-1.08 (m, 2H) ppm; ^{13}C NMR (100 MHz, $CDCl_3$) δ 147.8, 135.8, 129.7, 128.4, 126.7, 124.9, 47.9, 43.4, 43.1, 26.8, 24.8 ppm; HRMS (EI) (*m/z*) [M] $C_{13}H_{14}$ calcd. for 170.1096, found 170.1088. Data are consistent with the literature.[20]

(1-cyclohexylvinyl)benzene (2i)

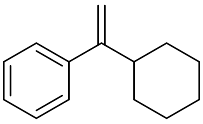

XT465

According to Procedure **11**. Yield: 23.0 mg, 61%; colorless liquid; ^{1}H NMR (400 MHz, $CDCl_3$) δ 7.29-7.16 (m, 5H), 5.09-5.03 (m, 1H), 4.97-4.90 (m, 1H), 2.40-2.30 (m, 1H), 1.82-1.68 (m, 4H), 1.67-1.59 (m, 1H), 1.33-1.19 (m, 2H), 1.18-1.03 (m, 3H) ppm; ^{13}C NMR (100 MHz, $CDCl_3$) δ 155.0, 143.0, 128.1, 127.0, 126.6, 110.3, 42.6, 32.7, 26.8, 26.5 ppm; HRMS (EI) (*m/z*) [M] $C_{14}H_{18}$ calcd. for 186.1409, found 186.1414. Data are consistent with the literature.[21]

4-(prop-1-en-1-yl)-1,1'-biphenyl (2j)

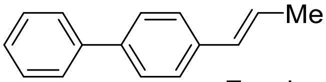

E- only XT297

According to Procedure **11**. Yield: 27.0 mg, 71%; white solid, m.p. 121-123 °C; ^{1}H NMR (400 MHz, $CDCl_3$) δ 7.46-7.42 (m, 2H), 7.40-7.36 (m, 1H), 7.30-7.23 (m, 4H), 7.21-7.15 (m, 1H), 6.29 (d, *J* = 15.6 Hz, 1H), 6.14 (dq, *J* = 15.6, 6.4 Hz, 1H), 1.76 (dd, *J* = 6.4, 1.6 Hz, 3H) ppm; ^{13}C NMR (100 MHz,

CDCl3) δ 140.9, 139.5, 137.0, 130.6, 128.8, 127.20, 127.15, 126.9, 126.2, 125.9, 18.6 ppm; HRMS (EI) (*m/z*) [M] $C_{15}H_{14}$ calcd. for 194.1096, found 194.1089. Data are consistent with the literature.[22]

(*E*)-4-(prop-1-en-1-yl)phenyl benzoate (2k)

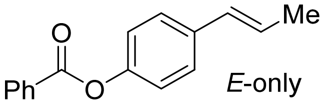

E-only XT261

According to Procedure **11**. Yield: 32.0 mg, 72%; colorless oil; ^{1}H NMR (400 MHz, CDCl3) δ 8.35-8.27 (m, 2H), 7.78-7.71 (m, 1H), 7.66-7.57 (m, 2H), 7.48 (d, *J* = 8.4 Hz, 2H), 7.25 (d, *J* = 8.4 Hz, 2H), 6.57-6.48 (m, 1H), 6.39-6.26 (m, 1H), 2.00 (dd, *J* = 1.6, 6.4 Hz, 3H) ppm; ^{13}C NMR (100 MHz, CDCl3) δ 165.2, 149.7, 135.9, 133.6, 130.2, 130.2, 129.6, 128.6, 126.8, 126.0, 121.7, 18.5 ppm; HRMS (EI) (*m/z*) [M] $C_{16}H_{14}O_2$ calcd. for 238.0994, found 238.1001. Data are consistent with the literature.[23]

(*E*)-1-methoxy-4-(prop-1-en-1-yl)benzene (2l)

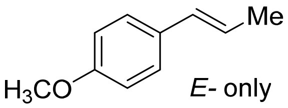

E- only XT350

According to Procedure **11**. Yield: 7.4 mg, 25%; colorless liquid; ^{1}H NMR (400 MHz, CDCl3) δ 7.30-7.24 (m, 2H), 6.88-6.81 (m, 2H), 6.35 (dq, *J* = 15.6, 1.6 Hz, 1H), 6.10 (dq, *J* = 15.6, 6.8 Hz, 1H), 3.81 (s, 3H), 1.87 (dd, *J* = 1.6, 6.8 Hz, 3H) ppm; ^{13}C NMR (100 MHz, CDCl3) δ 158.6, 130.8, 130.4, 126.9, 123.5, 113.9, 55.3, 18.4 ppm; HRMS (EI) (*m/z*) [M] $C_{10}H_{12}O$ calcd. for 148.0888, found 148.0885. Data are consistent with the literature.[24]

1-methoxy-4-vinylbenzene (2m)

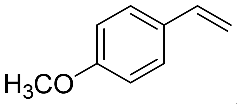

XT370

According to Procedure **11**. Yield: 4.0 mg, 15%; colorless liquid; ^{1}H NMR (400 MHz, CDCl3) δ 7.39-7.31 (m, 2H), 6.91-6.83 (m, 2H), 6.67 (dd, *J* = 10.8, 17.6 Hz, 1H), 5.61 (dd, *J* = 0.8, 17.6 Hz, 1H), 5.13 (dd, *J* = 0.8, 10.8 Hz, 1H), 3.81 (s, 3H) ppm; ^{13}C NMR (100 MHz, CDCl3) δ 159.4, 136.2, 130.5, 127.4, 113.9, 111.6, 55.3 ppm; HRMS (EI) (*m/z*) [M] $C_9H_{10}O$ calcd. for 134.0732, found 134.0727. Data are consistent with the literature.[25]

(*E*)-1-(prop-1-en-1-yl)-3-(trifluoromethyl)benzene (2n)

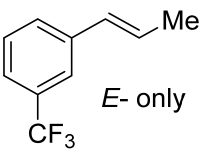

E- only

XT353

According to Procedure **11**. Yield: 29.0 mg, 78%; colorless liquid; ^{1}H NMR (300 MHz, CDCl3) δ 7.56 (s, 1H), 7.53-7.35 (m, 3H), 6.43 (d, *J* = 22.4 Hz, 1H), 6.31 (dq, *J* = 22.8, 8.0 Hz, 1H), 1.90 (dd, *J* = 1.2, 6.3

Hz, 3H) ppm; ^{13}C NMR (75 MHz, $CDCl_3$) δ 138.7, 129.8, 129.0, 128.9, 127.9, 126.0, 124.7 (q, $J_{C\text{-}F}$ = 270.9 Hz), 123.3 (q, $J_{C\text{-}F}$ = 15.6 Hz), 122.5 (q, $J_{C\text{-}F}$ = 15.3 Hz), 118.9, 118.2, 18.5 ppm; ^{19}F NMR (282 MHz, $CDCl_3$) δ -63.3 ppm; HRMS (EI) (*m/z*) [M] $C_{10}H_9F_3$ calcd. for 186.0656, found 186.0661. Data are consistent with the literature.[26]

(*E*)-1-(prop-1-en-1-yl)-3-chlorobenzene (2o)

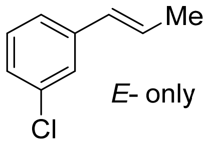

XT349

According to Procedure **11**. Yield: 17.0 mg, 56%; colorless liquid; ^1H NMR (400 MHz, $CDCl_3$) δ 7.32-7.29 (m, 1H), 7.24-7.13 (m, 3H), 6.34 (dq, *J* = 15.6, 1.2 Hz, 1H), 6.25 (dq, *J* = 15.6, 6.0 Hz, 1H), 1.88 (dd, *J* = 6.4, 1.2 Hz, 3H) ppm; ^{13}C NMR (100 MHz, $CDCl_3$) δ 139.8, 134.4, 129.8, 129.7, 127.4, 126.7, 125.8, 124.0, 18.5 ppm; HRMS (EI) (*m/z*) [M] C_9H_9Cl calcd. for 152.0393, found 152.0393. Data are consistent with the literature.[27]

(*E*)-1-chloro-4-(prop-1-en-1-yl)benzene (2p)

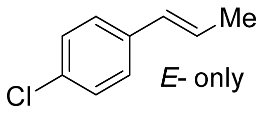

XT357

According to Procedure **11**. Yield: 21.0 mg, 69%; colorless liquid; ^1H NMR (400 MHz, $CDCl_3$) δ 7.28-7.23 (m, 4H), 6.37 (dq, *J* = 15.6, 1.6 Hz, 1H), 6.23 (dq, *J* = 15.6, 6.4 Hz, 1H), 1.89 (dd, *J* = 1.6, 6.4 Hz, 3H) ppm; ^{13}C NMR (100 MHz, $CDCl_3$) δ 136.4, 132.3, 129.9, 128.6, 127.0, 126.5, 18.5 ppm; HRMS (EI) (*m/z*) [M] C_9H_9Cl calcd. for 152.0393, found 152.0393. Data are consistent with the literature.[27]

(*Z*)-1-chloro-4-styrylbenzene (2q)

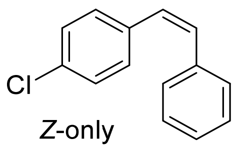

XT358

According to Procedure **11**. Yield: 24.0 mg, 57%; colorless liquid; ^1H NMR (300 MHz, $CDCl_3$) δ 7.25-7.20 (m, 5H), 7.20-7.13 (m, 4H), 6.63 (d, *J* = 12.3 Hz, 1H), 6.53 (d, *J* = 12.0 Hz, 1H) ppm; ^{13}C NMR (75 MHz, $CDCl_3$) δ 136.9, 135.7, 132.8, 131.0, 130.2, 129.0, 128.8, 128.4, 128.4, 127.4 ppm; HRMS (EI) (*m/z*) [M] $C_{14}H_{11}{}^{35}Cl$ calcd. for 214.0549, found 214.0551. Data are consistent with the literature.[28]

1-bromo-4-(prop-1-en-1-yl)benzene (2r)

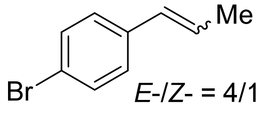

XT493

According to Procedure **11**. Yield: 15.0 mg, 39%; mixture of two isomers, *E*-/*Z*- = 4/1; colorless liquid; ^{1}H NMR (300 MHz, $CDCl_3$) δ 7.47-7.43 (m, 0.4H, the *Z*- isomer), 7.42-7.37 (m, 1.6H, the *E*- isomer), 7.22-7.13 (m, 2H), 6.39-6.30 (m, 1H), 6.23 (dq, *J* = 18.6, 5.6 Hz ppm, 0.8H, the E isomer), 5.82 (dq, *J* = 11.7, 7.2 Hz, 0.2H, the Z isomer), 1.87 (dd, *J* = 6.3, 1.2 Hz, 3H) ppm; ^{13}C NMR (100 MHz, $CDCl_3$) (the E isomer) δ 136.9, 131.5, 129.9, 127.4, 126.6, 120.3, 18.5 ppm; HRMS (EI) (*m/z*) [M] $C_9H_9{}^{79}Br$ calcd. for 195.9888, found 195.9887. Data are consistent with the literature.[29]

4-vinylphenyl diphenylphosphinate (2s)

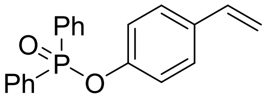

XT514

According to Procedure **11**. Yield: 38.0 mg, 60%; white solid, m.p. 86-88 °C; ^{1}H NMR (400 MHz, $CDCl_3$) δ 7.93-7.86 (m, 4H), 7.55-7.49 (m, 2H), 7.47-7.42 (m, 4H), 7.29-7.23 (m, 2H), 7.19-7.13 (m, 2H), 6.60 (dd, *J* = 17.6, 11.2 Hz, 1H), 5.61 (d, *J* = 17.6 Hz, 1H), 5.16 (d, *J* = 11.2 Hz, 1H) ppm; ^{13}C NMR (100 MHz, $CDCl_3$) δ 150.5 (d, $J_{C\text{-}P}$ = 8.3 Hz), 135.8, 134.1 (d, $J_{C\text{-}P}$ = 1.0 Hz), 132.5 (d, $J_{C\text{-}P}$ = 2.8 Hz), 131.8 (d, $J_{C\text{-}P}$ = 10.3 Hz), 130.9 (d, $J_{C\text{-}P}$ = 137.3 Hz), 128.6 (d, $J_{C\text{-}P}$ = 13.4 Hz), 127.4, 120.8 (d, $J_{C\text{-}P}$ = 4.8 Hz), 113.6 ppm ^{31}P NMR (162 MHz, $CDCl_3$) δ ppm; HRMS (ESI) (*m/z*) $[M+H]^+$ $C_{20}H_{18}O_2P$ calcd. for 321.1039, found 321.1040.

pent-1-en-1-ylbenzene (2t)

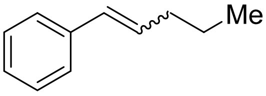

XT215

According to **Procedure 11**. Yield: 20.0 mg, 68%; as a mixture of isomers, *E*-/*Z*- = 10:1; colorless liquid; ^{1}H NMR (300 MHz, $CDCl_3$) δ 7.36-7.26 (m, 4H), 7.23-7.16 (m, 1H), 6.43 (d, *J* = 11.7 Hz, 0.09H, the *Z*- isomer), 6.39 (d, *J* = 15.9 Hz, 0.91H, the *E*- isomer), 6.23 (dt, *J* = 15.9, 6.9 Hz, 0.92H, the *E*- isomer), 5.68 (dt, *J* = 11.7, 7.2 Hz, 0.09H, the *Z*- isomer), 2.37-2.27 (m, 0.20H, the *Z*- isomer), 2.24-2.15 (m, 1.88H, the *E*- isomer), 1.58-1.43 (m, 2H), 0.99-0.93 (m, 3H) ppm; ^{13}C NMR (75 MHz, $CDCl_3$) δ 138.0, 131.0, 129.9, 128.5, 126.8, 125.9, 35.2, 22.6, 13.8 ppm; HRMS (EI) (*m/z*) [M] $C_{11}H_{14}$ calcd. for 146.1096, found 146.1089. Data are consistent with the literature.[30]

(*E*)-dodec-1-en-1-ylbenzene (2u)

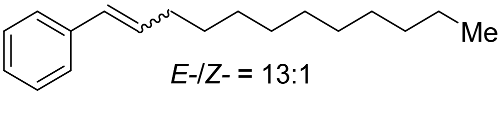

XT468

According to **Procedure 11**. Yield: 39.0 mg, 79%; *E*-/*Z*- =13:1; colorless liquid; ^{1}H NMR (400 MHz, $CDCl_3$) δ 7.35-7.29 (m, 2H), 7.29-7.23 (m, 2H), 7.18-7.13 (m, 1H), 6.38 (d, *J* = 11.6 Hz, 0.07H, the *Z*- isomer), 6.35 (d, *J* = 16.0 Hz, 0.93H, the *E*- isomer), 6.20 (dt, d, *J* = 15.6, 6.8 Hz, 0.93H, the *E*- isomer), 5.64 (dt, *J* = 11.6, 5.8 Hz, 0.07H), 2.33-2.27 (m, 0.15H, the *Z*- isomer), 2.21-2.14 (m, 1.90H, the *E*- isomer), 1.47-1.38 (m, 2H), 1.31-1.21 (m, 14H), 0.86 (t, *J* = 6.4 Hz, 3H) ppm; ^{13}C NMR (100

MHz, $CDCl_3$) δ (peaks for the *E*- isomer only) 138.0, 131.3, 129.7, 128.5, 126.8, 125.9, 33.1, 32.0, 29.7 (one carbon is overlapping), 29.6, 29.4, 29.4, 29.3, 22.7, 14.2 ppm; HRMS (EI) (*m/z*) [M] $C_{18}H_{28}$ calcd. for 244.2191, found 244.2186. Data are consistent with the literature.[31]

prop-1-ene-1,3-diyldibenzene (2v)

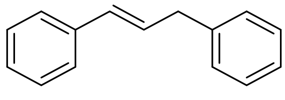

E-/Z- = 20/1 XT362

According to **Procedure 11**. Yield: 24.0 mg, 62%; *E-/Z-* = 20/1; colorless liquid; ^{1}H NMR (300 MHz, $CDCl_3$) δ 7.41-7.19 (m, 10H), 6.62 (dt, *J* = 7.6, 2.0 Hz, 0.05H, the *Z*- isomer), 6.49 (d, *J* = 15.9 Hz, 0.95H, the *E*- isomer), 6.38 (dt, *J* = 15.9, 6.3 Hz, 0.95H, the *E*- isomer), 5.89 (dt, *J* = 11.7, 6.0 Hz, 0.04H, the *Z*- isomer), 3.71 (d, *J* = 7.5 Hz, 0.09H), 3.58 (d, *J* = 6.3 Hz, 1.91H) ppm; ^{13}C NMR (75 MHz, $CDCl_3$) δ (*E*- isomer only) 140.2, 137.5, 131.1, 129.3, 128.7, 128.6 (one carbon is overlapping), 127.2, 126.23, 126.17 (2C), 39.4 ppm; HRMS (EI) (*m/z*) [M] $C_{15}H_{14}$ calcd. for 194.1096, found 194.1085. Data are consistent with the literature.[32]

(cyclobutylidenemethyl)benzene (2w)

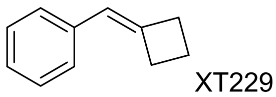

XT229

According to **Procedure 11**. Yield: 24.0 mg, 83%; colorless liquid; ^{1}H NMR (300 MHz, $CDCl_3$) δ 7.36-7.28 (m, 2H), 7.25-7.20 (m, 2H), 7.20-7.13 (m, 1H), 6.13-6.07 (m, 1H), 3.12-3.03 (m, 2H), 2.96-2.87 (m, 2H), 2.20-2.07 (m, 2H) ppm; ^{13}C NMR (75 MHz, $CDCl_3$) δ 144.9, 138.1, 128.4, 127.1, 125.8, 120.9, 32.8, 32.7, 18.4 ppm; HRMS (EI) (*m/z*) [M] $C_{11}H_{12}$ calcd. for 144.0939, found 144.0933. Data are consistent with the literature.[33]

(cyclopentylidenemethyl)benzene (2x)

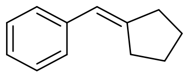

XT253

According to **Procedure 11**. Yield: 27.0 mg, 87%; colorless liquid; ^{1}H NMR (300 MHz, $CDCl_3$) δ 7.27-7.20 (m, 4H), 7.13-7.04 (m, 1H), 6.31-6.25 (m, 1H), 2.52-2.38 (m, 4H), 1.80-1.53 (m, 4H) ppm; ^{13}C NMR (75 MHz, $CDCl_3$) δ 147.3, 138.9, 128.2 (2C), 128.0 (2C), 125.6, 120.8, 36.0, 31.2, 27.3, 25.7 ppm; HRMS (EI) (*m/z*) [M] $C_{12}H_{14}$ calcd. for 158.1096, found 158.1090. Data are consistent with the literature.[34]

2-vinylnaphthalene (2y)

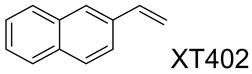

XT402

According to **Procedure 11**. Yield: 19.0 mg, 62%; white solid, m.p. 64-66 °C; ^{1}H NMR (400 MHz, $CDCl_3$) δ 7.87-7.78 (m, 3H), 7.76 (s, 1H), 7.65 (dd, *J* = 1.6, 8.8 Hz, 1H), 7.55-7.41(m, 2H), 6.90 (dd, *J*

= 10.8, 17.6 Hz, 1H), 5.89 (d, J = 17.6 Hz, 1H), 5.35 (d, J = 10.8 Hz, 1H) ppm; ^{13}C NMR (100 MHz, $CDCl_3$) δ 137.0, 135.0, 133.6, 133.2, 128.2, 128.1, 127.7, 126.4, 126.3, 125.9, 123.2, 114.2 ppm; HRMS (EI) (*m/z*) [M] $C_{12}H_{10}$ calcd. for 154.0783, found 154.0785. Data are consistent with the literature.[35]

7-vinyl-2*H*-benzo[*b*][1,4]oxazin-3(4*H*)-one (2z)

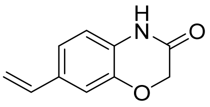

XT439

According to **Procedure 11**. Yield: 17.0 mg, 49%; pale yellow solid, m.p. 187-188 °C; ^{1}H NMR (400 MHz, $CDCl_3$) δ 9.20 (s, 1H), 7.03 (dd, J = 2.0, 8.4 Hz, 1H), 6.96-6.86 (m, 2H), 6.62 (dd, J = 10.8, 17.6 Hz, 1H), 5.64 (d, J = 17.6 Hz, 1H), 5.20 (d, J = 11.2 Hz, 1H), 4.63 (s, 2H) ppm; ^{13}C NMR (100 MHz, $CDCl_3$) δ 166.2, 143.3, 135.5, 132.8, 126.1, 122.4, 116.8, 113.6, 113.4, 67.3 ppm; HRMS (ESI) (*m/z*) $[M+H]^+$ $C_{10}H_{10}NO_2$ calcd. for 176.0633, found 175.0631.

(*S*)-4-(prop-1-en-2-yl)-1-vinylcyclohex-1-ene (2ab)

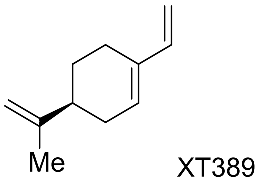

XT389

According to **Procedure 11**. Yield: 10.0 mg, 33%; colorless liquid; ^{1}H NMR (300 MHz, $CDCl_3$) δ 6.37 (dd, J = 10.8, 17.7 Hz, 1H), 5.81-5.74 (m, 1H), 5.07 (d, J = 17.4 Hz, 1H), 4.92 (d, J = 10.8 Hz, 1H), 4.76-4.70 (m, 2H), 2.39-2.00 (m, 5H), 1.97-1.85 (m, 1H), 1.78-1.71 (m, 3H), 1.57-1.47 (m, 1H) ppm; ^{13}C NMR (75 MHz, $CDCl_3$) δ 149.8, 139.7, 135.8, 129.2, 110.1, 108.7, 41.2, 31.2, 27.3, 24.3, 20.8 ppm; HRMS (EI) (*m/z*) [M] $C_{11}H_{16}$ calcd. for 148.1252, found 148.1251. Data are consistent with the literature.[36]

(1*R*,5*S*)-6,6-dimethyl-2-vinylbicyclo[3.1.1]hept-2-ene (2ac)

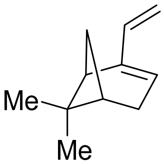

XT432

According to **Procedure 11**. Yield: 9.0 mg, 30%; colorless liquid; ^{1}H NMR (400 MHz, $CDCl_3$) δ 6.37 (dd, J = 10.8, 17.6 Hz, 1H), 5.59-5.53 (m, 1H), 5.05 (d, J = 17.2 Hz, 1H), 4.89 (d, J = 10.8 Hz, 1H), 2.57 (t, J = 5.2 Hz, 1H), 2.46-2.41 (m, 1H), 2.39-2.27 (m, 2H), 2.17-2.09 (m, 1H), 1.33 (s, 3H), 1.14 (d, J = 8.8 Hz, 1H), 0.80 (s, 3H) ppm; ^{13}C NMR (100 MHz, $CDCl_3$) δ 146.8, 137.8, 124.5, 109.6, 41.0, 40.3, 37.69, 3.9, 31.2, 26.4, 20.7 ppm; HRMS (EI) (*m/z*) [M] $C_{11}H_{16}$ calcd. for 148.1252, found 148.1251. Data are consistent with the literature[37]

(1*R*,2*S*,5*R*)-2-isopropyl-5-methylcyclohexyl 4-vinylbenzoate (2ad)

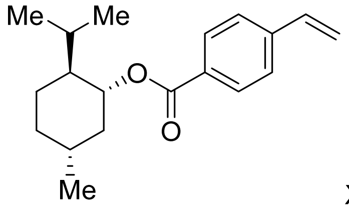

XT472

According to **Procedure 11**. Yield: 26.0 mg, 45%; colorless liquid; ^{1}H NMR (400 MHz, $CDCl_3$) δ 8.06-7.95 (m, 2H), 7.51-7.31 (m, 2H), 6.75 (dd, *J* = 10.8, 17.6 Hz, 1H), 5.86 (d, *J* = 17.6 Hz, 1H), 5.37 (d, *J* = 10.8 Hz, 1H), 4.93 (dt, *J* = 4.4, 10.8 Hz, 1H), 2.17-2.09 (m, 1H), 2.01-1.91 (m, 1H), 1.78-1.68 (m, 2H), 1.62-1.50 (m, 2H), 1.19-1.05 (m, 2H), 1.00-0.94 (m, 1H), 0.94-0.90 (m, 6H), 0.80 (d, *J* = 6.8 Hz, 3H) ppm; ^{13}C NMR (100 MHz, $CDCl_3$) δ 165.9, 141.8, 136.1, 130.0, 129.9, 126.1, 116.3, 74.8, 47.3, 41.0, 34.4, 31.5, 26.6, 23.7, 22.1, 20.8, 16.6 ppm; HRMS (ESI) (*m/z*) $[M+H]^+$ $C_{19}H_{27}O_2$ calcd. for 287.1933, found 286.1920. Data are consistent with the literature.[38]

(*E*)-3,7-dimethylocta-2,6-dien-1-yl 4-vinylbenzoate (2ae)

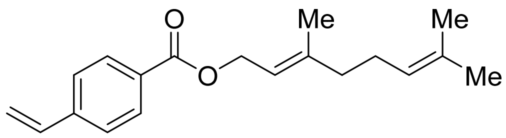

XT473

According to **Procedure 11**. Yield: 30.0 mg, 52%; colorless liquid; ^{1}H NMR (400 MHz, $CDCl_3$) δ 8.04-7.96 (m, 2H), 7.49-7.41 (m, 2H), 6.75 (dd, *J* = 10.8, 17.2 Hz, 1H), 5.85 (d, *J* = 17.2 Hz, 1H), 5.52-5.43 (m, 1H), 5.37 (d, *J* = 10.8 Hz, 1H), 5.15-5.04 (m, 1H), 4.84 (d, *J* = 7.2 Hz, 2H), 2.17-2.03 (m, 4H), 1.77 (s, 3H), 1.68 (s, 3H), 1.60 (s, 3H) ppm; ^{13}C NMR (100 MHz, $CDCl_3$) δ 166.4, 142.3, 141.8, 136.1, 131.9, 129.9, 129.7, 126.1, 123.8, 118.5, 116.4, 61.9, 39.6, 26.3, 25.7, 17.7, 16.6 ppm; HRMS (ESI) (*m/z*) $[M+H]^+$ $C_{19}H_{25}O_2$ calcd. for 285.1849, found 285.1847.

(4*R*,4a*S*,6*R*)-4,4a-dimethyl-6-(prop-1-en-2-yl)-2,3,4,4a,5,6,7,8-octahydronaphthalen-2-yl 4-vinylbenzoate (2af)

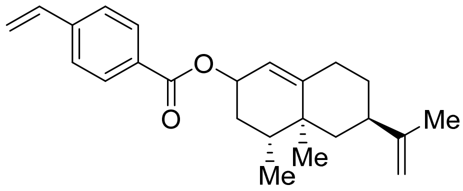

XT477

According to **Procedure 11**. Yield: 26.0 mg, 37%; colorless liquid; ^{1}H NMR (400 MHz, $CDCl_3$) δ 8.06-7.96 (m, 2H), 7.50-7.41 (m, 2H), 6.75 (dd, *J* = 10.8, 17.6 Hz, 1H), 5.86 (d, *J* = 17.6 Hz, 1H), 5.61-5.54 (m, 1H), 5.42-5.35 (m, 2H), 4.74-4.66 (m, 2H), 2.41-2.32 (m, 1H), 2.30-2.21 (m, 1H), 2.20-2.13 (m, 1H), 1.95-1.80 (m, 3H), 1.73 (s, 3H), 1.72-1.56 (m, 3H), 1.29-1.22 (m, 1H), 1.06 (s, 3H), 0.94 (d, *J* = 6.0 Hz, 1H) ppm; ^{13}C NMR (100 MHz, $CDCl_3$) δ 166.3, 150.1, 147.9, 141.8, 136.1, 129.9 (one carbon is overlapping), 126.0, 120.2, 116.3, 108.7, 71.6, 44.51, 40.8, 39.2, 38.2, 32.8, 32.7, 32.4, 20.8, 18.2, 15.4 ppm; HRMS (ESI) (*m/z*) $[M+Na]^+$ $C_{24}H_{30}NaO_2$ calcd. for 373.2138, found 373.2140.

(3S,8S,9S,10R,13R,14S,17R)-10,13-dimethyl-17-((R)-6-methylheptan-2-yl)-2,3,4,7,8,9,10,11,12, 13,14,15,16,17-tetradecahydro-1H-cyclopenta[a]phenanthren-3-yl 4-vinylbenzoate (2ag)

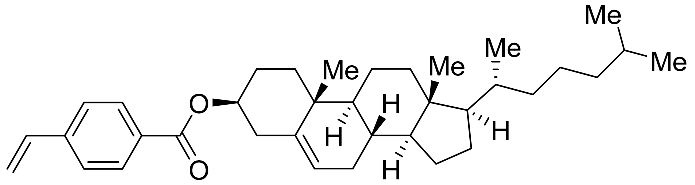

XT471

Yield: 41.0 mg, 40%; white solid, m.p. 179-181 °C; ^{1}H NMR (400 MHz, $CDCl_3$) δ 8.00 (d, *J* = 8.0 Hz, 2H), 7.45 (d, *J* = 8.4 Hz, 2H), 6.75 (dd, *J* = 10.8, 17.6 Hz, 1H), 5.86 (d, *J* = 17.6 Hz, 1H), 5.42 (d, *J* = 4.0 Hz, 1H), 5.37 (d, *J* = 11.2 Hz, 1H), 4.92-4.78 (m, 1H), 2.47 (d, *J* = 7.6 Hz, 2H), 2.07-1.95 (m, 3H), 1.95-1.88 (m, 1H), 1.87-1.77 (m, 1H), 1.77-1.66 (m, 1H), 1.64-1.45 (m, 6H), 1.42-1.31 (m, 3H), 1.31-1.08 (m, 8H), 1.07 (s, 3H), 1.06-0.95 (m, 4H), 0.93 (d, *J* = 6.4 Hz, 3H), 0.88 (dd, *J* = 2.0, 6.8 Hz, 6H), 0.69 (s, 3H) ppm; ^{13}C NMR (100 MHz, $CDCl_3$) δ 165.8, 141.8, 139.7, 136.1, 130.0, 129.9, 126.0, 122.8, 116.3, 74.6, 56.7, 56.2, 50.1, 42.4, 39.8, 39.6, 38.3, 37.1, 36.7, 36.2, 35.8, 32.0, 31.9, 28.3, 28.1, 27.9, 24.3, 23.9, 22.9, 22.6, 21.1, 19.4, 18.8, 11.9 ppm; HRMS (ESI) (*m/z*) $[M]^+$ $C_{36}H_{52}O_2$ calcd. for 516.3967, found 516.3967. Data are consistent with the literature.[39]

1-(2-chloro-2-phenylvinyl)-4-(trifluoromethyl)benzene (2ah)

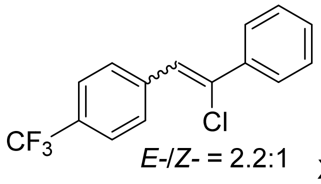

XT522A+B

According to **Procedure 11**. Yield: 39 mg, 69%; as a mixture of isomers, *E*-/*Z*- = 2.2:1; ^{1}H NMR (400 MHz, $CDCl_3$) δ 7.89 (d, *J* = 8.4 Hz, 0.63H, the *Z*- isomer), 7.84 (d, *J* = 7.6 Hz, 0.63H, the *Z*- isomer), 7.74 (d, *J* = 8.4 Hz, 0.66H, the *Z*- isomer), 7.64 (d, *J* = 8.0 Hz, 1.38H, the *E*- isomer), 7.57 (d, *J* = 8.4 Hz, 1.19H, the *E*- isomer), 7.50 (d, *J* = 7.6 Hz, 0.67H, the E isomer), 7.43 (d, *J* = 7.2 Hz, 0.32H, the *Z*- isomer), 7.29-7.25 (m, 1.19H, the *E*- isomer), 7.25-7.23 (m, 0.61H, the *Z*- isomer), 7.21 (s, 0.31H, the *Z*- isomer), 7.11 (s, 0.60H, the *E*- isomer), 7.07-7.02 (m, 1.22H, the *E*- isomer) ppm; ^{19}F NMR (377 MHz, $CDCl_3$) δ -63.2 (0.46F, the *E*- isomer), -63.3 (1F, the *Z*- isomer) ppm. The isomer below was isolated by careful chromatography for characterization and identity confirmation:

(*Z*)-1-(2-chloro-2-phenylvinyl)-4-(trifluoromethyl)benzene (*Z*-2ah)

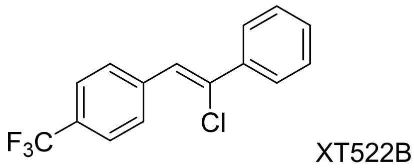

XT522B

According to **Procedure 11**. White solid, m.p. 143-144 °C; ^{1}H NMR (400 MHz, $CDCl_3$) δ 7.94 (d, *J* = 8.0 Hz, 2H), 7.88 (d, *J* = 7.2 Hz, 2H), 7.79 (d, *J* = 8.0 Hz, 2H), 7.58-7.52 (m, 2H), 7.50-7.44 (m, 1H), 7.26 (s, 1H) ppm; ^{13}C NMR (100 MHz, $CDCl_3$) δ 142.7, 134.7, 130.6 (q, $J_{C\text{-}F}$ = 32.4 Hz), 130.5, 129.6,

128.6, 128.4, 128.0, 127.0, 125.5 (q, $J_{C\text{-}F}$ = 3.8 Hz), 124.0 (q, $J_{C\text{-}F}$ = 270.4 Hz); ^{19}F NMR (377 MHz, $CDCl_3$) δ -63.2 ppm; HRMS (ESI) (*m/z*) $[M+H]^+$ $C_{15}H_{11}{}^{35}ClF_3$ calcd. for 283.0496, found 283.0423.

1,2-diphenylethane (2ai)

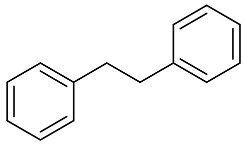

XT320

According to **Procedure 11**. Yield: 19.0 mg, 51%; colorless crystal, m.p. 46 °C; ^{1}H NMR (400 MHz, $CDCl_3$) δ 7.38-7.32 (m, 4H), 7.29-7.23 (m, 6H), 2.99 (s, 4H) ppm; ^{13}C NMR (100 MHz, $CDCl_3$) δ 141.8, 128.5, 128.4, 125.9, 37.8 (2C) ppm; HRMS (ESI) (*m/z*) [M] $C_{14}H_{14}$ calcd. for 182.1096, found 182.1101. Data are consistent with the literature.[40]

(*S*)-1-methyl-4-(prop-1-en-2-yl)cyclohex-1-ene (2aj)

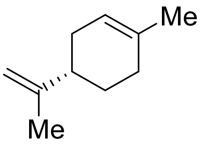

XT338

According to **Procedure 11**. Yield: 12.5 mg, 46%; colorless liquid; ^{1}H NMR (400 MHz, $CDCl_3$) δ 5.46-5.35 (m, 1H), 4.71 (s, 2H), 2.15-2.00 (m, 3H), 2.00-1.87 (m, 2H), 1.84-1.76 (m, 1H), 1.75-1.71 (m, 3H), 1.68-1.62 (m, 3H), 1.54-1.40 (m, 1H) ppm; ^{13}C NMR (100 MHz, $CDCl_3$) δ 150.3, 133.8, 120.7, 108.4, 41.1, 30.8, 30.6, 27.9, 23.5, 20.8 ppm; HRMS (ESI) (*m/z*) [M] $C_{10}H_{16}$ calcd. for 136.1252, found 136.1248. Data are consistent with the literature.[41]

10. Cyclic Voltammetry

Cyclic voltammetry was conducted using a three-electrode setup consisting of a glassy carbon disc working electrode 'WE' (d = 3.0 mm, BASi MF-2012), an Ag/AgCl wire reference electrode 'RE' (containing sat. aq. KCl) and a platinum wire counter-electrode 'CE'. Electrochemical measurements were carried out using an Metrohm Autolab PGSTAT 302N potentiostat at room temperature (298 K). Before use and between measurements, the WE was mechanically cleaned by polishing (Buehler Metadi Diamond polishing 1 Micron) and rinsed with distilled water repeatedly until its surface was reflective by eye, then allowed to air dry. The RE was washed with electrolyte solution and distilled water and stored in 3.0 M aq. KCl when not in use/between measurements. The CE was cleaned by soaking in 2.0 M HCl for 1-2 h, then rinsed with distilled water and allowed to air dry. Ferrocene was recrystallized twice from *n*-hexane prior to use. $^nBu_4N{\cdot}PF_6$ ('TBAP') was used as supplied commercially from TCI (98%+). Unless otherwise stated, all solutions were prepared at 10.0 mM concentration (in 0.1 M TBAP that had been bubbled for 5 min with Ar prior to the analyte being added) and were purged with Ar bubbling for 5 min prior to recording CV. After measuring one CV scan, Ferrocene (2.2 mg, 12 μmol) was added and the mixture purged with Ar bubbling for 5 min prior to recording CV again. Data acquisition and processing were performed with Metrohm Autolab Nova

1.10.4. The given redox potentials were calculated as follows: Potential of sample vs. Ag/AgCl, minus potential of Ferrocene/Ferrocenium couple against Ag/AgCl, plus a correction of +0.38 V for conversion to vs. saturated calomel electrode (vs. SCE). A peak height of 30 µA corresponds to a one-electron reduction event. Cyclic voltammograms of **NpMI** and **^{n}BuO-NpMI** reveal reversible, one-electron reductions at −1.32 V (vs. SCE) for **NpMI** and −1.4 V (vs. SCE) for **^{n}BuO-NpMI** (Figure S3). The second reductions of both catalysts are irreversible at a measured potential of > −2.0 V.

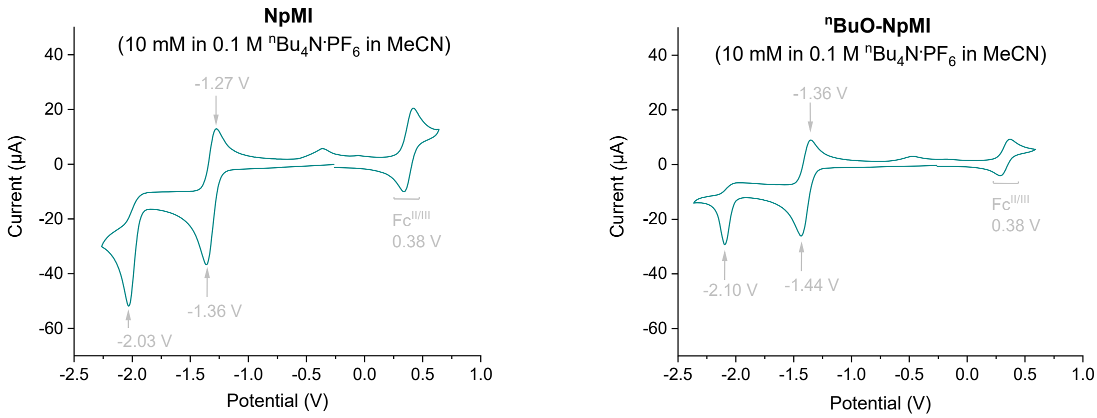

Figure S3. Cyclic voltammograms of **NpMI** and **^{n}BuO-NpMI** in MeCN (0.1 M TBAP) vs. Ferrocene.

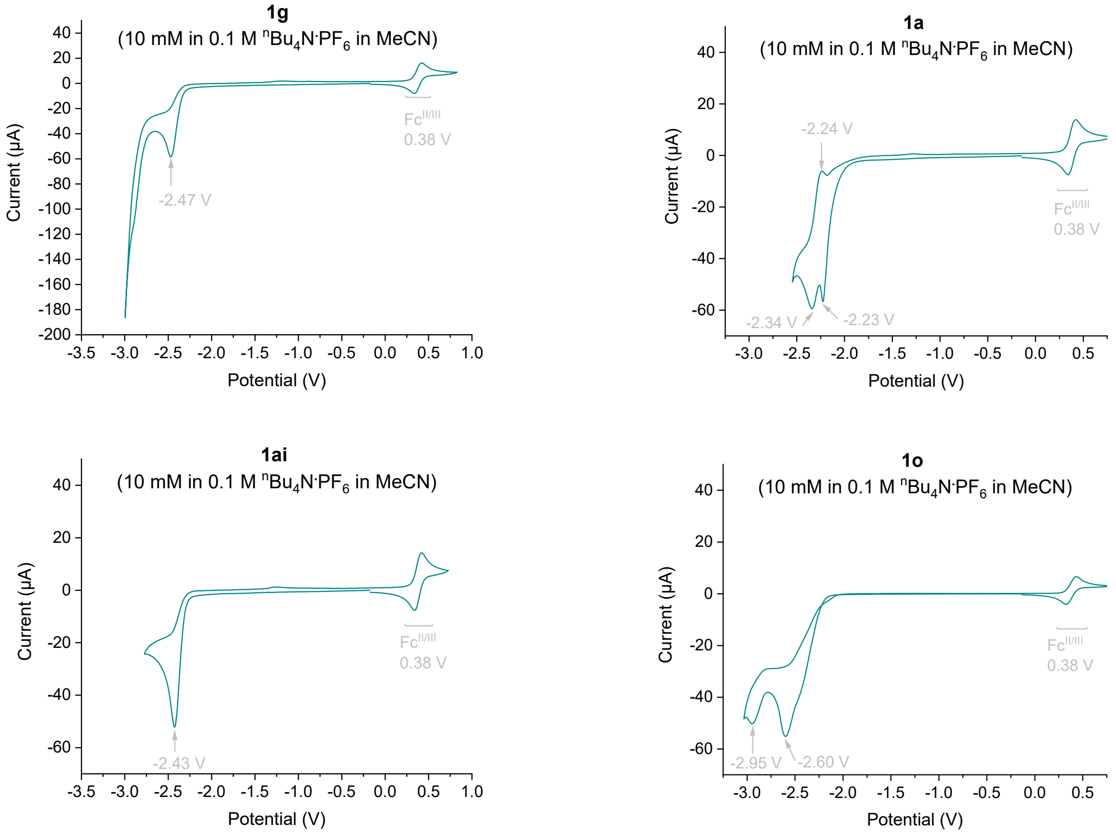

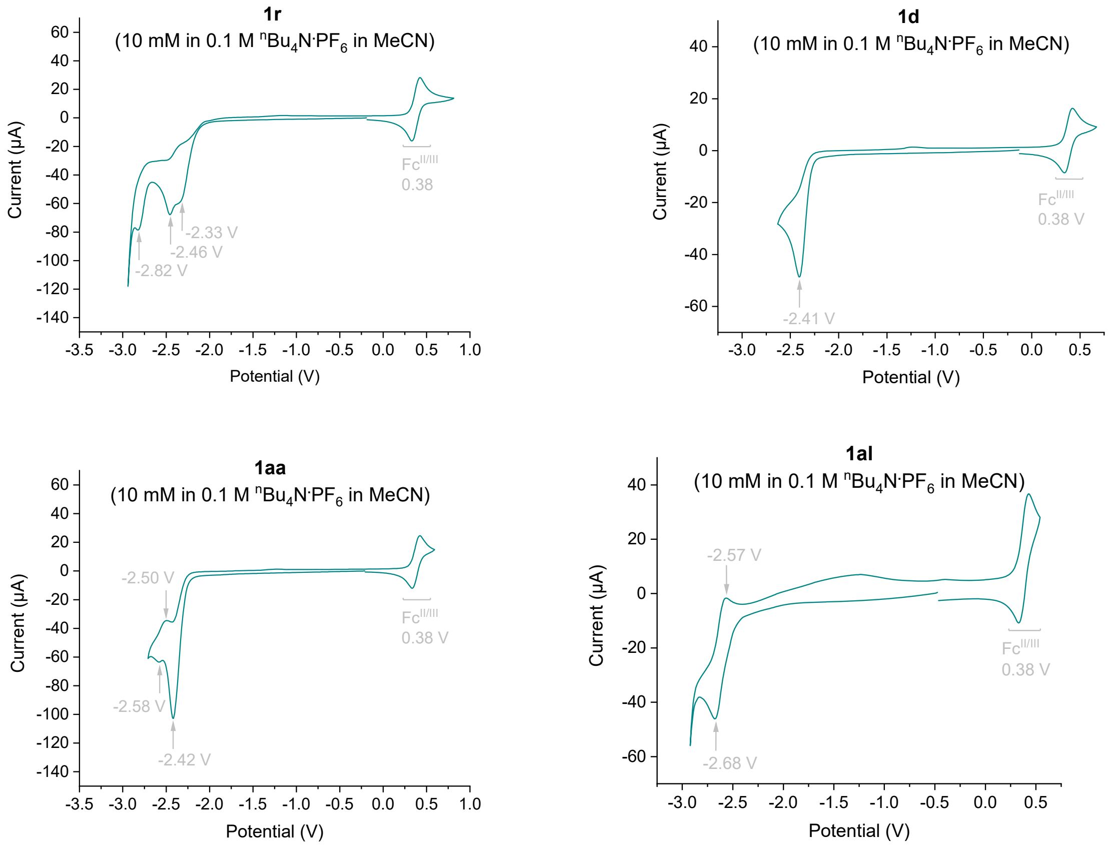

Figure S4. Cyclic voltammograms of phosphinates in MeCN (0.1 M TBAP) vs. Ferrocene.

A peak height of 50 µA reveals a one-electron reduction event. Cyclic voltammograms of **1g**, **1ai** and **1d** with increasingly negative potential up to −3.00 V (vs. SCE) reveal single one-electron irreversible reductions (Figure S4). For **1a**, an irreversible one-electron reduction occurs at −2.23 V, followed by a reversible one-electron reduction at −2.29 V. The former presumably corresponds to irreversible one-electron reductions of the phosphinates, while the latter event may correspond to a reversible reduction of the stilbene product, given a similar value to the literature potential for this reduction (E^{p}_{red} = −3.0 V vs. Ferrocene (0.1 M $^{n}Bu_4N\cdot Br$ in MeCN).[42] The DFT calculated phosphinate redox potential was E^{p}_{red} = −2.60 V vs. SCE (Section S18 for methods).

For **1o**, a superposition of two irreversible one-electron reductions appears to occur at −2.60 V (vs. SCE). Given the calculated phosphinate redox potential (E^{p}_{red} = −2.45 V vs. SCE) from DFT calculations (Section S18 for methods), together with the literature value for chlorobenzene (E^{p}_{red} = −2.78 V vs. SCE),[43] we propose that this peak may derive from the similar E^{p}_{red} values of the phosphinate and aryl chloride moieties. A similar pattern occurs for **1r**, with a superposition of two irreversible one-electron reductions at −2.33 V and −2.46 V. The DFT calculated phosphinate redox potential is E^{p}_{red} = −2.44 V vs. SCE from DFT calculations (Section S18 for methods). The literature value for bromobenzene (E^{p}_{red} = −2.44 V vs. SCE),[43] Given that the redox potentials of all other phosphinates measured herein (except **1a**) exceed −2.40 V, and that the peak potential of aryl chloride **1o** was 0.18 V more positive than the literature potential of chlorobenzene, we presume the

first reduction peak corresponds to the aryl bromide (0.11 V more positive than the literature potential of bromobenzene).

For **1o** and **1r**, at more negative potentials a third irreversible one-electron reduction occurs in each case at −2.95 V and −2.82 V, respectively. These peaks may derive from reduction of the halostyrene products, given that the styrenes require more negative potentials.[44]

For **1aa**, an irreversible two-electron reduction occurs at −2.42 V, followed by a reversible one-electron reduction at −2.54 V (vs. SCE) respectively. The latter reversible peak cannot be rationalized at this stage. Substrate **1al** exhibits a quasi-reversible one-electron reduction at −2.63 V (vs. SCE).

11. Direct Electrolysis Experiments

To confirm the selectivity benefit of e-PRC, we subjected substrates **1p** and **1r** to direct electrolysis at −3.0 V in the absence of catalyst and light and at 60 °C (Scheme 1). The relevance for higher temperature comes from a previous report already showed that no reaction occurred at similar applied constant potentials at rt, and that 60-110 °C was required for C(sp^3)-O cleavage.[11] In both cases, full conversion occurred to a complex mixture of products. No olefination products were observed.

The detrimental effect of high applied constant potentials in e-PRC reactions can also be found in the main manuscript, Table 1, entry 1.

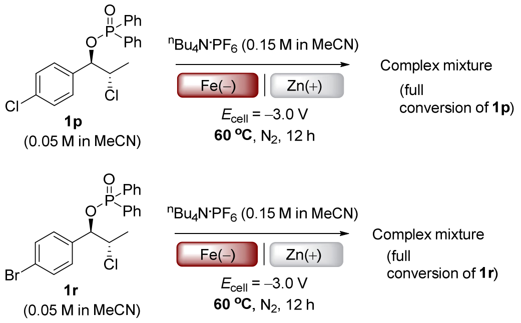

Scheme S1. Direct electrolysis experiments at room temperature and 60 °C.

12. Spectroelectrochemistry of Catalysts

Chrono amperometry was measured in an Ottle Cell (optically transparent thin-layer electrochemical cell) with Pt minigrid as working and counter electrode, an Ag wire as pseudo reference electrode and a pathlength of 0.02 cm.

For further details of Ottle Cell, see: https://research.reading.ac.uk/spectroelectrochemistry/optically-transparent-thin-layer-electrochemical-cells/room-temperature-ottle-cell/

Samples were prepared as 1.5 mM or 2.5 mM solutions of **NpMI** and **ⁿBuO-NpMI**, respectively, in MeCN (0.1 M $Bu_4N{\cdot}PF_6$). After degassing by bubbling the sample with Ar for 5 min, an increasingly negative potential from 0 to −1.6 V was applied and consecutive UV-vis absorption spectra were recorded at 20 s intervals via the Agilent 8453 spectrometer. The UV-vis spectra show, for both **NpMI** and **ⁿBuO-NpMI** (Figure S5), a decrease in the 330 - 350 nm band intensity accompanied by an increase in new bands at 270 nm, 415 nm, a broad band between 430-530 nm (with a peak at 490 nm) and a broad band between 600 - 900 nm (with peaks at 745 nm and 830 nm).

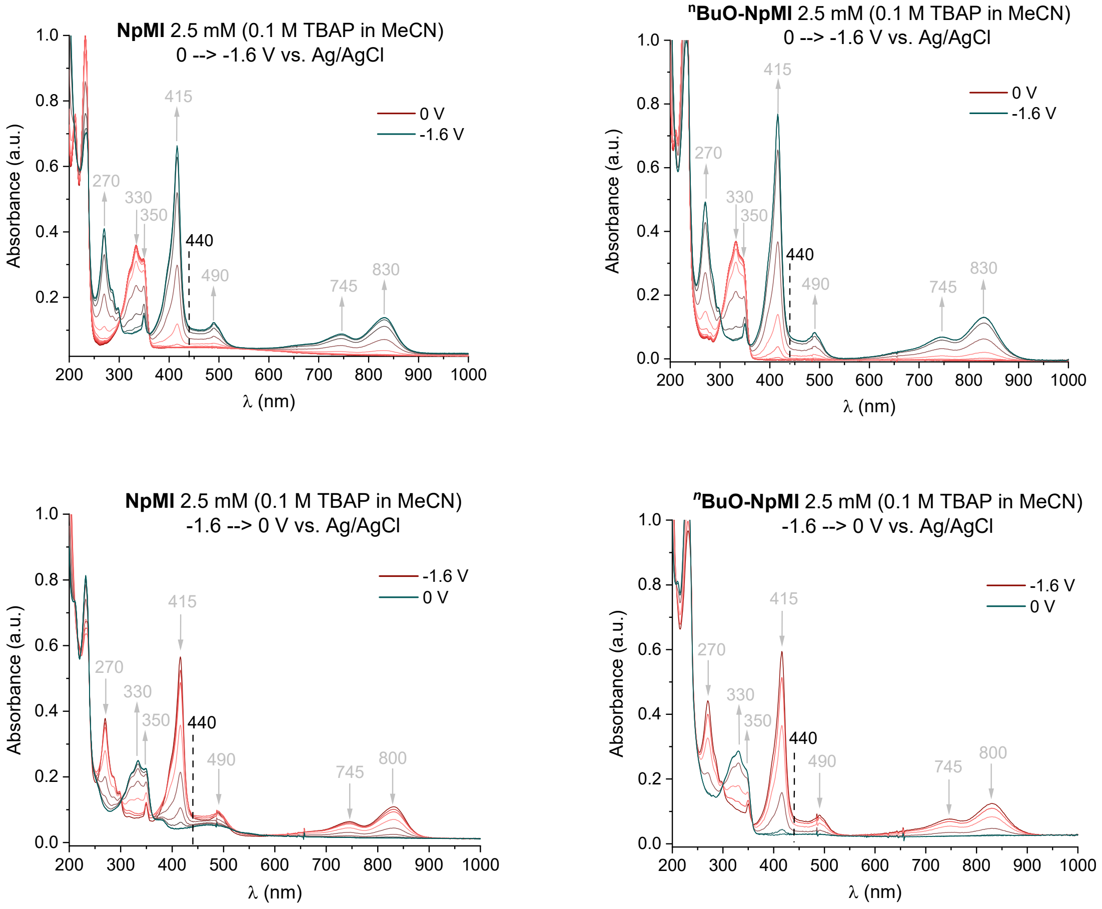

Figure S5. Consecutive UV-vis spectra (from red to teal) of **NpMI** (top, left) and **ⁿBuO-NpMI** (top right) from chrono amperometry.

13. UV-vis Spectroscopy of Electrogenerated Radical Anions

The reductions of **NpMI** and **ⁿBuO-NpMI** (2.5 mM in 0.1 M $Bu_4N{\cdot}PF_6$) was performed accordingly to **Procedure 11** without a phosphinate substrate. A green color was observed in the cathodic half-chamber of the divided cell. After 1 h, the contents of the cathodic chamber were taken for UV-vis analysis.

For samples in the presence of **1d**, the solid substrate was measured into a vial and the total cathodic chamber mixture was added to achieve the concentration of the preparative reactions. Then, 25.0 µL

of this mixture were added to a quartz cuvette which was made up to 2.0 mL total volume with anhydrous, degassed MeCN.

Samples was then covered in aluminum foil to prevent penetration of ambient light. Samples that had been stored in the dark were analyzed by UV-vis. Then, samples were irradiated with blue light by a 440 nm single spot LED perpendicular to the beam path of the spectrometer.

The UV-vis spectra of electrochemically reduced **NpMI** and **^{n}BuO-NpMI** show (Figure S6) the appearance of new bands for their radical anions with λ_{max} values (415 nm, 490 nm, 745 nm and 830 nm) in perfect agreement with their spectroelectrochemical analysis (Section S12). The radical anions are stable in the presence of **1d** in the dark (bordeaux line vs. black line are superimposed). Irradiation of the radical anions in the presence of **1d** leads to a clear decrease in the aforementioned peaks of the radical anion and regeneration of the neutral species **NpMI** and **^{n}BuO-NpMI** (333 nm and 350 nm bands). This confirms that SET is successful from both photoexcited radical anions to **1d**. No difference could be observed between the UV-vis spectra of **^{n}BuO-NpMI$^{\bullet-}$** vs. **^{n}BuO-NpMI$^{\bullet-}$** + **1d** (Figure S7).

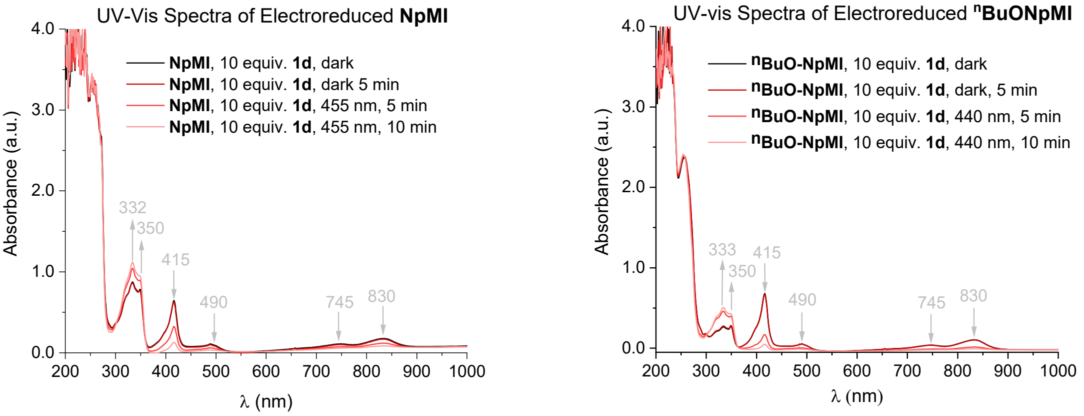

Figure S6. UV-vis spectra of electroreduced e-PRCats in the presence of substrate **1d**, in the dark and after 440 nm irradiation of samples.

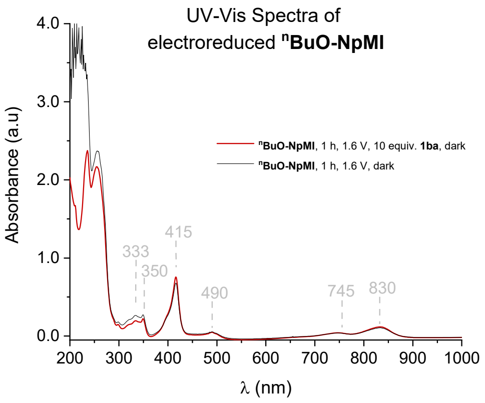

Figure S7. UV-vis spectra of electroreduced **^{n}BuO-NpMI** in the presence of substrate **1d**.

Online UV-Vis measurements were performed on an Agilent 8453 spectrometer using an ISMATEC ISM930C dosing pump to continuously pump the catholyte (1 mL/min) through a quartz flow cell (pathlength 1.0 cm). The concentration of **^{n}BuO-NpMI** was decreased to one sixth of the conditions in **Procedure 11**, spectra were recorded at 10 min intervals. Monitoring the olefination reaction of **1d** under the preparative conditions via UV-vis spectroscopy over a timescale of 100 min, the 330 nm and 350 nm bands of the neutral **^{n}BuO-NpMI** were increasing. The radical anion was difficult to observe at all. Since the radical anion is highly reactive under irradation in the presence of **1d**, no buildup can be observed by UV-Vis spectroscopy. Instead we observed a new feature arising between 450-600 nm (λ_{max} = 510, 545 nm), which might originate from oxidative processes (see Figure S8).

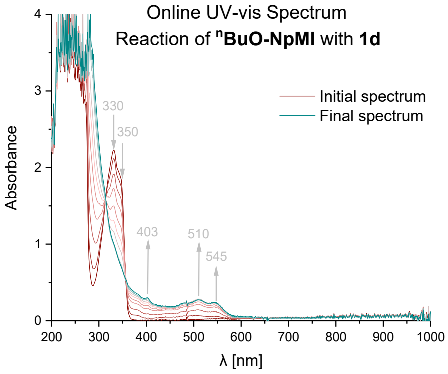

Figure S8. Consecutively recorded online UV-vis spectra of the olefination of **1d** by **^{n}BuO-NpMI**.

14. LUMINESCENCE SPECTROSCOPY OF CATALYSTS AND ELECTROREDUCED CATALYSTS

14.1. Steady-state Luminescence Emission Spectroscopy

Steady-state luminescence measurements were performed on a Horiba® Scientific FluoroMax-4 instrument, which comprised an USHIO S150MO xenon short arc lamp as an excitation source, 200 - 900 nm double grating excitation and emission monochromators and a R928 Hamamatsu photomultiplier tube. The integration time was set to 0.1 s and the slid widths were 3 nm for both excitation and emission. For neutral e-PRCats, the excitation monochromator was set to 352 nm and emission was set to measure 360 - 600 nm. For electroreduced e-PRCats, the excitation monochromator was set to 440 nm and emission was set to measure 460 - 800 nm. A signal detector (S1) was enabled to measure the intensity signal (counts s^{-1}) and a reference detector (R1) was enabled to compensate for light source fluctuations (μA). Therefore, 'Counts' refers to S1/R1 with the unit s^{-1} / μA but is given arbitrary units (a.u.) for simplicity.

FluoroEssence V3.9 software was used for data acquisition and processing. Sample preparation follows that described in Section S13 for UV-vis spectroscopy. The excitation spectra of neutral and

electroreduced e-PRCats (**NpMI** and **^{n}BuO-NpMI**), determined by scanning excitation wavelength at the peak emission wavelength, are reported in this section as overlays with their steady-state emission spectra (Figure S8-S9).

***NOTE –** In this section, the labels **NpMI**$^{\bullet-}$ and **^{n}BuO-NpMI**$^{\bullet-}$ are used which refer to the *samples*' spectral data measured after electroreduction of **NpMI** and **^{n}BuO-NpMI**. We do not claim to assign excitation and emission spectra to doublet states. The paragraphs below detail our proposals for luminescent species*

The emission bands of neutral **NpMI** and **^{n}BuO-NpMI** were identical (λ_{max} = 412 nm) when excited at 352 nm (Figure S9). The emission band corresponded to the determined excitation, exhibiting maxima at 320 nm (both **NpMI** and **^{n}BuO-NpMI**), 355 nm (**NpMI**) and 360 nm (**^{n}BuO-NpMI**). The differences between excitation and emission band maxima were ca. 50 nm in both cases, corroborating fluorescence from the singlet state, rather than phosphorescence. The excitation emission spectra herein accord with the absorption fluorescence spectra of a previous report.[50]

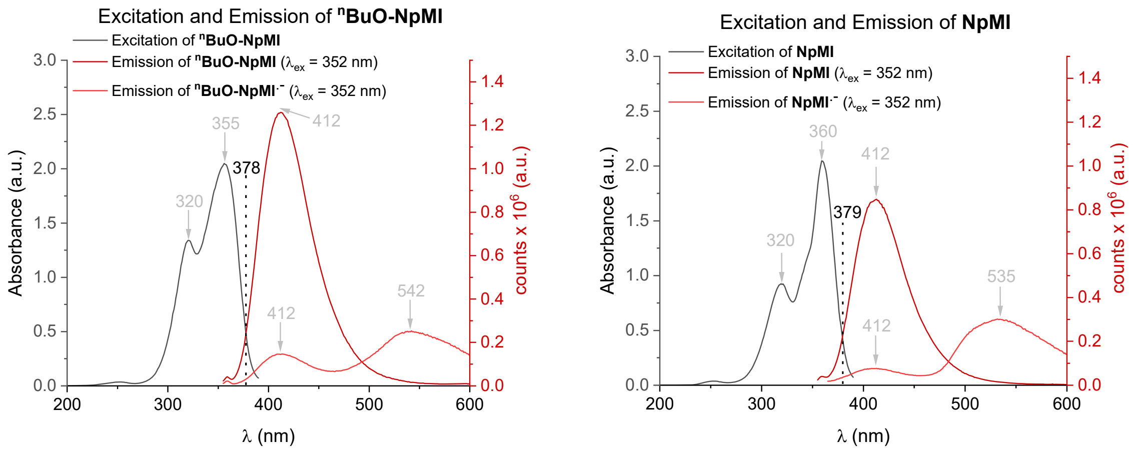

Figure S9. Excitation and emission spectra of the used catalysts in neutral and electroreduced forms: **^{n}BuO-NpMI** (left), **NpMI** (right), at excitation of λ = 352 nm.

The emission bands of **NpMI**$^{\bullet-}$ and **^{n}BuO-NpMI**$^{\bullet-}$ were similar (λ_{max} ≈ 540 nm) when excited at 352 nm. The electroreduction step was not 100% efficient, since neutral **NpMI** and **^{n}BuO-NpMI** remained (Figure S10). When excited at 440 nm, selective excitation of **NpMI**$^{\bullet-}$ and **^{n}BuO-NpMI**$^{\bullet-}$ revealed only the longer wavelength emitting species (λ_{max} ≈ 540 nm). The emission band corresponded to a determined excitation spectra, exhibiting maxima at ca. 282 nm (both **NpMI**$^{\bullet-}$ and **^{n}BuO-NpMI**$^{\bullet-}$, at 378 nm (both **NpMI**$^{\bullet-}$ and **^{n}BuO-NpMI**$^{\bullet-}$), at 460 nm (**^{n}BuO-NpMI**$^{\bullet-}$) and 466 nm (**NpMI**$^{\bullet-}$). The differences between excitation and emission band maxima were ca. 90 nm in both cases, corroborating phosphorescence. The excitation band was different from the UV vis absorption bands of **NpMI**$^{\bullet-}$ and **^{n}BuO-NpMI**$^{\bullet-}$ generated by spectroelectrochemical or preparative electrolytic measurements (Section S12-13). Therefore, the emission belongs to a different photoactive species, not the doublet state of the radical anion, but a lower energy, longer-lived excited state (termed ES_1).

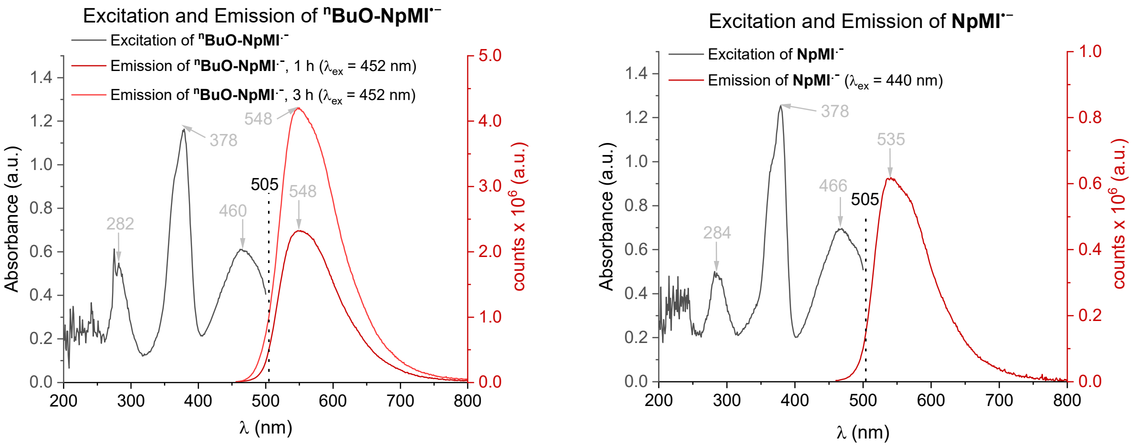

Figure S10. Excitation and emission spectra of the used catalysts in the reduced forms: **^{n}BuO-NpMI$^{\bullet-}$** (left), **NpMI$^{\bullet-}$** (right), at excitation of λ = 440 nm.

The intersection wavelength of the excitation (most red-shifted) and emission (most blue shifted) bands provides an estimate of the lowest excites state energy of the emitting species.[45] On the basis of literature and recorded lifetimes covered in the following section, we propose that emitters derived from neutral e-PRCats are singlet states (S_1), while quartet states (Q_1) could be candidates for the emitters derived from electroreduced e-PRCats. For **NpMI** and **^{n}BuO-NpMI**, these intersection wavelengths are 379 nm (E^{S} = 75.4 kcal mol^{-1}) and 378 nm (E^{S} = 75.6 kcal mol^{-1}) respectively. For **NpMI$^{\bullet-}$** and **^{n}BuO-NpMI$^{\bullet-}$**, this intersection wavelength is estimated by extrapolation to be 505 nm (E^{ES1} = 56.6 kcal mol^{-1}) in both cases.

14.2. Lifetime measurements

Luminescence lifetimes were determined by time-correlated single photon counting (TCSPC) via a Horiba® Scientific DeltaPro™ system equipped with a Horiba Scientific DeltaDiode™ laser (pulsed excitation at 375 nm or 452 nm at with a typical pulse width of 80 ps and a maximum repetition rate of 100 MHz). For neutral catalysts, λ_{ex} = 375 nm was used. For electroreduced catalysts, λ_{ex} = 452 nm was used. For full specifications of the DeltaDiodes, see: https://www.horiba.com/deu/products/detail/action/show/Product/deltadiode-1101/)

An emission monochromator was not employed. Instead, a bandpass filter was used and photons counted correspond to wavelengths either >400 nm (neutral catalysts) or >500 nm (electroreduced catalysts). An ND1 filter Data were collected either on the 100 ns (neutral catalysts) or 400 ns (electroreducted catalysts) measurement range. Low absorption was maintained at the excitation wavelength (A = 0.1) by the use of an ND1 cut-off filter at the excitation and lifetimes were measured at the magic angle (54.7°).

Sample preparation follows that described in Section S13 for UV-vis spectroscopy. Lifetimes were obtained using Horiba DAS6 decay analysis software where decays were fitted to one or two exponentials (as necessary to minimize the χ^2 fitting value).

*NOTE – In this section, the labels **NpMI**$^{•−}$ and ^{n}**BuO-NpMI**$^{•−}$ are used which refer to the *samples*' spectral data measured after electroreduction of **NpMI** and ^{n}**BuO-NpMI**. We do not claim to assign lifetimes to doublet states. The paragraphs below detail our proposals for luminescent species*

Luminescence lifetime measurements on the reduced ^{n}**BuO-NpMI** revealed a biexponential decay for the emitting species. The lifetimes recorded for ^{n}**BuO-NpMI**$^{•−}$ (τ_1 = 6.8 ns, τ_2 = 19.5 ns) and **NpMI**$^{•−}$ (τ_1 = 8.1 ns, τ_2 = 20.3 ns), following electroreductions for 1 h, were very similar to each other, and were longer than their neutral precursors (Figure S11 and Figure S12, left vs. Figure S12, right). The longer contributor to the decay (ca. 20 ns) is likely to be an emitter longer lived than this number suggests due to the restricted measurement time domain. A quartet state (presumably, Q_1), resulting from rapid intersystem crossing from the excited doublet states (D_n) of the reduced e-PRCats, is one candidate for this emitting species, since our EPR studies confirm the radical anion's doublet state is initially formed and reactive to SET. EPR. However, other possibilities, such as aggregation of the radical anion[46] or an excimer cannot be ruled out at this stage.

The doublet states of similar species (perylene diimides and naphthalene diimides) are reported to be ultrashort(picosecond)-lived and do not undergo luminescence.[47-49] A previously reported attempt to measure the lifetime of a ^{2}[*N*-arylnaphthalimide]$^{•−}$* derived from a neutral precursor that is a direct analog of **NpMI**, was unsuccessful and led to rapid decomposition of the sample under the conditions of transient absorption spectroscopy.[47]

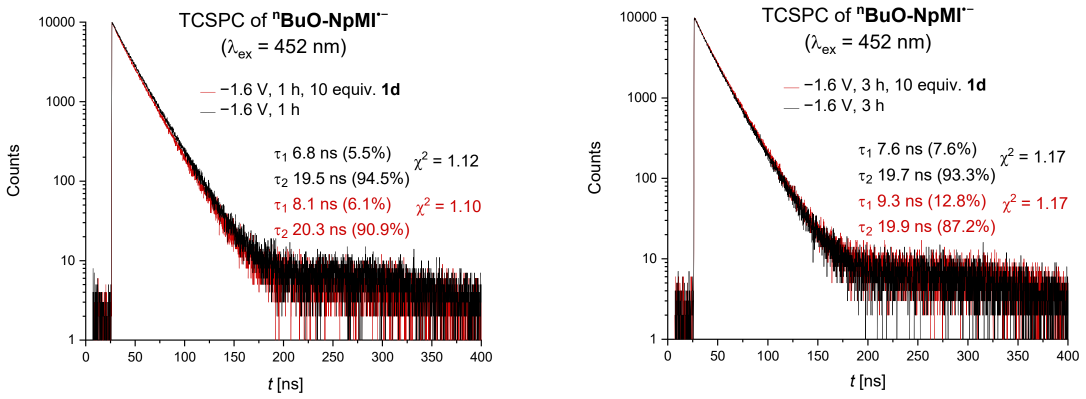

Figure S11. TCSPC of electroreduced ^{n}**BuO-NpMI** after 1 h (left) and 3 h (right) at −1.6 V.

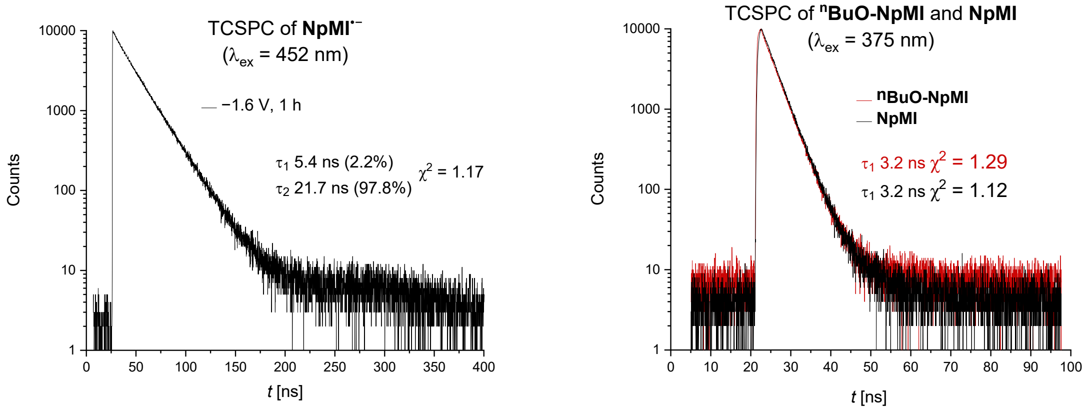

Figure S12. TCSPC of electroreduced **NpMI** after 1 h at −1.6 V (left). TCSPC of **^{n}BuO-NpMI** and **NpMI** neutral species (right).

The presence of **1d** (10 equiv.) did not significantly change the measured lifetime of **^{n}BuO-NpMI$^{\bullet-}$**. (Figure S11, left). We postulated whether the visual gradual color change from green to orange during an increased electroreduction time (from 1 - 3 h) would give rise to a different photoactive species, but similar results were obtained in each case (Figure S11, right).

A comparison of the neutral catalysts **NpMI** and **^{n}BuO-NpMI** reveals identical lifetimes of 3.2 ns (Figure S12, right). These lifetimes are longer than the previously reported lifetimes of singlet states of *N*-aryl-1,8-napthalimides,[50] where intersystem crossing is reported to occur to a triplet state (presumably, T_1).[51] However, phosphorescence is not reported for *N*-aryl-1,8-naphthalimides for which the *N*-aryl group rotation is considerably hindered. The discrepancy between lifetimes measured herein and therein[50] could be due to different sample preparations (inert, glovebox conditions were used herein, no mention is present in the previous study), different solvent media (0.1 M $^n Bu_4N.PF_6$ is present herein), or developments in TCSPC detector timing electronics over the last two decades.

Therefore, we assign the observed emitters are the singlet states (S^1) of **NpMI** and **^{n}BuO-NpMI**.

15. Spectroscopic Investigations of Preassociation

***NOTE –** In this section, the label **^{n}BuO-NpMI$^{\bullet-}$** is used which refers to the *samples*' spectral data measured after electroreduction of **^{n}BuO-NpMI**.*

15.1. NMR spectroscopy

^{1}H NMR of **^{n}BuO-NpMI$^{\bullet-}$** was measured by performing the electroreduction of **^{n}BuO-NpMI** (2.5 mM) in MeCN-d_3 (0.1 M $^{n}Bu_4N{\cdot}PF_6$) within a glovebox and then transferring the cathodic chamber solution to a sealed NMR tube. As expected for the open shell radical anion, the NMR was silent (Figures S13-14). The process was repeated, adding the cathodic chamber solution to **1d** (10 eq. with respect to neutral precursor **^{n}BuO-NpMI**) before transferring the resulting solution to a sealed NMR tube. The NMR of **^{n}BuO-NpMI$^{\bullet-}$** again was silent and no significant shifts could be seen in the ^{1}H (aromatic or aliphatic) or ^{31}P NMR peaks of **1d** (Figures S15-19), likely due to the excess of **1d** present. Preassociation between **^{n}BuO-NpMI$^{\bullet-}$** and **1d** could not be detected by NMR experiments.

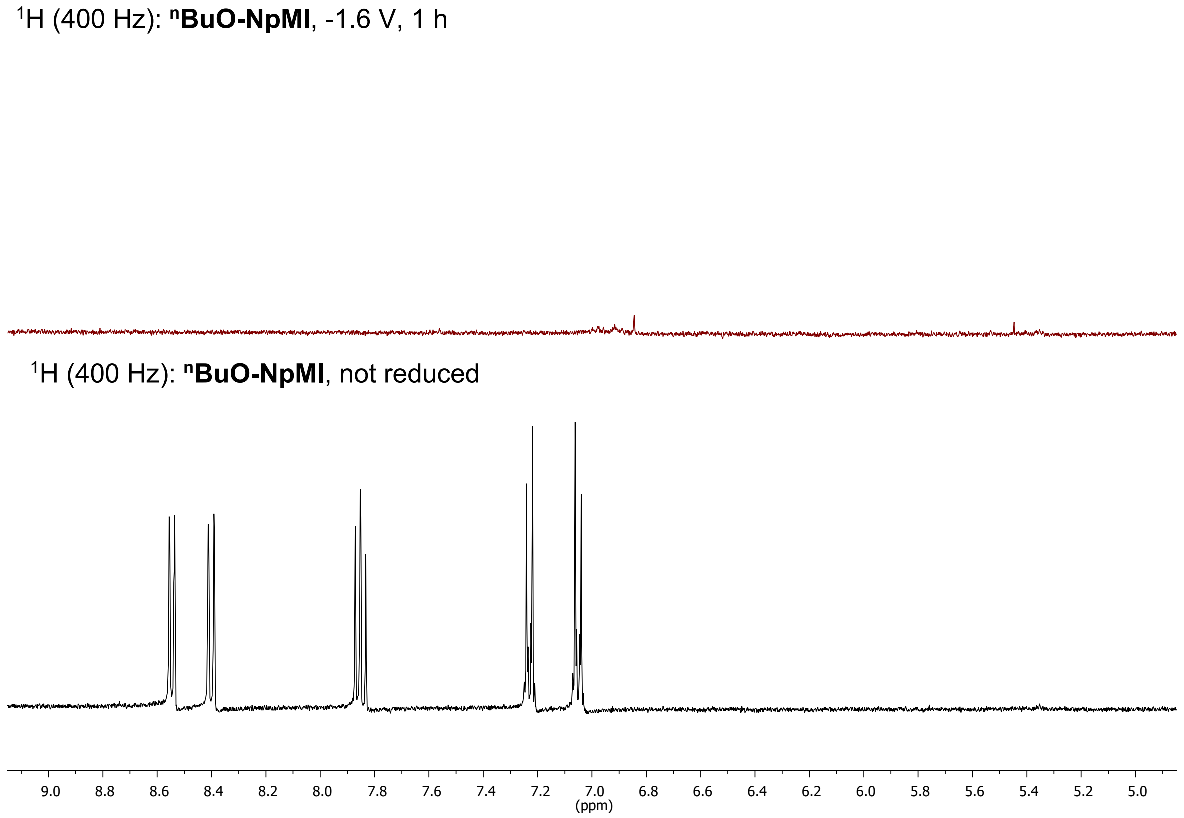

Figure S13. ^{1}H NMR of **^{n}BuO-NpMI** after (top) and before (bottom) electroreduction. Aromatic region.

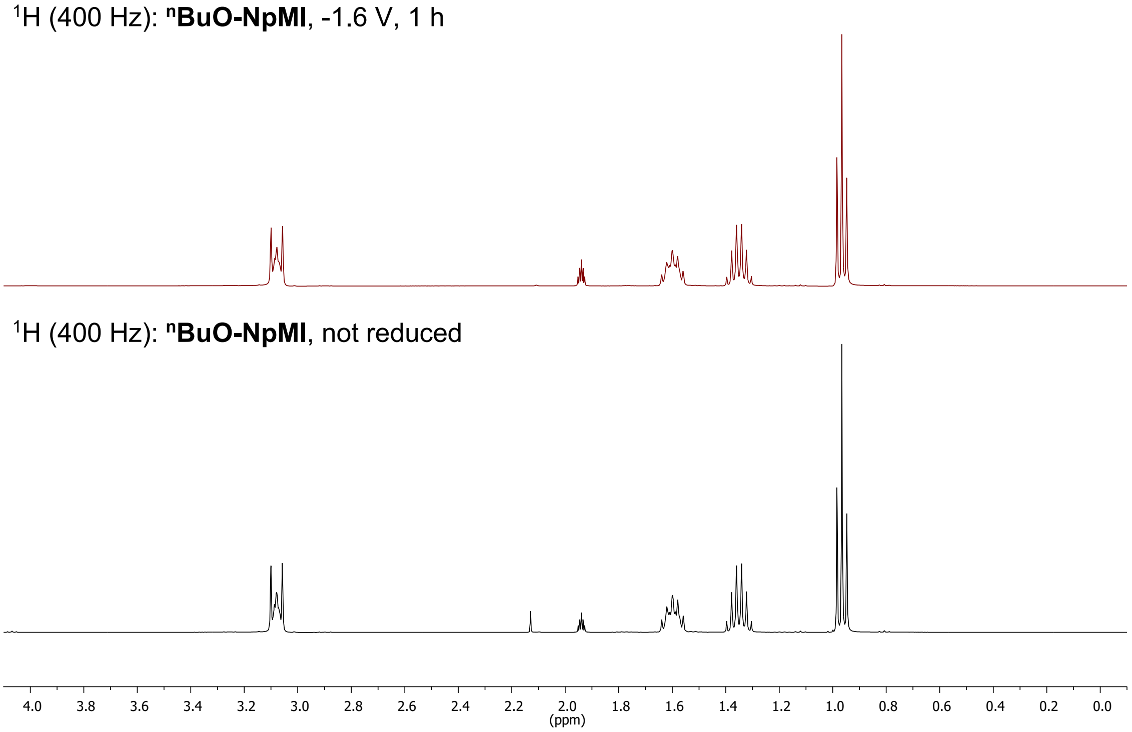

Figure S14. ^{1}H NMR of **^{n}BuO-NpMI** after (top) and before (bottom) electroreduction. Aliphatic region.

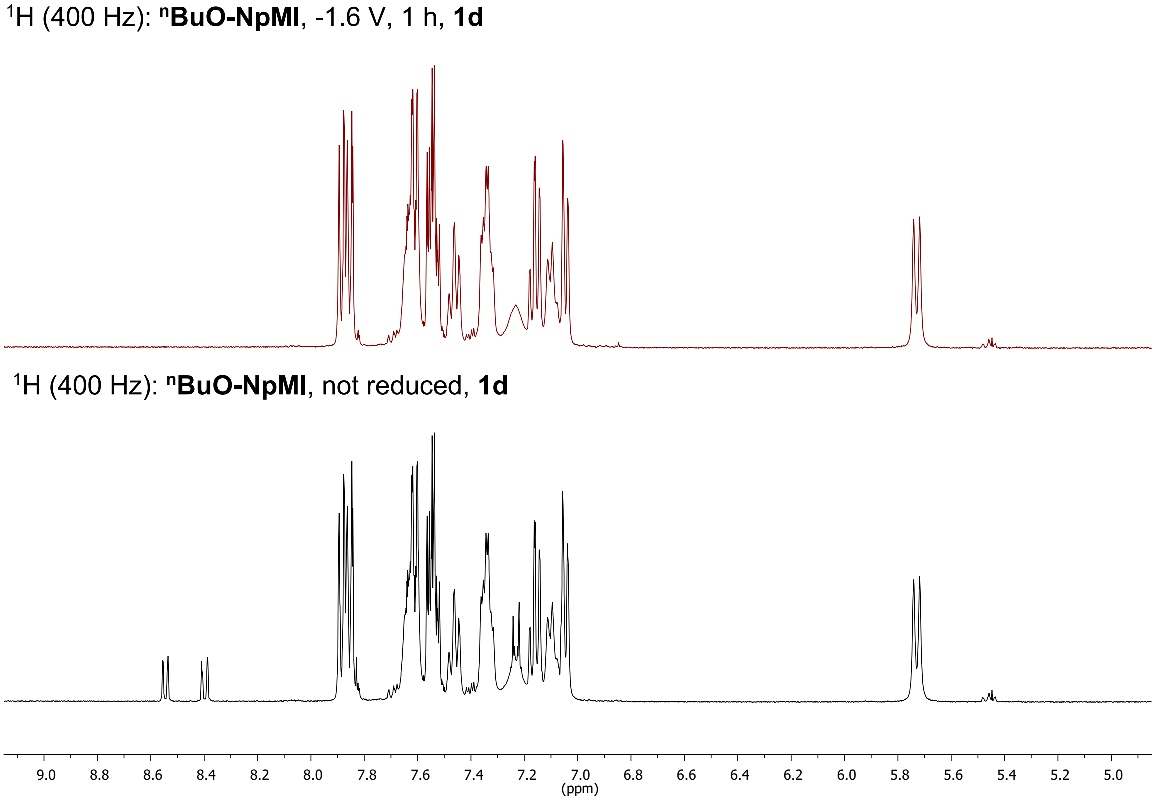

Figure S15. ^{1}H NMR of **^{n}BuO-NpMI** in the presence of **1d** (10 eq.) after (top) and before (bottom) electroreduction. Aromatic region.

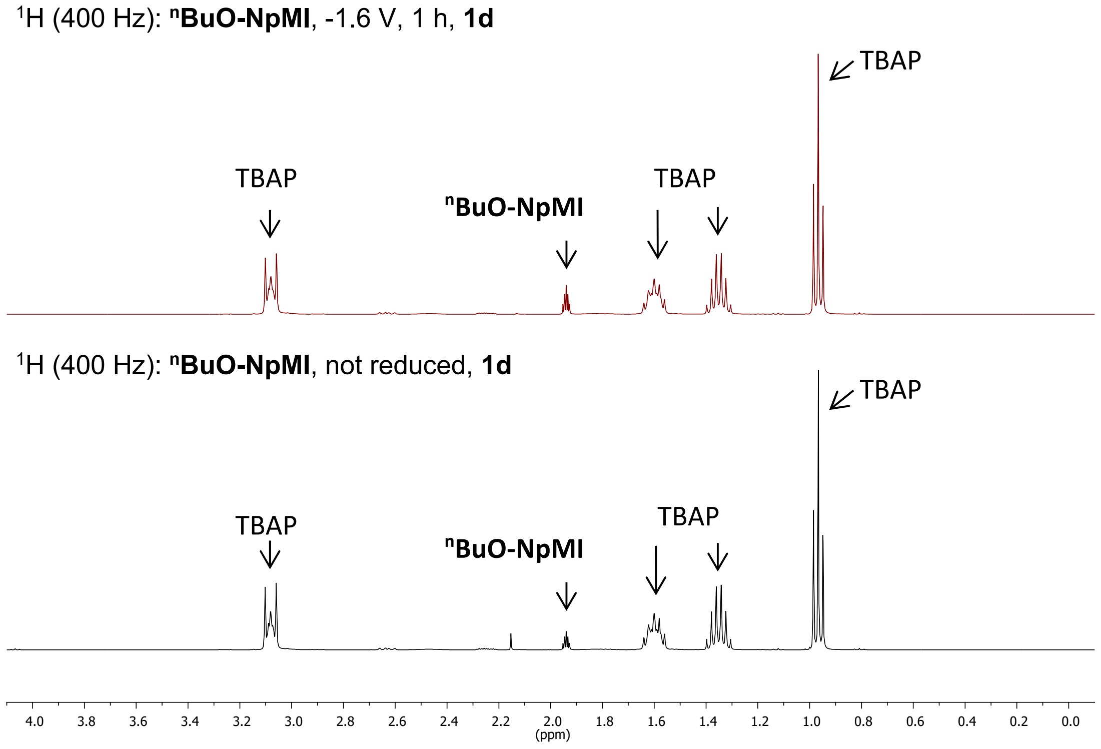

Figure S16. ^{1}H NMR of **^{n}BuO-NpMI** in the presence of **1d** (10 eq.) after (top) and before (bottom) electroreduction. Aliphatic region.

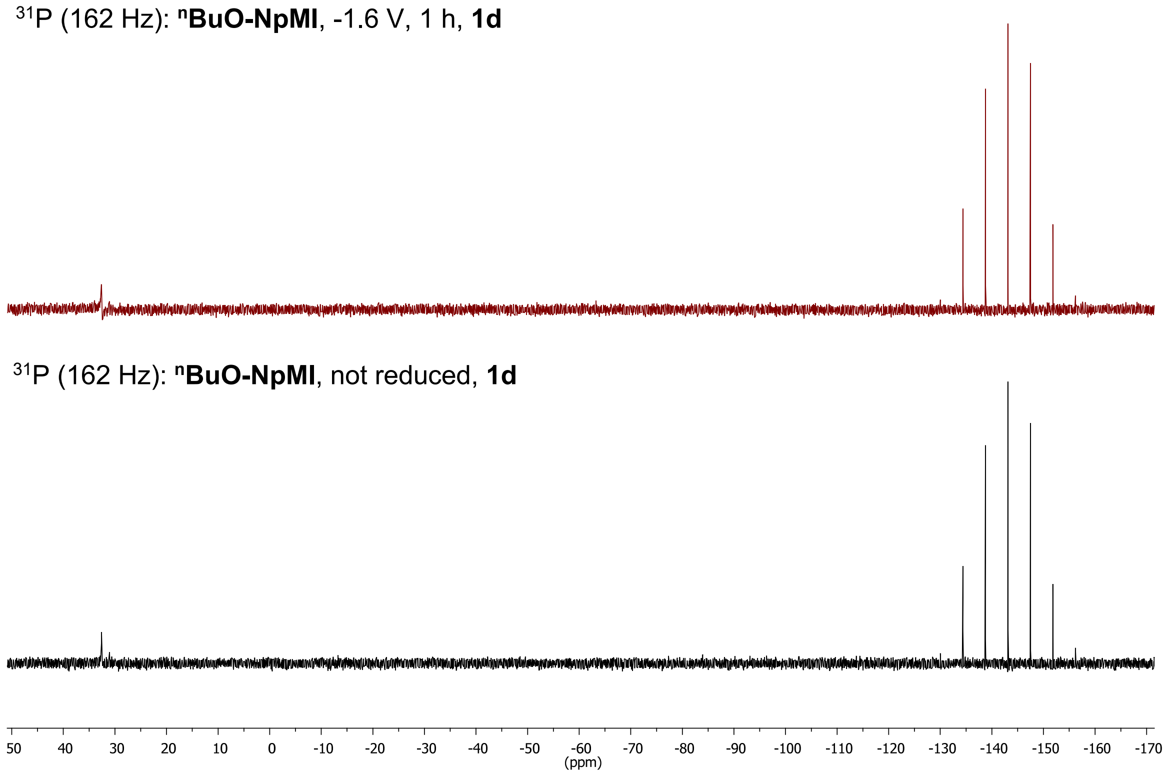

Figure S17. ^{31}P NMR of **^{n}BuO-NpMI** in the presence of **1d** (10 eq.) after (top) and before (bottom) electroreduction.

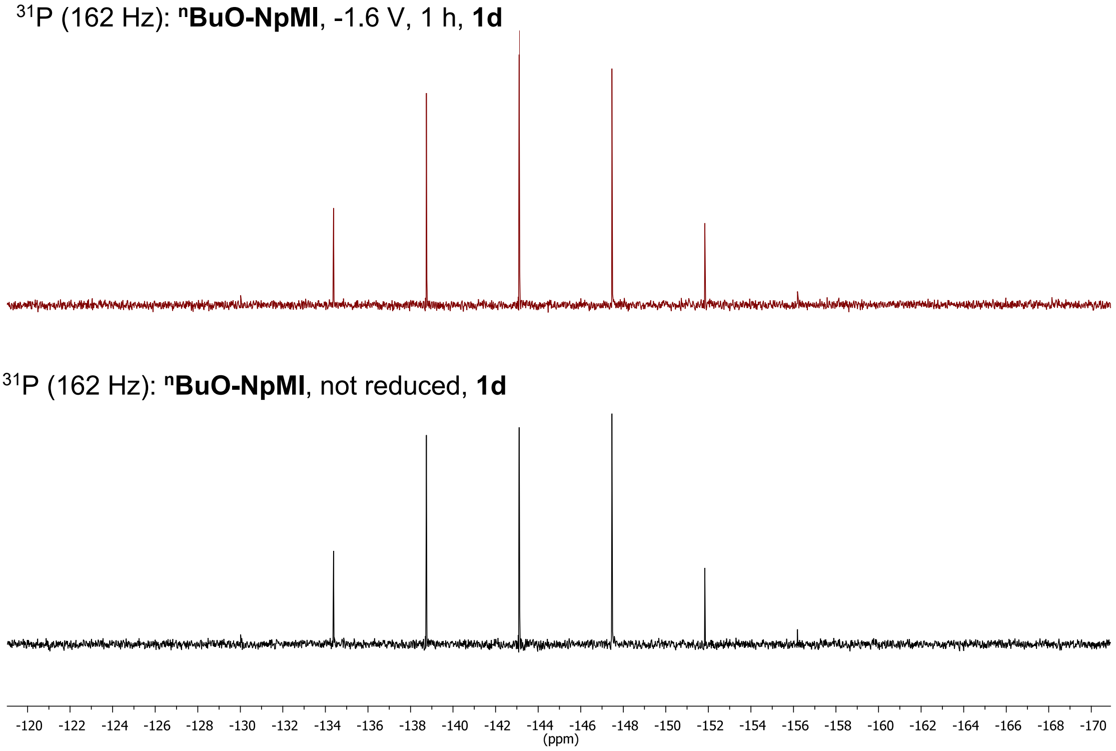

Figure S18. ^{31}P NMR of **^{n}BuO-NpMI** in the presence of **1d** (10 eq.) after (top) and before (bottom) electroreduction (expanded, electrolyte region).

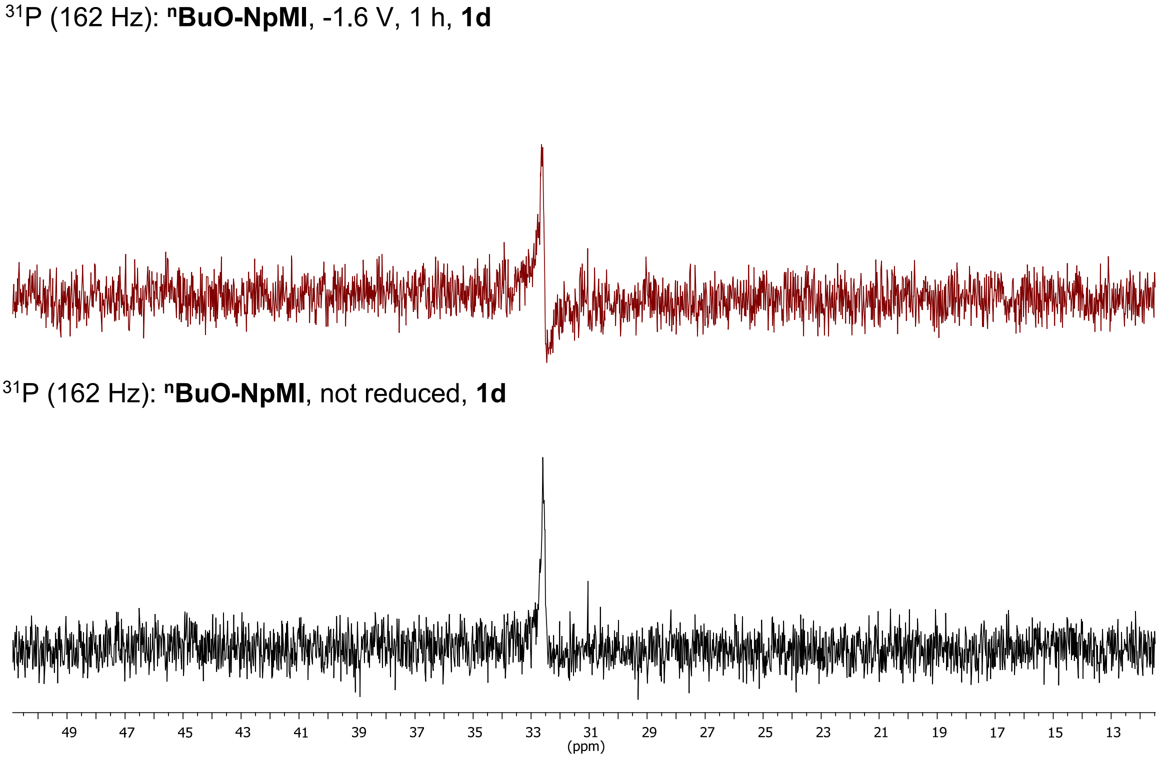

Figure S19. ^{31}P NMR of **^{n}BuO-NpMI** in the presence of **1d** (10 eq.) after (top) and before (bottom) electroreduction (expanded, **1d** region).

15.2. FT-IR Spectroscopy

***NOTE –** In this section, the label **ⁿBuO-NpMI•⁻** is used which refers to the *samples*' spectral data measured after electroreduction of **ⁿBuO-NpMI**.*

FT-IR spectra of **^{n}BuO-NpMI$^{\bullet-}$** were measured by performing the electroreduction of **^{n}BuO-NpMI** (2.5 mM) in MeCN (0.1 M ^{n}Bu$_4$N·PF$_6$) within a glovebox and then transferring the cathodic chamber solution to a NaCl cell as a thin film. The process was repeated, adding the cathodic chamber solution to **1d** (1.0 eq. was used with respect to neutral precursor **^{n}BuO-NpMI**) before transferring the resulting solution to the FTIR cell between NaCl discs. Control samples of **1d** only or solvent only were prepared in the same way (without electroreduction). We opted for only 1.0 eq. of **1d** due to concerns that 10 eq. of **1d** (as used in ^{1}H NMR and UV-vis studies) would swamp the IR signals of **^{n}BuO-NpMI$^{\bullet-}$**. The IR signals of the solvent (0.1 M ^{n}Bu$_4$N·PF$_6$ in MeCN) dominated spectra and obvious shifts in the peak wavenumbers were not apparent (Figure S20). The only detectable small shifts occurred i) at 1668 cm^{-1} (**^{n}BuO-NpMI$^{\bullet-}$**) which red-shifted to 1666 cm^{-1} in the presence of **1d**, and ii) 1242 cm^{-1} (**^{n}BuO-NpMI$^{\bullet-}$**) which red-shifted to 1239 cm^{-1} in the presence of **1d** (Figures S21-22). While these spectral shifts appear minor, they cannot be dismissed as experimental artefacts, since other peaks in the IR spectra overlay perfectly (1827, 1711, 1039, 918 cm^{-1}). FT-IR spectroscopy is a highly sensitive technique and has been utilized in the detection of non-covalent interactions, such as CH-π interactions in secondary folding of proteins.[51] Shifts in vibrational frequencies of 2-5 cm^{-1} have been reported for dimers/trimers of aromatic compounds binding via noncovalent interactions.[52]

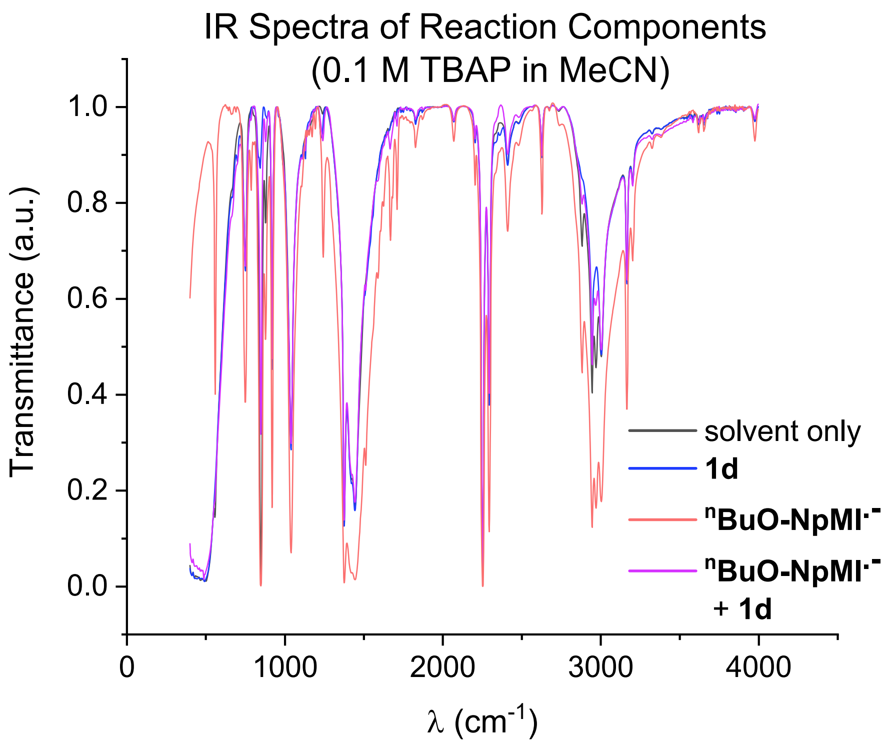

Figure S20. IR spectra of electroreduced **^{n}BuO-NpMI** in the presence of **1d** (1.0 eq.) and comparison to individual components.

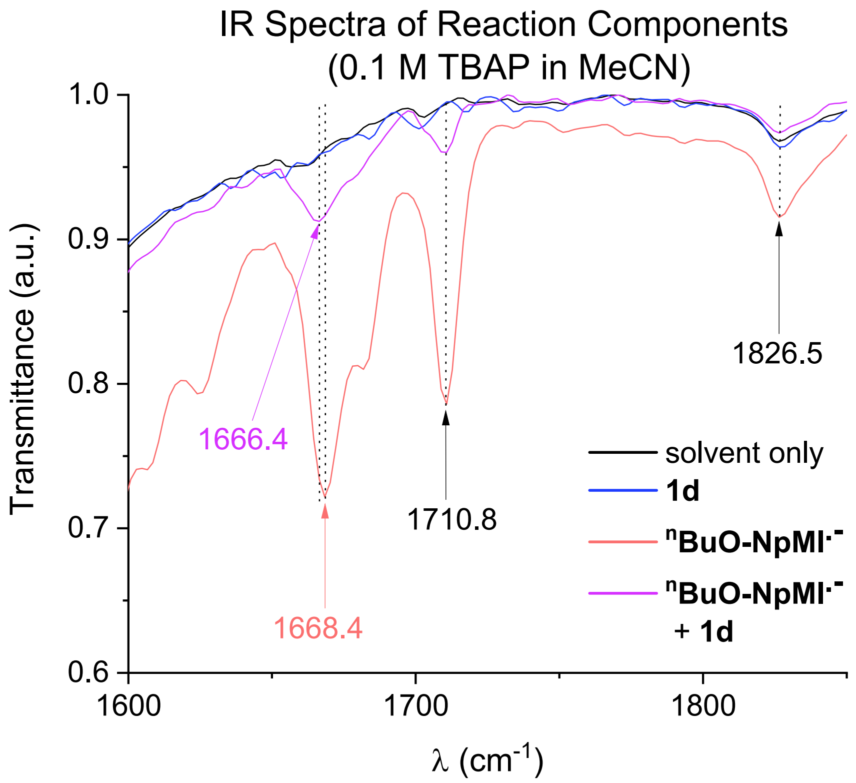

Figure S21. IR spectra of electroreduced **ⁿBuO-NpMI** in the presence of **1d** (1.0 eq.) and comparison to individual components. Expansion of 1600-1850 cm^{-1} region.

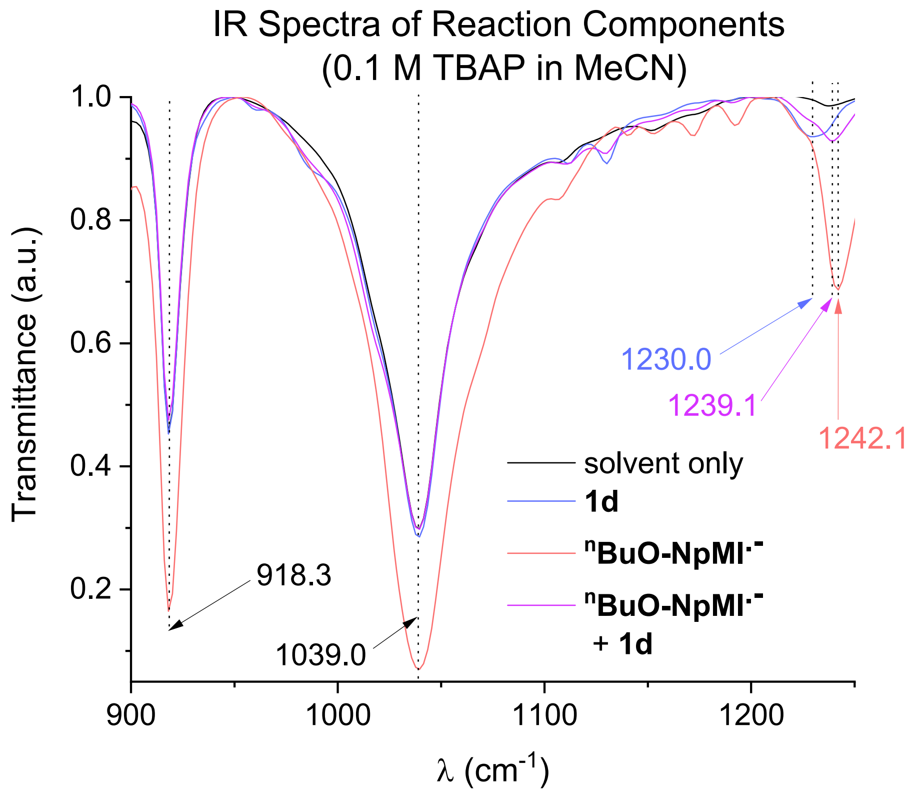

Figure S22. IR spectra of electroreduced **ⁿBuO-NpMI** in the presence of **1d** (1.0 eq.) and comparison to individual components. Expansion of 900-1250 cm^{-1} region.

16. EMISSION SPECTRA OF LEDs

Our method to compare LED emission intensity was previously reported,[2] using a BWTEK Inc. Examplar LS optical fiber spectrometer. Reproducibility of the method was confirmed in our previous study by comparing different batches of commercially-supplied LEDs.[2] The wavelengths and relative emission intensities of LEDs used in this study are reported in Table S6. The preparative yields of the reaction forming **2d** are shown earlier in Table S1.

Although the relative intensity maximum and relative intensity total peak area of the 440 nm LEDs is higher than other LEDs overall (Figure S23), the area overlap of the 740 nm LED with the near-IR spectral bands of **^{n}BuO-NpMI$^{•−}$** is similar to the overlap of the 440 nm LED with the near-UV band (Figure S24, right). However, no product was observed with 740 nm LED irradiation. This supports the conclusion of higher order photoexcited state participation to rationalize wavelength dependence on yield. Higher power LEDs (400 nm, 519 nm) gave improvements in product yields from trace levels, but the standard 440 nm LED was always superior. Although the high power 400 nm LED has the greatest overlap of any LED with the near-UV spectral band of **^{n}BuO-NpMI$^{•−}$**, we propose that either (i) the neutral **^{n}BuO-NpMI** also competes for light at this wavelength and shields photoexcitation of the **^{n}BuO-NpMI$^{•−}$** or (ii) higher doublet states accessed at 400 nm do not exhibit charge transfer behavior as identified by DFT-MRCI calculations (Section 18).

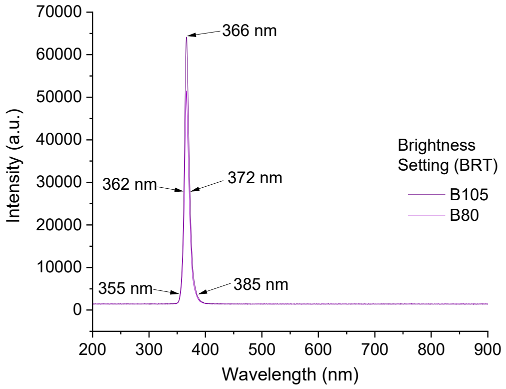

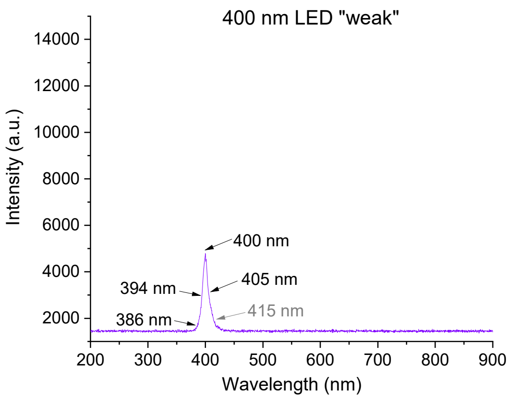

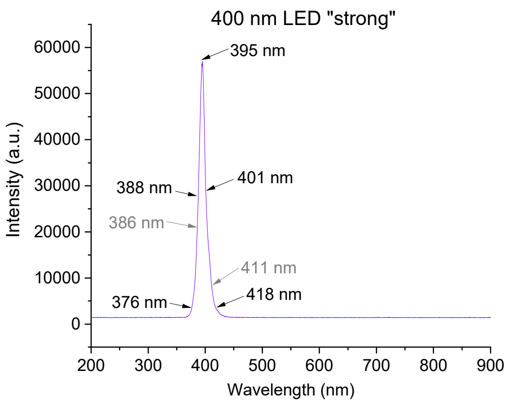

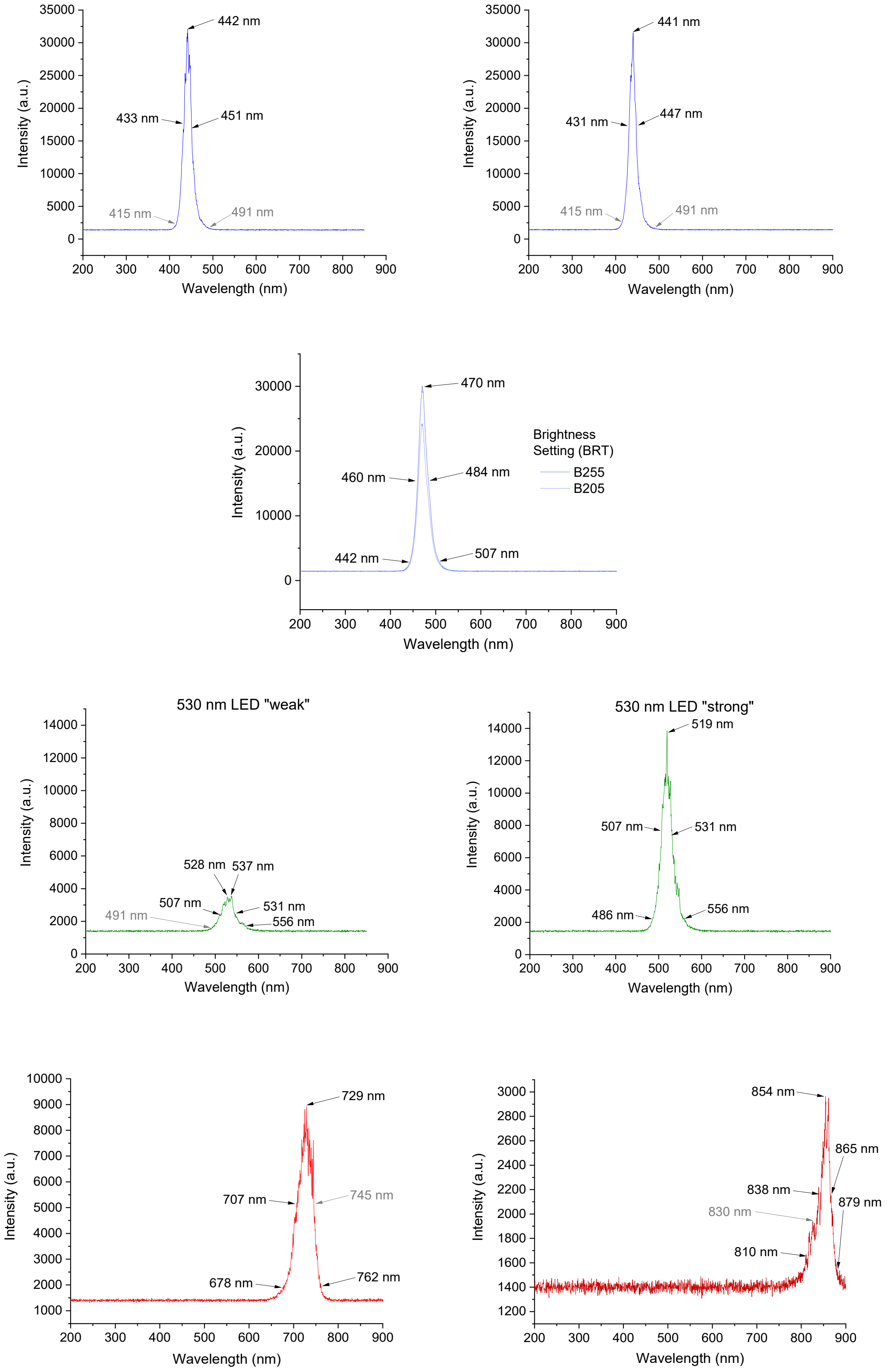

Figure S23. Emission spectra of LEDs used throughout the study at a fixed measurement distance.

Table S6. Characterization of LEDs used in this study and their measured wavelengths, optical powers and relative emitting intensities.

Manufacturer	Model/ Brand	Input Power per LED (W)	LED λ_{max} (nm)	Luminous Flux	Peak intensity, directly above LED[a] (a.u.) at λ_{max}	Peak area, directly above LED[a] (a.u.)	Peak intensity at λ_{max} of ^{n}BuO-NpMI
CCS (Creating Customer Satisfaction) Inc.	LDL-71X12UV12-365-N	1.5	366	[70 mW / cm^2][b]	57834[c]	627320[c]	-
Edison Edixeon	Edison EDEV-SLC1-03	3.5	400	0.35 W @ 700 mA	4788	43835	1859 (λ_{max} = 415 nm)
LED Engin	LZ440UB00-00U4	10.4	400	3.80 W @ 700 mA	56960	901278	5168 (λ_{max} = 415 nm)
OSRAM Oslon (batch 1)	Oslon SSL 80 LDCQ7P-2U3U LT1960	3.3	440	1.5 W @ 1000 mA	32040	693549	2215 (λ_{max} = 415 nm)<br>1698 (λ_{max} = 491 nm)
OSRAM Oslon (batch 2)	Oslon SSL 80 LDCQ7P-2U3U LT1960	3.3	440	1.5 W @ 1000 mA	31542	610066	2618 (λ_{max} = 415 nm)<br>1586 (λ_{max} = 491 nm)
CCS (Creating Customer Satisfaction) Inc.	LDL2-50X30BL2	7.6[d]	470	[100 mW / cm^2][b]	30049[e]	786117	-
OSRAM Oslon "weak"	Oslon SSL 80 LDCQ7P-2U3U LT1966	1.1	528	97 lm @ 350 mA	3508	89889	1539 (λ_{max} = 491 nm)
CREE "strong"	XPEGRN-L1-0000-00C02	1.1	519	150 lm @ 350 mA	13851	351401	2622 (λ_{max} = 491 nm)
LED Engin	LZ4-00R308	6.3	729	2.1 W @ 700 mA	8920	292177	5198 (λ_{max} = 745 nm)
LED Engin	LZ4-00R608	8.7	854	3.8 W @ 700 mA	2965	49816	1847 (λ_{max} = 830 nm)

[a]Measured by a BWTEC optical fiber spectrometer at a distance of 30 cm directly above the LED. The maximum observed intensity was recorded. The baseline (y axis) was ca. 1400 a.u. in each case. [b]Maximum intensity (mW / cm^2) reported by supplier at a 3 cm distance from LED. [c]The intensity-tunable LED was used at BRT100 setting for the preparative reaction. The mean average value of BRT105 and BRT80 (BRT95) was taken. [d]This input power drives a mini LED array (30 LEDs in a 20 mm x 50 mm area). [e]The intensity-tunable LED was used at the maximum power (BRT255) setting for the preparative reaction.

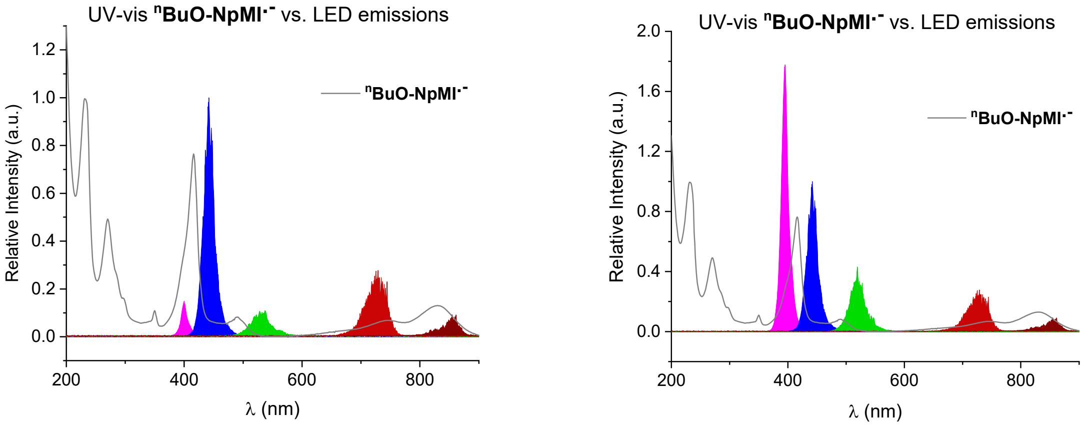

Figure S24. Overlay of UV-vis spectra of **^{n}BuO-NpMI$^{\bullet-}$** (spectroelectrochemistry) and LED emission spectra (calibrated relative intensities) of standard power LEDs used (left), or maximum available power LEDs (right).

17. Electron Paramagnetic Spectroscopy Investigations

As close as possible, EPR samples were prepared to mimic the photoelectrochemical reaction conditions. However, 10 eq. of **1d** were used instead of 20 eq. in the preparative reactions to ensure fully soluble conditions. EPR spectra were measured at 20 °C on a Magnettech MiniScope MS 400 spectrometer (9.45 GHz) which is a continuous-wave (CW) X-Band (9-10 GHz) spectrometer. Spectra were measured with a centre field of 340.7 G, a sweep width of 40.8 mT, a sweep time of 60 s, a resolution of 4096, a modulation amplitude of 0.2 mT, an microwave attenuation of 10.0 dB, a receiver gain of 20 and a time-constant of 0 s.

The electroreduction of **NpMI** and **^{n}BuONpMI** was performed in accordance to **Procedure 11** without substrate, within a nitrogen filled glovebox (MBraun UNILAB Plus). The electrochemically reduced samples were taken from the cathodic half-cell, without diffusion of anolyte into the sample, and transferred to Wilmad® quartz EPR tubes (O.D. = 1 mm, I.D. = 0.8 mm). Successful transfer was monitored by naked eye, as the reduced catholyte appears green (≤ 1 h, -1.6 V) or orange (>1 h, -1.6 V), exposure to air or contact with the anolyte vanishes the colour instantly. The quartz EPR tubes were placed in a larger quartz tube (O.D. = 5.0 mm, I.D. = 4.5 mm), which was sealed by an NMR cap and parafilm. If measurements were performed in presence of **1d**, the catholyte was added to a vial containing solid **1d**. After complete dissolution, the mixture was given into the quartz EPR tube. The sample was then covered in aluminium foil to prevent penetration of ambient light. Irradiation with blue light was done by a 440 nm single spot LED outside of the EPR-spectrometer. After irradiation for the specified time, the sample was returned to the spectrometer for measurement. Hyperfine couplings were extracted from simulations done in WINSIM2002 (fitting correlation = >0.995 in each case).[53]

Following electroreduction of **NpMI** for 1 h, a pentet signal at 337.8 mT was detected for **NpMI$^{\bullet-}$** (Figure S25, left). Following electroreduction of **^{n}BuO-NpMI** for 1 h, **^{n}BuO-NpMI$^{\bullet-}$** also appears as a

pentet at 338.2 mT (Figure S25, right). Hyperfine couplings, given in Table S7, were consistent with the literature for *N*-phenylnaphthalimide radical anion.[54]

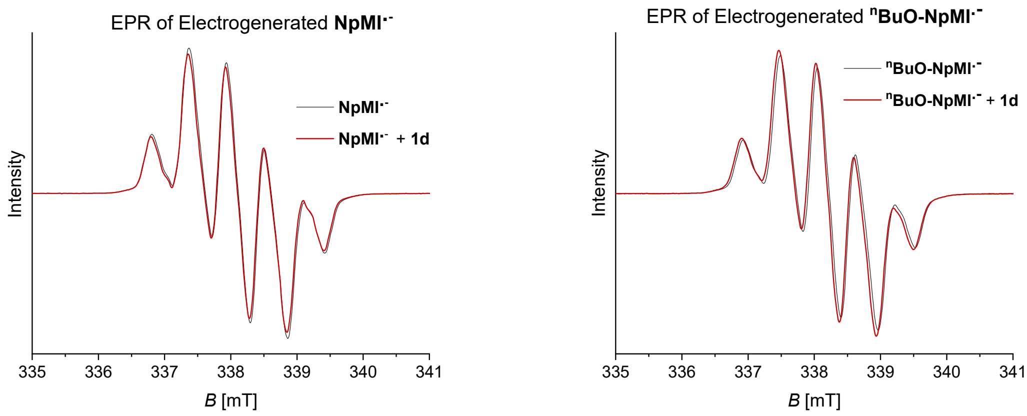

Figure S25. EPR spectra of electrochemically-reduced **NpMI** (left) and **^{n}BuO-NpMI** (right) in the absence and presence of **1d** (10 eq.).

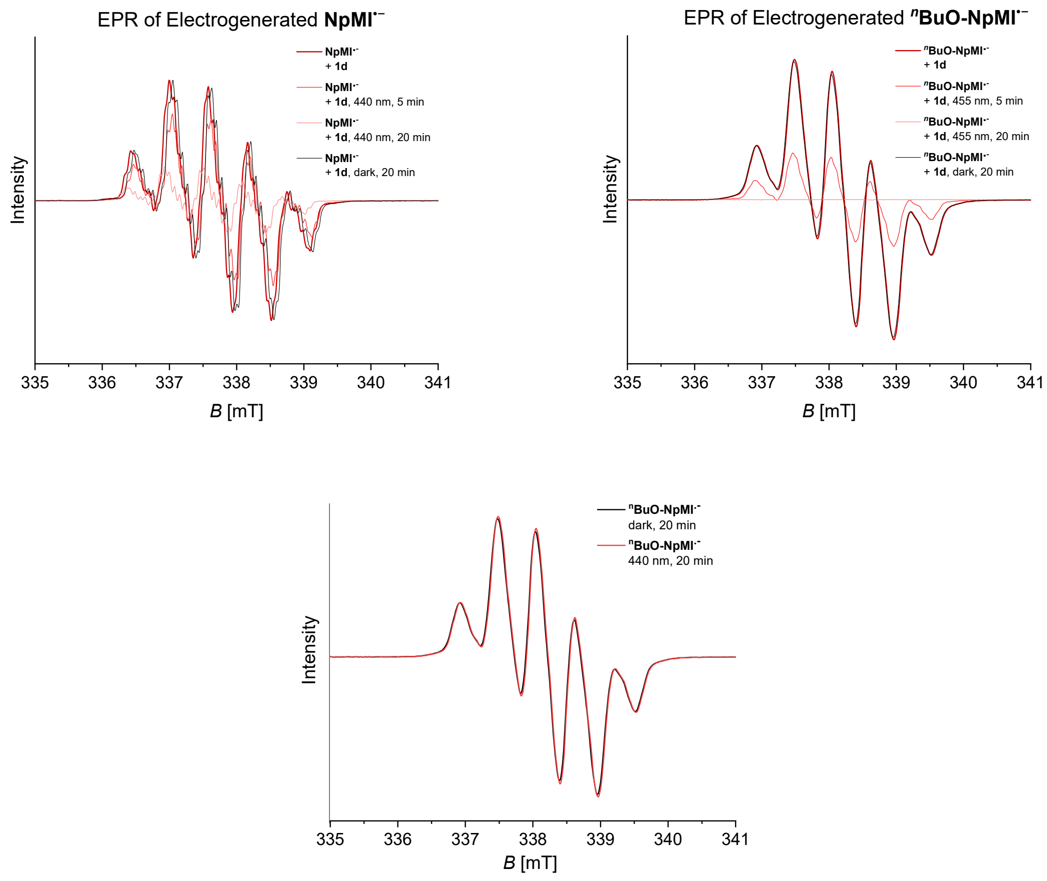

Figure S26. EPR spectra of electrochemically-reduced **NpMI** (top, left) and **^{n}BuO-NpMI** (top, right) in the presence of **1d** in the dark vs upon irradiation. Control experiment in the absence of **1d**, comparison of signal in the dark vs after irradiation (bottom).

In both cases, the intensity of the signal remains constant when kept in the dark for (monitored for up to 20 min, Figure S26). However, for both **NpMI•−** and **^{n}BuO-NpMI•−**, in the presence of **1d** and upon

irradiation with 440 nm for 5 min, signals are quenched to lower intensities. After 20 min of irradiation, signals almost completely disappear (Figure S26, top). Since both catalysts show similar behaviour, SET from both **NpMI•⁻** and **ⁿBuO-NpMI•⁻** to **1d** must be successful. This was surprising at first, since **NpMI** is an ineffective e-PRCat in transforming **1d** to its olefin product **2d**. Therefore, a subsequent step in the mechanism (likely C(sp^3)-O cleavage) must be rate-determining. For **ⁿBuO-NpMI•⁻** in the absence of **1d**, irradiation with 440 nm leaves the signal unchanged (monitored for up to 20 min, Figure S26, bottom). This evidences against the possibility of SET from **NpMI•⁻** and **ⁿBuO-NpMI•⁻** to the solvent or conducting salt, or suggests rapid back electron transfer should such a process occur.

Table S7. Simulated fitting of various EPR spectra and extracted hyperfine couplings.

Sample	α(H^{2,7})^a	α(H^{3,6})^a	α(H^{4,5})^a	α(N)^a
**NpMI•⁻**	4.99	0.69	5.45	1.44
**ⁿBuO-NpMI•⁻**	4.77	0.80	5.61	1.43
**ⁿBuO-NpMI•⁻ + 1d**	4.74	0.65	5.77	1.40

^{a}Determined using WINSIM2002 by simulated fitting of the spectrum, fitting R > 0.975 in each case.[23]

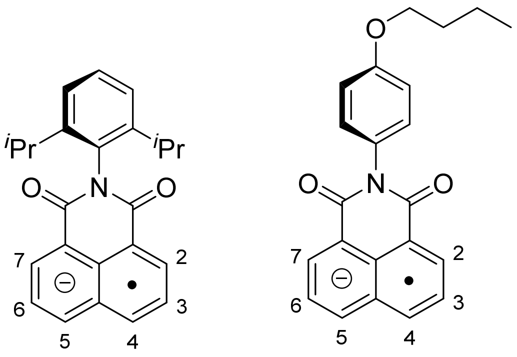

Figure S27. Assignments of EPR hyperfine couplings.

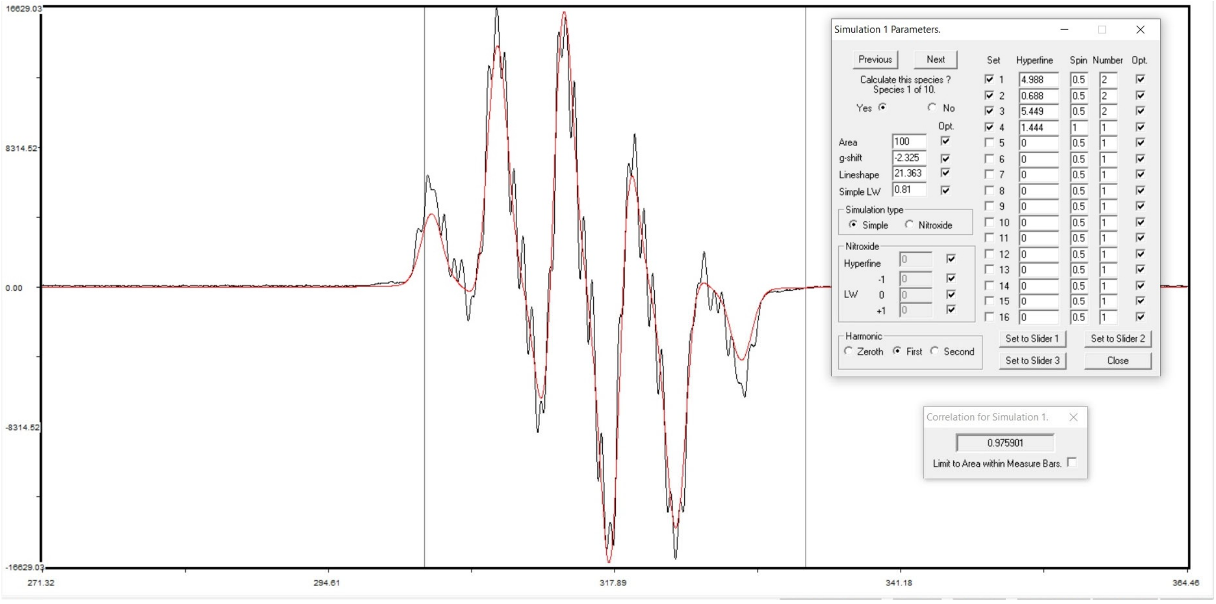

Figure S28. Simulation of the EPR spectrum of **NpMI•⁻**.

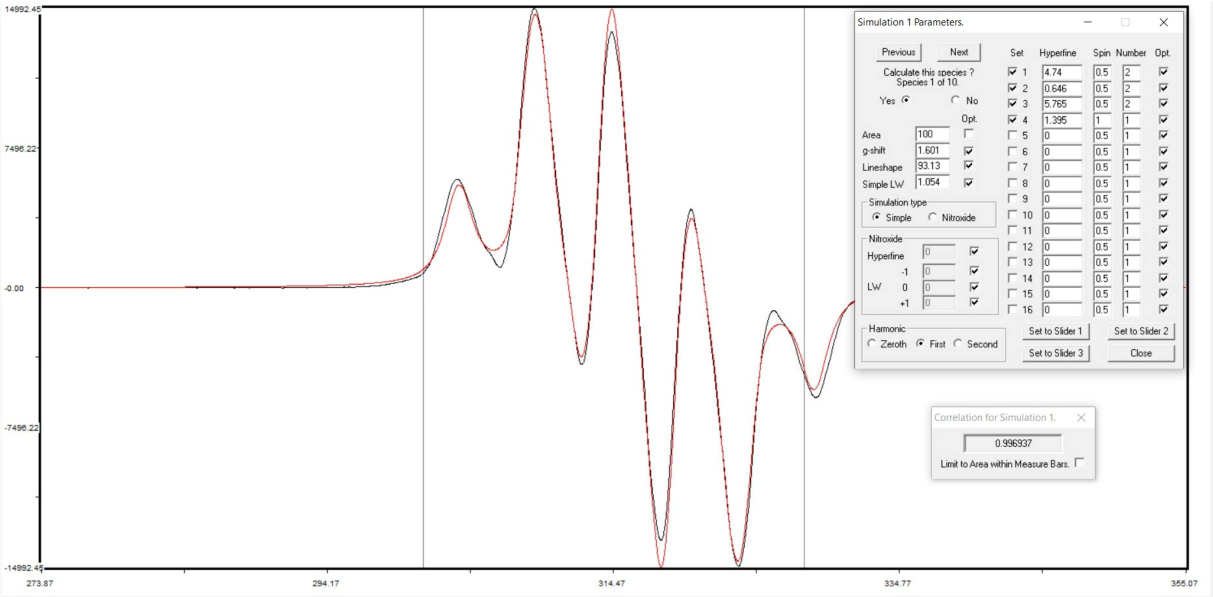

Figure S29. Simulation of the EPR spectrum of **^{n}BuO-NpMI$^{\bullet-}$**.

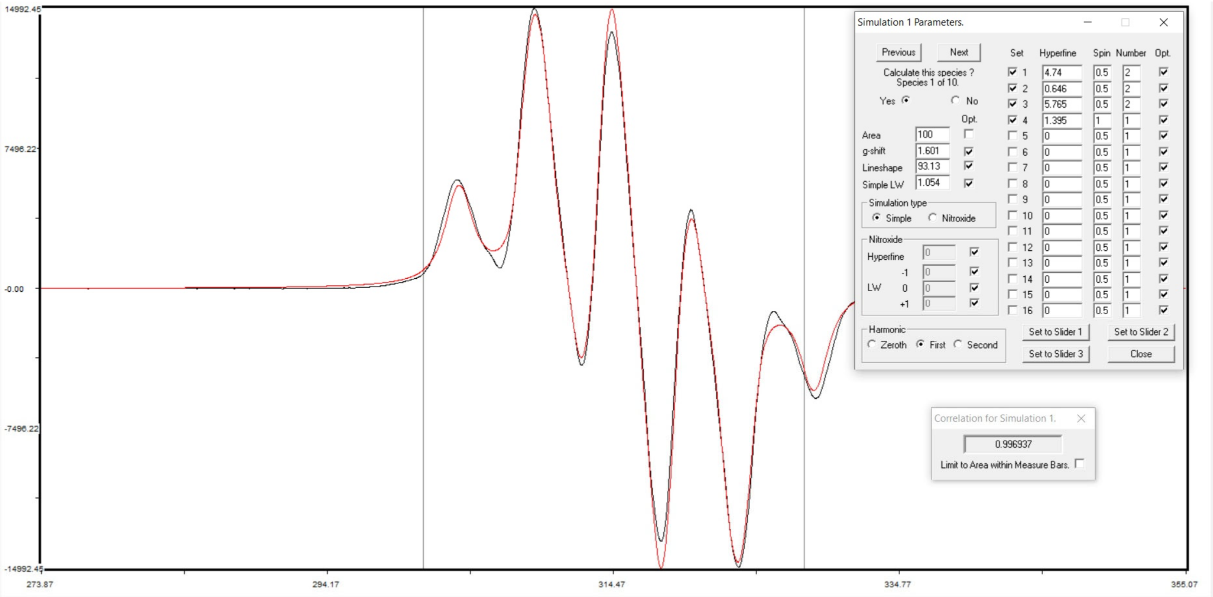

Figure S30. Simulation of the EPR spectrum of **^{n}BuO-NpMI$^{\bullet-}$**.

18. COMPUTATIONAL INVESTIGATIONS

18.1. General Information

All calculations were performed using Density Functional Theory (DFT)[55] using the Gaussian16 software package.[56] All minima were optimized using the ωB97X-D functional[57] with the 6-311+G* basis set.[58] Solvation effects were included with the integral equation formalism polarizable continuum model (IEFPCM),[59] with default parameters for acetonitrile, in which preparative e-PRC reactions and spectroscopy were performed. Frequency calculations were performed on all optimized structures in order to characterize minima (zero imaginary frequencies). Spin densities and molecular orbitals depicted in the main article and herein were visualized using VMD 1.9.3[60] with an isovalue of 0.004 and 0.02, respectively.

18.2. Bond dissociation free energies

Benchmarks have shown[61] that the range-separated and dispersion-corrected ωB97X-D functional[57] is well-suited to main group thermochemistry, while the use of a triple-zeta basis set is generally recommended to mitigate basis set incompleteness errors.[62,63] To further validate our DFT results, we calculated the bond dissociation energies of selected compounds with the open-shell DLPNO-CCSD(T)[64] method implemented in ORCA 4.2.1.[65] We used the def2-TZVPPD[66] basis set with the def2-TZVPPD/C[67] correlation basis. Tight SCF and PNO convergence criteria were applied and solvation effects were treated with the conductor-like polarizable continuum model (C-PCM)[68] using default parameters for acetonitrile, in which e-PRC reactions and spectroscopy were conducted. The DFT energies are on average 3.5 kcal/mol smaller than those obtained with DLPNO-CCSD(T) (Table S8), but the deviation is systematic and the trend is well reproduced, thus validating the use of DFT in this context.

Table S8. Calculated C–O bond dissociation energies (kcal/mol) without thermodynamic corrections at the ωB97X-D/6-311+G* and DLPNO-CCSD(T)/def2-TZVPPD levels of theory.

Phosphinate radical anion	DFT	DLPNO-CCSD(T)	Difference
**1bw**	-23.44	-28.03	4.59
**1d**	-19.98	-23.47	3.49
**1g**	-25.77	-28.86	3.09
**1o**	-23.87	-27.53	3.66
**1aa**	-11.99	-15.10	3.10
**1al**	-8.69	-11.33	2.64

Optimized structures were obtained using the theory level (ωB97X-D/6-311+G*) and solvent model (IEFPCM) specified in the general part. Bond dissociation free energies Δ*G* were obtained from the frequency analysis at 298.15 K and 1 atm according to:

$$\Delta G = \Delta(E_{\text{elec}} + ZPVE + \Delta G_{\text{trans,rot,vib}} + \Delta G_{\text{solv}})$$

Here, E_{elec} denotes the electronic energies, *ZPVE* is the zero point vibrational energy, $\Delta G_{trans,rot,vib}$ includes thermal contributions from translations, rotations and vibrations as well as entropic terms and ΔG_{solv} is the free energy of solvation.

18.3. Redox potentials

We investigated the reduction of a phosphinate substrate *P* by one electron, given by:

$$P + e^- \rightarrow P^{\cdot -}$$

The free energy change *ΔG* of this reaction contains the thermally corrected energies *G* of reactant and product as well as the thermal contributions of the free electron. There are multiple conventions to include the latter[69] and we have chosen the ionic convention where $G(e^-)$ = 0 eV.

$$\Delta G = G(P^{\cdot -}) - G(P) - G(e^-)$$

The absolute redox potential E°_{abs} is related to *ΔG* via:

$$E^\circ_{\text{abs}} = \frac{-\Delta G}{nF}$$

where *n* is the number of electrons involved in the redox process and *F* is the Faraday constant. Since we are only considering one-electron processes here, *n* is 1 in all cases. By supplying the energy in eV, the Faraday constant equals the elementary charge and the value of E°_{abs} is simply the negative change in free energy. *ΔG* is often calculated[70] by an indirect approach via a thermodynamic cycle. There, the reaction free energy is calculated for the gas phase geometries and the free energy of solvation ΔG_{solv} is added separately, possibly at a different level of theory, while neglecting the structural relaxation in solution. The success of this strategy is rooted at least partially in error cancellation effects.[71]

We therefore decided to use a simpler approach and calculated *ΔG* directly at the optimized geometries of reduced and oxidized species in solution,[71a] using the theory level (ωB97X-D/6-311+G*) and solvent model (IEFPCM) specified in the general part. To compare the calculated potential E°_{abs} to experimental results, it must be referenced against a standard potential:

$$E^\circ = E^\circ_{\text{abs}} - E_{\text{ref}}$$

Here, we used the Fc^+/Fc couple as an internal standard against the saturated calomel electrode (SCE), whose absolute reduction potential in acetonitrile has been determined computationally as 4.988 V.[72]

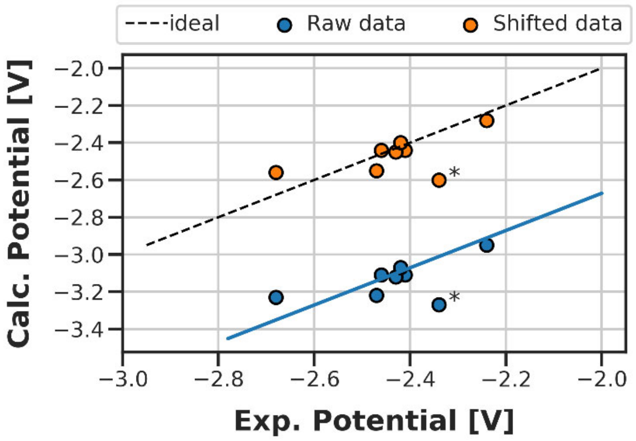

Figure S31. Correlation between experimental and calculated redox potentials. The dashed black line represents the ideal correlation between experiment and theory (slope 1 and y-intercept 0). The blue points and line denote the raw data obtained from calculations and the orange points illustrate the final potentials reported in the paper. The starred data point (**1a**) was omitted from the fit as an outlier. R^2 of the linear regression is 0.54.

The resulting redox potentials should ideally correlate 1:1 with the experimental ones (black line in Figure S31). However, because of experimental factors that cannot be reproduced well in calculations, such as conformational variations, as well as systematic errors specific to the chosen DFT functional and solvation model, we can expect a certain deviation from the ideal values. To correct for these deviations, we fitted a linear function with slope 1 to the data set of experimental and calculated potentials (blue line). In addition to the phosphinates in Table 2 of the main paper, we included compound **4a** (ΔE^{exp} = -2.24 V, ΔE^{calc} = -2.27 V) in the calibration protocol to enlarge the data set. In contrast, compound **1a** (marked with a star in Figure S31) was removed from the fit as an outlier. The y-intercept of the fit function (-0.67 V) represents the systematic shift of the calculated potentials and is on the higher end of the error margin reported for other DFT calculations.[70a-b,73]

Taking this into account, the final potentials reported in Table 2 are calculated as:

$$E^{\circ} = E^{\circ}_{\mathrm{abs}} - E_{\mathrm{ref}} + 0.67\,\mathrm{V}$$

18.4. DFT/MRCI calculations

The ground state geometry of **^{n}BuO-NpMI$^{\bullet-}$** was optimized at the ωB97X-D/6-311+G* level of theory with Gaussian 16 (for xyz-coordinates, see Section 18). For excited state calculations, we employed the DFT/MRCI method[74] with the R2018 Hamiltonian.[74d] Here, the ground state DFT reference was calculated with ORCA 4.2.1[65] using the BHLYP functional.[75] The def2-TZVPD basis set[66] was used on all atoms within the resolution-of-the-identity formalism for Coulomb and exchange integrals[76] (RI-JK) in conjunction with the def2-TZVPD/C[67a] and def2/JK[77] auxiliary basis sets. Solvation effects were accounted for by the C-PCM model[68] with default parameters for acetonitrile. Symmetry was turned off (keyword *NoUseSym*) and the SCF convergence threshold was set to 10-7 Eh (keyword *SCFCONV7*). The DFT orbitals for the radical anion were calculated in the unrestricted Kohn-Sham formalism and transformed to quasi-restricted orbitals[78] prior to the MRCI calculation. Ten roots were

calculated in the MRCI step. The reference space was iteratively optimized using a tight selection threshold of 0.8 Eh (keywords *$esel 0.8, $dftparam tight*), until all leading configurations were contained in the reference space.

18.5. CASSCF calculations

To identify the putative quartet state ES_1, we performed CASSCF(11,10) calculations[79–81] with Molpro 2021.1[82–84] using the aug-cc-pVTZ[85-86] basis set. Density fitting[87] was used to approximate the two-electron integrals and speed up the calculations. To further reduce the computational cost, we built a model system for **^{n}BuO-NpMI$^{\bullet-}$** by replacing the O-ether residue in the DFT-optimized structure with hydrogen and symmetrizing the resulting geometry to the C_{2v} point group. The active space was chosen with the goal of including correlating pairs of π and π* orbitals as well as including a balanced amount of orbitals localized on the aniline and naphthalene subunits, respectively. The resulting active space contained 11 electrons in 10 orbitals (Figure S32). State averaging was performed over forty states, namely five doublet and five quartet states in each irreducible representation.

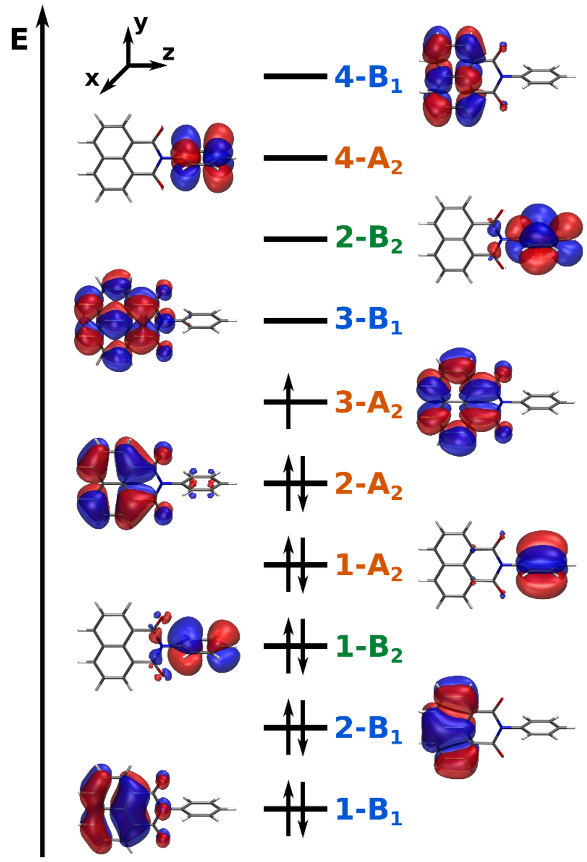

Figure S32. Active space of the CASSCF(11,10) calculation used to determine the energy of the putative quartet excited state. The naphthalene subunit resides in the yz-plane with the N-C bond aligned along the z-axis. Orbitals were visualized with VMD 1.9.3 using an isovalue of 0.02. The figure depicts the doublet ground state configuration.

The first ten states are summarized in Table S9. Compared to the DFT/MRCI spectrum (see Figure 3, main manuscript), the CASSCF energies are blue-shifted by 1-2 eV. This is within the usual range for

vertical excitation energies computed with CASSCF, due to the lack of dynamical correlation.[88] The tentative charge transfer doublet state, denoted D_n in the main manuscript is the fifth excited doublet D_5, in agreement with the DFT/MRCI results. Its vertical excitation energy at the CASSCF(11,10) level of theory is 4.34 eV and its dominant contribution is the single electron excitation 3-$A_2 \rightarrow$ 4-A_2. The transition dipole moment of this state is lower than predicted by DFT/MRCI, which is likely due to the symmetry constraints used in the CASSCF calculation.

Table S9. CASSCF(11,10) energies, norm of the transition dipole moments |μ| and dominant electronic transitions for the first ten electronic states of the ***n*BuO-NpMI**$^{\bullet-}$ model system.

State	Sym.	ΔE (eV)[a]	|μ| (a.u.)	Dominant transitions[b]
D_0	$^2A_2$	0.00	/	ground state
D_1	$^2B_1$	2.00	1.4219	3-$A_2 \rightarrow$ 3-B_1 (0.890)
D_2	$^2B_1$	2.30	0.0480	3-$A_2 \rightarrow$ 4-B_1 (0.904)
D_3	$^2A_2$	3.56	1.2666	1-$A_2 \rightarrow$ 3-A_2 (0.868)
Q_1	$^4B_1$	4.18	/	1-$A_2 \rightarrow$ 3-B_1 (0.936)
D_4	$^2B_2$	4.19	0.0022	3-$A_2 \rightarrow$ 2-B_2 (0.933)
D_5	$^2A_2$	4.43	0.0611	3-$A_2 \rightarrow$ 4-A_2 (0.927)
Q_2	$^4A_2$	4.72	/	1-$B_2 \rightarrow$ 2-B_2 (0.708)<br>2-$A_2 \rightarrow$ 4-A_2 (0.624)
D_6	$^2A_2$	4.73	0.0077	1-$B_2 \rightarrow$ 2-B_2 (0.576)<br>2-$A_2 \rightarrow$ 4-A_2 (0.503)
D_7	$^2B_1$	4.88	0.5000	2-$B_1 \rightarrow$ 3-A_2 (0.691)<br>1-$B_1 \rightarrow$ 3-A_2 (-0.307)

[a]Relative energies and transition moments are reported with respect to the doublet ground state. [b]CI coefficients for each transition are given in parentheses.

The first excited quartet state Q_1 has a vertical excitation energy of 4.18 eV. Taking into account the aforementioned blue-shift of 1-2 eV in CASSCF as well as vibrational relaxation in the excited state, this is in reasonable agreement with the new emission band at 540 nm (2.29 eV). The excitation energy of Q_1 is 0.25 eV below that of D_5 and only 0.01 eV below that of D_4. This indicates that coupling to the quartet domain is a possibility after excitation into one of these doublet states, at least from an energetic point of view. In light of the luminescence spectra reported in Section S14, it is reasonable to assume that the long-lived emitting species is indeed the lowest excited quartet state. This state with B_1 symmetry is characterized mainly by the single electron excitation 1-A2 → 3-B1, which corresponds to an opposite charge transfer from the aniline to the naphthalene subunit. EPR spectra (Section S17) and DFT calculations (Section S18.6) show that precomplexation of the substrate may occur on the aniline moiety. Therefore, Q_1 being the long-lived emitter state would explain why the luminescence is not quenched upon addition of substrate **1d** to the reaction mixture: In a precomplex, the charge would be shifted away from the substrate when the catalyst crosses to Q_1, thus impeding single electron transfer.

18.6. Computational Investigation of Preassembly Candidates

The variation of catalyst structure on the reactivity presented in the main article showed increasing yields for the reaction of **1d**, when reducing the steric bulk on the *ortho*-position of the catalyst's *N*-aniline moiety. Thus we performed DFT calculations to investigate possible arrangements for ground state precomplexation between substrate and the aniline unit of the catalyst radical anion. All calculations were performed at the ωB97X-D/6-311+G* level of theory with Gaussian16.[56] Solvation effects were included with the IEFPCM model,[59] with default parameters for acetonitrile. For the frequency analyses presented in this section, we reduced the two-electron integral accuracy threshold from 10^{-12} to 10^{-11} (keyword *int=(acc2e=11)*), because the calculations would not converge otherwise.

Due to their structural complexity, there are many ways for the phosphinate to dispersively bind to the radical anion catalyst. Assuming that the primary contributors to the binding mode are π-stacking interactions,[89] the P-bound phenyl groups as well as the aryl moiety from the benzylic position ("O-CHR-aryl") of the phosphinate may coordinate to the aniline or naphthalene moiety of the radical anion catalyst. Here, π-π stacked interactions (face-to-face, parallel displaced) as well as *T*-π shaped interactions (edge-to-face) are conceivable. We expect the strongest dispersive interactions when not just one but two of the substrate's aromatic units coordinate to the *N*-aniline group in a pincer-like complex. Indeed, the geometry optimizations shown later in this section always converged to an orientation where one of the aromatic units of the substrate coordinates (π-π, face-to-face) to the aniline ring and another one to the side (*T*-π, edge-to-face), regardless from the starting structure. To assess the accessibility of such a complex starting from isolated molecules, we first performed a series of unrelaxed potential energy scans. The scan coordinate was chosen such that the O-CHR-aryl component of substrate **1d** approaches the catalyst from the side and one of the P-bound phenyl units forms a T-π shaped complex with the aniline at small distances (Figure S32).

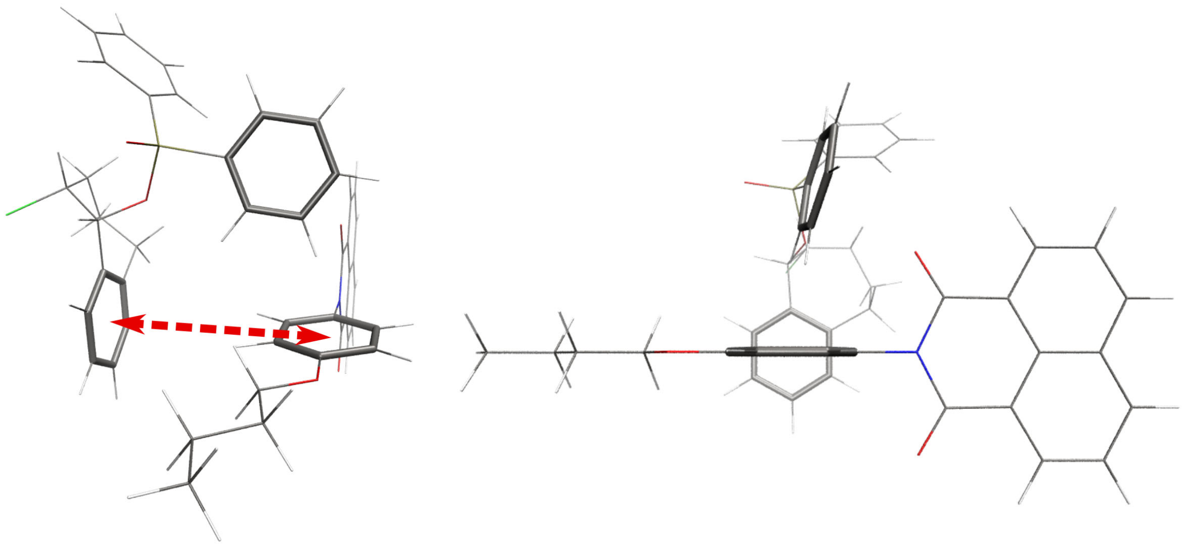

Figure S32. Two views on the orientation of **1d** and the catalyst (here **^{n}BuO-NpMI**) chosen for potential energy scans. The red arrow indicates the scan coordinate, i.e. the center-of-mass distance

between the aniline moiety of the catalyst and the O-CHR-aryl moiety of the substrate. The three interacting aromatic rings are highlighted in bold.

Two energy minima emerge along the scan coordinate (Figure S33, left). Going from large to small distances, the first minimum appears when the P-bound phenyl starts to interact dispersively with the *ortho*-substituent groups. It is followed by an energy barrier when the imide-oxygen and/or the *ortho*-substituent groups come in closer contact with the phenyl unit and repulsive terms presumably start to predominate. As could be expected, this barrier rises with increasing "steric bulk" on the aniline and makes the second minimum entirely inaccessible for **NpMI** (Figure S33, left). This second energy minimum represents the fully formed pincer-like complex, where the O-CHR-aryl moiety of the substrate is close enough to form dispersive T-π-stacking interactions with the aniline unit, which stabilizes the energy.

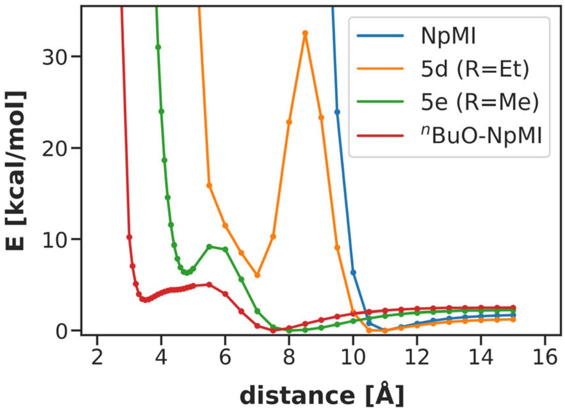

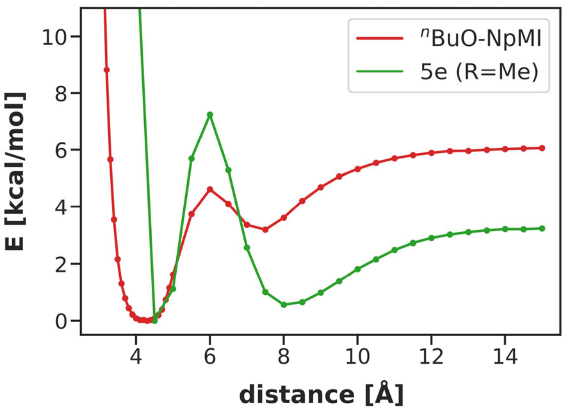

Figure S33. Potential energy scans along the approach of substrate **1d** towards the aniline unit of the catalyst. The scan coordinate refers to the center-of-mass distance between the O-CHR-aryl moiety and the aniline unit. Left: aniline and naphthalene units orthogonal, this is the ground state minimum of the catalyst. Right: naphthalene unit rotated by 90°, structure not relaxed. The minimum energy is shifted to zero in all scans.

For the less sterically hindered catalysts **^{n}BuO-NpMI** and **5e**, the aniline unit can in principle rotate with respect to the imide group. We calculated the rotational barrier in **^{n}BuO-NpMI$^{\bullet-}$** as 16 kcal/mol by performing a relaxed potential energy scan of the respective C-C-N-C dihedral angle between 90° and 180° with a step size of 2° at the same level of theory as before (Figure S34). In effect, the rotation of the aniline group decreases the repulsive "steric" interactions between one the imide oxygens and the P-bound phenyls, thus lowering the energy barrier for precomplexation and stabilizing the complex (Figure S33, right) even more so than the initial dispersive interaction with the *ortho*-substituent.

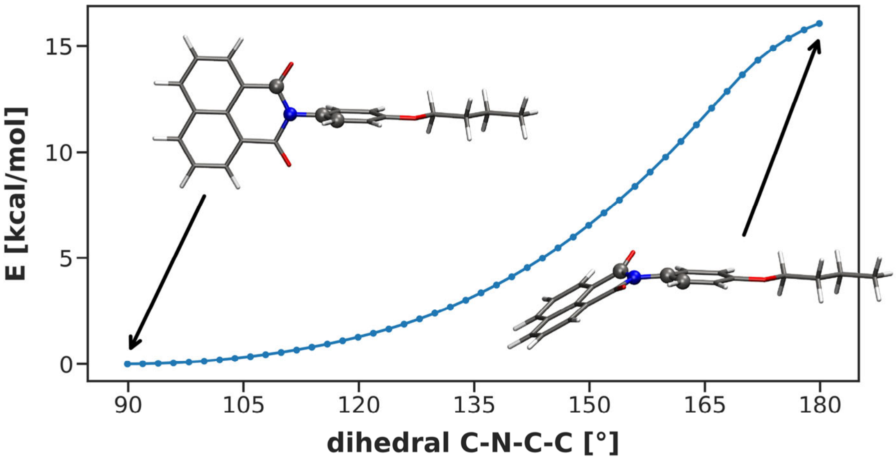

Figure S34. Relaxed potential energy surface scan for the rotation of the aniline moiety in **ⁿBuO-NpMI**. The four atoms defining the scanned dihedral angle are highlighted as spheres.

Assuming that a successful reaction requires formation of a precomplex, based on the arguments presented in the main manuscript:

(i) known picosecond lifetime of doublet states,[47-49] apparent anti-Kasha photochemistry,

(ii) the likely rate-limiting C(sp^3)-O cleavage and profound influence of catalyst structure on this step,

(iii) the identical redox (Section 11), UV-vis (Section 13) and emission (Section 14) properties of **NpMI** and **ⁿBuO-NpMI** that confirm electronic differences cannot explain the success of the latter catalyst for most substrates,

the potential energy scans depicted in Figure S33 could possibly explain the reactivity trend observed in the experimental SAR study. Increasing "steric bulk" at the *ortho*-position of the aniline impedes the formation of the most stable and intimate preassembly as the pincer-like π-stacking interaction becomes less accessible. Of course, an unrelaxed potential energy scan for complex molecular assemblies can only convey a rough picture of the real situation. For example, it neglects the internal flexibility of the substituents and the fact that the substrate can re-orient itself upon approaching the catalyst, thus minimizing clashes and stabilizing the complex. To take these effects into account, we optimized several candidate structures for a ground state precomplex. Using different catalysts and relative orientations, we were able to optimize 13 stable ground state preassemblies (Figures S35-39).

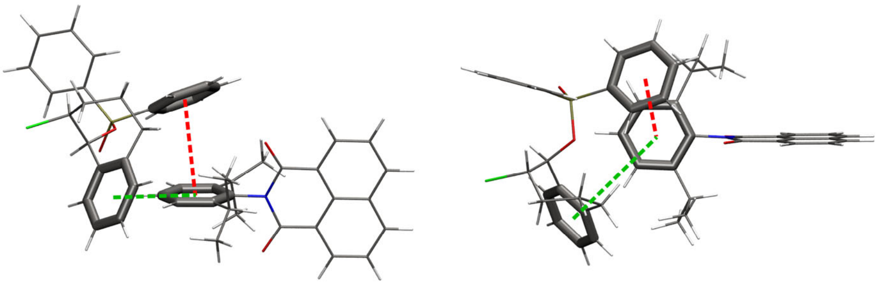

Orientation **1**

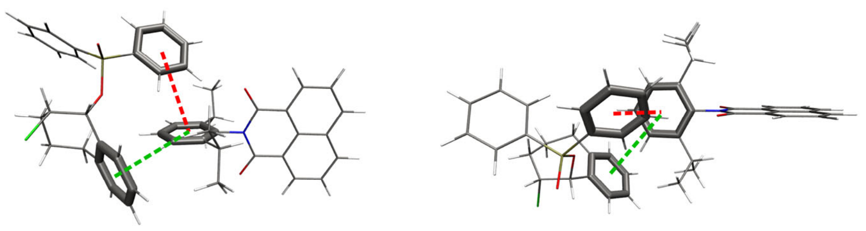

Orientation **2**

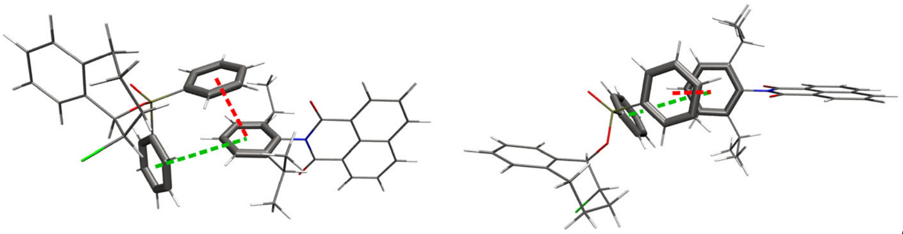

Orientation **3**

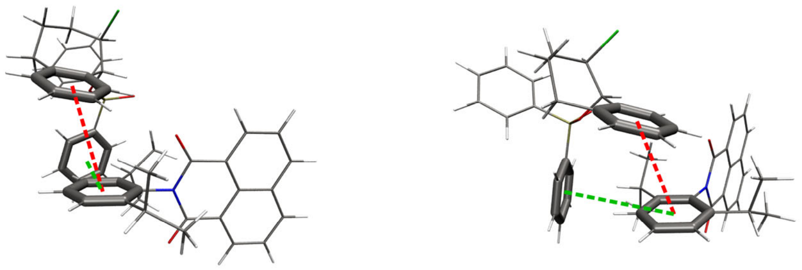

Orientation **4**

Figure S35. Candidate preassemblies of **NpMI**$^{\bullet-}$**/1d** at the *N*-aniline moiety.

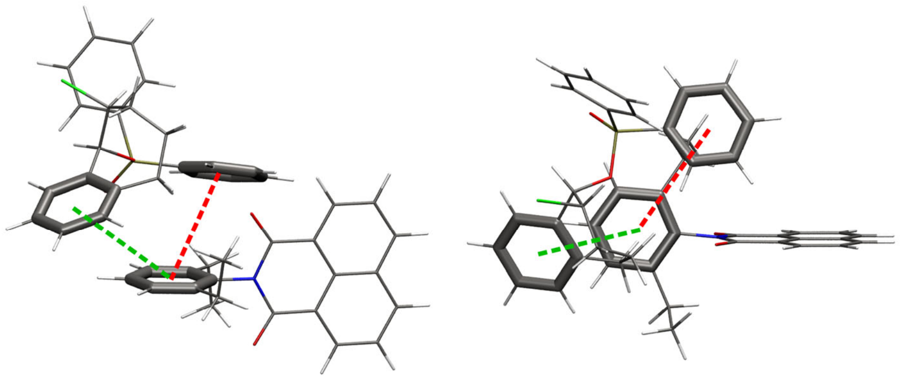

Orientation **1**

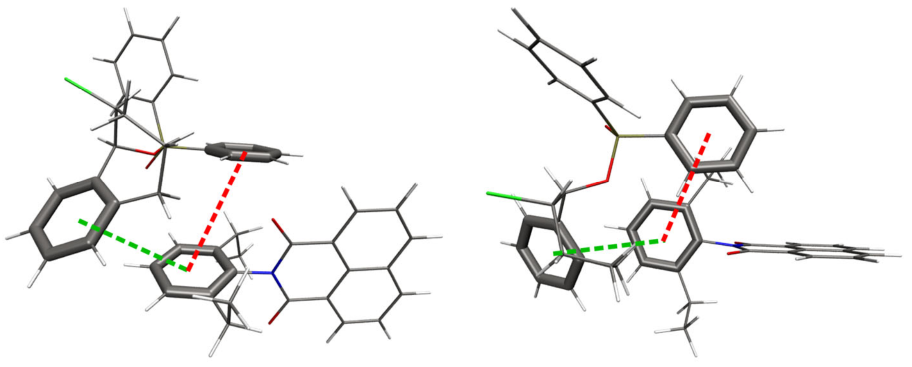

Orientation **2**

Figure S36. Candidate preassemblies of **5d**$^{\bullet-}$**/1d** at the *N*-aniline moiety.

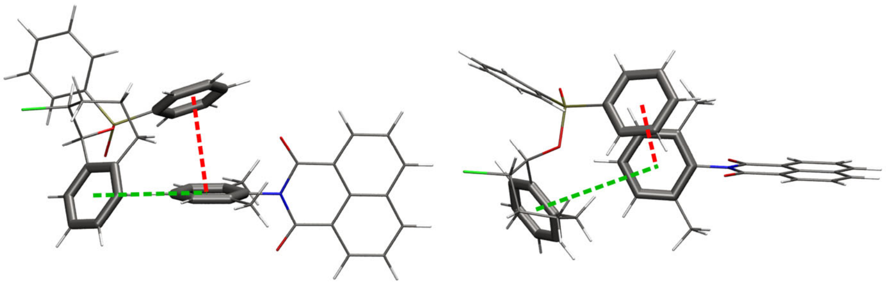

Orientation **1**

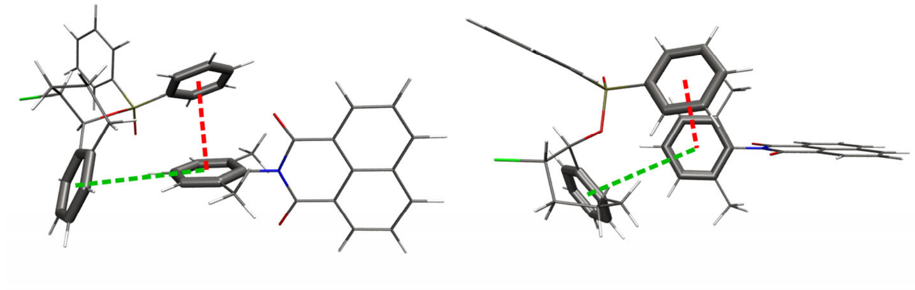

Orientation **2**

Figure S37: Candidate preassemblies of **5e**$^{\bullet-}$**/1d** at the *N*-aniline moiety.

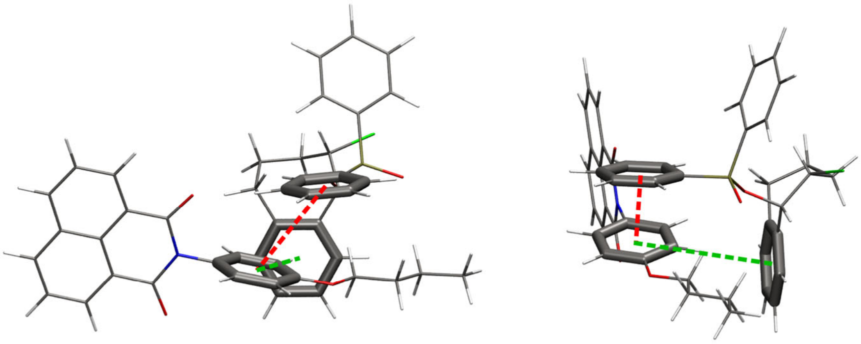

Orientation **1**

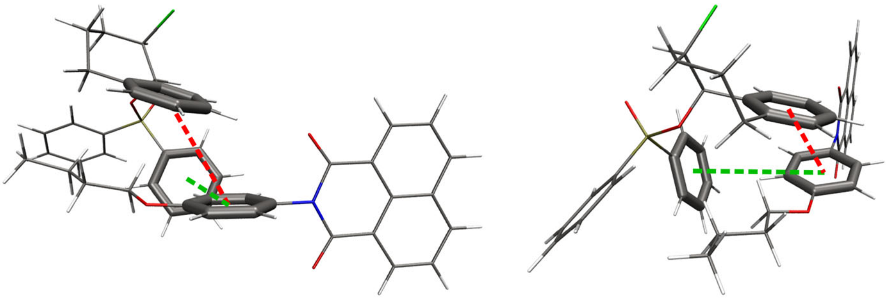

Orientation **2**

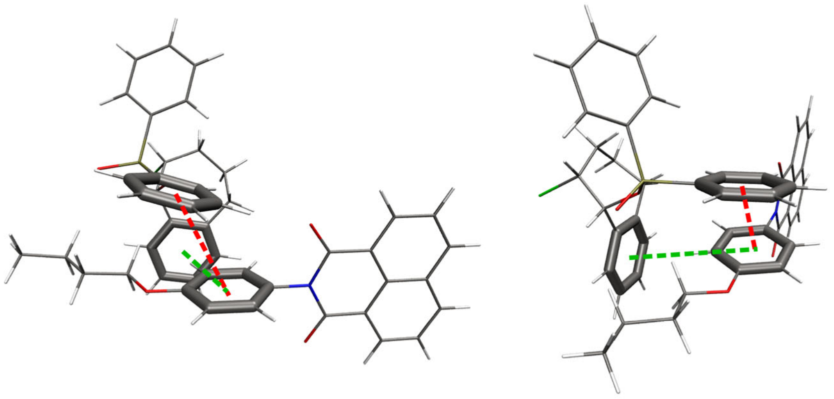

Orientation **3**

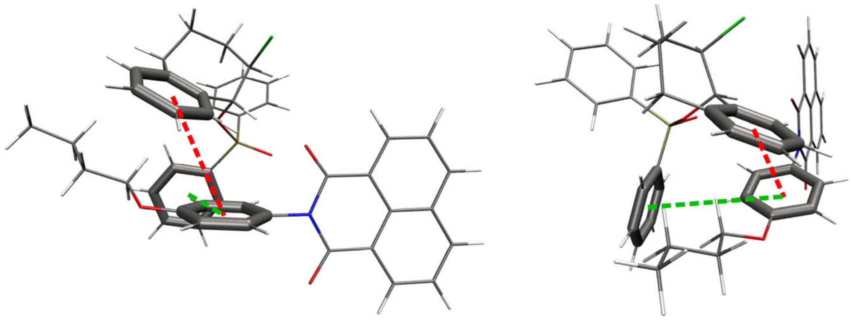

Orientation **4**

Figure S38: Candidate preassemblies of **^{n}BuO-NpMI$^{\bullet-}$/1d** at the *N*-aniline moiety.

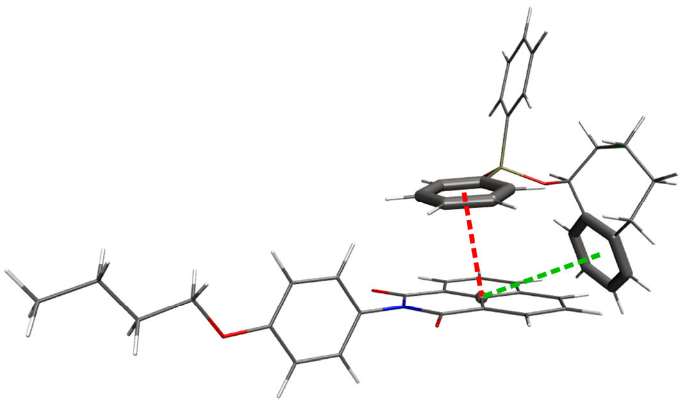

Figure S39. Candidate preassembly of **^{n}BuO-NpMI$^{\bullet-}$/1d** at the naphthalene moiety.

As a quantitative measure for the stability of the converged structures, we defined the free energy of complexation ΔG^{compl} as the free energy difference between the optimized precomplex and its isolated components (Table S9). For the latter, we calculated the neutral form of phosphinate **1d** and the radical anion of the respective catalyst. In all cases, the optimized structures of isolated radical anion catalysts and preassemblies finds the *N*-aniline moiety perpendicular to the naphthalene moiety. Multiple stable structures exist for the preassemblies, differing in the relative orientation of the substrate with respect to the catalyst and the calculations presented here are not intended to be comprehensive. However, comparing the relative energies of a few optimized structures is helpful to

rationalize the observed SAR in the context of a potential preassembly. Regardless of initial input geometries, all optimizations converged to a pincer-like structure where one of the substrate-bound aromatic units coordinates to the side of the aniline and another is oriented parallel to the aniline and coordinates from the top.

Table S9. Calculated free energies of complexation (kcal/mol) for ground state assemblies of substrate **1d** with various residues R on the *ortho*-position of the *N*-aniline unit of the catalyst (ωB97X-D/6-311+G*, IEFPCM(MeCN)).

Components	Orientation	Complexation free energy ΔG^{compl} (kcal mol^{-1})	Intermolecular distance (Å)[a]	Preparative reaction yield (% **2d**)
**NpMI**$^{\cdot-}$ (R=^{i}Pr) / **1d**	**1**	+10.44	4.88 (T-π)[b]<br>4.23 (π-π)[c]	n.d.
	**2**	+3.41	4.86 (T-π)[c]<br>4.48 (π-π)[b]	
	**3**	+5.35	4.98 (T-π)[c]<br>3.84 (π-π)[c]	
	**4**	+11.65	5.65 (T-π)[b]<br>4.41 (π-π)[c]	
**5d**$^{\cdot-}$ (R=Et) / **1d**	**1**	+4.97	4.52[b,d]<br>5.37[c,d]	40
	**2**	+4.66	4.63[b,d]<br>5.23[c,d]	
**5e**$^{\cdot-}$ (R=Me) / **1d**	**1**	+5.88	5.16 (T-π)[b]<br>4.33 (π-π)[c]	55
	**2**	+6.11	5.17 (T-π)[b]<br>4.32 (π-π)[c]	
**^{n}BuO-NpMI**$^{\cdot-}$ (R=H) / **1d**	**1**	+2.59	4.90 (T-π)[b]<br>4.07 (π-π)[c]	75
	**2**	-0.18	5.14 (T-π)[c]<br>4.26 (π-π)[b]	
	**3**	-0.33	4.89 (T-π)[b]<br>4.06 (π-π)[c]	
	**4**	0.70	5.14 (T-π)[c]<br>4.33 (π-π)[b]	
	**Napthalene complex**	-0.16	4.50 (T-π)[e]<br>4.52 (π-π)[f]	

n.d., not determined. [a]The distances between the aromatic centerpoints of each aromatic ring of **1d** to the centerpoint of the *N*-aniline were taken, unless stated otherwise. [b]Interaction between the *N*-aniline and the O-CHR-Ar arene. [c]Interaction between the N-aniline and the O-P(O)Ph arene. [d]The interaction could not be assigned as T-π or π-π and resembled something in between. [e]Distance between the central carbon of the naphthalene moiety and the centerpoint of the O-CHR-Ar arene. [f]Distance between the central carbon of the naphthalene moiety and the centerpoint of the O-P(O)Ph arene.

Out of all the optimized complexes, the four orientational candidates for **^{n}BuO-NpMI** are consistently the most thermodynamically favorable, again supporting the hypothesis that precomplexation in the ground state is an important aspect in the catalytic process. The trend among the other catalysts is not as clear, however. It is difficult to draw a concrete relationship between increasingly bulky *ortho*-substituents and thermodynamics, since the substrate can rearrange to coordinate from a different direction. Nonetheless, formation of all complexes with *ortho*-substituted catalysts is endergonic at this level of theory, indicating that steric hindrance on the aniline unit indeed impedes the stacking interaction and destabilizes the complex.

The spin density in all of the optimized precomplex radical anions, where complexation occurs on the *N*-aniline unit of the catalyst, remains identical to those of the isolated catalysts. In contrast, the spin density of the one precomplex candidate, where the substrate coordinates to the naphthalene moiety of **ⁿBuO-NpMI**, is asymmetrical and thus deviates slightly from that of the isolated catalyst (Figure S40). Such a change in the electronic structure should manifest in a different EPR signal, which we do not observe. This again supports the thesis that if preassociation occurs, it will involve the *N*-aniline moiety rather than the naphthalene moiety of the catalyst.

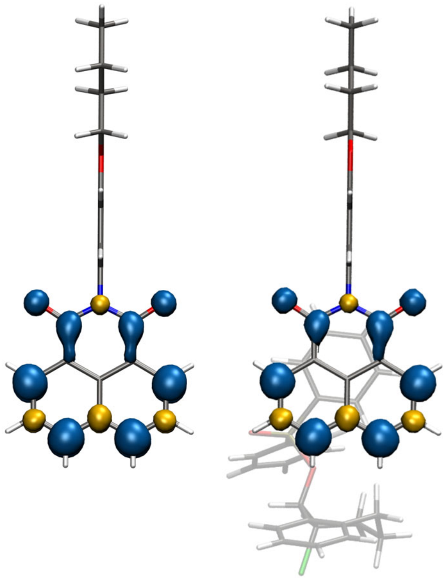

Figure S40. Spin densities of **^{n}BuO-NpMI$^{\bullet-}$** (left) and an **^{n}BuO-NpMI$^{\bullet-}$/1d** precomplex structure where the substrate coordinates to the naphthalene moiety of the catalyst (right).

Note that the mere existence of a ground state minimum does not mean that it is catalytically active upon photoexcitation. For example, Barham and co-workers recently proposed the detection of two different triarylaminium radical cation/haloarene precomplex geometries (edge-to-face *T*-π and face-to-face π-π) on the basis of changes in EPR spectra and DFT calculations.[2] There, it was proposed the face-to-face π-π geometry was ‘unreactive’ upon photoexcitation.

Therefore, the thermodynamics of precomplexation in the ground state is only one aspect to consider in a larger scheme. Our computational results demonstrate that there indeed are stable candidate preassemblies of phosphinate substrate and radical anion catalyst. Moreover, we show that the introduction of substituents at the *ortho*-position of the *N*-aniline moiety destabilizes these complexes and makes their formation less likely due to a higher kinetic barrier upon approach of the two molecules. Further studies are required to investigate the excited states of our optimized candidate structures and whether certain geometric factors can facilitate SET within preassemblies upon photoexcitation.

19. XYZ Co-ordinates of Computed Structures

19.1. e-PRC Catalyst for CASSCF calculation

^{n}BuO-NpMI$^{\bullet-}$ model system in C_{2v} symmetry

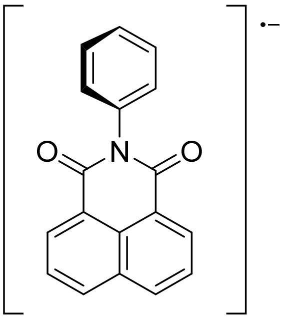

32 atoms

(No G available)

(No spin density available)

Charge = -1; Multiplicity = 2

atom	x [Å]	y [Å]	z [Å]
C	0.000000	-1.241770	4.016662
C	0.000000	-0.000000	3.339850
C	-0.000000	1.241770	4.016662
C	0.000000	-2.443795	3.310524
C	0.000000	-0.000000	1.902897
C	0.000000	-1.234646	1.208833
C	0.000000	-2.452509	1.925975
C	0.000000	-1.251034	-0.232985
C	-0.000000	1.251034	-0.232985
C	-0.000000	1.234646	1.208833
C	-0.000000	2.452509	1.925975
C	-0.000000	2.443795	3.310524
H	0.000000	3.383801	3.854528
H	0.000000	3.385695	1.375045
H	0.000000	-3.383801	3.854528
H	0.000000	-3.385695	1.375045
H	0.000000	1.246707	5.103025
H	0.000000	-1.246707	5.103025
O	0.000000	-2.283619	-0.918319
O	-0.000000	2.283619	-0.918319
N	0.000000	-0.000000	-0.870165
C	0.000000	-0.000000	-2.306403
C	-1.198488	0.000000	-3.001653
C	1.198488	0.000000	-3.001653
C	-1.205152	0.000000	-4.385360
C	1.205152	0.000000	-4.385360
C	0.000000	0.000000	-5.094558
H	-2.139003	0.000000	-4.936881
H	2.139003	0.000000	-4.936881
H	2.138721	-0.000000	-2.462143
H	-2.138721	-0.000000	-2.462143
H	0.000000	0.000000	-6.161170

19.2. e-PRC Catalysts for spin density calculations

^{n}BuO-NpMI$^{\bullet-}$

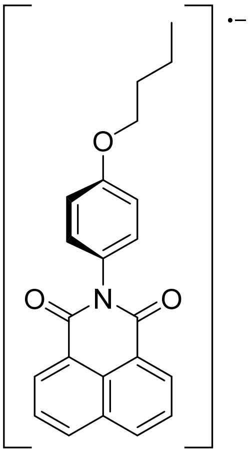

45 atoms

G = -1129.959949 E_h

Charge = -1; Multiplicity = 2

atom	x [Å]	y [Å]	z [Å]
C	5.937055	-1.241857	-0.296906
C	5.262949	-0.000098	-0.236814
C	5.937157	1.241610	-0.296922
C	5.233885	-2.443863	-0.231855
C	3.831743	-0.000040	-0.108425
C	3.140501	-1.234658	-0.045529
C	3.854939	-2.452540	-0.107425
C	1.704468	-1.251007	0.083499
C	1.704560	1.251099	0.083263
C	3.140596	1.234633	-0.045599
C	3.855135	2.452460	-0.107493
C	5.234082	2.443671	-0.231879
H	5.775972	3.383718	-0.279339
H	3.306574	3.385679	-0.055931
H	5.775702	-3.383951	-0.279330
H	3.306302	-3.385714	-0.055857
H	7.019108	1.246591	-0.394330
H	7.019004	-1.246925	-0.394318
O	1.022261	-2.283574	0.149329
O	1.022392	2.283700	0.148801
N	1.069345	0.000075	0.134610
C	-0.360997	0.000162	0.264625
C	-0.945260	0.000383	1.526972
C	-1.164765	-0.000060	-0.861271
C	-2.322779	0.000396	1.657853
C	-2.553095	-0.000138	-0.745139
C	-3.137324	0.000094	0.521244
H	-2.788221	0.000568	2.637453
H	-3.155683	-0.000406	-1.644306
H	-0.707579	-0.000246	-1.845200
H	-0.313829	0.000545	2.409218
O	-4.474078	0.000021	0.750039
C	-5.357577	-0.000265	-0.365787
C	-6.778331	-0.000140	0.158341
H	-5.172482	-0.888969	-0.981447
H	-5.172500	0.888098	-0.981944
C	-7.805052	0.000213	-0.972341
H	-6.924465	-0.880869	0.793575
H	-6.924166	0.880410	0.793890
C	-9.239901	-0.000138	-0.454608
H	-7.646155	0.877697	-1.610026
H	-7.645998	-0.876722	-1.610747
H	-9.959700	0.000158	-1.277375
H	-9.436836	-0.883741	0.160394
H	-9.436994	0.882892	0.161168

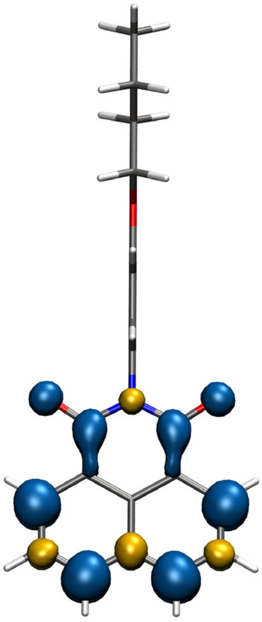

5d$^{\bullet-}$ (R=Et)

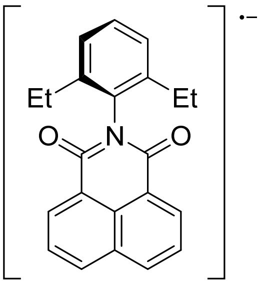

44 atoms

G = -1054.756960 E_h

Charge = -1; Multiplicity = 2

atom	x [Å]	y [Å]	z [Å]
C	-3.850715	0.110669	2.441121
C	-4.556937	0.056337	1.240484
C	-3.880187	-0.000002	-0.000037
C	-2.443451	0.000006	0.000021
C	-1.749267	0.054797	1.233306
C	-2.466102	0.110046	2.449827
H	-5.643160	-0.057012	-1.245879
H	-4.394588	0.153814	3.380231
H	-5.643260	0.056990	1.245664
C	-4.556837	-0.056348	-1.240612
C	-1.749168	-0.054777	-1.233209
H	-1.914800	0.152008	3.381891
C	-2.465905	-0.110032	-2.449787
C	-3.850518	-0.110671	-2.441192
H	-1.914528	-0.151989	-3.381807
H	-4.394316	-0.153822	-3.380346
O	0.379863	-0.095677	-2.278798
O	0.379679	0.095726	2.279066
C	-0.308094	-0.053589	-1.247738
C	-0.308195	0.053624	1.247951
N	0.327839	0.000016	0.000132
C	1.768476	0.000014	0.000191
C	2.435864	-1.224559	0.068579
C	2.435873	1.224585	-0.068139
C	3.829935	-1.204203	0.067912
C	3.829943	1.204226	-0.067346
C	4.519552	0.000011	0.000314
H	4.388389	-2.132055	0.120201
H	4.388405	2.132077	-0.119584
H	5.605100	0.000010	0.000363
C	1.634308	2.505942	-0.140061
H	0.966433	2.446359	-1.006399
H	0.973691	2.549470	0.733000
C	1.634289	-2.505914	0.140430
H	0.966327	-2.446322	1.006700
H	0.973759	-2.549446	-0.732697
C	2.443015	3.794795	-0.220653
H	3.078457	3.821706	-1.110473
H	1.770024	4.654018	-0.270999
H	3.084004	3.929677	0.655210
C	2.442986	-3.794767	0.221115
H	1.769988	-4.653988	0.271403
H	3.084061	-3.929660	-0.654683
H	3.078339	-3.821669	1.110998

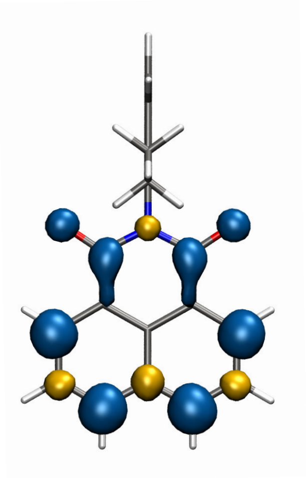

5e$^{•-}$ (R=Me)

38 atoms

G = -976.192957 E_h

Charge = -1; Multiplicity = 2

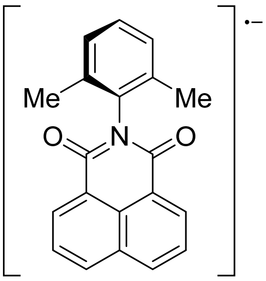

atom	x [Å]	y [Å]	z [Å]
C	-3.906754	0.120708	2.440648
C	-4.612823	0.061706	1.240159
C	-3.935996	0.000006	-0.000066
C	-2.499229	0.000005	-0.000041
C	-1.805175	0.058692	1.233281
C	-2.522159	0.119081	2.449519
H	-5.699097	-0.062850	-1.245377
H	-4.450711	0.168257	3.379487
H	-5.699140	0.062866	1.245184
C	-4.612780	-0.061692	-1.240314
C	-1.805133	-0.058683	-1.233340
H	-1.971008	0.164392	3.381515
C	-2.522075	-0.119068	-2.449602
C	-3.906670	-0.120694	-2.440778
H	-1.970892	-0.164380	-3.381579
H	-4.450595	-0.168241	-3.379636
O	0.324517	-0.099731	-2.278684
O	0.324439	0.099750	2.278699
C	-0.364011	-0.056165	-1.248243
C	-0.364054	0.056171	1.248234
N	0.271579	0.000002	0.000007
C	1.711035	0.000001	0.000032
C	2.382595	-1.220264	0.071461
C	2.382599	1.220266	-0.071372
C	3.776635	-1.202220	0.070887
C	3.776639	1.202220	-0.070743
C	4.470077	0.000000	0.000086
H	4.322355	-2.139527	0.126271
H	4.322362	2.139527	-0.126104
H	5.555456	-0.000001	0.000108
C	1.614549	2.511189	-0.147868
H	0.973547	2.536224	-1.033579
H	0.965903	2.637455	0.723610
H	2.290015	3.367143	-0.194259
C	1.614541	-2.511186	0.147930
H	0.973503	-2.536218	1.033615
H	0.965929	-2.637454	-0.723574
H	2.290004	-3.367141	0.194349

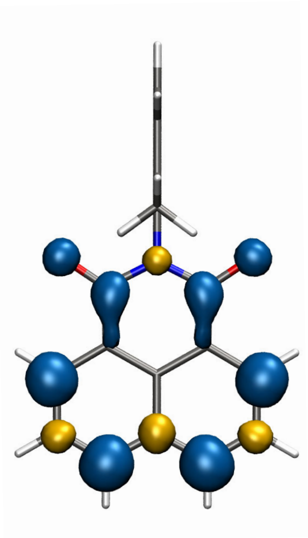

NpMI$^{\bullet-}$

50 atoms

G = -1133.328344 E_h

Charge = -1; Multiplicity = 2

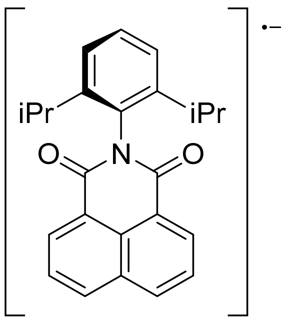

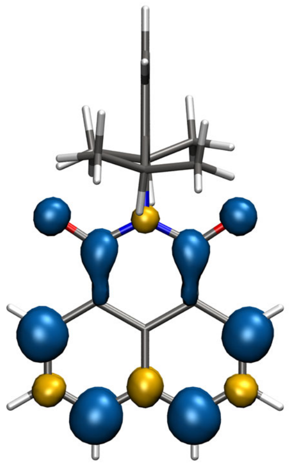

atom	x [Å]	y [Å]	z [Å]
C	-3.917443	0.138180	2.439745
C	-4.623775	0.070358	1.239782
C	-3.947116	0.000013	-0.000046
C	-2.510268	0.000002	-0.000030
C	-1.816105	0.069297	1.232443
C	-2.532891	0.137876	2.448302
H	-5.710083	-0.070869	-1.245133
H	-4.461257	0.191602	3.378373
H	-5.710109	0.070928	1.245002
C	-4.623749	-0.070318	-1.239889
C	-1.816079	-0.069302	-1.232489
H	-1.981369	0.189950	3.379713
C	-2.532840	-0.137862	-2.448363
C	-3.917392	-0.138146	-2.439836
H	-1.981298	-0.189940	-3.379763
H	-4.461186	-0.191556	-3.378477
O	0.312035	-0.118045	-2.278389
O	0.311987	0.118058	2.278388
C	-0.374637	-0.067550	-1.247752
C	-0.374664	0.067522	1.247738
N	0.260983	-0.000012	-0.000000
C	1.701082	-0.000006	0.000017
C	2.376471	-1.222811	0.069055
C	2.376462	1.222804	-0.068997
C	3.770633	-1.200279	0.062544
C	3.770624	1.200285	-0.062437
C	4.464607	0.000006	0.000066
H	4.324084	-2.133275	0.106768
H	4.324068	2.133287	-0.106640
H	5.550120	0.000011	0.000086
C	1.635943	2.546924	-0.128000
C	2.061690	3.389959	-1.333984
C	1.808353	3.326328	1.180644
H	0.570608	2.334184	-0.242681
H	1.942461	2.835059	-2.268052
H	1.450529	4.295117	-1.396956
H	3.106999	3.705115	-1.259967
H	1.471977	2.733199	2.034284
H	2.856985	3.595553	1.344599
H	1.226303	4.252616	1.155252
C	1.635963	-2.546938	0.128039
C	2.061655	-3.389950	1.334059
C	1.808452	-3.326361	-1.180583
H	0.570619	-2.334208	0.242660
H	1.942369	-2.835036	2.268112
H	1.450500	-4.295113	1.397012
H	3.106971	-3.705095	1.260103
H	1.472120	-2.733247	-2.034251
H	2.857095	-3.595581	-1.344475
H	1.226407	-4.252653	-1.155210

19.3. Phosphinate radical anions for BDFE and redox potential calculations

1a$^{\bullet-}$

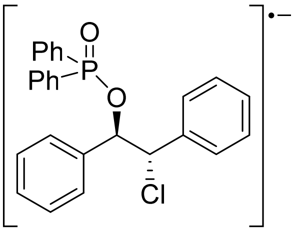

52 atoms

G = -1955.683217

Charge = -1; Multiplicity = 2

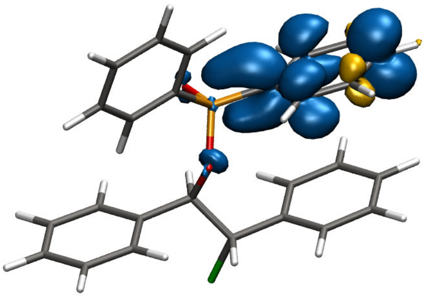

atom	x [Å]	y [Å]	z [Å]
P	-0.173548	1.148976	0.739393
C	0.901446	2.491857	0.123296
C	0.401014	3.790198	0.010292
C	2.258714	2.265610	-0.123630
C	1.237284	4.842173	-0.351548
H	-0.651674	3.973649	0.200253
C	3.096708	3.316536	-0.477436
H	2.664362	1.262511	-0.045574
C	2.586544	4.606978	-0.594567
H	0.834292	5.845592	-0.442885
H	4.148756	3.127835	-0.665922
H	3.240033	5.426850	-0.874883
C	-1.816769	1.520277	0.373950
C	-2.227771	1.925452	-0.955956
C	-2.864243	1.321547	1.344856
C	-3.553012	2.147567	-1.245493
H	-1.480630	2.062584	-1.734239
C	-4.177388	1.556089	1.030668
H	-2.599441	0.984659	2.343093
C	-4.566463	1.979144	-0.272242
H	-3.825899	2.457178	-2.252717
H	-4.937972	1.403014	1.793609
H	-5.609377	2.153403	-0.515708
O	0.122105	0.780558	2.172075
O	0.387025	-0.066136	-0.266778
C	0.853416	-1.311376	0.235279
H	0.588520	-1.405369	1.291460
C	0.113195	-2.392168	-0.566817
H	0.399738	-2.327834	-1.615757
Cl	0.716009	-4.034025	-0.029528
C	-1.382788	-2.324355	-0.436447
C	-2.163847	-2.162180	-1.577292
C	-2.005836	-2.397374	0.810069
C	-3.549465	-2.068583	-1.477149
H	-1.688477	-2.098795	-2.551611
C	-3.386650	-2.312104	0.910795
H	-1.411734	-2.527177	1.709483
C	-4.163063	-2.149165	-0.234089
H	-4.146444	-1.933736	-2.373059
H	-3.859966	-2.366944	1.885333
H	-5.242420	-2.074614	-0.153265
C	2.358546	-1.374301	0.098403
C	3.160686	-1.524101	1.225241
C	2.963950	-1.220088	-1.149587
C	4.548695	-1.524601	1.112111
H	2.698692	-1.634661	2.201736
C	4.347521	-1.218749	-1.266740

atom	x [Å]	y [Å]	z [Å]
H	2.351922	-1.075767	-2.034589
C	5.144705	-1.371582	-0.134118
H	5.162433	-1.639344	1.999515
H	4.806640	-1.093453	-2.241869
H	6.225930	-1.367366	-0.224776

1d•−

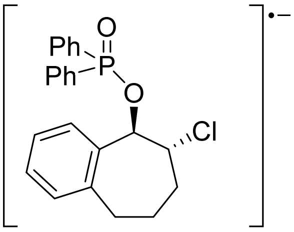

49 atoms

G = -1841.409053 E_h

Charge = -1; Multiplicity = 2

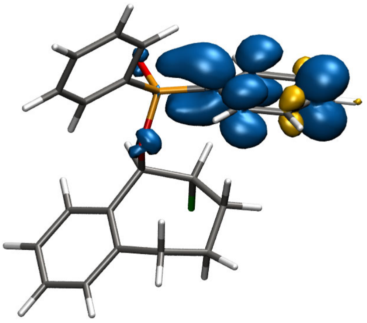

atom	x [Å]	y [Å]	z [Å]
P	1.388613	0.158986	-0.956577
C	2.211355	1.647197	-0.301966
C	3.596327	1.635448	-0.122491
C	1.506061	2.831272	-0.070672
C	4.265663	2.784421	0.284956
H	4.149899	0.717293	-0.293509
C	2.175478	3.981009	0.333730
H	0.429656	2.847943	-0.198603
C	3.555660	3.959166	0.512823
H	5.341231	2.761900	0.426313
H	1.618308	4.894725	0.513435
H	4.076714	4.856247	0.831294
C	2.132338	-1.248030	-0.285324
C	2.469304	-1.322186	1.122375
C	2.155605	-2.491302	-1.021266
C	2.826142	-2.514459	1.702811
H	2.443225	-0.420484	1.729765
C	2.508631	-3.669910	-0.413888
H	1.894977	-2.485092	-2.076402
C	2.859212	-3.722297	0.962902
H	3.085538	-2.527864	2.759630
H	2.524976	-4.583073	-1.005381
H	3.133972	-4.659790	1.434552
O	-0.098411	0.449487	-0.256186
O	1.226012	0.153580	-2.457093
C	-1.286310	-0.127020	-0.790332
C	-1.314767	-1.638616	-0.487678
H	-1.256521	-0.032069	-1.879711
C	-1.122263	-2.072114	0.953944
H	-0.541885	-2.115184	-1.091158
C	-2.090483	-1.513014	1.990998
H	-0.099707	-1.784837	1.220417
H	-1.143788	-3.165817	0.978434
C	-2.036082	0.012426	2.145307
H	-3.116673	-1.825141	1.767190
H	-1.834889	-1.967741	2.953161
H	-2.453391	0.284999	3.118308
H	-0.989330	0.333215	2.151701
Cl	-2.868792	-2.317735	-1.177528
C	-2.792691	0.767615	1.077589
C	-2.460325	0.683427	-0.284223
C	-3.874370	1.567188	1.447296

atom	x [Å]	y [Å]	z [Å]
H	-4.141720	1.637080	2.497972
C	-4.618541	2.274533	0.508034
H	-5.454756	2.887185	0.829317
C	-4.284196	2.189756	-0.836075
H	-4.852127	2.734774	-1.582739
C	-3.207191	1.397496	-1.219006
H	-2.941583	1.331854	-2.270196

1g•–

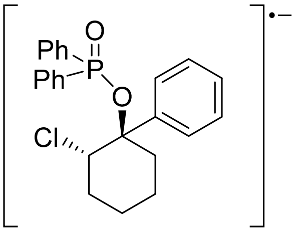

52 atoms

G = -1880.689430 E_h

Charge = -1; Multiplicity = 2

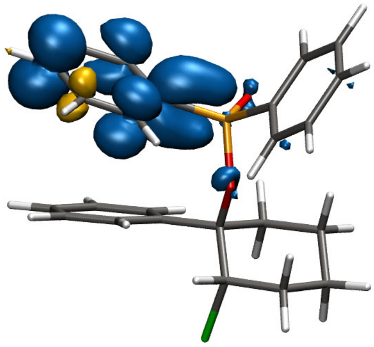

atom	x [Å]	y [Å]	z [Å]
P	-1.093200	0.340403	0.769861
C	-0.906461	1.945217	0.149543
C	-0.557007	3.045521	1.009185
C	-1.057678	2.240106	-1.257848
C	-0.400376	4.315143	0.510690
H	-0.412220	2.860999	2.070017
C	-0.900401	3.521700	-1.731253
H	-1.295757	1.442157	-1.956578
C	-0.573511	4.596690	-0.872606
H	-0.135288	5.120566	1.192625
H	-1.024854	3.707336	-2.796553
H	-0.444167	5.602087	-1.259440
C	-2.678190	-0.354139	0.204339
C	-2.871064	-0.851098	-1.089332
C	-3.767537	-0.314162	1.080618
C	-4.126808	-1.290079	-1.496848
H	-2.032715	-0.913190	-1.774249
C	-5.019550	-0.760931	0.675437
H	-3.629191	0.063940	2.088924
C	-5.204032	-1.247305	-0.617125
H	-4.261972	-1.675112	-2.502741
H	-5.853317	-0.732158	1.369777
H	-6.181785	-1.594771	-0.934755
O	-0.152774	-0.716810	-0.091548
O	-0.923604	0.212201	2.260804
C	2.097325	0.174946	0.186928
C	2.094674	1.027647	-0.919821
C	2.919520	0.493661	1.264572
C	2.901883	2.154219	-0.957203
H	1.429861	0.829337	-1.753267
C	3.728304	1.628227	1.234627
H	2.944314	-0.138481	2.143962
C	3.727061	2.459631	0.122717
H	2.876520	2.804412	-1.825742
H	4.360705	1.857259	2.086390
H	4.357775	3.342362	0.097507
C	1.214280	-1.072313	0.168110
C	1.524705	-1.957157	-1.068410
C	1.291036	-1.917686	1.447211
C	0.671090	-3.217306	-1.119953

atom	x [Å]	y [Å]	z [Å]
H	1.385231	-1.368284	-1.972728
C	0.389291	-3.149887	1.380042
H	2.327812	-2.236305	1.594722
H	1.008347	-1.295776	2.297176
C	0.722780	-4.025801	0.173951
H	0.972397	-3.821181	-1.979748
H	-0.355617	-2.886655	-1.305239
H	0.499635	-3.723730	2.305098
H	-0.657361	-2.831935	1.329064
H	0.025183	-4.865621	0.105424
H	1.722359	-4.458058	0.299198
Cl	3.298067	-2.401613	-1.099191

1o•−

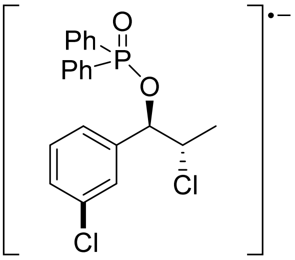

45 atoms

G = -2223.647199 E_h

Charge = -1; Multiplicity = 2

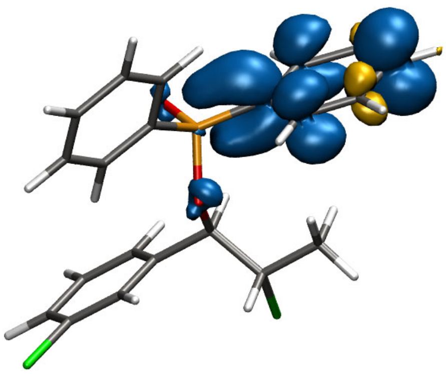

atom	x [Å]	y [Å]	z [Å]
P	1.308097	0.398273	-0.777787
C	1.326550	2.121771	-0.188126
C	2.529385	2.825374	-0.112812
C	0.128353	2.790592	0.079639
C	2.537846	4.172353	0.236468
H	3.463131	2.312913	-0.321256
C	0.135787	4.137456	0.422389
H	-0.812488	2.253346	0.023389
C	1.341422	4.829591	0.503875
H	3.479281	4.708230	0.299025
H	-0.799518	4.647602	0.628756
H	1.347210	5.880404	0.774878
C	2.800832	-0.383783	-0.414237
C	3.429090	-0.254682	0.886699
C	3.367357	-1.361065	-1.312667
C	4.532693	-1.004703	1.214295
H	3.026220	0.446584	1.613601
C	4.466996	-2.099524	-0.957490
H	2.913707	-1.504074	-2.289636
C	5.089920	-1.943012	0.313430
H	4.984516	-0.873195	2.195669
H	4.870940	-2.818792	-1.666929
H	5.957591	-2.533849	0.587252
O	0.099970	-0.118793	0.262778
O	0.829116	0.257886	-2.201077
C	-0.651755	-1.278717	-0.062739
H	-0.199685	-1.806929	-0.909834
C	-0.584728	-2.195199	1.167730
H	-1.055445	-1.698427	2.016973
Cl	-1.623471	-3.666050	0.861041
C	0.821259	-2.642633	1.504375
H	1.280223	-3.162944	0.659625
H	1.439647	-1.773652	1.733395
H	0.816354	-3.307669	2.369574
C	-2.058460	-0.859895	-0.435037

atom	x [Å]	y [Å]	z [Å]
C	-2.680751	-1.354253	-1.576066
C	-2.735162	0.049968	0.375789
C	-3.970665	-0.951401	-1.905203
H	-2.154643	-2.056246	-2.214694
C	-4.017786	0.443157	0.031536
H	-2.256917	0.458400	1.258701
C	-4.653367	-0.046078	-1.102136
H	-4.449285	-1.339084	-2.797762
H	-5.656183	0.277281	-1.354688
Cl	-4.857569	1.597306	1.053599

1r•−

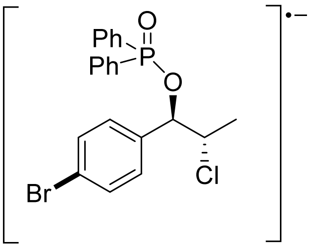

45 atoms

G = -4337.614007 E_h

Charge = -1; Multiplicity = 2

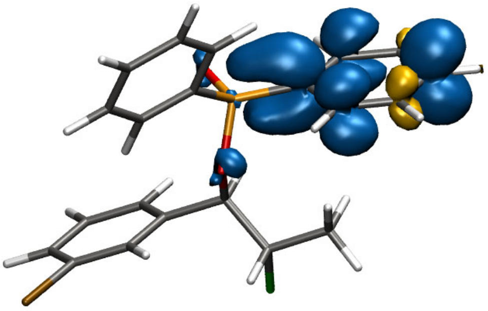

atom	x [Å]	y [Å]	z [Å]
P	-1.824355	0.492612	0.713618
C	-1.970602	2.166675	0.011217
C	-3.174042	2.866124	0.114465
C	-0.852245	2.811291	-0.525431
C	-3.263240	4.184610	-0.320950
H	-4.046073	2.372514	0.531442
C	-0.939515	4.130230	-0.955309
H	0.087620	2.276313	-0.610719
C	-2.146086	4.818064	-0.855522
H	-4.205369	4.717039	-0.241686
H	-0.065881	4.621980	-1.370716
H	-2.214418	5.846982	-1.193652
C	-3.343511	-0.321206	0.654134
C	-4.180579	-0.278424	-0.529897
C	-3.729924	-1.254683	1.684991
C	-5.300017	-1.068266	-0.631621
H	-3.923331	0.389146	-1.348842
C	-4.850694	-2.034095	1.555163
H	-3.121162	-1.332096	2.581669
C	-5.675220	-1.965659	0.396323
H	-5.909919	-1.001154	-1.530578
H	-5.113445	-2.717818	2.359763
H	-6.557858	-2.589032	0.300159
O	-0.790157	-0.096417	-0.466283
O	-1.118035	0.461998	2.046224
C	0.005464	-1.237734	-0.176842
H	-0.316245	-1.702251	0.762246
C	-0.242647	-2.238297	-1.314532
H	0.096089	-1.809650	-2.258431
Cl	0.833700	-3.692034	-1.056889
C	-1.682001	-2.696621	-1.405251
H	-2.010835	-3.145802	-0.464362
H	-2.328504	-1.841097	-1.605905
H	-1.804361	-3.425185	-2.208439
C	1.449654	-0.806660	-0.046324
C	2.212211	-1.182890	1.053436
C	2.030447	-0.005972	-1.028486

atom	x [Å]	y [Å]	z [Å]
C	3.538025	-0.779915	1.177386
H	1.771939	-1.798025	1.831697
C	3.351787	0.407371	-0.923087
H	1.444200	0.311853	-1.884449
C	4.093930	0.012000	0.183377
H	4.121727	-1.077628	2.040303
H	3.793583	1.030338	-1.691738
Br	5.907801	0.576248	0.340632

1aa•–

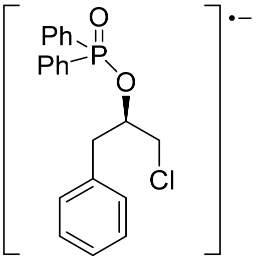

45 atoms

G = -1764.026754

Charge = -1; Multiplicity = 2

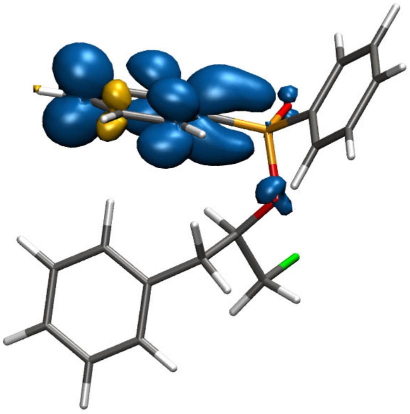

atom	x [Å]	y [Å]	z [Å]
P	-1.514239	-0.211597	0.830534
C	-0.733453	1.335227	0.825519
C	-0.017846	1.792639	1.994394
C	-0.648038	2.173314	-0.353597
C	0.696804	2.963615	1.979295
H	-0.052989	1.194799	2.900835
C	0.072593	3.343108	-0.338975
H	-1.177506	1.889928	-1.259526
C	0.771794	3.774747	0.813846
H	1.220653	3.272422	2.881492
H	0.104215	3.950050	-1.241656
H	1.347826	4.693872	0.807393
C	-2.993137	-0.172205	-0.223505
C	-2.920251	-0.235595	-1.617481
C	-4.239231	0.001131	0.384725
C	-4.072429	-0.119599	-2.388600
H	-1.961275	-0.389260	-2.100551
C	-5.390945	0.110065	-0.385574
H	-4.305621	0.046470	1.467142
C	-5.308872	0.053082	-1.774657
H	-4.004697	-0.170454	-3.470494
H	-6.353978	0.238667	0.097692
H	-6.207562	0.140153	-2.376577
O	-1.792173	-0.770575	2.201677
O	-0.647525	-1.306995	-0.083647
C	0.773230	-1.294959	-0.029596
H	1.111920	-0.768930	0.869756
C	1.278721	-2.722502	-0.001741
H	2.362667	-2.744940	-0.098732
H	0.827417	-3.307375	-0.801799
C	1.337437	-0.574262	-1.265751
H	1.223237	-1.214689	-2.146714
H	0.720979	0.312380	-1.425485
Cl	0.893147	-3.577973	1.541351
C	2.772609	-0.152398	-1.082700
C	3.825504	-0.843796	-1.680523
C	3.064877	0.947631	-0.271050
C	5.145079	-0.449209	-1.473871
H	3.613363	-1.698437	-2.317354

atom	x [Å]	y [Å]	z [Å]
C	4.380763	1.343681	-0.063438
H	2.252426	1.500582	0.193997
C	5.426278	0.645418	-0.663528
H	5.952730	-0.997564	-1.948240
H	4.591183	2.202411	0.566555
H	6.453754	0.955181	-0.502094

1ai$^{\bullet-}$

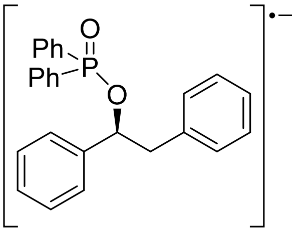

52 atoms

G = -1496.063330 E_h

Charge = -1; Multiplicity = 2

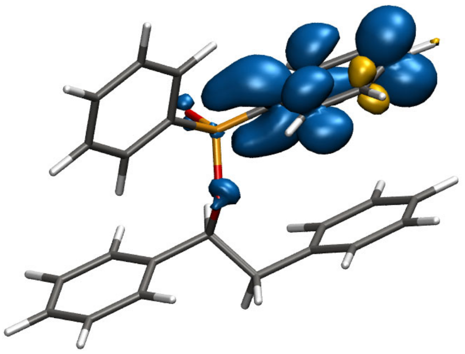

atom	x [Å]	y [Å]	z [Å]
P	0.006152	0.892913	0.726428
C	1.206412	2.140801	0.141624
C	0.862845	3.494057	0.129031
C	2.515443	1.772714	-0.181755
C	1.804467	4.460663	-0.210692
H	-0.151401	3.788824	0.378346
C	3.459550	2.737637	-0.513545
H	2.798150	0.725478	-0.180908
C	3.104775	4.083898	-0.531234
H	1.522198	5.508437	-0.224330
H	4.472693	2.438355	-0.762598
H	3.840443	4.837083	-0.794600
C	-1.594354	1.460865	0.413151
C	-1.972671	2.022632	-0.868471
C	-2.659125	1.244215	1.363348
C	-3.274954	2.374650	-1.128073
H	-1.216457	2.176238	-1.634833
C	-3.950803	1.607467	1.079824
H	-2.423775	0.796174	2.324893
C	-4.303110	2.190468	-0.170565
H	-3.519700	2.803504	-2.098208
H	-4.722218	1.439183	1.828721
H	-5.328999	2.466708	-0.390581
O	0.272264	0.450274	2.144900
O	0.415124	-0.334141	-0.320329
C	0.691991	-1.660920	0.130251
H	0.375092	-1.755451	1.172260
C	-0.104632	-2.647672	-0.728013
H	0.178007	-2.514363	-1.776475
C	-1.596000	-2.500851	-0.566210
C	-2.381581	-1.944410	-1.573986
C	-2.220736	-2.914651	0.612291
C	-3.757125	-1.803466	-1.412706
H	-1.911406	-1.610234	-2.494264
C	-3.594310	-2.779502	0.778208
H	-1.625095	-3.353338	1.408720
C	-4.368811	-2.225250	-0.237379
H	-4.350645	-1.362011	-2.207005
H	-4.061693	-3.109276	1.700663
H	-5.440836	-2.116128	-0.109330
C	2.180972	-1.922407	0.057465

atom	x [Å]	y [Å]	z [Å]
C	2.886199	-2.311193	1.193081
C	2.873008	-1.762762	-1.144892
C	4.259039	-2.540026	1.133747
H	2.360483	-2.427844	2.136447
C	4.242406	-1.986175	-1.207911
H	2.339798	-1.439372	-2.033589
C	4.940295	-2.376712	-0.066709
H	4.795702	-2.838633	2.028465
H	4.768743	-1.852521	-2.147538
H	6.010558	-2.549080	-0.114741
H	0.203832	-3.658903	-0.441801

1al•–

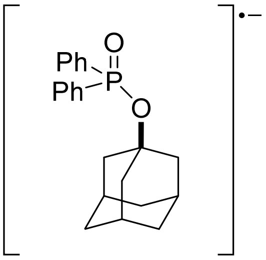

50 atoms

G = -1344.893594 E_h

Charge = -1; Multiplicity = 2

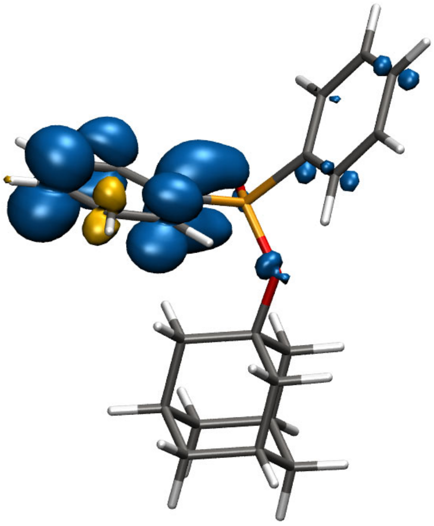

atom	x [Å]	y [Å]	z [Å]
P	1.076634	0.097909	0.795447
C	1.227128	-1.535914	0.229189
C	1.392514	-2.635322	1.140172
C	1.187618	-1.857281	-1.180289
C	1.525863	-3.924647	0.685786
H	1.404190	-2.436971	2.208498
C	1.325419	-3.156805	-1.611781
H	1.024789	-1.067974	-1.909578
C	1.506737	-4.226639	-0.705082
H	1.645560	-4.729619	1.408135
H	1.284629	-3.362967	-2.679872
H	1.607210	-5.247900	-1.057403
C	2.475650	1.090596	0.184667
C	2.584775	1.485833	-1.154775
C	3.539337	1.362116	1.052973
C	3.729071	2.131021	-1.611133
H	1.763219	1.302986	-1.838712
C	4.680668	2.011009	0.596642
H	3.466551	1.065390	2.094605
C	4.781397	2.395568	-0.738732
H	3.796789	2.435025	-2.651153
H	5.493079	2.221014	1.285381
H	5.672478	2.901890	-1.095570
O	0.929150	0.235241	2.289407
O	-0.075643	0.854413	-0.082026
C	-1.499344	0.633217	-0.046117
C	-2.121952	1.602219	0.964898
C	-2.006059	0.966126	-1.451326
C	-1.903861	-0.804458	0.306633
H	-1.808705	2.622969	0.718703
H	-1.740319	1.373635	1.965449
C	-3.653166	1.483655	0.925510
H	-1.541362	0.280858	-2.169206
H	-1.689700	1.981819	-1.713535
C	-3.536202	0.848310	-1.500255
H	-1.534303	-1.066380	1.303404

atom	x [Å]	y [Å]	z [Å]
H	-1.447127	-1.502514	-0.400456
C	-3.436884	-0.925026	0.266858
H	-4.087621	2.181084	1.649355
C	-4.153964	1.822806	-0.486243
C	-4.057747	0.045927	1.280767
H	-3.885038	1.093182	-2.508934
C	-3.942419	-0.588072	-1.142731
H	-3.714673	-1.952962	0.522191
H	-3.884652	2.854066	-0.743289
H	-5.247716	1.757902	-0.522520
H	-3.718805	-0.200708	2.293825
H	-5.149801	-0.050635	1.274278
H	-5.032735	-0.693633	-1.187038
H	-3.520968	-1.290861	-1.871099

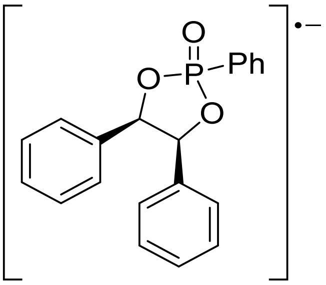

4a•–

41 atoms

G = -1339.167055 E_h

Charge = -1; Multiplicity = 2

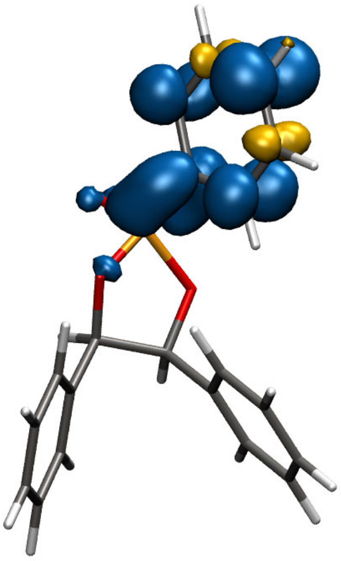

atom	x [Å]	y [Å]	z [Å]
P	-1.693719	-1.025324	-0.930579
O	-2.117062	-2.170693	-1.797757
O	-0.779277	0.098718	-1.722959
O	-0.407184	-1.484813	0.027973
C	-2.915249	-0.225937	-0.046255
C	-4.217568	-0.837055	0.117316
C	-2.701095	1.041264	0.624912
C	-5.184553	-0.244030	0.887118
H	-4.426613	-1.778047	-0.382894
C	-3.692652	1.609376	1.386720
H	-1.759566	1.565081	0.498497
C	-4.953889	0.989579	1.553908
H	-6.151777	-0.732297	0.984054
H	-3.500657	2.567499	1.865382
H	-5.726019	1.448689	2.161809
C	0.635837	-0.044846	-1.535051
H	1.086610	-0.180779	-2.520332
C	0.793263	-1.369989	-0.720504
H	0.832196	-2.193248	-1.445176
C	2.013871	-1.445018	0.157307
C	1.917115	-1.520063	1.542480
C	3.274982	-1.426603	-0.439438
C	3.067978	-1.565704	2.324594
H	0.938549	-1.537019	2.007585
C	4.423707	-1.466391	0.340414
H	3.361753	-1.376226	-1.521413
C	4.323195	-1.534474	1.727906
H	2.980809	-1.623477	3.404746
H	5.398880	-1.446220	-0.134994
H	5.219531	-1.566951	2.338443
C	1.207999	1.200129	-0.903046
C	2.035542	2.030503	-1.653850
C	0.933115	1.542742	0.422721

atom	x [Å]	y [Å]	z [Å]
C	2.577674	3.187121	-1.098629
H	2.262582	1.773152	-2.684560
C	1.470259	2.694830	0.979985
H	0.307652	0.896949	1.028297
C	2.294575	3.521999	0.219722
H	3.219916	3.824091	-1.697880
H	1.250264	2.946673	2.012284
H	2.715243	4.421688	0.656780

19.4. Neutral Phosphinates for redox potential calculations

1a

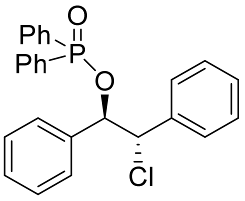

52 atoms

G = -1955.620283 E_h

Charge = 0; Multiplicity = 1

atom	x [Å]	y [Å]	z [Å]
P	-0.269799	1.041658	0.736313
C	0.623993	2.557445	0.323272
C	-0.027727	3.789771	0.406098
C	1.983133	2.511529	0.005224
C	0.674638	4.966061	0.172561
H	-1.086172	3.835624	0.643556
C	2.681889	3.690055	-0.226564
H	2.495319	1.558158	-0.072724
C	2.028851	4.916208	-0.143263
H	0.163601	5.920693	0.233335
H	3.736345	3.650272	-0.477544
H	2.575615	5.834650	-0.328769
C	-1.939945	1.214258	0.081136
C	-2.149112	1.493114	-1.272023
C	-3.026055	1.084582	0.942554
C	-3.440555	1.639530	-1.756051
H	-1.305169	1.595921	-1.947203
C	-4.319699	1.237007	0.454208
H	-2.857991	0.859363	1.989895
C	-4.525909	1.514096	-0.891125
H	-3.603371	1.851908	-2.807130
H	-5.165274	1.132462	1.125328
H	-5.535388	1.630515	-1.271436
O	-0.229170	0.668702	2.181242
O	0.441682	-0.041820	-0.237245
C	1.035390	-1.258910	0.238194
H	0.851695	-1.354843	1.310943
C	0.294866	-2.383977	-0.500586
H	0.486743	-2.302148	-1.569741
Cl	1.034568	-3.974639	-0.013628
C	-1.186356	-2.374147	-0.235932
C	-2.064966	-2.097025	-1.279368
C	-1.694943	-2.580871	1.046910
C	-3.434485	-2.026607	-1.048295
H	-1.677220	-1.921236	-2.278083
C	-3.061810	-2.516117	1.277668
H	-1.023077	-2.800779	1.870857

atom	x [Å]	y [Å]	z [Å]
C	-3.934825	-2.237762	0.229633
H	-4.107546	-1.795832	-1.866767
H	-3.446917	-2.676334	2.279145
H	-5.002155	-2.175345	0.413261
C	2.520504	-1.198291	-0.020123
C	3.417675	-1.321273	1.036009
C	3.006827	-0.968947	-1.307778
C	4.788233	-1.224599	0.811727
H	3.045605	-1.490331	2.041975
C	4.373149	-0.868740	-1.533335
H	2.316339	-0.848028	-2.136700
C	5.267561	-0.997973	-0.472931
H	5.478959	-1.321288	1.642578
H	4.742389	-0.686711	-2.537022
H	6.334986	-0.918242	-0.649656

1d

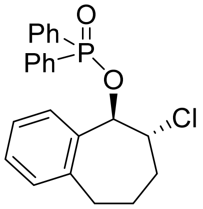

49 atoms

G = -1841.340454 E_h

Charge = 0; Multiplicity = 1

atom	x [Å]	y [Å]	z [Å]
P	1.329097	0.193389	-0.962374
C	2.116335	1.687869	-0.332138
C	3.509710	1.782714	-0.371308
C	1.357216	2.763396	0.131397
C	4.138365	2.947340	0.049690
H	4.107933	0.946923	-0.721926
C	1.991452	3.926836	0.551908
H	0.276573	2.685854	0.172074
C	3.378896	4.019054	0.510817
H	5.220109	3.017616	0.021158
H	1.400877	4.760611	0.915546
H	3.870988	4.927197	0.842369
C	2.155307	-1.221593	-0.201763
C	2.469150	-1.213590	1.159712
C	2.399808	-2.361109	-0.966696
C	3.020965	-2.342072	1.750278
H	2.280309	-0.328828	1.760247
C	2.949629	-3.491341	-0.370964
H	2.157445	-2.361327	-2.024067
C	3.257943	-3.481705	0.985076
H	3.264307	-2.334960	2.807011
H	3.138094	-4.377921	-0.966414
H	3.686717	-4.363788	1.448612
O	-0.107825	0.284971	-0.223369
O	1.236693	0.072575	-2.447238
C	-1.318123	-0.249658	-0.790211
C	-1.378411	-1.754861	-0.476601
H	-1.254633	-0.153471	-1.876282
C	-1.211410	-2.174170	0.974481
H	-0.604443	-2.245321	-1.068674
C	-2.181617	-1.581586	1.991128
H	-0.188627	-1.904852	1.257998

atom	x [Å]	y [Å]	z [Å]
H	-1.256463	-3.266247	1.010108
C	-2.090962	-0.057170	2.136758
H	-3.210552	-1.870255	1.751427
H	-1.953300	-2.036244	2.959642
H	-2.519480	0.230604	3.099950
H	-1.037034	0.239176	2.165072
Cl	-2.929221	-2.402666	-1.176471
C	-2.809066	0.711581	1.052119
C	-2.465279	0.605145	-0.304800
C	-3.867100	1.551168	1.399852
H	-4.144923	1.641274	2.445962
C	-4.575367	2.271911	0.443607
H	-5.394441	2.915095	0.748428
C	-4.229455	2.162463	-0.895723
H	-4.770616	2.717769	-1.654196
C	-3.174809	1.332310	-1.258061
H	-2.898574	1.247624	-2.304928

1g

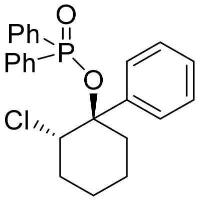

52

G = -1880.624592 E_h

Charge = 0; Multiplicity = 1

atom	x [Å]	y [Å]	z [Å]
P	-1.131375	0.194880	0.813156
C	-0.833063	1.878239	0.220767
C	-0.167483	2.776790	1.053085
C	-1.219938	2.272665	-1.061187
C	0.122401	4.058026	0.600565
H	0.132472	2.466752	2.047842
C	-0.930953	3.554601	-1.509573
H	-1.743117	1.581432	-1.714763
C	-0.256941	4.446191	-0.679651
H	0.649451	4.751860	1.246548
H	-1.230749	3.858547	-2.506749
H	-0.027980	5.446271	-1.032778
C	-2.736765	-0.329641	0.173154
C	-2.876202	-0.998601	-1.044434
C	-3.870108	-0.015539	0.926399
C	-4.140663	-1.350433	-1.502018
H	-1.998739	-1.246205	-1.630206
C	-5.132288	-0.369272	0.465127
H	-3.767086	0.499931	1.875932
C	-5.267529	-1.036234	-0.748735
H	-4.245421	-1.872319	-2.447013
H	-6.009709	-0.127137	1.054818
H	-6.253224	-1.312672	-1.107782
O	-0.179962	-0.782116	-0.051940
O	-0.998141	0.102297	2.296574
C	2.034887	0.202152	0.084069
C	1.945382	0.988102	-1.068076
C	2.836514	0.646815	1.130880
C	2.636597	2.184568	-1.171223
H	1.302765	0.681510	-1.886310

atom	x [Å]	y [Å]	z [Å]
C	3.534364	1.848421	1.030316
H	2.929311	0.066574	2.040760
C	3.437182	2.620102	-0.118764
H	2.540192	2.785550	-2.069052
H	4.152187	2.178641	1.858884
H	3.975469	3.559124	-0.194311
C	1.226428	-1.088879	0.166402
C	1.507152	-2.024286	-1.037898
C	1.383661	-1.857164	1.483039
C	0.715507	-3.324231	-0.977883
H	1.290561	-1.494234	-1.963037
C	0.544958	-3.134424	1.522298
H	2.440820	-2.114699	1.599100
H	1.111200	-1.206600	2.314496
C	0.871363	-4.057913	0.351089
H	1.005037	-3.956664	-1.820491
H	-0.335474	-3.058099	-1.128255
H	0.726381	-3.646377	2.471434
H	-0.519291	-2.876456	1.504957
H	0.217106	-4.933994	0.360893
H	1.897124	-4.430594	0.450336
Cl	3.292316	-2.377948	-1.129774

1o

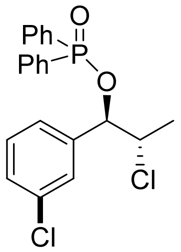

45 atoms

G = -2223.578801 E_h

Charge = 0; Multiplicity = 1

atom	x [Å]	y [Å]	z [Å]
P	-1.342307	0.287288	0.791236
C	-1.173305	2.030267	0.360545
C	-2.292529	2.864787	0.390833
C	0.087301	2.566424	0.088583
C	-2.151009	4.225426	0.145994
H	-3.277147	2.456666	0.596860
C	0.223957	3.927172	-0.154937
H	0.959493	1.922270	0.058624
C	-0.893937	4.755849	-0.126784
H	-3.022610	4.870282	0.165254
H	1.203414	4.340106	-0.370309
H	-0.785472	5.817636	-0.320687
C	-2.899659	-0.310010	0.104879
C	-3.242946	-0.048062	-1.224027
C	-3.731069	-1.098732	0.898109
C	-4.412184	-0.576142	-1.753596
H	-2.599122	0.565651	-1.846618
C	-4.901678	-1.626753	0.363608
H	-3.458644	-1.299475	1.928511
C	-5.240386	-1.366513	-0.959608
H	-4.678502	-0.372893	-2.784974
H	-5.548185	-2.240664	0.981051
H	-6.153383	-1.779101	-1.375627
O	-0.204146	-0.387428	-0.151403
O	-1.180509	-0.038414	2.238569

atom	x [Å]	y [Å]	z [Å]
C	0.684800	-1.408744	0.325699
H	0.331273	-1.779642	1.291381
C	0.609038	-2.546630	-0.698591
H	1.014164	-2.214395	-1.654577
Cl	1.736212	-3.862302	-0.143569
C	-0.786855	-3.110491	-0.866140
H	-1.196283	-3.439715	0.092330
H	-1.445396	-2.344303	-1.279131
H	-0.777343	-3.957752	-1.552825
C	2.057950	-0.800937	0.492281
C	2.727122	-0.883502	1.707678
C	2.642101	-0.116761	-0.573224
C	3.978083	-0.294482	1.860686
H	2.270653	-1.407600	2.540738
C	3.883867	0.470991	-0.400287
H	2.124898	-0.023644	-1.521643
C	4.567695	0.390534	0.806366
H	4.496894	-0.361773	2.810313
H	5.538410	0.858002	0.920337
Cl	4.604764	1.346161	-1.737213

1r

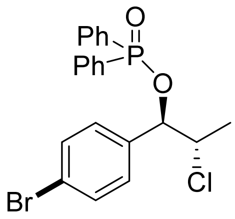

45 atoms

G = -4337.545268 E_h

Charge = 0; Multiplicity = 1

atom	x [Å]	y [Å]	z [Å]
P	-1.621468	0.615449	0.699156
C	-2.624081	1.927081	-0.018560
C	-3.214541	2.844413	0.853786
C	-2.853289	2.025187	-1.392689
C	-4.030857	3.851151	0.353699
H	-3.033967	2.775574	1.921652
C	-3.669264	3.035428	-1.887559
H	-2.389071	1.320830	-2.072995
C	-4.258563	3.945792	-1.015926
H	-4.486437	4.563338	1.032772
H	-3.843290	3.112243	-2.955193
H	-4.895528	4.732846	-1.405223
C	-2.688255	-0.799973	1.062555
C	-3.817355	-1.073897	0.287770
C	-2.331977	-1.663766	2.099776
C	-4.576957	-2.207710	0.543590
H	-4.109800	-0.403767	-0.514358
C	-3.091463	-2.801294	2.349170
H	-1.463260	-1.443049	2.711035
C	-4.211517	-3.073538	1.570734
H	-5.454907	-2.415955	-0.057954
H	-2.809757	-3.472347	3.153177
H	-4.804898	-3.960079	1.767499
O	-0.778898	0.161074	-0.611754
O	-0.801413	1.015559	1.877786
C	0.111065	-0.964897	-0.572285
H	-0.206251	-1.665424	0.208017

atom	x [Å]	y [Å]	z [Å]
C	-0.061730	-1.660470	-1.929027
H	0.299309	-1.007038	-2.723731
Cl	1.045414	-3.101720	-1.962852
C	-1.483589	-2.110669	-2.194013
H	-1.851048	-2.752609	-1.389446
H	-2.136461	-1.239432	-2.264477
H	-1.542444	-2.656675	-3.136228
C	1.517379	-0.493364	-0.290698
C	2.278073	-1.097574	0.703491
C	2.067623	0.552493	-1.028569
C	3.578367	-0.676794	0.960113
H	1.858446	-1.907670	1.291375
C	3.362898	0.987626	-0.784392
H	1.480061	1.046821	-1.795025
C	4.107102	0.363306	0.209542
H	4.163487	-1.153240	1.737406
H	3.783428	1.803000	-1.360546
Br	5.885156	0.953025	0.552060

1aa

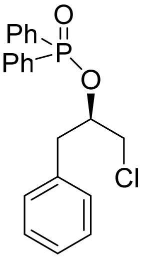

52 atoms

G = -1955.620283 E_h

Charge = 0; Multiplicity = 1

atom	x [Å]	y [Å]	z [Å]
P	-1.543735	-0.322613	0.787184
C	-0.747570	1.307183	0.807915
C	-0.070603	1.704232	1.962380
C	-0.764183	2.147378	-0.308117
C	0.595977	2.924100	1.994725
H	-0.068078	1.057905	2.833438
C	-0.099322	3.366502	-0.271718
H	-1.299374	1.857797	-1.207005
C	0.583325	3.753481	0.878221
H	1.124618	3.225875	2.892163
H	-0.115086	4.015019	-1.140590
H	1.104244	4.704539	0.903812
C	-3.007165	-0.238874	-0.259133
C	-2.941620	-0.413515	-1.643271
C	-4.229431	0.058541	0.348120
C	-4.092556	-0.288463	-2.412192
H	-1.996693	-0.655244	-2.116394
C	-5.376810	0.183219	-0.425261
H	-4.284469	0.187170	1.424061
C	-5.307959	0.010810	-1.804459
H	-4.040463	-0.427919	-3.486418
H	-6.325029	0.411507	0.048901
H	-6.204752	0.107490	-2.407158
O	-1.801048	-0.820194	2.168386
O	-0.586979	-1.256356	-0.127509
C	0.844581	-1.231781	-0.031838
H	1.148725	-0.659594	0.849711
C	1.346376	-2.653281	0.094222
H	2.432639	-2.674213	0.030325

atom	x [Å]	y [Å]	z [Å]
H	0.920848	-3.283631	-0.684742
C	1.413206	-0.567776	-1.292915
H	1.309985	-1.249652	-2.142232
H	0.794673	0.305254	-1.509940
Cl	0.907354	-3.399545	1.673629
C	2.846373	-0.135805	-1.110574
C	3.903427	-0.847186	-1.675452
C	3.131861	0.991339	-0.336267
C	5.220236	-0.443271	-1.471466
H	3.697477	-1.723370	-2.283795
C	4.444380	1.397368	-0.130050
H	2.316647	1.559455	0.104530
C	5.493944	0.679094	-0.697555
H	6.032098	-1.006587	-1.919802
H	4.648764	2.277122	0.471550
H	6.519644	0.995176	-0.538763

1ai

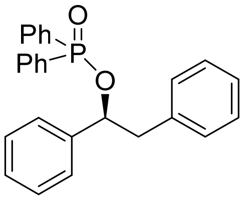

52 atoms

G = -1496.001615 E_h

Charge = 0; Multiplicity = 1

atom	x [Å]	y [Å]	z [Å]
P	-0.080157	0.794698	0.729779
C	0.967479	2.216317	0.339578
C	0.470801	3.508843	0.523392
C	2.291488	2.033675	-0.063758
C	1.291918	4.608436	0.305880
H	-0.560255	3.661480	0.827998
C	3.110250	3.136104	-0.279435
H	2.680896	1.033107	-0.219592
C	2.611612	4.421996	-0.095167
H	0.900808	5.610320	0.445554
H	4.137276	2.990441	-0.596563
H	3.251214	5.281039	-0.268073
C	-1.728518	1.166988	0.098608
C	-1.912637	1.550458	-1.232175
C	-2.823833	1.064907	0.951889
C	-3.187778	1.827739	-1.702489
H	-1.061556	1.632500	-1.901466
C	-4.100743	1.347730	0.478081
H	-2.676246	0.759595	1.981905
C	-4.282036	1.728381	-0.845397
H	-3.330910	2.122183	-2.736591
H	-4.953662	1.263513	1.142801
H	-5.278766	1.946046	-1.214825
O	-0.080816	0.401889	2.171356
O	0.502248	-0.337936	-0.261387
C	0.907970	-1.647460	0.189101
H	0.695414	-1.724095	1.258046
C	0.080282	-2.686134	-0.568017
H	0.292336	-2.597713	-1.637431
C	-1.398075	-2.539465	-0.309291
C	-2.244611	-1.996933	-1.274536

atom	x [Å]	y [Å]	z [Å]
C	-1.939959	-2.907654	0.923338
C	-3.601660	-1.831836	-1.021103
H	-1.835159	-1.690174	-2.232497
C	-3.295611	-2.741197	1.183149
H	-1.294647	-3.331323	1.688598
C	-4.131060	-2.203422	0.208988
H	-4.242430	-1.395691	-1.780106
H	-3.699973	-3.032112	2.147478
H	-5.188101	-2.065876	0.412125
C	2.393185	-1.796881	-0.034582
C	3.222544	-2.200023	1.008000
C	2.952525	-1.531217	-1.285669
C	4.593041	-2.343208	0.806401
H	2.798134	-2.398901	1.987793
C	4.319955	-1.666467	-1.487580
H	2.317198	-1.199894	-2.101117
C	5.144046	-2.075169	-0.441104
H	5.229179	-2.656353	1.627644
H	4.745405	-1.451955	-2.462380
H	6.212304	-2.180257	-0.599001
H	0.429000	-3.674998	-0.253344

1al

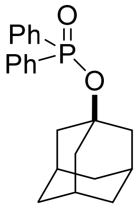

50 atoms

G = -1344.829210 E_h

Charge = 0; Multiplicity = 1

atom	x [Å]	y [Å]	z [Å]
P	1.045302	-0.156312	-0.754729
C	1.262637	1.545876	-0.170683
C	1.411320	2.575571	-1.097522
C	1.285270	1.832770	1.196198
C	1.574925	3.886681	-0.661252
H	1.388172	2.349904	-2.158145
C	1.448488	3.141500	1.629556
H	1.161976	1.036871	1.924086
C	1.592609	4.169336	0.700106
H	1.686106	4.686752	-1.385237
H	1.460235	3.362031	2.691493
H	1.718290	5.192008	1.039842
C	2.481296	-1.092843	-0.180873
C	2.540590	-1.659106	1.094327
C	3.577333	-1.200999	-1.038959
C	3.688019	-2.327595	1.504222
H	1.687930	-1.588473	1.759963
C	4.723506	-1.869750	-0.624703
H	3.531950	-0.768498	-2.033253
C	4.779463	-2.431753	0.646724
H	3.728985	-2.770625	2.493485
H	5.571142	-1.954743	-1.296072
H	5.673771	-2.954457	0.969227
O	0.844899	-0.213474	-2.233574
O	-0.091965	-0.795474	0.174495
C	-1.532124	-0.597316	0.087480

atom	x [Å]	y [Å]	z [Å]
C	-2.102743	-1.556970	-0.958124
C	-2.073022	-0.952797	1.470951
C	-1.916644	0.844186	-0.257729
H	-1.793909	-2.578488	-0.712017
H	-1.689678	-1.314395	-1.941829
C	-3.635261	-1.441280	-0.969532
H	-1.635324	-0.277054	2.213715
H	-1.763911	-1.971487	1.727572
C	-3.605117	-0.836923	1.466156
H	-1.510820	1.121685	-1.236529
H	-1.494995	1.531434	0.481751
C	-3.450703	0.961124	-0.273834
H	-4.039743	-2.130192	-1.717594
C	-4.184211	-1.799866	0.419078
C	-4.029219	0.000279	-1.322045
H	-3.986086	-1.095178	2.459093
C	-4.002498	0.603422	1.113372
H	-3.720548	1.991298	-0.525813
H	-3.922121	-2.833666	0.672055
H	-5.278259	-1.736935	0.417613
H	-3.654839	0.260123	-2.318913
H	-5.120480	0.094774	-1.353490
H	-5.093609	0.704235	1.119702
H	-3.610736	1.298056	1.865382

4a

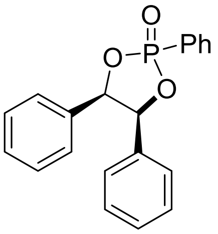

45 atoms

G = -2223.578801 E_h

Charge = 0; Multiplicity = 1

atom	x [Å]	y [Å]	z [Å]
P	-1.562038	-1.266652	-1.050223
O	-2.202729	-2.372305	-1.794241
O	-0.757569	-0.192594	-1.948971
O	-0.295545	-1.695221	-0.127060
C	-2.684208	-0.356013	0.007637
C	-3.547968	-1.071987	0.842077
C	-2.716340	1.039336	-0.000285
C	-4.420470	-0.390923	1.679994
H	-3.540069	-2.156923	0.839226
C	-3.600714	1.714103	0.832883
H	-2.058021	1.597028	-0.655434
C	-4.446814	1.001146	1.675152
H	-5.084087	-0.946804	2.332886
H	-3.627905	2.798016	0.821769
H	-5.133304	1.530769	2.326949
C	0.653585	-0.106721	-1.618455
H	1.186128	-0.152621	-2.567748
C	0.932306	-1.411899	-0.816803
H	1.100733	-2.216244	-1.540300
C	2.094477	-1.347832	0.135415
C	1.925898	-1.432229	1.512901
C	3.375062	-1.183022	-0.392444
C	3.028532	-1.340744	2.357435

atom	x [Å]	y [Å]	z [Å]
H	0.933656	-1.566326	1.926869
C	4.473818	-1.085828	0.451380
H	3.516985	-1.127295	-1.468024
C	4.302625	-1.162706	1.831091
H	2.887901	-1.407493	3.431127
H	5.465009	-0.952860	0.031149
H	5.160347	-1.088733	2.491144
C	0.963590	1.197743	-0.930561
C	1.679106	2.170706	-1.623685
C	0.554923	1.461860	0.378753
C	1.980759	3.391151	-1.026059
H	2.006878	1.976345	-2.640613
C	0.851437	2.678571	0.976201
H	0.005356	0.716362	0.941891
C	1.565735	3.647074	0.274886
H	2.538896	4.139314	-1.578644
H	0.527979	2.870610	1.993635
H	1.798794	4.596856	0.744544

19.5. Products after bond cleavages for BDFE calculations

$Ph_2P(O)$-O^-

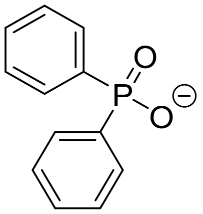

25 atoms

G = -955.099869 E_h

Charge = -1; Multiplicity = 1

atom	x [Å]	y [Å]	z [Å]
P	-0.000002	1.419619	0.000247
C	-1.429625	0.263759	-0.016268
C	-1.906840	-0.260266	-1.220244
C	-2.037325	-0.132739	1.176744
C	-2.967140	-1.161048	-1.232510
H	-1.446287	0.044919	-2.154703
C	-3.098979	-1.032967	1.168990
H	-1.677333	0.274357	2.116271
C	-3.565086	-1.549272	-0.036536
H	-3.329899	-1.558441	-2.175318
H	-3.564582	-1.329933	2.103588
H	-4.393167	-2.250921	-0.044635
C	1.429606	0.263729	0.016383
C	2.037474	-0.132144	-1.176758
C	1.906676	-0.260917	1.220144
C	3.099139	-1.032352	-1.169333
H	1.677572	0.275426	-2.116112
C	2.966982	-1.161693	1.232079
H	1.446004	0.043753	2.154714
C	3.565100	-1.549283	0.035986
H	3.564876	-1.328828	-2.104021
H	3.329626	-1.559584	2.174722
H	4.393192	-2.250920	0.043813
O	-0.018222	2.175852	1.313860
O	0.018239	2.176279	-1.313120

Radical from C(sp^3)-O cleavage of 1a$^{•-}$

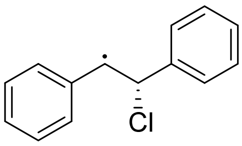

27 atoms

G = -1000.645974 E_h

Charge = 0; Multiplicity = 2

atom	x [Å]	y [Å]	z [Å]
C	-0.537841	-0.374214	0.422737
H	-0.179012	-1.079264	1.165729
C	-1.924110	-0.374908	0.123239
C	-2.761640	-1.338556	0.739255
C	-2.527739	0.546839	-0.767440
C	-4.117618	-1.381666	0.473569
H	-2.322258	-2.053018	1.428702
C	-3.886387	0.495976	-1.025237
H	-1.930705	1.312450	-1.250473
C	-4.690141	-0.465684	-0.411223
H	-4.737492	-2.130032	0.955896
H	-4.328284	1.213117	-1.708925
H	-5.754156	-0.499219	-0.618504
C	0.450999	0.544502	-0.163077
C	1.861322	0.026767	-0.171634
C	2.487636	-0.402309	1.000481
C	2.551433	-0.052042	-1.379395
C	3.780540	-0.905201	0.961325
H	1.968819	-0.331436	1.951530
C	3.846696	-0.560326	-1.420072
H	2.075764	0.281946	-2.296585
C	4.463383	-0.987483	-0.250533
H	4.258781	-1.232667	1.878290
H	4.372225	-0.619206	-2.367199
H	5.473644	-1.381621	-0.279732
H	0.169253	0.871569	-1.161243
Cl	0.409072	2.177483	0.794738

Radical from C(sp^3)-O cleavage of 1d$^{•-}$

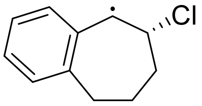

24 atoms

G = -886.364357 E_h

Charge = 0; Multiplicity = 2

atom	x [Å]	y [Å]	z [Å]
C	-0.518738	-0.954014	0.939864
C	-1.876389	-0.430694	0.711027
H	-0.518174	-1.963626	1.341968
C	-2.144229	1.058664	0.653112
H	-2.563184	-0.895612	1.415488
C	-1.274643	1.859625	-0.304663
H	-2.007263	1.437607	1.673760
H	-3.201527	1.200130	0.414921
C	0.171554	2.008080	0.163886
H	-1.307572	1.422670	-1.308039
H	-1.704471	2.861681	-0.391454
H	0.630454	2.840486	-0.375675
H	0.164073	2.310286	1.220133

atom	x [Å]	y [Å]	z [Å]
Cl	-2.474161	-1.198895	-0.922270
C	1.083476	0.812331	0.026411
C	0.750685	-0.500926	0.473125
C	2.365228	1.038268	-0.470047
H	2.617925	2.034856	-0.820078
C	3.339920	0.045926	-0.513249
H	4.324966	0.269677	-0.908545
C	3.042184	-1.226278	-0.028943
H	3.791735	-2.010325	-0.038089
C	1.776478	-1.485204	0.455599
H	1.540616	-2.481285	0.817465

Radical from C(sp^3)-O cleavage of 1g$^{•−}$

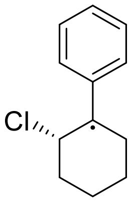

27 atoms

G = -925.655897 E_h

Charge = 0; Multiplicity = 2

atom	x [Å]	y [Å]	z [Å]
C	1.150654	0.210099	-0.046406
C	1.775024	-1.014427	-0.395965
C	2.011137	1.290987	0.268602
C	3.153263	-1.140709	-0.432660
H	1.177644	-1.891619	-0.613608
C	3.388756	1.158880	0.221861
H	1.598962	2.256524	0.533116
C	3.974394	-0.056309	-0.128487
H	3.592864	-2.097476	-0.695102
H	4.013400	2.013800	0.459525
H	5.053729	-0.157773	-0.160810
C	-0.279836	0.347870	-0.010849
C	-1.154614	-0.720976	-0.551330
C	-0.973966	1.466722	0.713018
C	-2.453376	-0.227032	-1.175069
H	-0.640349	-1.368889	-1.254433
C	-2.234573	1.968333	-0.000831
H	-1.265062	1.089987	1.706528
H	-0.302457	2.303093	0.902994
C	-3.175245	0.814464	-0.329589
H	-3.095153	-1.081813	-1.400736
H	-2.174319	0.218644	-2.138662
H	-2.736260	2.707020	0.630676
H	-1.947535	2.482577	-0.925920
H	-4.055276	1.176235	-0.868557
H	-3.537895	0.355450	0.597386
Cl	-1.574824	-1.917245	0.849049

Radical from C(sp^3)-O cleavage of 1o$^{•−}$

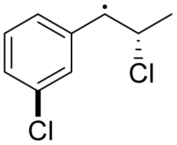

20 atoms

G = -1268.607783 E_h

Charge = 0; Multiplicity = 2

atom	x [Å]	y [Å]	z [Å]
C	-1.454507	1.004941	-0.183652
H	-1.942766	1.952118	0.028420
C	-2.332152	-0.126961	-0.534630
H	-1.833732	-0.861265	-1.164955
Cl	-2.685923	-1.109532	1.021849
C	-3.654121	0.291081	-1.143992
H	-4.196656	0.969177	-0.481625
H	-3.460429	0.811570	-2.086193
H	-4.282516	-0.575238	-1.353908
C	-0.044904	0.943738	-0.053066
C	0.677616	2.121483	0.260535
C	0.686509	-0.257619	-0.221484
C	2.053142	2.101018	0.388160
H	0.135121	3.051046	0.397659
C	2.059834	-0.246285	-0.086075
H	0.182506	-1.189679	-0.445427
C	2.766747	0.914681	0.215456
H	2.588822	3.013326	0.625665
H	3.844972	0.892892	0.315442
Cl	2.945670	-1.745786	-0.297295

Radical from C(sp^2)-Cl cleavage of 1o$^{\bullet -}$

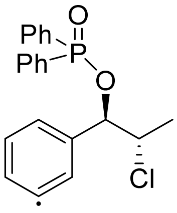

44 atoms

G = -1763.282792 E_h

Charge = 0; Multiplicity = 2

atom	x [Å]	y [Å]	z [Å]
P	-0.959183	0.191301	0.722594
C	-1.138417	1.914544	0.219360
C	-2.379249	2.544017	0.340297
C	-0.025972	2.645092	-0.203720
C	-2.505871	3.894243	0.037229
H	-3.251016	1.982749	0.662549
C	-0.157289	3.995193	-0.504369
H	0.939262	2.160808	-0.307965
C	-1.395528	4.619089	-0.384657
H	-3.471682	4.379058	0.127443
H	0.707292	4.559323	-0.836839
H	-1.496152	5.672380	-0.623926
C	-2.431750	-0.698659	0.181941
C	-2.891382	-0.588408	-1.132841
C	-3.078201	-1.547182	1.079042
C	-3.991226	-1.328045	-1.545192
H	-2.392963	0.073022	-1.834838
C	-4.180496	-2.286090	0.662256
H	-2.715914	-1.628943	2.097963
C	-4.634675	-2.177183	-0.647436
H	-4.347883	-1.243269	-2.565777
H	-4.683529	-2.945938	1.360587
H	-5.494142	-2.754273	-0.971659
O	0.203184	-0.336808	-0.279979
O	-0.640263	-0.021924	2.164956
C	1.376592	-1.024790	0.182785
H	1.261020	-1.259133	1.244294

atom	x [Å]	y [Å]	z [Å]
C	1.438001	-2.337516	-0.604338
H	1.615015	-2.136039	-1.660700
Cl	2.915332	-3.240672	-0.045905
C	0.208994	-3.203432	-0.417161
H	0.022332	-3.398888	0.642005
H	-0.663418	-2.697810	-0.835672
H	0.329102	-4.156486	-0.933581
C	2.563614	-0.109479	-0.004619
C	3.307195	0.310958	1.094639
C	2.891189	0.369673	-1.282390
C	4.375885	1.192575	0.946929
H	3.047995	-0.051799	2.084122
C	3.949364	1.234903	-1.367044
H	2.315092	0.078800	-2.155568
C	4.718363	1.677707	-0.317258
H	4.942794	1.508074	1.816732
H	5.545679	2.366864	-0.448247

Radical from C(sp^3)-O cleavage of $1r^{\bullet-}$

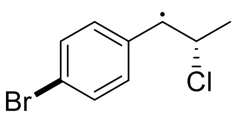

20 atoms

G = -3382.574974 E_h

Charge = 0; Multiplicity = 2

atom	x [Å]	y [Å]	z [Å]
C	2.314955	0.922863	-0.033668
H	2.625123	1.838234	-0.530250
C	3.388833	0.071144	0.509964
H	3.055613	-0.533280	1.351593
Cl	3.822939	-1.230982	-0.768627
C	4.651829	0.829450	0.861525
H	5.035207	1.378073	-0.001620
H	4.423710	1.548965	1.653021
H	5.428451	0.155239	1.224613
C	0.933347	0.618509	-0.004291
C	0.004682	1.543448	-0.544442
C	0.414325	-0.582097	0.541003
C	-1.353630	1.295176	-0.534026
H	0.370494	2.470480	-0.974118
C	-0.944394	-0.835896	0.552476
H	1.081142	-1.330911	0.952678
C	-1.823757	0.103448	0.016884
H	-2.043617	2.019156	-0.950828
H	-1.319340	-1.761593	0.972783
Br	-3.690397	-0.247255	0.033607

Radical from C(sp^2)-Br cleavage of $1r^{\bullet-}$

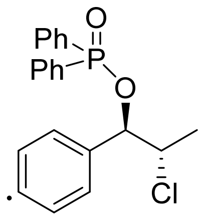

44 atoms
G = -1763.283534 E_h

Charge = 0; Multiplicity = 2

atom	x [Å]	y [Å]	z [Å]
P	-0.952781	0.181760	0.726503
C	-1.172820	1.898790	0.217751
C	-2.428950	2.498462	0.331465
C	-0.076620	2.654642	-0.203280
C	-2.587044	3.844160	0.022871
H	-3.288167	1.917411	0.652324
C	-0.239323	4.000108	-0.509311
H	0.900401	2.193252	-0.301597
C	-1.492863	4.594178	-0.397204
H	-3.564683	4.305750	0.107325
H	0.612780	4.583866	-0.840136
H	-1.617997	5.643846	-0.640832
C	-2.404574	-0.744129	0.189410
C	-2.868480	-0.647132	-1.124908
C	-3.029062	-1.606809	1.088513
C	-3.950591	-1.413782	-1.534782
H	-2.386984	0.024992	-1.828572
C	-4.113585	-2.372967	0.674247
H	-2.663521	-1.678082	2.107090
C	-4.572052	-2.277016	-0.634962
H	-4.310525	-1.339162	-2.555021
H	-4.599475	-3.043866	1.374193
H	-5.417705	-2.875250	-0.957188
O	0.221874	-0.319005	-0.274461
O	-0.630172	-0.019954	2.169786
C	1.404229	-0.993975	0.188870
H	1.277324	-1.256468	1.242338
C	1.497825	-2.287327	-0.627201
H	1.684744	-2.058751	-1.676315
Cl	2.983505	-3.178651	-0.070766
C	0.282461	-3.179097	-0.475878
H	0.087098	-3.403847	0.575919
H	-0.594030	-2.678657	-0.891973
H	0.425405	-4.117030	-1.013785
C	2.576261	-0.053476	0.039703
C	3.292226	0.344344	1.165764
C	2.909063	0.461233	-1.215297
C	4.352220	1.250758	1.054034
H	3.026872	-0.048124	2.143041
C	3.958569	1.370089	-1.354558
H	2.340062	0.172639	-2.093988
C	4.629340	1.716142	-0.205700
H	4.911810	1.561679	1.929677
H	4.217719	1.774960	-2.326903

Radical from C(sp^3)-O cleavage of 1aa$^{\bullet-}$

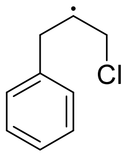

20 atoms

G = -808.970653 E_h

Charge = 0; Multiplicity = 2

atom	x [Å]	y [Å]	z [Å]
C	-1.580644	1.431978	-0.042872
H	-2.337338	2.193659	-0.203268
C	-1.847766	0.402272	0.950882

atom	x [Å]	y [Å]	z [Å]
H	-2.513087	0.725653	1.745856
H	-0.954609	-0.068263	1.355367
C	-0.476939	1.325414	-1.046678
H	-0.870395	0.839410	-1.952964
H	-0.189837	2.333823	-1.362660
Cl	-2.770567	-1.048543	0.164146
C	0.733114	0.565470	-0.554841
C	0.936166	-0.768634	-0.904338
C	1.651187	1.182046	0.297401
C	2.031916	-1.474083	-0.414401
H	0.228108	-1.262961	-1.563120
C	2.747418	0.482121	0.788353
H	1.504071	2.221030	0.579345
C	2.940460	-0.850571	0.433856
H	2.174723	-2.512109	-0.696807
H	3.453008	0.977335	1.447609
H	3.795525	-1.398415	0.815983

Radical from C(sp^3)-O cleavage of 1ai$^{•-}$

27 atoms

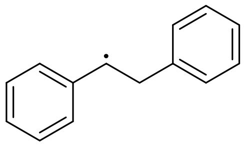

G = -541.025169 E_h

Charge = 0; Multiplicity = 2

atom	x [Å]	y [Å]	z [Å]
C	0.456252	0.308758	0.008513
H	0.028538	1.307336	0.024412
C	1.864735	0.188969	0.004737
C	2.680704	1.351302	0.021091
C	2.522311	-1.068580	-0.015140
C	4.059700	1.258735	0.017591
H	2.203745	2.327067	0.036505
C	3.904711	-1.148955	-0.018509
H	1.937439	-1.982355	-0.027909
C	4.684829	0.008487	-0.002303
H	4.659493	2.163214	0.030329
H	4.384100	-2.122656	-0.033875
H	5.767240	-0.061643	-0.005042
C	-0.487691	-0.855869	-0.007420
C	-1.935510	-0.428773	-0.004399
C	-2.616499	-0.228900	1.196749
C	-2.608053	-0.185599	-1.202365
C	-3.939151	0.203320	1.202577
H	-2.106640	-0.414989	2.138038
C	-3.930683	0.246650	-1.201948
H	-2.091529	-0.337710	-2.146123
C	-4.600654	0.442698	0.001908
H	-4.454312	0.350687	2.146381
H	-4.439195	0.427917	-2.143459
H	-5.632757	0.777566	0.004346
H	-0.291747	-1.483051	-0.887867
H	-0.294387	-1.504838	0.857762

Radical from C(sp^3)-O cleavage of 1al$^{•-}$

25 atoms

G = -389.829027 E$_h$

Charge = 0; Multiplicity = 2

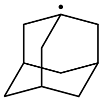

atom	x [Å]	y [Å]	z [Å]
C	0.000807	-0.000461	1.482886
C	-1.260248	-0.708575	1.076039
C	0.017595	1.445703	1.075934
C	1.244295	-0.738266	1.074783
H	-2.153064	-0.194197	1.449848
H	-1.285584	-1.738870	1.449213
C	-1.269387	-0.712964	-0.478451
H	0.909796	1.961736	1.448953
H	-0.861697	1.982648	1.450027
C	0.016692	1.455794	-0.478508
H	1.245119	-1.769037	1.447563
H	2.149412	-0.245573	1.448192
C	1.251952	-0.742306	-0.479669
H	-2.170887	-1.219320	-0.840778
C	-1.247614	0.739685	-0.980588
C	-0.017378	-1.449257	-0.981951
H	0.028627	2.489710	-0.840791
C	1.263507	0.710571	-0.981632
H	2.141022	-1.269546	-0.842913
H	-2.142823	1.270590	-0.635213
H	-1.272126	0.754132	-2.076667
H	-0.029374	-2.490383	-0.637795
H	-0.018326	-1.476446	-2.078067
H	1.287440	0.724614	-2.077717
H	2.171130	1.220392	-0.636922

Radical anion from C(sp^3)-O cleavage of 4a$^{•-}$

41 atoms

G = -1339.208685 E$_h$

Charge = -1; Multiplicity = 2

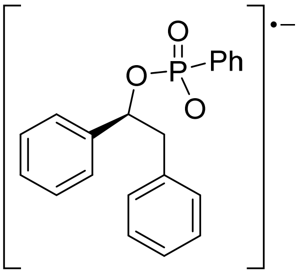

atom	x [Å]	y [Å]	z [Å]
P	-1.253386	-1.130423	0.056630
O	-1.198031	-2.524792	-0.515311
O	-0.810545	-0.041271	-1.117124
O	-0.539470	-0.820816	1.345420
C	-2.995793	-0.592495	0.179844
C	-3.970765	-1.120380	-0.668971
C	-3.372897	0.361237	1.127569
C	-5.293012	-0.696435	-0.580994
H	-3.690655	-1.871633	-1.400335
C	-4.694473	0.787028	1.219399
H	-2.624759	0.765815	1.801810
C	-5.656861	0.259187	0.363635
H	-6.041197	-1.114646	-1.246965
H	-4.974546	1.527811	1.961669

atom	x [Å]	y [Å]	z [Å]
H	-6.688432	0.589016	0.434796
C	0.532631	0.294747	-1.478509
H	0.454950	0.506168	-2.551861
C	1.491408	-0.861296	-1.378065
H	1.401798	-1.571350	-2.195606
C	2.482178	-1.153091	-0.407880
C	2.661091	-0.444555	0.808284
C	3.363540	-2.238812	-0.659268
C	3.668037	-0.792917	1.690927
H	1.980167	0.353984	1.065661
C	4.367321	-2.577803	0.229374
H	3.244186	-2.808044	-1.576678
C	4.532671	-1.853416	1.411386
H	3.778736	-0.237957	2.617348
H	5.026430	-3.410421	0.005002
H	5.319517	-2.117336	2.110145
C	0.981905	1.596712	-0.835391
C	2.190154	2.163937	-1.245200
C	0.215843	2.259794	0.116445
C	2.637489	3.357148	-0.694045
H	2.793783	1.657310	-1.993524
C	0.662340	3.456793	0.672071
H	-0.724196	1.828093	0.436171
C	1.874881	4.007390	0.273942
H	3.582472	3.780886	-1.018718
H	0.057874	3.958486	1.421268
H	2.222898	4.938564	0.709173

19.6. Candidate preassemblies of radical anion e-PRCat with 1d

NpMI•⁻ + 1d converged preassembly orientation **1**

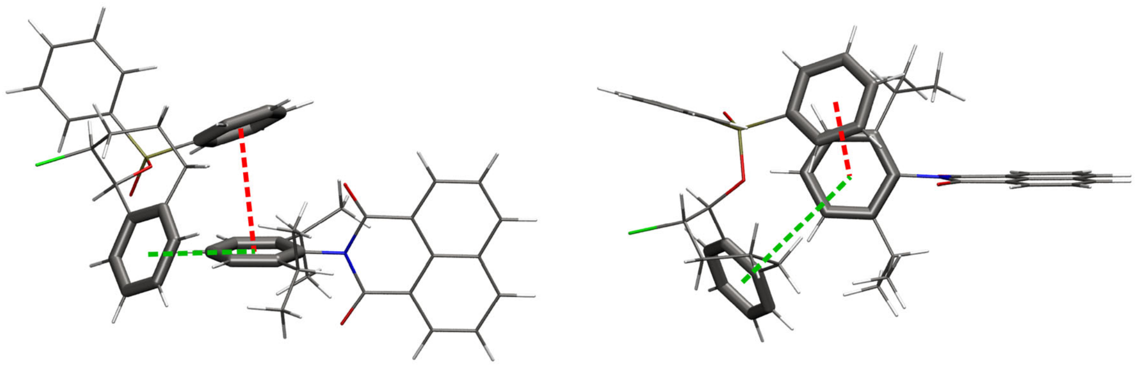

N-aniline to O-CHR-Ar: *T*-π interaction (centroid-to-centroid) = 4.88 Å.
N-aniline to O-P(O)-Ph: π-π interaction (centroid-to-centroid) = 4.23 Å.

99 atoms

G = -2974.652162 E_h

Charge = -1; Multiplicity = 2

atom	x [Å]	y [Å]	z [Å]
C	-6.664186	-1.204402	3.149079

atom	x [Å]	y [Å]	z [Å]
C	-7.741993	-0.885890	2.323993
C	-7.535621	-0.353003	1.030630
C	-6.186770	-0.150731	0.578749
C	-5.109848	-0.483543	1.434278
C	-5.361825	-1.008138	2.721449
H	-9.620243	-0.159481	0.499424
H	-6.850055	-1.611449	4.138754
H	-8.759645	-1.042937	2.670425
C	-8.598354	-0.006494	0.163930
C	-5.958915	0.390369	-0.709949
H	-4.521998	-1.255501	3.360213
C	-7.051255	0.732348	-1.539142
C	-8.348080	0.528096	-1.099389
H	-6.854097	1.150163	-2.519378
H	-9.182101	0.788231	-1.744745
O	-4.326182	1.125446	-2.261054
O	-2.755783	-0.557393	1.677375
C	-4.610766	0.604828	-1.172316
C	-3.750683	-0.300002	0.987478
N	-3.583057	0.180761	-0.318496
C	-2.233706	0.322639	-0.805467
C	-0.358854	-0.498297	-2.038901
C	-0.255839	1.666097	-1.015323
H	0.120019	-1.279270	-2.621671
H	0.300027	2.572549	-0.802297
C	-1.548223	1.510155	-0.518580
C	-1.654414	-0.706120	-1.560649
C	0.200611	-2.657734	1.675067
C	1.264096	-1.927873	1.159737
C	1.813987	-2.278734	-0.072827
C	1.297128	-3.362387	-0.785094
C	0.233966	-4.088855	-0.265760
C	-0.313242	-3.736556	0.963917
H	-0.235756	-2.375099	2.626093
H	1.657299	-1.080654	1.708746
H	1.713535	-3.628226	-1.751456
H	-0.174754	-4.923196	-0.825299
H	-1.148144	-4.300506	1.365684
P	3.203473	-1.393618	-0.791205
C	4.737094	-2.178521	-0.233976
C	4.855363	-2.692903	1.059781
C	5.842129	-2.178648	-1.085404
C	6.073251	-3.195311	1.498306
H	4.000019	-2.698639	1.728326
C	7.061479	-2.679231	-0.641494
H	5.746441	-1.783949	-2.091167
C	7.177282	-3.184169	0.649055
H	6.162694	-3.594773	2.502568
H	7.920331	-2.673918	-1.303692
H	8.128932	-3.573487	0.994859
O	3.129291	-0.001664	0.037696
O	3.175206	-1.242084	-2.274994
C	3.263787	2.381468	-0.277623
C	2.548775	2.814803	0.851801
C	3.317972	3.180736	-1.415910
C	1.949053	4.072594	0.804509
C	2.687349	4.419619	-1.456174
H	3.861676	2.828283	-2.287380

atom	x [Å]	y [Å]	z [Å]
C	2.009318	4.871084	-0.332953
H	1.410215	4.432881	1.675655
H	2.734339	5.025475	-2.354489
H	1.520929	5.839980	-0.338990
C	2.347511	1.948772	2.075709
H	1.853542	2.558926	2.835120
H	1.625781	1.171943	1.807527
C	3.610791	1.298241	2.697523
H	3.649183	1.558767	3.757485
H	3.531578	0.208438	2.658583
C	4.936656	1.731175	2.067597
H	4.951283	2.817604	1.942702
H	5.763947	1.479692	2.735703
C	4.026388	1.081143	-0.278639
H	4.433025	0.912200	-1.278021
Cl	6.638126	1.835978	-0.065689
C	5.194941	1.058250	0.728193
H	5.496911	0.023244	0.877043
C	0.335231	0.671756	-1.777406
H	1.344281	0.798293	-2.153249
C	-2.161930	2.609487	0.330547
H	-3.221256	2.382942	0.472388
C	-2.324455	-2.015031	-1.972258
H	-1.491339	-2.704207	-2.149022
C	-2.078785	3.979951	-0.348764
H	-1.043389	4.318643	-0.452300
H	-2.528644	3.958403	-1.345122
H	-2.609837	4.728799	0.246571
C	-3.058316	-1.853648	-3.310522
H	-3.895076	-1.157832	-3.215494
H	-2.389071	-1.468938	-4.085492
H	-3.448462	-2.819277	-3.648104
C	-1.506439	2.644245	1.716122
H	-1.604525	1.677908	2.214359
H	-0.441929	2.880122	1.632889
H	-1.971985	3.410010	2.344858
C	-3.229732	-2.703610	-0.946953
H	-2.792842	-2.694237	0.052606
H	-4.219982	-2.245482	-0.892103
H	-3.371512	-3.747733	-1.242893

NpMI$^{\bullet-}$ + 1d converged preassembly orientation **2**

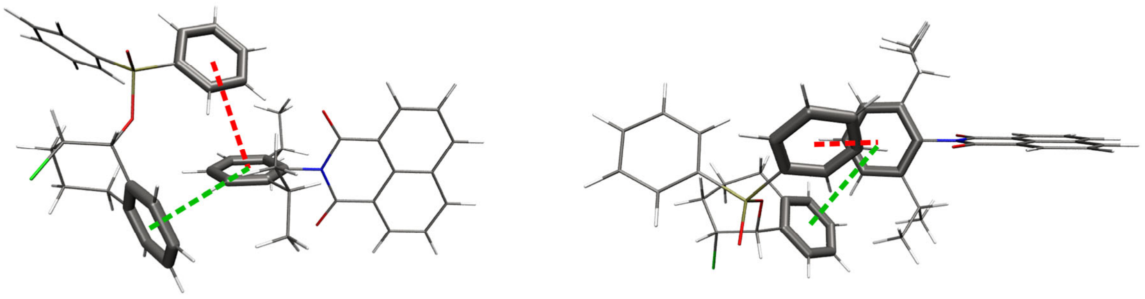

N-aniline to O-CHR-Ar: *T*-π interaction (centroid-to-centroid) = 4.86 Å.
N-aniline to O-P(O)-Ph: π-π interaction (centroid-to-centroid) = 4.48 Å.

99 atoms

G = -2974.663363 E_h

Charge = -1; Multiplicity = 2

atom	x [Å]	y [Å]	z [Å]
C	-8.492967	-1.645811	0.535273
C	-8.868905	-0.686664	-0.403930
C	-7.920796	0.211546	-0.946136
C	-6.555618	0.115984	-0.508774
C	-6.199003	-0.866753	0.446031
C	-7.179379	-1.743704	0.962219
H	-9.288859	1.276524	-2.233871
H	-9.240777	-2.323727	0.936287
H	-9.902583	-0.618918	-0.731157
C	-8.257965	1.200000	-1.899869
C	-5.591875	1.006805	-1.040834
H	-6.885150	-2.488152	1.692805
C	-5.972880	1.979771	-1.992329
C	-7.290699	2.065800	-2.408326
H	-5.219051	2.651310	-2.386264
H	-7.575943	2.816224	-3.139837
O	-3.310597	1.650250	-1.036642
O	-4.438789	-1.792666	1.736270
C	-4.218586	0.918759	-0.614298
C	-4.833204	-0.971127	0.896363
N	-3.919447	-0.068172	0.332878
C	-2.552507	-0.176188	0.777546
C	-1.680355	-1.016959	0.079676
C	-2.149212	0.540181	1.908568
C	-0.386127	-1.171406	0.572293
C	-0.838936	0.373206	2.355223
C	0.031791	-0.487379	1.703259
H	0.309655	-1.829395	0.063429
H	-0.492708	0.922701	3.224904
H	1.046169	-0.615714	2.067221
C	-3.082492	1.506989	2.613691
C	-2.717315	2.950888	2.248857
C	-3.106493	1.304359	4.130717
H	-4.096388	1.324921	2.249060
H	-2.743221	3.091150	1.166027
H	-3.417999	3.655114	2.708141
H	-1.709977	3.199784	2.599235
H	-3.341667	0.268163	4.388556
H	-2.147703	1.558064	4.592792
H	-3.866301	1.947421	4.584372
C	-2.108555	-1.758989	-1.172662
C	-2.258893	-3.258522	-0.895416
C	-1.146072	-1.501485	-2.336562
H	-3.088731	-1.380958	-1.472839
H	-2.980531	-3.434399	-0.093930
H	-2.604898	-3.783519	-1.791248
H	-1.302719	-3.698206	-0.596392
H	-1.035845	-0.430901	-2.530614
H	-0.152000	-1.912519	-2.138130
H	-1.520566	-1.974194	-3.249391
P	3.784560	1.359737	-1.246030
C	2.261718	2.260798	-0.919121

atom	x [Å]	y [Å]	z [Å]
C	2.291465	3.646271	-0.741460
C	1.041301	1.583187	-0.913903
C	1.104790	4.343633	-0.551993
H	3.235647	4.181984	-0.739497
C	-0.144016	2.280628	-0.717901
H	1.016144	0.506993	-1.038838
C	-0.107928	3.660135	-0.537616
H	1.128456	5.418643	-0.409020
H	-1.095135	1.758045	-0.694183
H	-1.032815	4.205210	-0.379208
C	5.100880	2.120906	-0.269958
C	4.900566	2.426023	1.079086
C	6.353286	2.315402	-0.849295
C	5.949943	2.919937	1.841216
H	3.927995	2.271932	1.536491
C	7.404320	2.806623	-0.081631
H	6.503822	2.077261	-1.896857
C	7.202806	3.106617	1.261016
H	5.793760	3.155609	2.888115
H	8.379305	2.954665	-0.532826
H	8.023319	3.488578	1.859043
O	3.478399	-0.045808	-0.492369
O	4.158473	1.188947	-2.682105
C	4.000690	-1.302182	-0.962380
C	5.492617	-1.401178	-0.592206
H	3.949417	-1.299202	-2.053230
C	5.881383	-1.191304	0.862520
H	6.029236	-0.671819	-1.200748
C	5.218934	-2.084128	1.906256
H	5.654206	-0.146524	1.096350
H	6.969038	-1.283973	0.928880
C	3.695707	-1.921996	1.988240
H	5.470964	-3.135180	1.730053
H	5.650336	-1.822784	2.877016
H	3.350976	-2.274511	2.963435
H	3.448641	-0.856317	1.936592
Cl	6.103615	-3.002180	-1.209376
C	2.946419	-2.679414	0.917848
C	3.099398	-2.400855	-0.449213
C	2.088057	-3.713560	1.289343
H	1.958186	-3.935976	2.344308
C	1.394513	-4.463410	0.345446
H	0.732178	-5.259930	0.668148
C	1.547166	-4.180756	-1.004204
H	1.005985	-4.748804	-1.753097
C	2.395007	-3.149017	-1.390027
H	2.512628	-2.922218	-2.445494

NpMI•⁻ + 1d converged preassembly orientation **3**

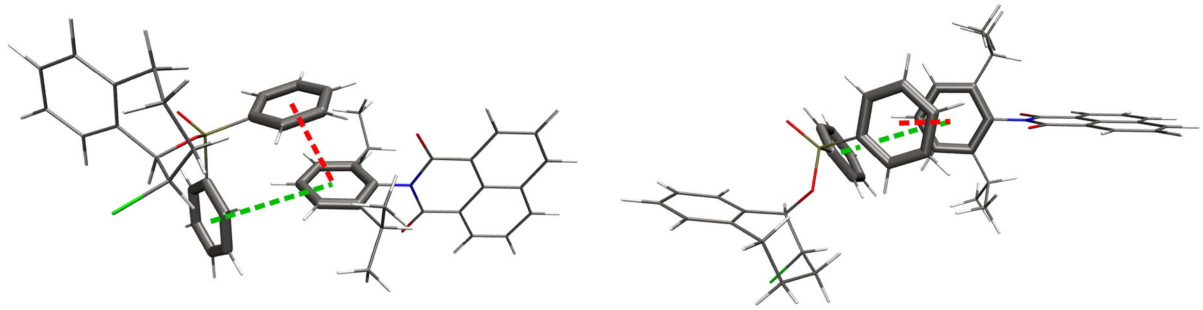

N-aniline to O-P(O)-Ar: *T*-π interaction (centroid-to-centroid) = 4.98 Å.
N-aniline to O-P(O)-Ph: π-π interaction (centroid-to-centroid) = 3.84 Å.
99 atoms

G = -2974.660276 E_h

Charge = -1; Multiplicity = 2

atom	x [Å]	y [Å]	z [Å]
C	9.060722	0.306840	-1.606240
C	9.318073	-0.907300	-0.970942
C	8.300147	-1.588519	-0.263814
C	6.989200	-1.002367	-0.211470
C	6.753143	0.231436	-0.865666
C	7.799960	0.877394	-1.561584
H	9.505669	-3.269003	0.357725
H	9.860142	0.809942	-2.142413
H	10.311119	-1.345789	-1.013135
C	8.516419	-2.821478	0.394188
C	5.957896	-1.669555	0.492769
H	7.597931	1.820998	-2.054882
C	6.218527	-2.900138	1.136402
C	7.484138	-3.459047	1.080893
H	5.414631	-3.392784	1.670820
H	7.676750	-4.405272	1.578119
O	3.683378	-1.605233	1.156469
O	5.146391	1.899092	-1.375494
C	4.638147	-1.090988	0.557900
C	5.442026	0.829418	-0.822803
N	4.460497	0.131270	-0.104898
C	3.152478	0.732568	-0.046170
C	2.218892	0.419273	-1.038770
C	2.859787	1.617081	0.996451
C	0.966863	1.025213	-0.971333
C	1.598583	2.210722	1.016469
C	0.657765	1.920868	0.040665
H	0.216034	0.790695	-1.717643
H	1.343039	2.902170	1.813644
H	-0.324074	2.381783	0.074277
C	3.866308	1.939213	2.085955
C	3.323369	1.586952	3.474724
C	4.306125	3.405442	2.017969
H	4.754786	1.325277	1.920472
H	3.020811	0.538213	3.525116
H	4.089042	1.758118	4.237391

atom	x [Å]	y [Å]	z [Å]
H	2.454647	2.199421	3.735169
H	4.720857	3.642653	1.035166
H	3.465016	4.081299	2.203713
H	5.072006	3.612433	2.771713
C	2.523746	-0.574137	-2.145270
C	2.196469	-0.016736	-3.533547
C	1.791961	-1.897956	-1.894639
H	3.595958	-0.784672	-2.126484
H	2.704535	0.935246	-3.709205
H	2.516552	-0.719841	-4.308163
H	1.122144	0.145951	-3.664310
H	2.077636	-2.320135	-0.928523
H	0.706414	-1.751618	-1.895303
H	2.033703	-2.626291	-2.675006
P	-3.502128	0.815333	0.919675
C	-2.006417	0.045835	1.557004
C	-1.446458	0.570466	2.723071
C	-1.364043	-0.994223	0.882716
C	-0.251746	0.056441	3.211044
H	-1.944746	1.380455	3.245564
C	-0.171732	-1.504880	1.376507
H	-1.792548	-1.406647	-0.023994
C	0.385526	-0.979419	2.537612
H	0.187086	0.471817	4.111759
H	0.331022	-2.306127	0.847370
H	1.328831	-1.369790	2.902552
C	-3.038205	1.933482	-0.425739
C	-2.508337	1.440942	-1.620846
C	-3.154028	3.310172	-0.234936
C	-2.097620	2.318204	-2.614790
H	-2.402069	0.371767	-1.773736
C	-2.735171	4.187282	-1.229524
H	-3.568116	3.690791	0.692323
C	-2.206440	3.692015	-2.416787
H	-1.684131	1.931465	-3.539879
H	-2.821341	5.257492	-1.076652
H	-1.877613	4.377210	-3.190707
O	-4.171975	-0.452641	0.170201
O	-4.325988	1.499169	1.957087
C	-5.337550	-0.328500	-0.666617
C	-5.135481	-1.383556	-1.763937
H	-5.313695	0.648151	-1.160186
C	-4.853982	-2.804317	-1.301858
H	-4.306092	-1.050042	-2.387168
C	-5.888756	-3.464806	-0.395496
H	-3.891753	-2.769016	-0.779971
H	-4.696738	-3.417546	-2.193500
C	-6.077248	-2.775993	0.962297
H	-6.853054	-3.539774	-0.909384
H	-5.555078	-4.491567	-0.218739
H	-6.534750	-3.486179	1.655492
H	-5.095693	-2.525559	1.378090
Cl	-6.573001	-1.319201	-2.876223
C	-6.943582	-1.539135	0.913458
C	-6.610145	-0.412975	0.145668
C	-8.131939	-1.509983	1.642956
H	-8.403121	-2.374825	2.241439
C	-8.978101	-0.406000	1.620447

atom	x [Å]	y [Å]	z [Å]
H	-9.896011	-0.416498	2.199090
C	-8.642498	0.703045	0.856497
H	-9.290466	1.572448	0.828914
C	-7.458879	0.691178	0.128322
H	-7.187736	1.560776	-0.463282

NpMI•− + 1d converged preassembly orientation **4**

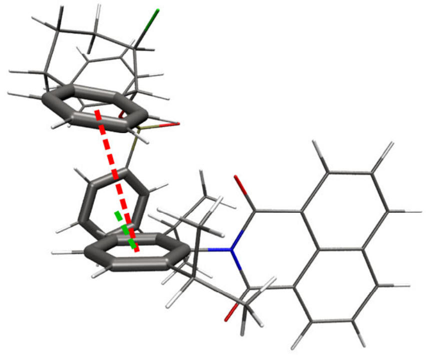

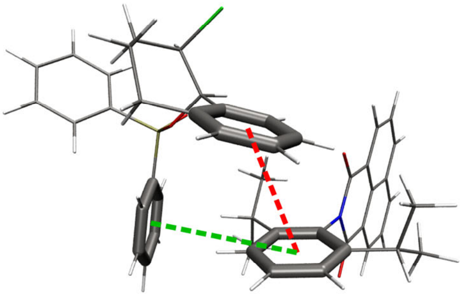

N-aniline to O-P(O)-Ph: *T*-π interaction (centroid-to-centroid) = 5.65 Å.
N-aniline to O-CHR-Ar: π-π interaction (centroid-to-centroid) = 4.41 Å.

99 atoms

G = -2974.650236 E_h

Charge = -1; Multiplicity = 2

atom	x [Å]	y [Å]	z [Å]
C	-6.854998	2.549081	-2.193314
C	-5.893810	1.581212	-2.567066
C	-5.700094	1.167464	-3.905213
C	-7.017130	2.920983	-0.859378
C	-5.079415	0.990665	-1.541272
C	-5.259968	1.388514	-0.194995
C	-6.238604	2.353070	0.134949
C	-4.459338	0.793875	0.844778
C	-3.284687	-0.578975	-0.876274
C	-4.116956	0.014713	-1.893190
C	-3.957064	-0.369594	-3.243301
C	-4.742818	0.206176	-4.227264
H	-4.612782	-0.093097	-5.263230
H	-3.210375	-1.114877	-3.490817
H	-7.764447	3.664802	-0.598622
H	-6.364355	2.637981	1.172919
H	-6.311526	1.611821	-4.685578
H	-7.472755	3.000148	-2.964792
O	-4.595730	1.045841	2.050362
O	-2.422729	-1.436914	-1.106971
N	-3.482292	-0.123989	0.435445
C	-2.646268	-0.681662	1.469488
C	-3.021762	-1.882395	2.089691
C	-1.475589	0.007289	1.830930
C	-2.196620	-2.374444	3.104643

atom	x [Å]	y [Å]	z [Å]
C	-0.687418	-0.536805	2.846062
C	-1.041604	-1.714205	3.485735
H	-2.469754	-3.303664	3.597579
H	0.222954	-0.021108	3.136734
P	3.096214	1.583537	-0.421287
C	2.556562	2.282644	1.148508
C	2.006107	3.564819	1.150759
C	2.657334	1.563306	2.341506
C	1.552326	4.121094	2.340228
H	1.912339	4.118876	0.222580
C	2.206728	2.126798	3.528488
H	3.057285	0.555456	2.337263
C	1.651209	3.402641	3.527072
H	1.109398	5.110919	2.338124
H	2.276448	1.563794	4.452886
H	1.288354	3.835510	4.453176
C	4.874793	1.860414	-0.600003
C	5.746314	1.690804	0.478280
C	5.382976	2.156386	-1.864554
C	7.116879	1.806389	0.287474
H	5.361088	1.464245	1.467405
C	6.756185	2.269346	-2.052657
H	4.703253	2.293463	-2.698816
C	7.621843	2.091344	-0.978786
H	7.791917	1.673068	1.125706
H	7.149209	2.496232	-3.037680
H	8.693026	2.177888	-1.126546
O	2.980066	0.002798	-0.091861
O	2.332193	2.036955	-1.618504
C	2.567954	-0.989398	-1.049960
C	3.727974	-1.248722	-2.028599
H	1.747449	-0.569557	-1.636771
C	5.083793	-1.609491	-1.441948
H	3.844368	-0.353802	-2.641920
C	5.150761	-2.817175	-0.513517
H	5.433019	-0.726029	-0.897825
H	5.778285	-1.741453	-2.276489
C	4.321723	-2.668461	0.768431
H	4.851237	-3.726990	-1.044613
H	6.200365	-2.954227	-0.236608
H	4.705167	-3.363321	1.519817
H	4.466661	-1.661443	1.174522
Cl	3.170043	-2.510904	-3.216697
C	2.847717	-2.941026	0.579663
C	2.040729	-2.176043	-0.277937
C	2.260663	-4.005947	1.262564
H	2.874967	-4.606460	1.927561
C	0.912816	-4.318363	1.111734
H	0.485941	-5.152212	1.660154
C	0.121684	-3.553446	0.268357
H	-0.936028	-3.758169	0.153278
C	0.690237	-2.485804	-0.415929
H	0.052369	-1.881268	-1.053144
C	-4.216460	-2.768832	1.730181
H	-4.474976	-3.274278	2.667933
C	-3.781423	-3.863605	0.744935
H	-3.434871	-3.421037	-0.191565
H	-2.967619	-4.466044	1.158909

atom	x [Å]	y [Å]	z [Å]
H	-4.618927	-4.534496	0.528311
C	-5.509946	-2.104390	1.250101
H	-5.786903	-1.250916	1.871104
H	-5.451338	-1.770551	0.211767
H	-6.319967	-2.837965	1.305712
C	-0.978489	1.319113	1.224740
H	0.057349	1.392392	1.565122
C	-0.916515	1.388055	-0.303576
H	-0.544175	0.457520	-0.736482
H	-1.890572	1.600796	-0.750321
H	-0.233826	2.189139	-0.598451
C	-1.693766	2.549921	1.793403
H	-2.738474	2.595357	1.483078
H	-1.668259	2.552017	2.887017
H	-1.191929	3.457470	1.444945
H	-0.414258	-2.119092	4.273712

5d•− + 1d converged preassembly orientation **1**

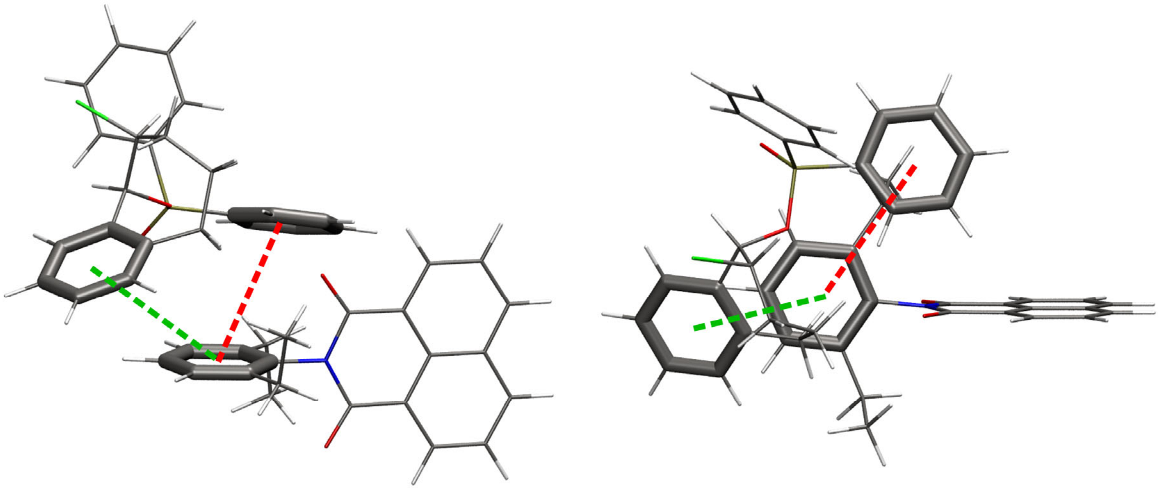

N-aniline to O-CHR-Ar: undefined (centroid-to-centroid) = 4.52 Å.
N-aniline to O-P(O)-Ph: undefined (centroid-to-centroid) = 5.37 Å.

93 atoms

G = -2896.089491 E_h

Charge = -1; Multiplicity = 2

atom	x [Å]	y [Å]	z [Å]
C	-4.932296	-2.231602	3.067801
C	-6.165537	-2.268183	2.418835
C	-6.394252	-1.509618	1.246994
C	-5.322330	-0.704949	0.729322
C	-4.077503	-0.690173	1.402931
C	-3.896528	-1.453985	2.577899
H	-8.446225	-2.113741	0.950644
H	-4.784172	-2.819162	3.969202
H	-6.971787	-2.880455	2.812951
C	-7.632752	-1.505828	0.564757
C	-5.534206	0.067981	-0.438395

atom	x [Å]	y [Å]	z [Å]
H	-2.938850	-1.418825	3.084209
C	-6.790684	0.046519	-1.084623
C	-7.817361	-0.735098	-0.582517
H	-6.932010	0.645995	-1.976287
H	-8.779042	-0.748932	-1.087167
O	-4.575355	1.602290	-1.970459
O	-1.876700	0.181427	1.425709
C	-4.465327	0.876237	-0.971263
C	-2.988725	0.103825	0.889179
N	-3.237779	0.809159	-0.299459
C	-2.120340	1.505059	-0.885676
C	-0.176364	1.471534	-2.284784
C	-0.772197	3.469999	-1.094202
H	0.496170	0.934949	-2.948017
H	-0.569423	4.505205	-0.835352
C	-1.887448	2.839763	-0.547173
C	-1.295391	0.811702	-1.776090
C	-0.868366	-2.743386	0.512944
C	0.264695	-1.970623	0.283031
C	1.125288	-2.298815	-0.762540
C	0.845834	-3.399778	-1.578970
C	-0.286581	-4.166533	-1.345400
C	-1.142529	-3.838617	-0.296015
H	-1.547828	-2.464621	1.309654
H	0.450939	-1.098688	0.896666
H	1.507014	-3.654198	-2.401975
H	-0.504340	-5.016659	-1.982757
H	-2.031497	-4.434551	-0.116904
P	2.613941	-1.360723	-1.132990
C	4.031848	-2.340487	-0.574244
C	3.955501	-3.108780	0.590394
C	5.231170	-2.271572	-1.282690
C	5.073949	-3.794483	1.044963
H	3.023224	-3.171335	1.144013
C	6.350426	-2.957892	-0.823745
H	5.285494	-1.682653	-2.192152
C	6.272287	-3.715851	0.339355
H	5.012010	-4.390430	1.948880
H	7.282531	-2.900060	-1.375100
H	7.145835	-4.250630	0.696769
O	2.495793	-0.148722	-0.065752
O	2.772279	-0.929301	-2.552824
C	3.261694	2.154248	0.003261
C	2.222050	2.730715	0.750546
C	4.014846	2.937576	-0.867412
C	2.006412	4.100874	0.621221
C	3.768620	4.299463	-1.001811
H	4.809005	2.476424	-1.447379
C	2.763475	4.883636	-0.243332
H	1.212489	4.563787	1.199274
H	4.362136	4.895374	-1.686875
H	2.561743	5.946748	-0.326567
C	1.308643	1.912345	1.632350
H	0.628856	2.600239	2.138201
H	0.672159	1.304326	0.985241
C	1.992731	1.010784	2.694505
H	1.630229	1.299667	3.683827
H	1.687255	-0.030062	2.556074

atom	x [Å]	y [Å]	z [Å]
C	3.521222	1.085859	2.733362
H	3.841291	2.131746	2.742167
H	3.888217	0.635801	3.659199
C	3.637116	0.704552	0.166605
H	4.413724	0.461429	-0.561705
Cl	5.969200	0.705957	1.585057
C	4.179216	0.363328	1.568662
H	4.111052	-0.714922	1.700347
C	0.091087	2.788936	-1.941095
H	0.978681	3.284363	-2.322682
C	-2.787115	3.595593	0.400724
H	-3.790278	3.163746	0.390079
H	-2.888975	4.624289	0.040088
C	-1.603732	-0.592907	-2.232466
H	-2.132399	-1.141460	-1.451215
H	-0.663550	-1.124212	-2.395177
C	-2.256544	3.614665	1.836887
H	-2.134087	2.598647	2.217864
H	-1.283344	4.112057	1.886001
H	-2.942115	4.154267	2.496528
C	-2.424245	-0.623612	-3.525461
H	-3.379224	-0.109229	-3.399077
H	-1.884224	-0.133162	-4.341364
H	-2.624448	-1.655678	-3.828162

5d$^{\bullet-}$ + 1d converged preassembly orientation **2**

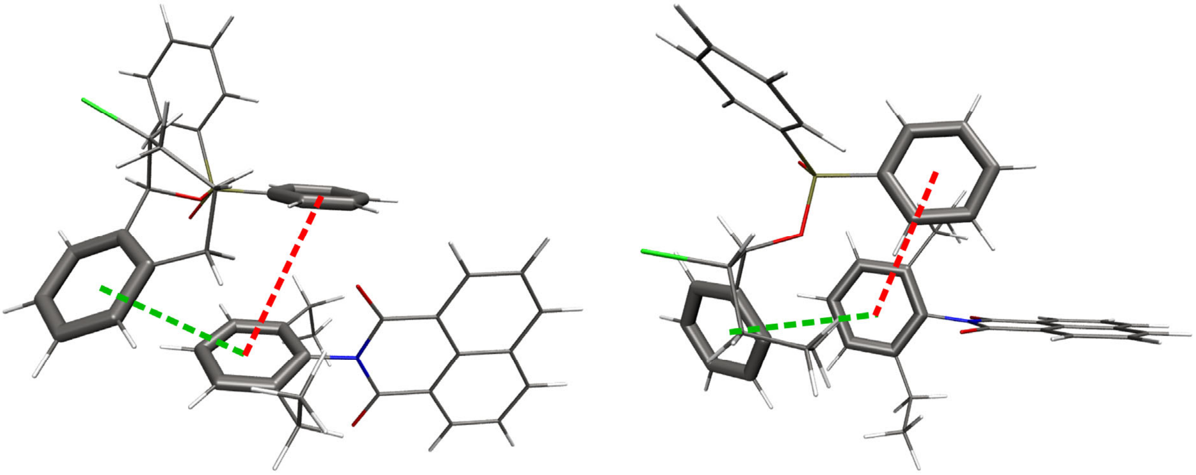

N-aniline to O-CHR-Ar: undefined (centroid-to-centroid) = 4.63 Å.
N-aniline to O-P(O)-Ph: undefined (centroid-to-centroid) = 5.23 Å.

93 atoms

G = -2896.089990 E_h

Charge = -1; Multiplicity = 2

atom	x [Å]	y [Å]	z [Å]
C	-5.233145	-2.195514	3.032974
C	-6.452131	-2.182230	2.356499
C	-6.626254	-1.409869	1.184388
C	-5.514108	-0.642308	0.696280
C	-4.286960	-0.673093	1.401143

atom	x [Å]	y [Å]	z [Å]
C	-4.159400	-1.451732	2.573212
H	-8.691509	-1.937276	0.836791
H	-5.126738	-2.795521	3.932052
H	-7.288765	-2.767276	2.728016
C	-7.847431	-1.358050	0.473145
C	-5.668866	0.139459	-0.474648
H	-3.211813	-1.455626	3.099363
C	-6.909733	0.166870	-1.150255
C	-7.976405	-0.576879	-0.674707
H	-7.007101	0.773266	-2.043170
H	-8.925947	-0.553062	-1.201490
O	-4.608834	1.619462	-1.993716
O	-2.066054	0.140298	1.495916
C	-4.555797	0.901028	-0.984420
C	-3.161364	0.090461	0.922307
N	-3.350852	0.794309	-0.277462
C	-2.188661	1.425881	-0.848049
C	-0.217545	1.265051	-2.200022
C	-0.736486	3.314829	-1.061515
H	0.442312	0.682188	-2.836477
H	-0.486083	4.342964	-0.816621
C	-1.892276	2.751288	-0.524934
C	-1.379096	0.673217	-1.703910
C	-0.788392	-2.664794	0.584505
C	0.348663	-1.908870	0.321969
C	1.197691	-2.272220	-0.722182
C	0.902959	-3.394714	-1.502876
C	-0.231365	-4.147771	-1.234448
C	-1.075743	-3.782728	-0.188425
H	-1.460339	-2.351826	1.374979
H	0.554687	-1.026586	0.914419
H	1.554820	-3.678537	-2.323686
H	-0.459844	-5.015671	-1.843434
H	-1.967087	-4.366883	0.015743
P	2.695738	-1.362416	-1.127236
C	4.105443	-2.347478	-0.557679
C	4.030918	-3.079781	0.630091
C	5.297775	-2.316952	-1.280438
C	5.144454	-3.767452	1.093504
H	3.103634	-3.113565	1.194567
C	6.412077	-3.005544	-0.812807
H	5.350698	-1.755896	-2.207407
C	6.336039	-3.727007	0.373413
H	5.083860	-4.335177	2.015487
H	7.338844	-2.977697	-1.375374
H	7.205903	-4.263141	0.737709
O	2.601837	-0.128923	-0.083632
O	2.845922	-0.962351	-2.557005
C	3.405452	2.160113	-0.054173
C	2.354271	2.763281	0.655480
C	4.187706	2.917128	-0.921622
C	2.151607	4.131037	0.491876
C	3.956222	4.278189	-1.090772
H	4.991167	2.436892	-1.472517
C	2.937215	4.887546	-0.371532
H	1.348550	4.612519	1.041553
H	4.571346	4.854242	-1.773706
H	2.747484	5.950125	-0.483928

atom	x [Å]	y [Å]	z [Å]
C	1.422407	1.958951	1.529147
H	0.681251	2.636395	1.955838
H	0.865717	1.287522	0.873636
C	2.088631	1.147366	2.671889
H	1.746696	1.544629	3.630628
H	1.747073	0.108578	2.638520
C	3.619046	1.177720	2.699814
H	3.967567	2.213882	2.662933
H	3.973255	0.759618	3.645395
C	3.754061	0.708035	0.148425
H	4.539018	0.433922	-0.559485
Cl	6.063255	0.663111	1.603657
C	4.261542	0.389985	1.569301
H	4.148580	-0.679985	1.733858
C	0.107073	2.575709	-1.878842
H	1.024915	3.019366	-2.252772
C	-2.758881	3.563323	0.406366
H	-3.769550	3.150782	0.435262
H	-2.849126	4.579193	0.008031
C	-1.746389	-0.724994	-2.135635
H	-2.336582	-1.220592	-1.362990
H	-0.831503	-1.311718	-2.244175
C	-2.192196	3.624689	1.827695
H	-2.064317	2.619173	2.234499
H	-1.216478	4.119564	1.837163
H	-2.858308	4.186534	2.488620
C	-2.514893	-0.746259	-3.460366
H	-3.445153	-0.178986	-3.385534
H	-1.917140	-0.306854	-4.264941
H	-2.758817	-1.773658	-3.746247

5e$^{\bullet-}$ + 1d converged preassembly orientation **1**

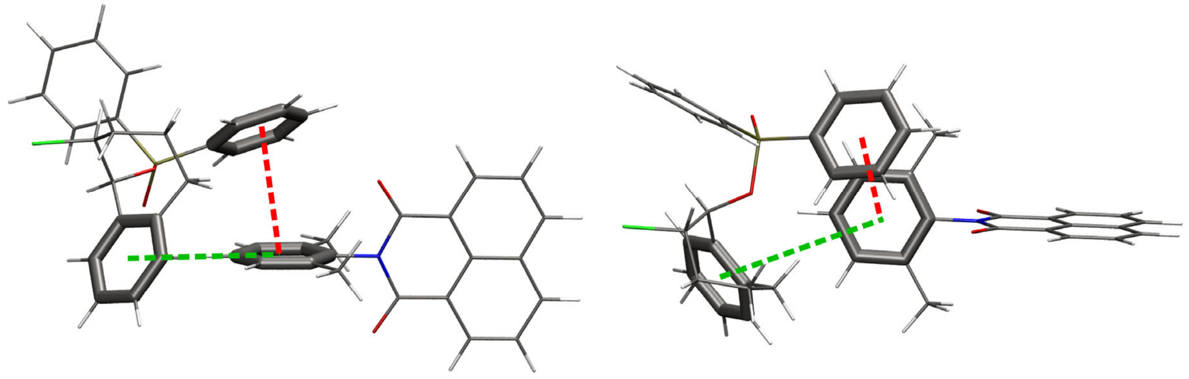

N-aniline to O-CHR-Ar: *T*-π interaction (centroid-to-centroid) = 5.16 Å.
N-aniline to O-P(O)-Ph: π-π interaction (centroid-to-centroid) = 4.33 Å.

87 atoms

G = -2817.524038 E_h

Charge = -1; Multiplicity = 2

atom	x [Å]	y [Å]	z [Å]
C	-7.325311	-1.823169	2.480765

atom	x [Å]	y [Å]	z [Å]
C	-8.331393	-1.365758	1.630947
C	-8.025840	-0.565221	0.505796
C	-6.651461	-0.231027	0.253025
C	-5.648035	-0.709393	1.130332
C	-5.998581	-1.505552	2.243798
H	-10.055091	-0.329094	-0.194521
H	-7.587032	-2.435795	3.338562
H	-9.369020	-1.621109	1.826644
C	-9.014546	-0.078541	-0.380687
C	-6.326787	0.572614	-0.867204
H	-5.214285	-1.859255	2.902848
C	-7.347549	1.039115	-1.726160
C	-8.669355	0.710947	-1.476764
H	-7.077160	1.653840	-2.576796
H	-9.448092	1.072558	-2.141918
O	-4.579471	1.619062	-2.082983
O	-3.325373	-0.753648	1.606955
C	-4.952322	0.916979	-1.131143
C	-4.264739	-0.382060	0.888711
N	-3.999020	0.408872	-0.236988
C	-2.622559	0.738267	-0.501436
C	-0.569333	0.274264	-1.635197
C	-0.747528	2.185226	-0.183770
H	0.021915	-0.342620	-2.303982
H	-0.295070	3.058634	0.275612
C	-2.074876	1.870512	0.100651
C	-1.891692	-0.075512	-1.366539
C	-0.006378	-2.589257	1.359592
C	1.121408	-1.908754	0.919051
C	1.588362	-2.107365	-0.380514
C	0.926764	-2.993074	-1.232743
C	-0.198853	-3.672536	-0.786525
C	-0.667151	-3.467643	0.507137
H	-0.377620	-2.423577	2.364679
H	1.626508	-1.211996	1.577455
H	1.278464	-3.137083	-2.249287
H	-0.719919	-4.350329	-1.453667
H	-1.554995	-3.988155	0.850007
P	3.025144	-1.239350	-1.022348
C	4.516988	-2.198663	-0.660701
C	4.636818	-2.920219	0.529945
C	5.590769	-2.141913	-1.549316
C	5.824656	-3.573822	0.829264
H	3.805167	-2.972506	1.226074
C	6.780178	-2.795441	-1.244818
H	5.493279	-1.588194	-2.477025
C	6.897458	-3.508051	-0.056592
H	5.914554	-4.134403	1.753228
H	7.614474	-2.747126	-1.936032
H	7.825636	-4.017127	0.180268
O	3.090263	0.005950	0.010547
O	2.947363	-0.857917	-2.462408
C	3.602191	2.350597	-0.149095
C	2.691949	2.820847	0.813727
C	4.015784	3.181434	-1.185566
C	2.237876	4.133024	0.699458
C	3.544481	4.486133	-1.288887
H	4.713124	2.802505	-1.926852

atom	x [Å]	y [Å]	z [Å]
C	2.652318	4.962206	-0.338906
H	1.539698	4.515185	1.438613
H	3.872663	5.120235	-2.105345
H	2.274857	5.977532	-0.402045
C	2.163261	1.934499	1.918499
H	1.490580	2.530346	2.539206
H	1.540397	1.168023	1.453519
C	3.225750	1.259630	2.827054
H	3.120594	1.652793	3.840991
H	3.022892	0.186897	2.899662
C	4.686422	1.470471	2.420976
H	4.882605	2.538399	2.288348
H	5.337812	1.129009	3.229243
C	4.169939	0.956702	-0.067832
H	4.752999	0.758149	-0.969705
Cl	6.777555	1.203371	0.694697
C	5.079690	0.727112	1.154528
H	5.138309	-0.345110	1.335937
C	0.001018	1.395635	-1.047361
H	1.036342	1.649965	-1.251686
C	-2.901096	2.721340	1.025854
H	-3.793454	3.103489	0.521747
H	-3.241580	2.146363	1.891676
H	-2.326308	3.574763	1.390273
C	-2.524272	-1.287609	-1.992699
H	-2.882021	-1.983778	-1.229086
H	-3.383583	-1.009892	-2.609991
H	-1.808896	-1.820105	-2.621166

5e$^{\bullet-}$ + 1d converged preassembly orientation **2**

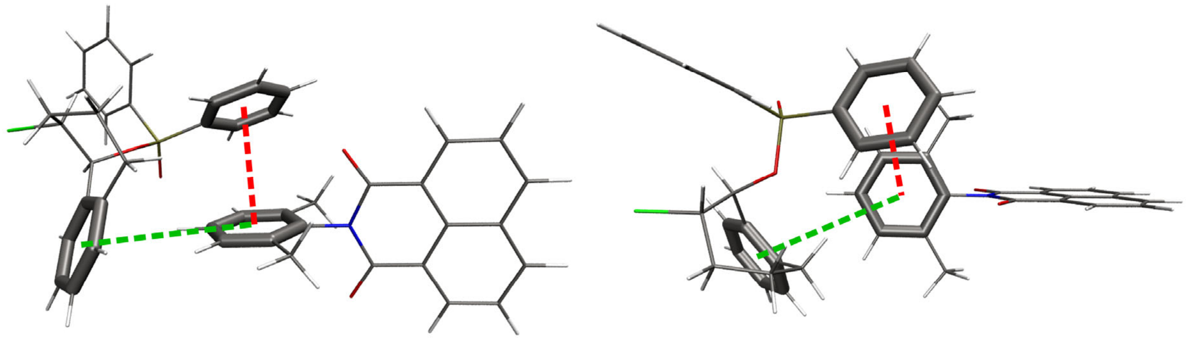

N-aniline to O-CHR-Ar: *T*-π interaction (centroid-to-centroid) = 5.17 Å.

N-aniline to O-P(O)-Ph: π-π interaction (centroid-to-centroid) = 4.32 Å.

87 atoms

G = -2817.523682 E_h

Charge = -1; Multiplicity = 2

atom	x [Å]	y [Å]	z [Å]
C	-7.327370	-1.826134	2.480545
C	-8.333958	-1.365259	1.633208

atom	x [Å]	y [Å]	z [Å]
C	-8.028732	-0.563039	0.509165
C	-6.654160	-0.230771	0.254930
C	-5.650221	-0.712561	1.129791
C	-6.000450	-1.510343	2.242189
H	-10.058645	-0.321899	-0.187528
H	-7.588835	-2.440017	3.337524
H	-9.371730	-1.619170	1.830012
C	-9.017939	-0.072858	-0.374831
C	-6.329766	0.574414	-0.864287
H	-5.215772	-1.866705	2.899353
C	-7.351022	1.044403	-1.720742
C	-8.673032	0.718158	-1.469892
H	-7.080877	1.660342	-2.570578
H	-9.452153	1.082503	-2.133104
O	-4.582410	1.619714	-2.081009
O	-3.326957	-0.761327	1.603071
C	-4.955086	0.916782	-1.129721
C	-4.266755	-0.386936	0.886825
N	-4.001388	0.405768	-0.237674
C	-2.624817	0.734017	-0.502997
C	-0.572940	0.268987	-1.638752
C	-0.747917	2.178463	-0.185054
H	0.016862	-0.347968	-2.308729
H	-0.294022	3.050701	0.275094
C	-2.075444	1.864988	0.099936
C	-1.895464	-0.079641	-1.369479
C	-0.004695	-2.590190	1.351945
C	1.124190	-1.909824	0.913994
C	1.593157	-2.107257	-0.385050
C	0.932424	-2.991580	-1.239373
C	-0.194413	-3.670781	-0.795814
C	-0.664640	-3.467135	0.497345
H	-0.377471	-2.425486	2.356623
H	1.628642	-1.214101	1.573996
H	1.285750	-3.134627	-2.255485
H	-0.714823	-4.347475	-1.464582
H	-1.553373	-3.987490	0.838149
P	3.031364	-1.239181	-1.023544
C	4.522697	-2.197536	-0.657292
C	4.639315	-2.919200	0.533607
C	5.599183	-2.140004	-1.542602
C	5.826615	-3.572164	0.836460
H	3.805581	-2.972074	1.227189
C	6.788035	-2.792879	-1.234557
H	5.504258	-1.586198	-2.470525
C	6.902089	-3.505628	-0.046092
H	5.914002	-4.132859	1.760595
H	7.624417	-2.743967	-1.923205
H	7.829824	-4.014227	0.193511
O	3.092966	0.006897	0.008702
O	2.957350	-0.858648	-2.464028
C	3.600307	2.352492	-0.149164
C	2.689129	2.820155	0.814035
C	4.012366	3.185066	-1.184861
C	2.232661	4.131617	0.701011
C	3.538655	4.488991	-1.286970
H	4.710441	2.808130	-1.926470
C	2.645625	4.962553	-0.336536

atom	x [Å]	y [Å]	z [Å]
H	1.533869	4.511832	1.440604
H	3.865702	5.124461	-2.102822
H	2.266374	5.977275	-0.398678
C	2.162041	1.931885	1.918040
H	1.488348	2.526018	2.539299
H	1.540484	1.164738	1.452428
C	3.225671	1.257992	2.825975
H	3.119520	1.649700	3.840374
H	3.024938	0.184769	2.897240
C	4.685982	1.472281	2.420457
H	4.879987	2.540764	2.289091
H	5.337911	1.131297	3.228496
C	4.170893	0.959694	-0.068921
H	4.754306	0.762893	-0.970955
Cl	6.777806	1.212203	0.694013
C	5.081136	0.731158	1.153271
H	5.142462	-0.341096	1.333630
C	-0.000826	1.388997	-1.050021
H	1.034696	1.642377	-1.254607
C	-2.899997	2.715692	1.026737
H	-3.791924	3.100043	0.523534
H	-3.241097	2.139955	1.891813
H	-2.323728	3.567647	1.392259
C	-2.529688	-1.290614	-1.996128
H	-2.886318	-1.987590	-1.232714
H	-3.390018	-1.011799	-2.611486
H	-1.815663	-1.822610	-2.626567

^{n}BuO-NpMI$^{\bullet-}$ + 1d converged preassembly orientation **1**

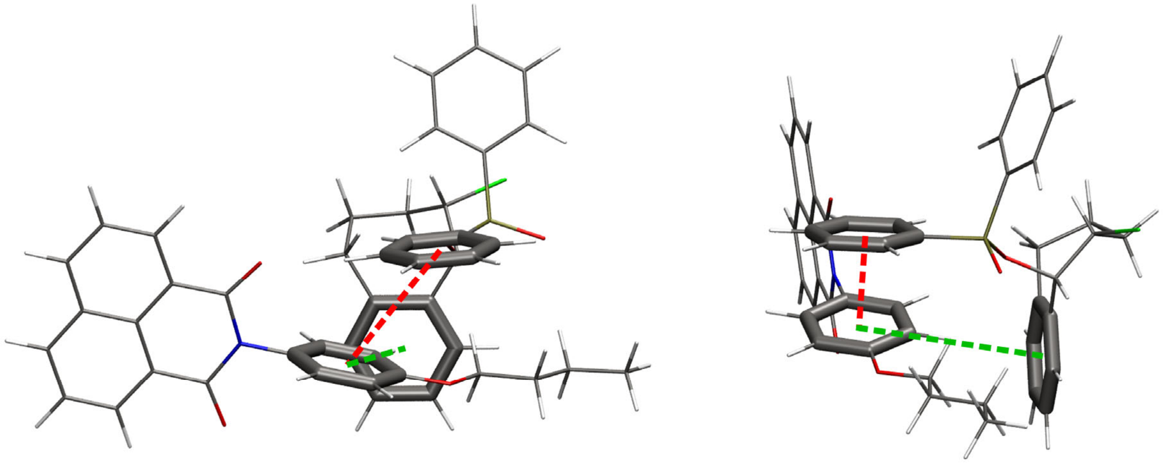

N-aniline to O-CHR-Ar: *T*-π interaction (centroid-to-centroid) = 4.90 Å.

N-aniline to O-P(O)-Ph: π-π interaction (centroid-to-centroid) = 4.07 Å.

94 atoms

G = -2971.296269 E_h

Charge = -1; Multiplicity = 2

atom	x [Å]	y [Å]	z [Å]
C	-2.084854	-4.431159	2.332506
C	-2.966601	-3.473244	2.831262
C	-2.627745	-2.100078	2.827429
C	-1.350988	-1.708448	2.297244
C	-0.473273	-2.700730	1.796305
C	-0.852884	-4.062022	1.819548
H	-4.457790	-1.386640	3.724620
H	-2.369430	-5.479191	2.346923
H	-3.930742	-3.774371	3.231090
C	-3.491389	-1.095749	3.322682
C	-0.994781	-0.337966	2.284982
H	-0.165962	-4.804643	1.430726
C	-1.889240	0.632575	2.790148
C	-3.117761	0.247133	3.299695
H	-1.596934	1.675990	2.770895
H	-3.799679	0.998865	3.686250
O	0.670906	1.246348	1.704137
O	1.637327	-3.126903	0.804963
C	0.282841	0.070296	1.754422
C	0.810624	-2.322946	1.261434
N	1.113881	-0.953004	1.272470
C	2.392047	-0.562691	0.744779
C	3.773494	0.055397	-1.125484
C	4.724064	-0.058057	1.088732
C	4.877635	0.165737	-0.277674
H	3.904447	0.232044	-2.187543
H	5.558216	0.027836	1.771165
C	3.481028	-0.430195	1.588092
H	3.365188	-0.618800	2.650700
C	2.539882	-0.309067	-0.614520
H	1.684353	-0.409449	-1.274372
O	6.061187	0.471020	-0.866338
C	4.569494	-3.231045	-0.823213
C	5.553039	-3.213635	0.156028
C	6.902634	-3.218064	-0.204667
C	7.264318	-3.234849	-1.552546
C	6.277188	-3.250174	-2.531604
C	4.934324	-3.251198	-2.166253
H	3.526731	-3.191221	-0.524632
H	5.265645	-3.161338	1.199719
H	8.311963	-3.224324	-1.835385
H	6.557834	-3.254629	-3.579461
H	4.167181	-3.253290	-2.933888
P	8.208241	-3.232106	1.033136
C	8.414016	-4.925390	1.638763
C	7.334063	-5.636445	2.169498
C	9.675117	-5.516420	1.585753
C	7.520283	-6.924672	2.651605
H	6.347466	-5.186013	2.215708
C	9.857980	-6.809242	2.065586
H	10.510995	-4.959290	1.177344
C	8.783162	-7.510942	2.599724
H	6.681693	-7.472055	3.067798
H	10.841066	-7.265604	2.025780
H	8.927082	-8.517916	2.976818
O	7.450580	-2.438676	2.224478
O	9.504384	-2.636614	0.597022
C	7.214550	0.533723	-0.034105

atom	x [Å]	y [Å]	z [Å]
H	7.287923	-0.381962	0.561025
H	7.116761	1.377323	0.660257
C	7.366277	-0.610495	3.725223
C	6.095309	-0.680690	4.316593
C	7.919949	0.628395	3.418730
C	5.417224	0.503203	4.590187
C	7.227207	1.805083	3.685496
H	8.898385	0.676801	2.950133
C	5.971204	1.740316	4.273206
H	4.434235	0.456214	5.049744
H	7.667024	2.762945	3.429444
H	5.418749	2.649940	4.484039
C	5.508409	-2.017965	4.678560
H	4.443128	-1.915927	4.897995
H	5.583034	-2.690637	3.818909
C	6.219675	-2.646966	5.888483
H	5.876336	-2.163352	6.807668
H	5.926429	-3.699274	5.955842
C	7.749824	-2.547186	5.842012
H	8.082255	-1.539631	6.107869
H	8.167800	-3.224859	6.588576
C	8.136258	-1.857753	3.352168
H	9.127501	-1.549164	3.017753
Cl	10.079354	-3.322625	4.630900
C	8.304015	-2.920813	4.474099
H	7.854630	-3.854274	4.138343
C	8.439932	0.686184	-0.905724
H	8.508096	-0.180075	-1.574153
H	8.333162	1.574560	-1.538782
C	9.707966	0.790032	-0.061026
H	9.639235	1.667661	0.593254
H	9.772463	-0.088772	0.589128
C	10.969676	0.884305	-0.912041
H	11.865503	0.962324	-0.289975
H	11.081223	-0.000621	-1.546609
H	10.941922	1.760570	-1.567680

^{n}BuO-NpMI$^{\bullet-}$ + 1d converged preassembly orientation **2**

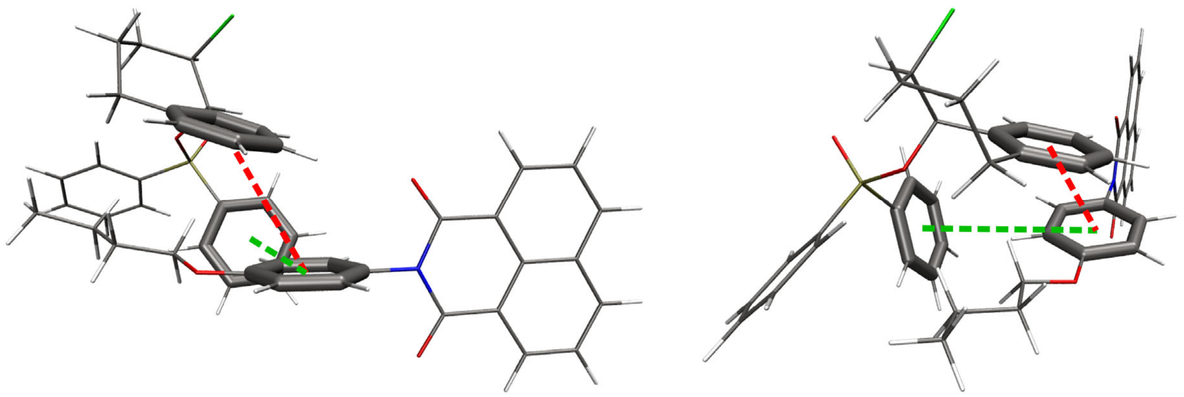

N-aniline to O-P(O)Ph: *T*-π interaction (centroid-to-centroid) = 5.14 Å.
N-aniline to O-CHR-Ar: π-π interaction (centroid-to-centroid) = 4.26 Å.

94 atoms

G = -2971.300693 E_h

Charge = -1; Multiplicity = 2

atom	x [Å]	y [Å]	z [Å]
C	9.261185	-0.763506	-1.279721
C	8.317961	0.289479	-1.241350
C	8.559580	1.556284	-1.821748
C	8.982808	-1.997609	-0.694122
C	7.059869	0.062181	-0.585218
C	6.801508	-1.200125	0.003232
C	7.773870	-2.224012	-0.058209
C	5.542845	-1.443212	0.664372
C	4.832967	0.890175	0.108304
C	6.102760	1.105218	-0.539942
C	6.387019	2.356636	-1.131883
C	7.602066	2.567785	-1.761227
H	7.813039	3.532157	-2.214081
H	5.640684	3.140979	-1.083931
H	9.724238	-2.790064	-0.737985
H	7.555929	-3.183144	0.396613
H	9.509232	1.732565	-2.319002
H	10.213328	-0.596813	-1.775507
O	5.236858	-2.514079	1.208522
O	3.938367	1.744206	0.189476
N	4.628169	-0.379203	0.672191
C	3.359370	-0.606129	1.305622
C	3.187081	-0.311533	2.653477
C	2.295953	-1.100018	0.570938
C	1.952052	-0.488653	3.254127
C	1.051397	-1.280656	1.162390
C	0.870463	-0.955263	2.504708
H	1.800962	-0.237243	4.298375
H	0.231721	-1.642422	0.557834
H	2.430156	-1.333163	-0.480652
H	4.023147	0.072661	3.228591
O	-0.321407	-1.034500	3.147126
C	-1.480851	-1.255376	2.350670
C	-2.712446	-1.014613	3.193612
H	-1.467916	-2.281973	1.964711
H	-1.473500	-0.568035	1.497043
C	-3.984944	-1.277203	2.391529
H	-2.687164	-1.656435	4.081400
H	-2.701559	0.023158	3.544555
C	-5.247291	-0.896372	3.155438
H	-3.937784	-0.717742	1.450928
H	-4.031537	-2.336892	2.112860
H	-6.145649	-1.107781	2.570332
H	-5.323166	-1.452953	4.094789
H	-5.251374	0.170054	3.402139
P	-3.698918	-0.499353	-1.967689
C	-2.284347	-1.611048	-1.779301
C	-2.286432	-2.646065	-0.841905
C	-1.169095	-1.420505	-2.598101
C	-1.191114	-3.494225	-0.740417
H	-3.145926	-2.801259	-0.198169
C	-0.066340	-2.258333	-2.479113
H	-1.169813	-0.626727	-3.337528
C	-0.081544	-3.300570	-1.558111

atom	x [Å]	y [Å]	z [Å]
H	-1.199976	-4.302391	-0.017203
H	0.799605	-2.103775	-3.113336
H	0.775759	-3.959408	-1.471109
C	-5.173619	-1.375598	-1.414944
C	-6.047141	-0.828866	-0.474886
C	-5.470557	-2.607403	-2.005200
C	-7.207983	-1.510212	-0.128162
H	-5.817769	0.121229	-0.007804
C	-6.628620	-3.286849	-1.652392
H	-4.795913	-3.043208	-2.736001
C	-7.497790	-2.737620	-0.713751
H	-7.885692	-1.081868	0.602119
H	-6.852571	-4.244433	-2.109177
H	-8.402627	-3.268774	-0.438471
O	-3.559622	0.621324	-0.806674
O	-3.764922	0.081007	-3.342329
C	-2.687780	1.755754	-1.014018
C	-3.593567	2.929703	-1.419495
H	-2.041544	1.553691	-1.872406
C	-4.759418	3.254661	-0.499428
H	-3.983283	2.701317	-2.411120
C	-4.440953	3.571780	0.958818
H	-5.430435	2.390078	-0.541131
H	-5.311690	4.085649	-0.946944
C	-3.791946	2.415829	1.729835
H	-3.806952	4.462238	1.025365
H	-5.384956	3.829215	1.448346
H	-3.931998	2.581429	2.801120
H	-4.319952	1.486968	1.492500
Cl	-2.535151	4.387569	-1.682340
C	-2.314586	2.248448	1.465265
C	-1.804450	1.946383	0.193741
C	-1.410533	2.415223	2.512860
H	-1.791084	2.645877	3.503902
C	-0.038765	2.289507	2.322504
H	0.636583	2.411188	3.162974
C	0.461392	1.983801	1.065474
H	1.527153	1.849407	0.907010
C	-0.429206	1.812355	0.012552
H	-0.044714	1.561286	-0.972086

^{n}BuO-NpMI$^{•-}$ + 1d converged preassembly orientation **3**

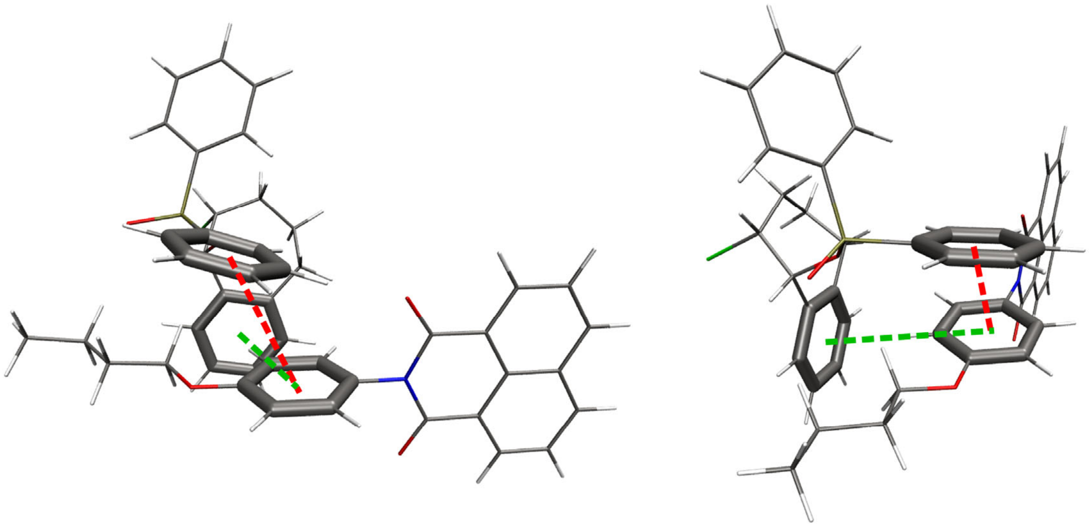

N-aniline to O-P(O)-Ph: *T*-π interaction (centroid-to-centroid) = 4.89 Å.
N-aniline to O-CHR-Ar: π-π interaction (centroid-to-centroid) = 4.06 Å.

94 atoms

G = -2971.300928 E_h

Charge = -1; Multiplicity = 2

atom	x [Å]	y [Å]	z [Å]
C	8.541041	-0.725336	1.126909
C	7.533601	-1.404052	0.402970
C	7.678732	-2.736775	-0.047922
C	8.356827	0.589438	1.552421
C	6.309495	-0.709755	0.113025
C	6.147240	0.625357	0.555640
C	7.180531	1.265518	1.275988
C	4.924577	1.335900	0.271559
C	4.054231	-0.690820	-0.905614
C	5.288646	-1.373720	-0.610383
C	5.476894	-2.706300	-1.042552
C	6.659508	-3.368286	-0.759375
H	6.796353	-4.392161	-1.094900
H	4.683386	-3.195238	-1.595445
H	9.146452	1.087921	2.107015
H	7.034253	2.287627	1.605166
H	8.602294	-3.266185	0.168663
H	9.468246	-1.246021	1.349033
O	4.703327	2.504795	0.618558
O	3.110324	-1.198817	-1.529526
N	3.945072	0.630001	-0.443887
C	2.716508	1.320465	-0.723406
C	2.570510	2.039556	-1.904635
C	1.674329	1.278247	0.185549
C	1.381061	2.690722	-2.182030
C	0.476425	1.932774	-0.078321
C	0.319233	2.623690	-1.277693
H	1.248583	3.233438	-3.111606
H	-0.322979	1.874060	0.647122
H	1.790213	0.726514	1.113027

atom	x [Å]	y [Å]	z [Å]
H	3.391095	2.077222	-2.613627
O	-0.831841	3.236391	-1.652364
C	-1.950008	3.128290	-0.778147
C	-3.164017	3.715339	-1.460910
H	-1.732477	3.654387	0.159601
H	-2.125825	2.074833	-0.536790
C	-4.390976	3.656785	-0.553570
H	-2.956989	4.752145	-1.749915
H	-3.356416	3.155641	-2.383587
C	-5.645350	4.194479	-1.233604
H	-4.558708	2.617802	-0.250999
H	-4.195186	4.229221	0.361303
H	-6.511143	4.148222	-0.567425
H	-5.515209	5.237678	-1.539000
H	-5.884159	3.614087	-2.130370
P	-3.393299	-0.642031	-1.031088
C	-2.109768	-0.337069	-2.251718
C	-2.488370	0.151875	-3.503112
C	-0.764546	-0.597663	-1.980899
C	-1.521812	0.373664	-4.477149
H	-3.531170	0.368817	-3.710830
C	0.197831	-0.381226	-2.957382
H	-0.461406	-0.941426	-0.998813
C	-0.184000	0.103313	-4.204768
H	-1.813673	0.762638	-5.446799
H	1.241318	-0.561054	-2.718984
H	0.567895	0.285212	-4.965880
C	-3.812013	-2.403602	-1.052174
C	-2.828003	-3.383263	-1.204034
C	-5.140391	-2.780245	-0.851243
C	-3.170807	-4.727681	-1.144266
H	-1.792538	-3.102913	-1.368674
C	-5.479869	-4.127203	-0.789193
H	-5.904756	-2.018194	-0.742512
C	-4.495838	-5.099630	-0.933126
H	-2.404048	-5.485460	-1.262147
H	-6.513059	-4.416409	-0.630547
H	-4.761469	-6.150330	-0.885224
O	-2.541456	-0.427852	0.332572
O	-4.605698	0.219580	-1.141810
C	-3.145971	-0.076916	1.590387
C	-3.608824	-1.366801	2.289411
H	-4.040965	0.515327	1.383426
C	-2.585107	-2.478879	2.444511
H	-4.460322	-1.758602	1.732601
C	-1.300054	-2.150292	3.197176
H	-2.332250	-2.806997	1.430965
H	-3.087040	-3.326452	2.919462
C	-0.434551	-1.072376	2.533962
H	-1.524947	-1.859662	4.228823
H	-0.715540	-3.072900	3.258630
H	0.585767	-1.156244	2.916272
H	-0.376383	-1.267369	1.457481
Cl	-4.326790	-0.897635	3.895396
C	-0.910517	0.341866	2.766102
C	-2.167581	0.798901	2.338328
C	-0.068911	1.241889	3.418670
H	0.905639	0.901149	3.755766

atom	x [Å]	y [Å]	z [Å]
C	-0.438733	2.565048	3.636349
H	0.244582	3.241697	4.138619
C	-1.678569	3.012231	3.203183
H	-1.980732	4.042384	3.357977
C	-2.533145	2.124221	2.560323
H	-3.501471	2.473022	2.214056

^{n}BuO-NpMI$^{\bullet-}$ + 1d converged preassembly orientation **4**

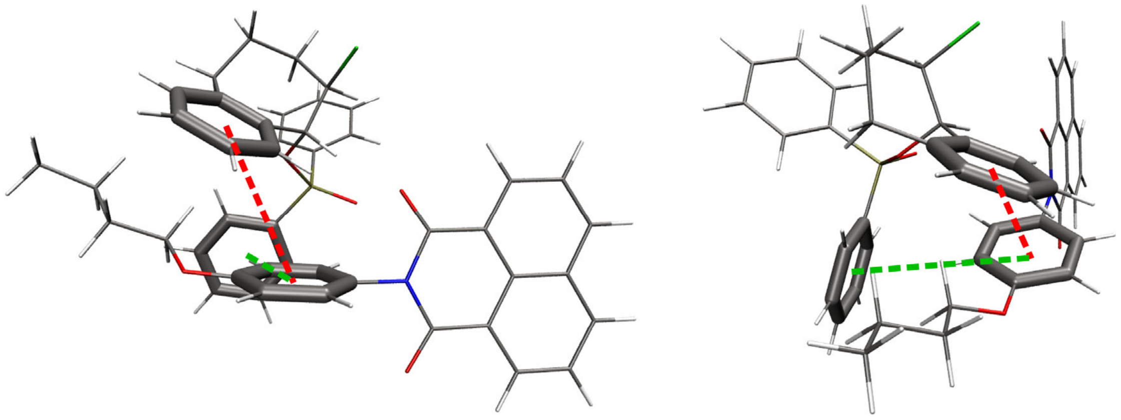

N-aniline to O-P(O)-Ph: *T*-π interaction (centroid-to-centroid) = 5.14 Å.

N-aniline to O-CHR-Ar: π-π interaction (centroid-to-centroid) = 4.33 Å.

94 atoms

G = -2971.299285 E_h

Charge = -1; Multiplicity = 2

atom	x [Å]	y [Å]	z [Å]
C	7.901445	-0.419402	0.535091
C	6.810395	-0.758531	-0.298348
C	6.865362	-1.808013	-1.244860
C	7.803791	0.622482	1.456283
C	5.592941	-0.005031	-0.177489
C	5.517857	1.046796	0.768128
C	6.633172	1.351474	1.580611
C	4.297587	1.802890	0.910338
C	3.265515	0.420253	-0.899162
C	4.491256	-0.329474	-1.006203
C	4.589818	-1.386359	-1.939180
C	5.765748	-2.108972	-2.047596
H	5.834107	-2.920897	-2.765767
H	3.733021	-1.617888	-2.561199
H	8.656656	0.863087	2.084276
H	6.554252	2.160280	2.297686
H	7.783198	-2.381532	-1.339032
H	8.824253	-0.985937	0.447559
O	4.140080	2.725068	1.723677
O	2.259581	0.213303	-1.593418
N	3.241235	1.443864	0.061659
C	2.000709	2.152504	0.206618
C	1.790814	3.358041	-0.452410

atom	x [Å]	y [Å]	z [Å]
C	0.989488	1.601681	0.974022
C	0.556749	3.985530	-0.380336
C	-0.250485	2.221501	1.051012
C	-0.482010	3.397879	0.342342
H	0.368909	4.905037	-0.924062
H	-1.034581	1.761492	1.636863
H	1.154411	0.657109	1.480907
H	2.588490	3.790985	-1.047090
O	-1.698347	4.001188	0.283819
C	-2.829750	3.169316	0.540926
C	-4.075254	3.833879	-0.000481
H	-2.922027	2.991658	1.619111
H	-2.677712	2.203103	0.046441
C	-5.289761	2.919469	0.147992
H	-4.250927	4.783372	0.517995
H	-3.915928	4.065780	-1.058462
C	-6.546415	3.509722	-0.481979
H	-5.067982	1.952048	-0.318440
H	-5.469382	2.715272	1.210324
H	-7.405212	2.844157	-0.360109
H	-6.802983	4.471884	-0.027615
H	-6.403718	3.677428	-1.554300
P	-0.921039	-1.682937	1.330705
C	-1.612508	-0.652652	2.639584
C	-0.872439	-0.477565	3.809012
C	-2.842064	-0.005159	2.488645
C	-1.358430	0.342525	4.821134
H	0.090358	-0.965157	3.920925
C	-3.326897	0.805844	3.506090
H	-3.403904	-0.108888	1.566931
C	-2.584541	0.981884	4.670562
H	-0.775885	0.487186	5.724285
H	-4.278822	1.311316	3.384639
H	-2.960597	1.624108	5.459742
C	-1.667425	-3.328440	1.423361
C	-3.021324	-3.498510	1.721673
C	-0.885225	-4.434907	1.092242
C	-3.588372	-4.765485	1.677310
H	-3.639476	-2.646290	1.985261
C	-1.456825	-5.701606	1.045659
H	0.167870	-4.301967	0.868243
C	-2.807377	-5.866174	1.334506
H	-4.640033	-4.894561	1.908630
H	-0.847019	-6.559449	0.784028
H	-3.252859	-6.854536	1.296398
O	-1.620459	-1.002933	0.045233
O	0.568772	-1.741576	1.298167
C	-1.013521	-0.868646	-1.256691
C	-1.103578	-2.229159	-1.970807
H	0.044745	-0.632630	-1.121250
C	-2.469297	-2.896034	-2.042769
H	-0.412810	-2.910061	-1.470968
C	-3.613140	-2.101486	-2.666072
H	-2.741538	-3.159698	-1.015939
H	-2.345337	-3.844696	-2.572838
C	-3.974130	-0.813813	-1.913274
H	-3.390317	-1.865538	-3.712236
H	-4.490386	-2.755433	-2.678832

atom	x [Å]	y [Å]	z [Å]
H	-4.992443	-0.520697	-2.181668
H	-3.982344	-1.017337	-0.837172
Cl	-0.383558	-2.037642	-3.633302
C	-3.052291	0.343954	-2.214674
C	-1.676276	0.305087	-1.938363
C	-3.572741	1.489591	-2.814627
H	-4.636959	1.536342	-3.028273
C	-2.763249	2.572269	-3.143793
H	-3.199154	3.450936	-3.608692
C	-1.405606	2.524491	-2.866100
H	-0.764100	3.367749	-3.098538
C	-0.870467	1.393787	-2.259352
H	0.187070	1.354810	-2.015078

^{n}BuO-NpMI$^{\bullet-}$ + 1d converged preassembly – naphthalene moiety complex

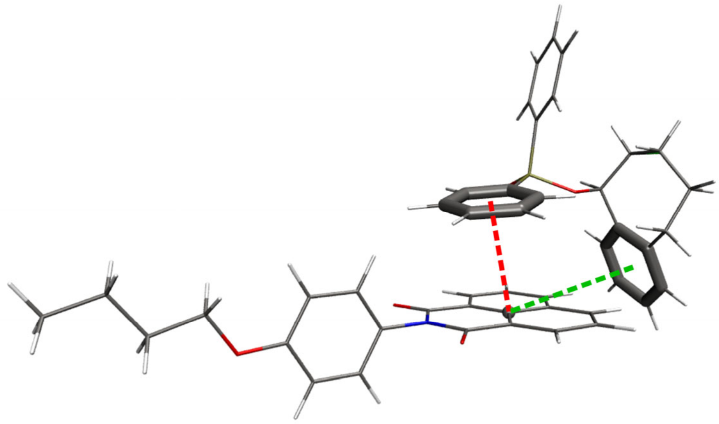

Central C of naphthalene unit to O-CHR-Ar: *T*-π interaction (centroid-to-centroid) = 4.50 Å.
Central C of naphthalene unit to O-P(O)-Ph: π-π interaction (centroid-to-centroid) = 4.52 Å.

94 atoms

G = -2971.300665 E_h

Charge = -1; Multiplicity = 2

atom	x [Å]	y [Å]	z [Å]
C	-1.575442	-2.366693	1.186277
C	-0.809965	-2.511584	0.008457
C	-1.356827	-2.997109	-1.202191
C	-1.003799	-1.868116	2.353181
C	0.578641	-2.148981	0.039344
C	1.135073	-1.643595	1.237462
C	0.331786	-1.504066	2.389123
C	2.523855	-1.259100	1.280471
C	2.752834	-1.931980	-1.117022
C	1.362918	-2.300304	-1.130429
C	0.773927	-2.783729	-2.320577
C	-0.568807	-3.121453	-2.344761
H	-1.014210	-3.491003	-3.264340
H	1.388016	-2.877623	-3.208818
H	-1.617802	-1.752744	3.241568
H	0.776318	-1.104852	3.293359

atom	x [Å]	y [Å]	z [Å]
H	-2.406406	-3.272214	-1.226918
H	-2.627684	-2.632149	1.163908
O	3.089582	-0.796721	2.283155
O	3.511660	-2.022178	-2.096449
N	3.256218	-1.438901	0.098379
C	4.649126	-1.092602	0.135994
C	5.591144	-2.046192	0.509234
C	5.067705	0.183339	-0.195529
C	6.936030	-1.722576	0.548736
C	6.417698	0.524874	-0.160820
C	7.359489	-0.432804	0.213691
H	7.676848	-2.459785	0.838653
H	6.711844	1.532103	-0.426488
H	4.337838	0.929633	-0.487938
H	5.264104	-3.047361	0.769774
O	8.695612	-0.209838	0.282095
C	9.187716	1.085544	-0.042426
C	10.693676	1.072273	0.116442
H	8.735124	1.828880	0.625214
H	8.910172	1.337143	-1.073369
C	11.314758	2.428532	-0.211560
H	10.941862	0.788523	1.145238
H	11.115072	0.301601	-0.538595
C	12.832056	2.426464	-0.054618
H	11.053273	2.711734	-1.237864
H	10.880481	3.196338	0.439437
H	13.257958	3.404645	-0.293015
H	13.121880	2.177597	0.970960
H	13.295957	1.690083	-0.718153
P	-2.530953	1.611333	0.854290
C	-0.990931	1.350177	-0.032150
C	0.195758	1.736004	0.589574
C	-0.963611	0.832270	-1.329372
C	1.402845	1.615786	-0.087954
H	0.179864	2.118046	1.604980
C	0.243803	0.719207	-2.002310
H	-1.880705	0.503639	-1.804139
C	1.425324	1.115307	-1.384173
H	2.324039	1.905369	0.405817
H	0.267357	0.298159	-3.000853
H	2.366906	1.012318	-1.913457
C	-3.228625	3.210040	0.358393
C	-3.168974	3.647970	-0.966943
C	-3.924296	3.962111	1.305456
C	-3.810519	4.820955	-1.341633
H	-2.621297	3.076816	-1.710056
C	-4.567817	5.135689	0.926815
H	-3.964635	3.623308	2.334949
C	-4.514435	5.562291	-0.395719
H	-3.762333	5.157480	-2.371508
H	-5.111315	5.715449	1.664943
H	-5.018387	6.476465	-0.690900
O	-3.477229	0.528286	0.109389
O	-2.463765	1.494199	2.339183
C	-4.833365	0.294997	0.533124
C	-5.758924	1.063836	-0.425851
H	-4.973427	0.726844	1.528007
C	-5.552058	0.834204	-1.912840

atom	x [Å]	y [Å]	z [Å]
H	-5.638031	2.127155	-0.221182
C	-5.683662	-0.596842	-2.423399
H	-4.547406	1.206472	-2.141580
H	-6.246726	1.486316	-2.449500
C	-4.646314	-1.570178	-1.852896
H	-6.693083	-0.977047	-2.234079
H	-5.566068	-0.566423	-3.510632
H	-4.580120	-2.439320	-2.512306
H	-3.657558	-1.099410	-1.869839
Cl	-7.477970	0.722310	0.066670
C	-4.937920	-2.061270	-0.454469
C	-5.037253	-1.198046	0.647418
C	-5.096736	-3.429320	-0.235522
H	-5.022028	-4.109009	-1.079486
C	-5.338820	-3.945785	1.033277
H	-5.451386	-5.016377	1.169007
C	-5.429510	-3.086341	2.118792
H	-5.611990	-3.471949	3.116000
C	-5.278052	-1.719340	1.916041
H	-5.339915	-1.044995	2.765007

20. ^{1}H NMR, ^{13}C NMR AND ^{19}F NMR SPECTRA OF THE COMPOUNDS PREPARED

^{1}H NMR of **^{n}BuO-NpMI** in $CDCl_3$

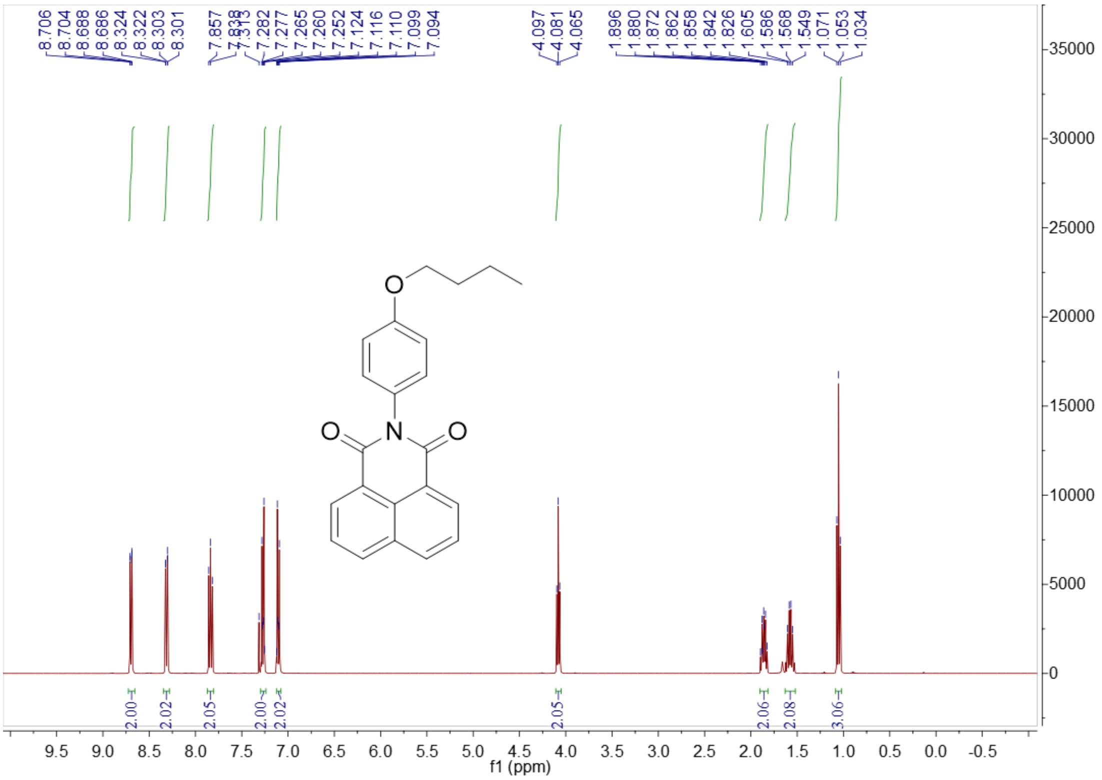

^{13}C NMR of **^{n}BuO-NpMI** in $CDCl_3$

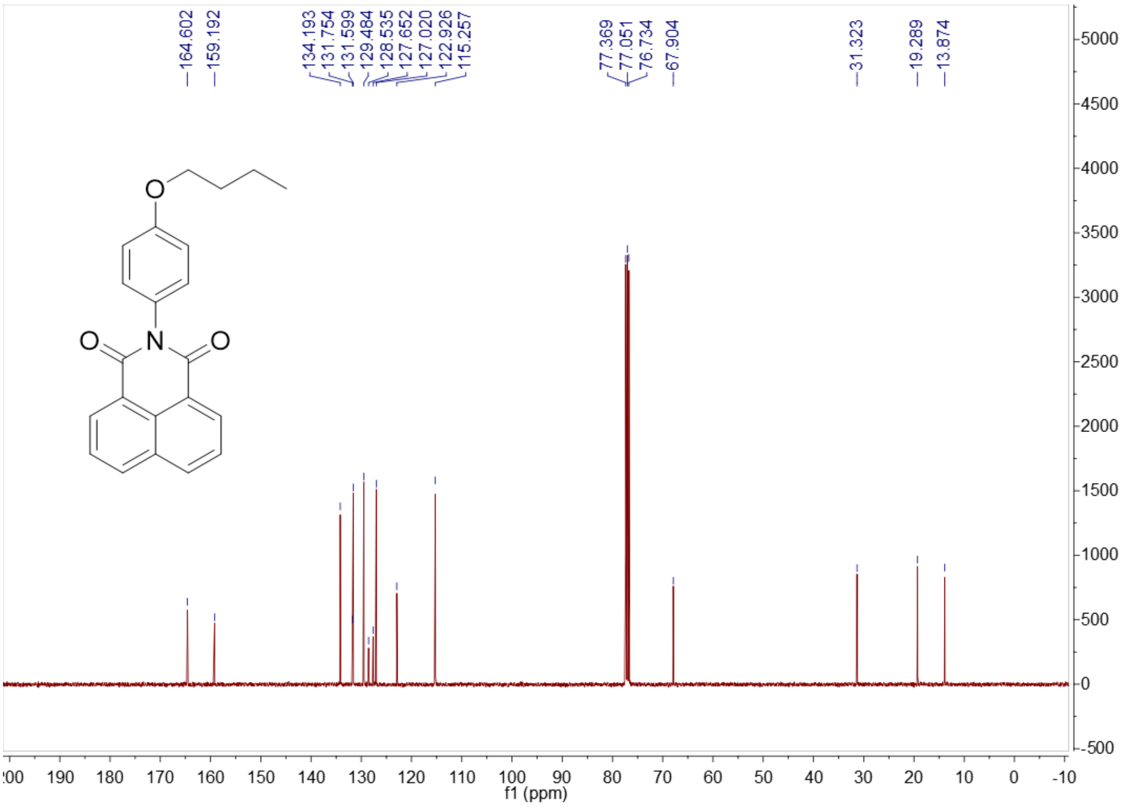

^{1}H NMR of **5a** in $CDCl_3$

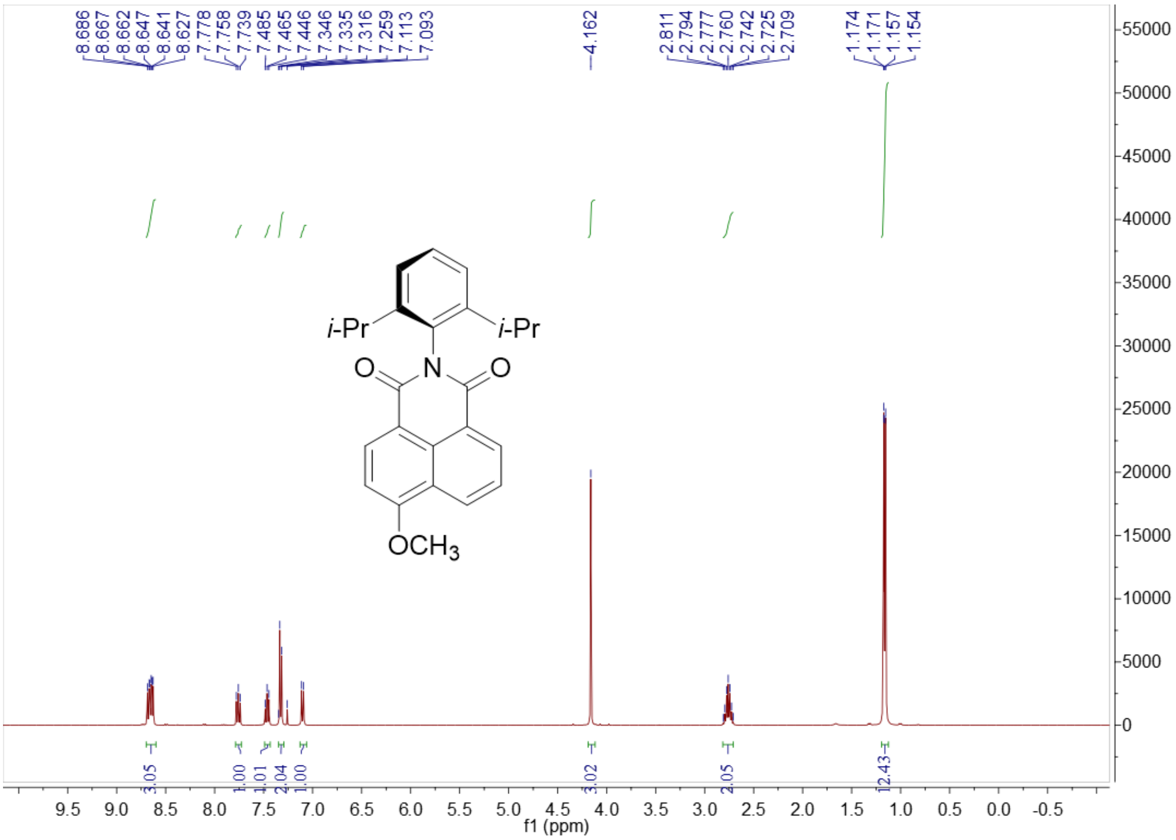

^{13}C NMR of **5a** in $CDCl_3$

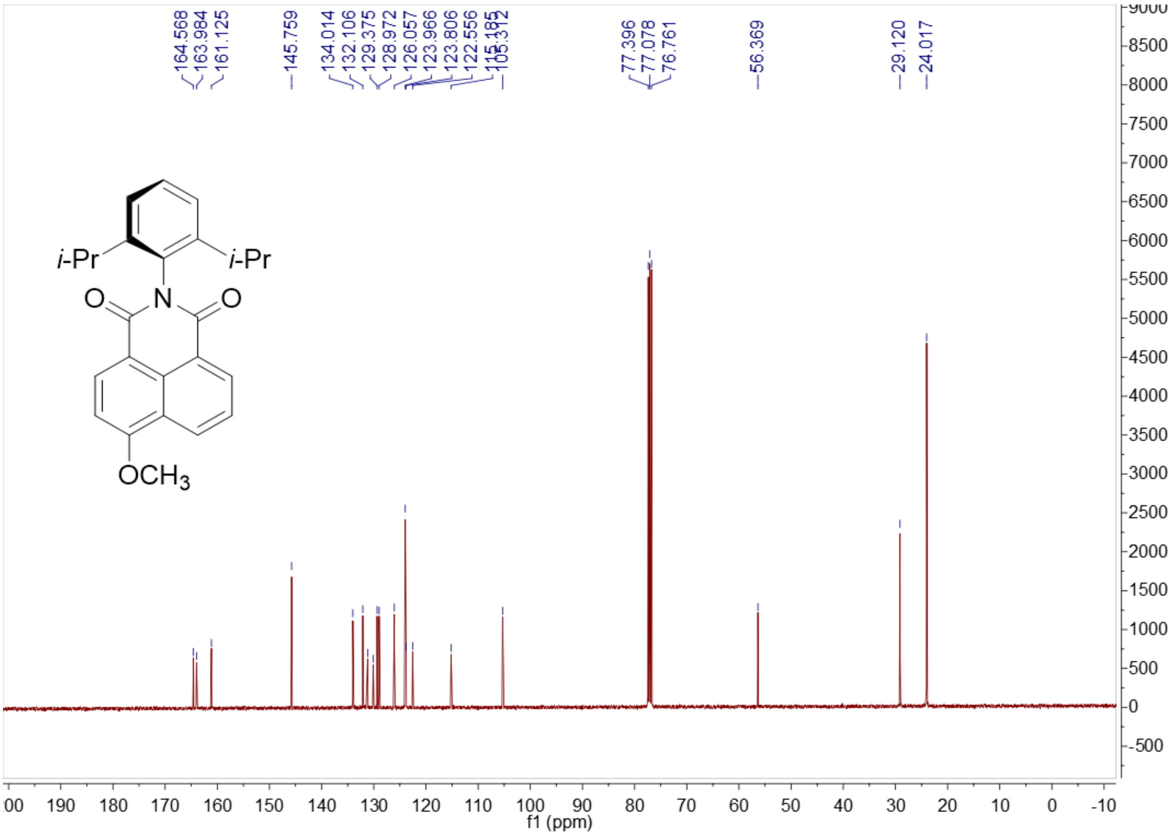

^1H NMR of **5b** in $CDCl_3$

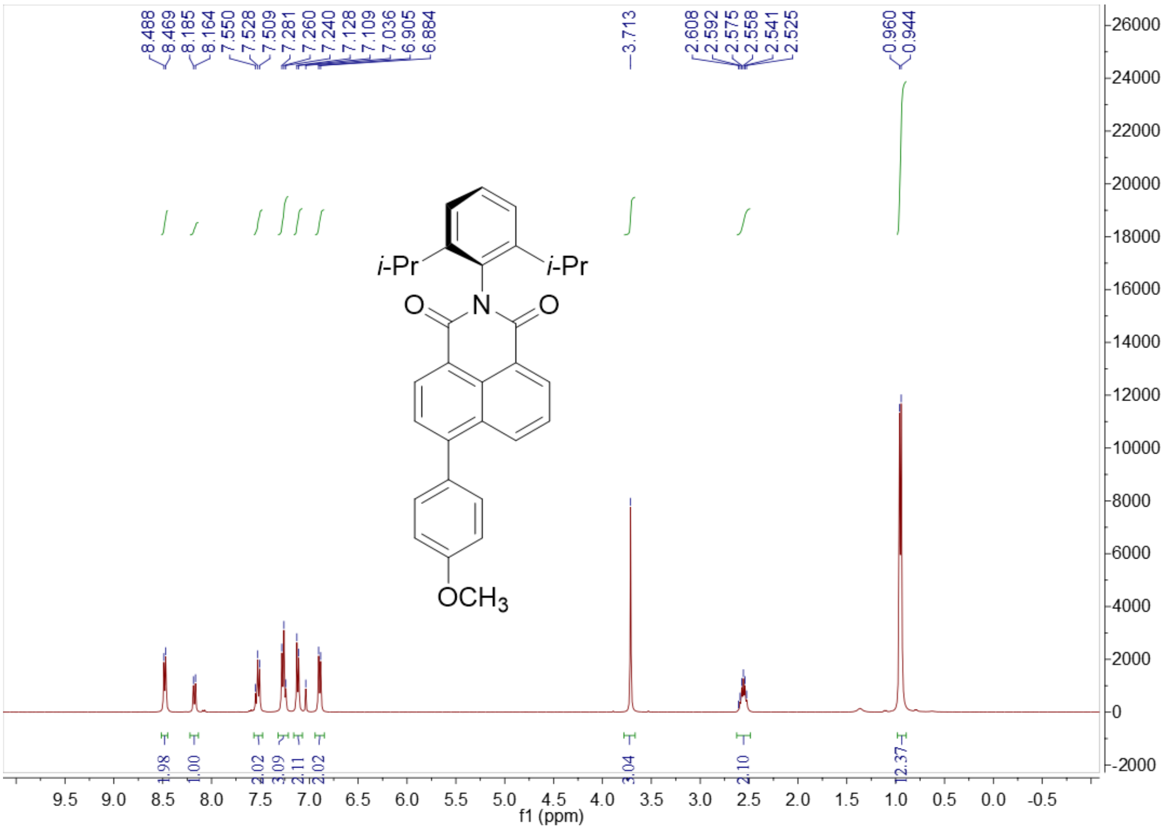

^{13}C NMR of **5b** in $CDCl_3$

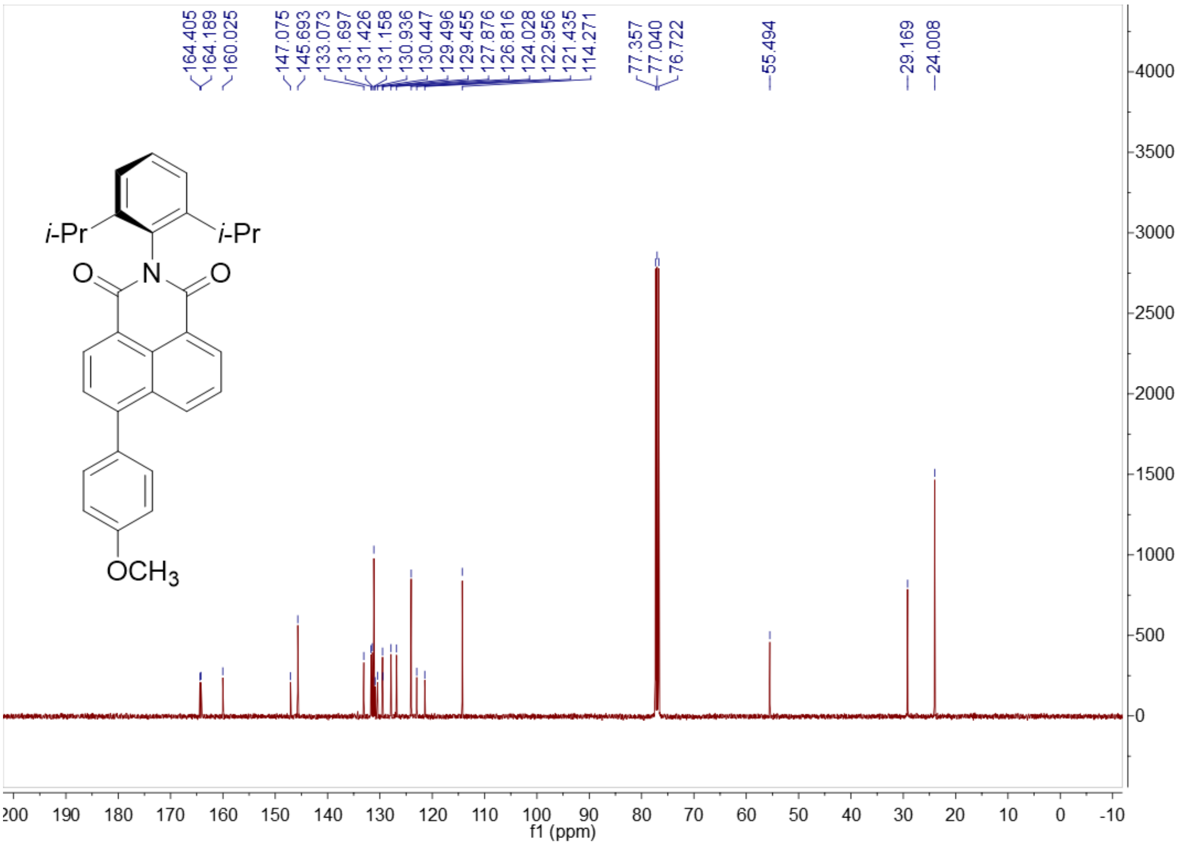

^{1}H NMR of **5c** in $CDCl_3$

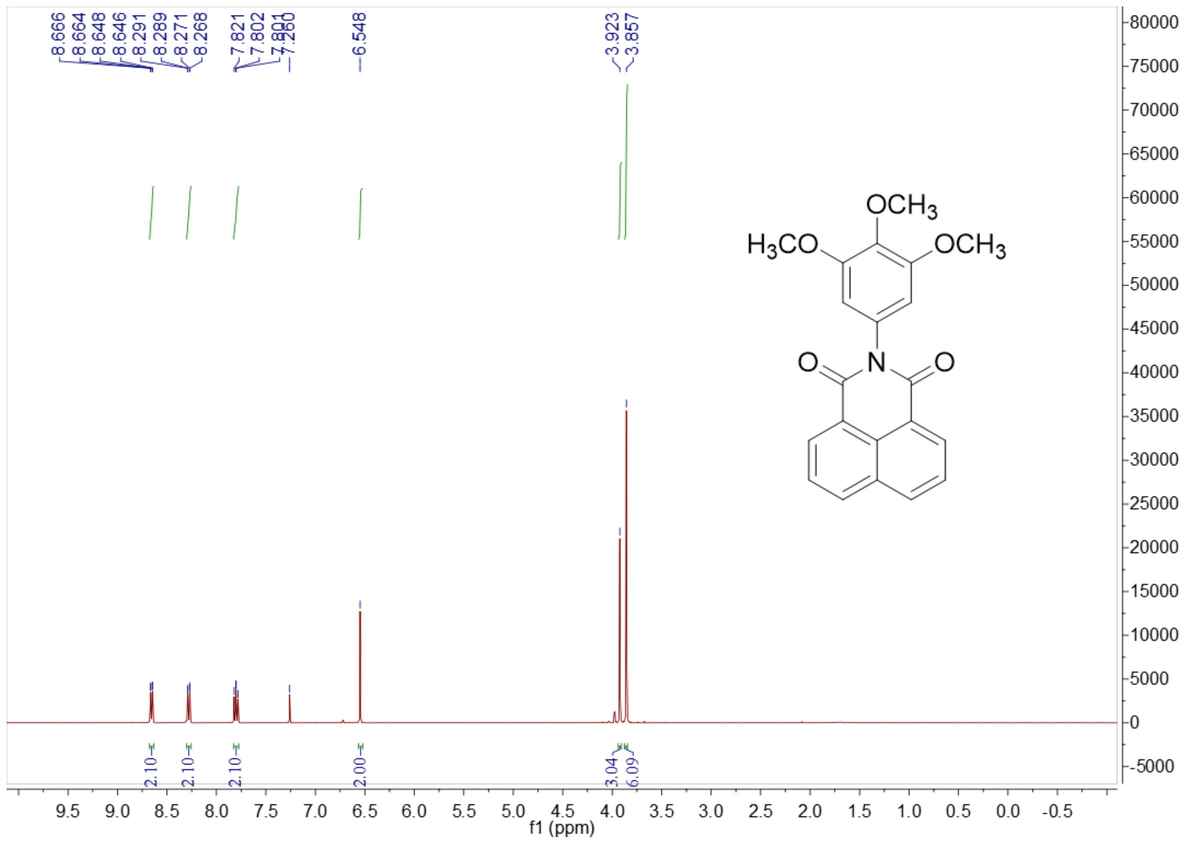

^{13}C NMR of **5c** in $CDCl_3$

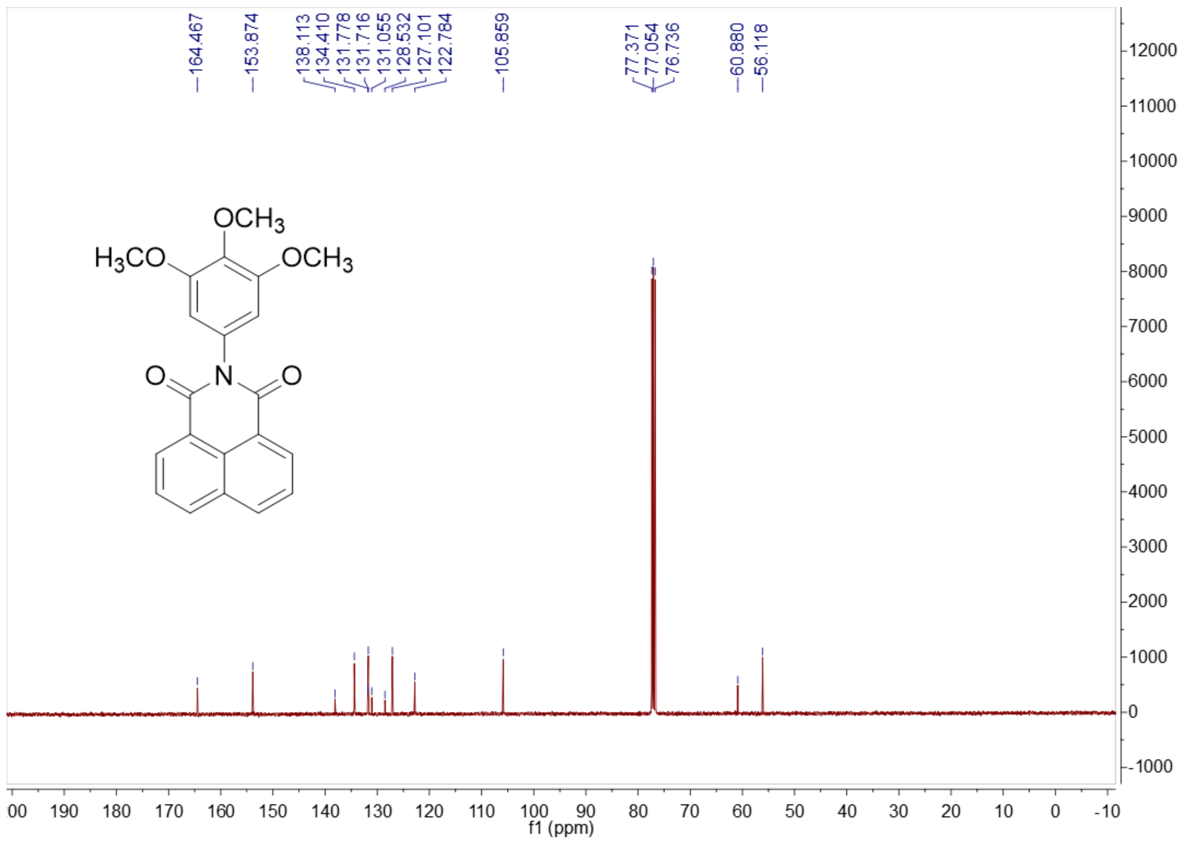

^{1}H NMR of **5d** in $CDCl_3$

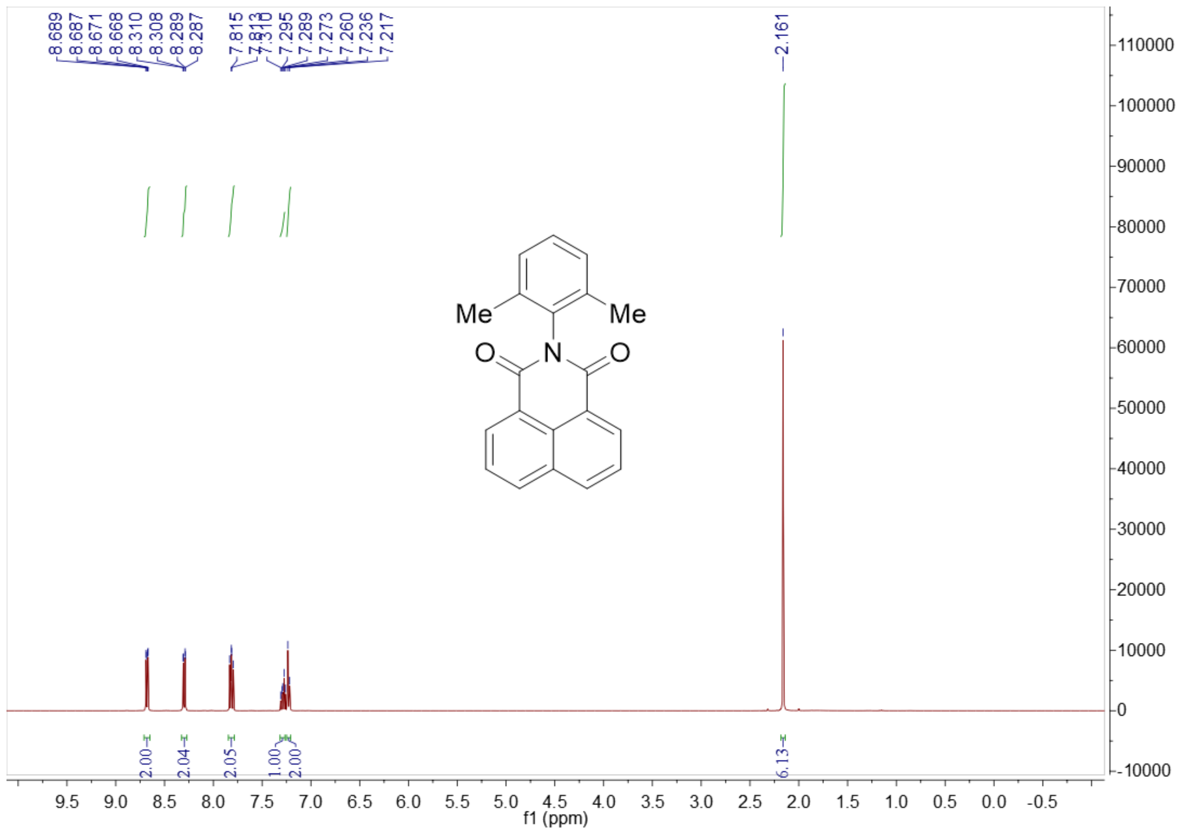

^{13}C NMR of **5d** in $CDCl_3$

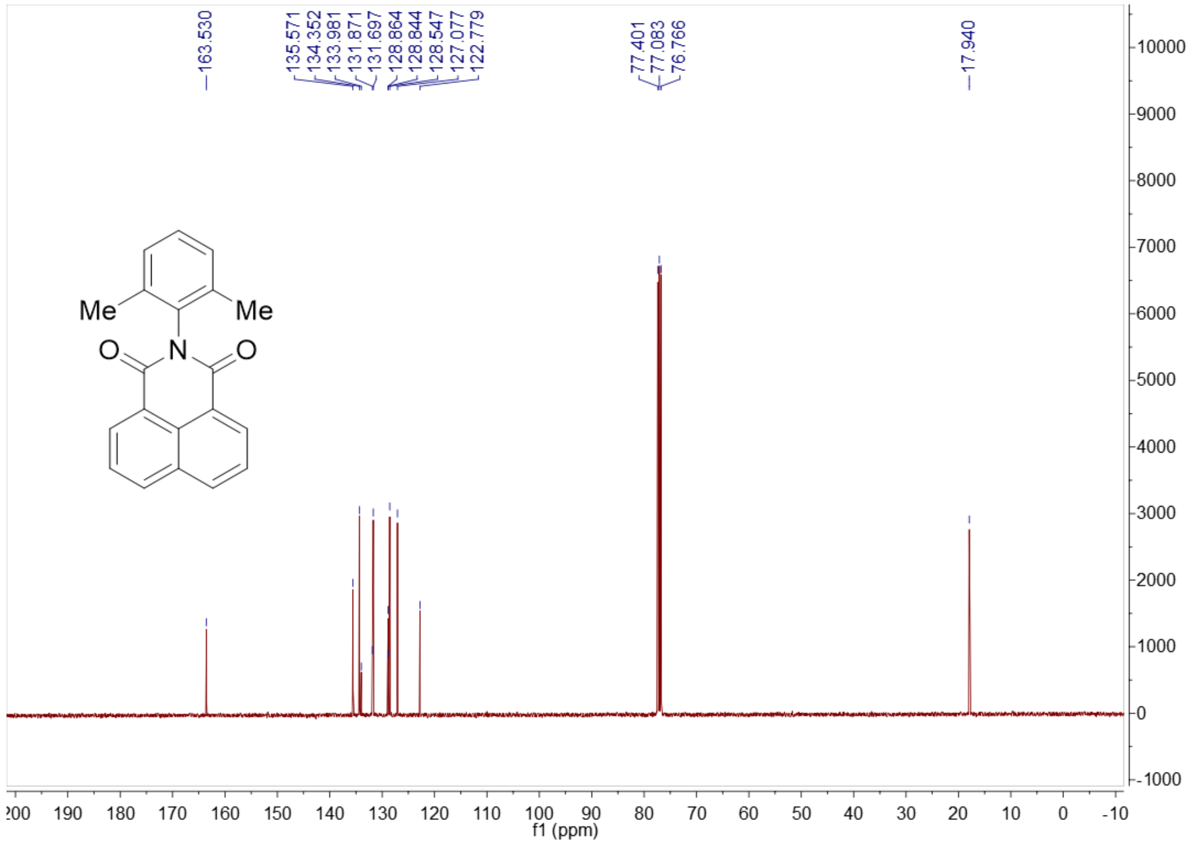

^{1}H NMR of **5e** in $CDCl_3$

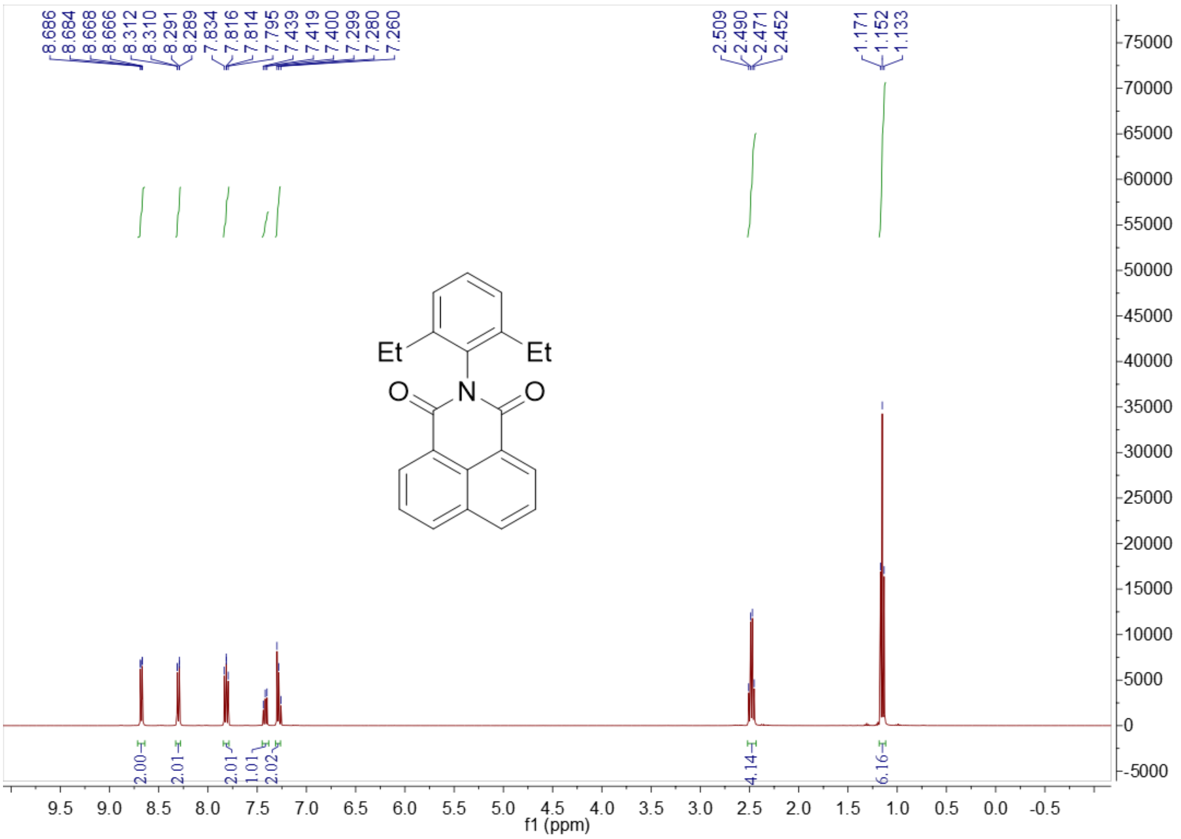

^{13}C NMR of **5e** in $CDCl_3$

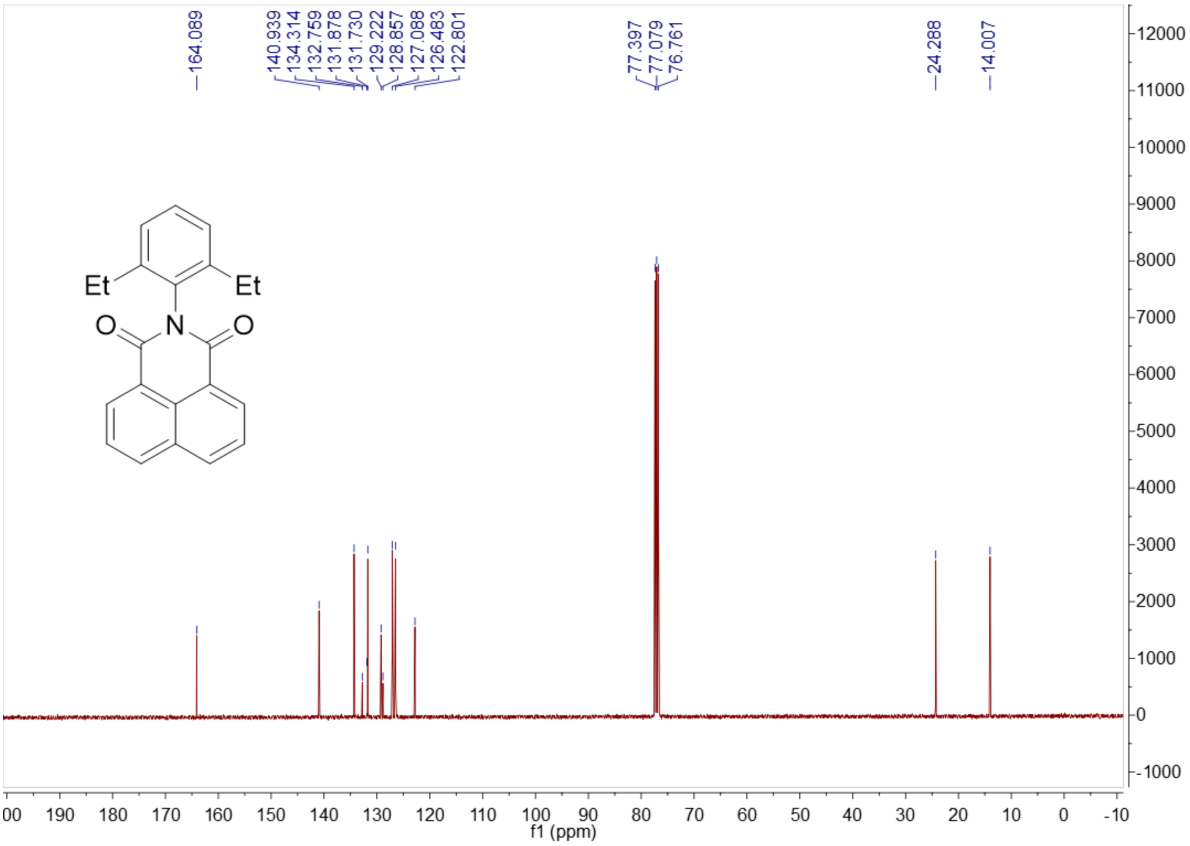

^{1}H NMR of compound **1a** in $CDCl_3$

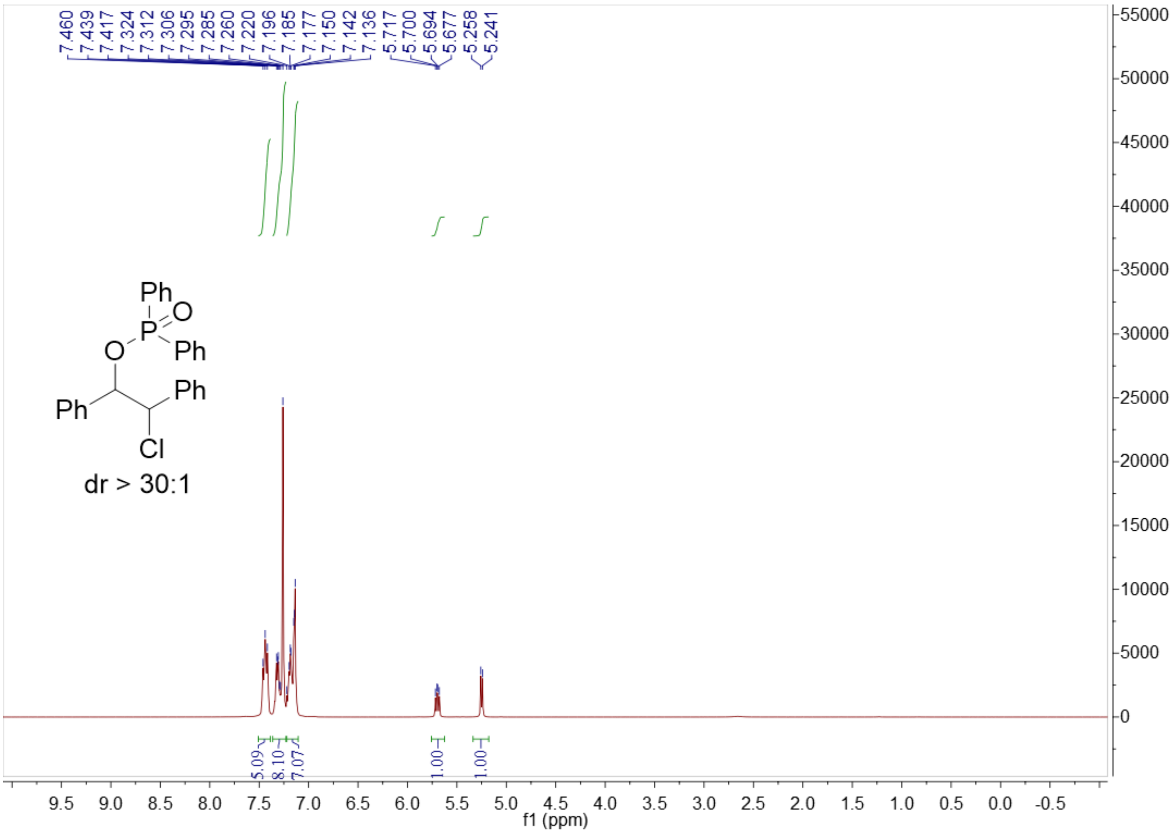

^{13}C NMR of compound **1a** in $CDCl_3$

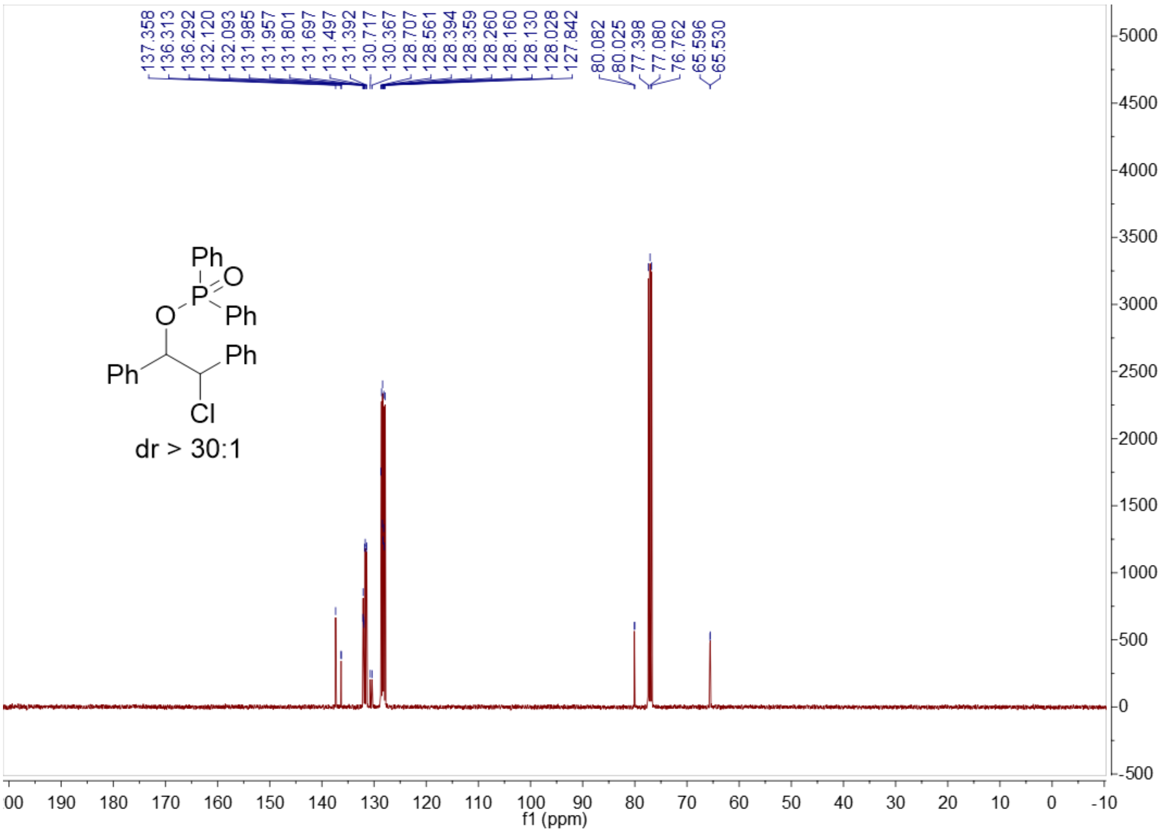

^{1}H NMR of compound **1b** in $CDCl_3$

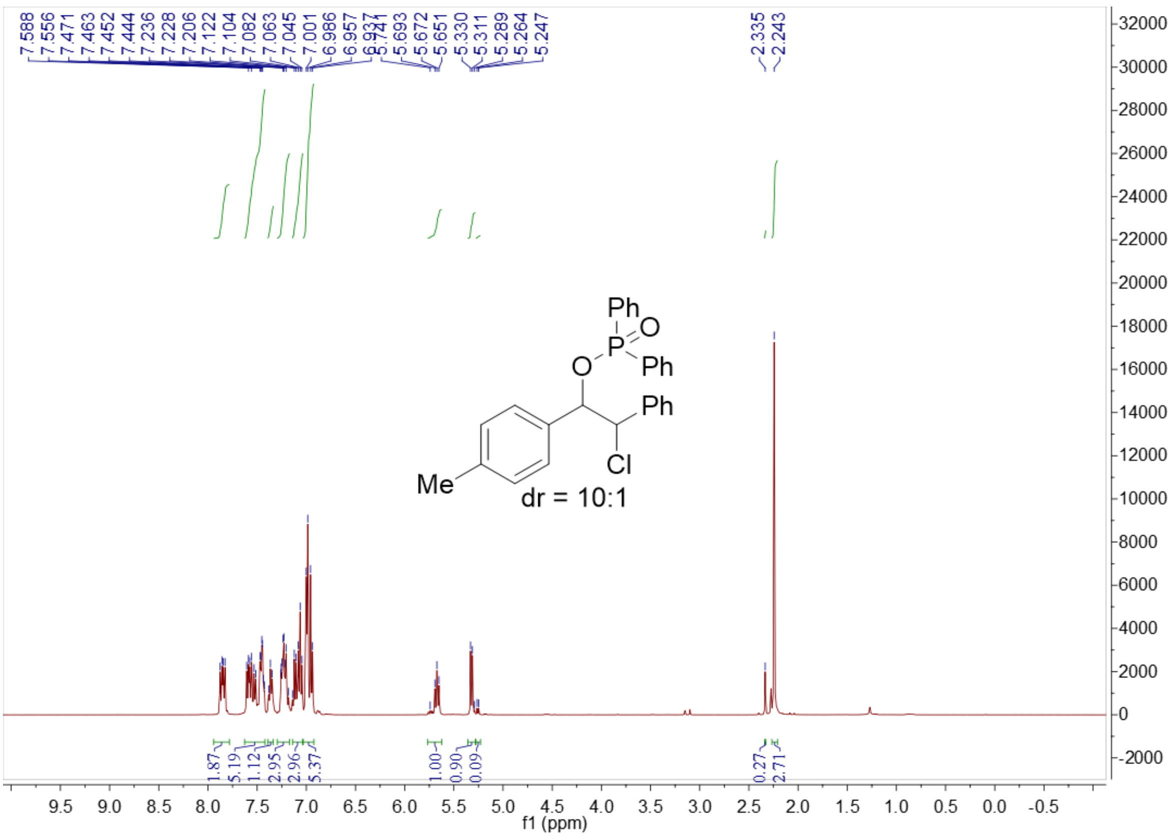

^{13}C NMR of compound **1b** in $CDCl_3$

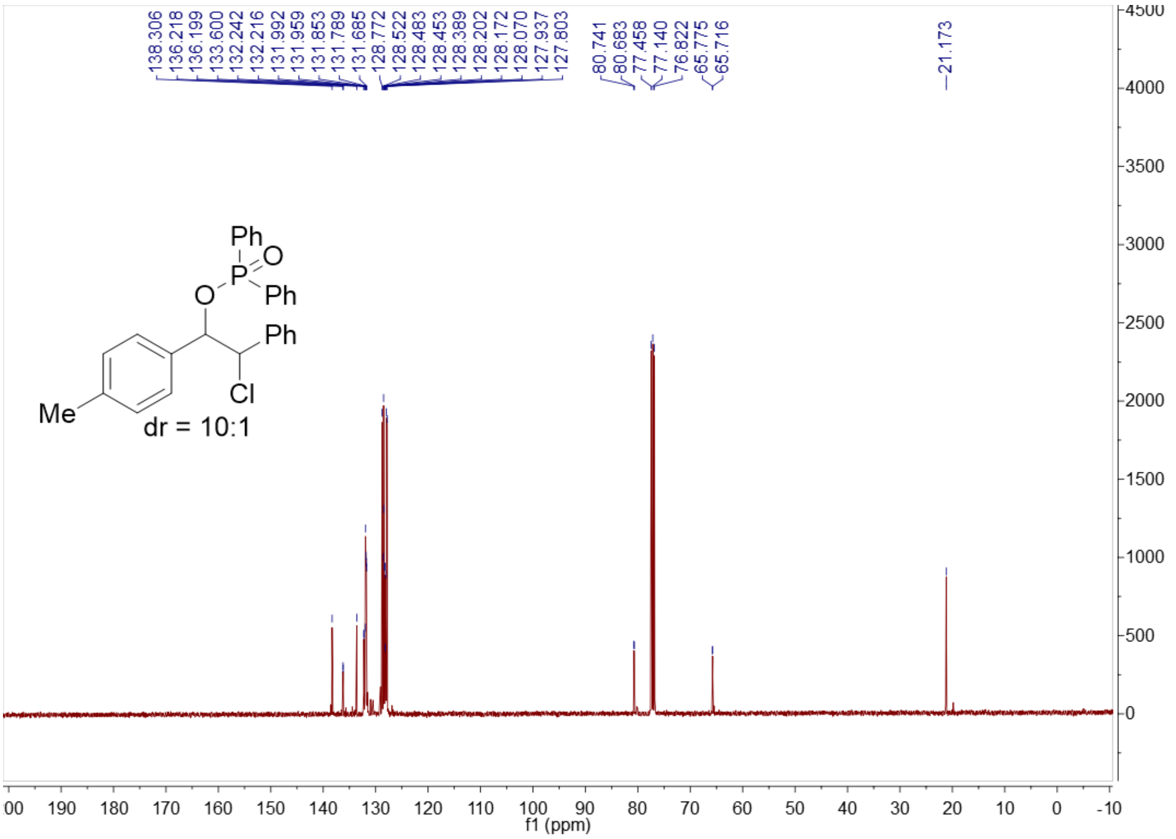

^{1}H NMR of compound **1c** in CDCl$_3$

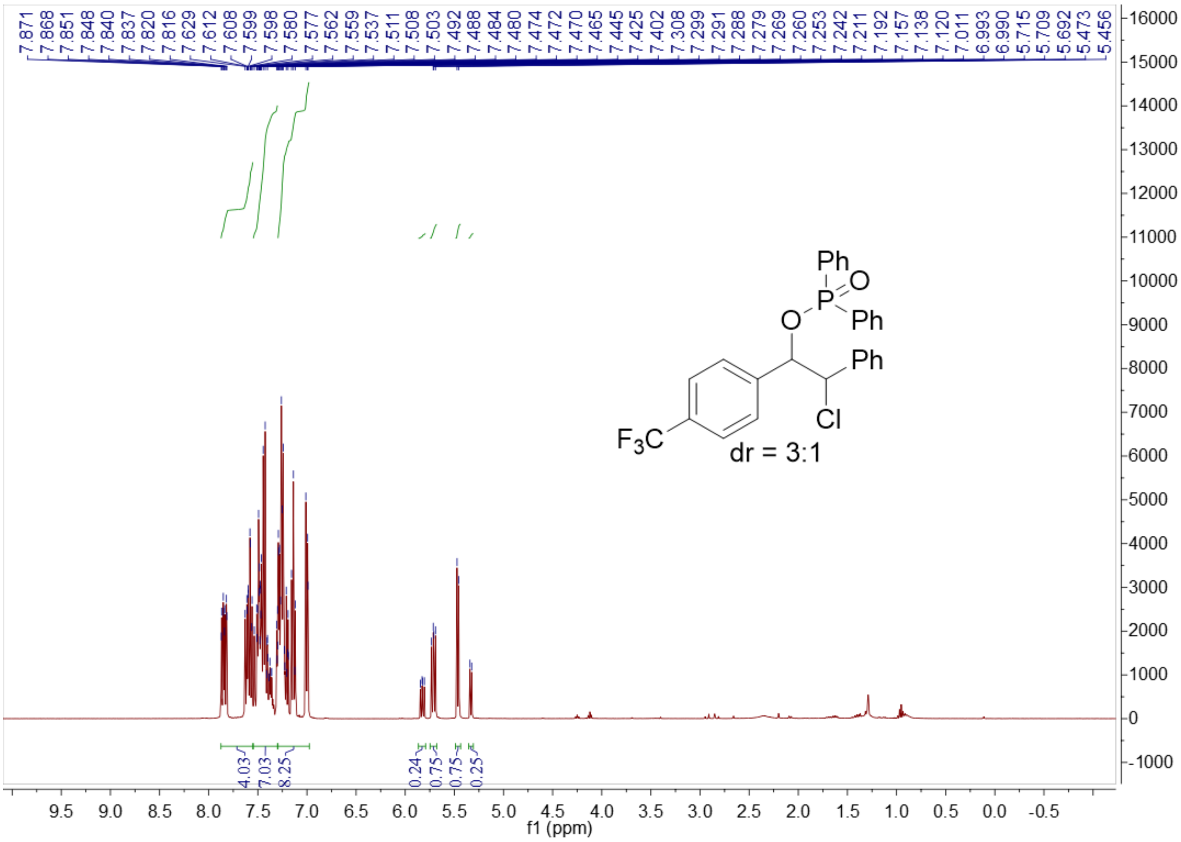

^{13}C NMR of compound **1c** in CDCl$_3$

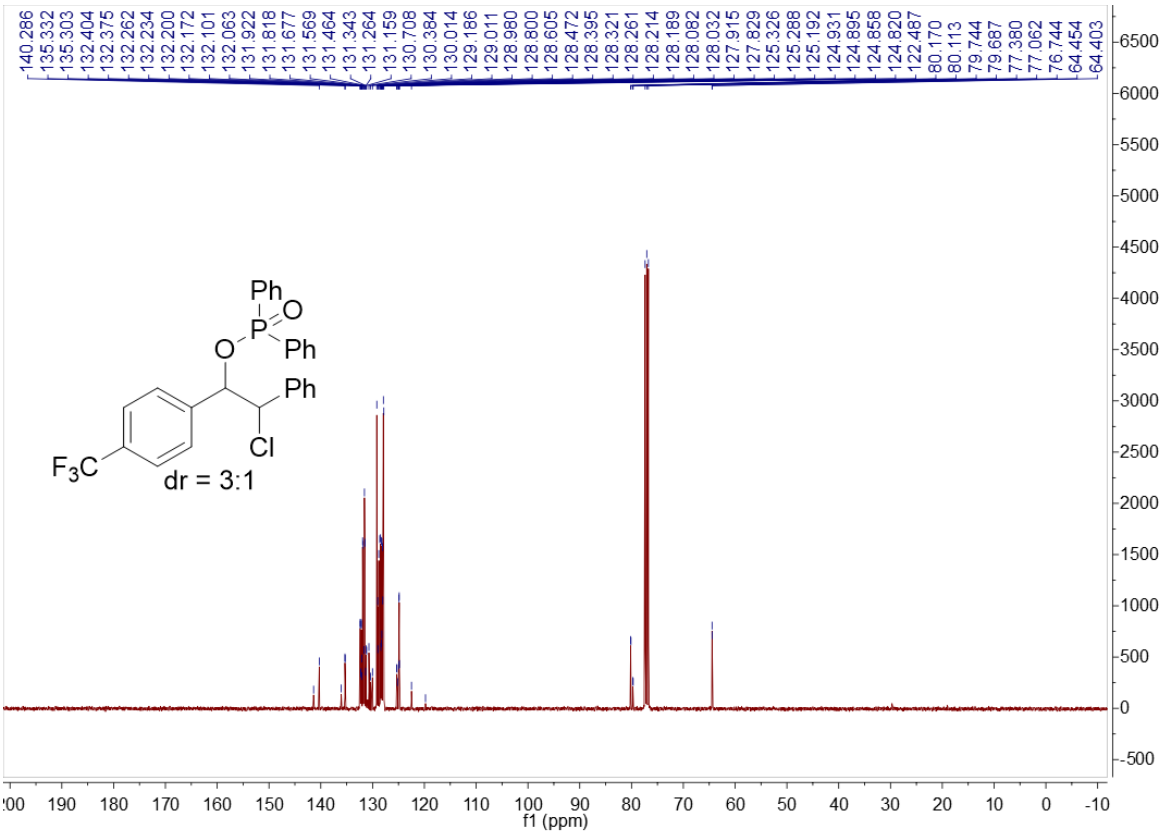

^{1}H NMR of compound **1d** in $CDCl_3$

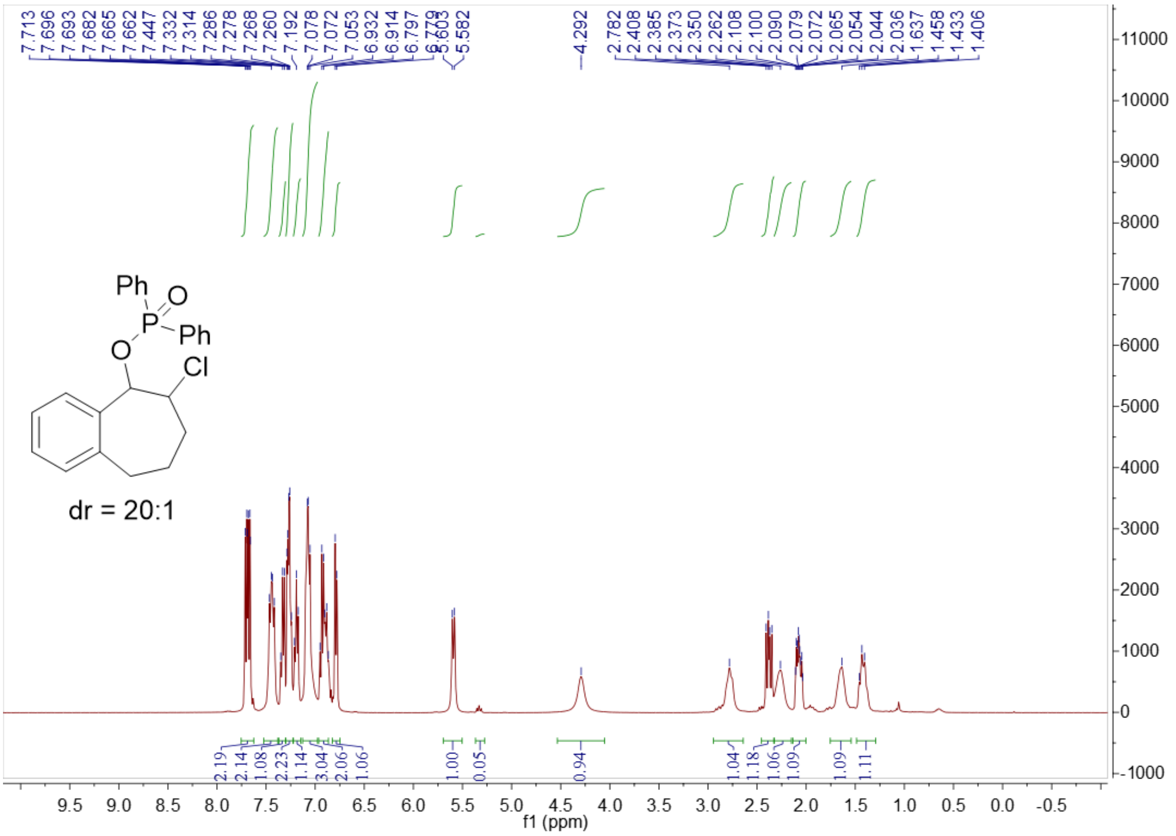

^{13}C NMR of compound **1d** in $CDCl_3$

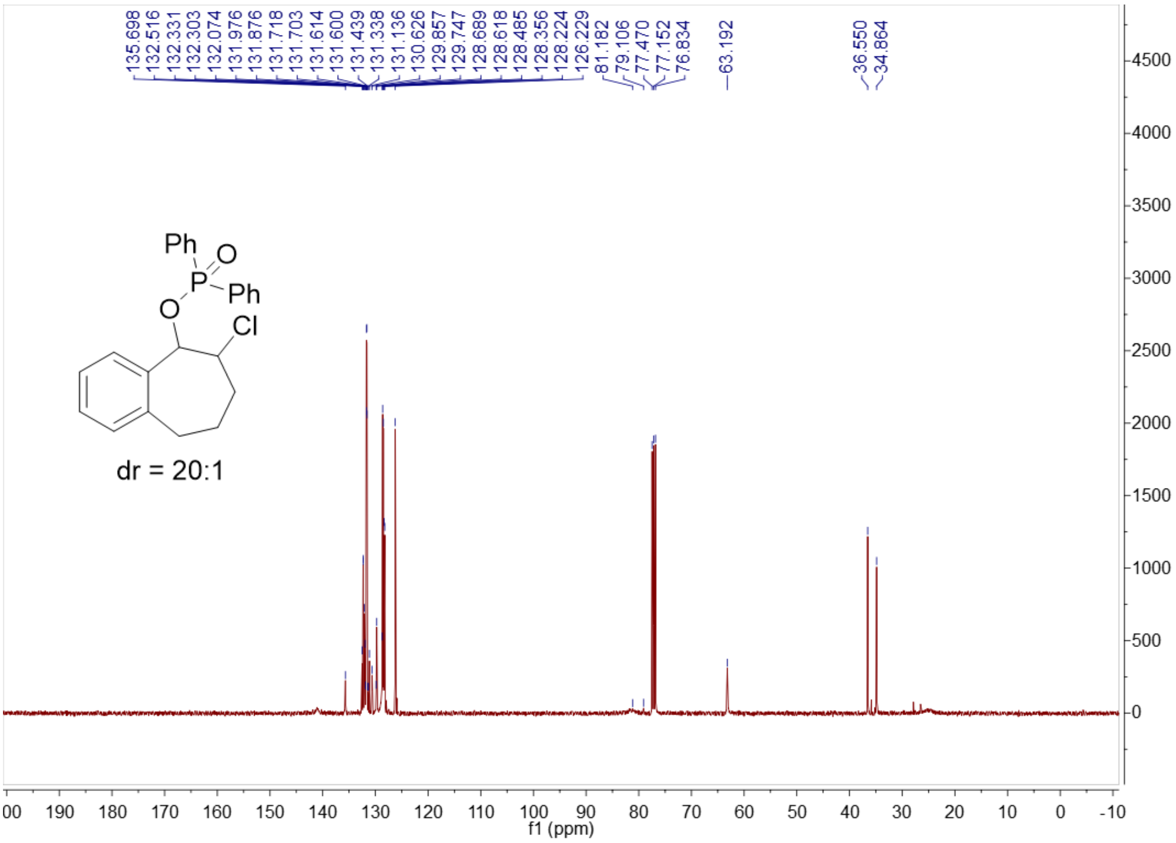

^{1}H NMR of compound **1e** in $CDCl_3$

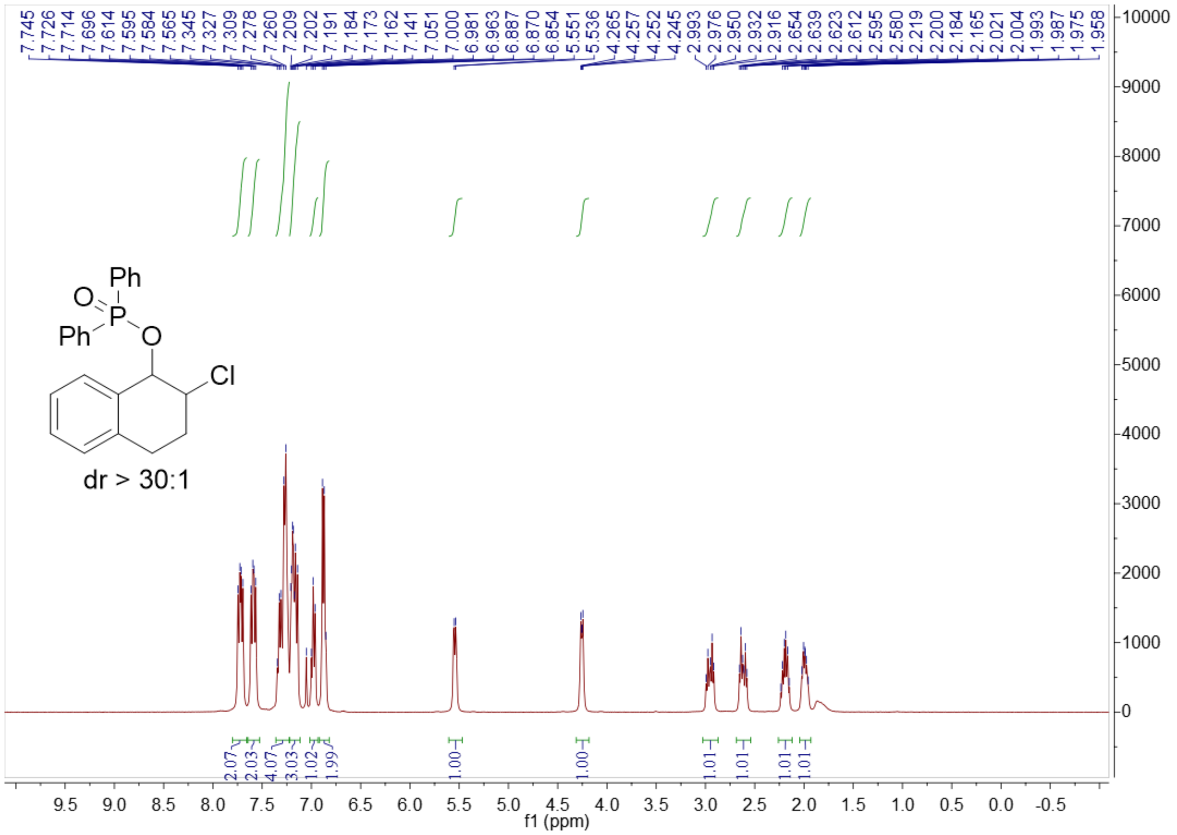

^{13}C NMR of compound **1e** in $CDCl_3$

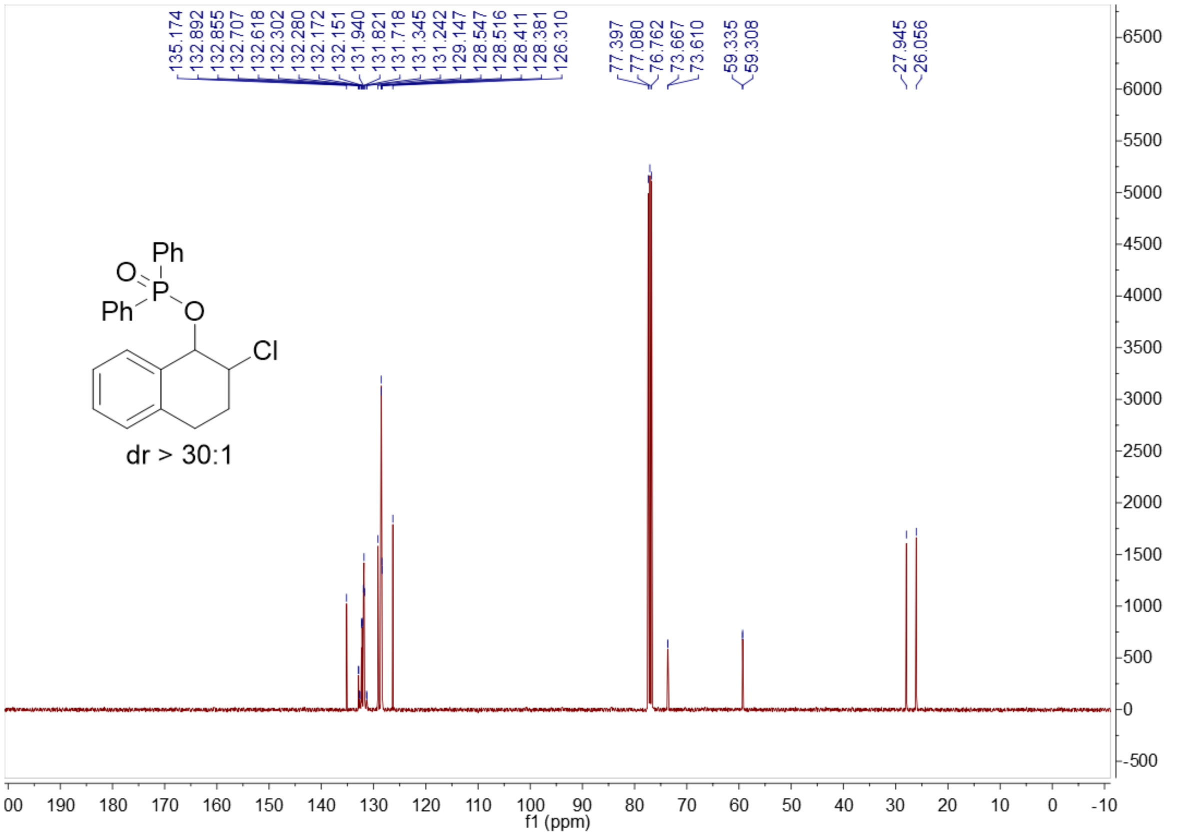

^{1}H NMR of compound **1f** in $CDCl_3$

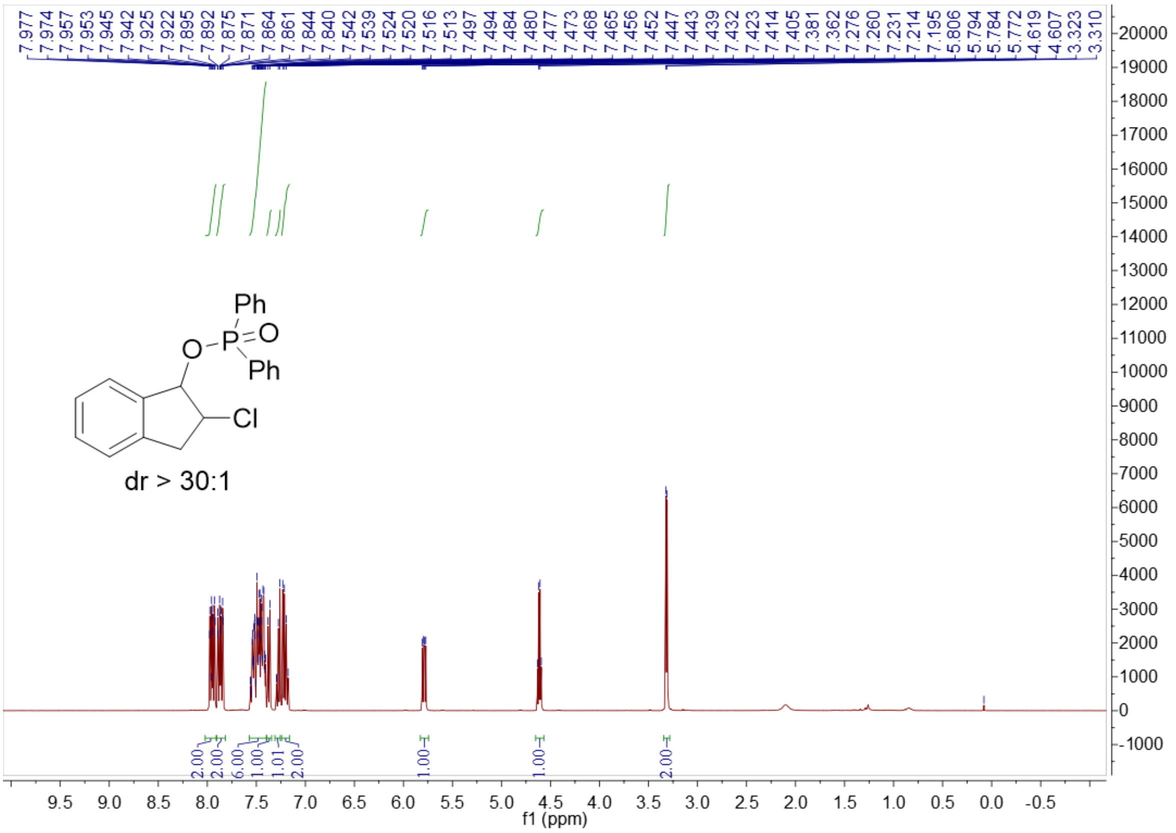

^{13}C NMR of compound **1f** in $CDCl_3$

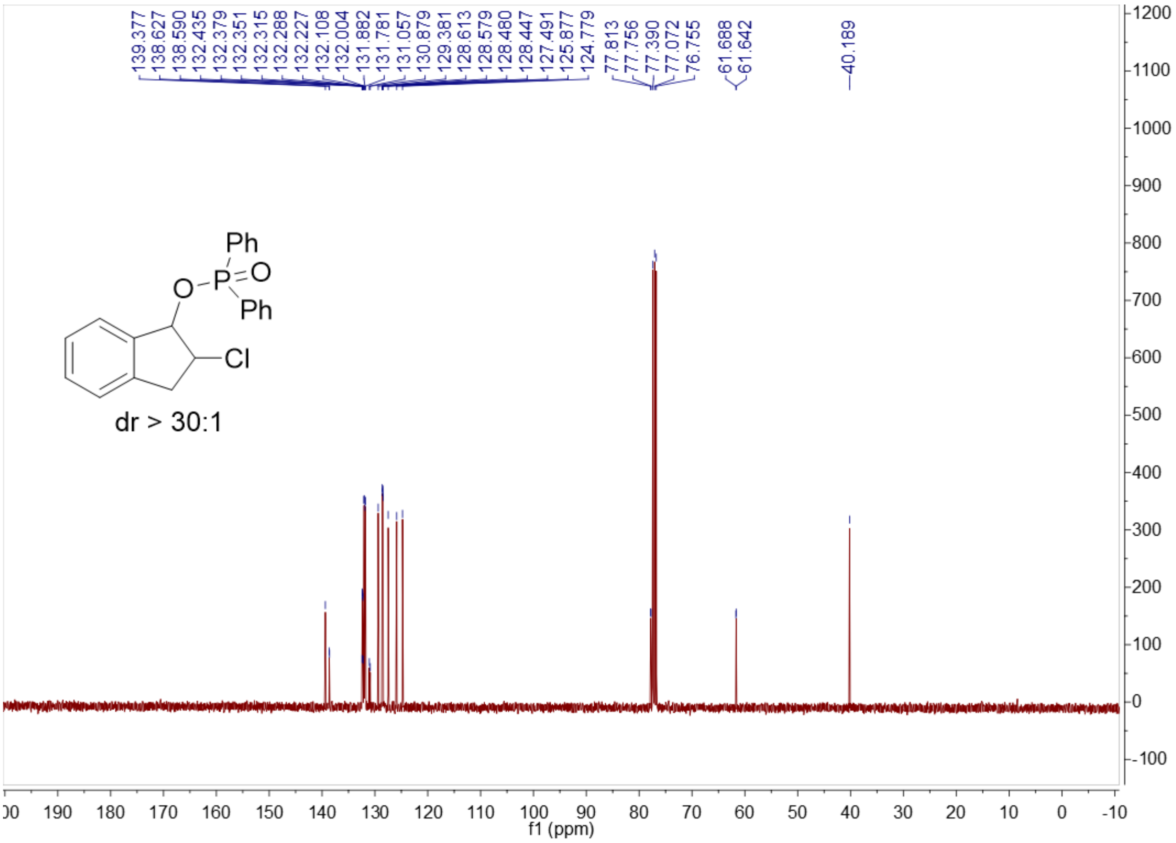

^{1}H NMR of compound **1g** in $CDCl_3$

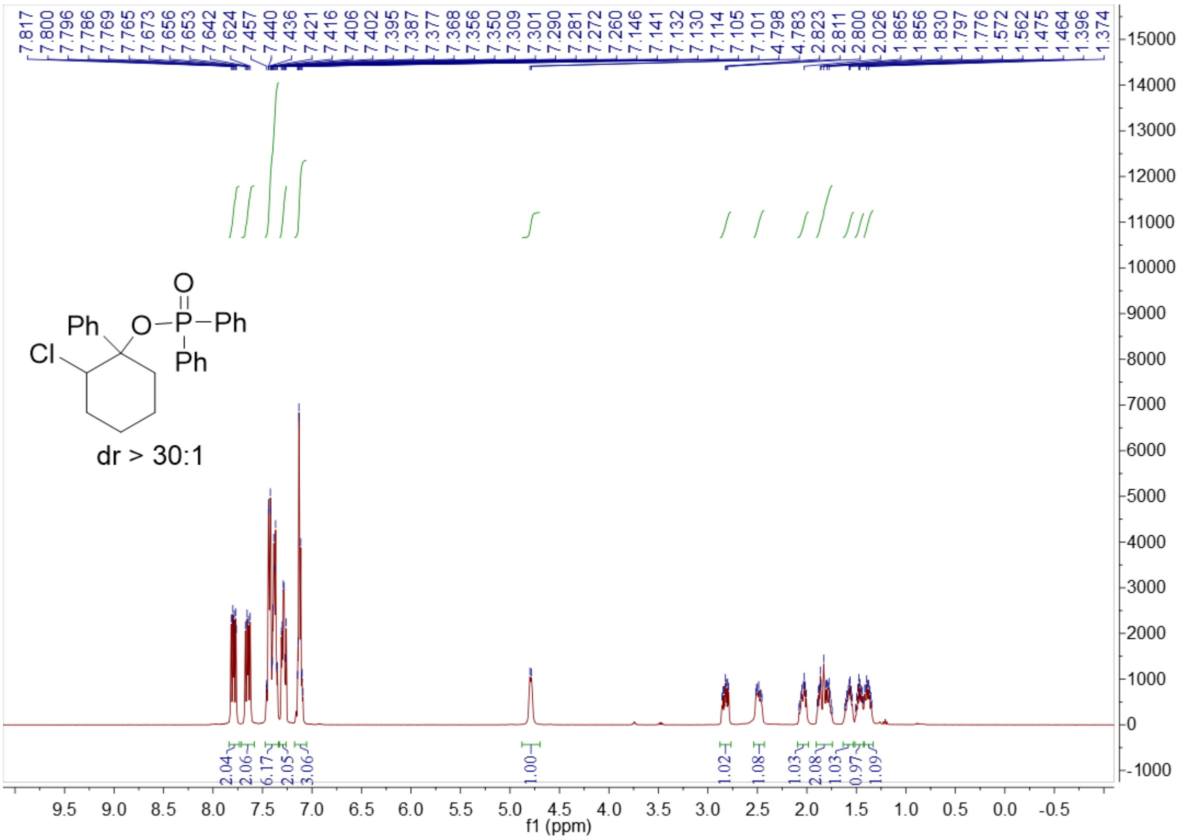

^{13}C NMR of compound **1g** in $CDCl_3$

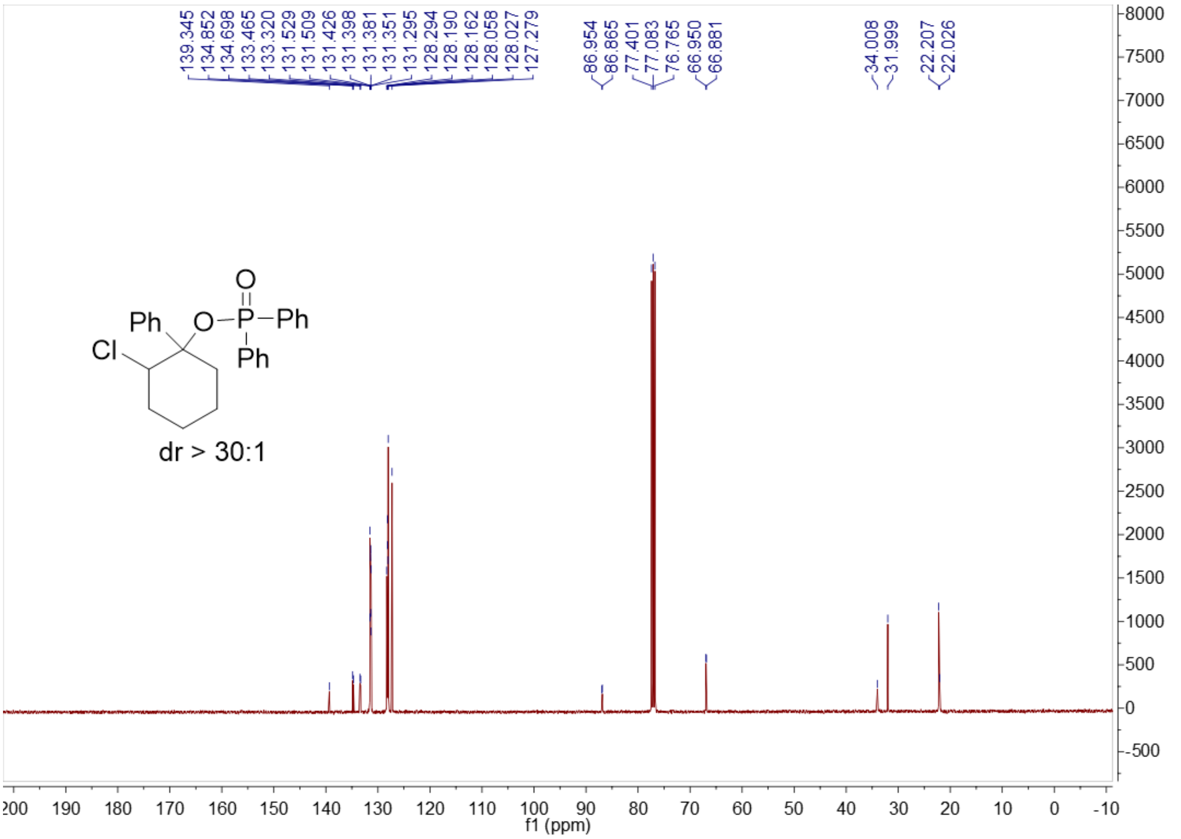

^{1}H NMR of compound **1h** in $CDCl_3$

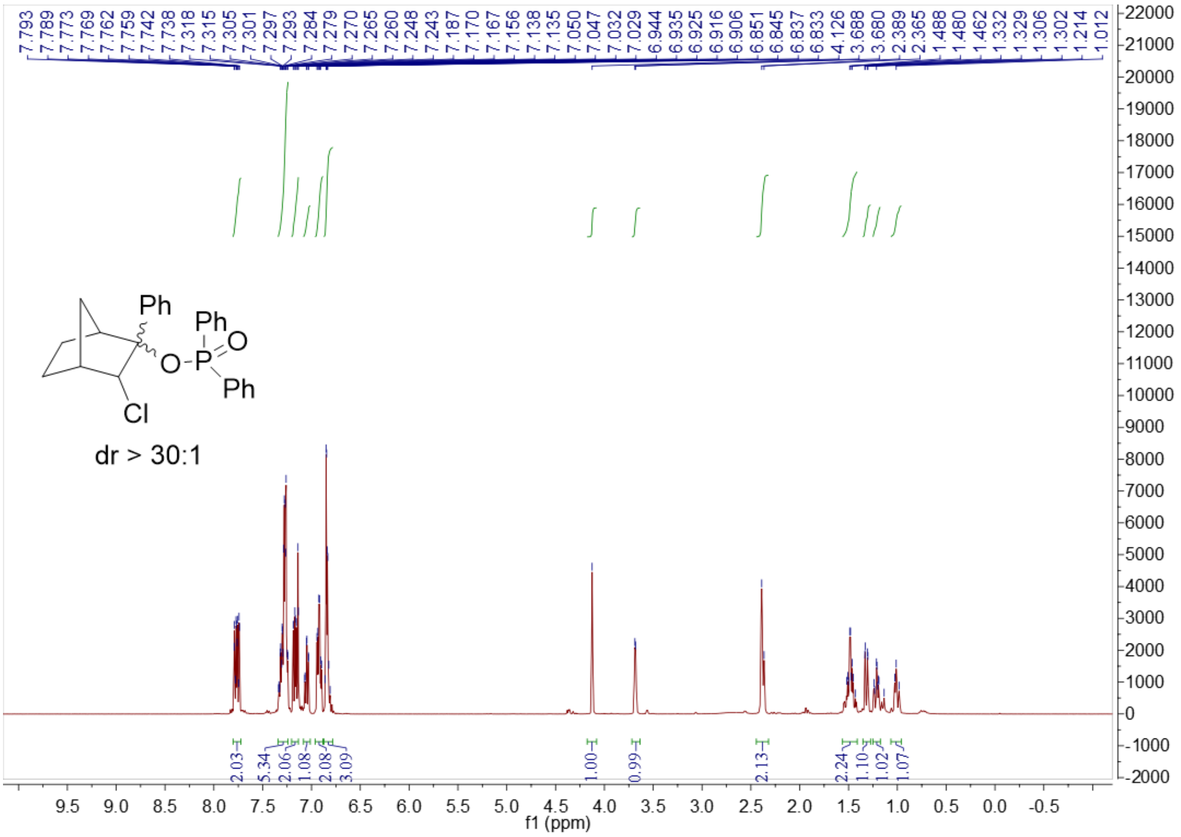

^{13}C NMR of compound **1h** in $CDCl_3$

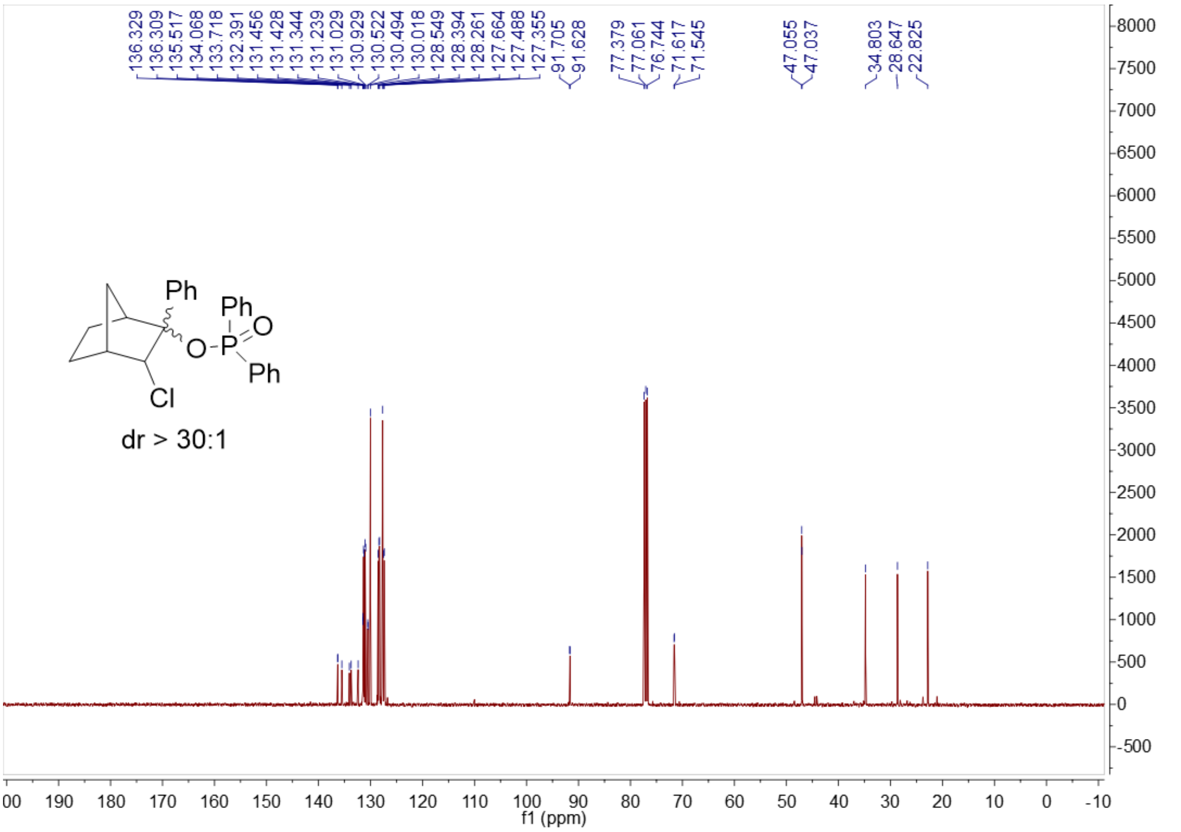

^{1}H NMR of compound **1j** in $CDCl_3$

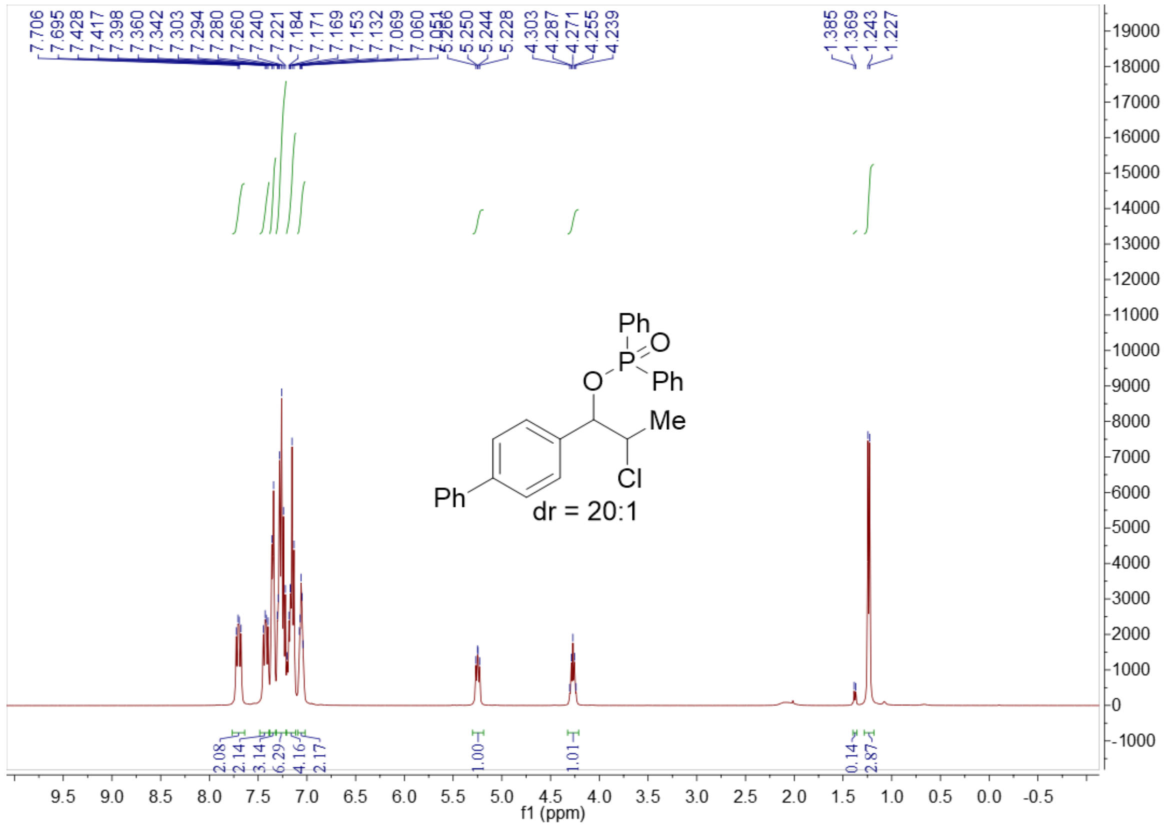

^{13}C NMR of compound **1j** in $CDCl_3$

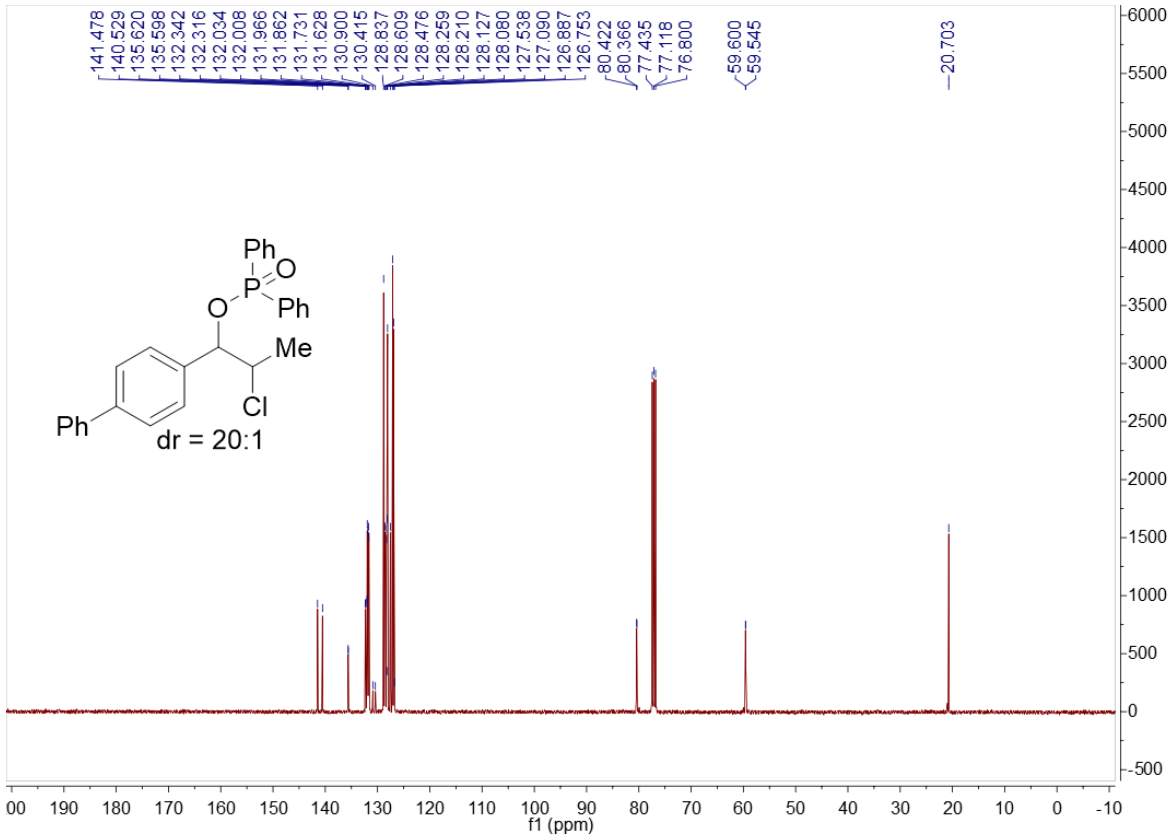

^{1}H NMR of compound **1k** in $CDCl_3$

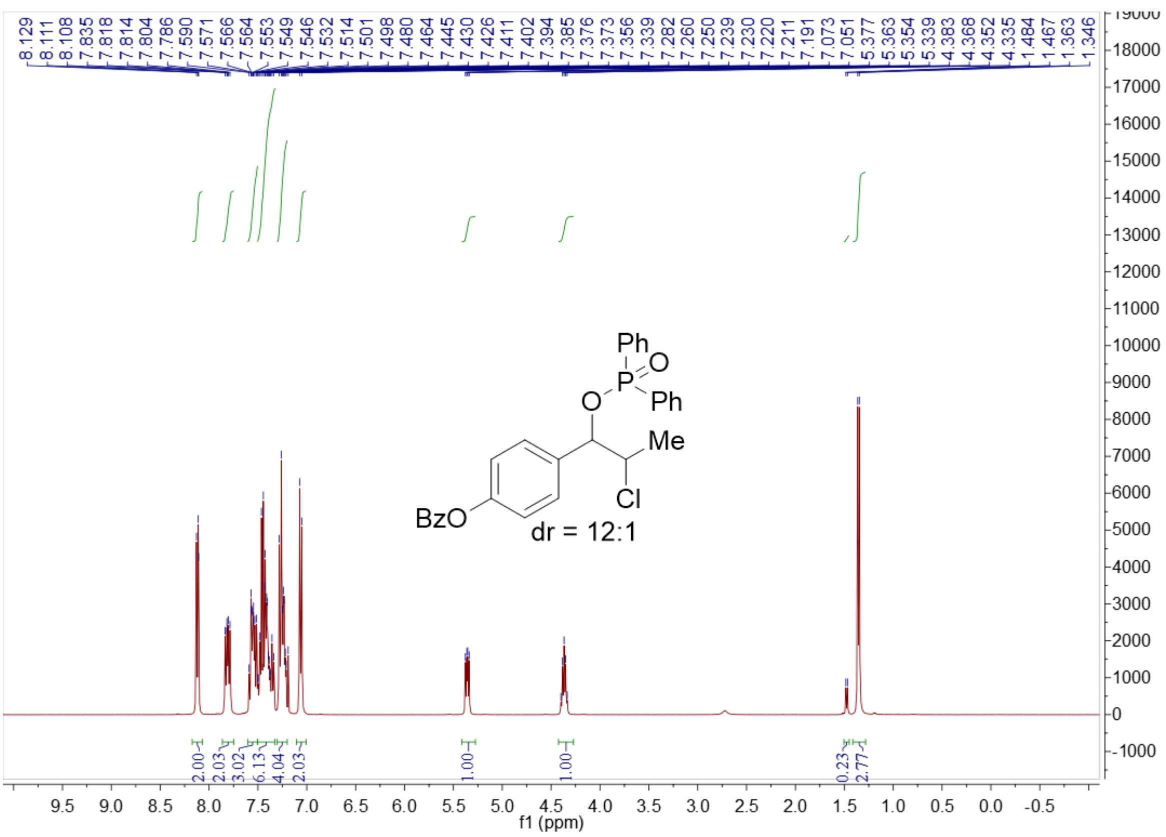

^{13}C NMR of compound **1k** in $CDCl_3$

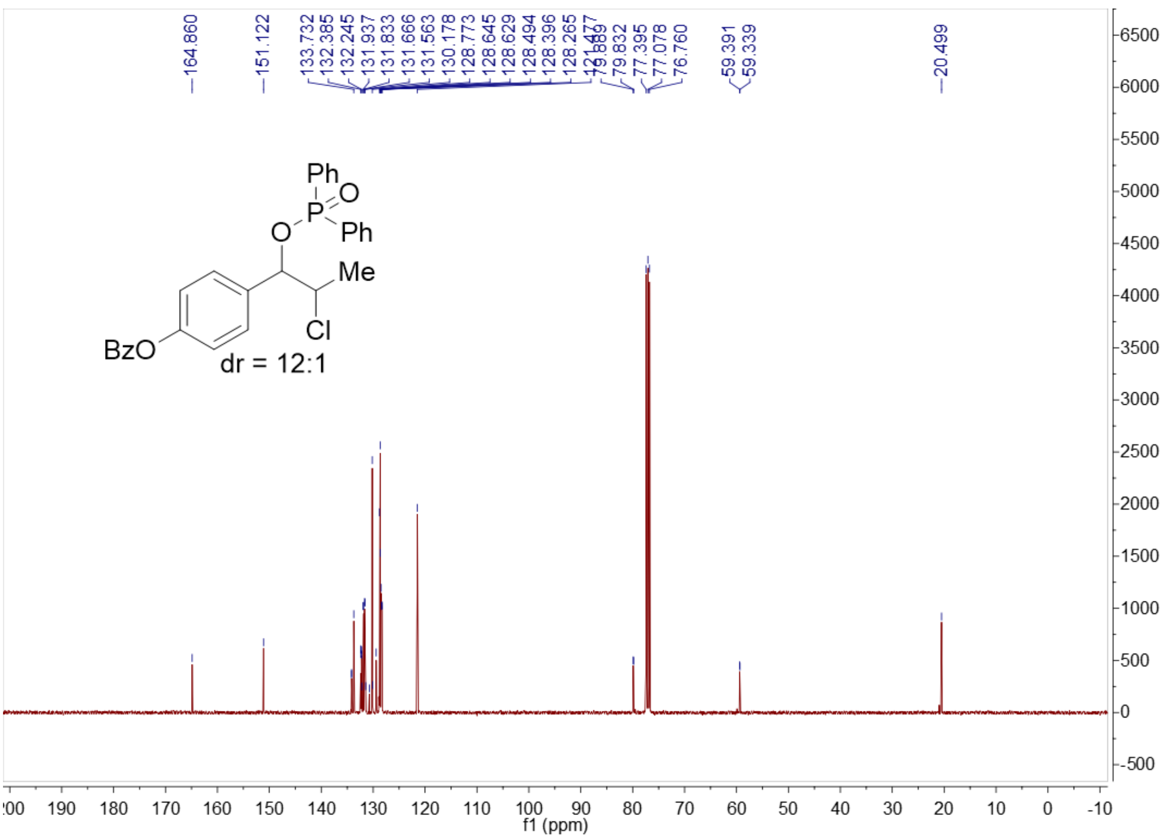

^{1}H NMR of compound **1l** in $CDCl_3$

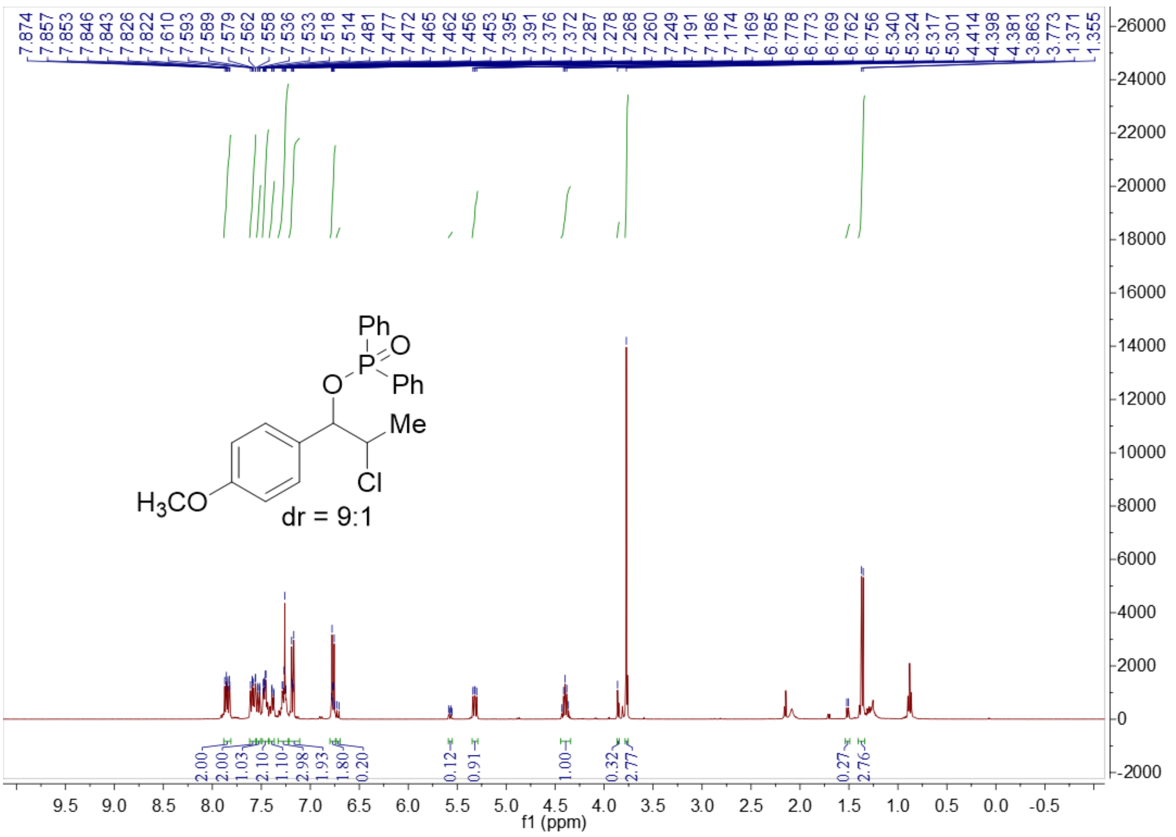

^{13}C NMR of compound **1l** in $CDCl_3$

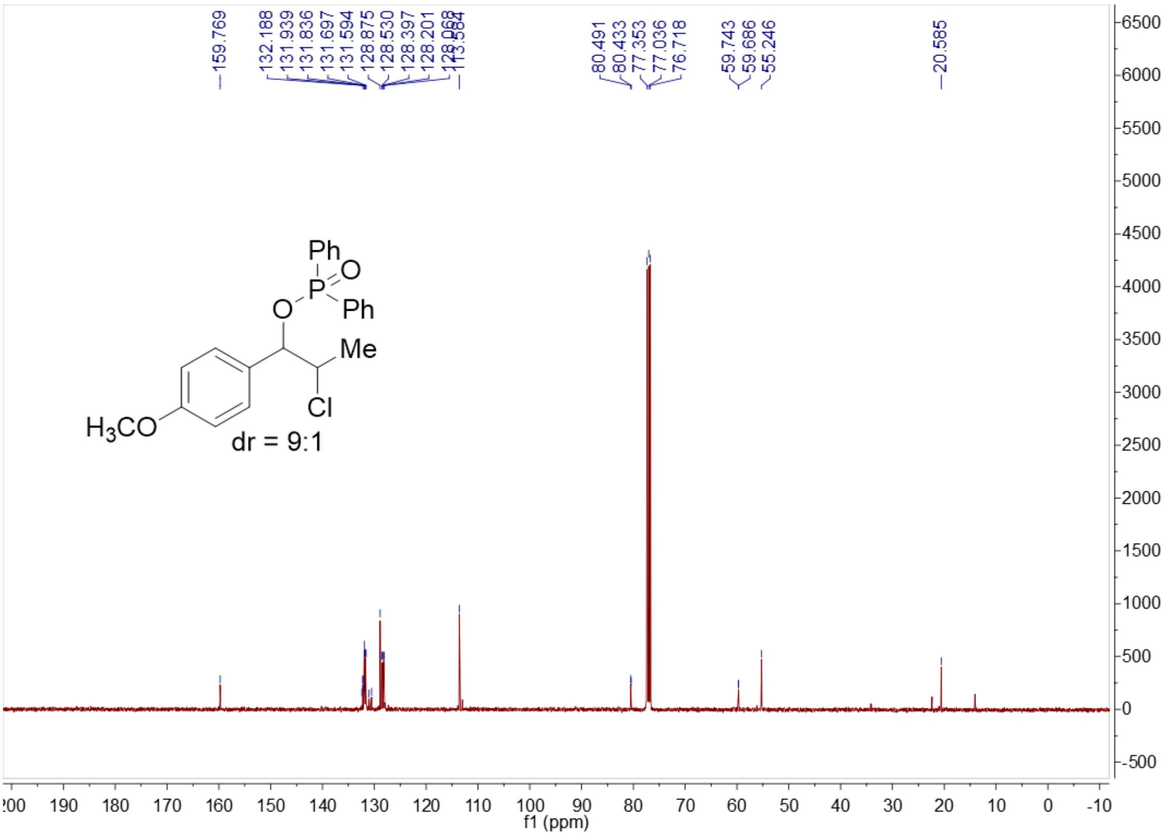

^{1}H NMR of compound **1m** in $CDCl_3$

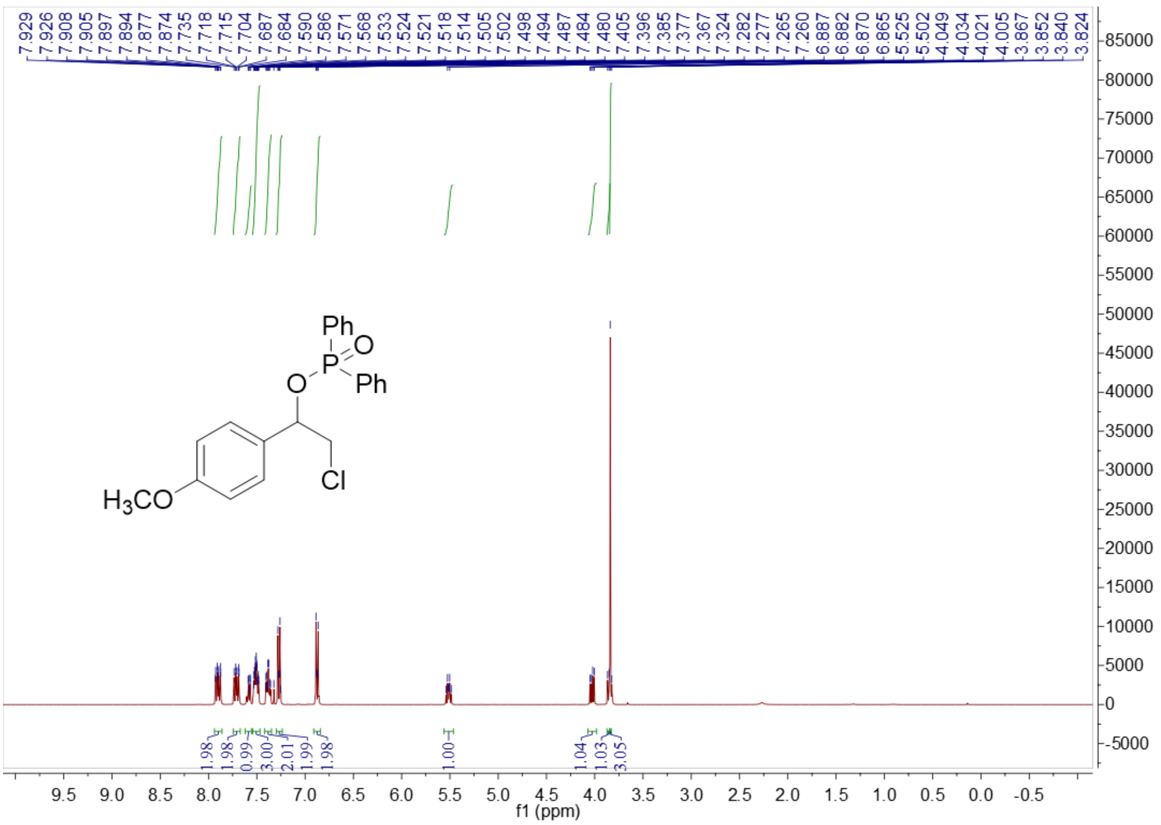

^{13}C NMR of compound **1m** in $CDCl_3$

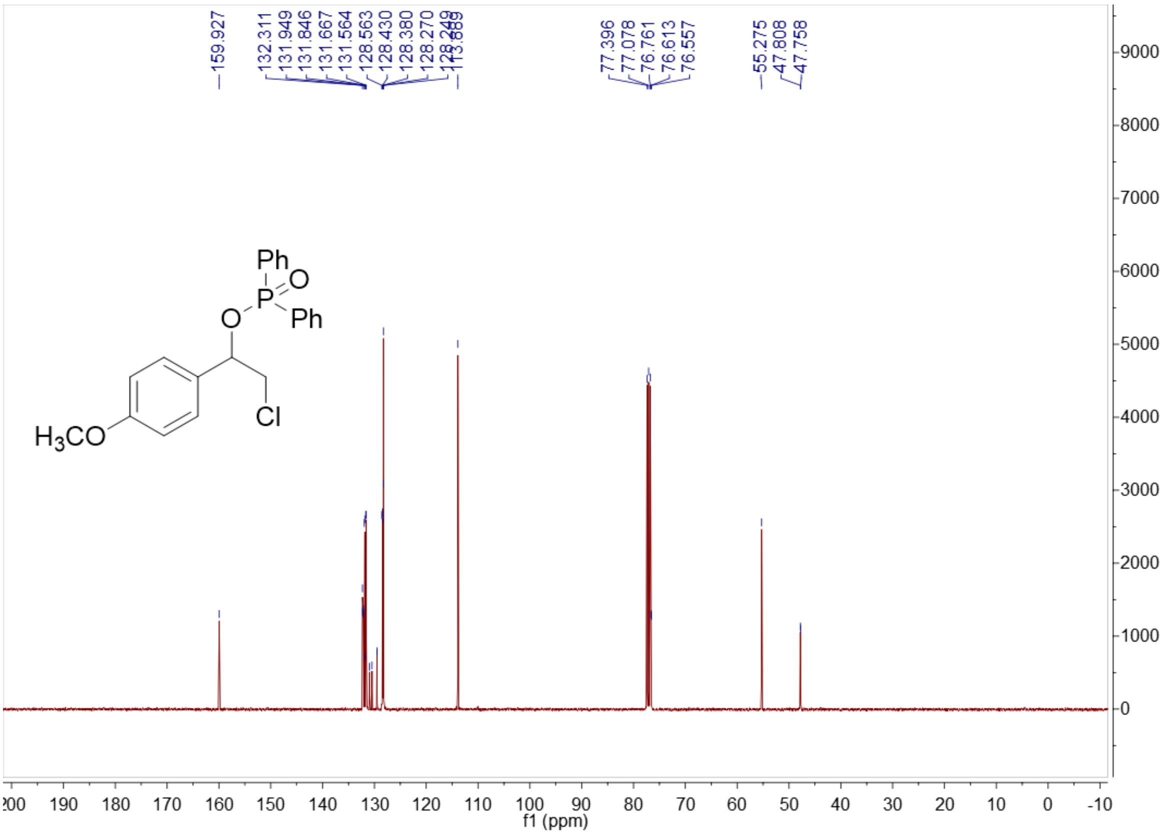

^{1}H NMR of compound **1n** in $CDCl_3$

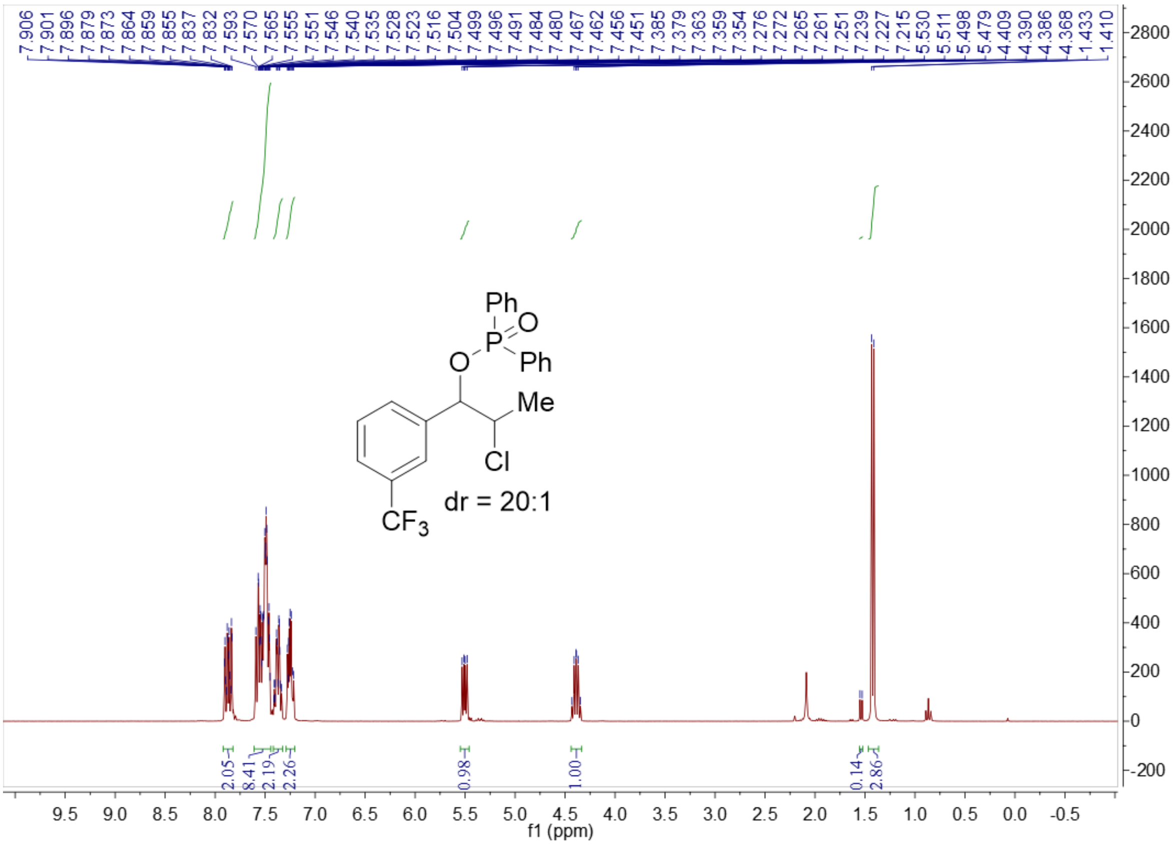

^{13}C NMR of compound **1n** in $CDCl_3$

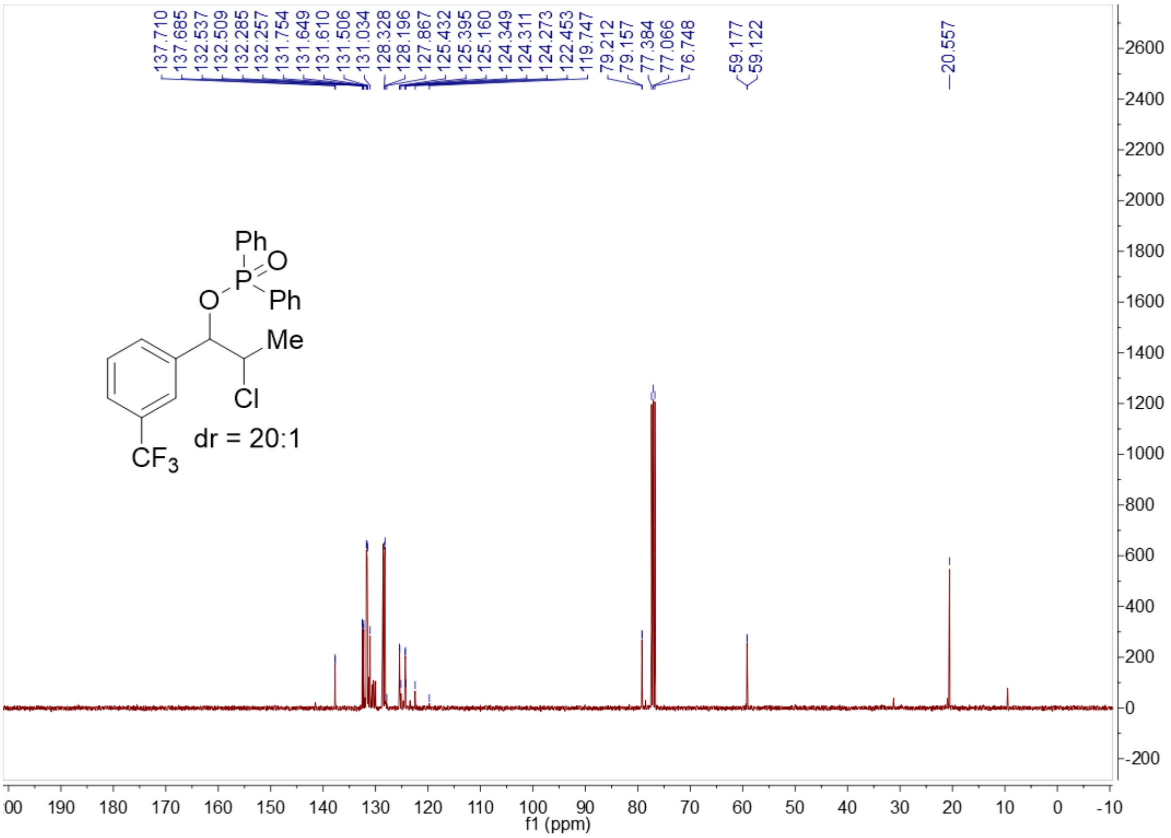

^{1}H NMR of compound **1o** in $CDCl_3$

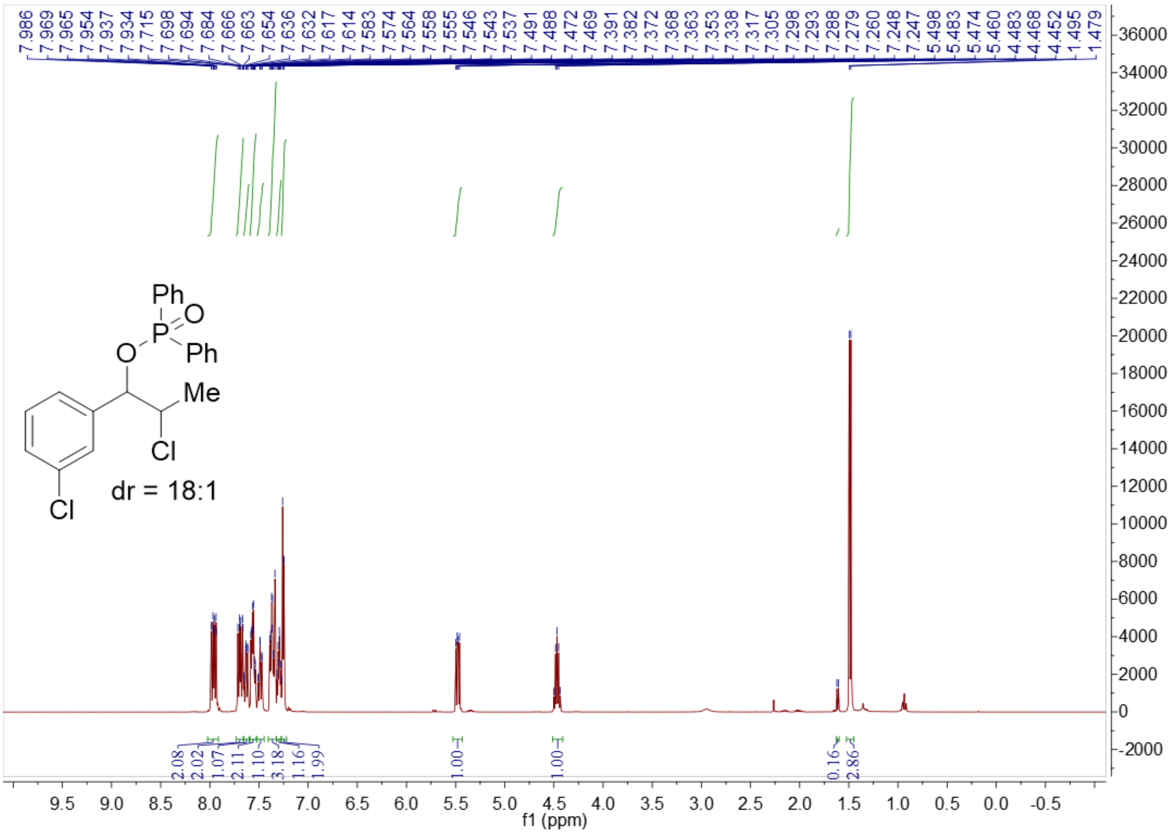

^{13}C NMR of compound **1o** in $CDCl_3$

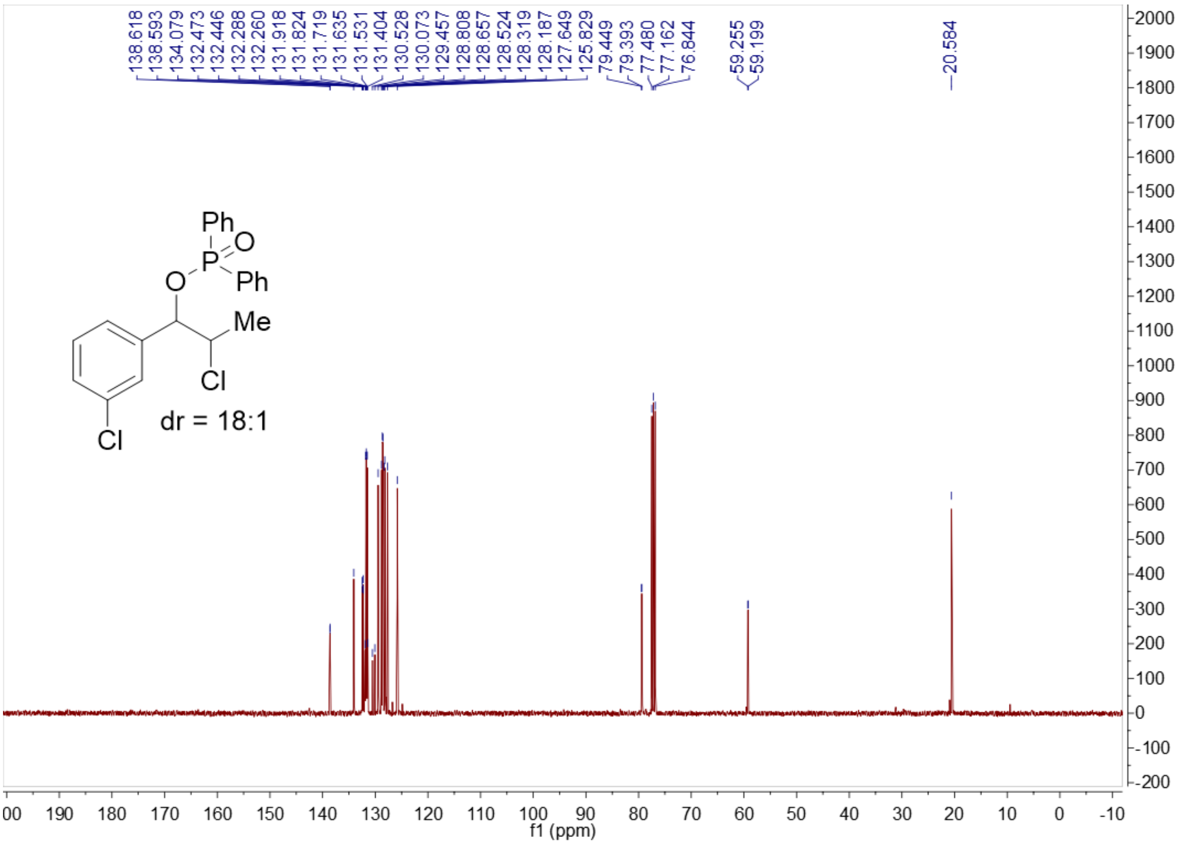

^{1}H NMR of compound **1p** in $CDCl_3$

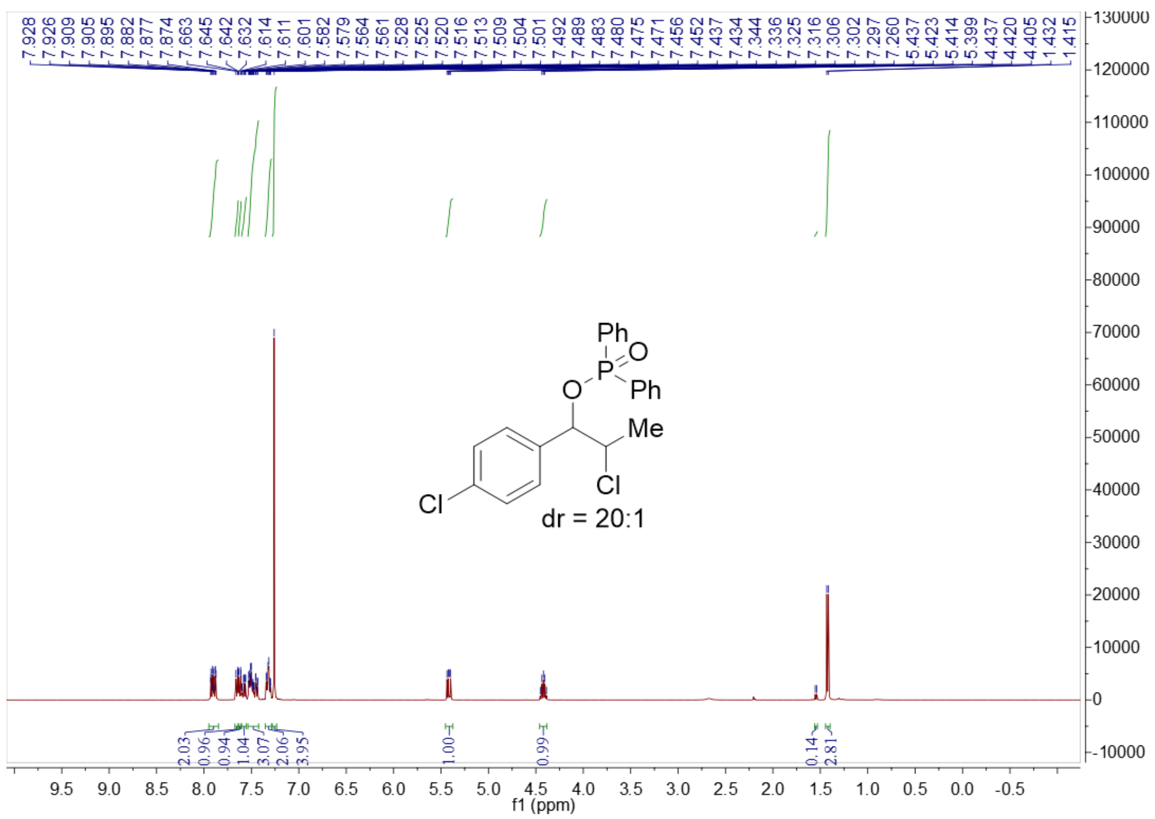

^{13}C NMR of compound **1p** in $CDCl_3$

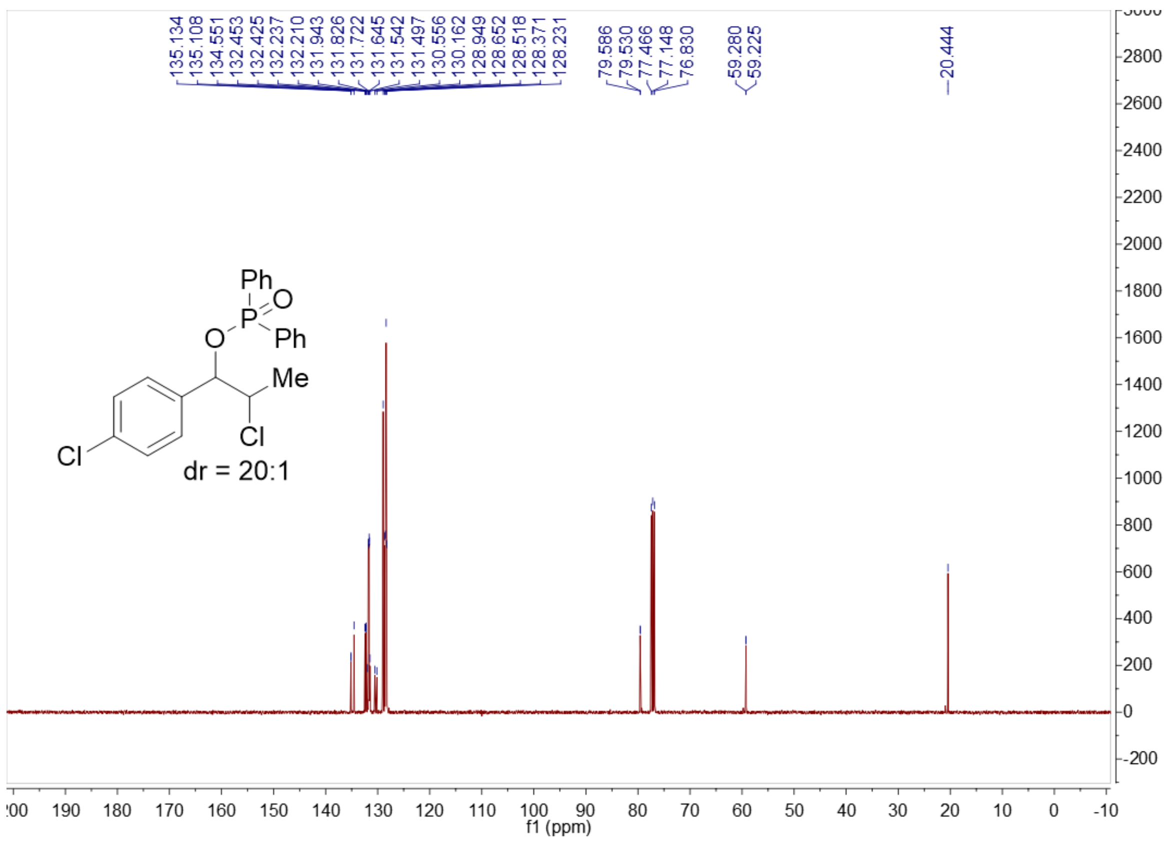

^{1}H NMR of compound **1q** in $CDCl_3$

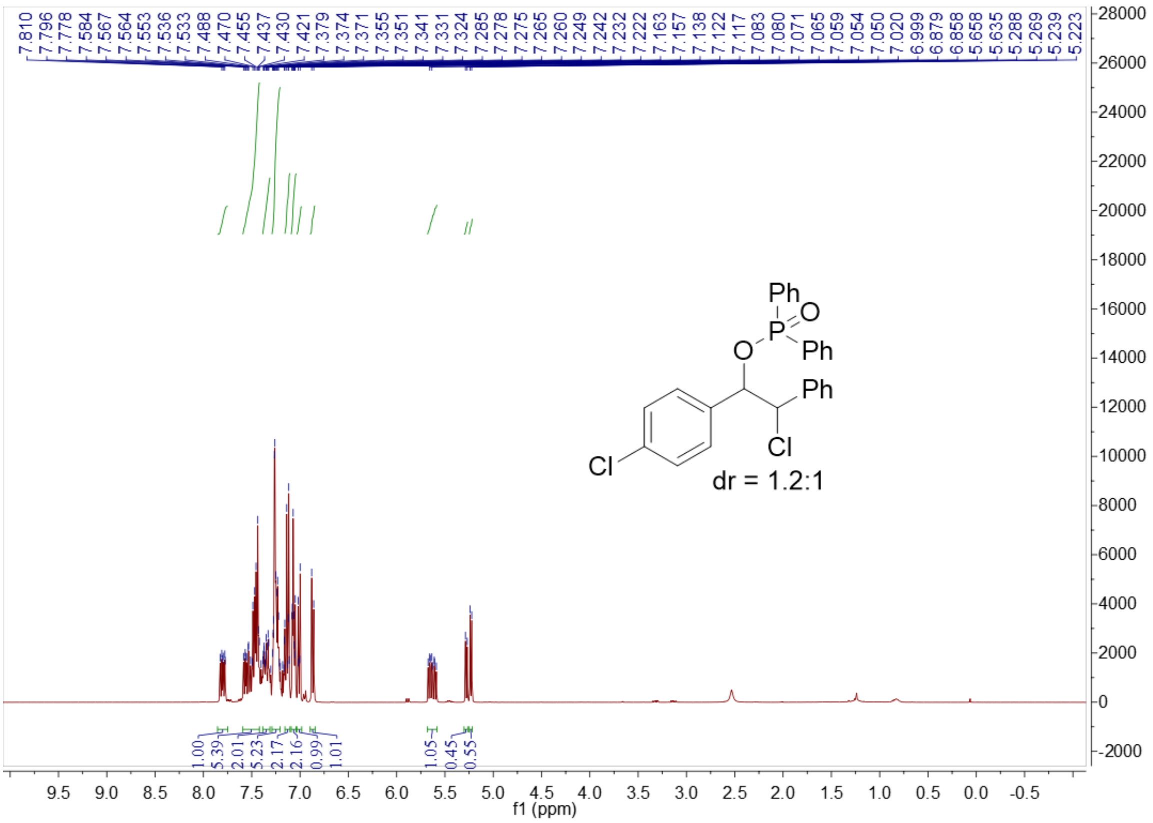

^{13}C NMR of compound **1q** in $CDCl_3$

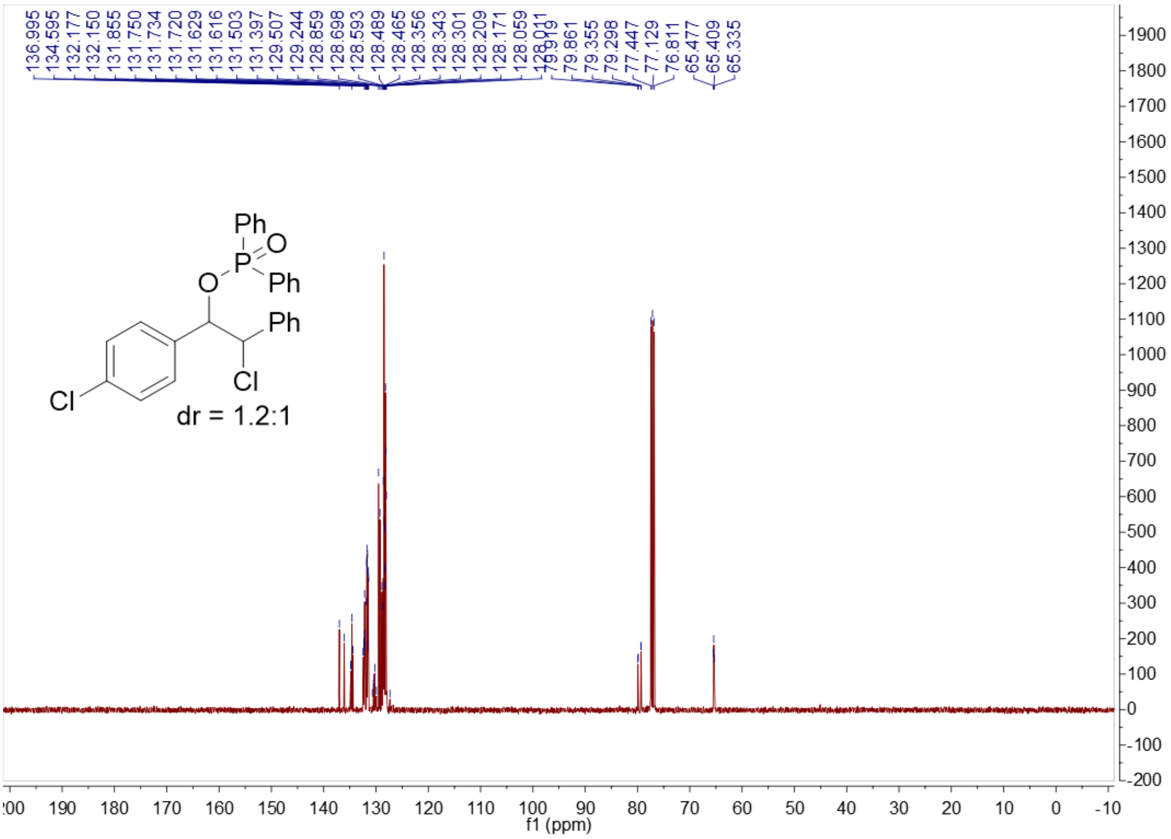

^{1}H NMR of compound **1r** in $CDCl_3$

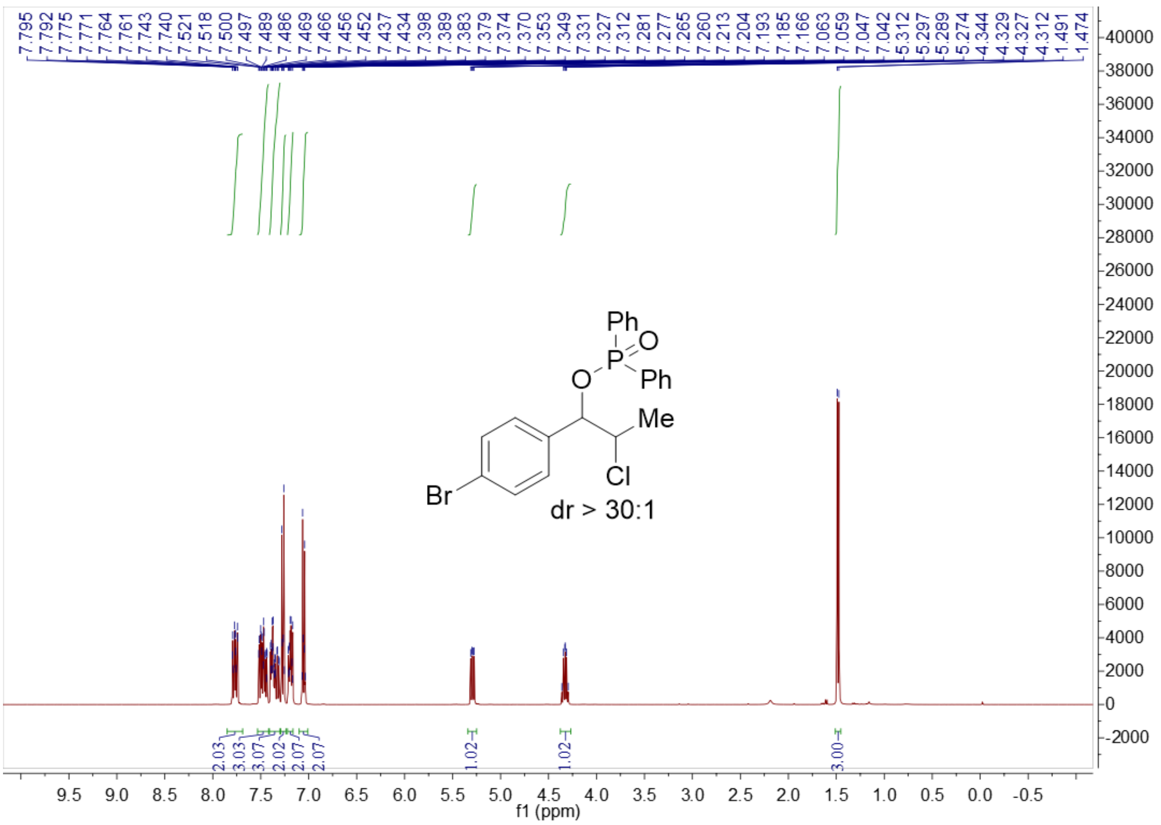

^{13}C NMR of compound **1r** in $CDCl_3$

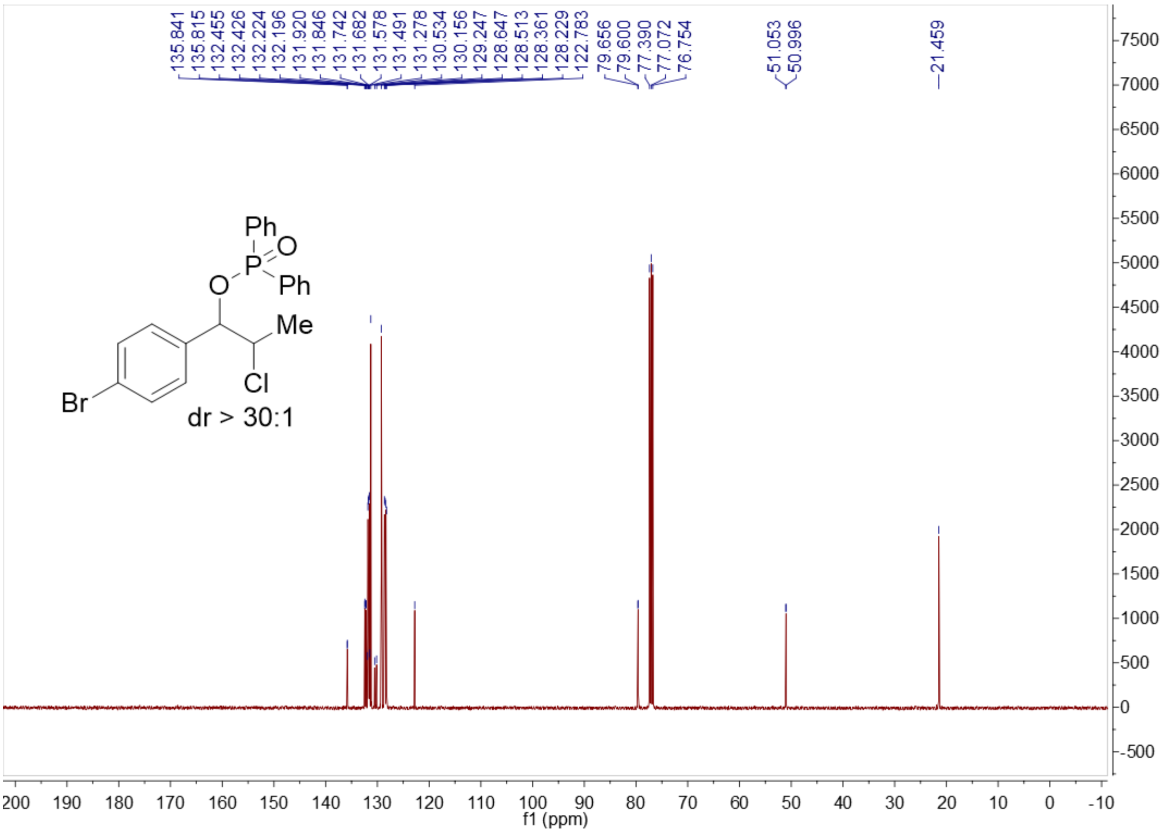

^{1}H NMR of compound **1s** in $CDCl_3$

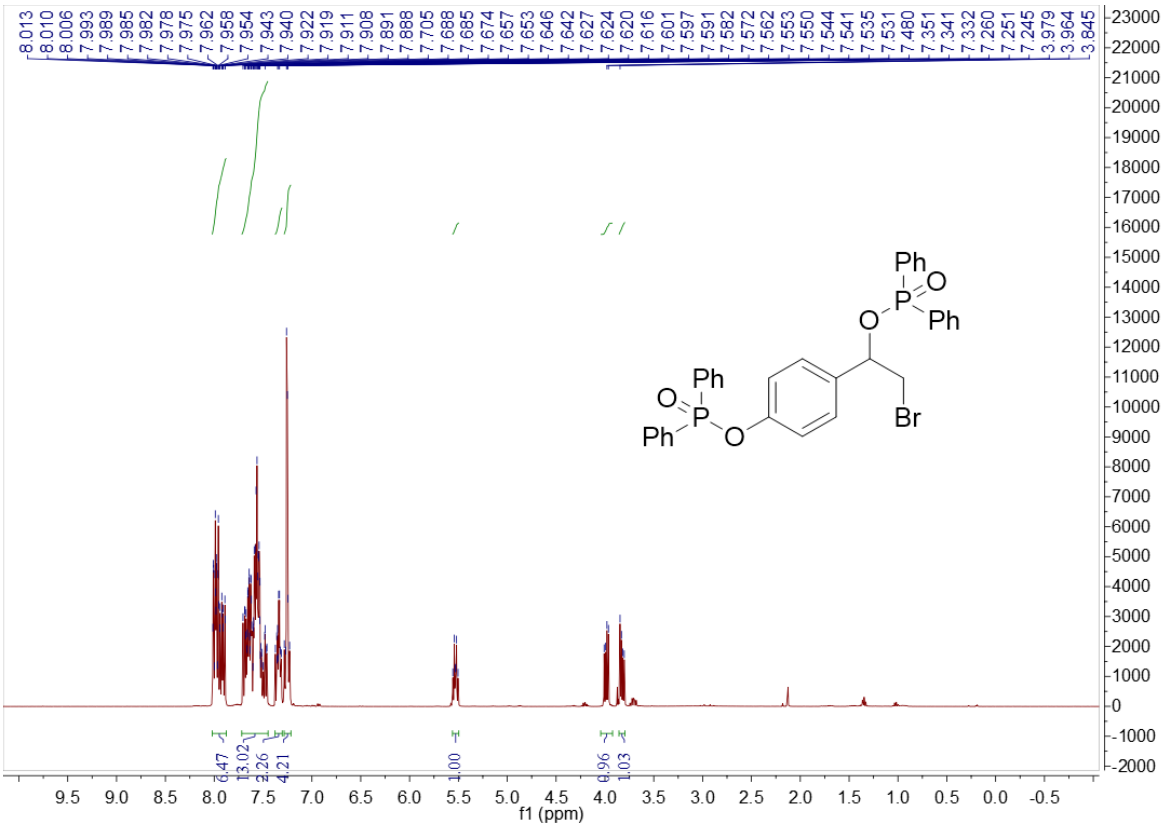

^{13}C NMR of compound **1s** in $CDCl_3$

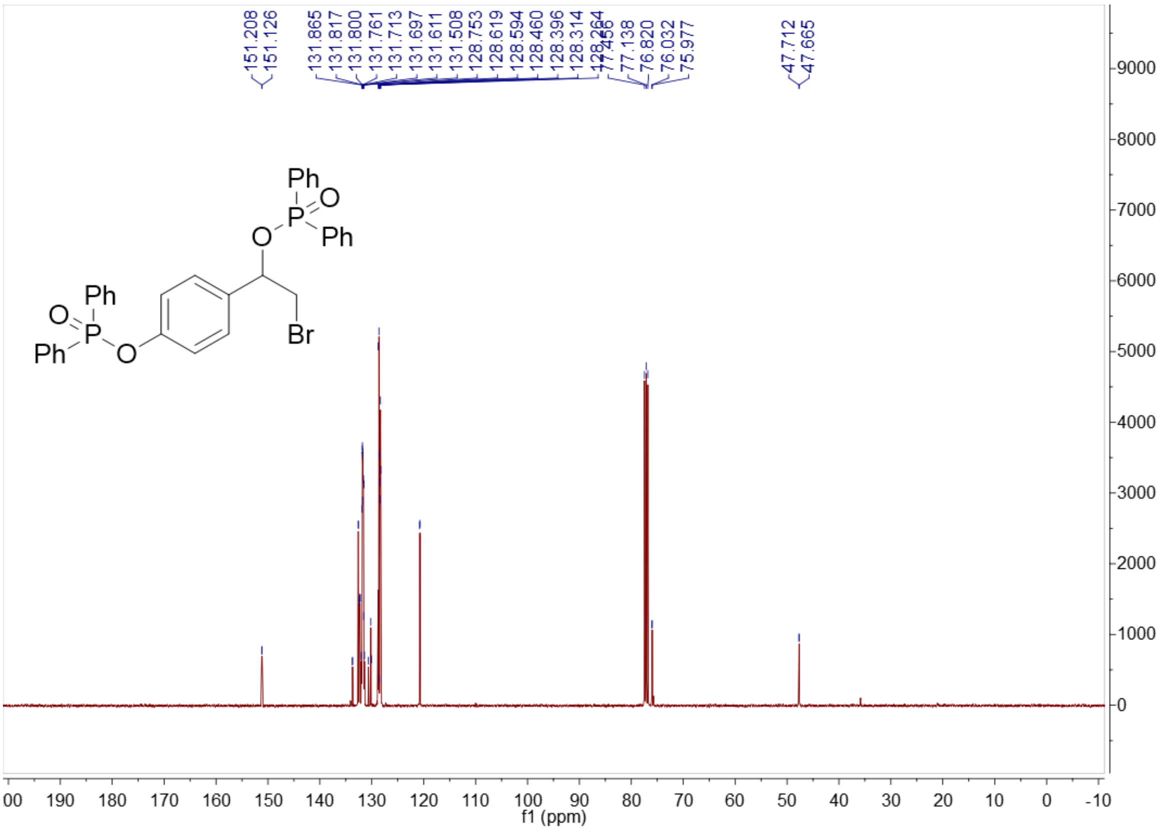

^{1}H NMR of compound **1t** in $CDCl_3$

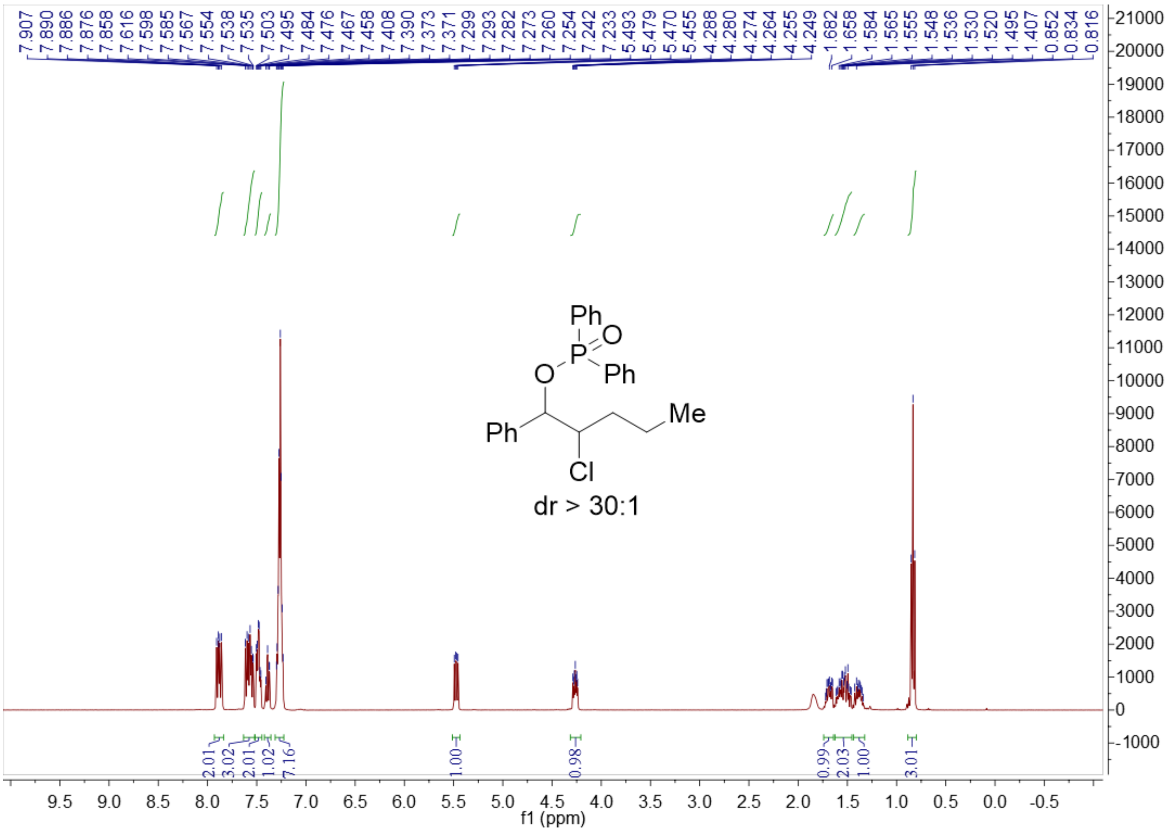

^{13}C NMR of compound **1t** in $CDCl_3$

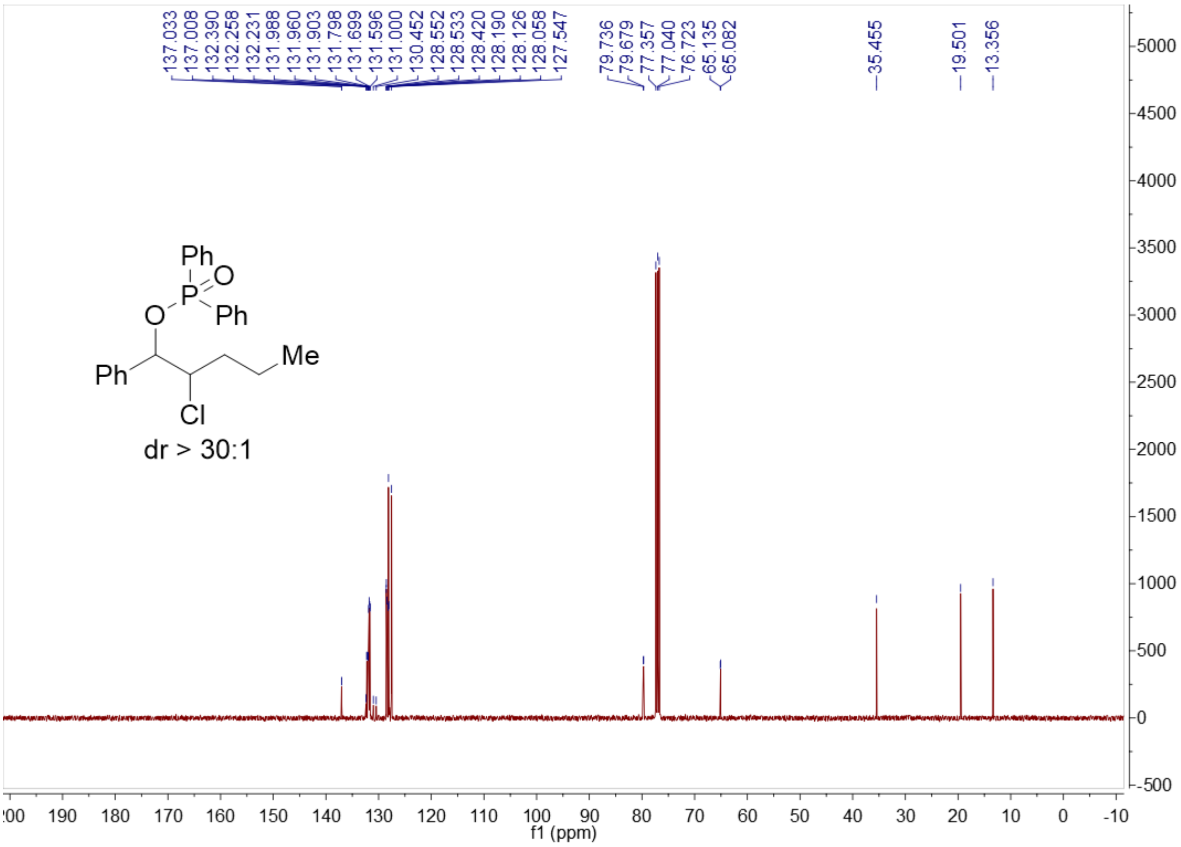

^{1}H NMR of compound **1u** in $CDCl_3$

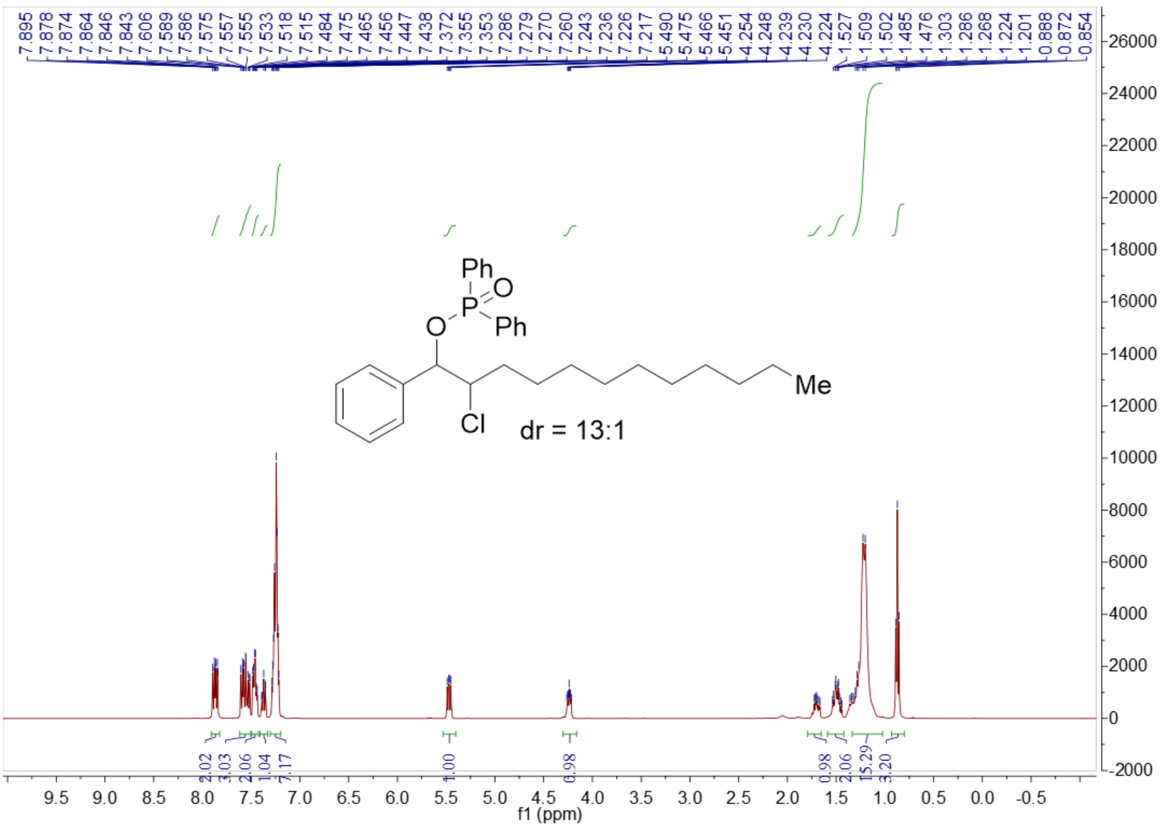

^{13}C NMR of compound **1u** in $CDCl_3$

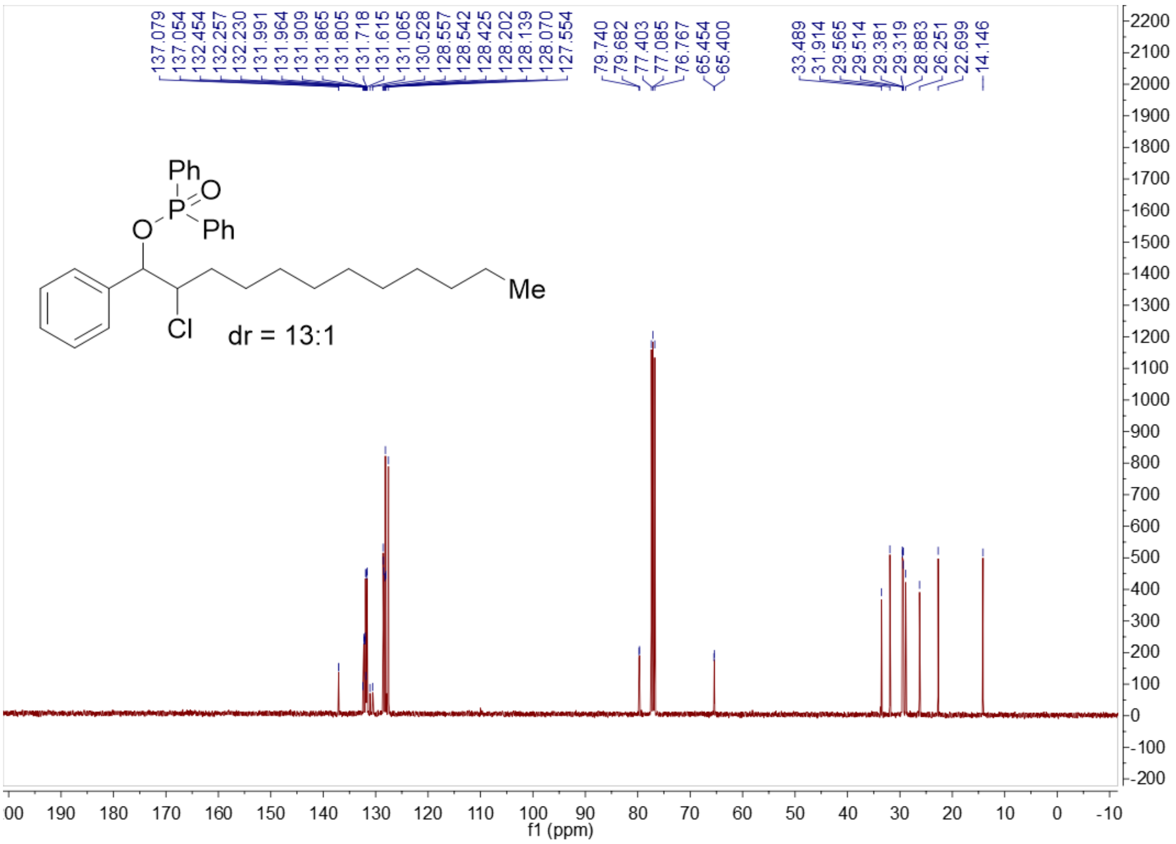

^{1}H NMR of compound **1v** in CDCl$_3$

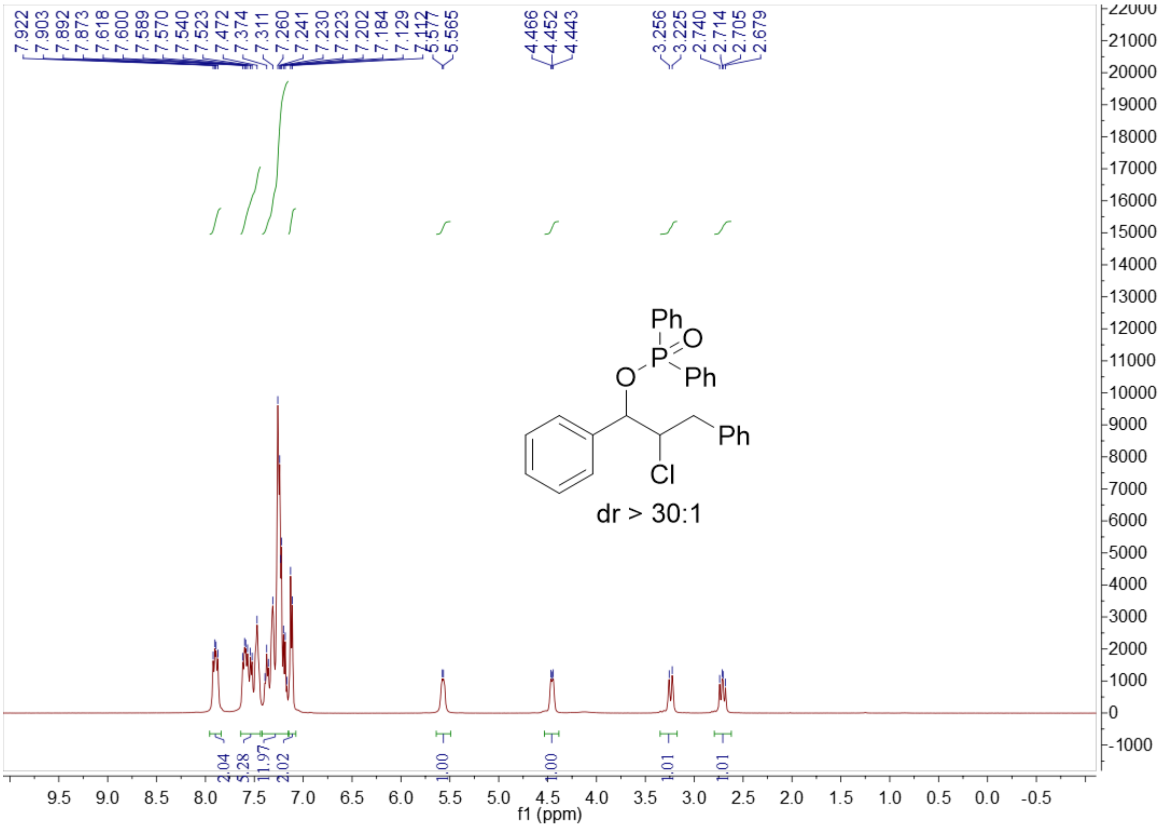

^{13}C NMR of compound **1v** in CDCl$_3$

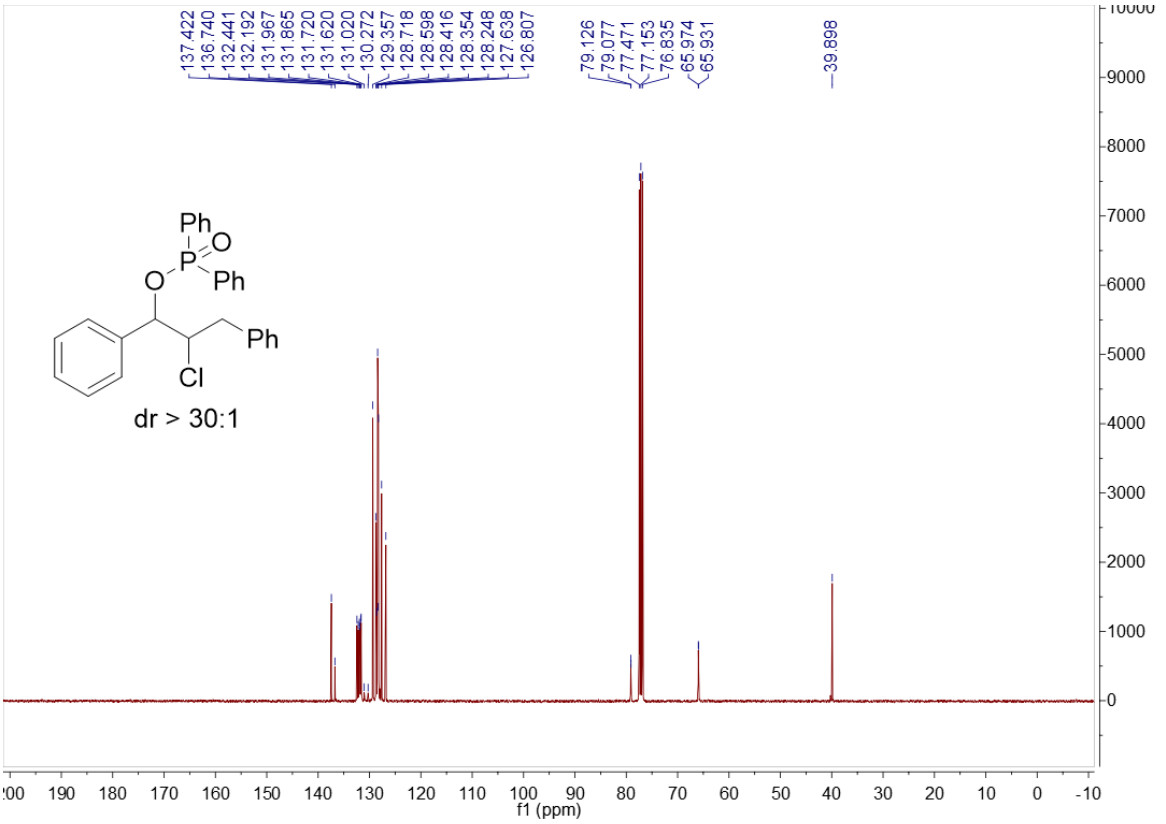

^{1}H NMR of compound **1w** in $CDCl_3$

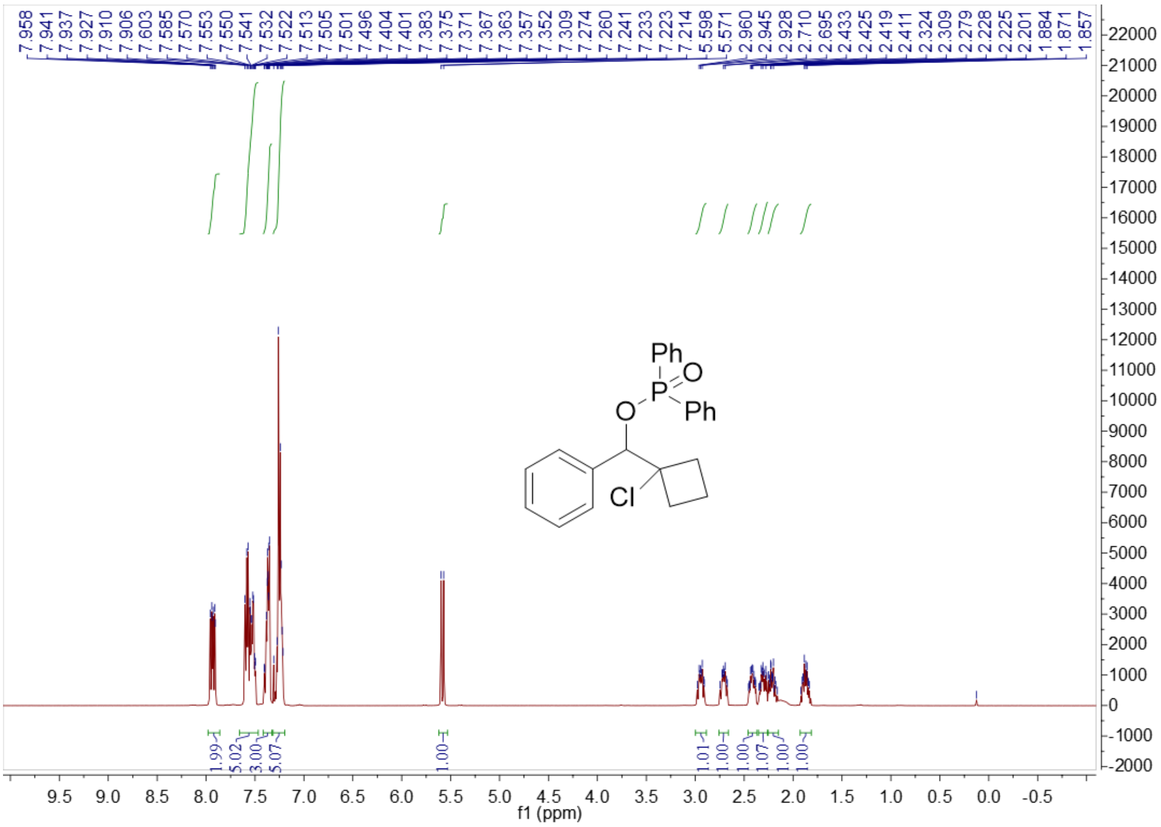

^{13}C NMR of compound **1w** in $CDCl_3$

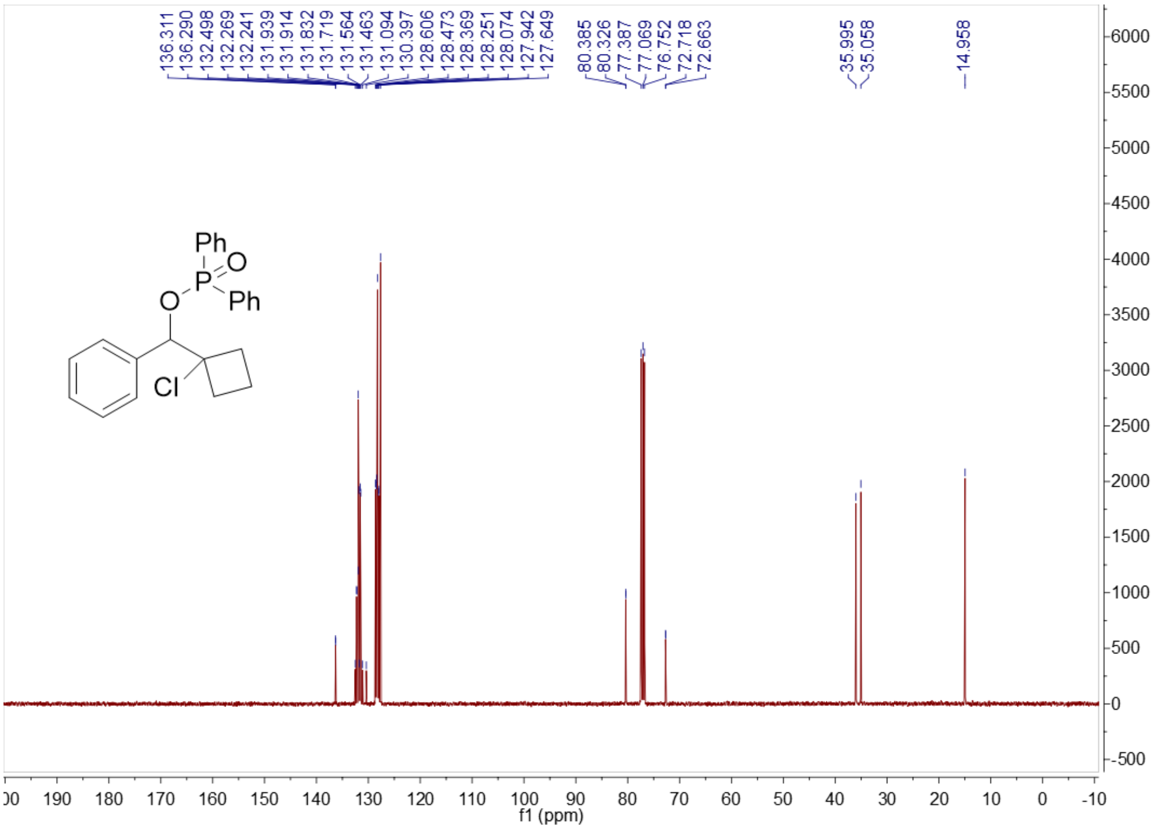

^{1}H NMR of compound **1x** in $CDCl_3$

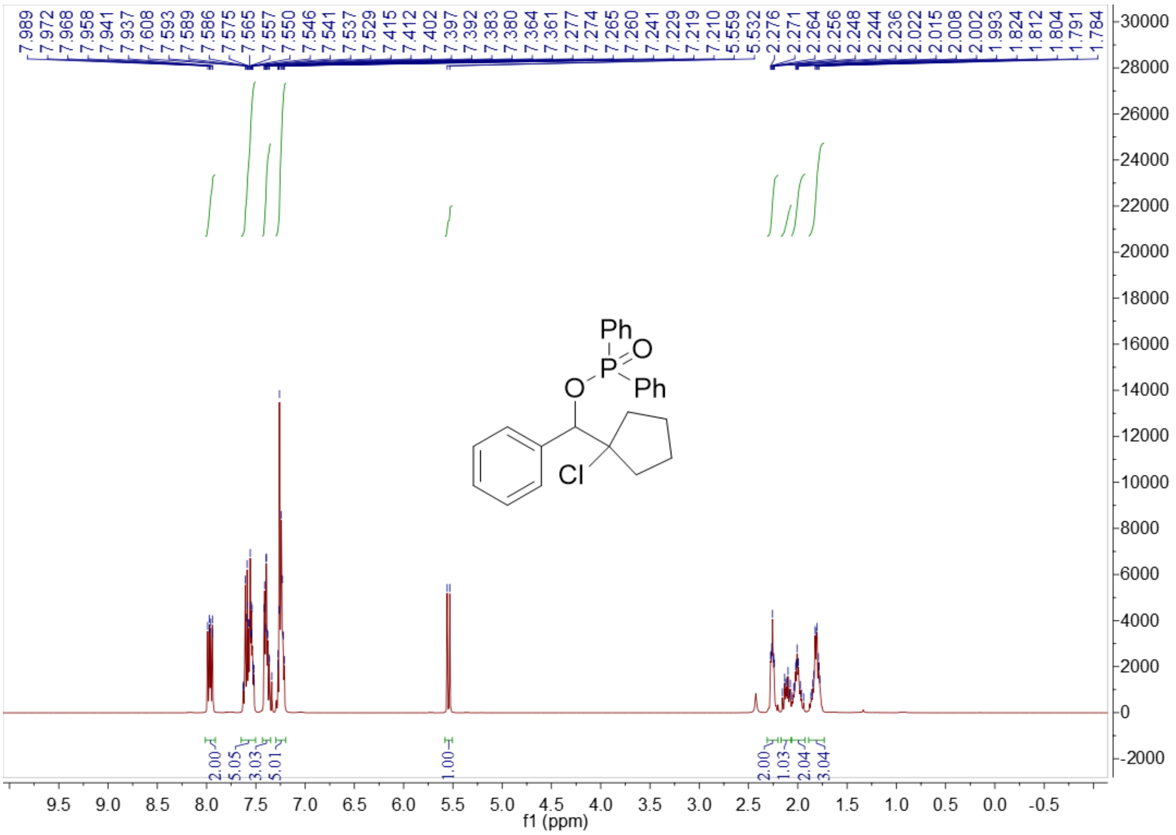

^{13}C NMR of compound **1x** in $CDCl_3$

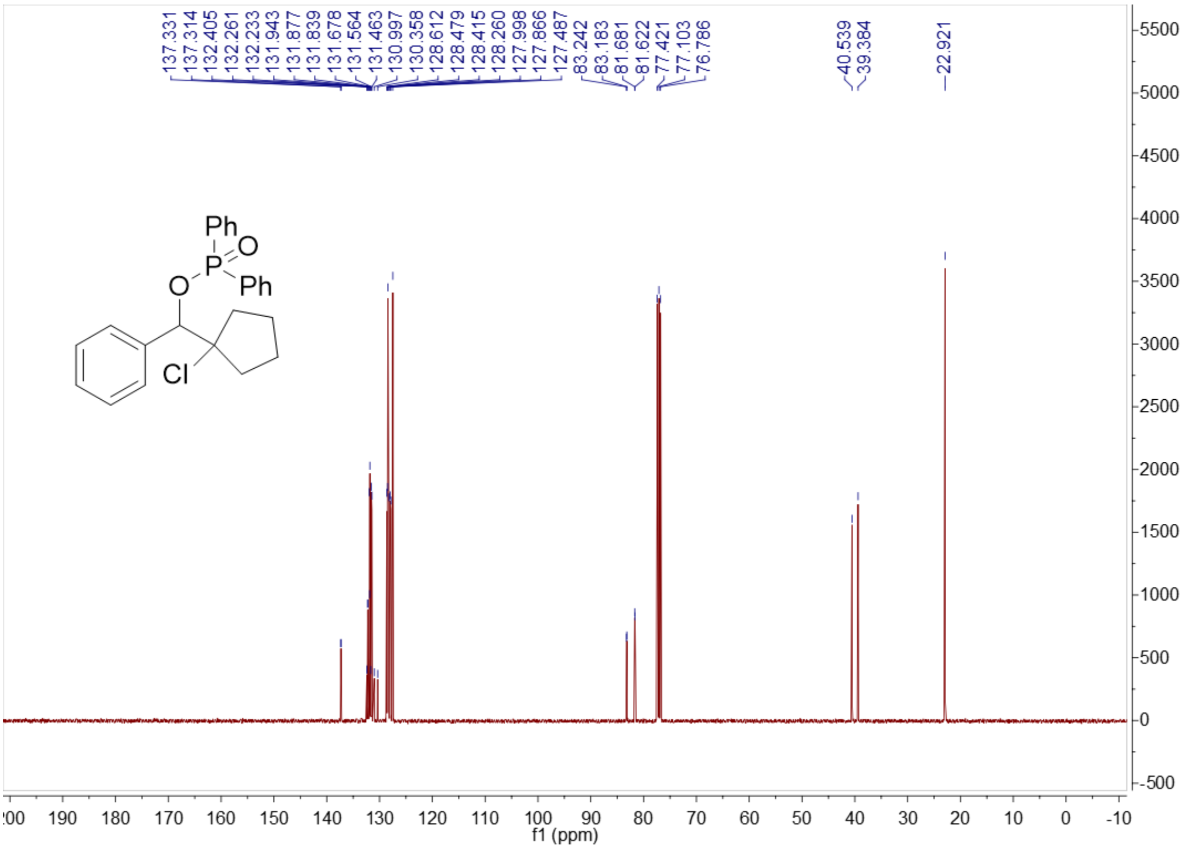

^{1}H NMR of compound **1y** in $CDCl_3$

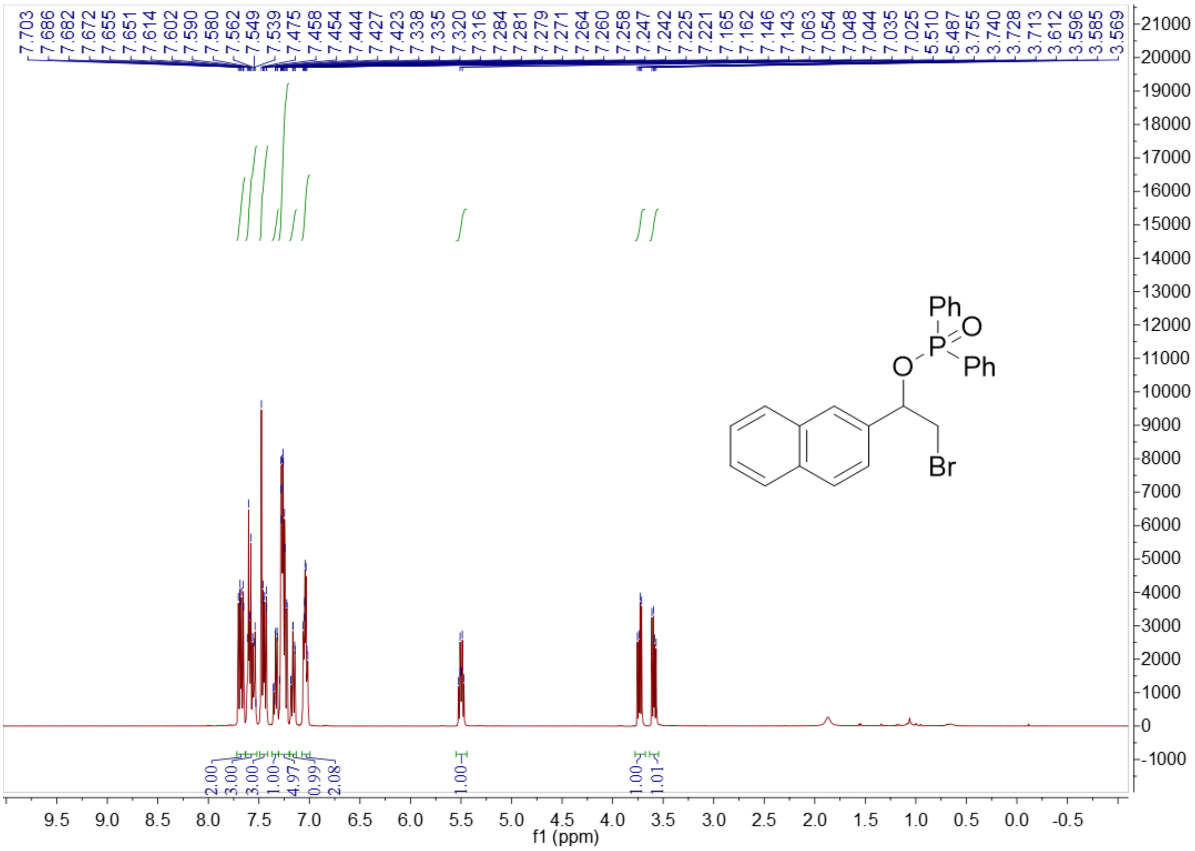

^{1}H NMR of compound **1y** in $CDCl_3$

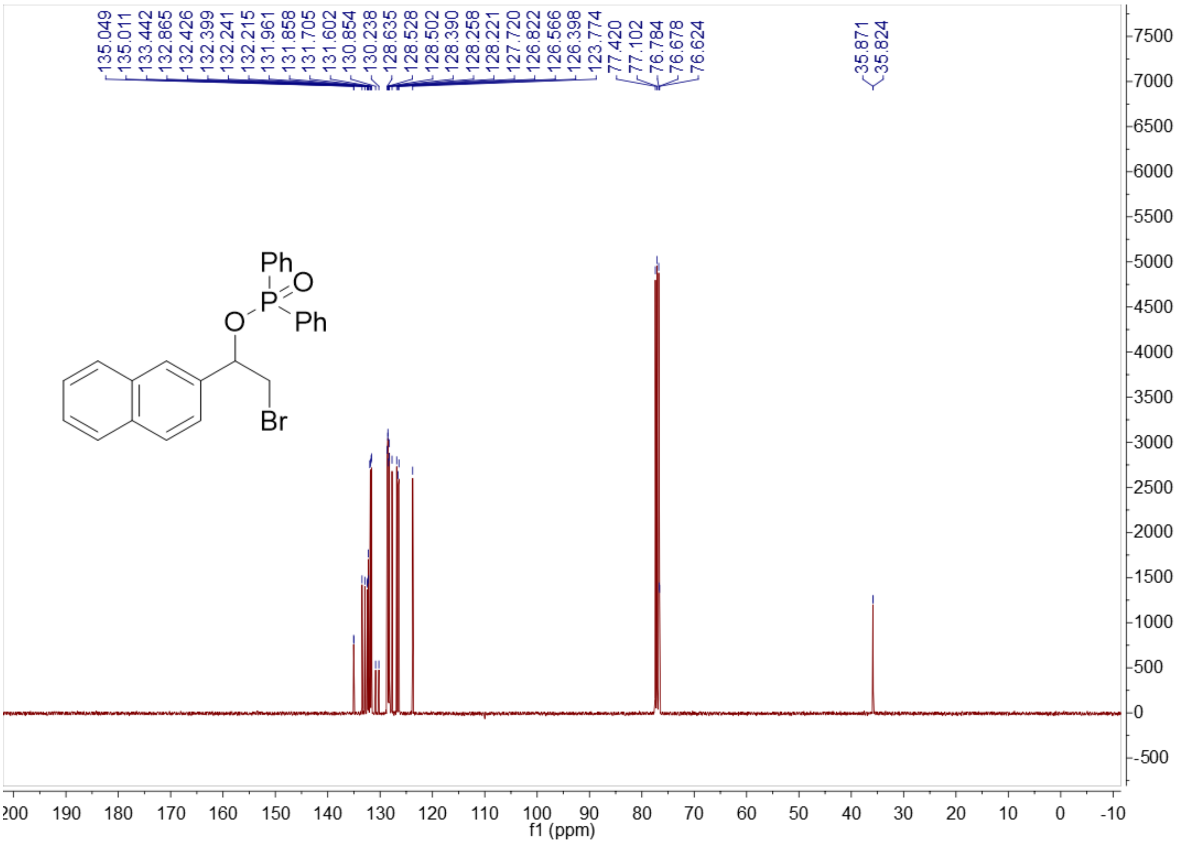

^{1}H NMR of compound **1z** in Acetone-d_6

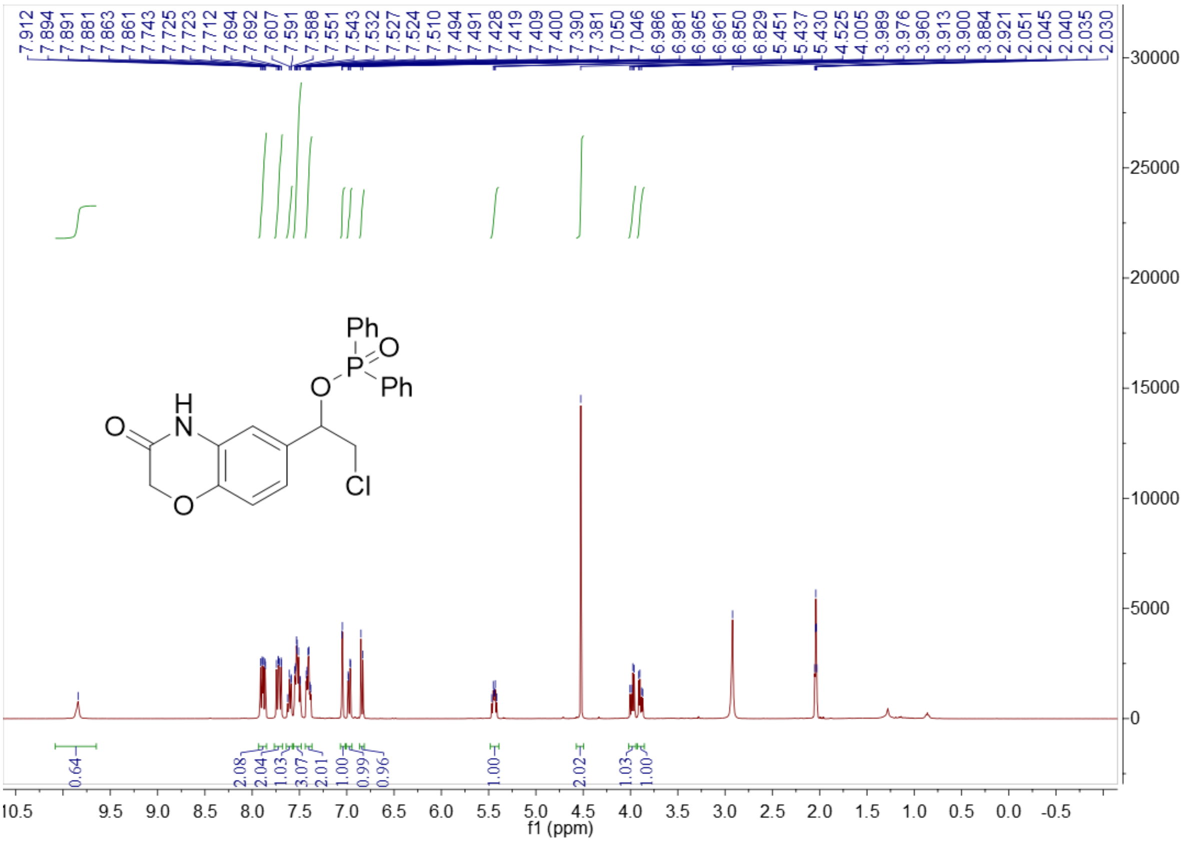

^{13}C NMR of compound **1z** in Acetone-d_6

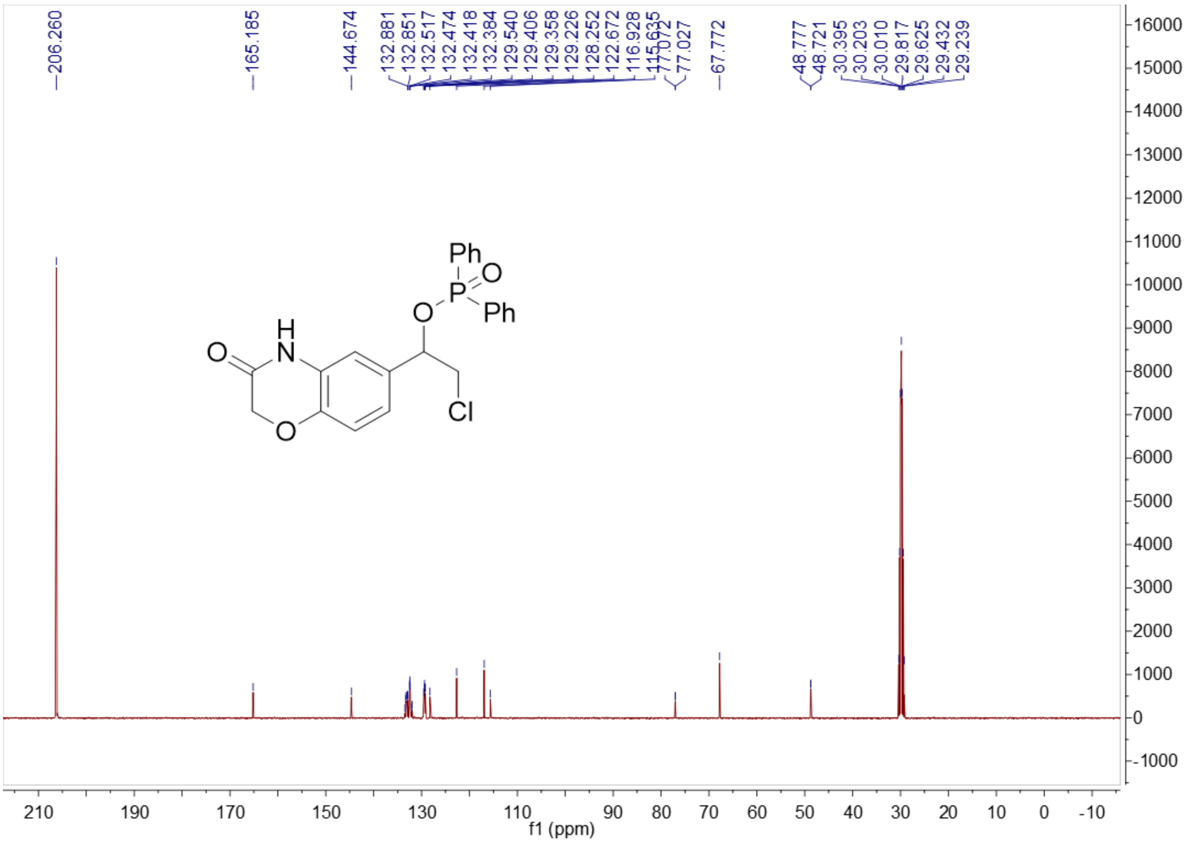

^1H NMR of compound **1aa** in $CDCl_3$

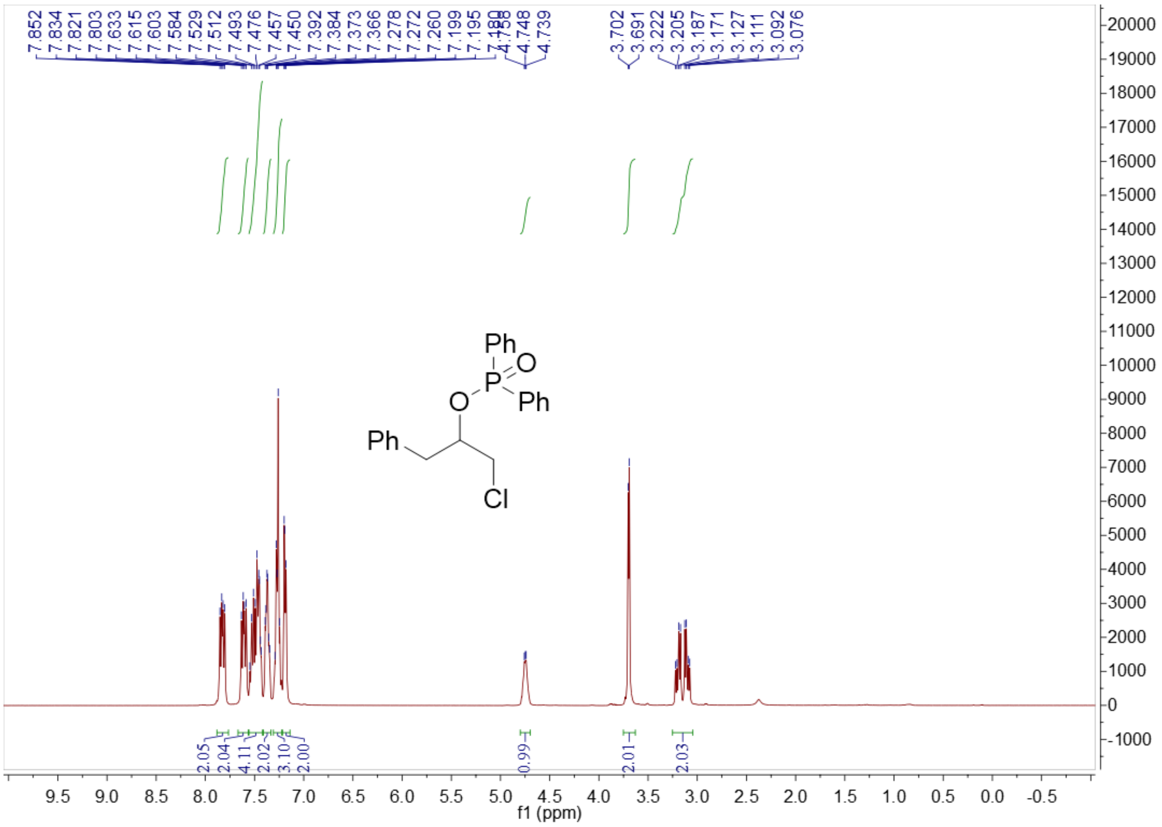

^{13}C NMR of compound **1aa** in $CDCl_3$

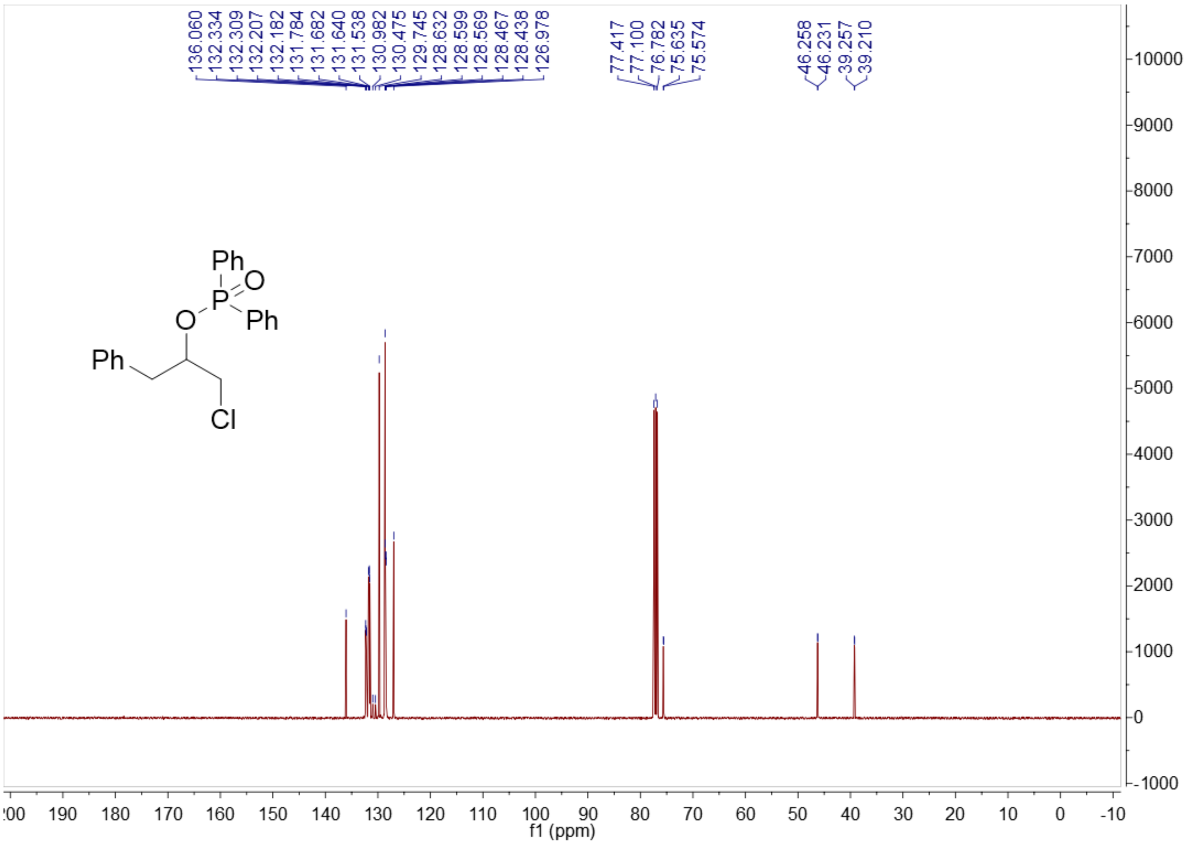

^{1}H NMR of compound **1ab** in $CDCl_3$

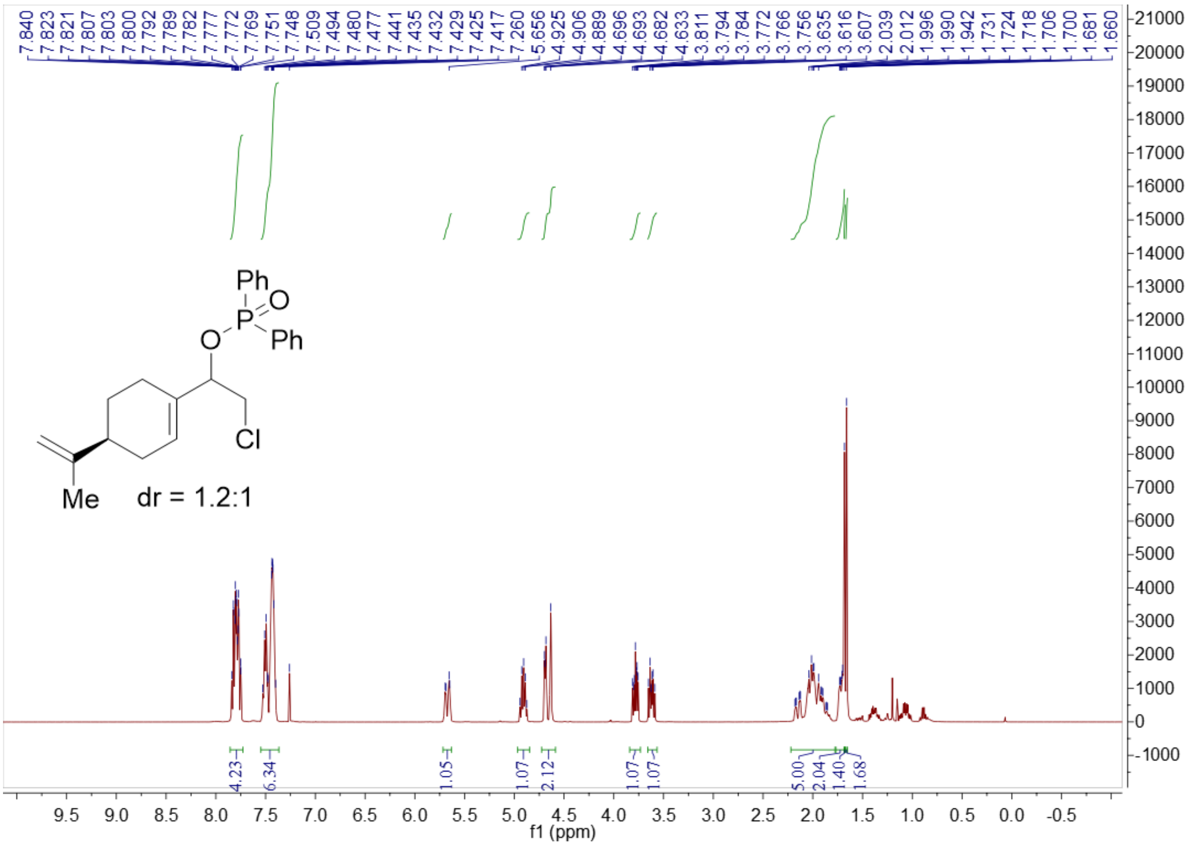

^{13}C NMR of compound **1ab** in $CDCl_3$

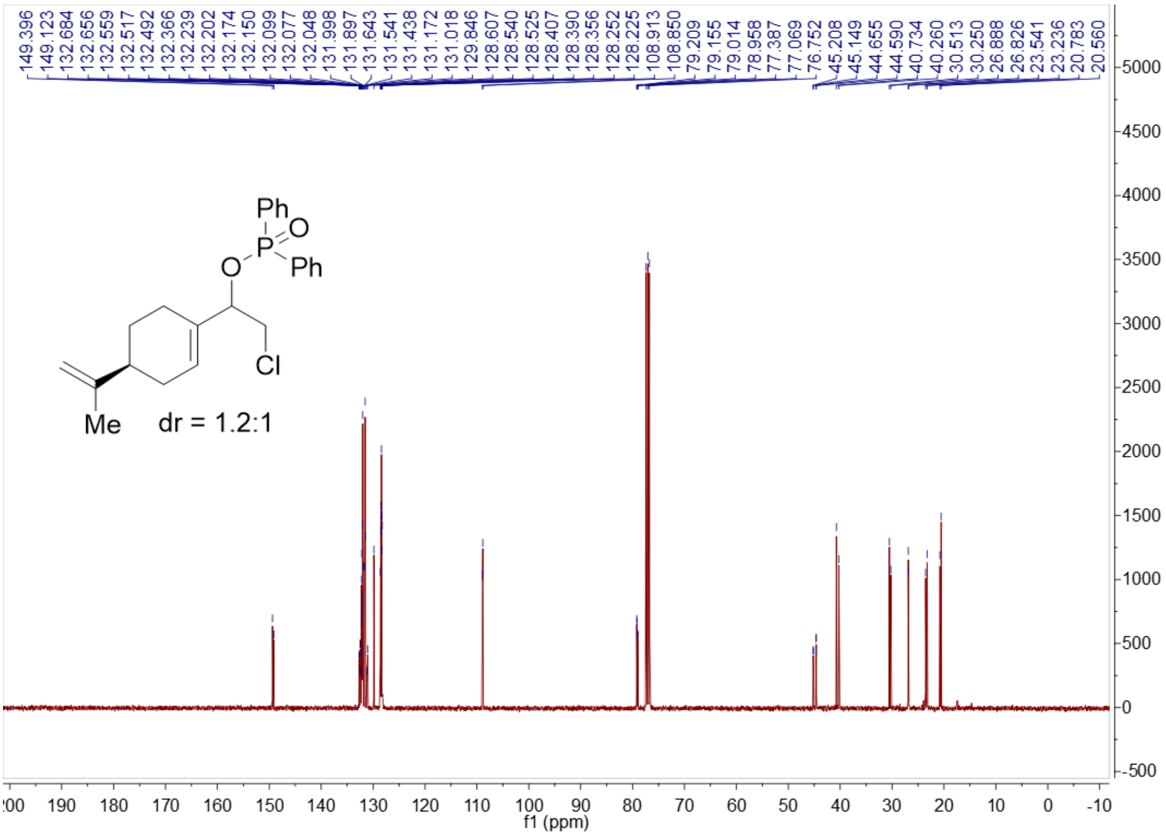

^{1}H NMR of compound **1ac** in $CDCl_3$

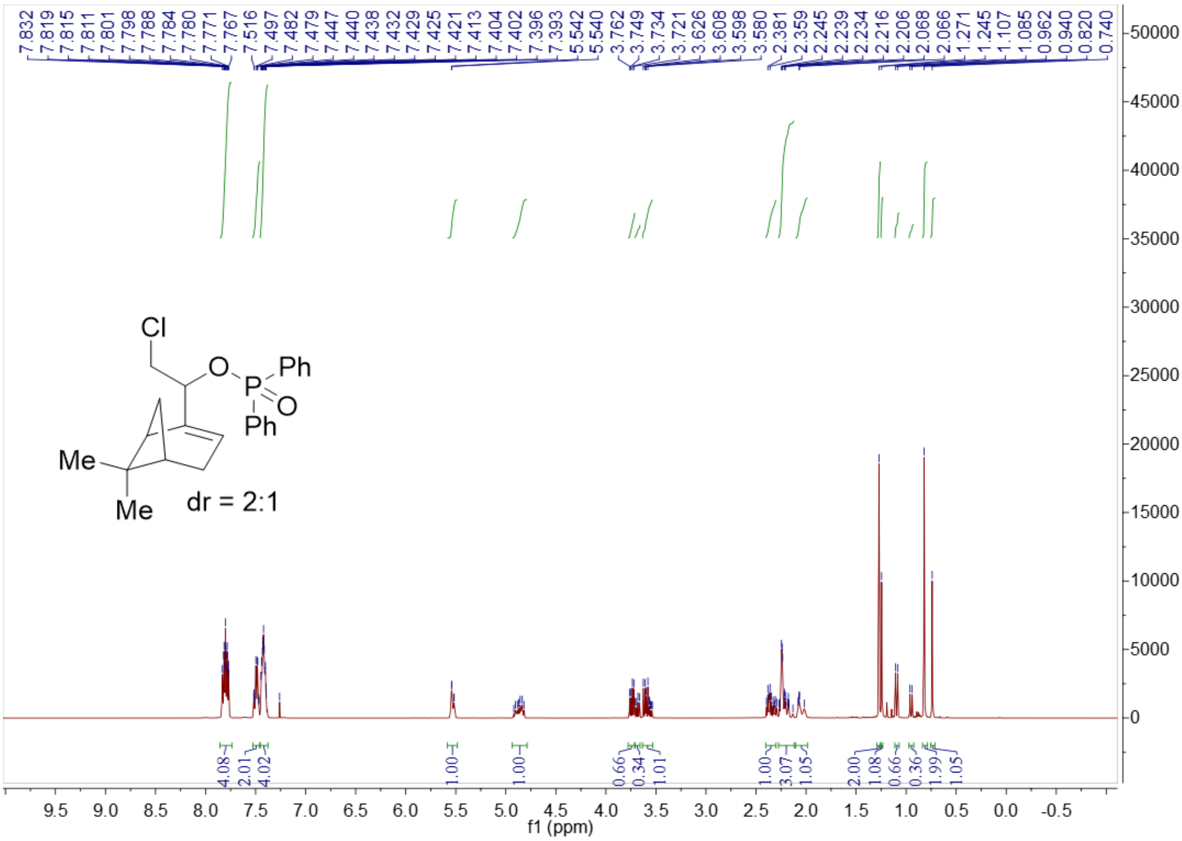

^{13}C NMR of compound **1ac** in $CDCl_3$

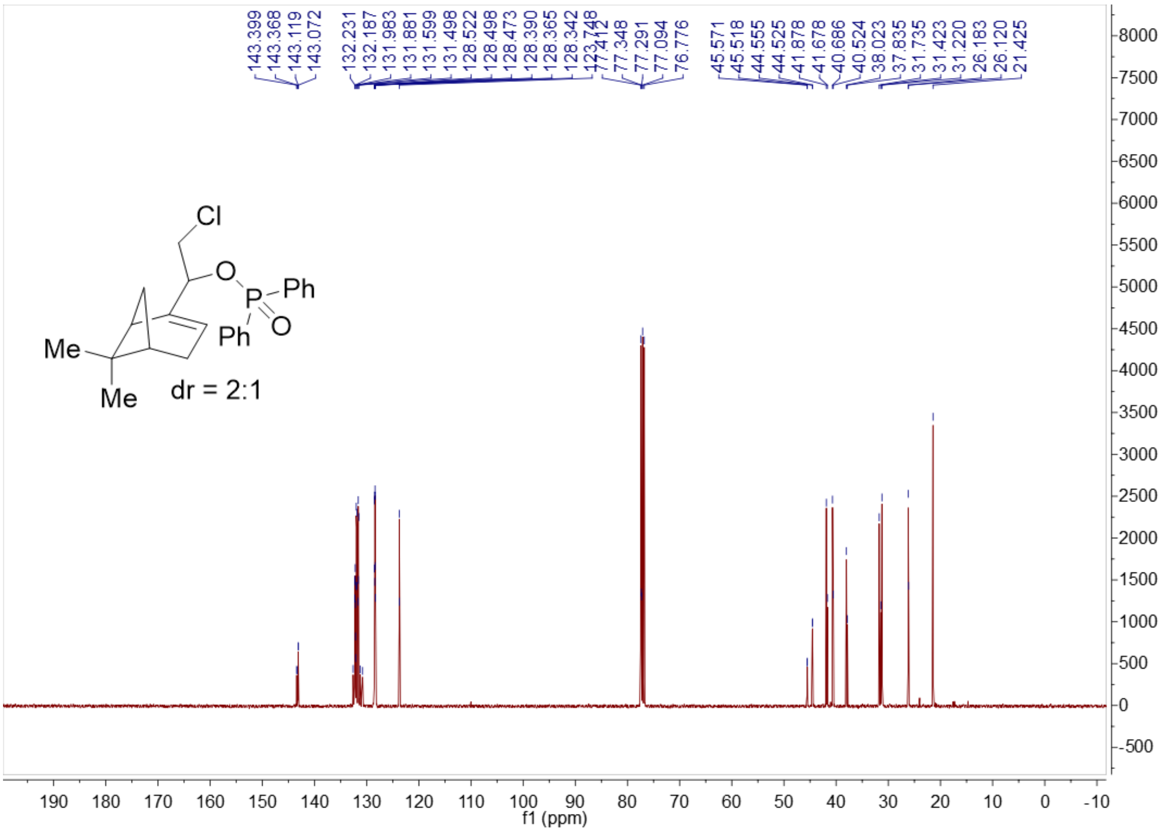

^{1}H NMR of compound **1ad** in $CDCl_3$

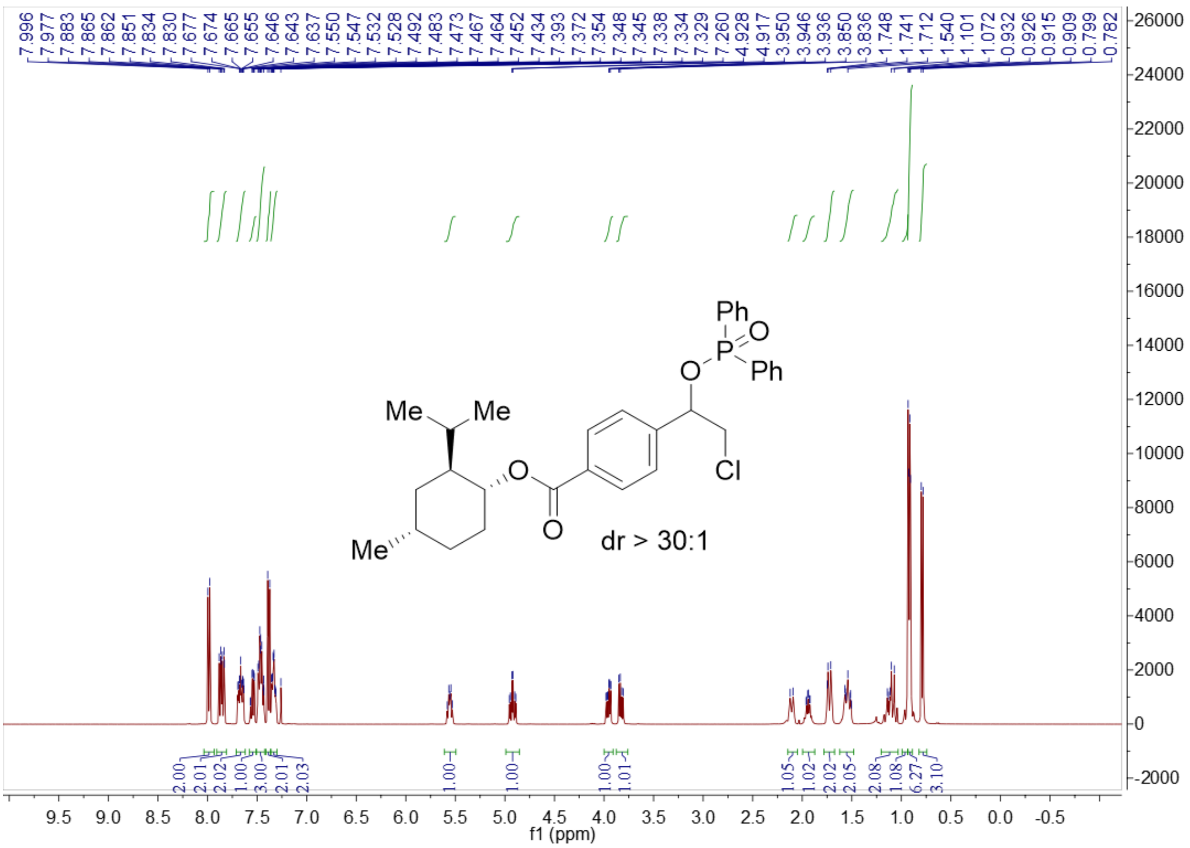

^{13}C NMR of compound **1ad** in $CDCl_3$

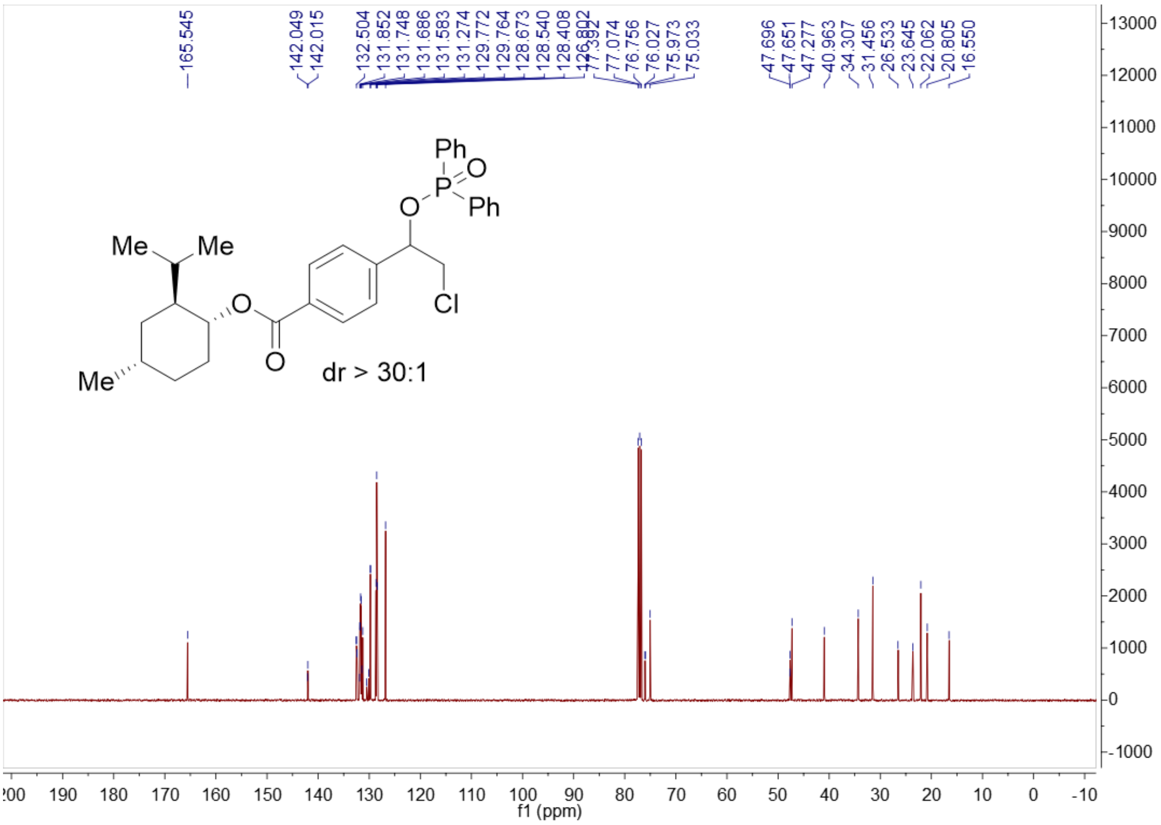

^{1}H NMR of compound **1ah** in $CDCl_3$

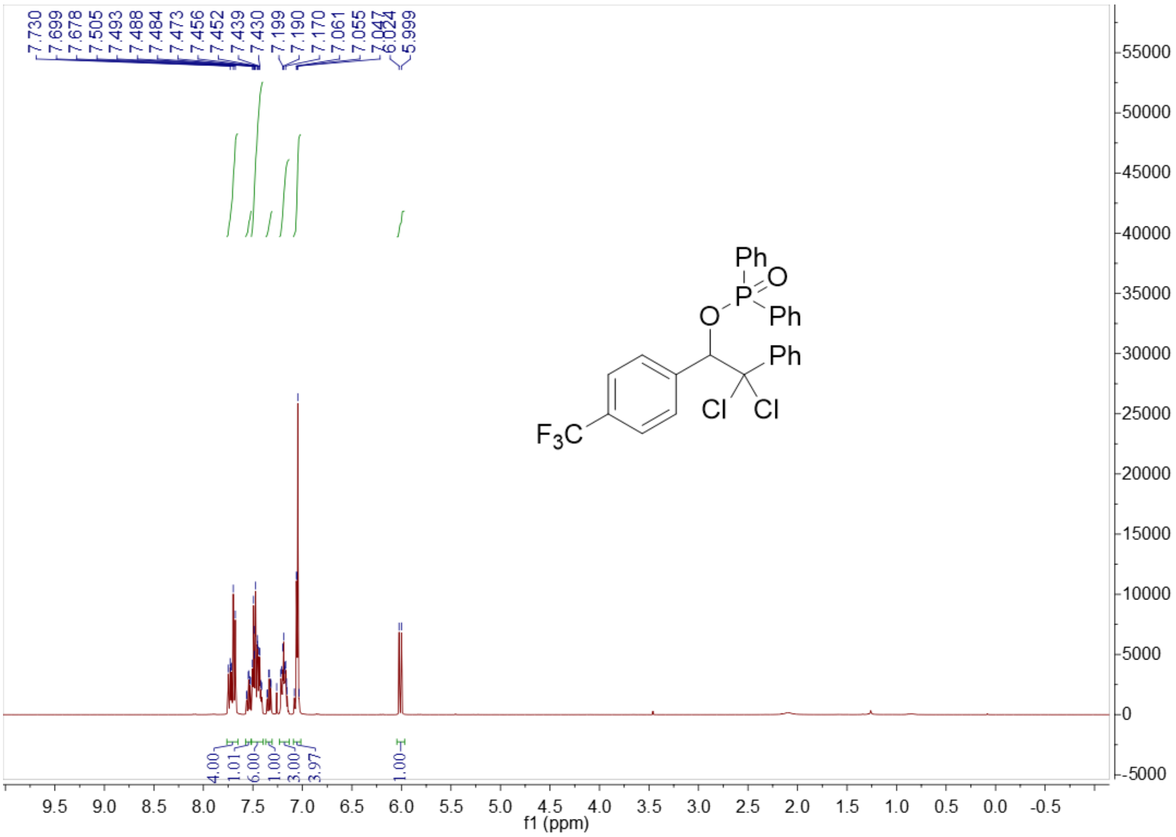

^{13}C NMR of compound **1ah** in $CDCl_3$

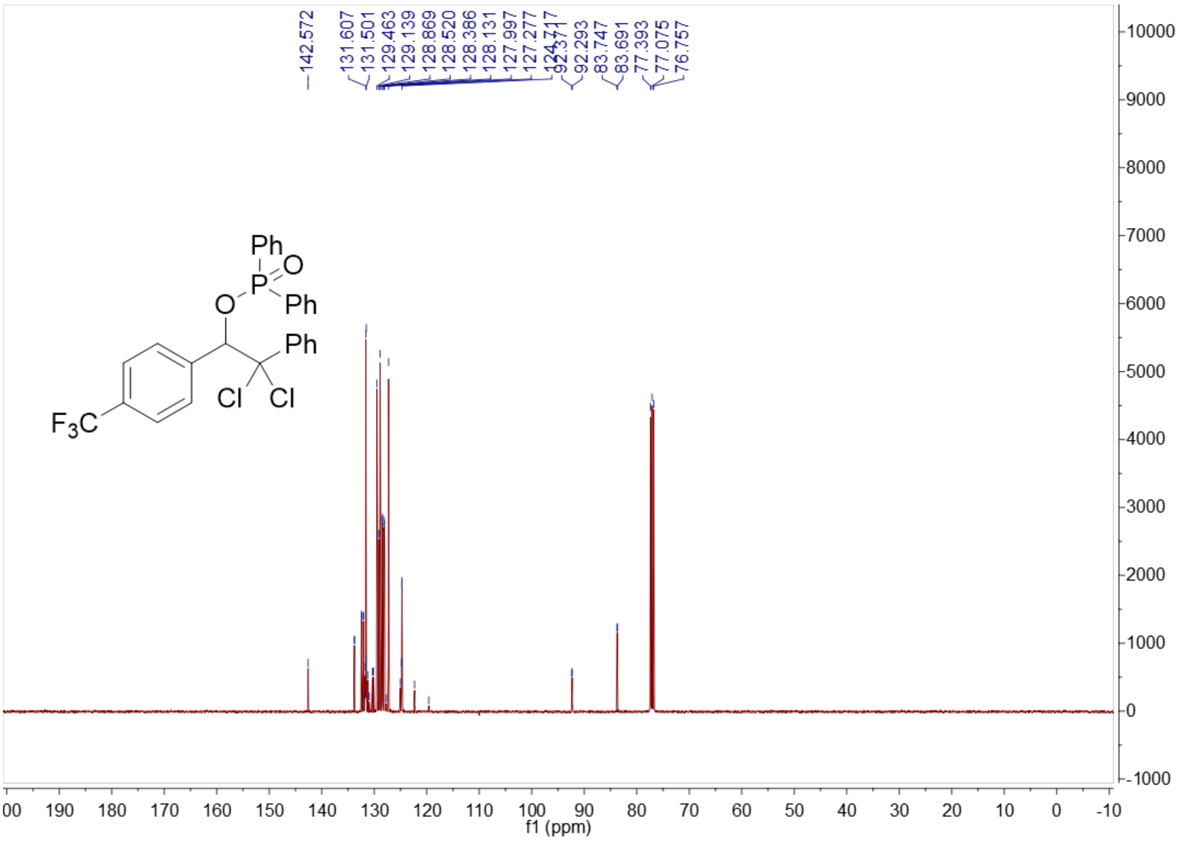

^{1}H NMR of compound **1ai** in $CDCl_3$

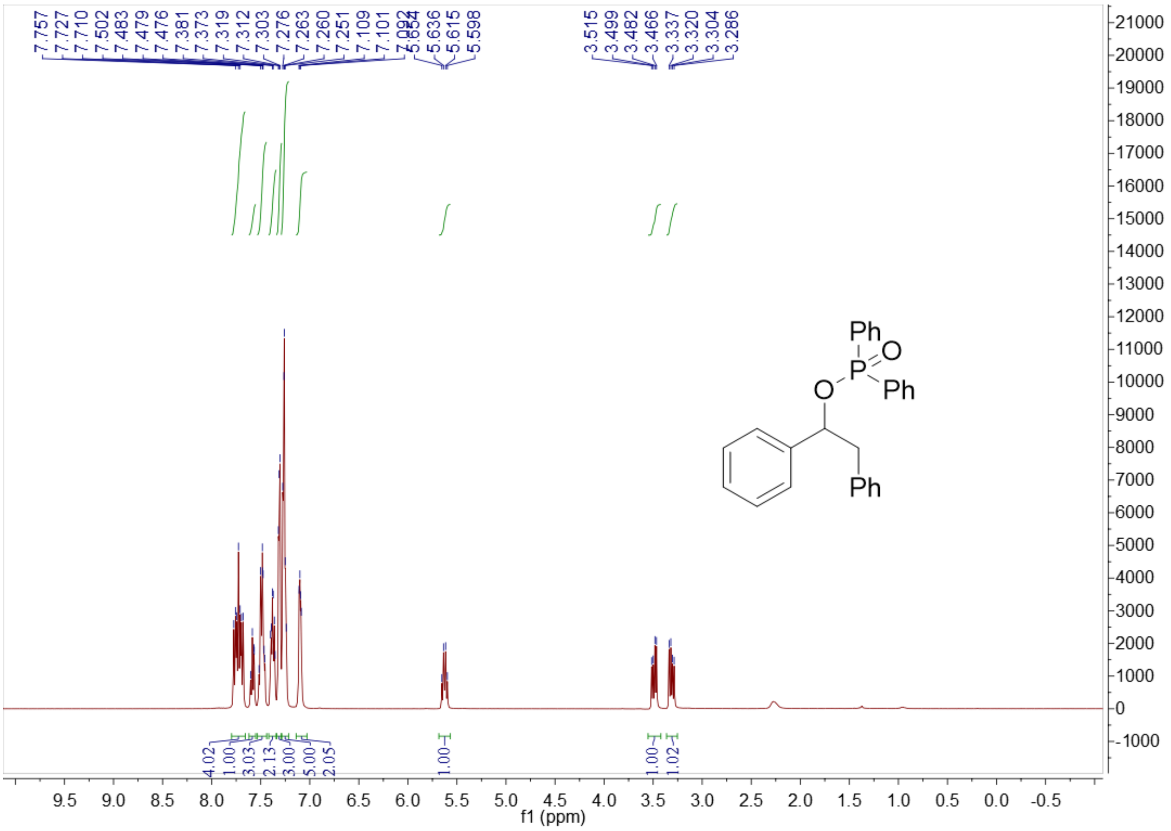

^{13}C NMR of compound **1ai** in $CDCl_3$

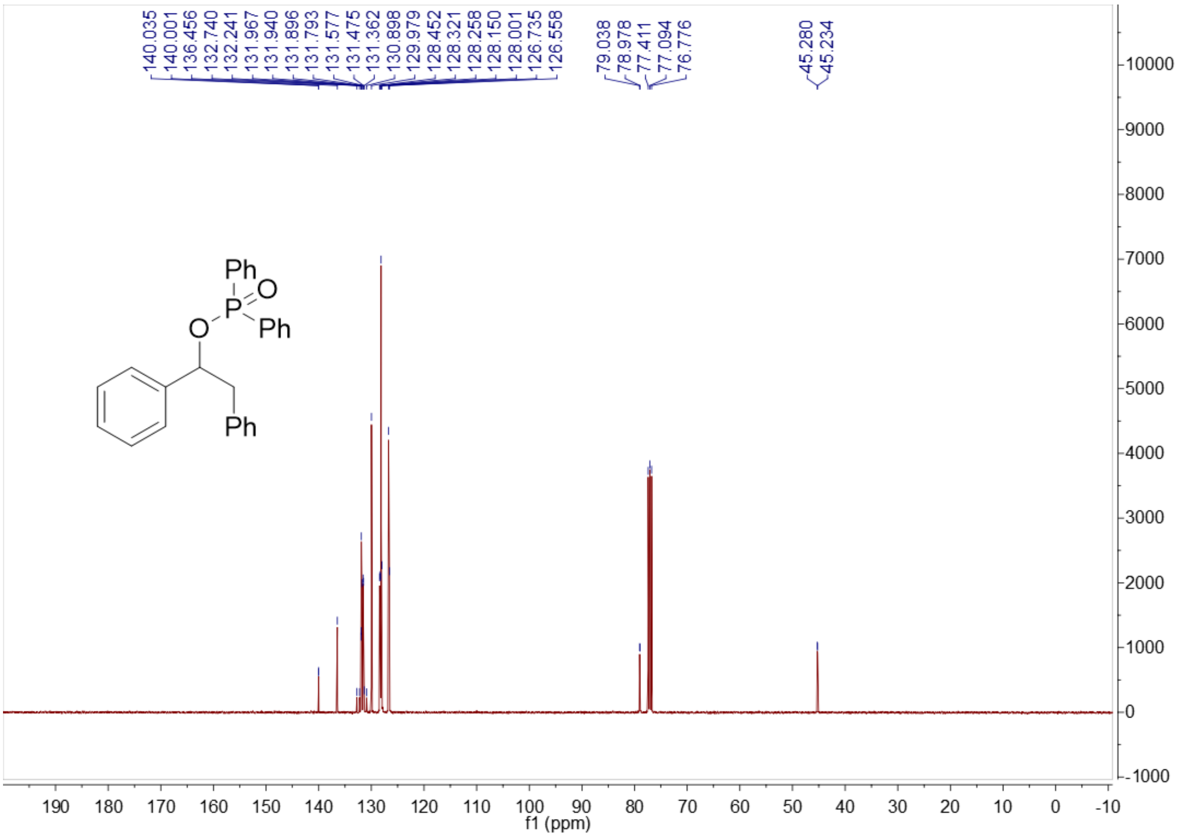

^{1}H NMR of compound **1aj** in $CDCl_3$

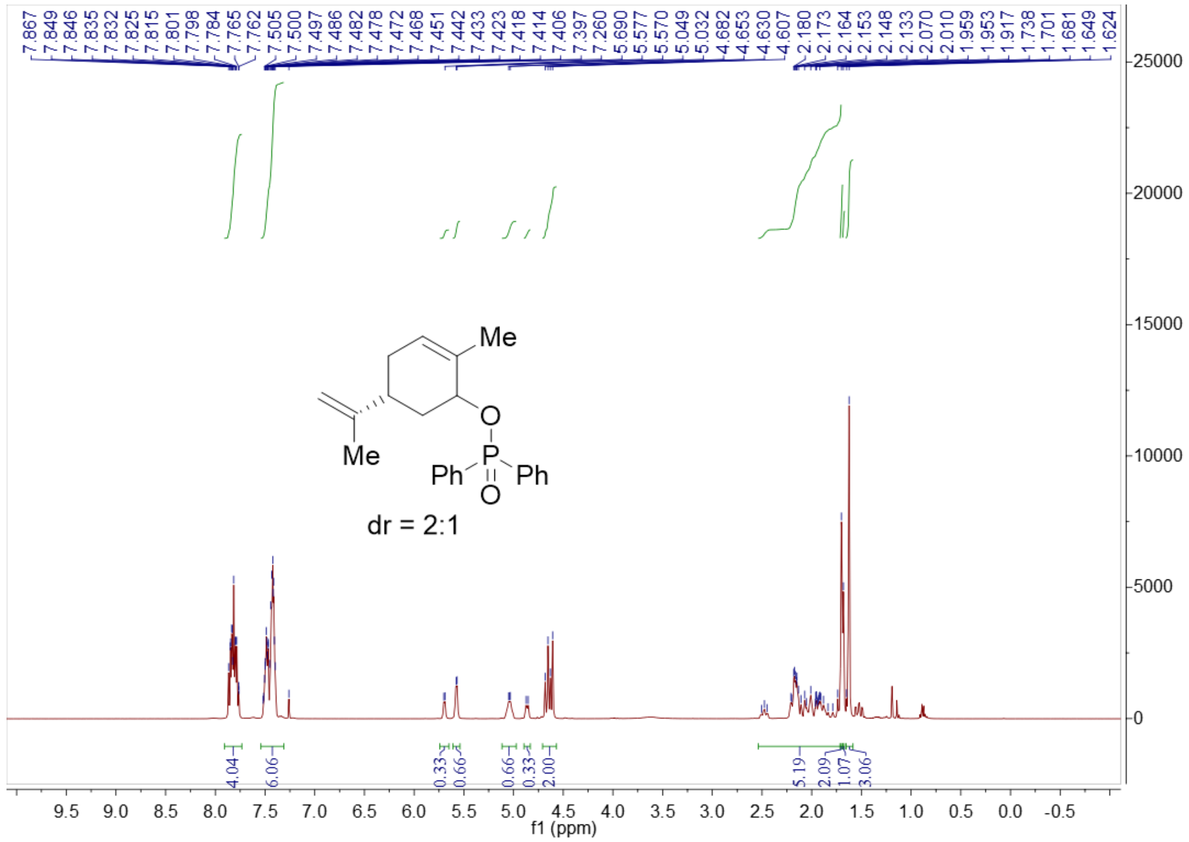

^{13}C NMR of compound **1aj** in $CDCl_3$

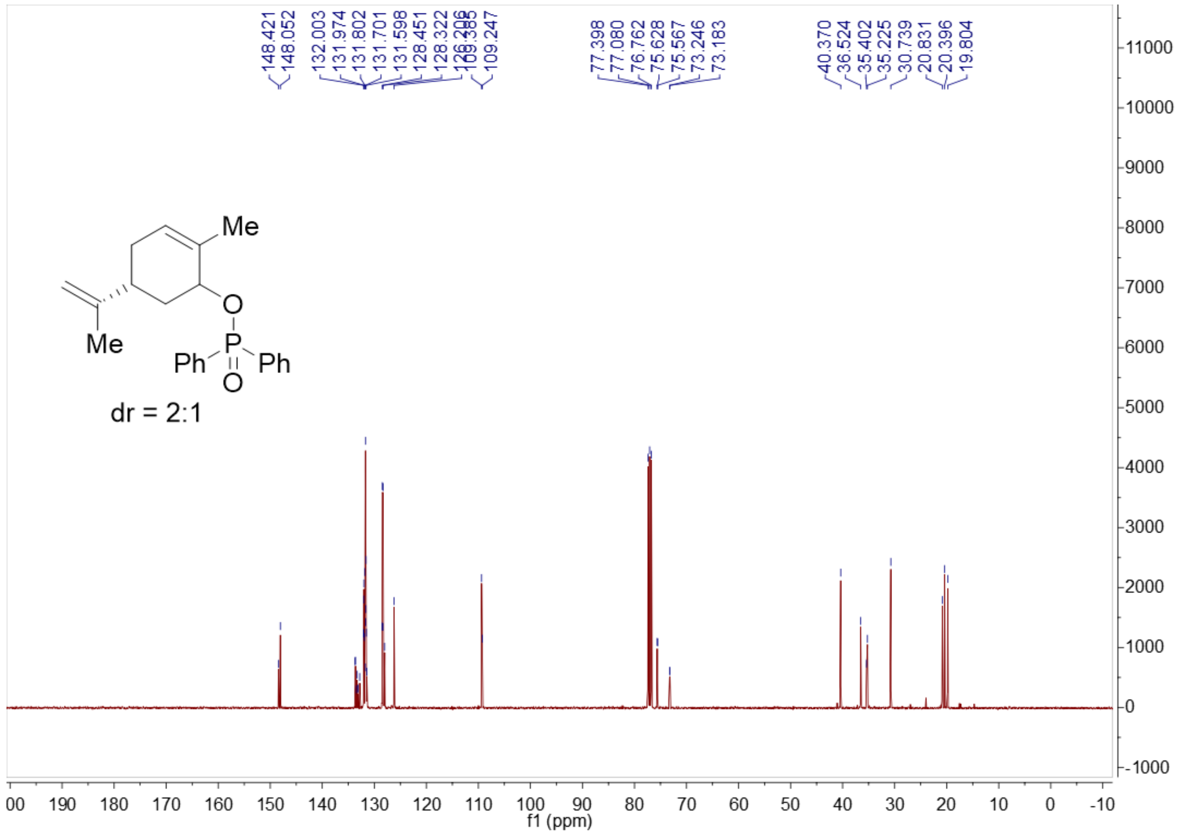

^{1}H NMR of compound **1ak** in $CDCl_3$

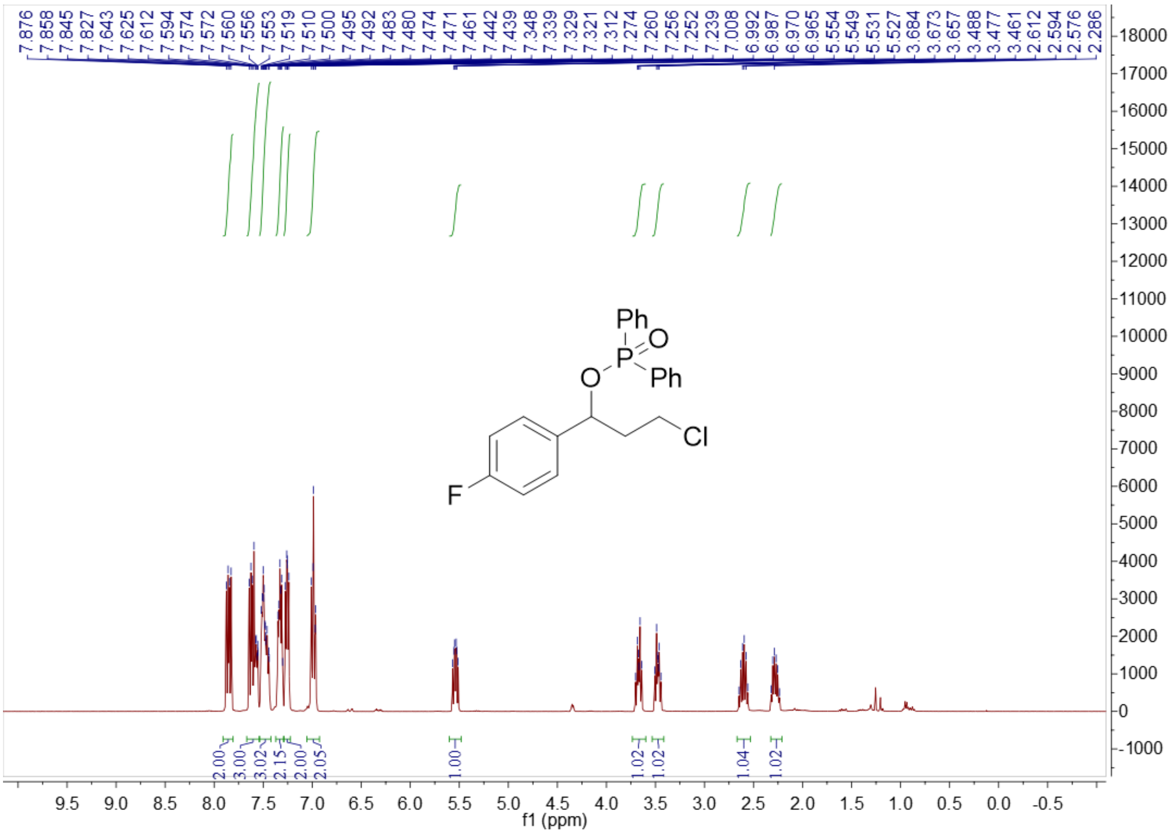

^{13}C NMR of compound **1ak** in $CDCl_3$

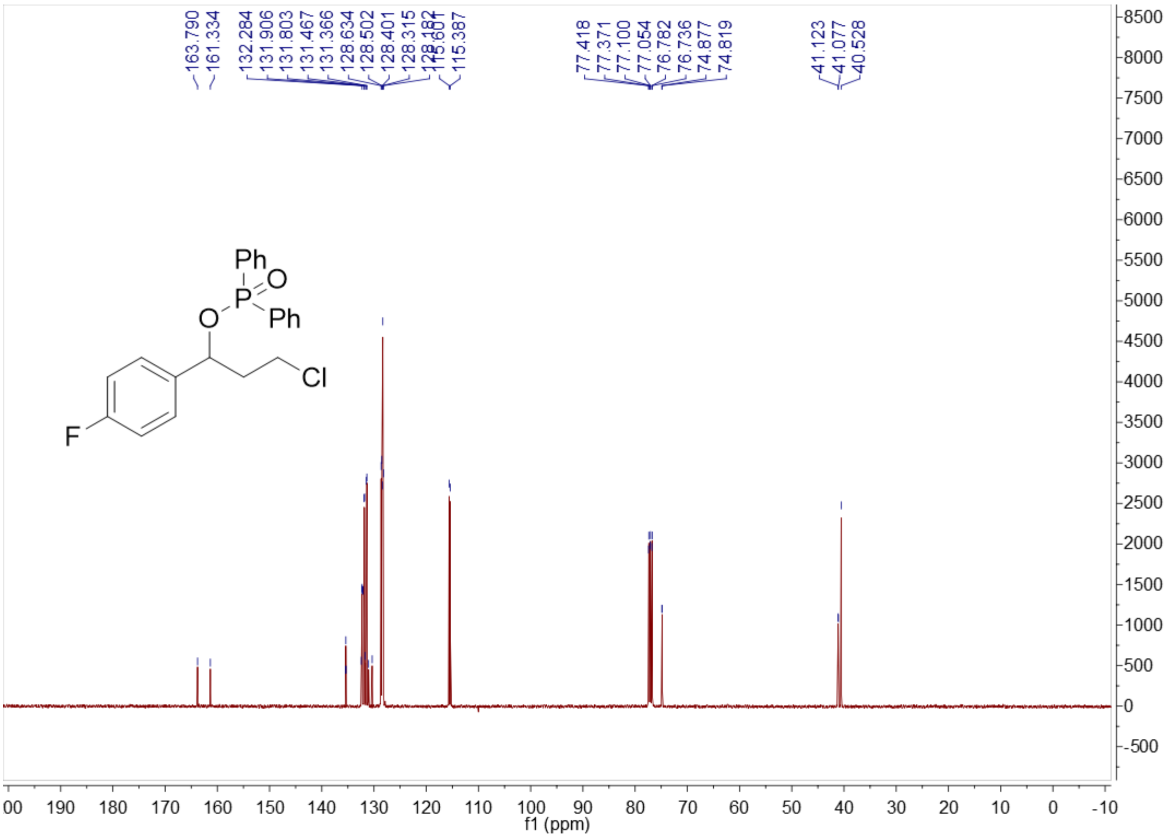

^{1}H NMR of compound **1al** in $CDCl_3$

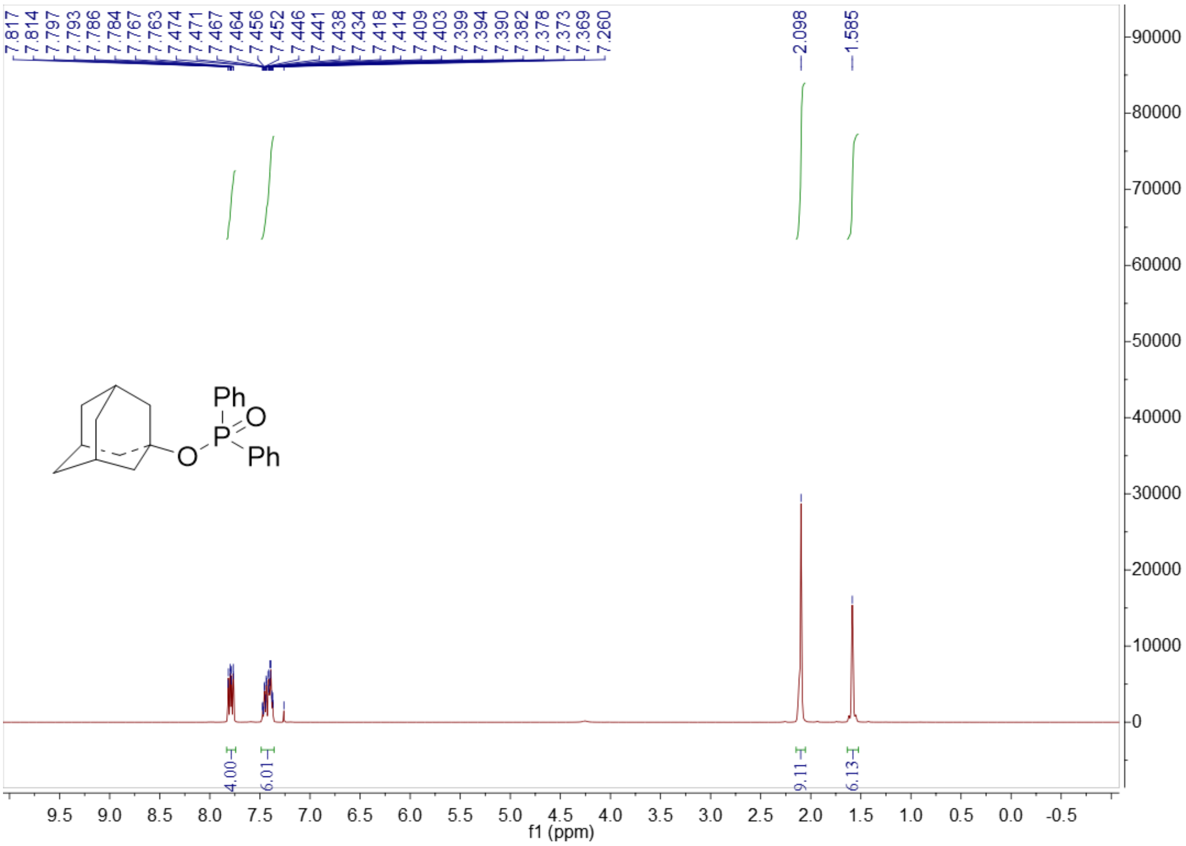

^{13}C NMR of compound **1al** in $CDCl_3$

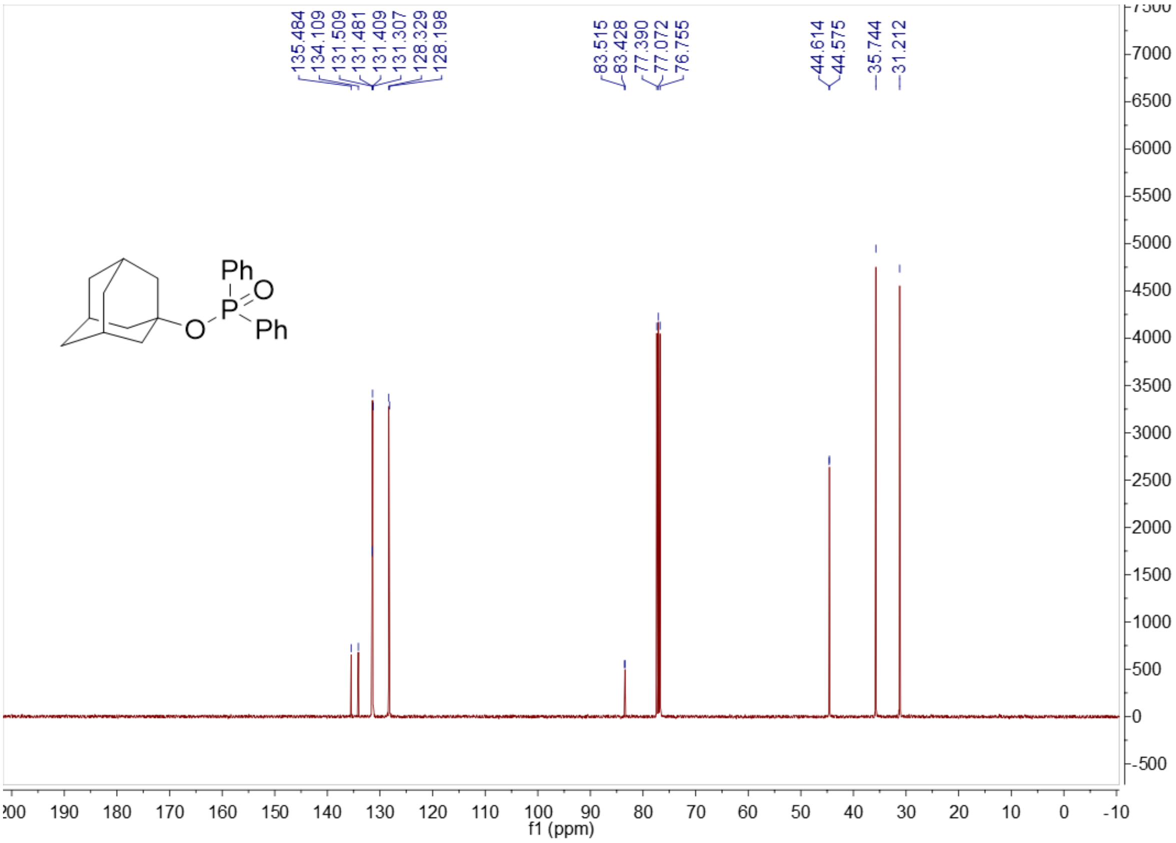

^{1}H NMR of compound **4a** in $CDCl_3$

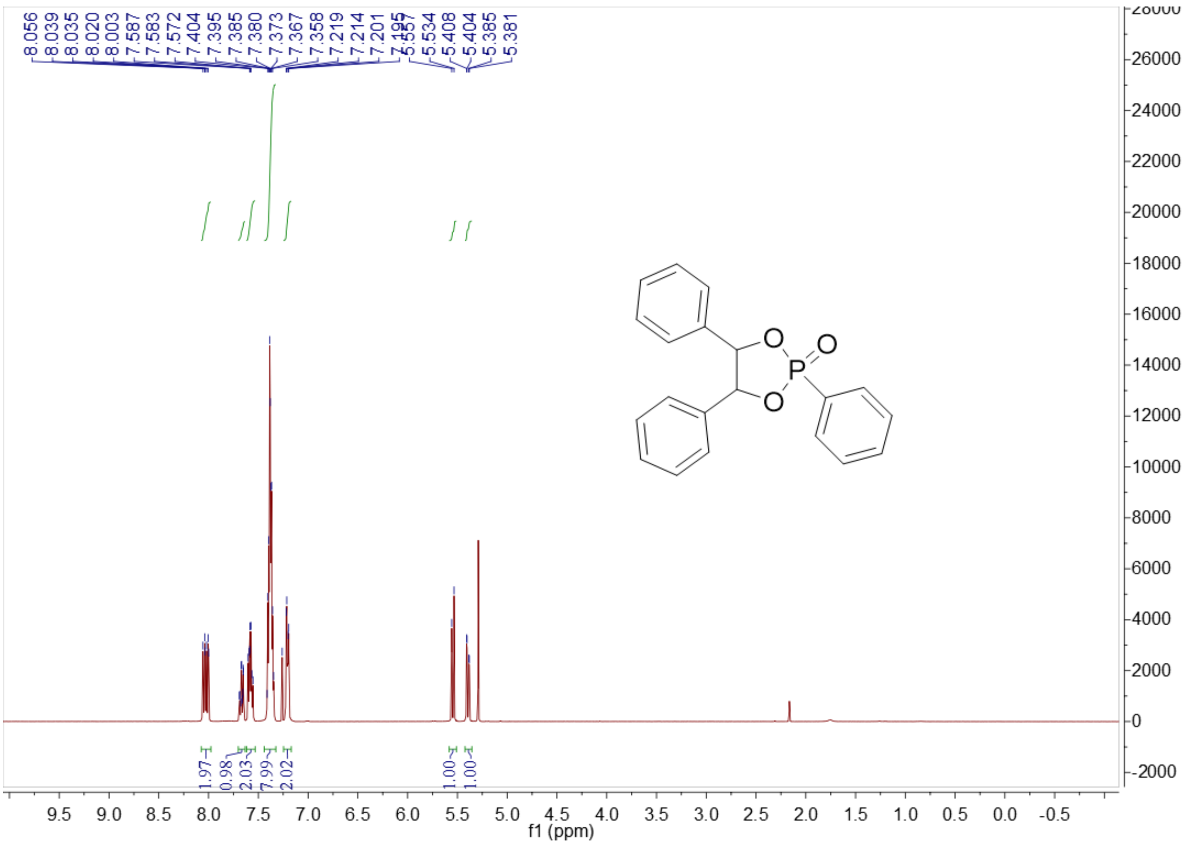

^{13}C NMR of compound **4a** in $CDCl_3$

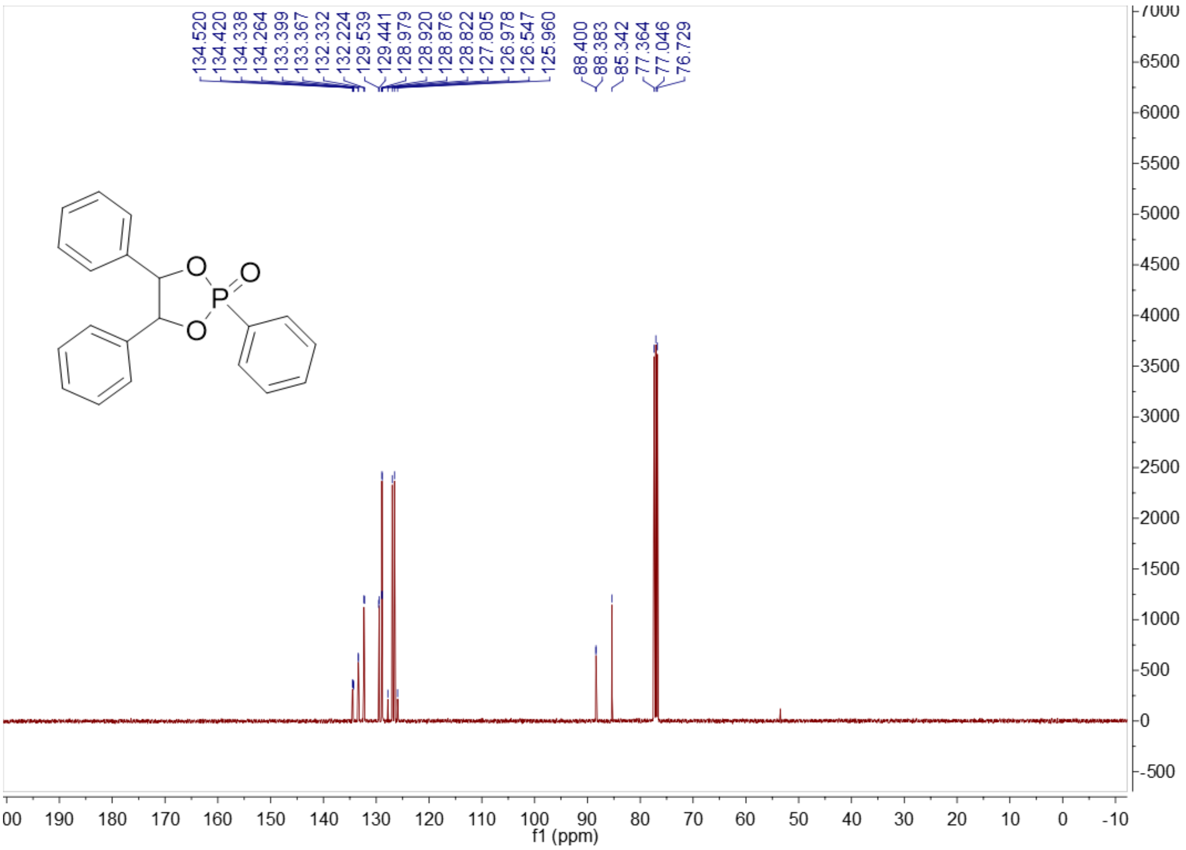

^{1}H NMR of compound ***E*-2a** in $CDCl_3$

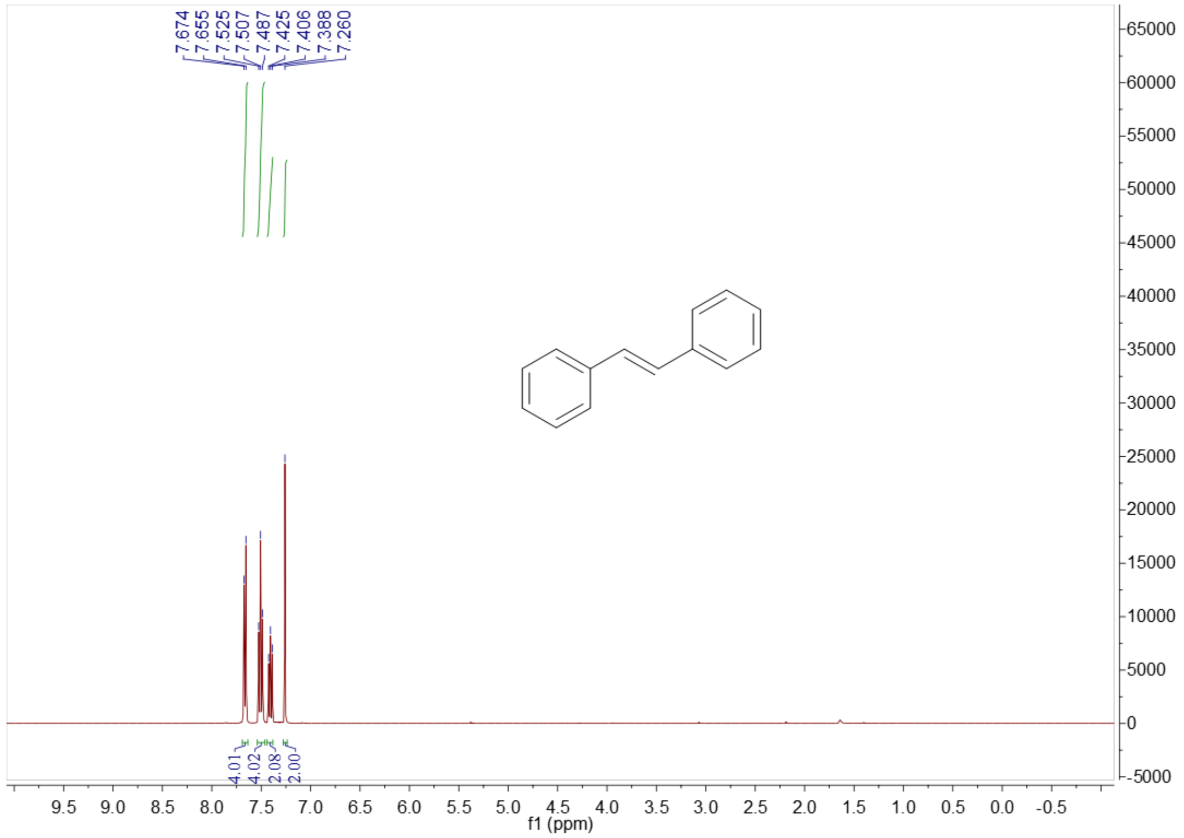

^{13}C NMR of compound ***E*-2a** in $CDCl_3$

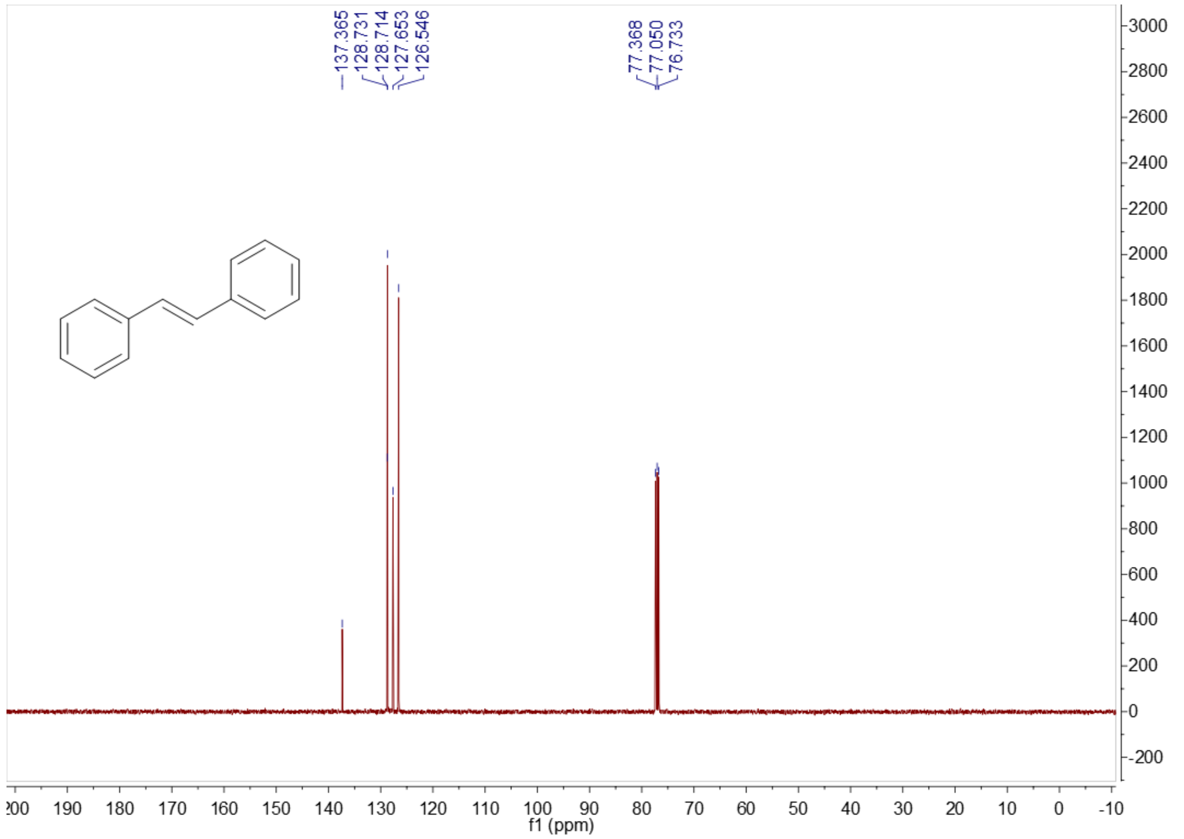

^{1}H NMR of compound ***Z*-2a** in CDCl$_3$

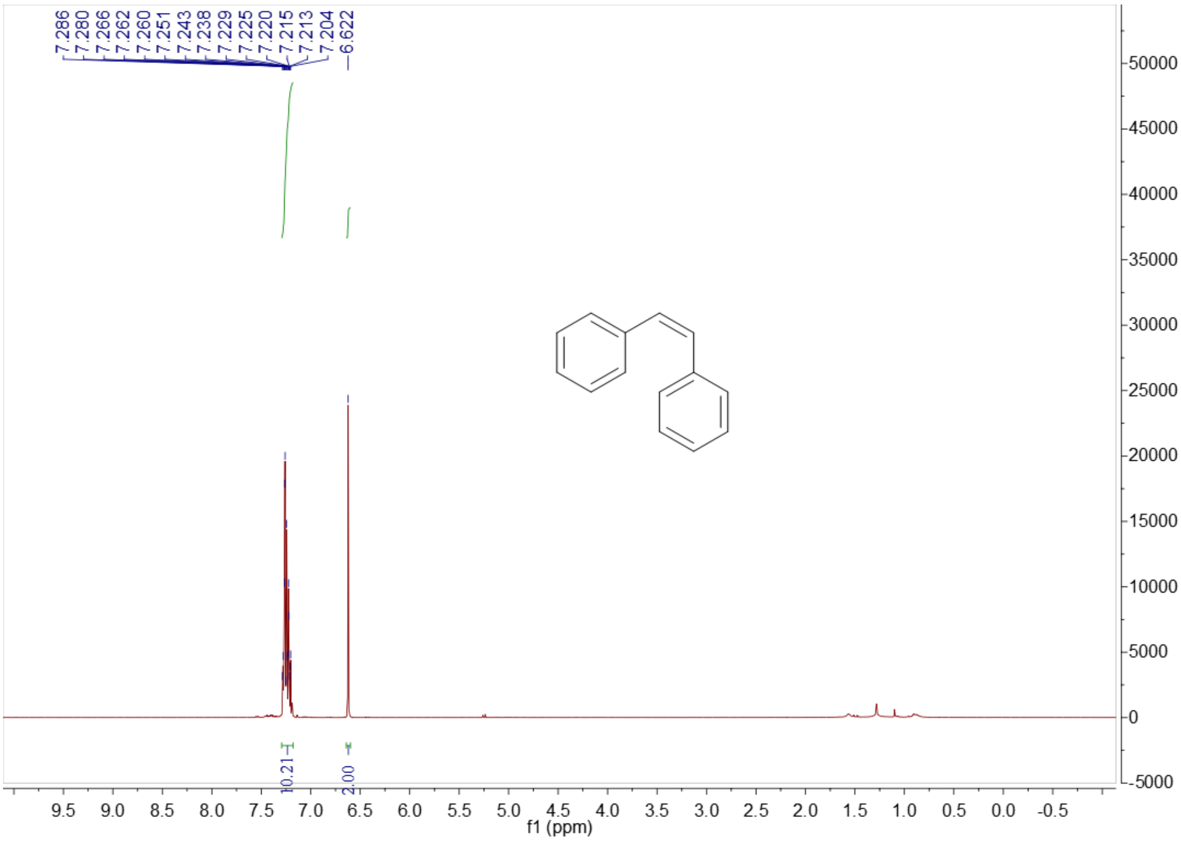

^{13}C NMR of compound ***Z*-2a** in CDCl$_3$

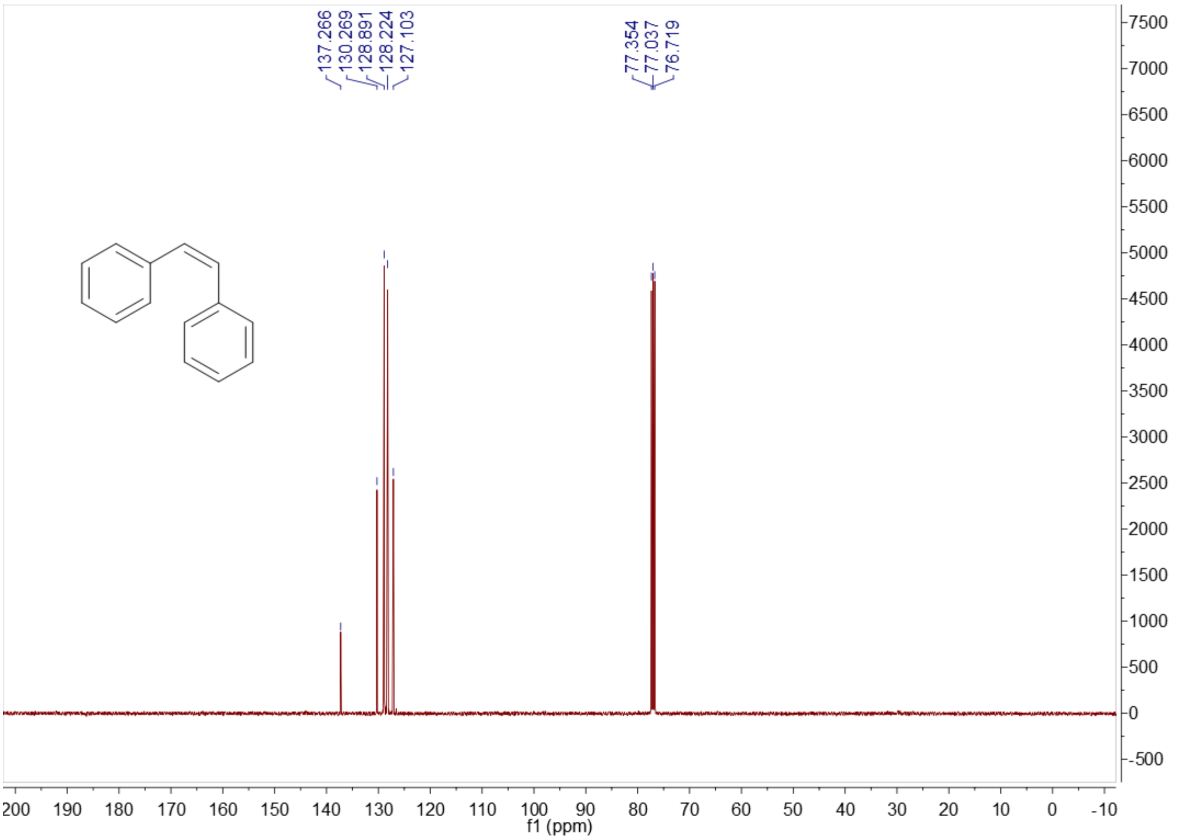

^{1}H NMR of compound **2b** in $CDCl_3$

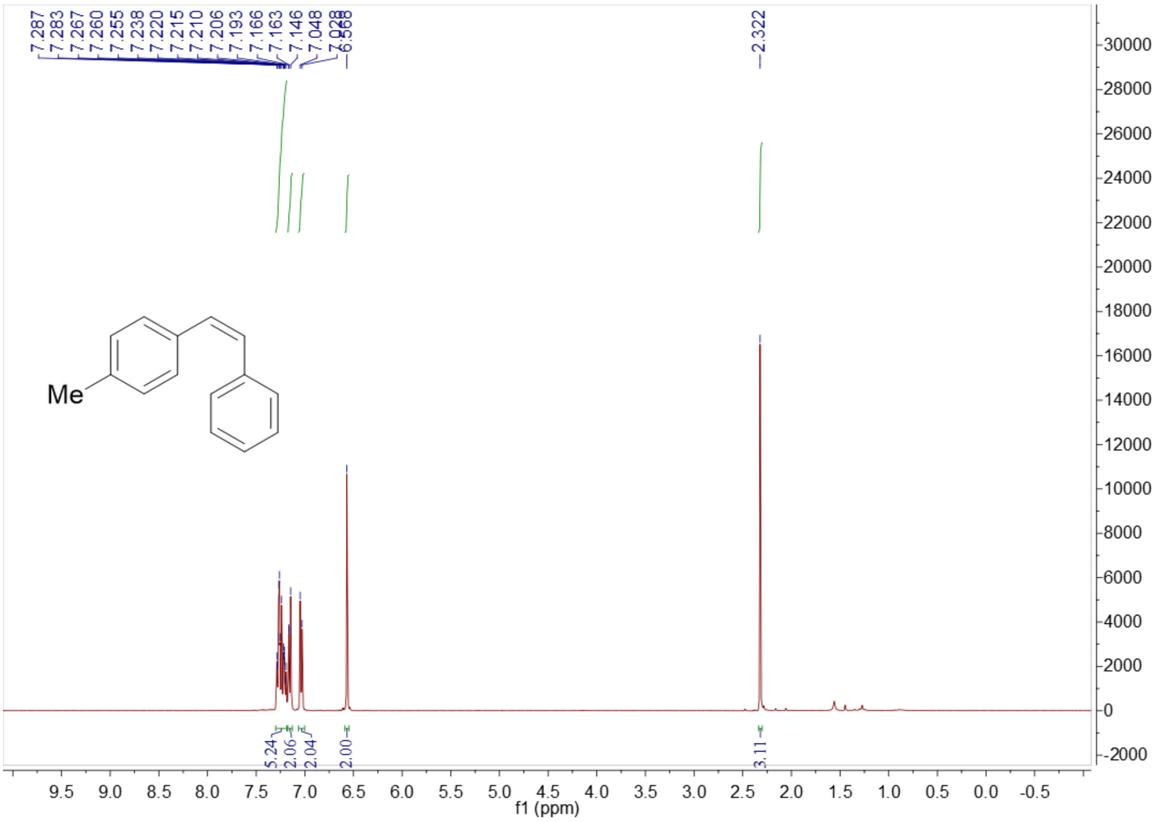

^{13}C NMR of compound **2b** in $CDCl_3$

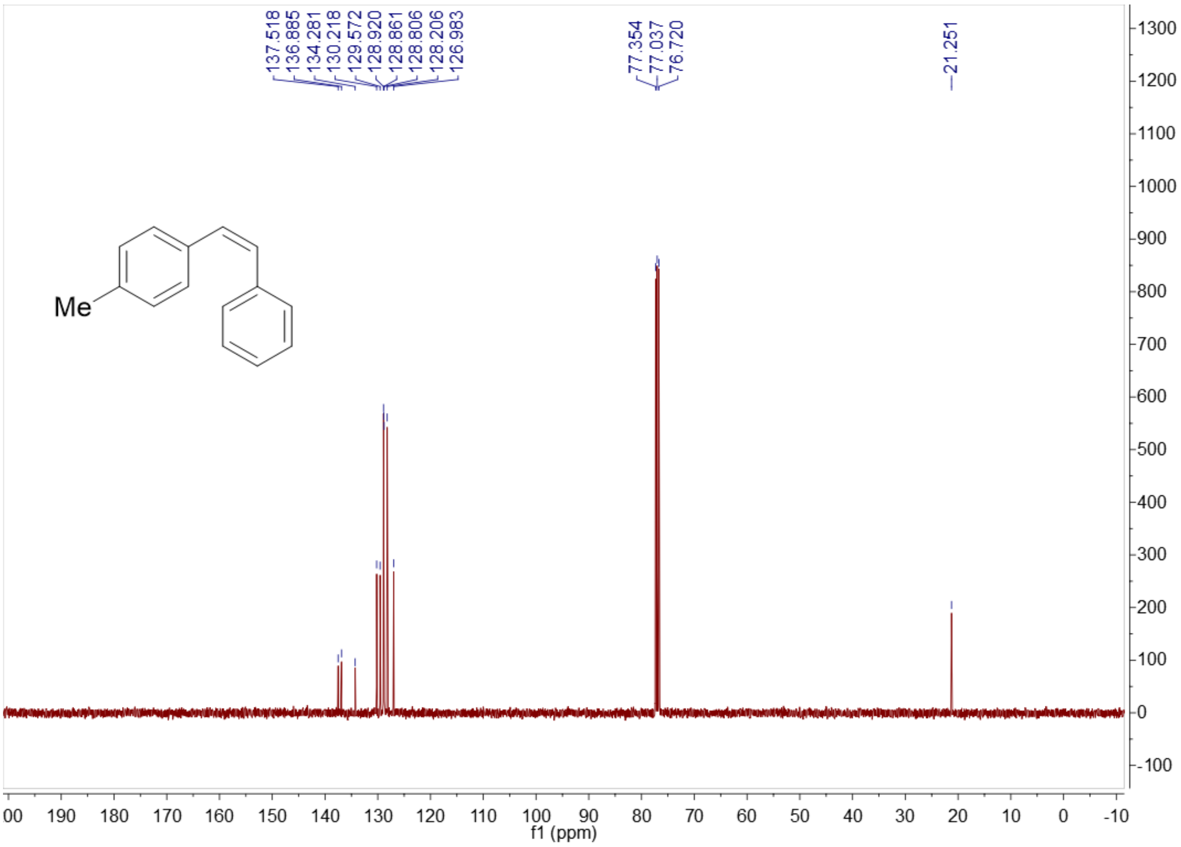

^{1}H NMR of compound **2c** in $CDCl_3$

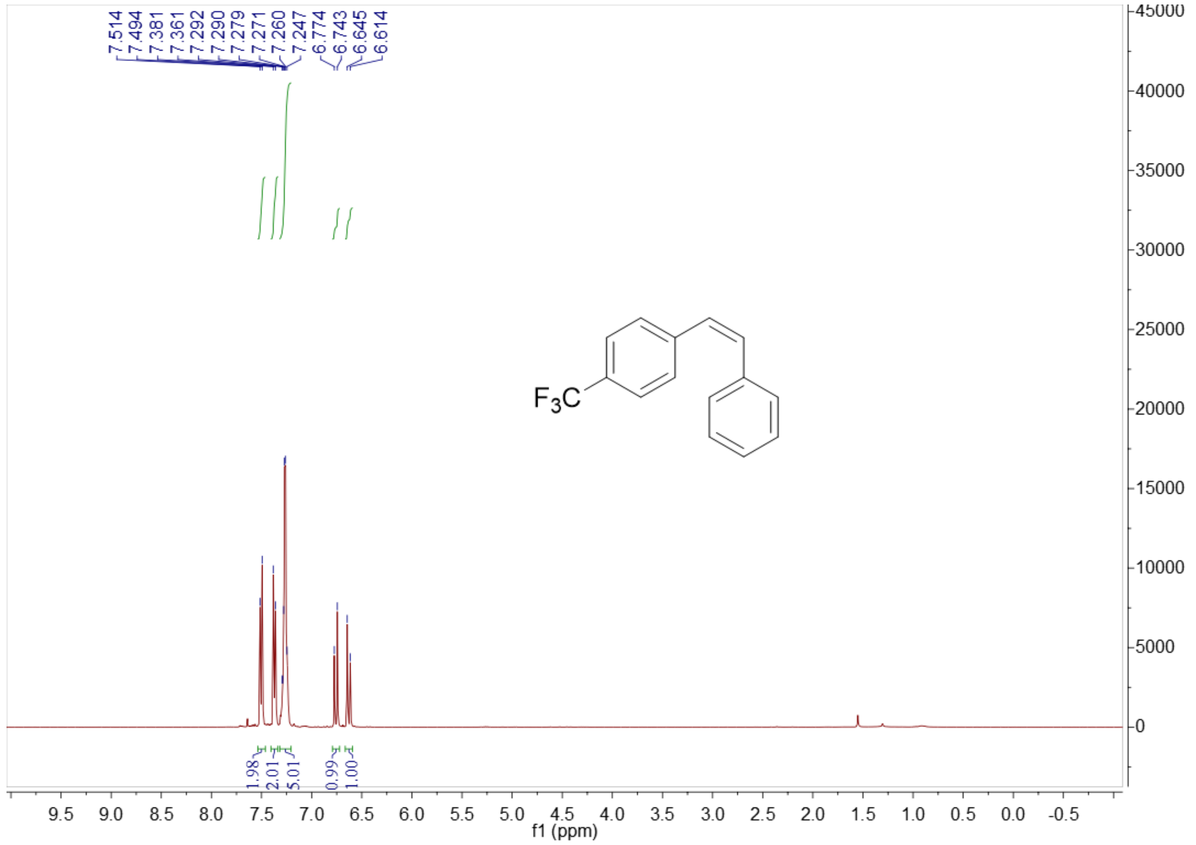

^{13}C NMR of compound **2c** in $CDCl_3$

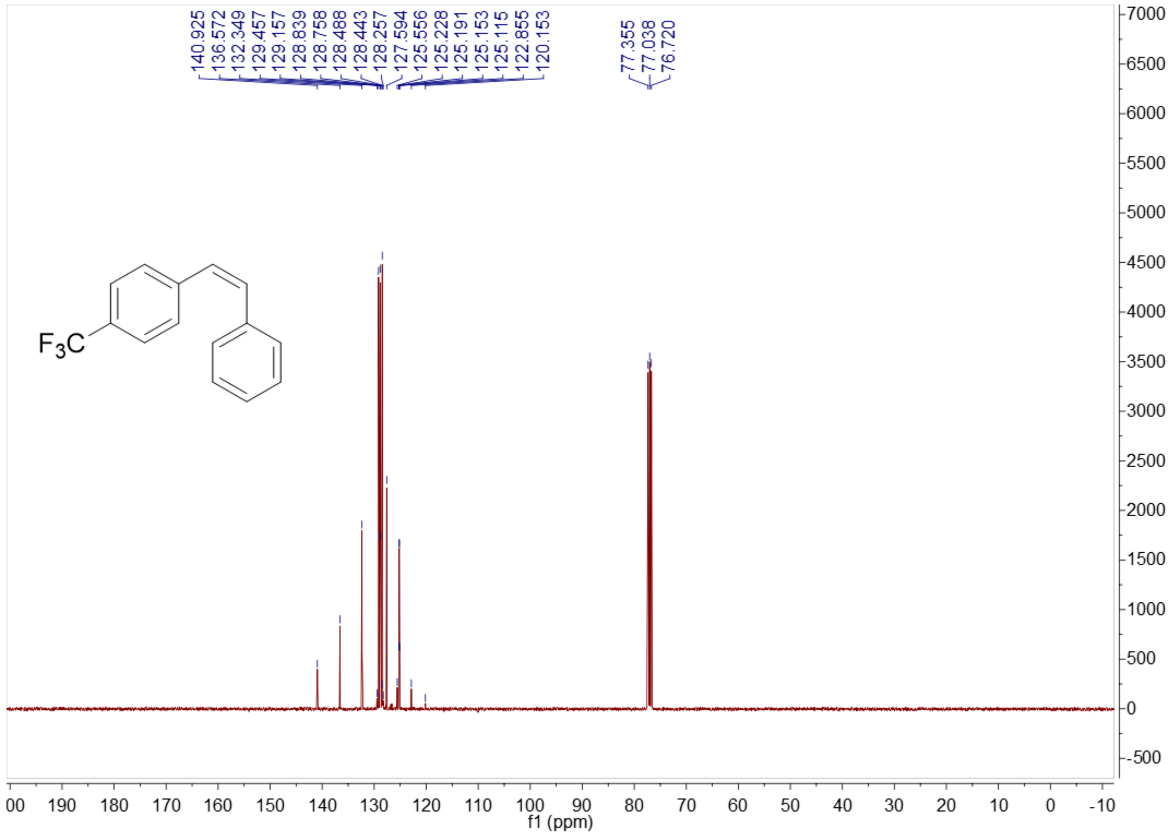

^{1}H NMR of compound **2d** in $CDCl_3$

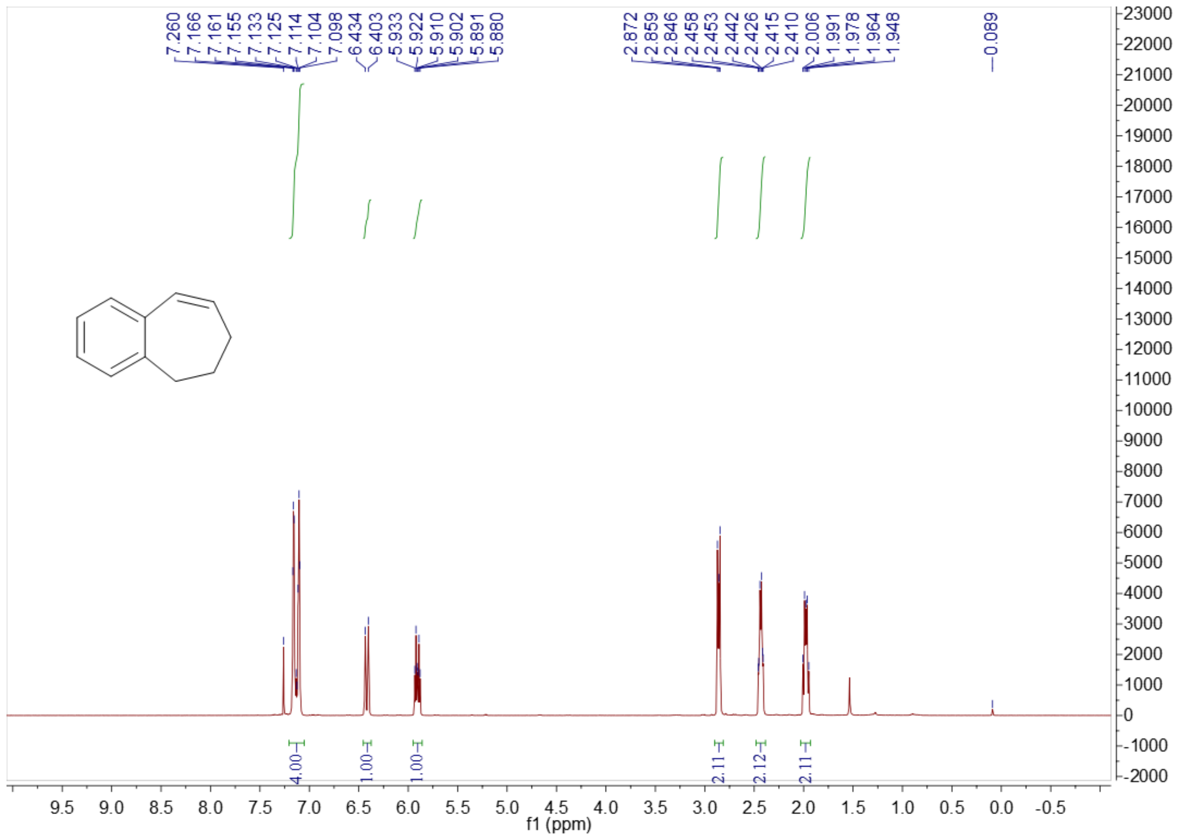

^{13}C NMR of compound **2d** in $CDCl_3$

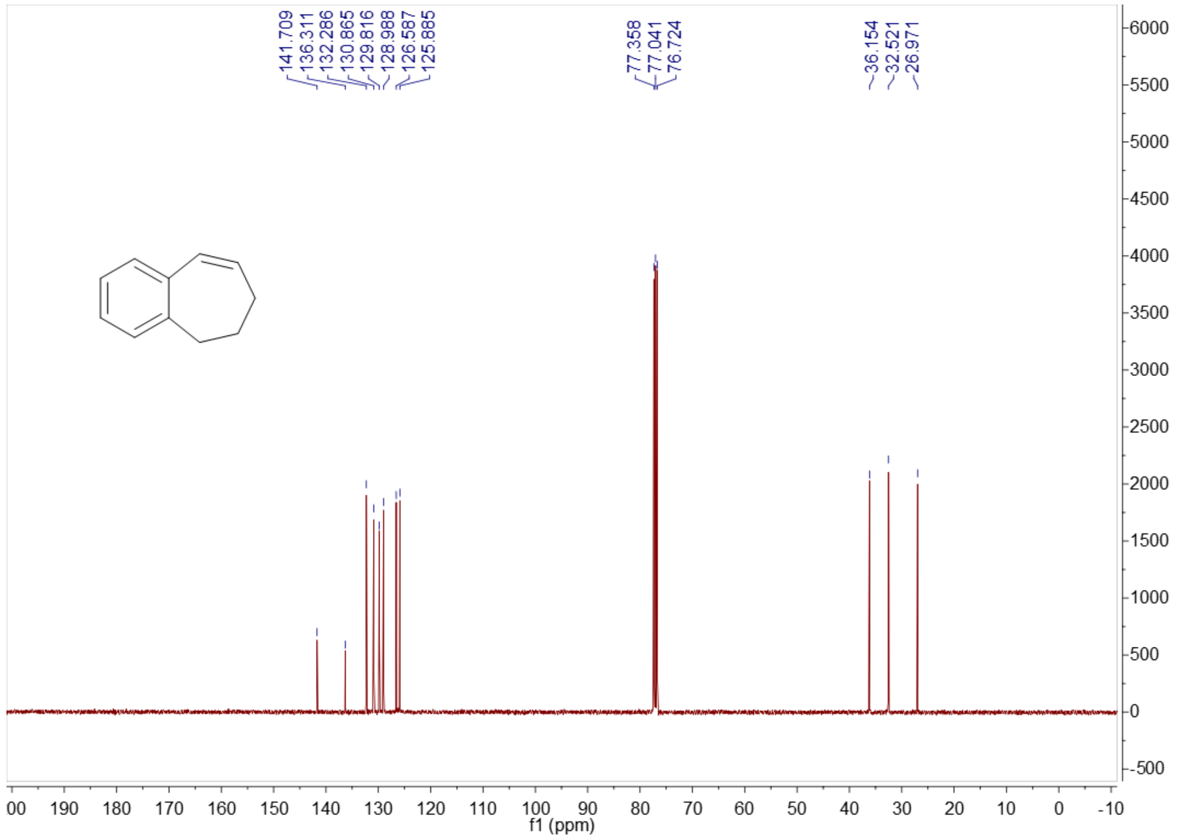

^{1}H NMR of compound **2e** in $CDCl_3$

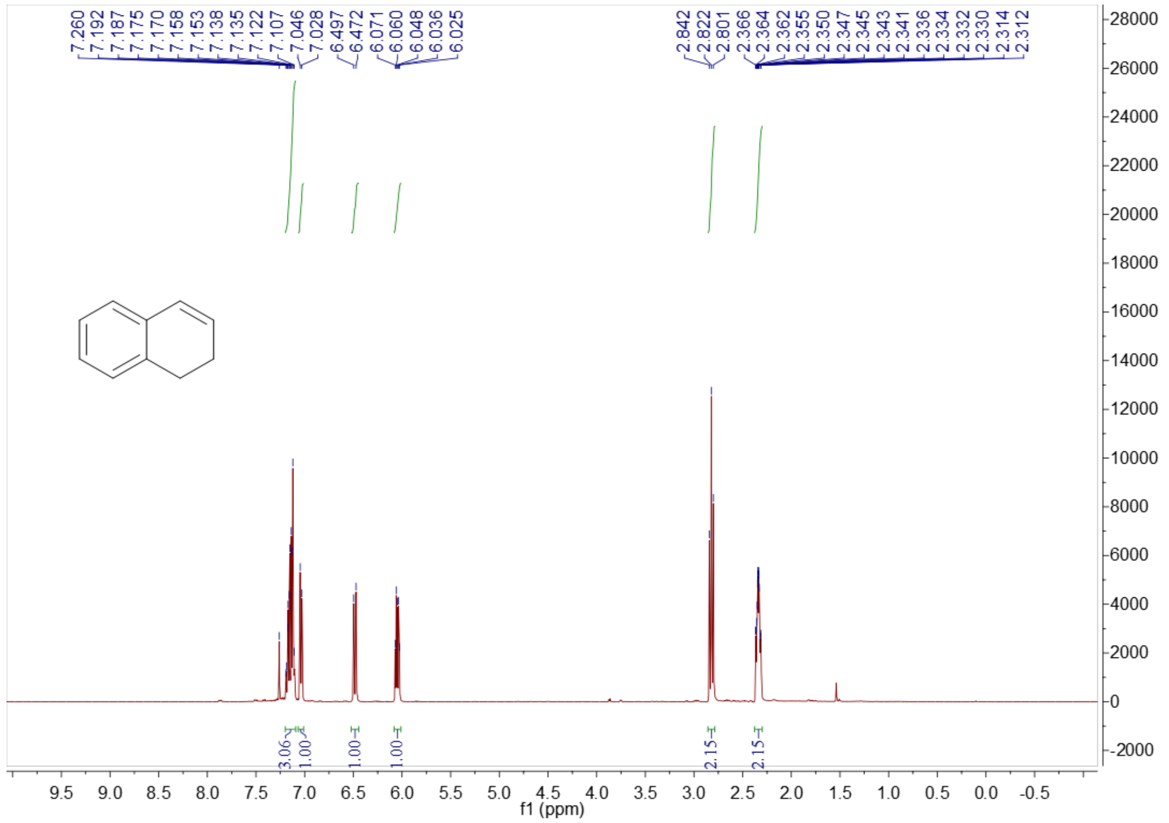

^{13}C NMR of compound **2e** in $CDCl_3$

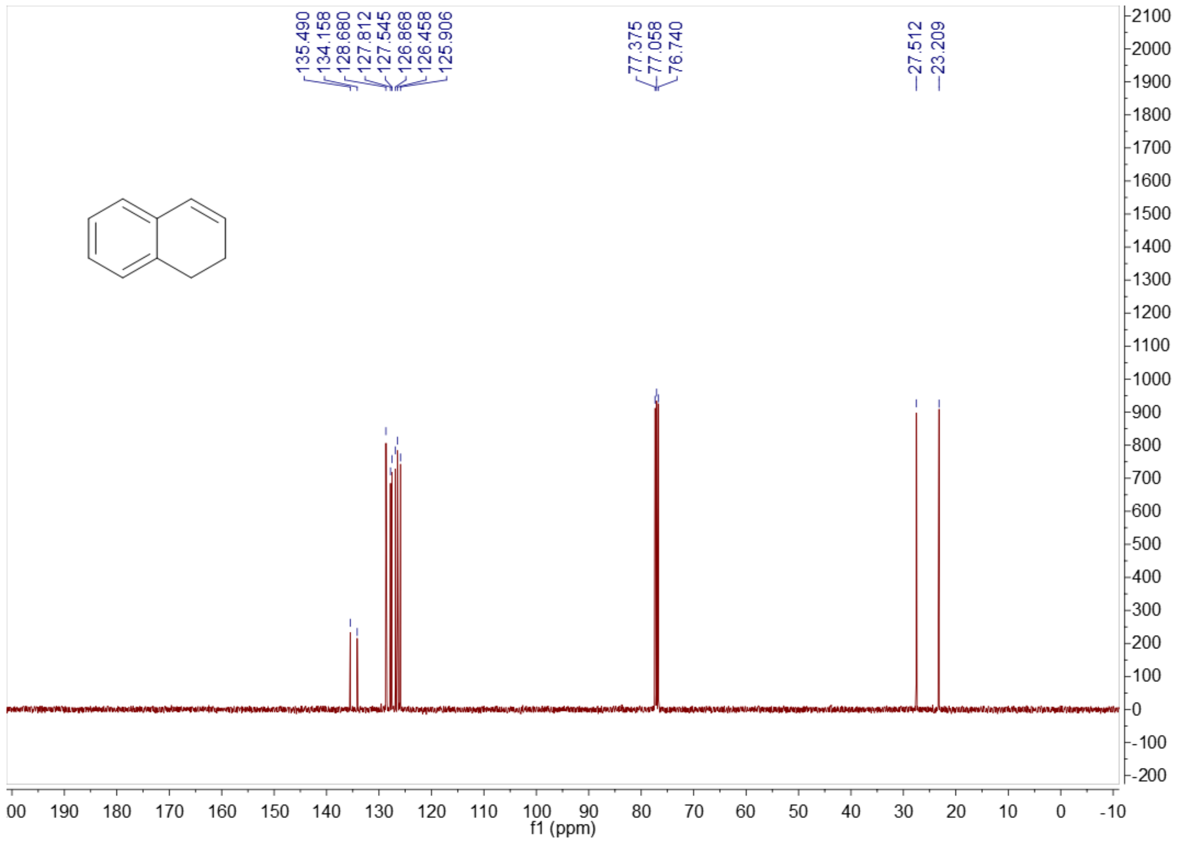

^{1}H NMR of compound **2f** in $CDCl_3$

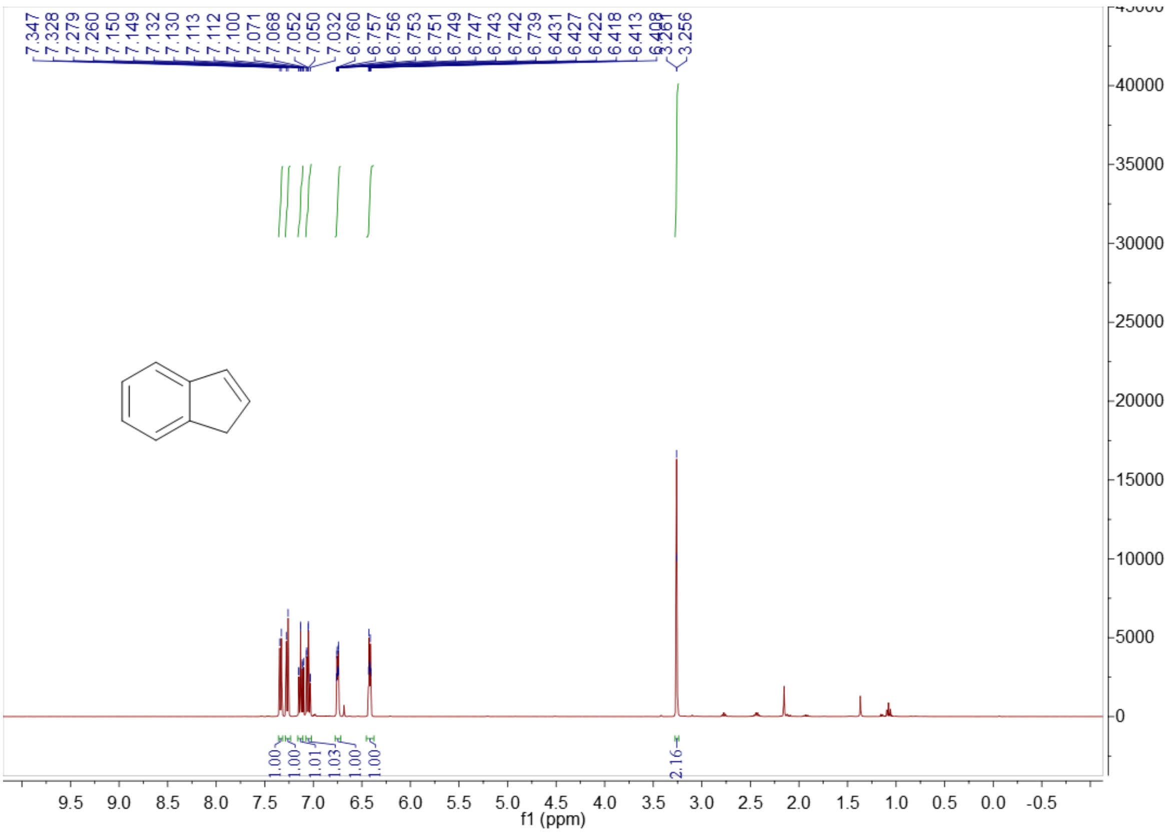

^{13}C NMR of compound **2f** in $CDCl_3$

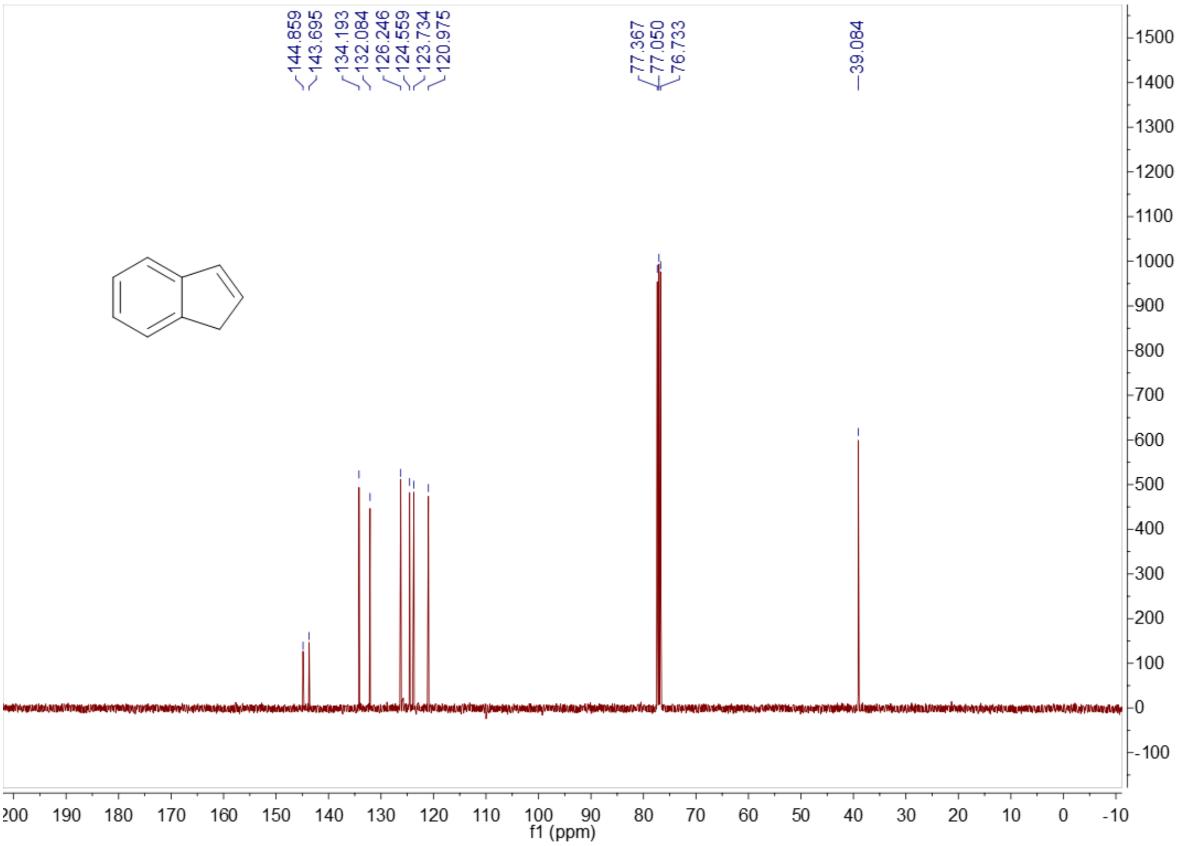

^{1}H NMR of compound **2g** in $CDCl_3$

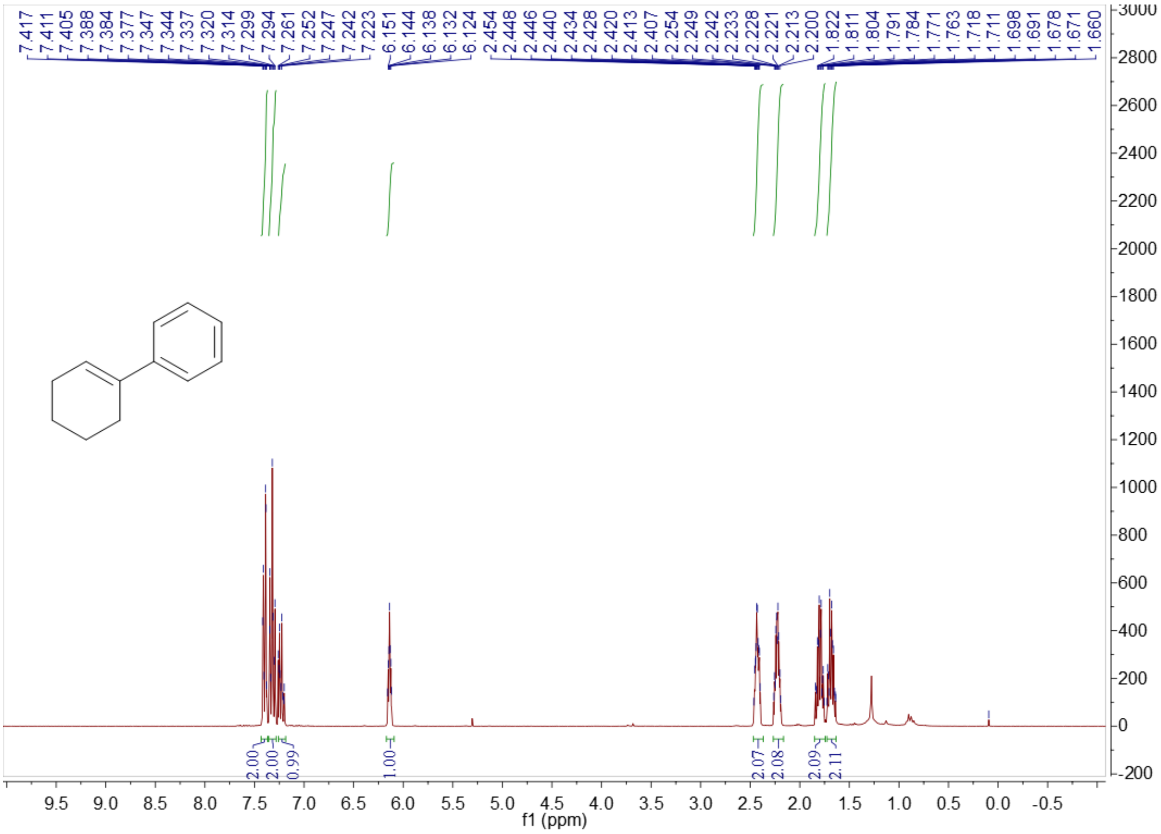

^{13}C NMR of compound **2g** in $CDCl_3$

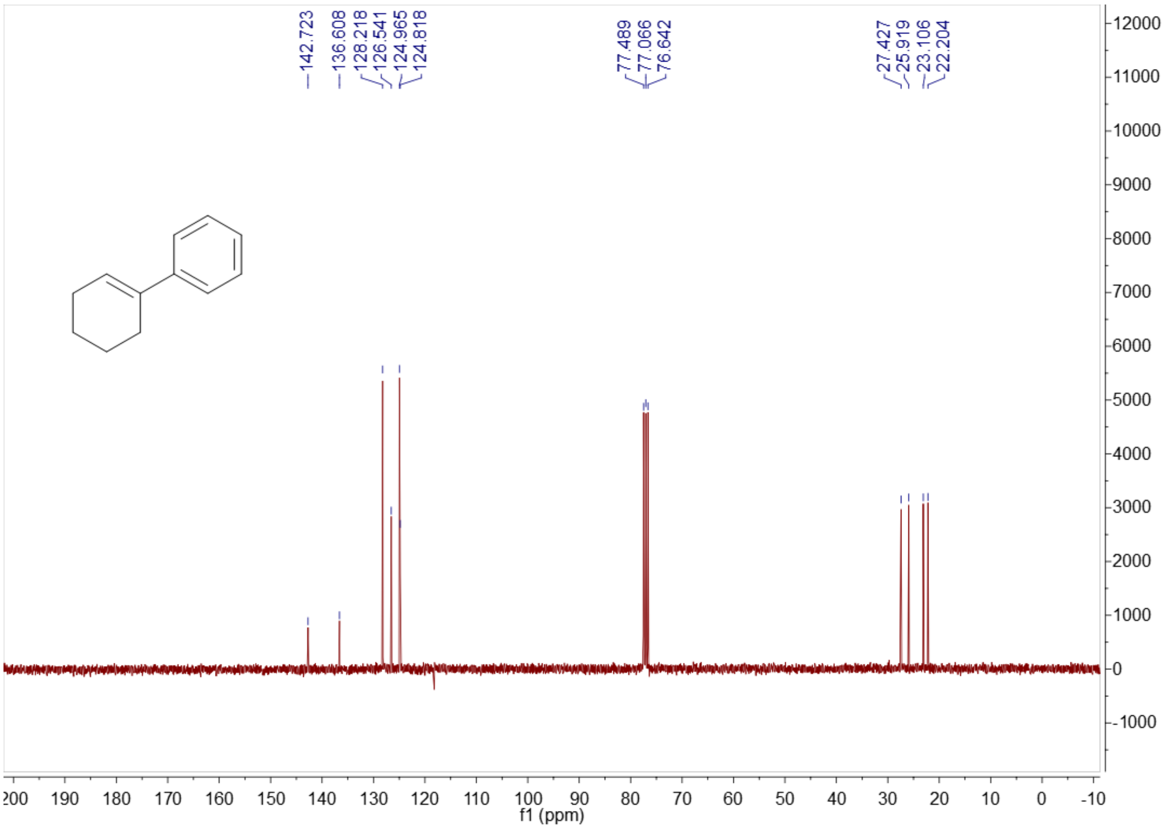

^{1}H NMR of compound **2h** in $CDCl_3$

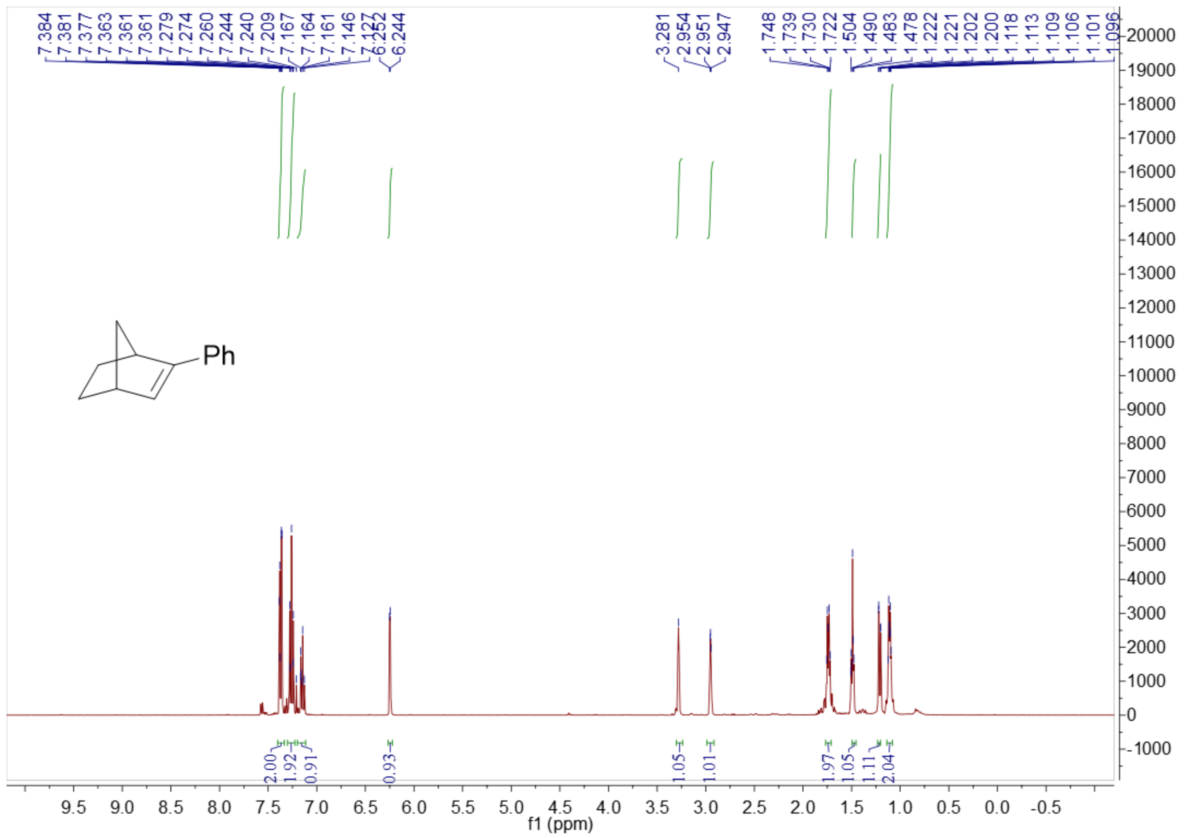

^{13}C NMR of compound **2h** in $CDCl_3$

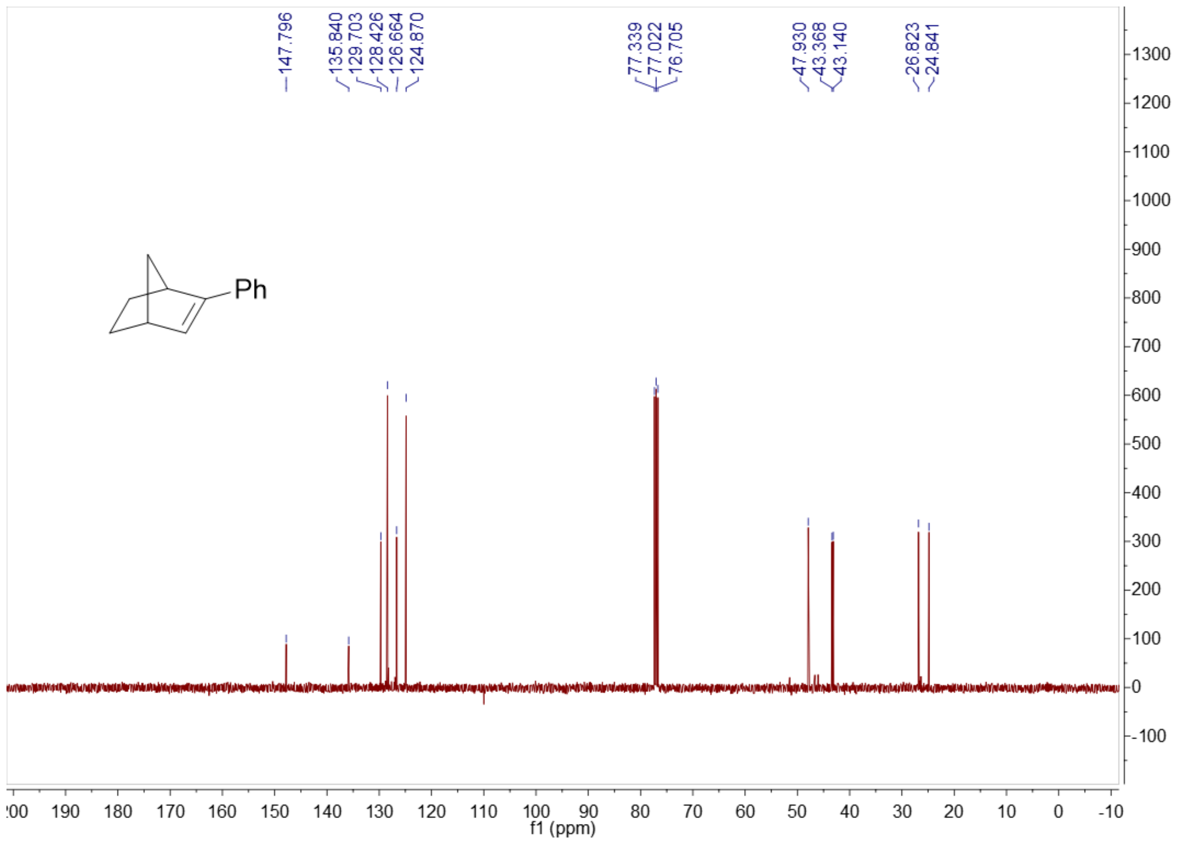

^{1}H NMR of compound **2i** in $CDCl_3$

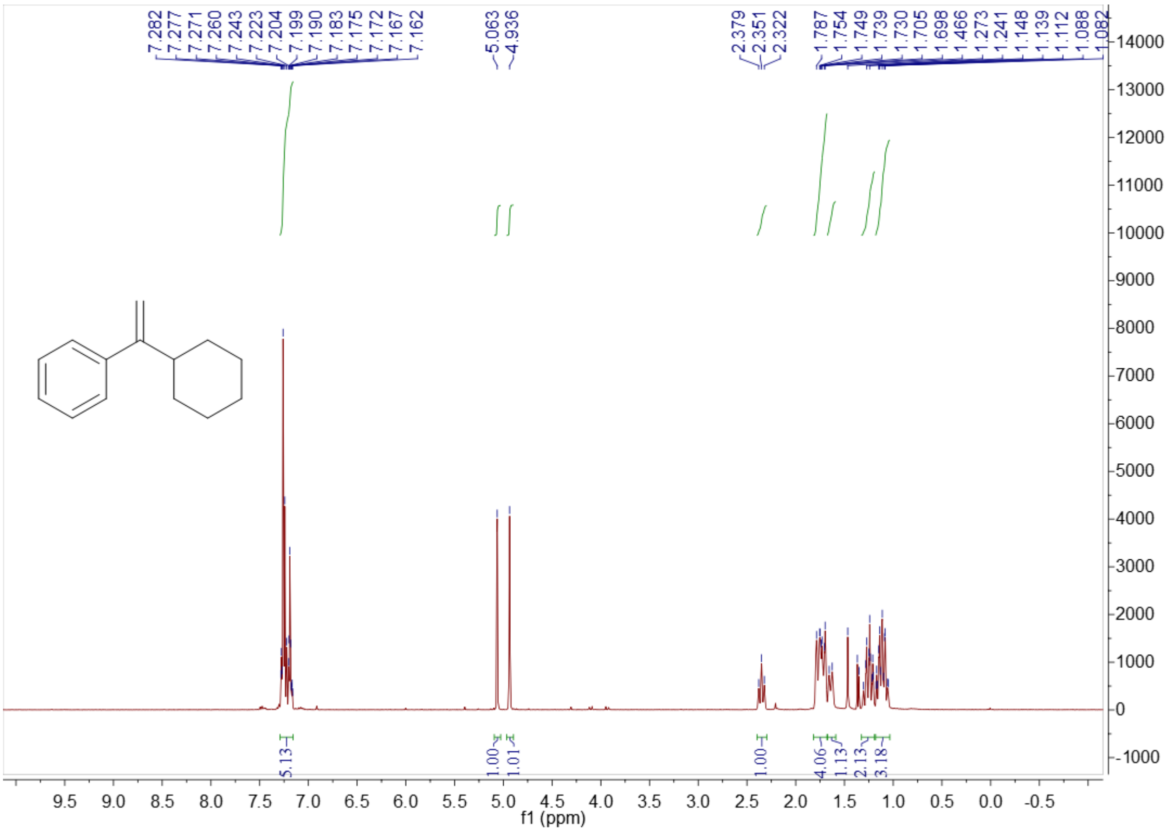

^{13}C NMR of compound **2i** in $CDCl_3$

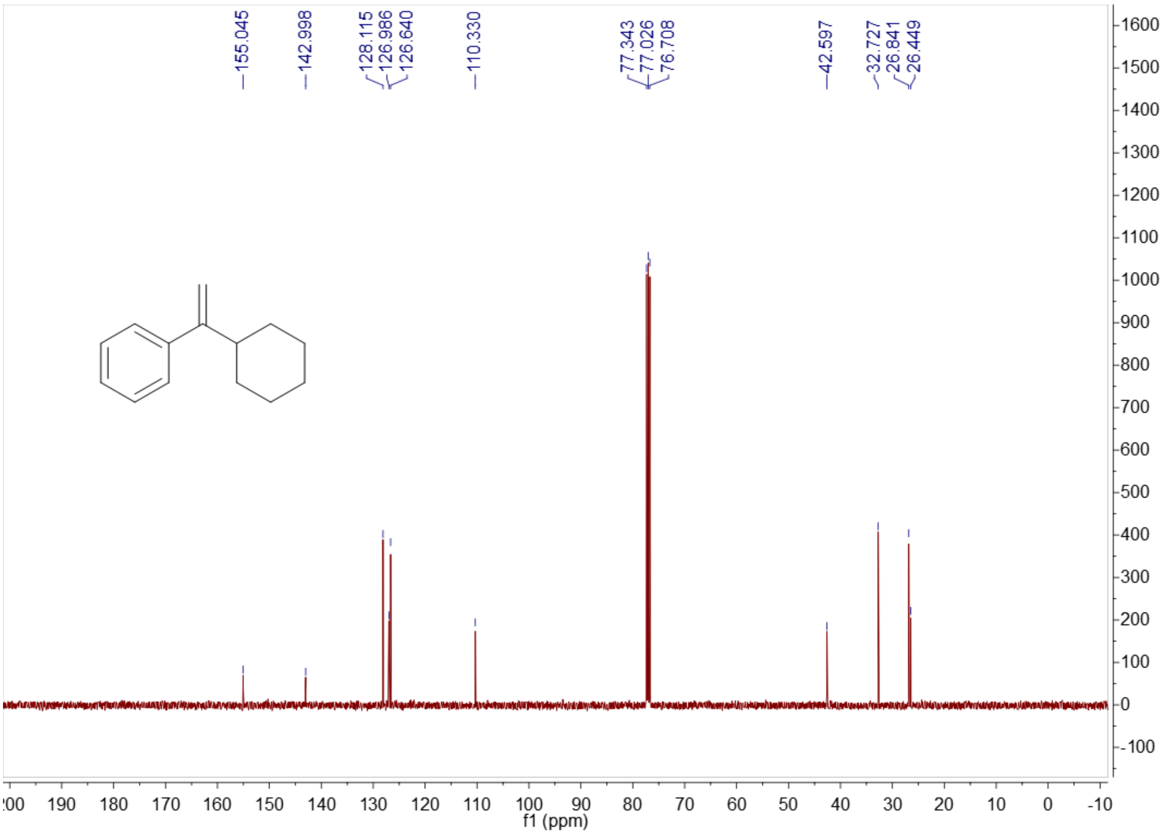

^{1}H NMR of compound **2j** in $CDCl_3$

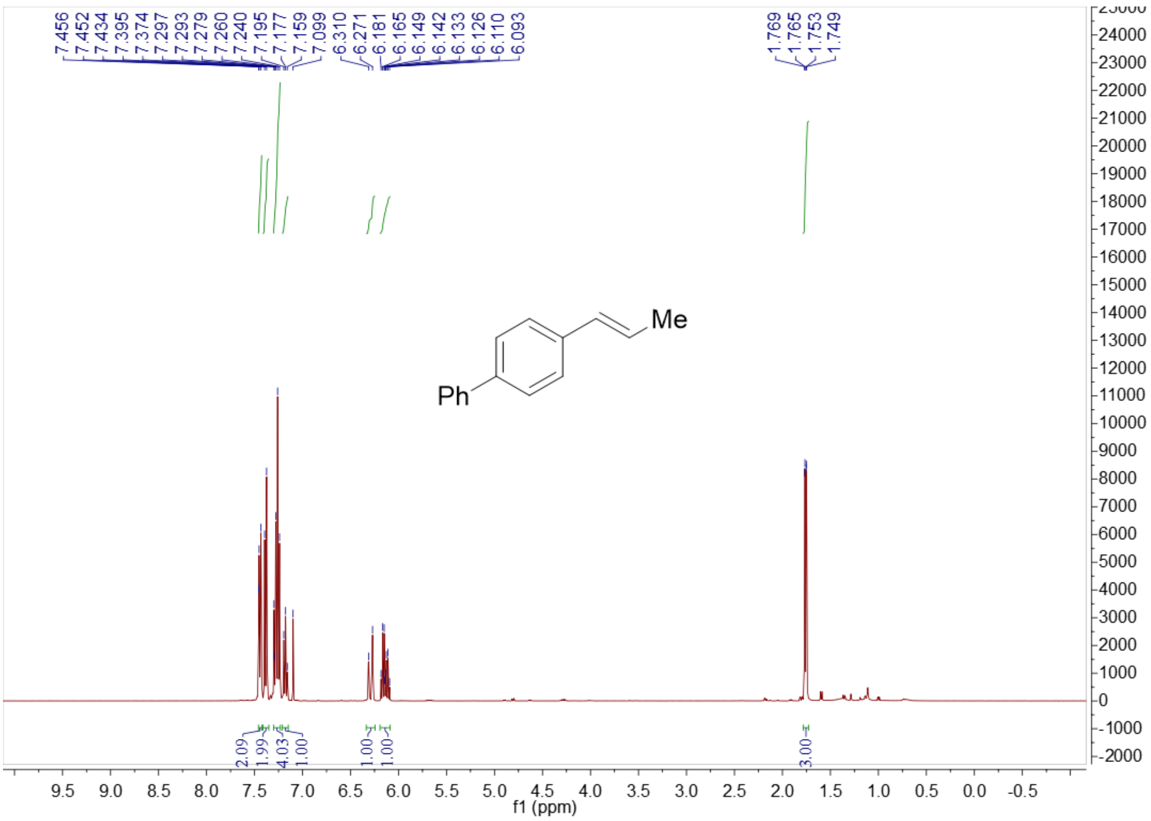

^{13}C NMR of compound **2j** in $CDCl_3$

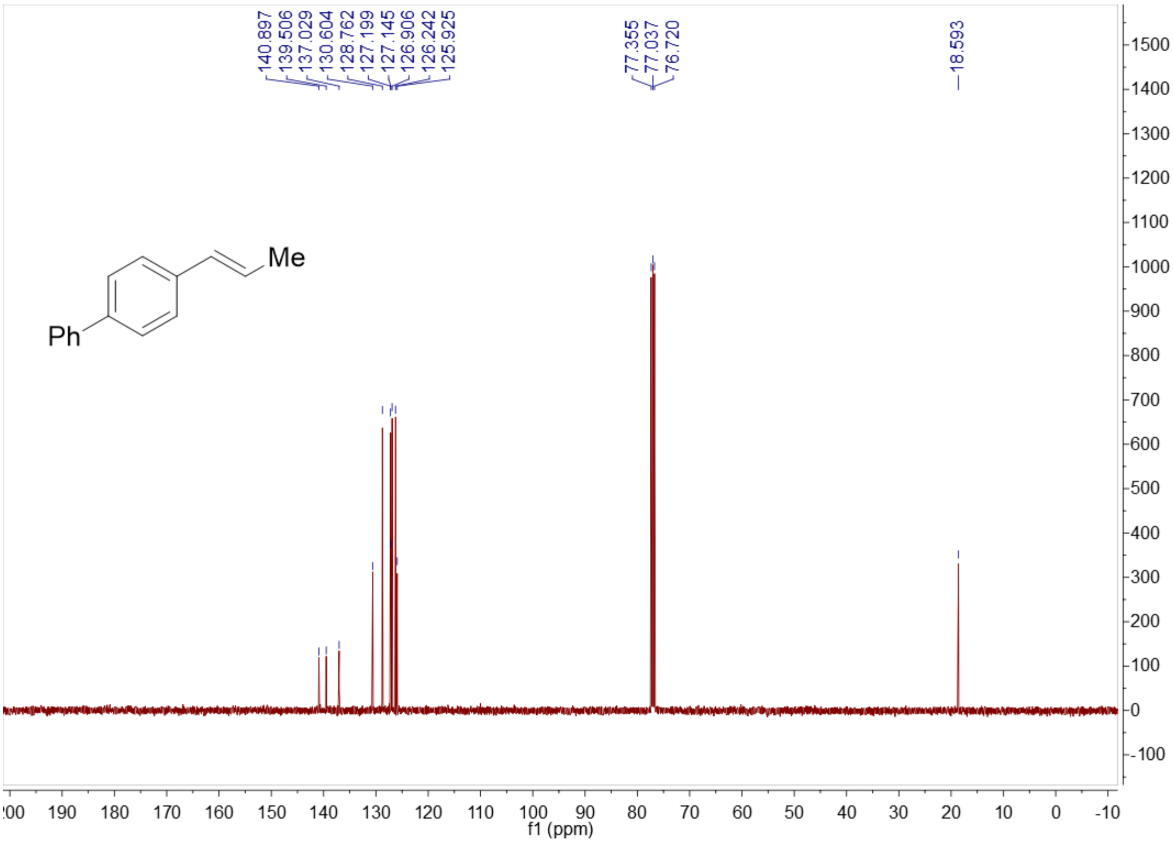

^{1}H NMR of compound **2k** in CDCl$_3$

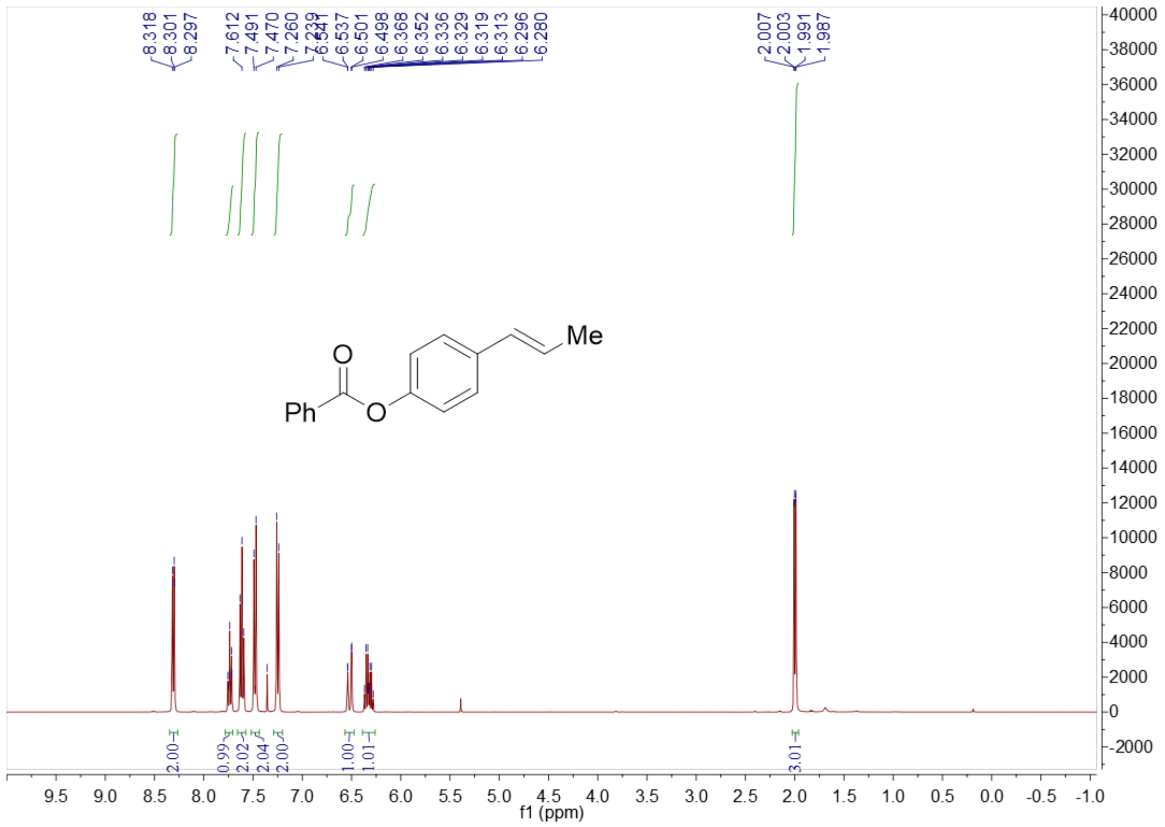

^{13}C NMR of compound **2k** in CDCl$_3$

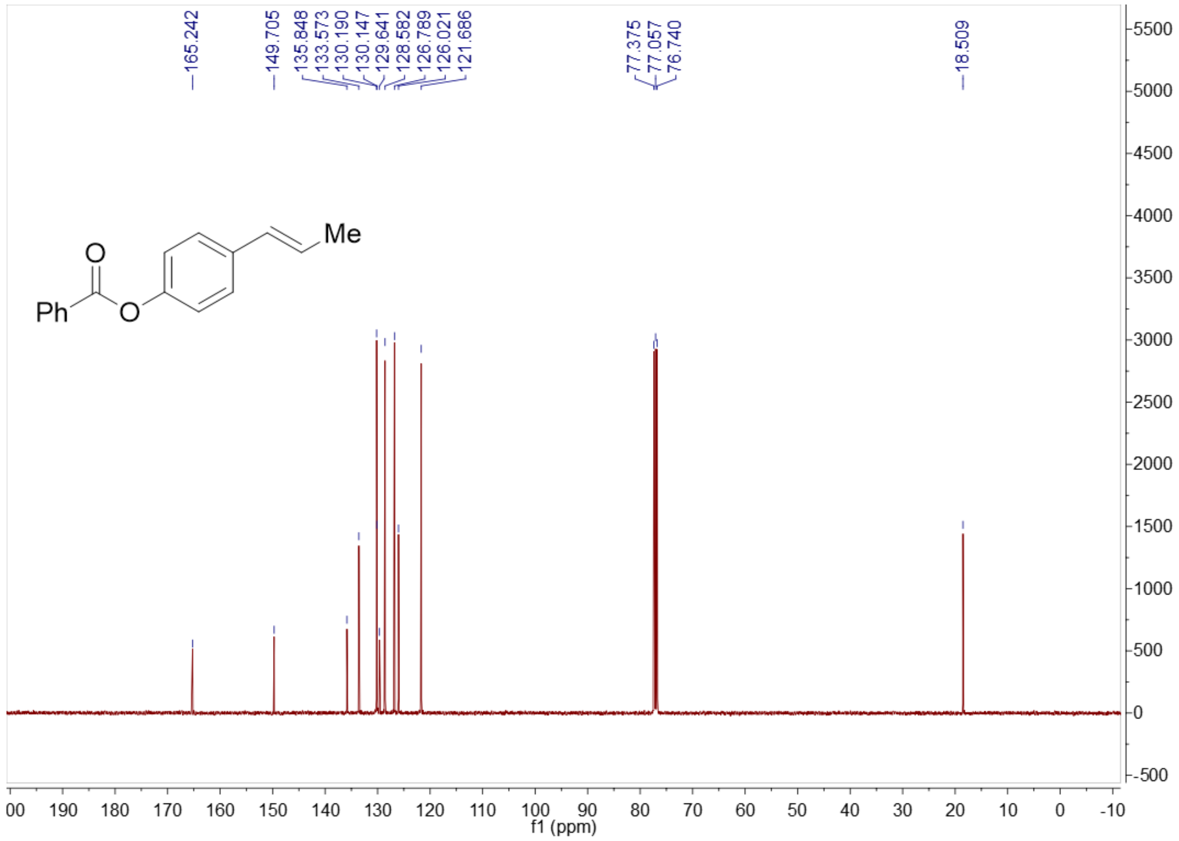

^{1}H NMR of compound **2l** in $CDCl_3$

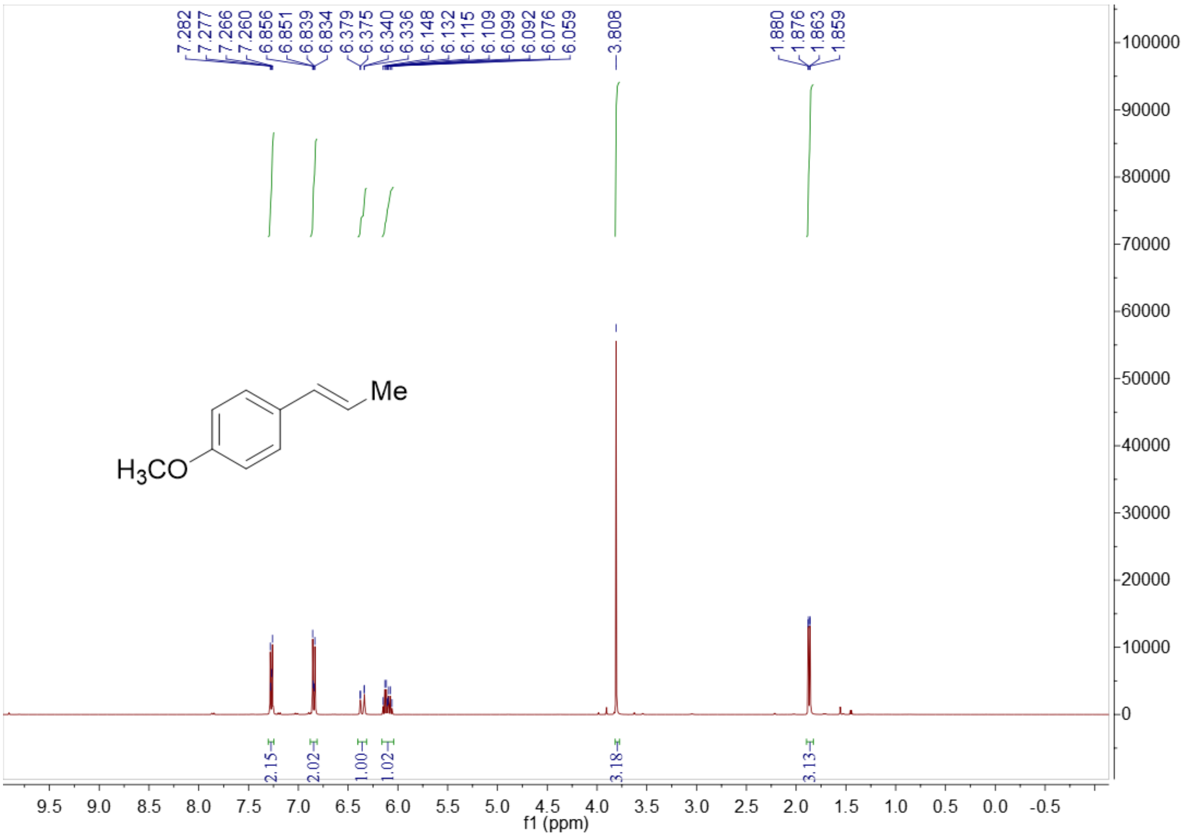

^{13}C NMR of compound **2l** in $CDCl_3$

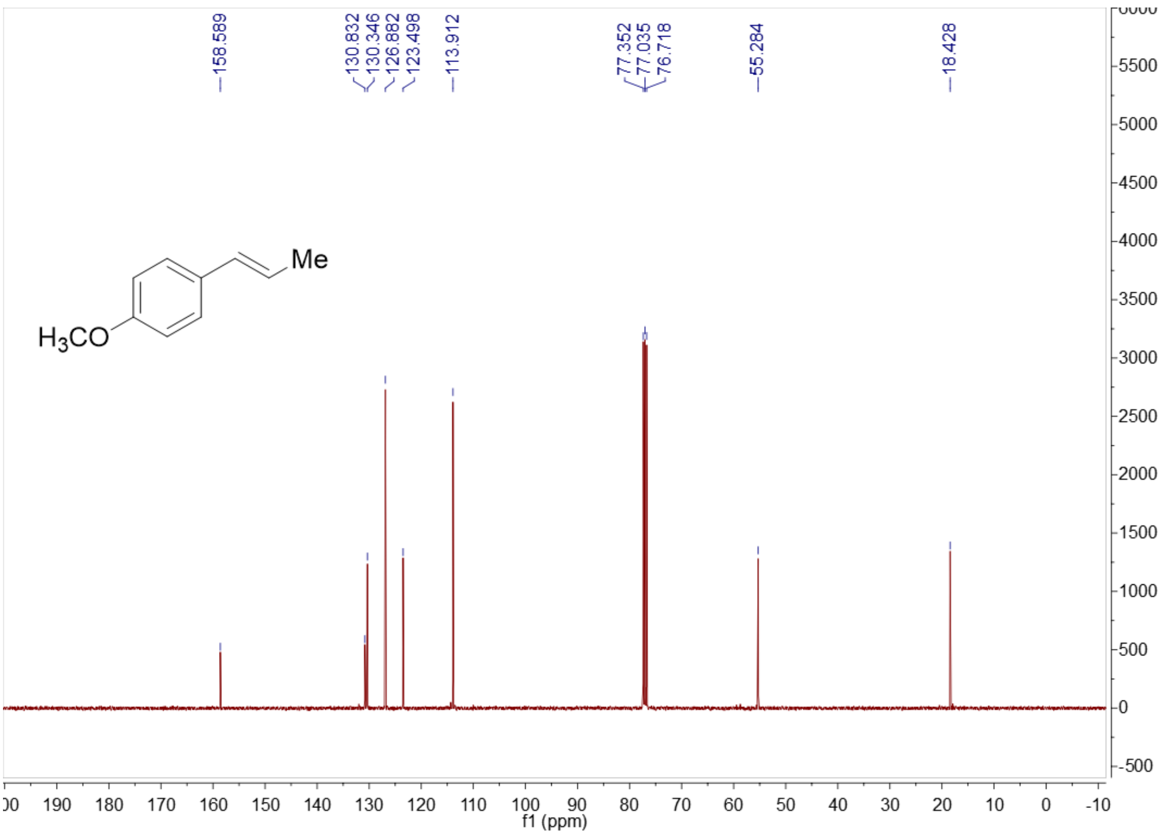

^{1}H NMR of compound **2m** in $CDCl_3$

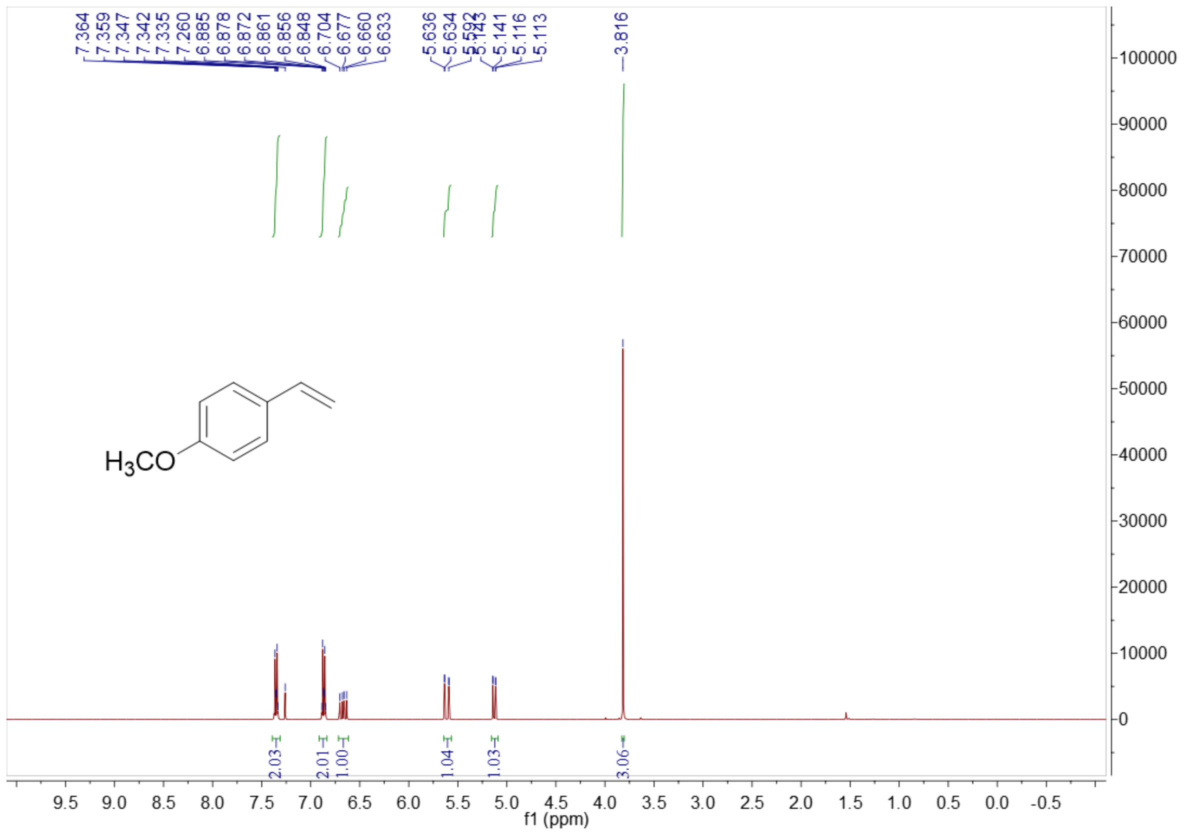

^{13}C NMR of compound **2m** in $CDCl_3$

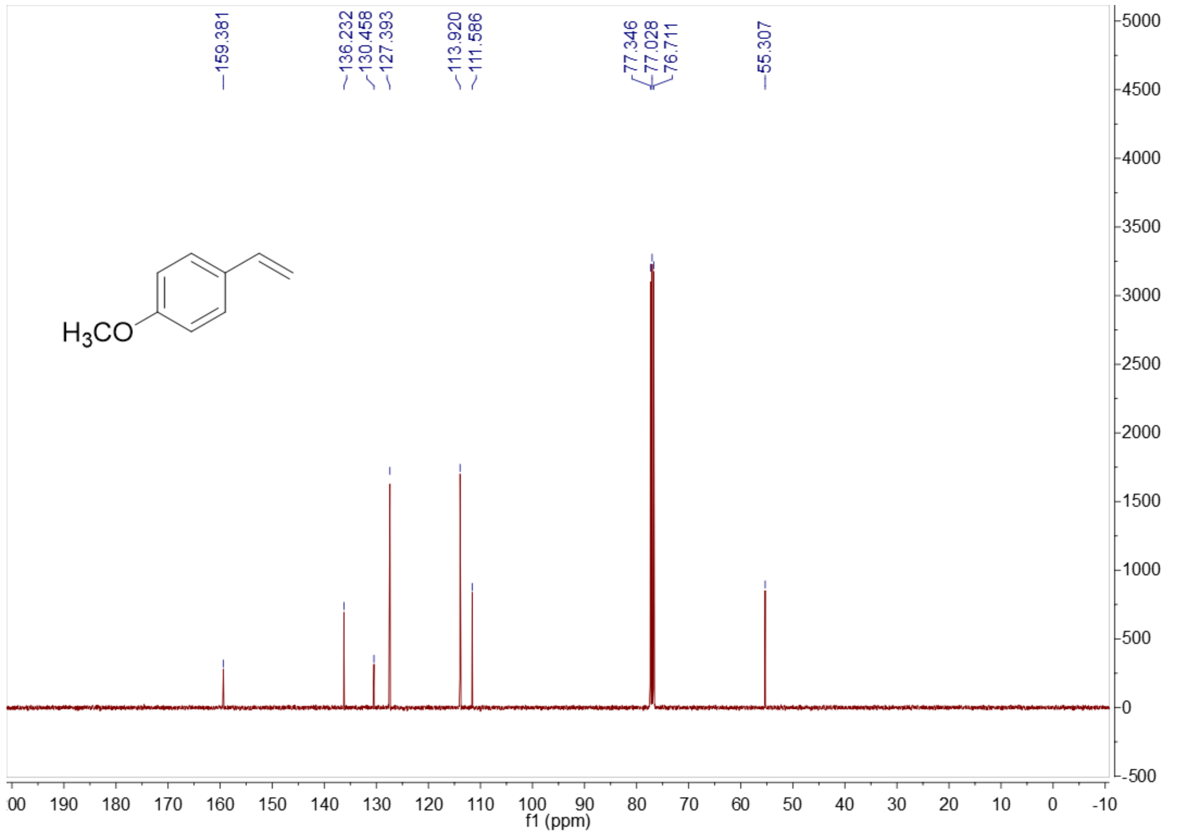

^{1}H NMR of compound **2n** in $CDCl_3$

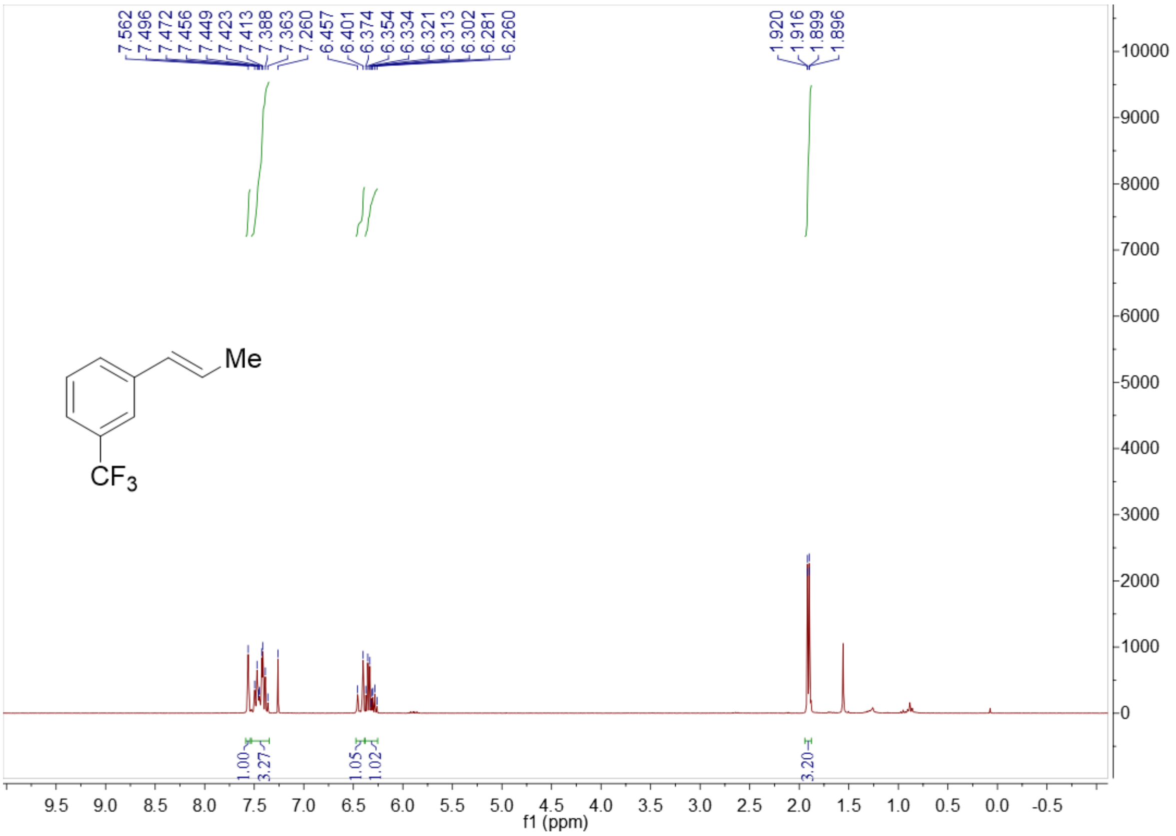

^{13}C NMR of compound **2n** in $CDCl_3$

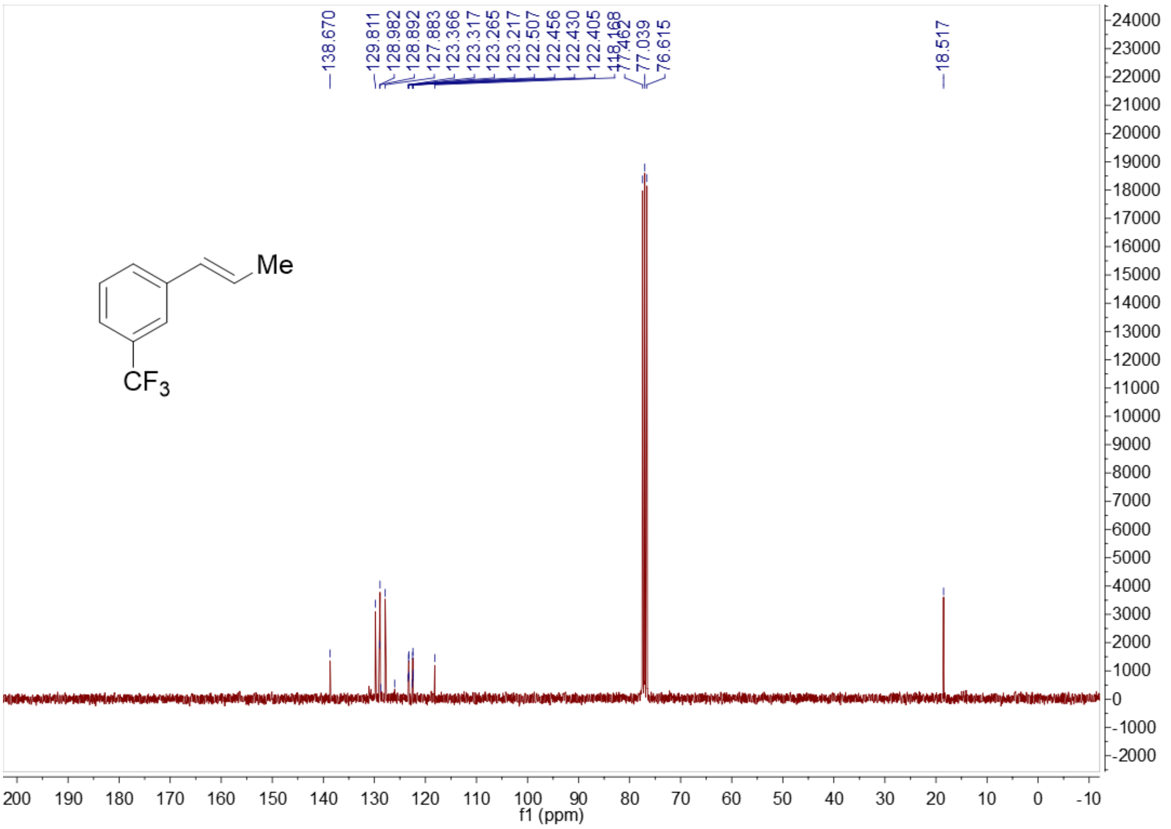

^{1}H NMR of compound **2o** in $CDCl_3$

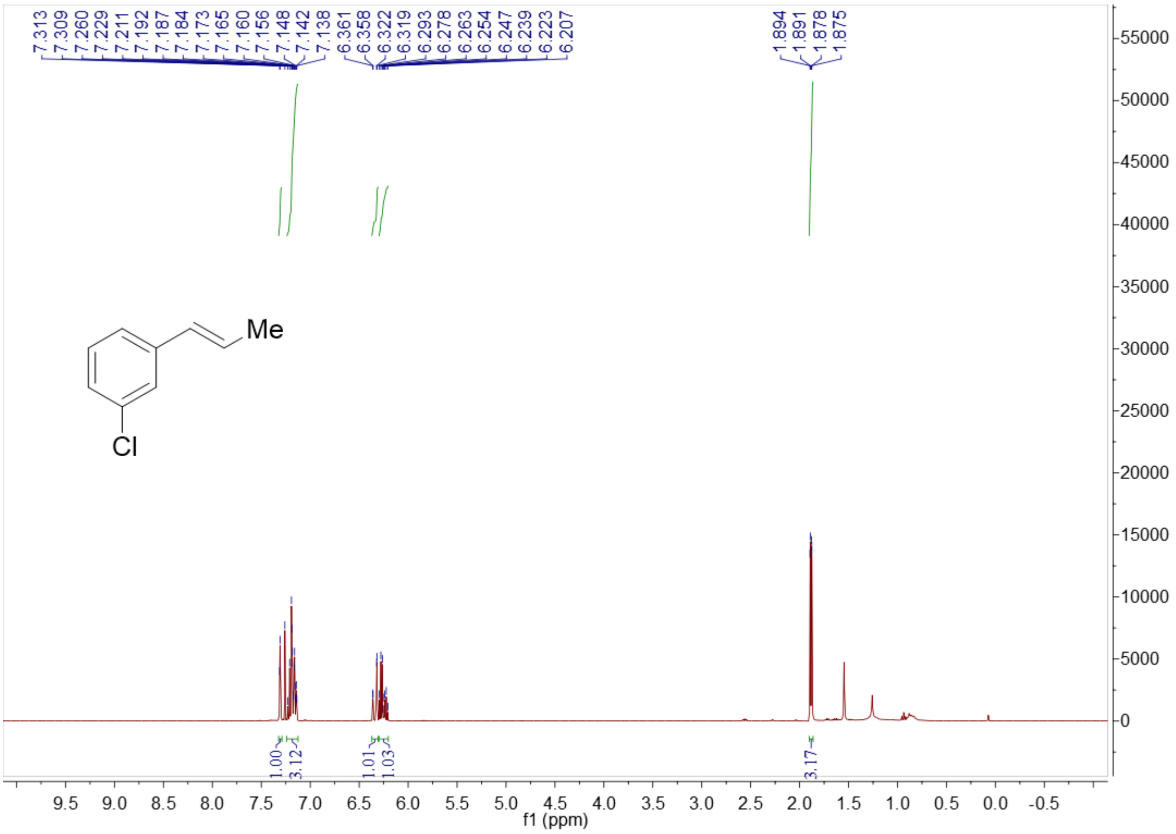

^{13}C NMR of compound **2o** in $CDCl_3$

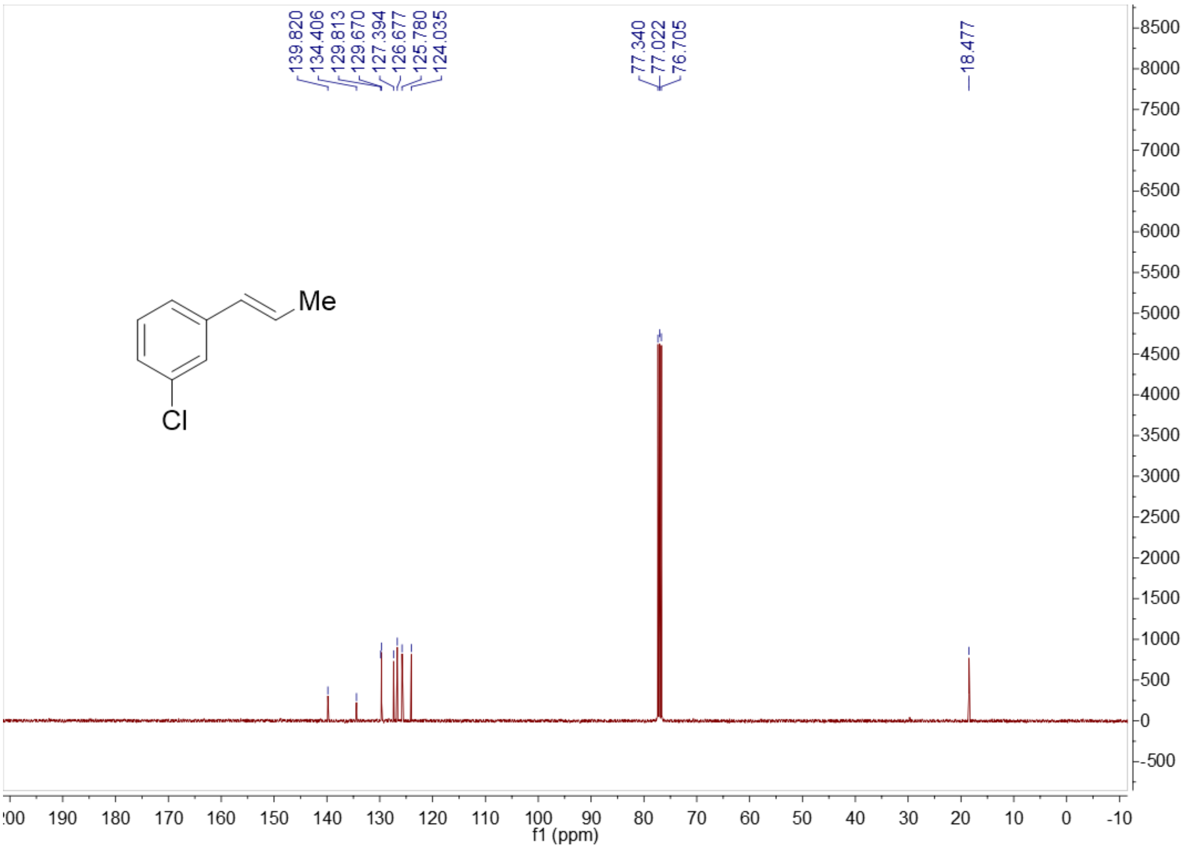

^{1}H NMR of compound **2p** in $CDCl_3$

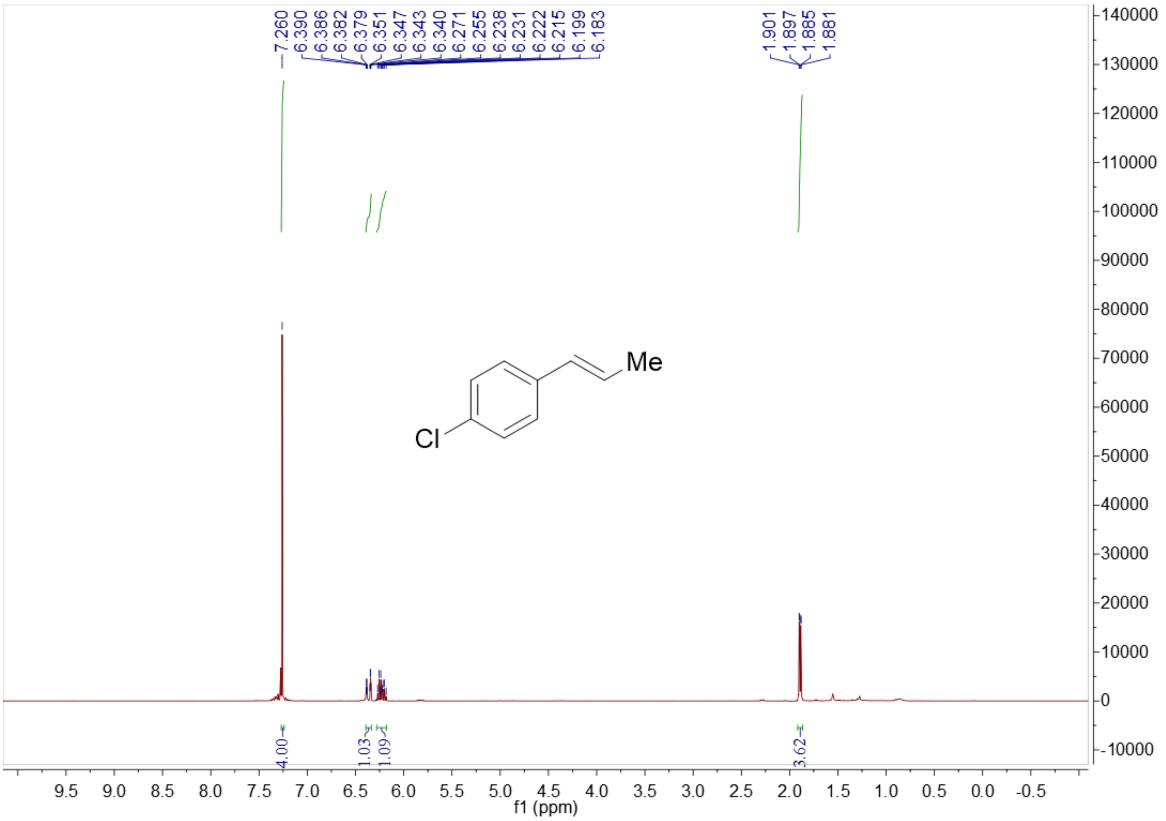

^{13}C NMR of compound **2p** in $CDCl_3$

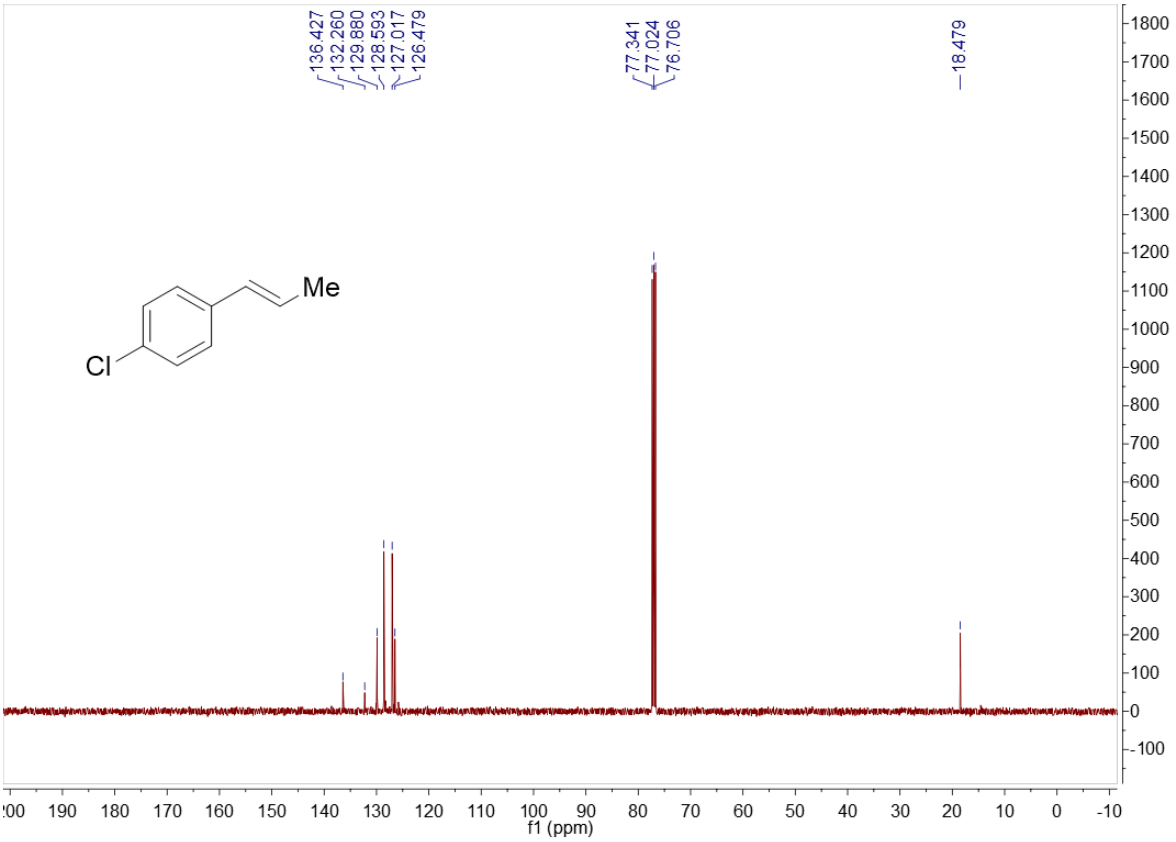

^{1}H NMR of compound **2q** in $CDCl_3$

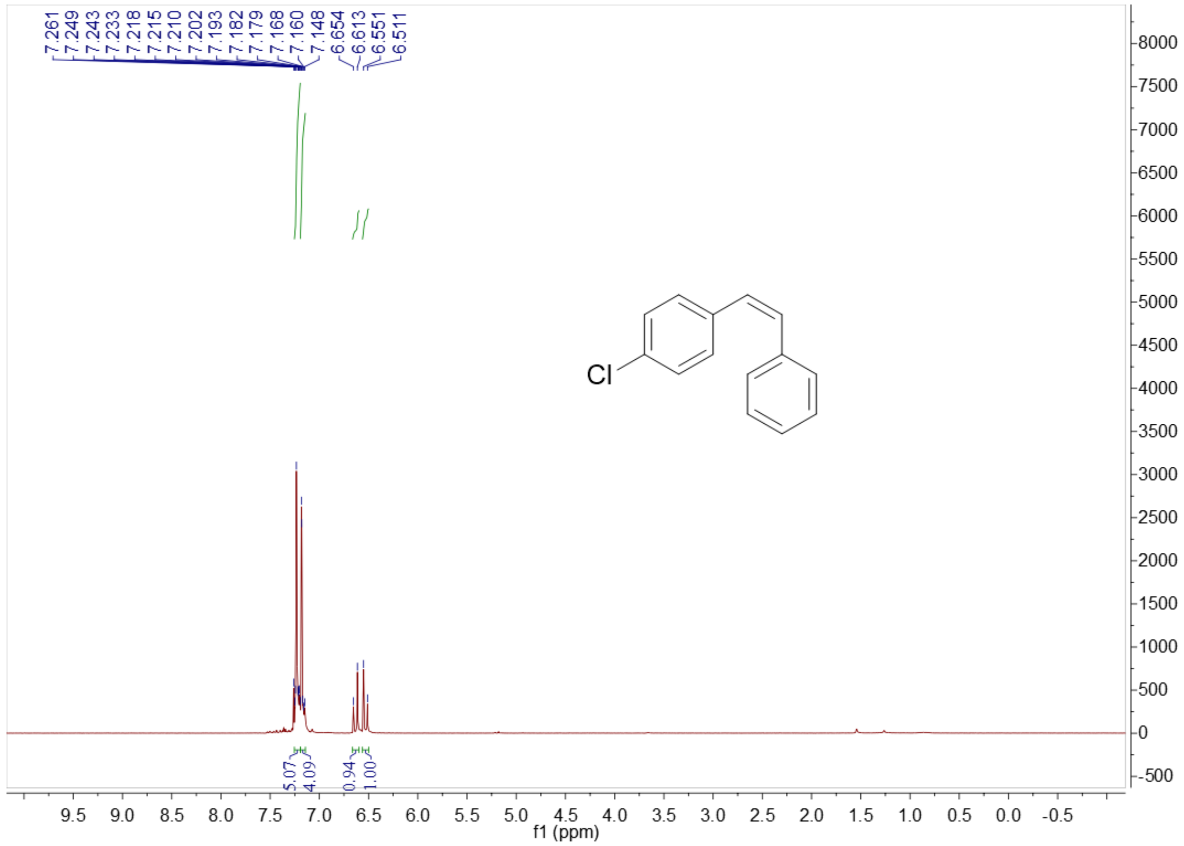

^{13}C NMR of compound **2q** in $CDCl_3$

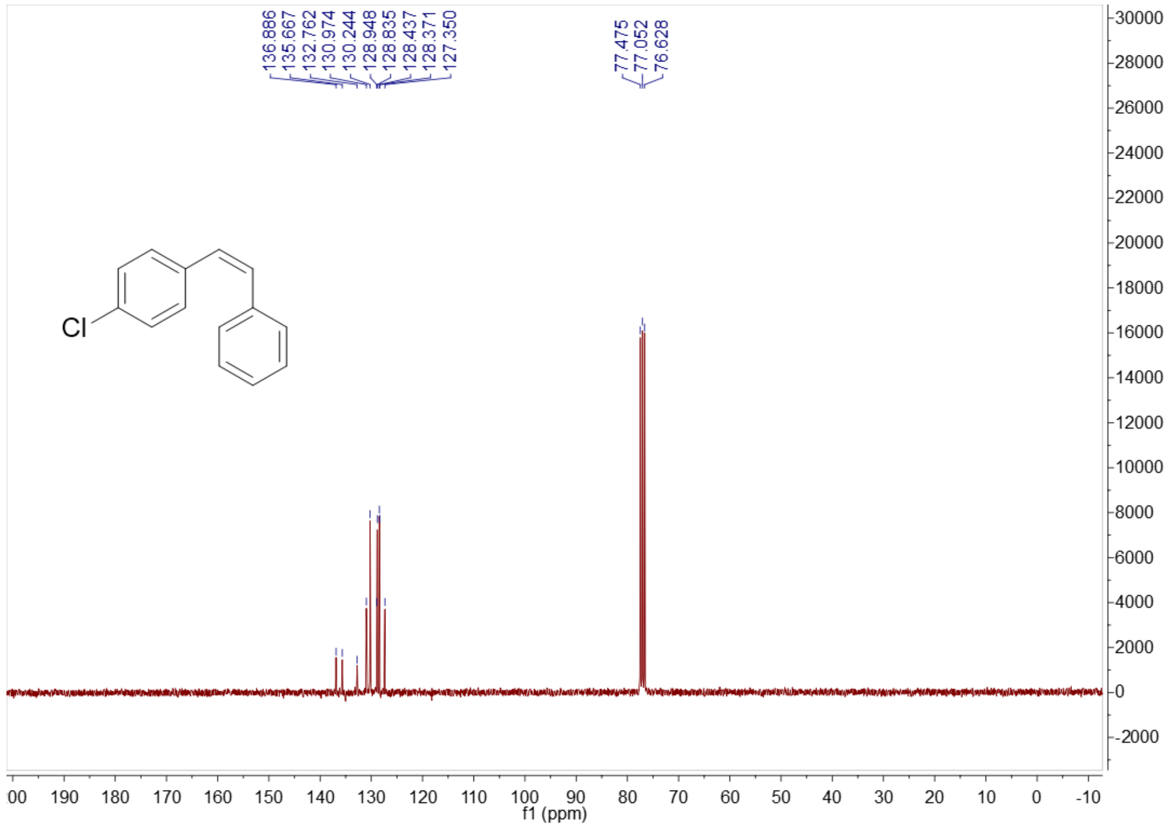

^{1}H NMR of compound **2r** in $CDCl_3$

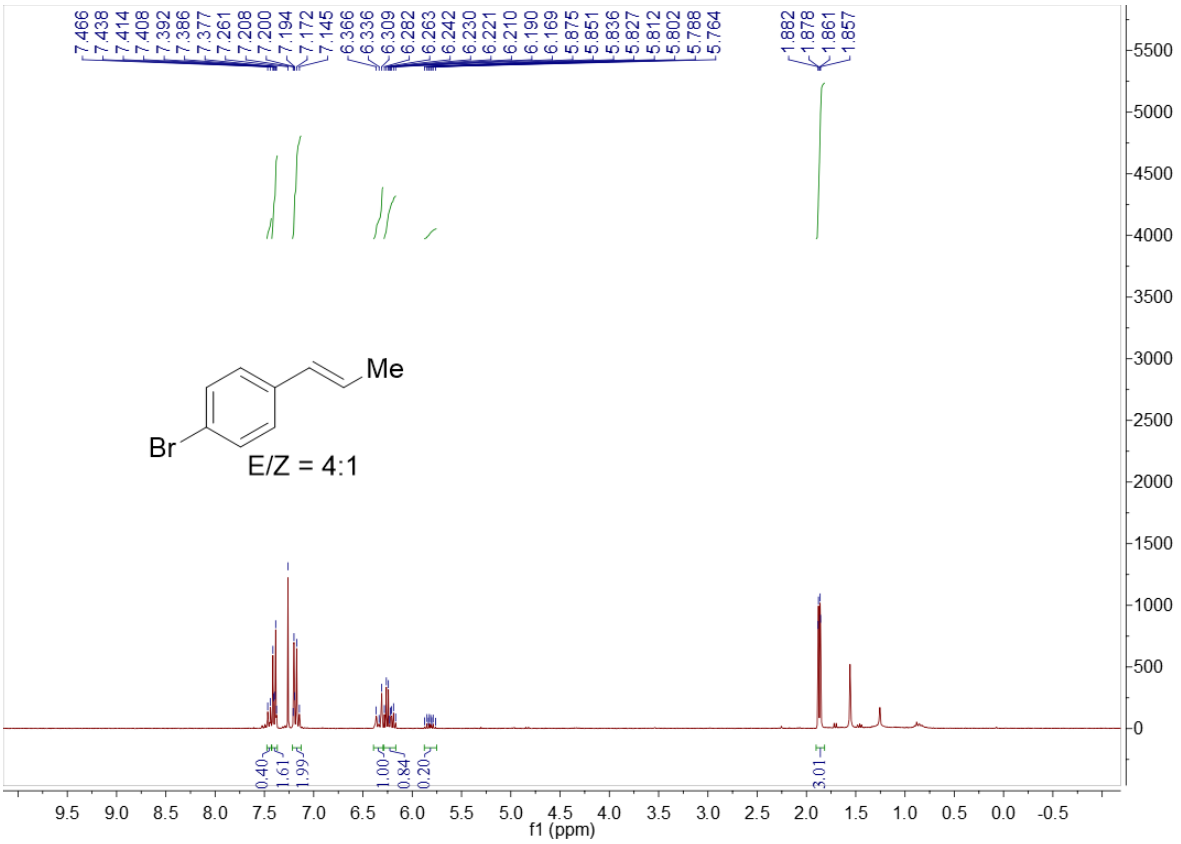

^{13}C NMR of compound **2r** in $CDCl_3$

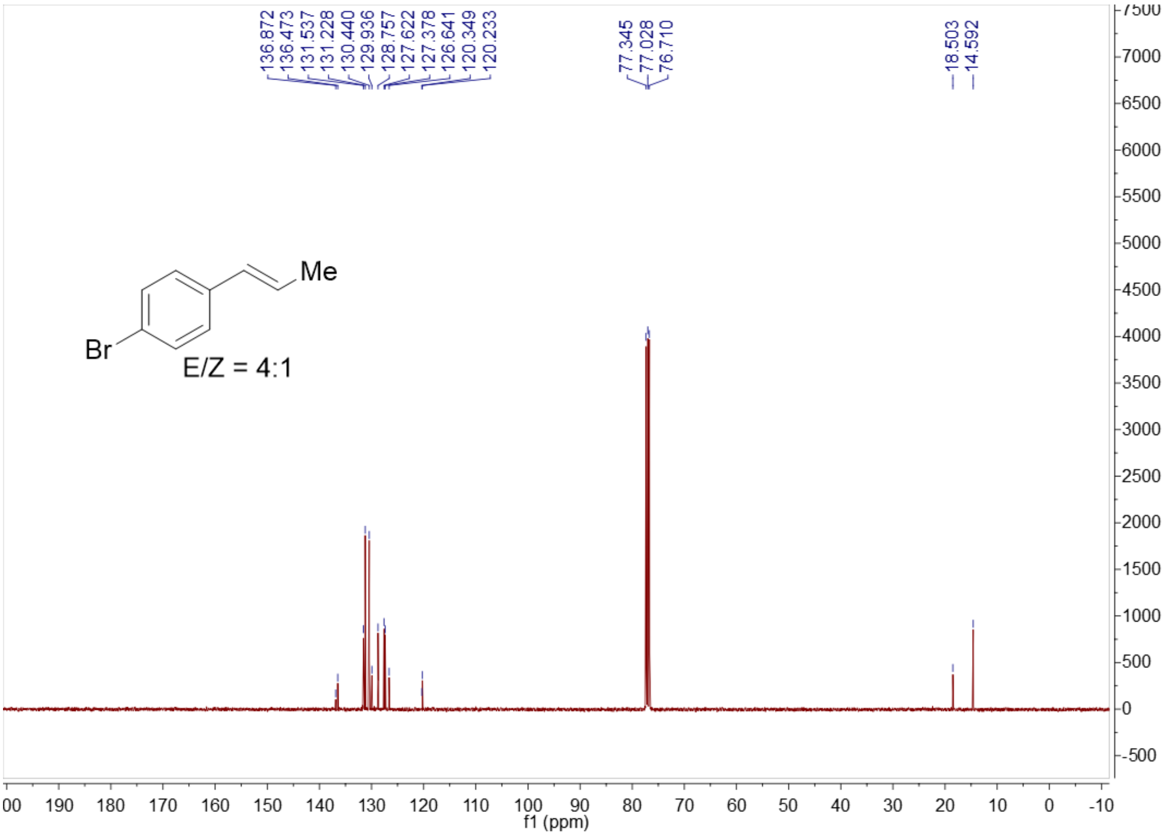

^{1}H NMR of compound **2s** in $CDCl_3$

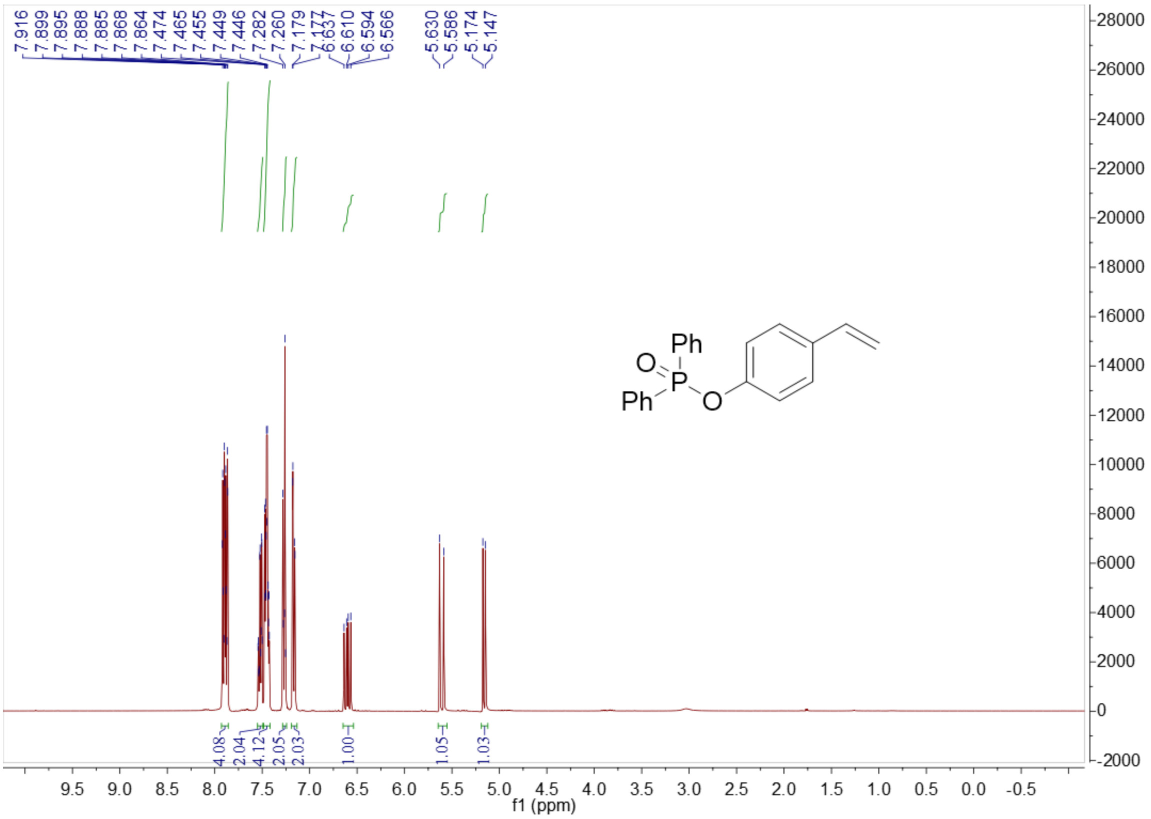

^{13}C NMR of compound **2s** in $CDCl_3$

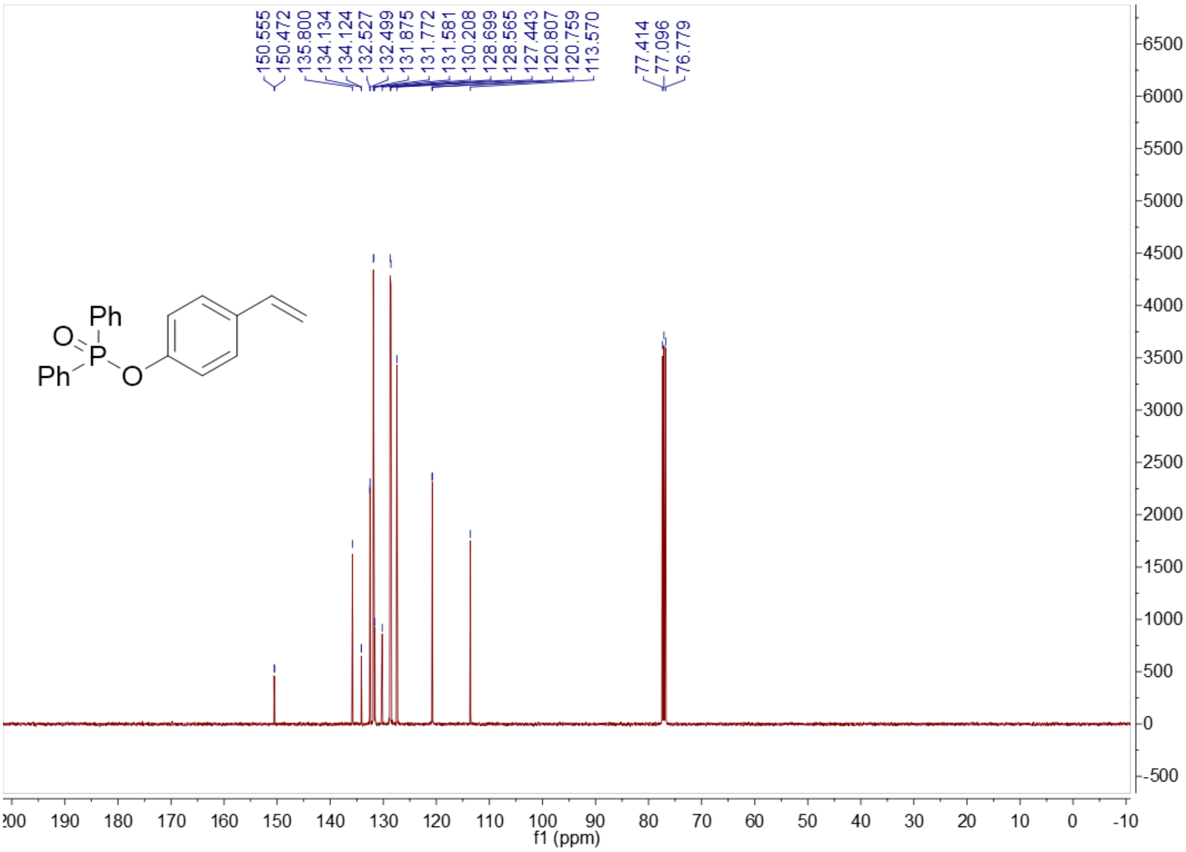

^{1}H NMR of compound **2t** in $CDCl_3$

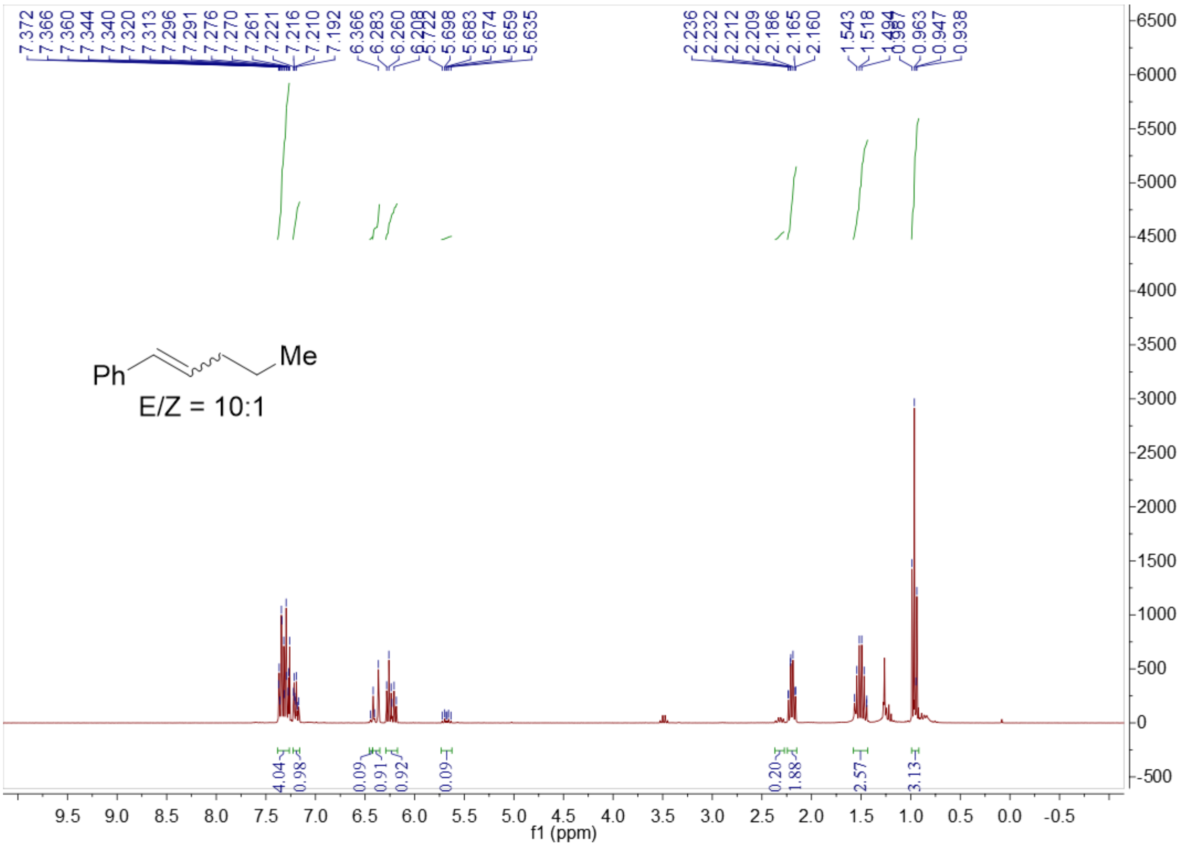

^{13}C NMR of compound **2t** in $CDCl_3$

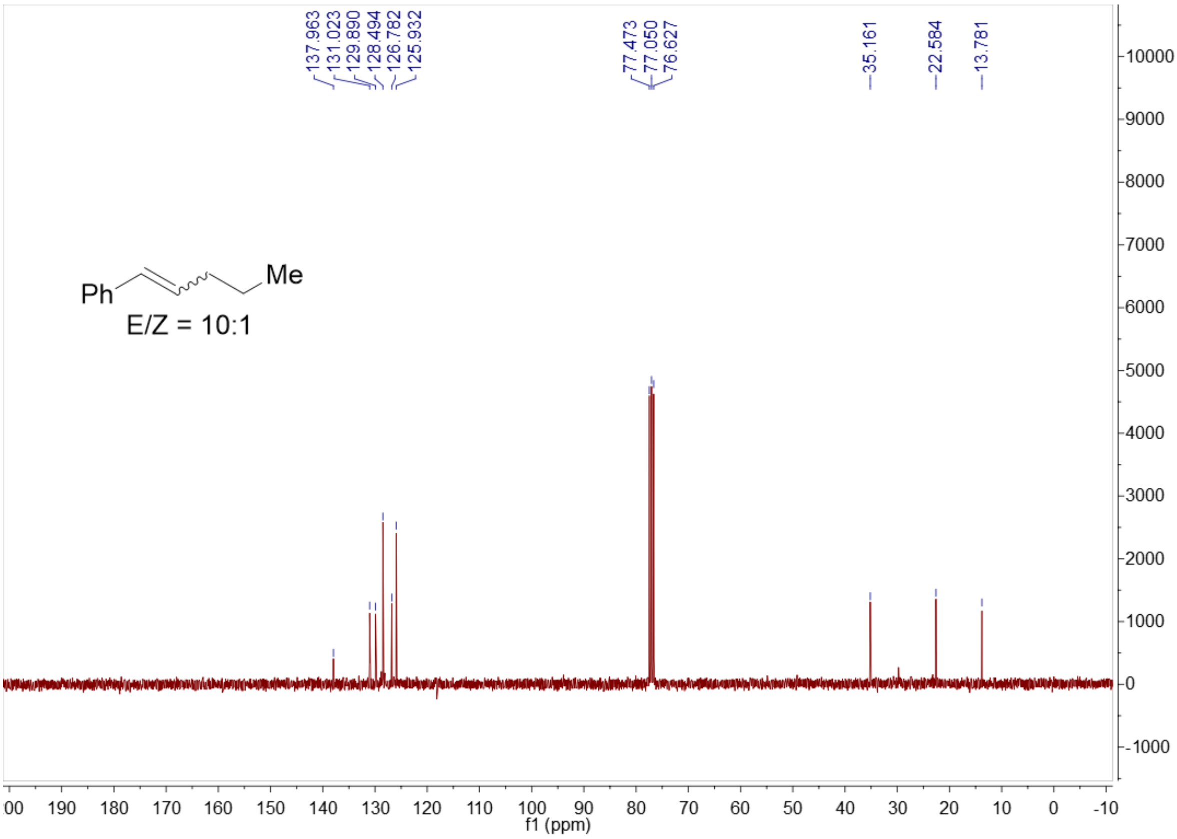

^{1}H NMR of compound **2u** in $CDCl_3$

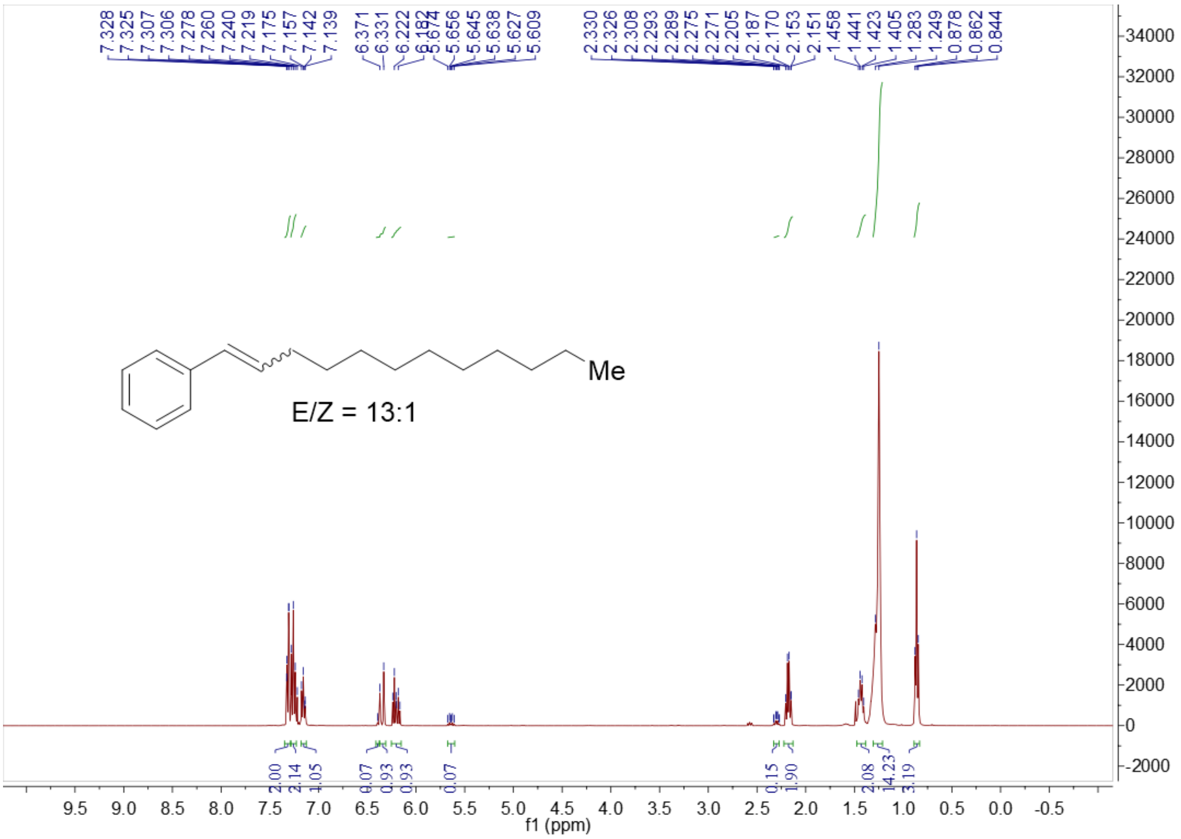

^{13}C NMR of compound **2u** in $CDCl_3$

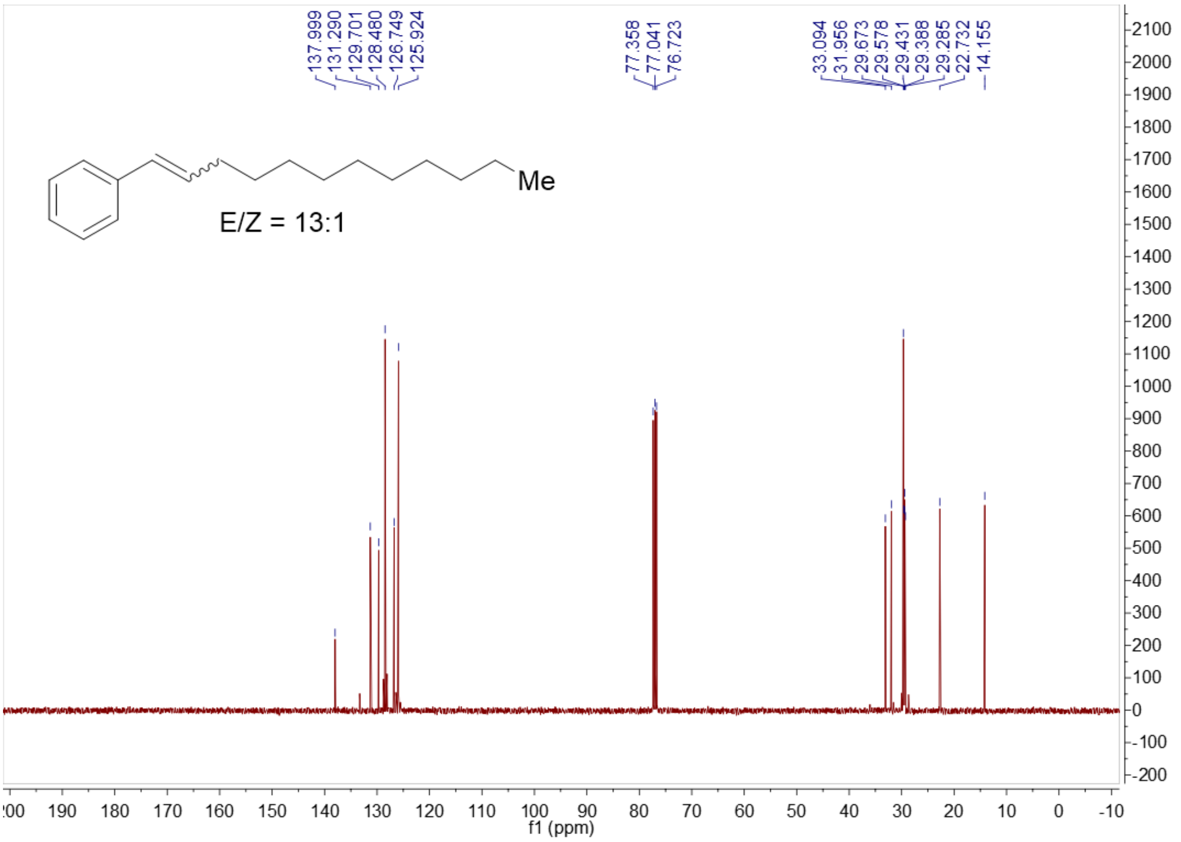

^{1}H NMR of compound **2v** in $CDCl_3$

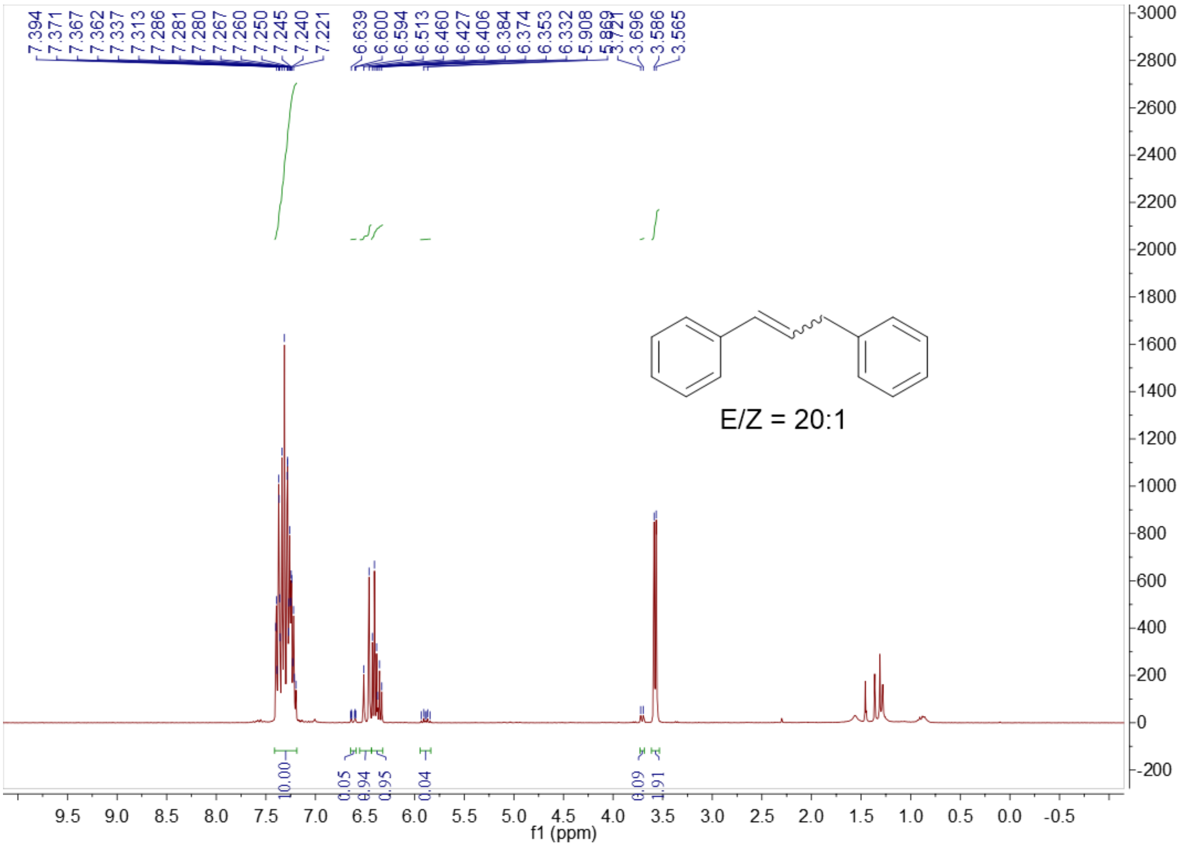

^{13}C NMR of compound **2v** in $CDCl_3$

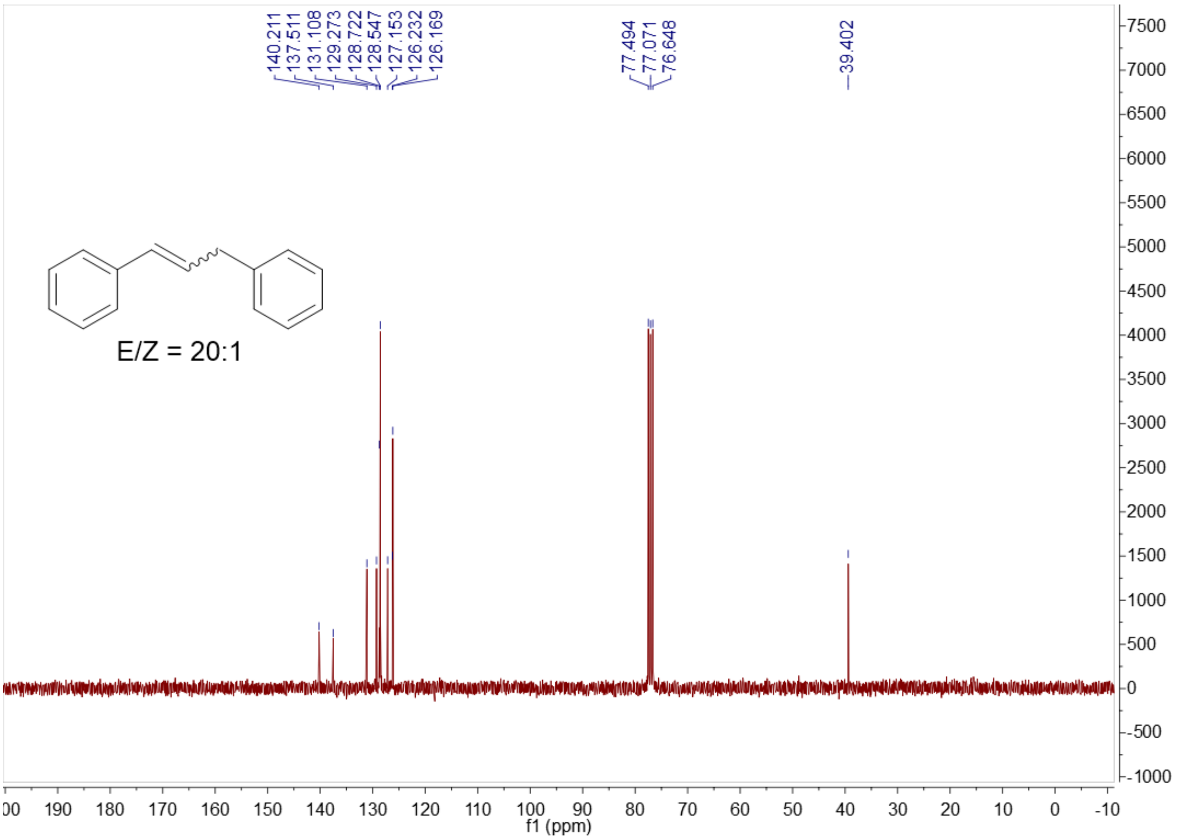

^{1}H NMR of compound **2w** in $CDCl_3$

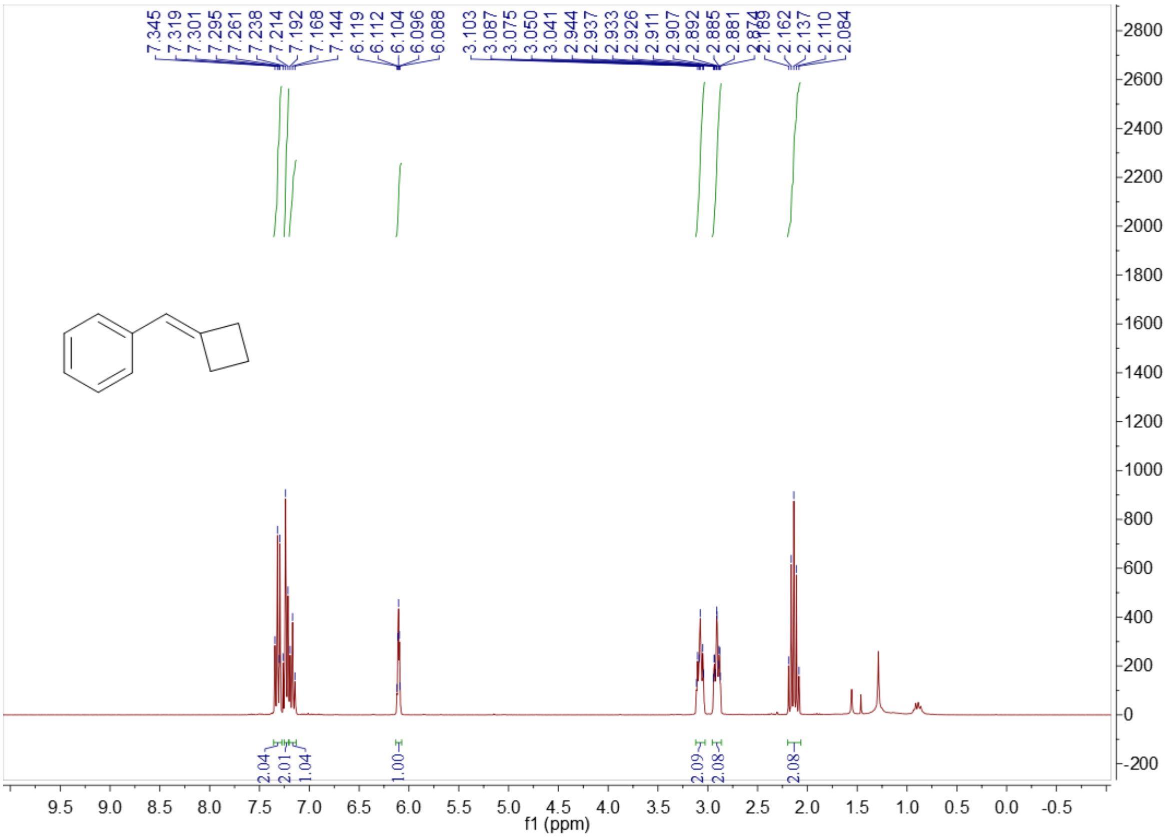

^{13}C NMR of compound **2w** in $CDCl_3$

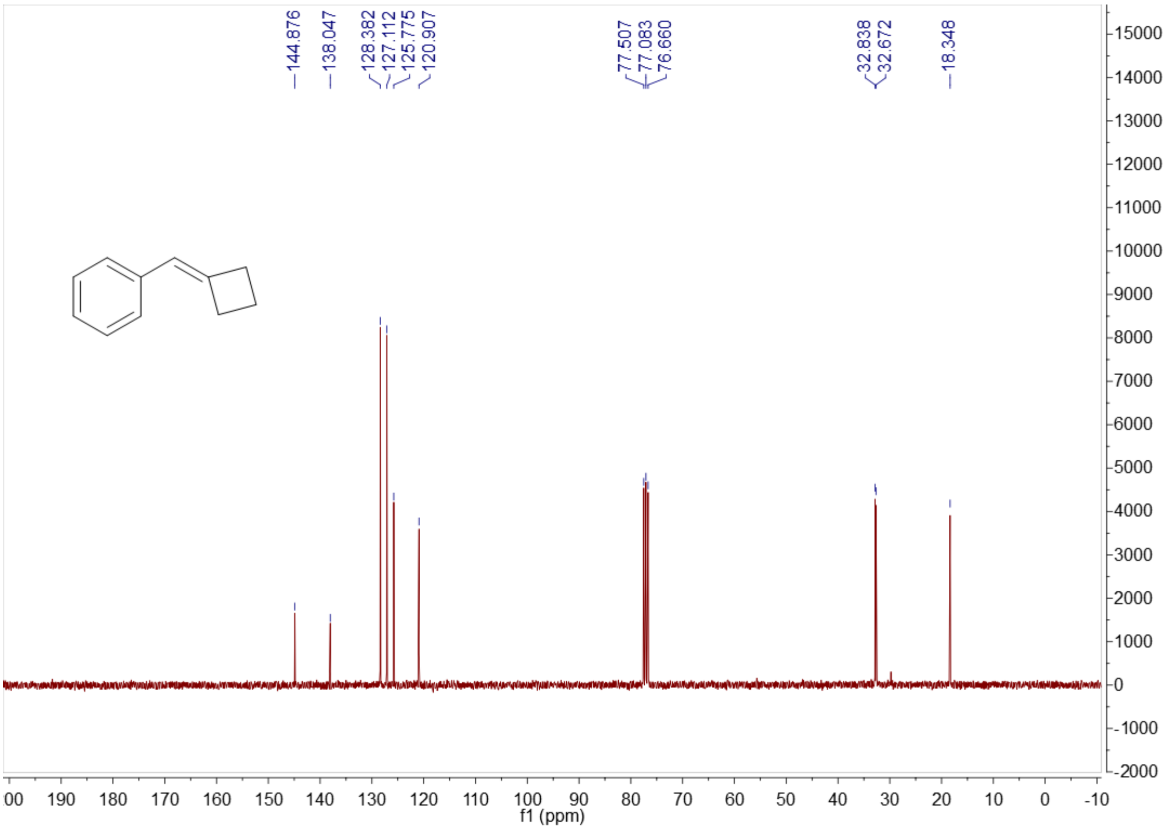

^{1}H NMR of compound **2x** in $CDCl_3$

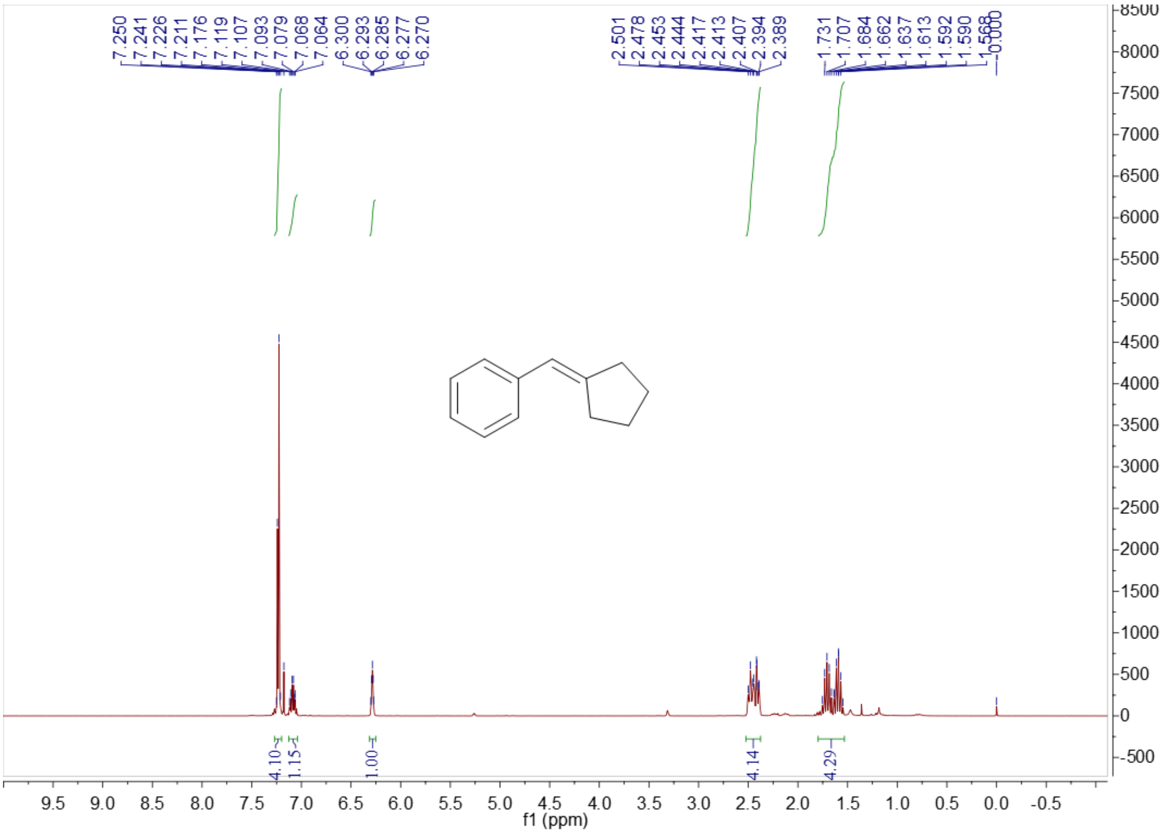

^{13}C NMR of compound **2x** in $CDCl_3$

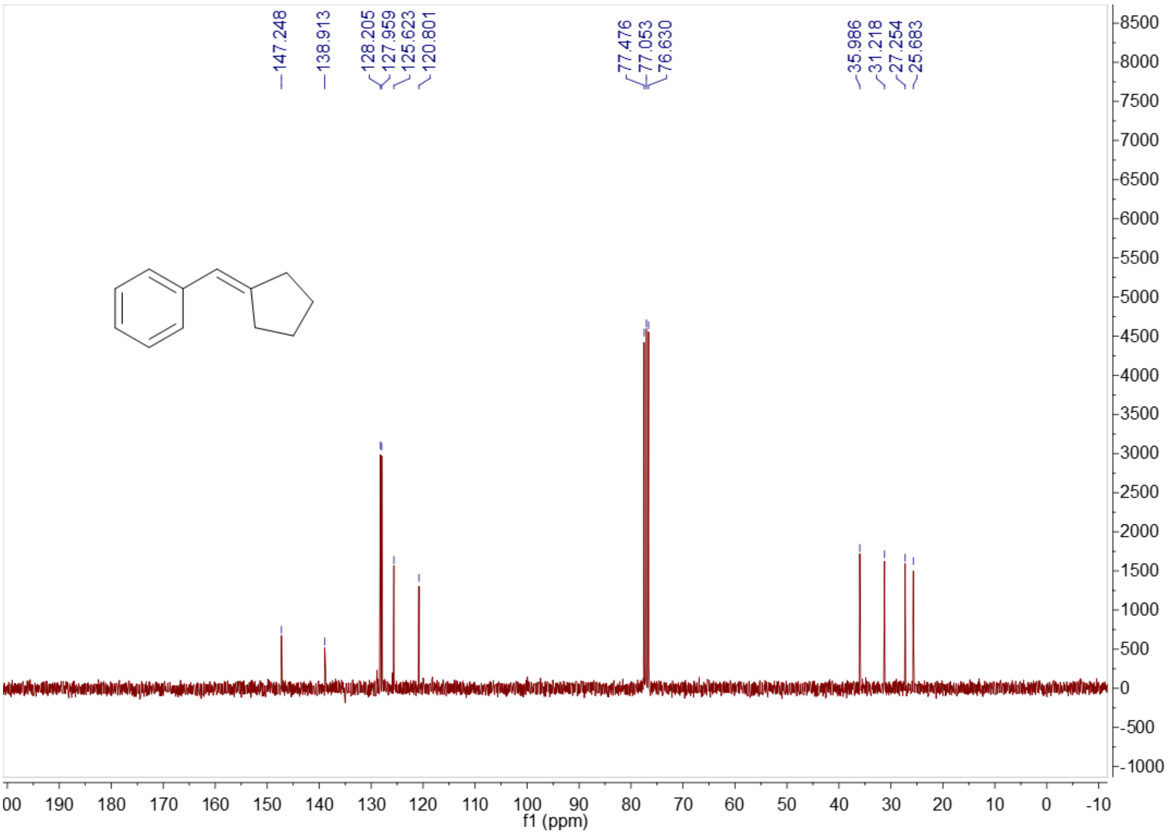

^{1}H NMR of compound **2y** in $CDCl_3$

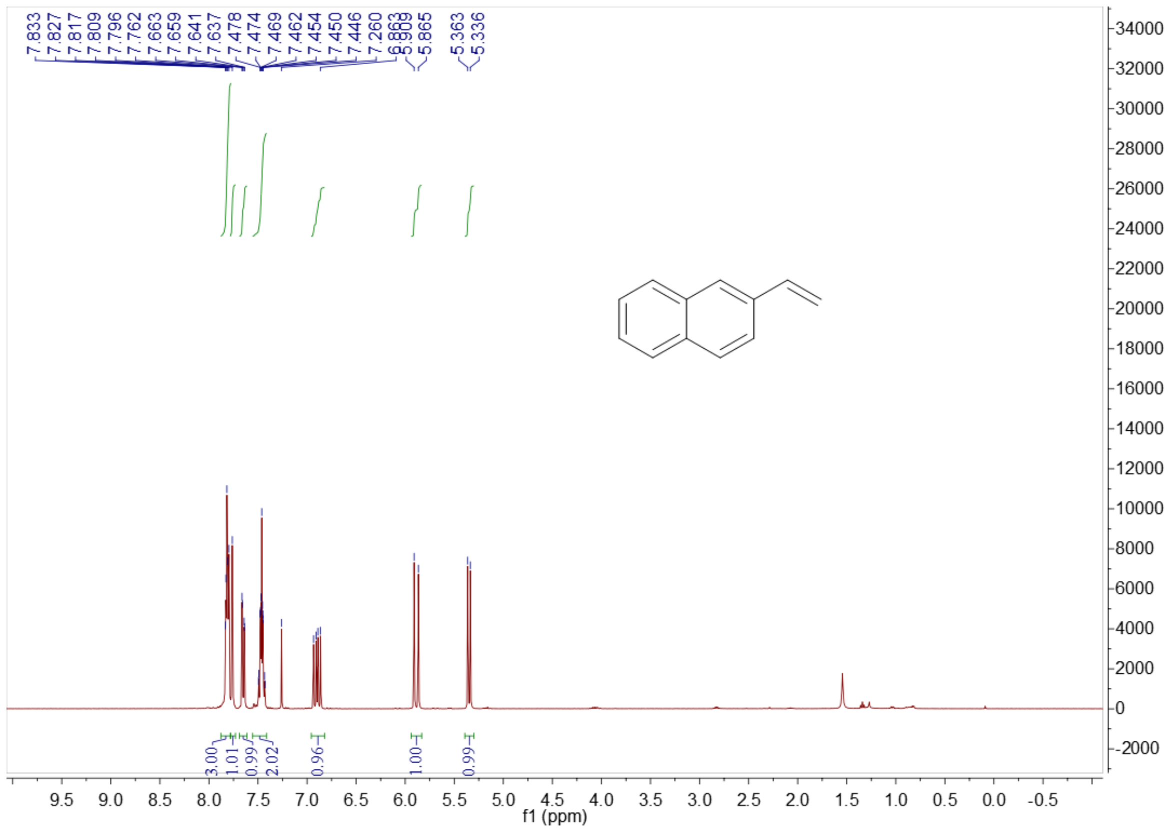

^{13}C NMR of compound **2y** in $CDCl_3$

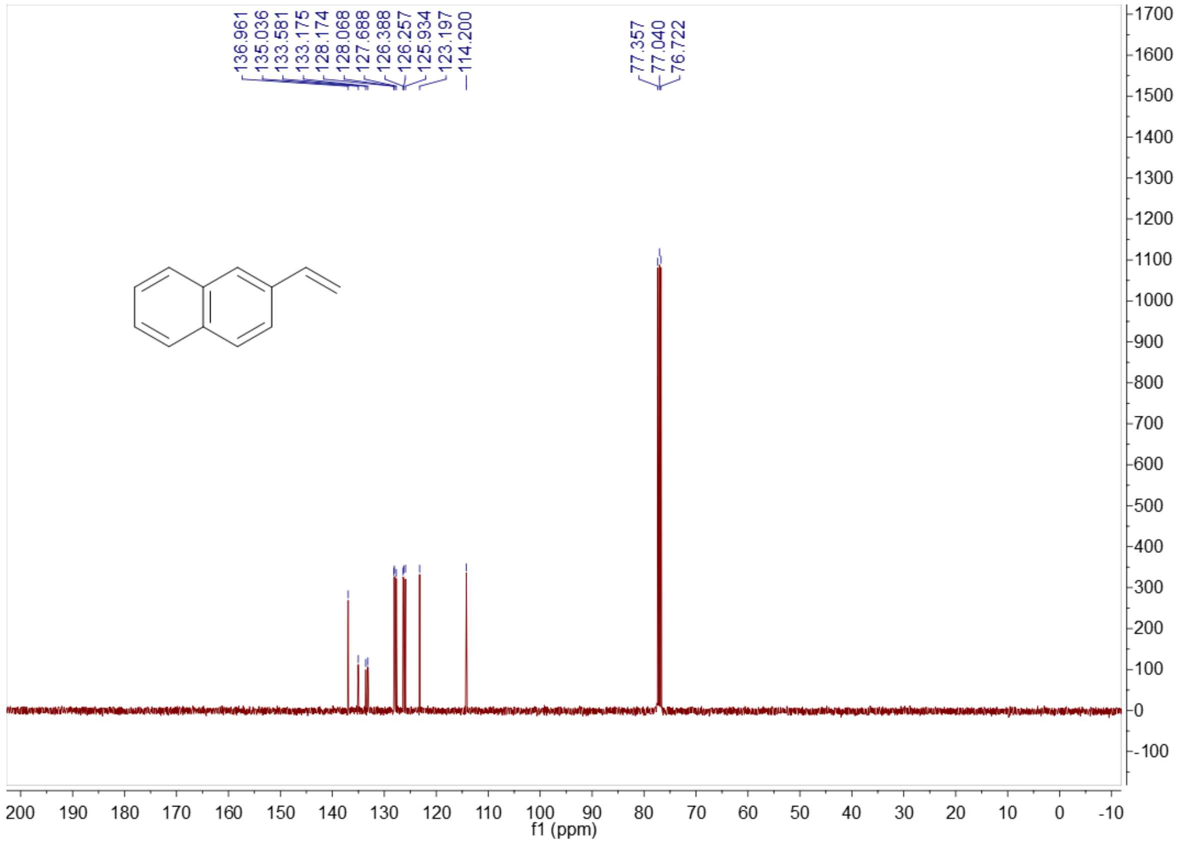

^{1}H NMR of compound **2z** in $CDCl_3$

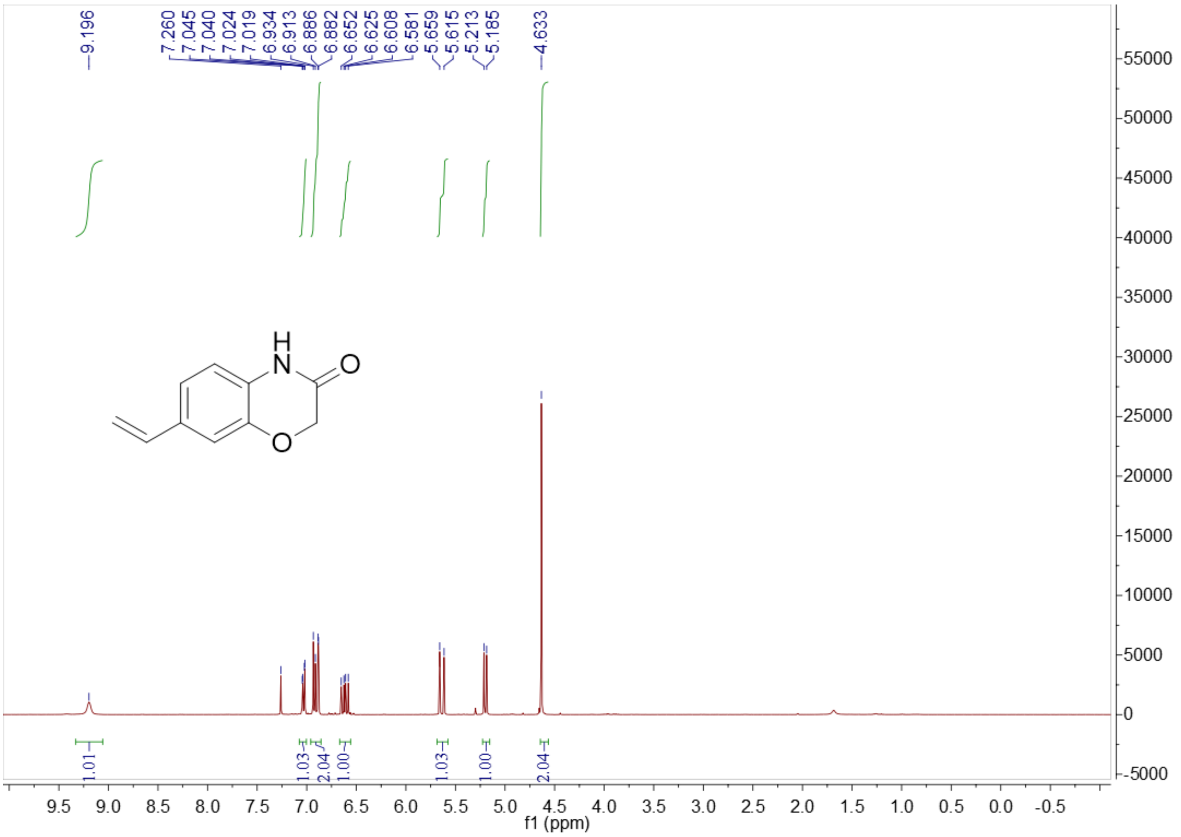

^{13}C NMR of compound **2z** in $CDCl_3$

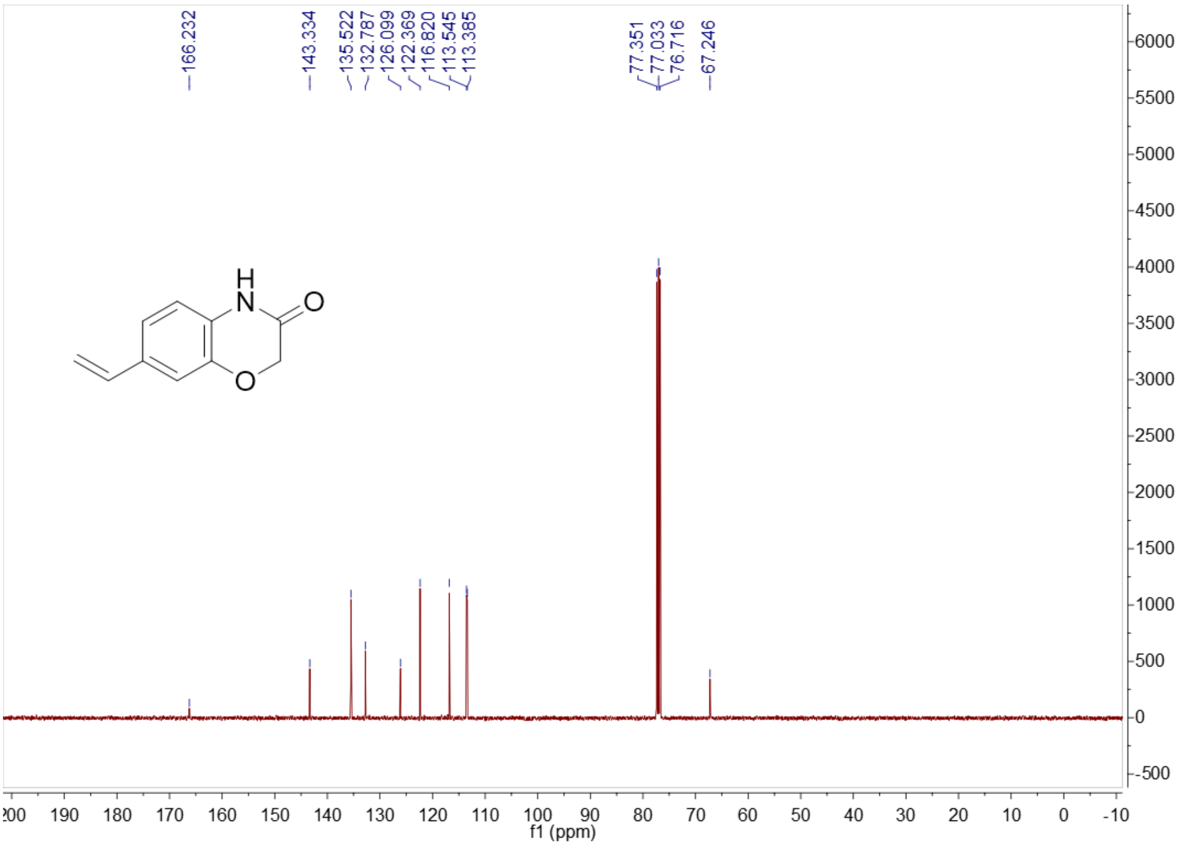

^{1}H NMR of compound **2ab** in $CDCl_3$

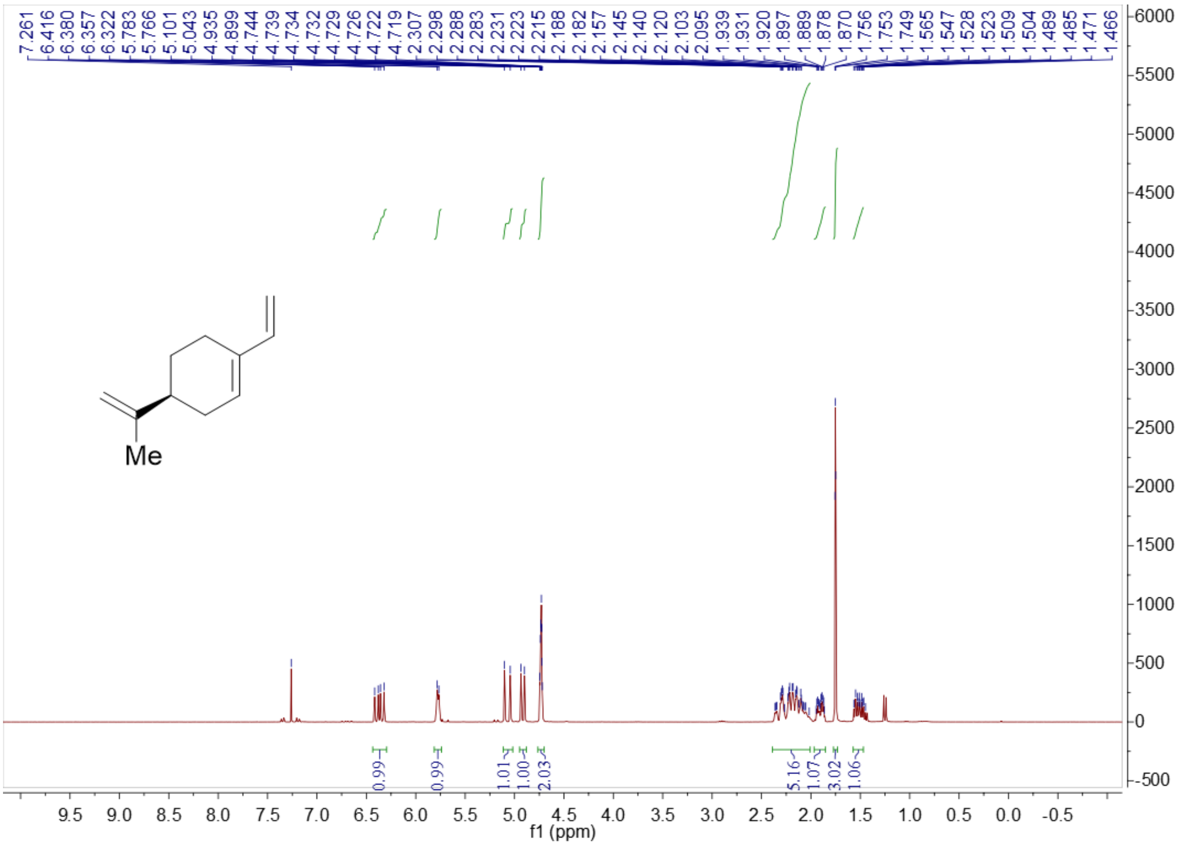

^{13}C NMR of compound **2ab** in $CDCl_3$

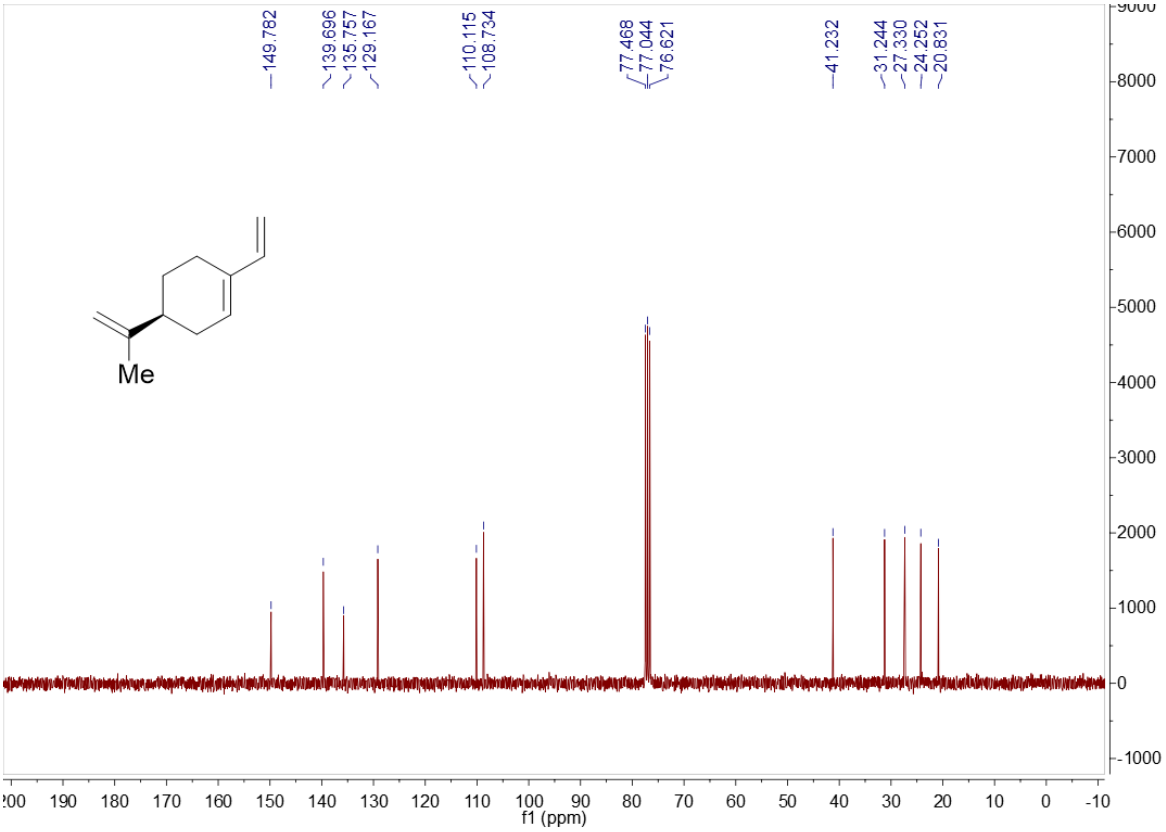

^{1}H NMR of compound **2ac** in $CDCl_3$

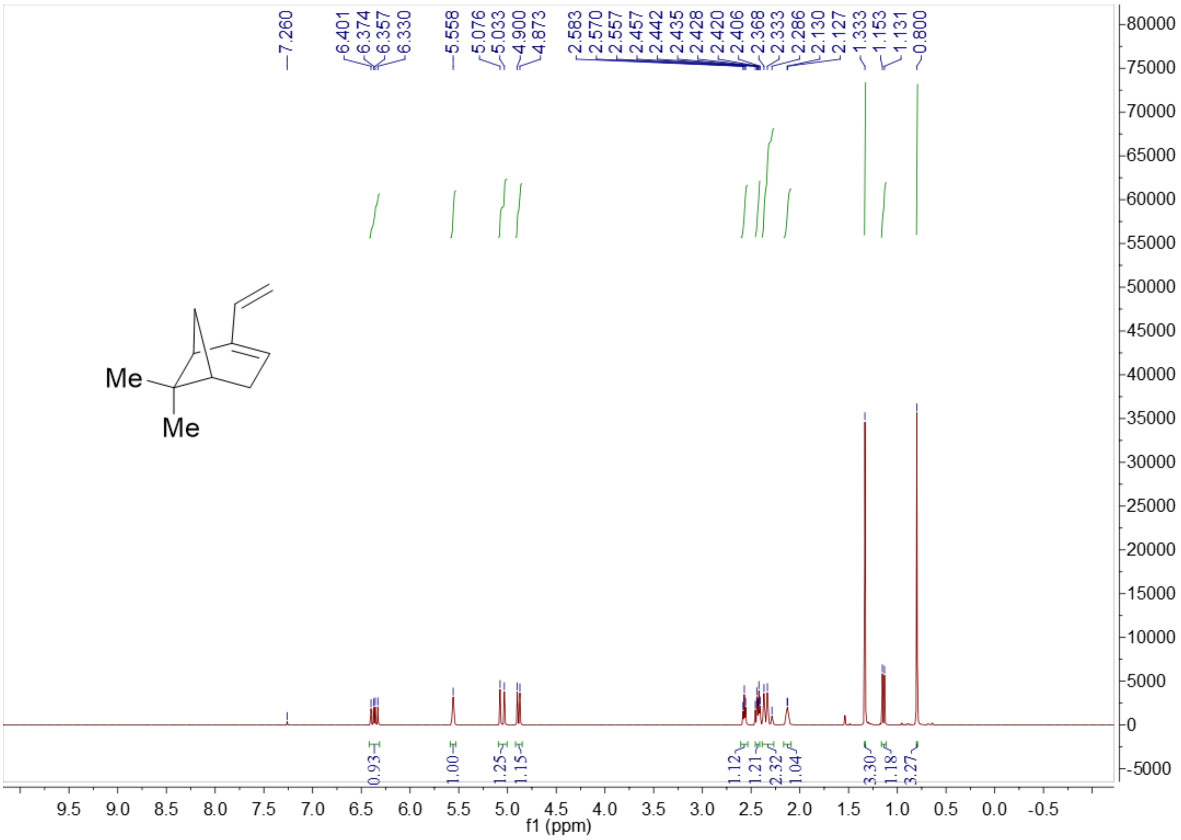

^{13}C NMR of compound **2ac** in $CDCl_3$

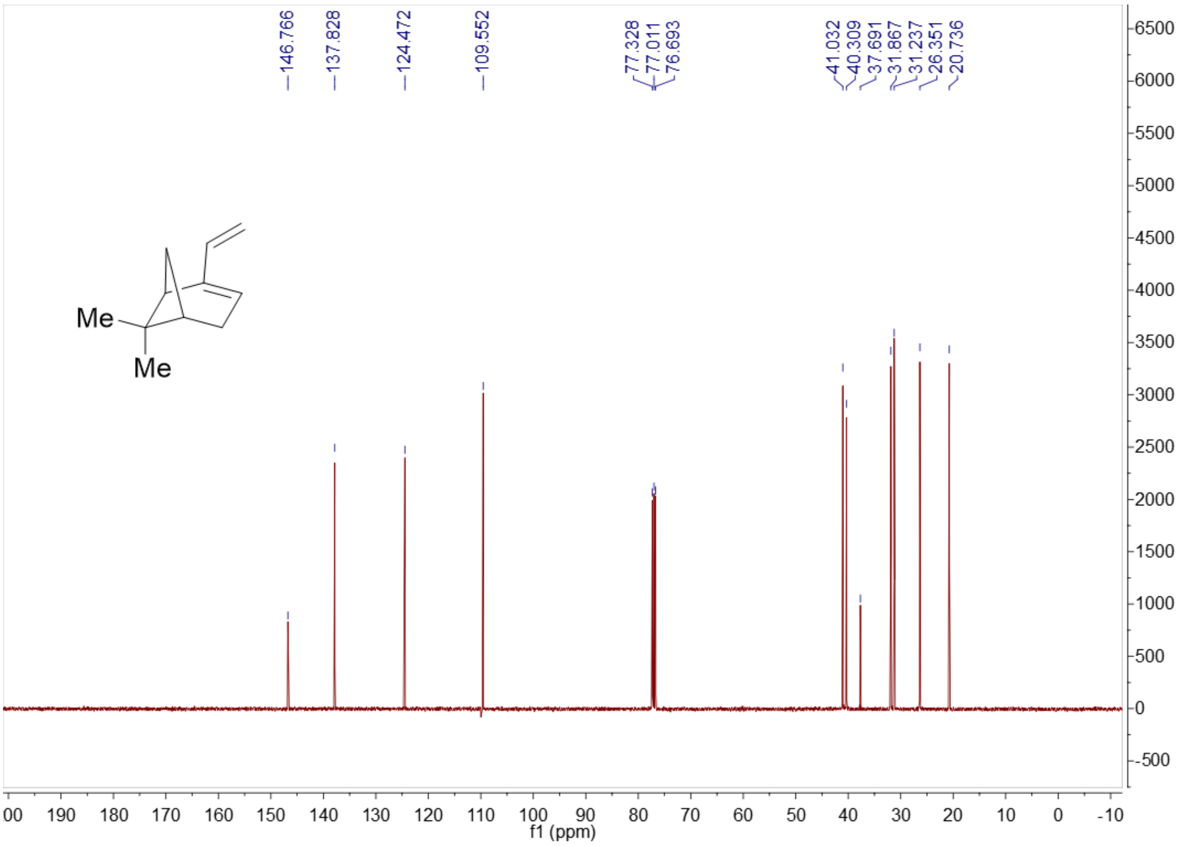

^{1}H NMR of compound **2ad** in $CDCl_3$

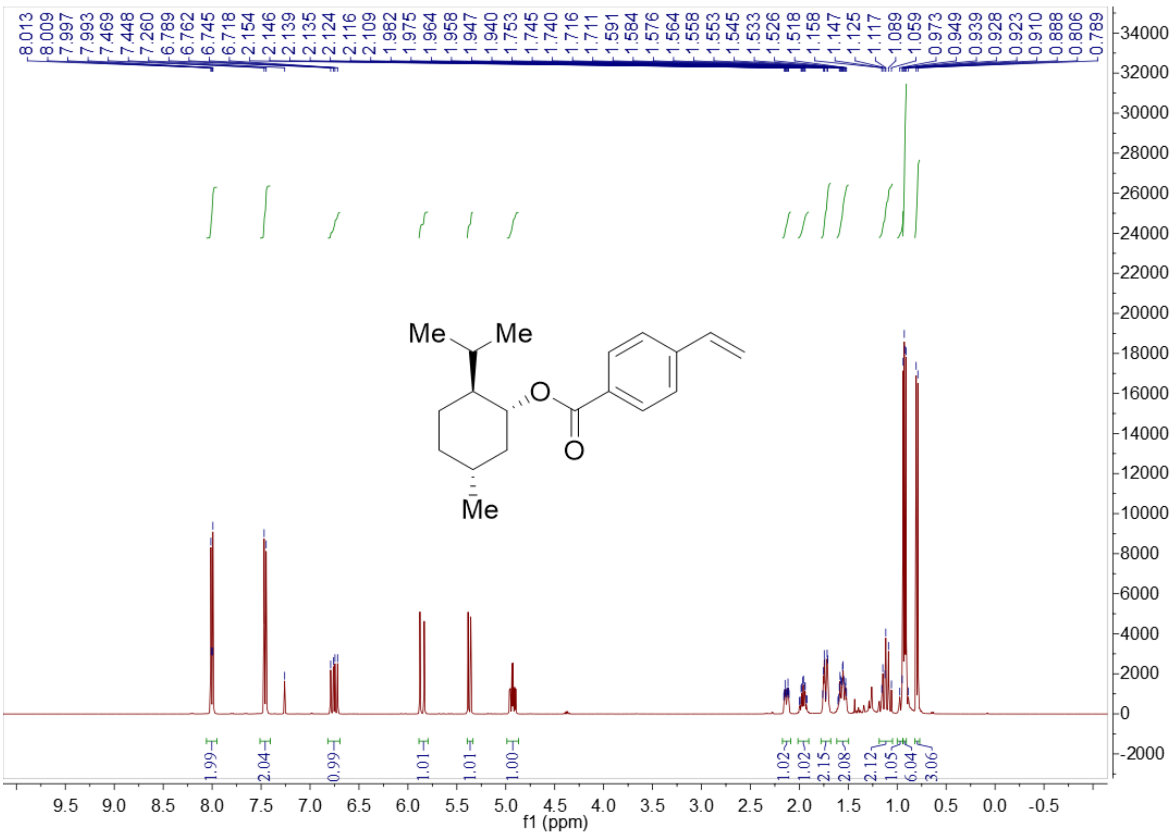

^{13}C NMR of compound **2ad** in $CDCl_3$

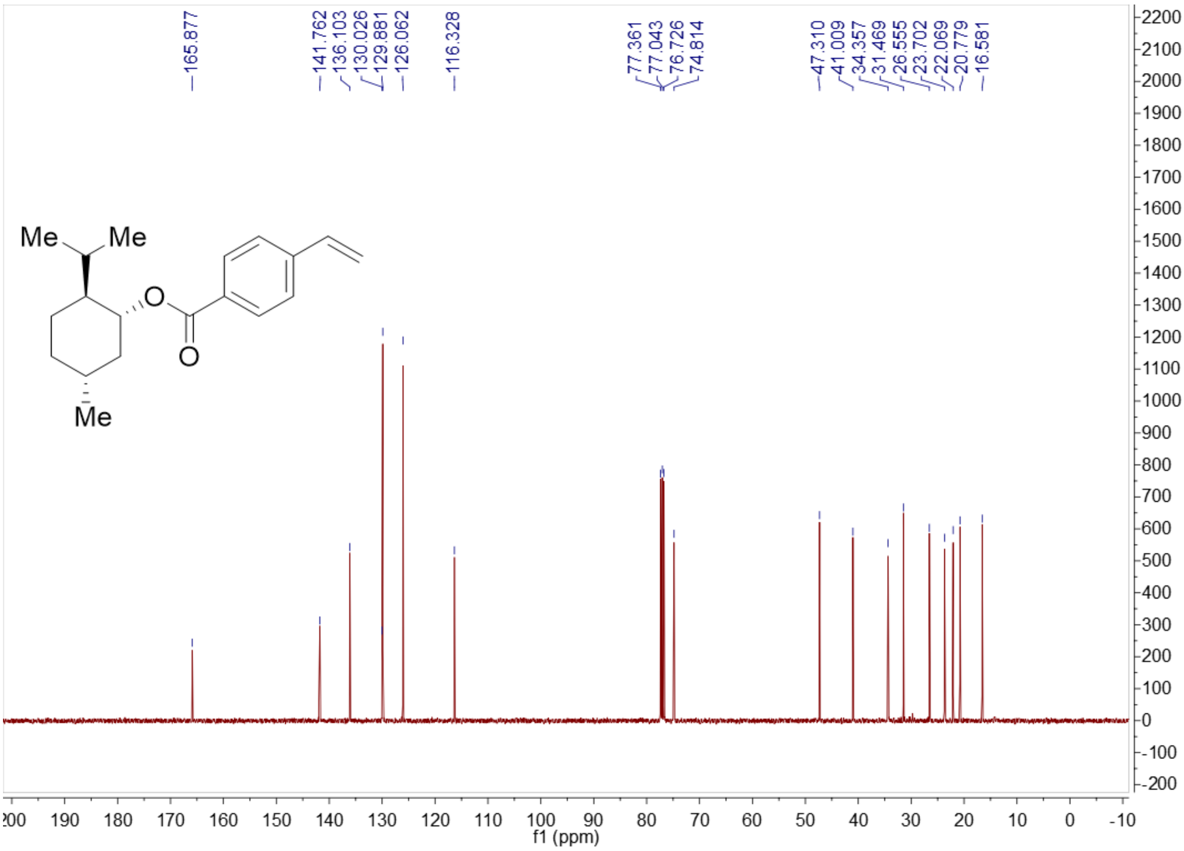

^{1}H NMR of compound **2ae** in $CDCl_3$

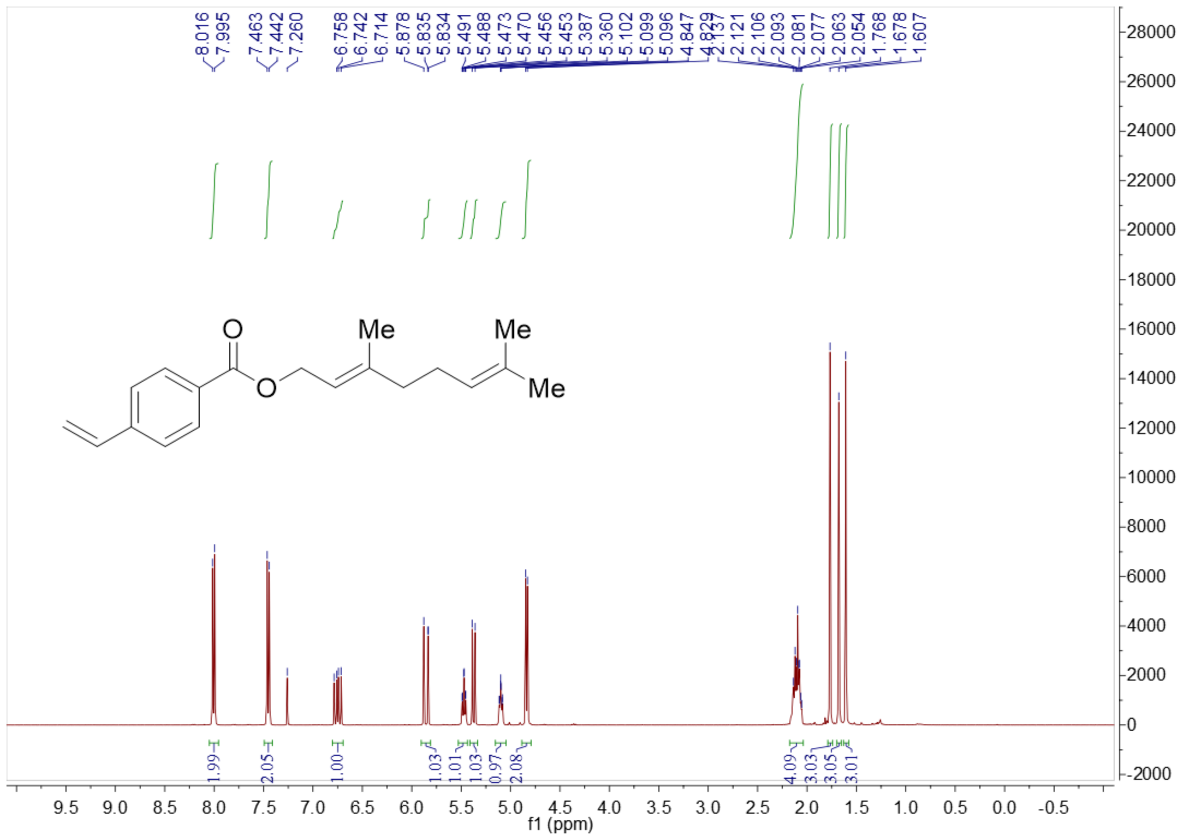

^{13}C NMR of compound **2ae** in $CDCl_3$

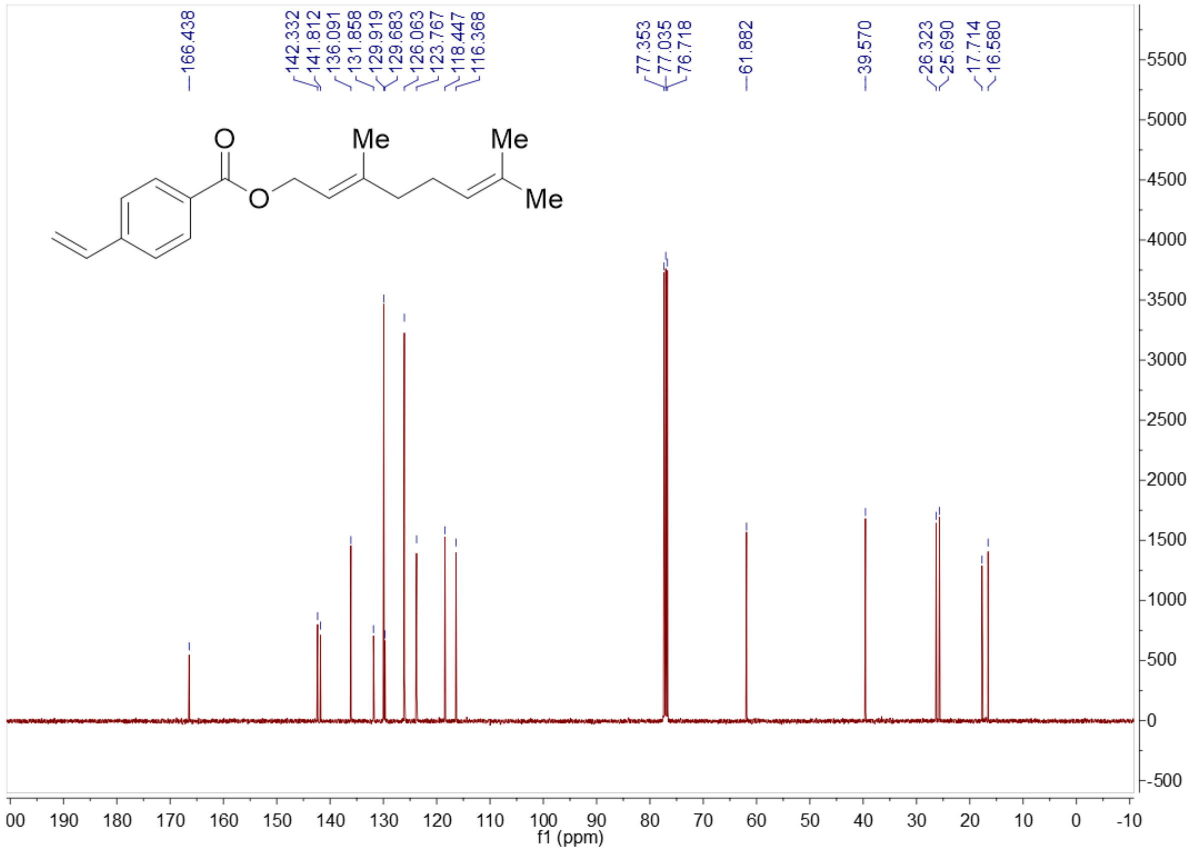

^{1}H NMR of compound **2af** in $CDCl_3$

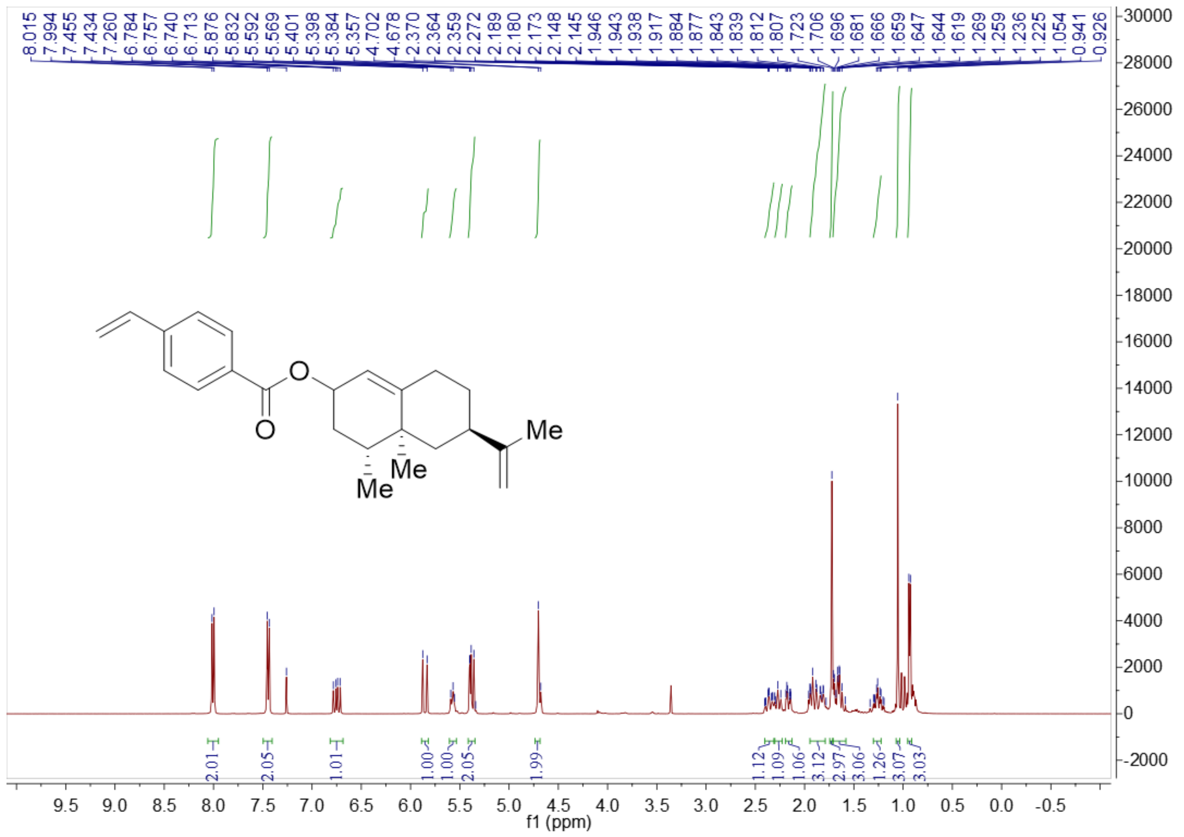

^{13}C NMR of compound **2af** in $CDCl_3$

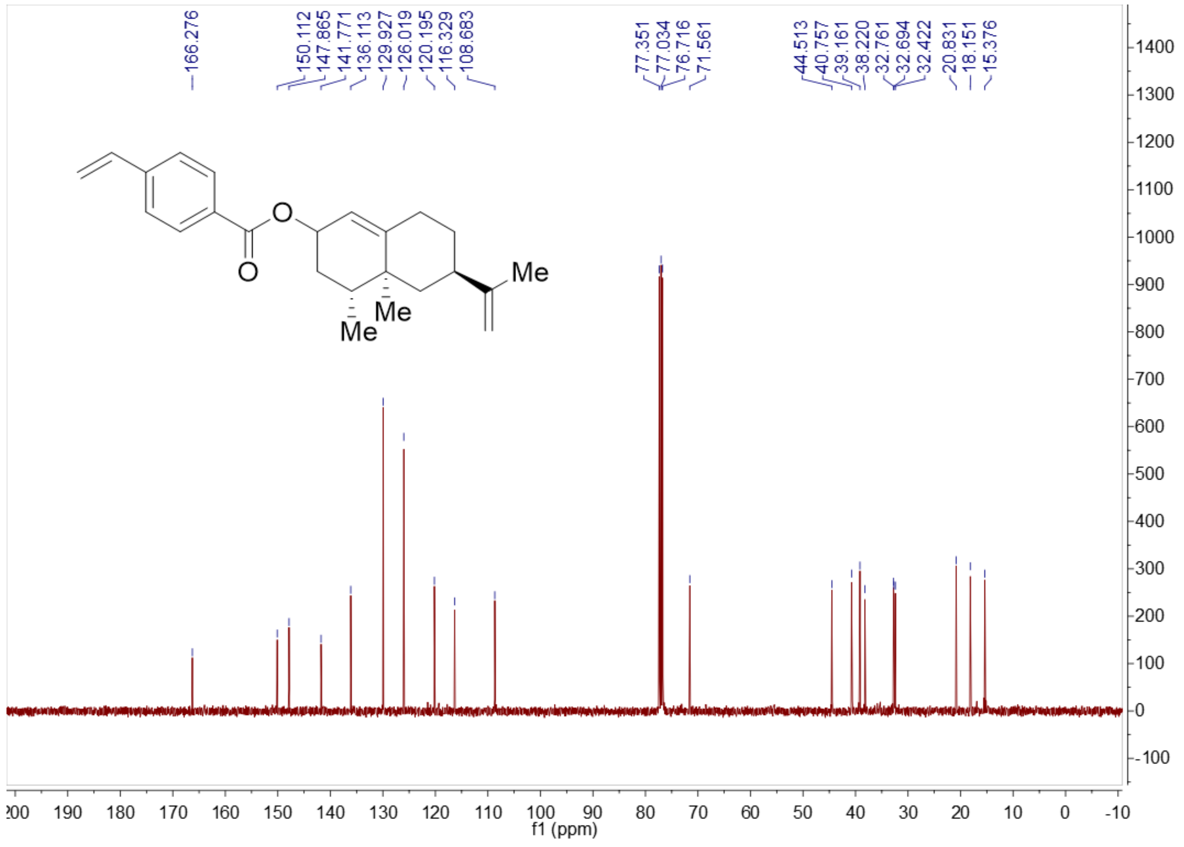

^{1}H NMR of compound **2ag** in $CDCl_3$

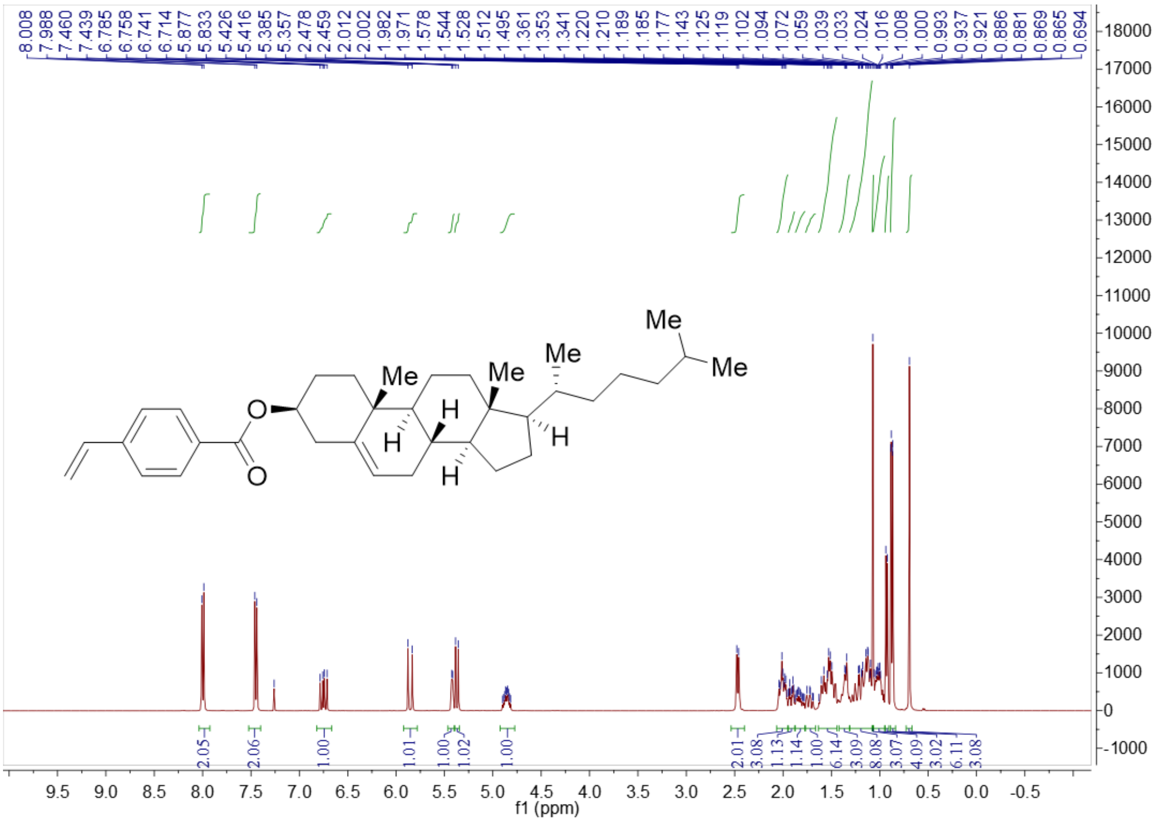

^{13}C NMR of compound **2ag** in $CDCl_3$

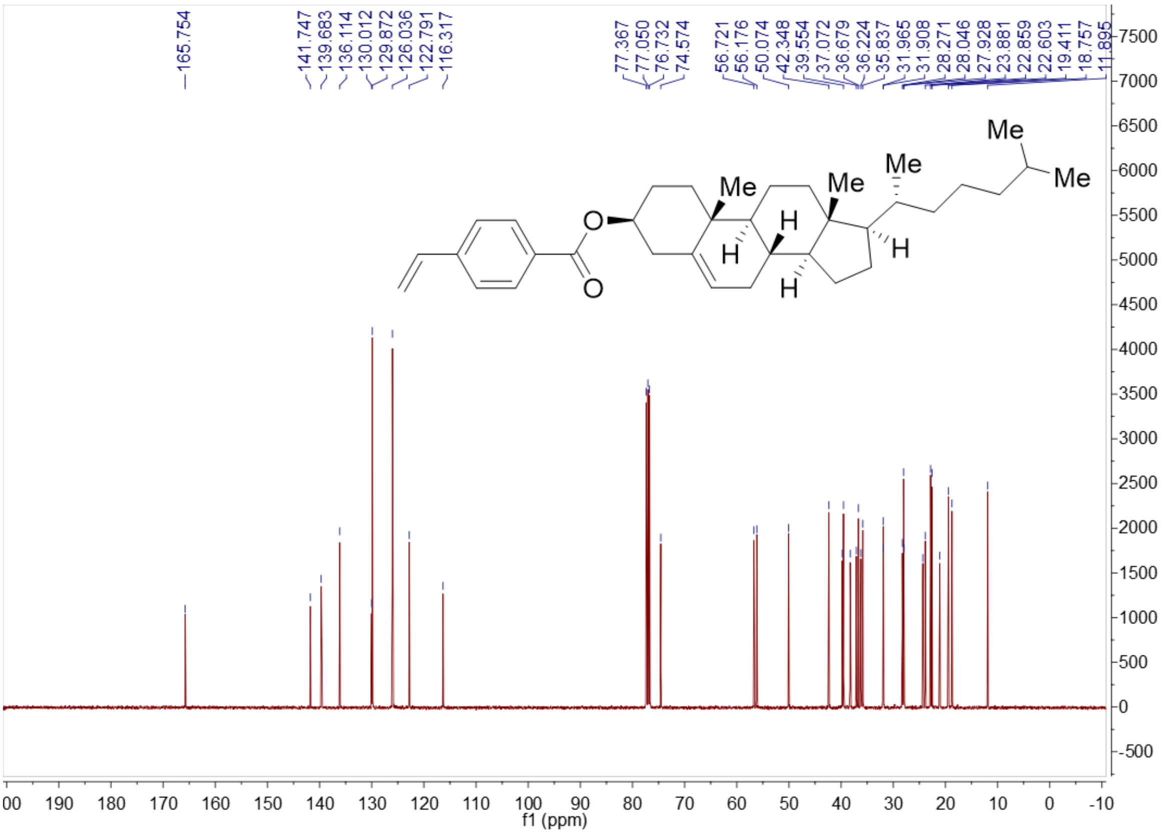

^{1}H NMR of compound **2ah** in $CDCl_3$

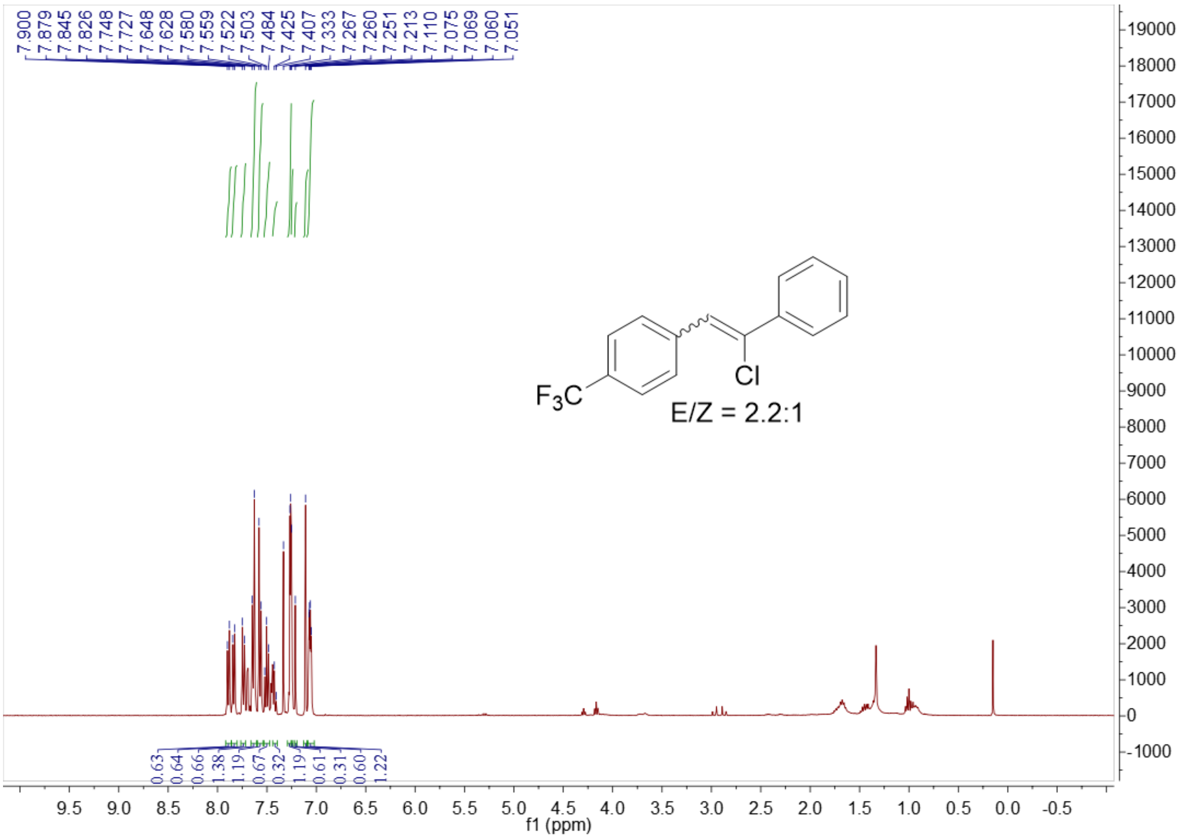

^{19}F NMR of compound **2ah** in $CDCl_3$

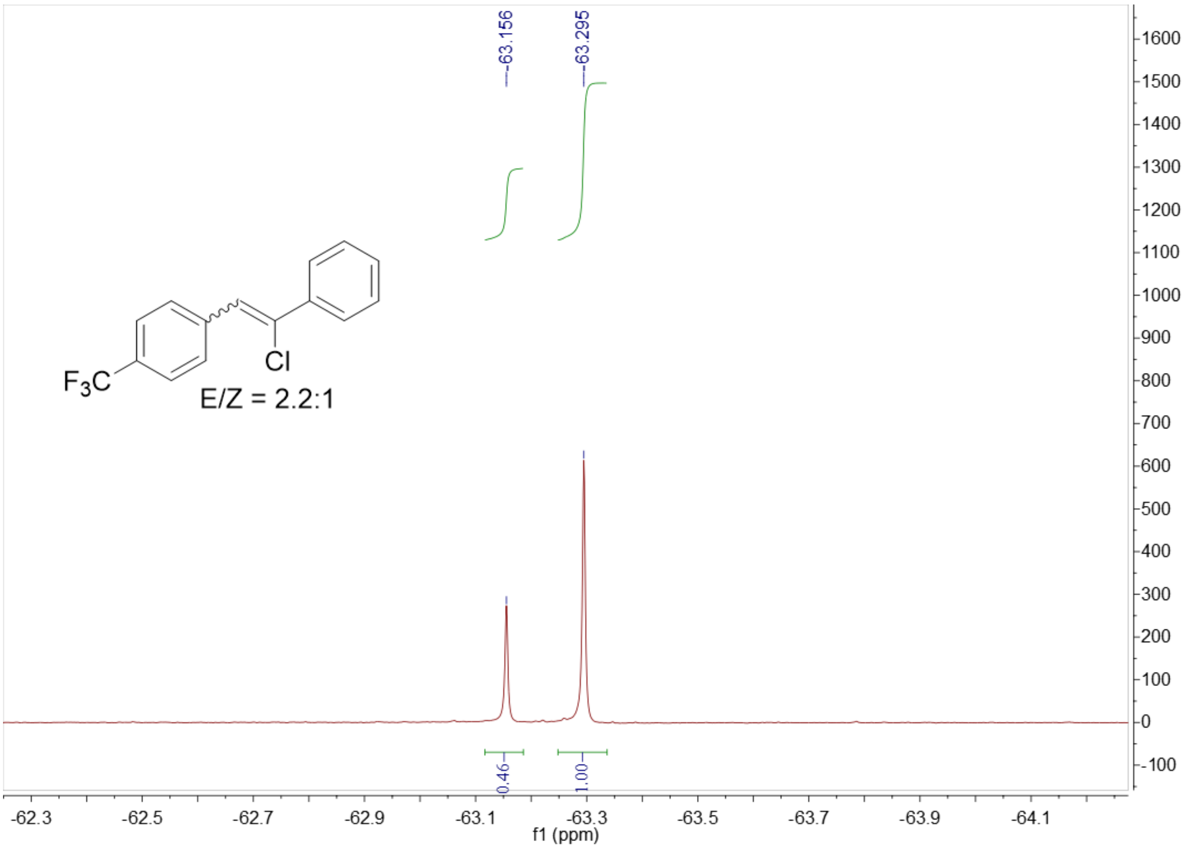

^{1}H NMR of compound ***Z*-2ah** in $CDCl_3$

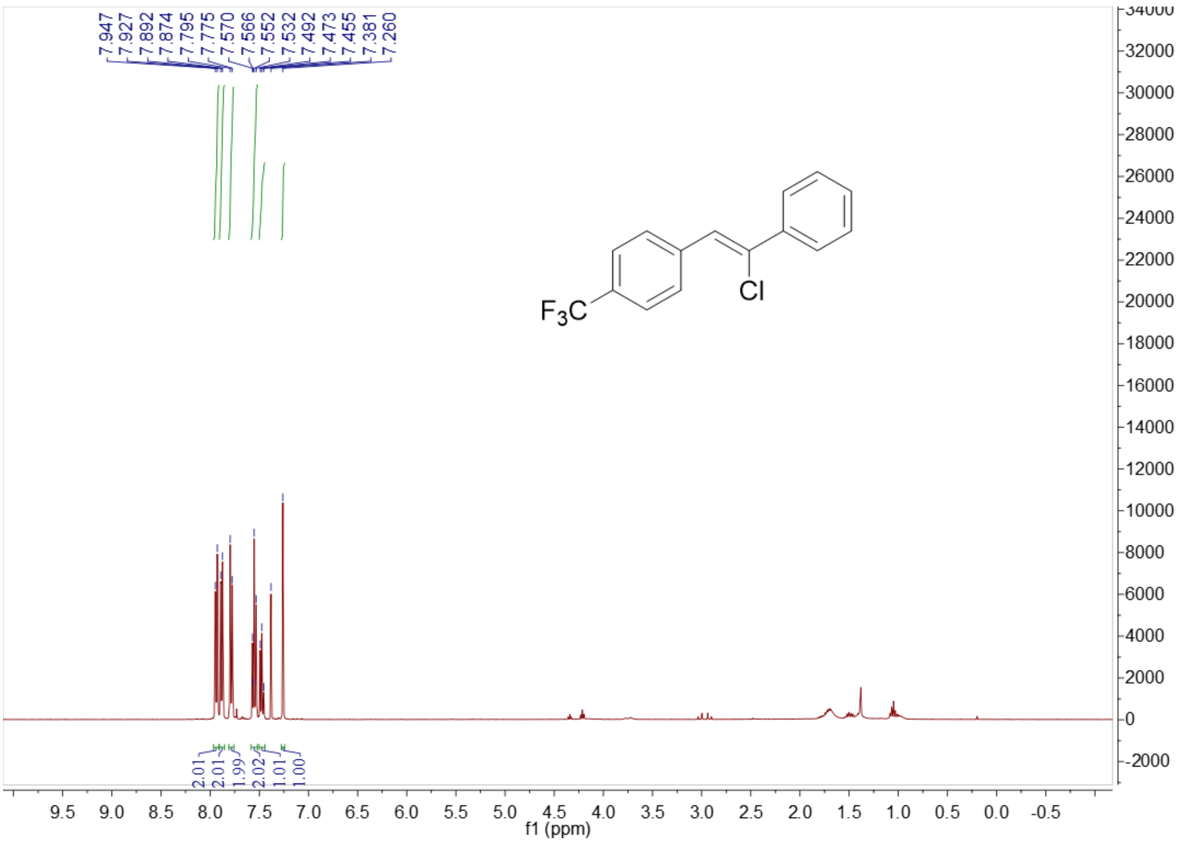

^{13}C NMR of compound ***Z*-2ah** in $CDCl_3$

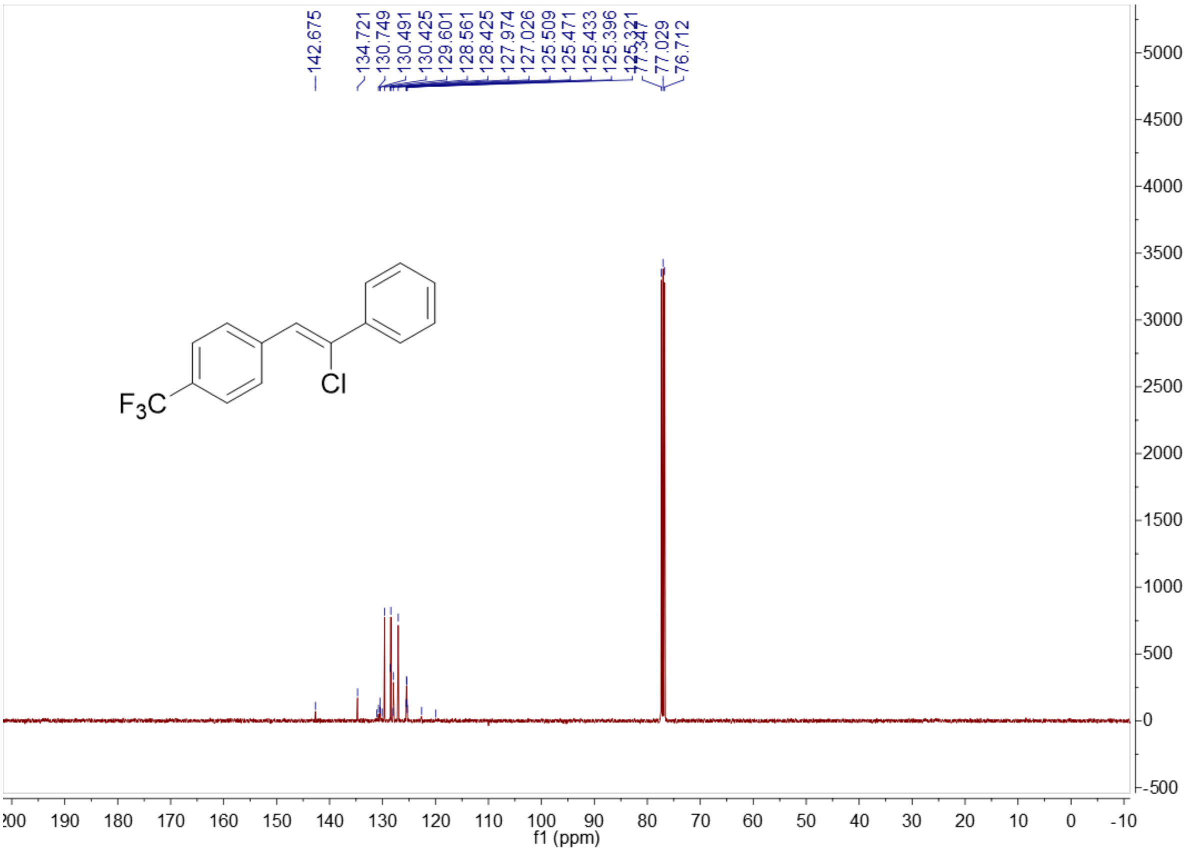

^{1}H NMR of compound **2ai** in $CDCl_3$

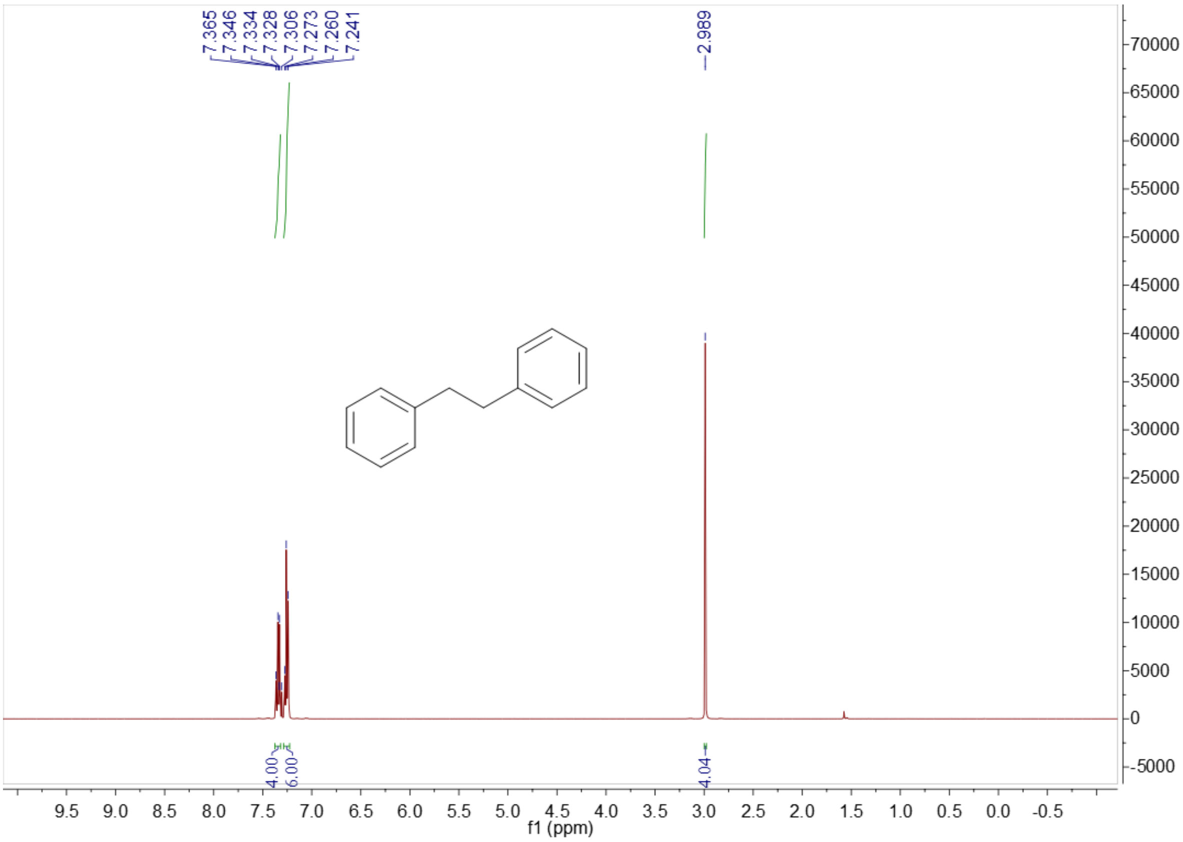

^{13}C NMR of compound **2ai** in $CDCl_3$

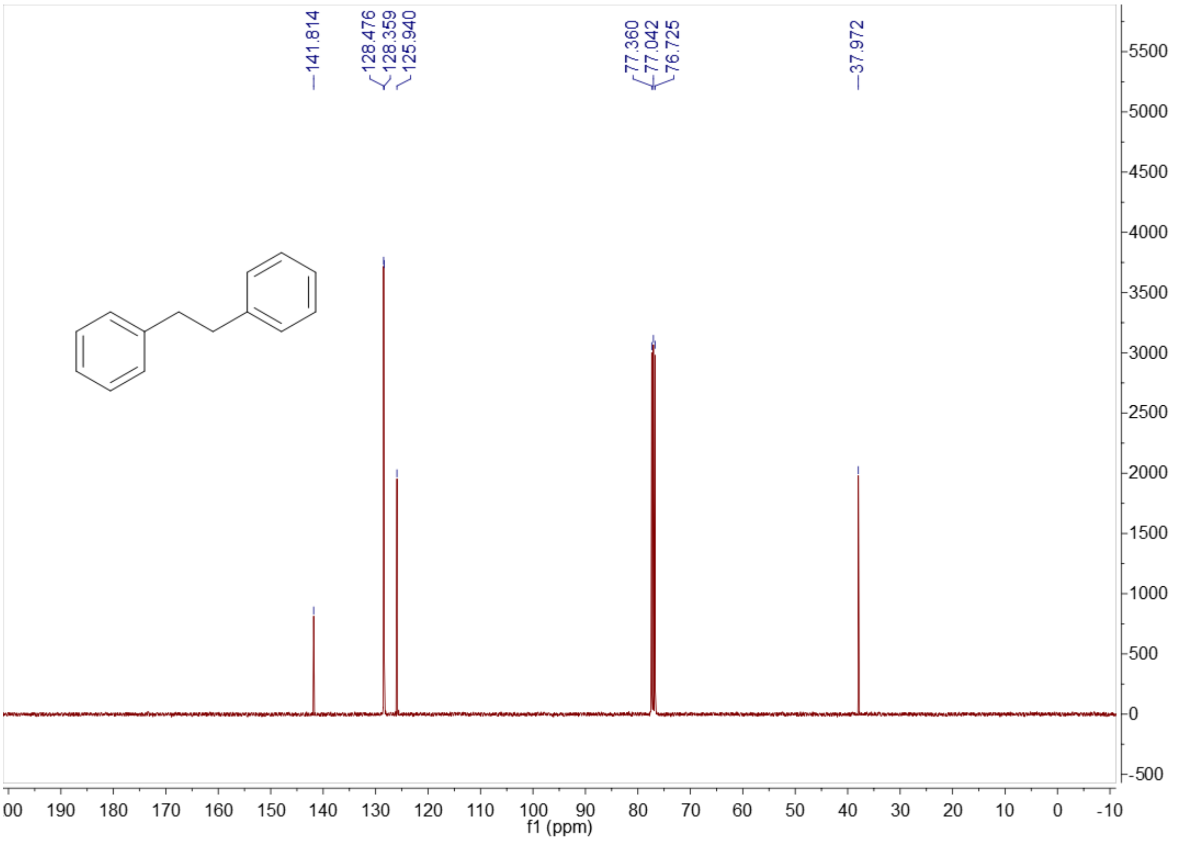

^{1}H NMR of compound **2aj** in $CDCl_3$

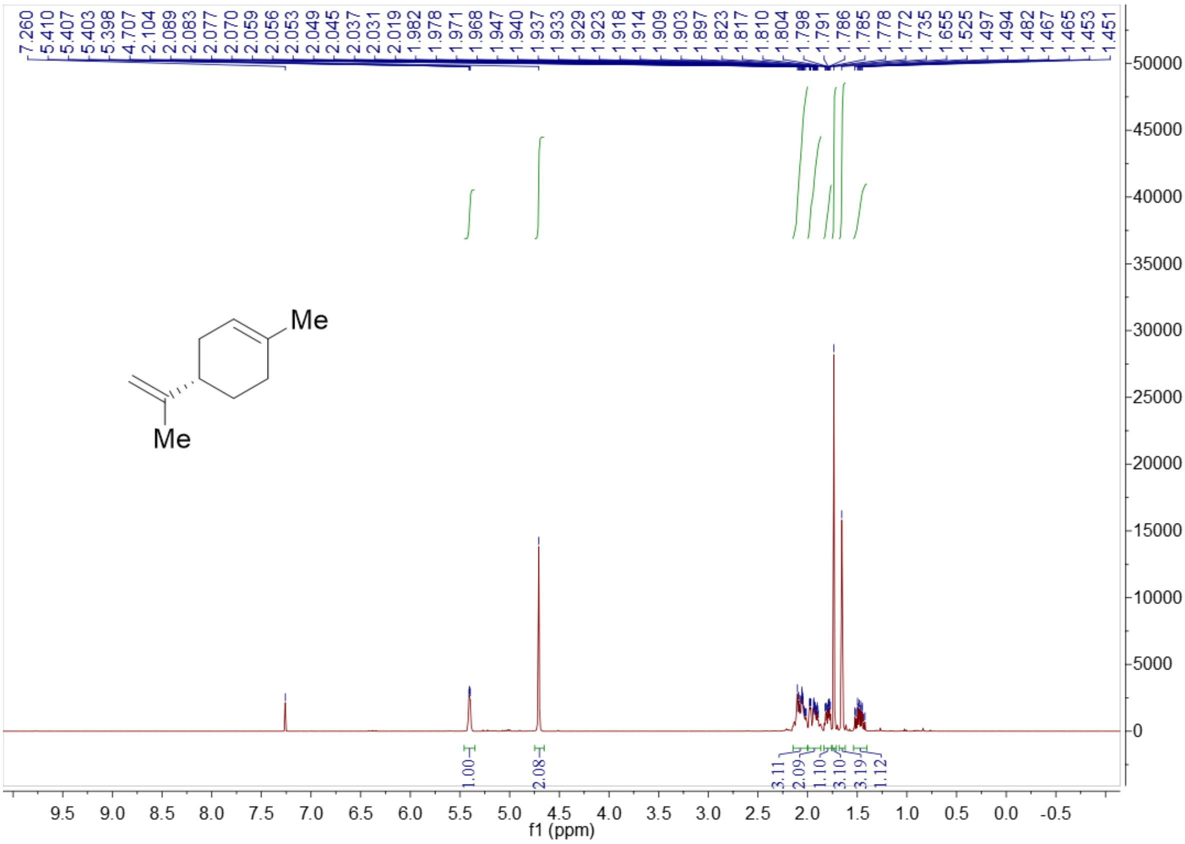

^{13}C NMR of compound **2aj** in $CDCl_3$

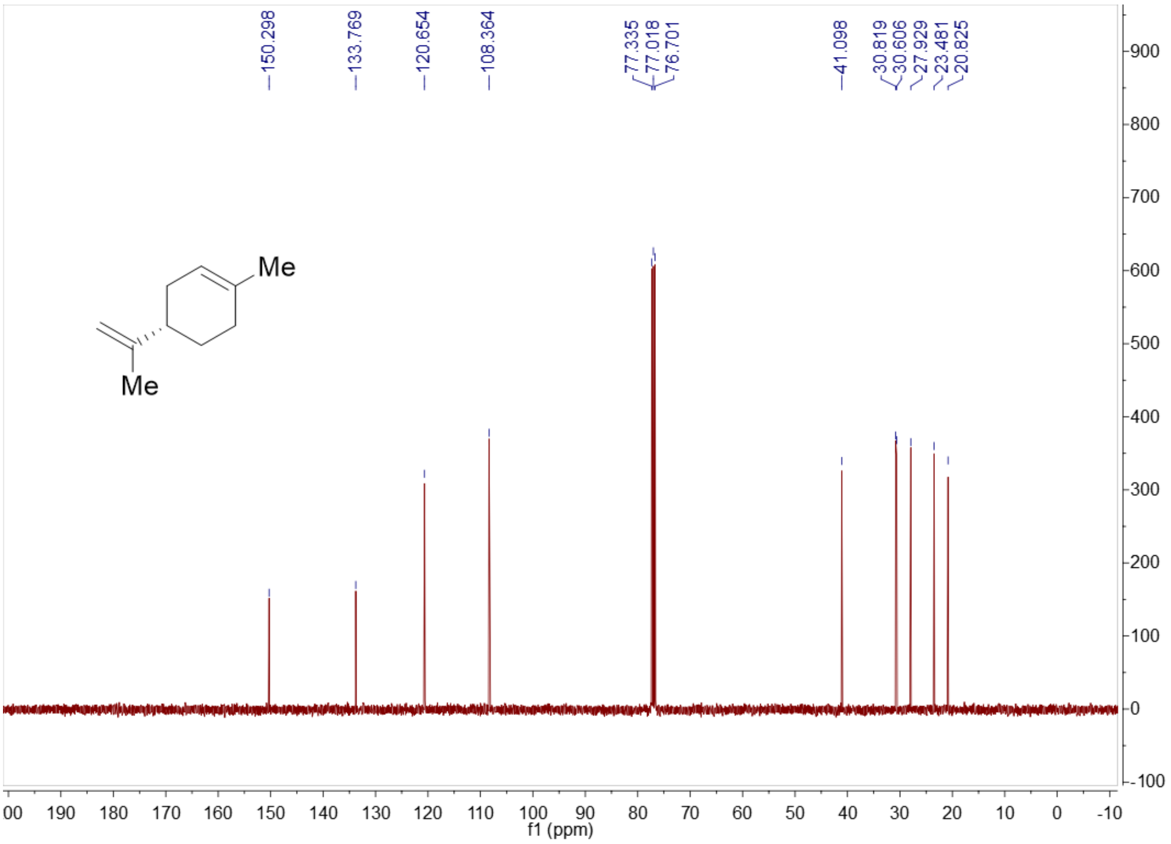

21. X-RAY CRYSTALLOGRAPHY

Single crystal x-ray diffraction data were recorded for a suitable crystal of **ⁿBuO-NpMI**. The crystal was mounted on a MITIGEN holder with inert oil on a XtaLAB Synergy R, DW system, HyPix-Arc 150 diffractometer using Cu-K_a radiation (l= 1.54184 Å). The crystal was kept at a steady T = 100.01(10) K during data collection. Empirical multi-scan[90] and analytical absorption corrections[91] were applied to the data. Structures were solved using SHELXT[92] using dual methods and Olex2 as the graphical interface,[93] and least-squares refinements on F^2 were carried out using SHELXL.[92,94]

All non-hydrogen atoms were refined anisotropically. Hydrogen atom positions were calculated geometrically and refined using the riding model. Most hydrogen atom positions were calculated geometrically and refined using the riding model, but some hydrogen atoms were refined freely.

2078961 (**^{n}BuO-NpMI**) contain the supplementary crystallographic data for this paper. These data are provided free of charge by The Cambridge Crystallographic Data Centre.

Table S10. Crystallographic data and structure refinement for **^{n}BuO-NpMI**.

**Compound**	**^{n}BuO-NpMI**
**Empirical formula**	$C_{22}H_{19}NO_3$
**ρ_{calc}/(g/cm^3)**	1.363
**μ/mm^{-1}**	0.731
**Formula weight**	345.38 g mol^{-1}
**Crystal colour**	clear colourless
**Crystal shape**	plate
**Crystal size/mm^3**	0.18 × 0.13 × 0.05
**Temperature/K**	100.01(10)
**Crystal system**	triclinic
**Space group**	*P*-1
***a*/Å**	5.32030(10)
***b*/Å**	8.91200(10)
***c*/Å**	18.2867(2)
***α*/°**	83.2940(10)
***β*/°**	84.9920(10)
***γ*/°**	78.2530(10)
**Volume/Å^3**	841.29(2)
Z	2
Z'	1

**Wavelength/ Å**	1.54184
**Radiation**	Cu K_α
Θ_{min}/°	2.438
Θ_{max}/°	74.411
**Reflections collected**	31649
**Independent reflections**	3319
**Reflections I≥2 σ(I)**	3018
R_{int}	0.0289
**Parameters**	300
**Restraints**	0
**Largest peak**	0.226
**Deepest hole**	-0.248
**GooF**	1.064
wR_2 **(all data)**	0.0981
wR_2	0.0961
R_1 **(all data)**	0.0374
R_1	0.0345

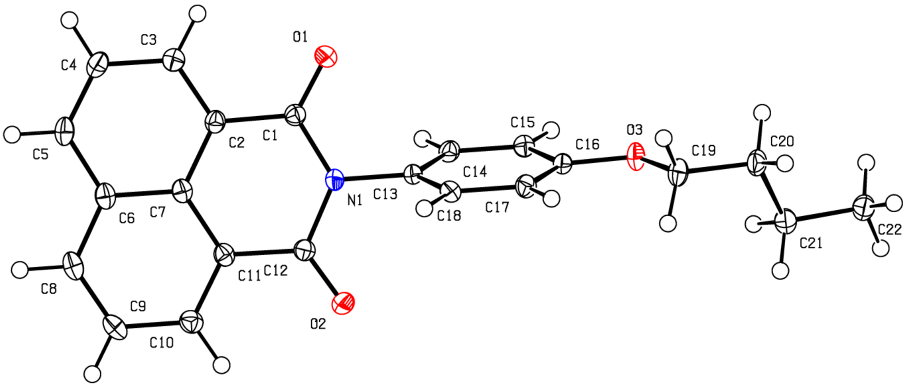

Figure S41. Solid-state molecular structure of **TCBPA**, including atom numbering scheme. Thermal ellipsoids are set at the 50% probability level. C atoms shown in grey, N atoms in blue and O atoms in red.

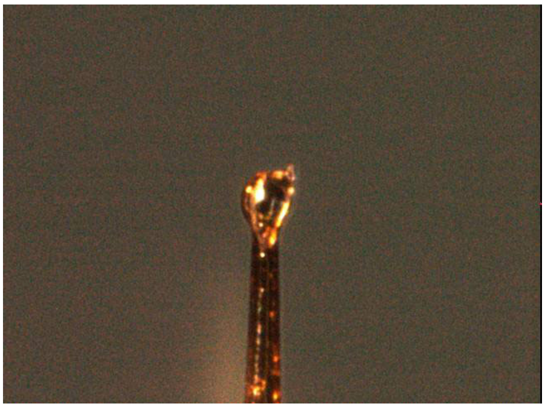

Figure S42. Picture of **ⁿBuO-NpMI** crystal inside the diffractometer.

Table S11. Bond lengths for **^{n}BuO-NpMI**.

Atom	Atom	Length/Å
O3	C16	1.3661(12)
O3	C19	1.4394(13)
O1	C1	1.2188(12)
O2	C12	1.2124(13)
N1	C12	1.4122(13)
N1	C13	1.4522(12)
N1	C1	1.4001(13)
C7	C11	1.4123(14)
C7	C2	1.4132(15)
C7	C6	1.4247(14)
C17	C16	1.3915(15)
C17	C18	1.3978(14)
C16	C15	1.3966(15)
C12	C11	1.4850(14)
C13	C18	1.3803(15)
C13	C14	1.3891(14)
C1	C2	1.4830(14)
C11	C10	1.3780(14)
C2	C3	1.3739(15)
C15	C14	1.3858(14)
C6	C5	1.4149(15)
C6	C8	1.4179(15)
C10	C9	1.4123(15)
C5	C4	1.3710(16)
C3	C4	1.4108(14)
C9	C8	1.3709(16)
C20	C19	1.5115(14)
C20	C21	1.5256(15)
C21	C22	1.5233(14)

Table S12. Bond angles for **TCBPA**.

Atom	Atom	Atom	Angle/°
C16	O3	C19	117.76(8)
C12	N1	C13	117.27(8)
C1	N1	C12	125.33(8)
C1	N1	C13	117.40(8)

Atom	Atom	Atom	Angle/°
C11	C7	C2	120.94(9)
C11	C7	C6	119.67(9)
C2	C7	C6	119.39(10)
C16	C17	C18	119.48(10)
O3	C16	C17	124.35(9)
O3	C16	C15	115.52(9)
C17	C16	C15	120.13(9)
O2	C12	N1	120.65(9)
O2	C12	C11	122.86(9)
N1	C12	C11	116.49(9)
C18	C13	N1	119.21(9)
C18	C13	C14	121.06(9)
C14	C13	N1	119.73(9)
O1	C1	N1	120.85(9)
O1	C1	C2	122.49(9)
N1	C1	C2	116.65(9)
C13	C18	C17	119.80(10)
C7	C11	C12	120.20(9)
C10	C11	C7	120.33(10)
C10	C11	C12	119.47(9)
C7	C2	C1	120.37(9)
C3	C2	C7	120.40(9)
C3	C2	C1	119.21(9)
C14	C15	C16	120.19(10)
C5	C6	C7	118.51(10)
C5	C6	C8	123.13(10)
C8	C6	C7	118.36(10)
C11	C10	C9	120.27(10)
C4	C5	C6	121.10(10)
C15	C14	C13	119.34(9)
C2	C3	C4	120.49(10)
C8	C9	C10	120.27(10)
C9	C8	C6	121.04(10)
C19	C20	C21	113.49(9)
O3	C19	C20	107.37(9)
C5	C4	C3	120.04(10)
C22	C21	C20	112.33(9)

22. REFERENCES

(1) D. D. Perrin; L. F. Armarego, *Purification of Laboratory Compounds*, 3rd Ed.; Pergamon Press, New York, 1992.
(2) S. Wu, J. Žurauskas, M. Domański, P. S. Hitzfeld, V. Butera, D. J. Scott, J. Rehbein, A. Kumar, E. Thyrhaug, J. Hauer, J. P. Barham, *Org. Chem. Front.* **2021**, 8, 1132-1142.
(3) N. G. W. Cowper, C. P. Chernowsky, O. P. Williams, Z. K. Wickens, *J. Am. Chem. Soc.* **2020** *142,* 2093-2099.
(4) L. Song, Y. Yang, Q. Zhang, H. Tian, W. Zhu, *J. Phys. Chem. B* **2011**, *115*, 14648-14658.
(5) W. Zhang, Y. Xu, M. Hanif, S. Zhang, J. Zhou, D. Hu, Z. Xie, Y. Ma, *J. Phys. Chem. C* **2017**, *121*, 23218-23223.
(6) (a) I. Pravst, M. Zupan, S. Stavber, *Tetrahedron*, **2008**, *64*, 5191-5199; (b) J. C. Lee, Y. H. Bae, S.-K. Chang, *Bull. Korean Chem. Soc.* **2003**, *24*, 407-408.
(7) K. Lam, I. E. Markó, *Org. Lett.* **2011**, *13*, 406-409.
(8) S. Yakubov, J. P. Barham, *Beilstein J. Org. Chem.* **2020**, *16*, 2151-2192.
(9) X. Chen, C. Xu, T. Wang, C. Zhou, J. Du, Z. Wang, H. Xu, T. Xie, G. Bi, J. Jiang, X. Zhang, J. N. Demas, C. O. Trindle, Y. Luo, G. Zhang, *Angew. Chem. Int. Ed.* **2016**, *55*, 9872-9876.
(10) H. Kakuta, A. Tanatani, K. Nagasawa, Y. Hashimoto, *Chem. Pharm. Bull.* **2003**, *51*, 1273-1282.
(11) D. J. Fox, D. S. Pedersen, A. B. Petersen, S. Warren, *Org. Biomol. Chem.* **2006**, *4*, 3117.
(12) R. Chauvin, *Tetrahedron: Asymmetry*, **1990**, *1*, 737-742.
(13) F. W. Lewis, T. C. McCabe, D. H. Grayson, *Tetrahedron*, **2011**, *67*, 7517-7528.
(14) C. Belger, N. M. Neisius, B. Plietker, *Chem.-Eur. J.* **2010**, *16*, 12214-12220.
(15) T. Shimasaki, Y. Konno, M. Tobisu, N. Chatani, *Org. Lett.* **2009**, *11*, 4890-4892.
(16) T. Ljungdahl, T. Bennur, A. Dallas, H. Emtenäs, J. Mårtensson, *Organometallics* **2008**, *27*, 2490-2498.
(17) M. Yasuda, R. Kojima, H. Tsutsui, D. Utsunomiya, K. Ishii, K. Jinnouchi, T. Shiragami, T. Yamashita, *J. Org. Chem.* **2003**, *68*, 7618-7624.
(18) N. Taniguchi, *J. Org. Chem.* **2006**, *71*, 7874-7876.
(19) B. Scheiper, M. Bonnekessel, H. Krause, A. Fürstner, *J. Org. Chem.* **2004**, *69*, 3943-3949.
(20) P. Mayo, W. Tam, *Tetrahedron*, **2002**, *58*, 9527-9540.
(21) A. L. Hansen, J.-P. Ebran, T. M. Gøgsig, T. Skrydstrup, *J. Org. Chem.* **2007**, *72*, 6464-6472.
(22) R. Kumar, A. Sharma, N. Sharma, V. Kumar, A. K. Sinha, *Eur. J. Org. Chem.* **2008**, 5577-5582.
(23) A. E. Díaz-Álvarez, P. Crochet, V. Cadierno, *Tetrahedron* **2012**, *68*, 2611-2620.
(24) S. Fantauzzi, E. Gallo, A. Caselli, F. Ragaini, P. Macchi, N. Casati, S. Cenini, *Organometallics* **2005**, *24*, 4710-4713.
(25) K. Ahmad, N. F. Thomas, M. R. Mukhtar, I. Noorbatcha, J.-F. F. Weber, M. A. Nafiah, S. S. Velu, K. Takeya, H. Morita, C.-G. Lim, A. H. A. Hadi, K. Awang, *Tetrahedron* **2009**, *65*, 1504-1516.
(26) B. M. Trost, H. C. Arndt, *J. Am. Chem. Soc.* **1973**, *95*, 5288-5298.
(27) M. Al-Masum, S. Alam, *Tetrahedron Lett.* **2009**, *50*, 5201-5204.
(28) Y. Zhao, Q. Liu, J. Li, Z. Liu, B. Zhou, *Synlett*, **2010**, 1870-1872.
(29) U. P. N. Tran, G. Oss, D. P. Pace, J. Ho, T. V. Nguyen, *Chem. Sci.* **2018**, *9*, 5145-5151.
(30) P. Batsomboon, W. Phakhodee, S. Ruchirawat, P. Ploypradith, *J. Org. Chem.* **2009**, *74*, 4009-4012.
(31) D. M. Hodgson, M. J. Fleming, S. J. Stanway, *J. Am. Chem. Soc.* **2004**, *126*, 12250-12251.
(32) E. Alacid, C. Nájera, *J. Org. Chem.* **2009**, *74*, 2321-2327.
(33) T. Satoh, T. Kasuya, M. Ishigaki, M. Inumaru, T. Miyagawa, N. Nakaya, S. Sugiyama, *Synthesis* **2011**, 397-408.
(34) N. Kambe, Y. Moriwaki, Y. Fujii, T. Iwasaki, J. Terao, *Org. Lett.* **2011**, *13*, 4656-4659.
(35) S. E. Denmark, C. R. Butler, *Org. Lett.* **2006**, *8*, 63-66.
(36) V. Paquet, H. Lebel, *Synthesis* **2005**, 1901-1905.
(37) J. Y. Wu, B. Moreau, T. Ritter, *J. Am. Chem. Soc.* **2009**, *131*, 12915-12917.
(38) M. Teraguchi , M. Ohtake , H. Inoue , A. Yoshida , T. Aoki , T. Kaneko , K. Yamanada, *J. Polym. Sci., Part A: Polym. Chem.* **2005**, *43*, 2348-2357.
(39) L. Chen, S. Jin, J. Gao, T. Liu, Y. Shao, J. Feng, K. Wang, T. Lu, D. Du, *Org. Lett.* **2021**, *23*, 394-399.
(40) P. J. Black, M. G. Edwards, J. M. J. Williams, *Eur. J. Org. Chem.* **2006**, 4367-4378.
(41) Y. Takada, J. Caner, S. Kaliyamoorthy, H. Naka, S. Saito, *Chem. Eur. J.* **2017**, *23*, 18025-18032.
(42) O. Abdul-Rahim, A. N. Simonov, T. Rüther, J. F. Boas, A. A. J. Torriero, D. J. Collins, P. Perlmutter, A. M. Bond, *Anal. Chem.* **2013**, *85*, 6113-6120.
(43) L. Pause, M. Robert, J.-M. Savéant, *J. Am. Chem. Soc.* **1999**, *121*, 7158-7159.
(44) P. Cankař, D. Dubas, S. C. Banfield, M'hamed Chahma, T. Hudlicky, *Tetrahedron Lett.* **2005**, *46*, 6851-6854.
(45) F. Strieth-Kalthoff, M. J. James, M. Teders, L. Pitzer, F. Glorius, *Chem. Soc. Rev.* **2018**, *47*, 7190-7202.
(46) R. O. Marcon, S. Brochsztain, *J. Phys. Chem. A* **2009**, *113*, 1747-1752.
(47) D. Gosztola, M. P. Niemczyk, W. Svec, A. S. Lukas, M. R. Wasielewski, *J. Phys. Chem. A* **2000**, *104*, 6545-6551.
(48) C. Lu, M. Fujitsuka, A. Sugimoto, T. Majima, *J. Phys. Chem. C.* **2016**, *120*, 12734-12741.

(49) C. J. Zeman IV, S. Kim, F. Zhang, K. S. Schanze, *J. Am. Chem. Soc.* **2020**, *142*, 2204-2207.
(50) A. Demeter, T. Bercés, L. Biczók, V. Wintgens, P. Valat, J. Kossanyi, *J. Phys. Chem.* **1996**, *100*, 2001-2011.
(51) (a) J. Kong, S. Yu, *Acta Biochemica et Biophysica Sinica* **2007**, *39*, 549-559; (b) R. Chaudret, B. De Courcy, J. Contreras-Garciá, E. Gloaguen, A. Zehnacker-Rentein, M. Mons, J.-P. Piquemal, *Phys. Chem. Chem. Phys.* **2014**, *16*, 9876-9891.
(52) A. K. Lemmens, P. Chopra, D. Garg, A. L. Steber, M. Schnell, W. J. Buma, *Molecular Physics* **2021**, *119*, e1811908.
(53) D. R. Duling *J. Mag. Reson.* **1994**, *104*, 105-110.
(54) V. A. Ryabinin, V. F. Starichenko, G. N. Vorozhtsov, S. M. Shein, *J. Struct. Chem.* **1978**, *19*, 693-701.
(55) (a) Hohenberg, P., Kohn, W. *Phys. Rev.* **1964**, *36*, 864-871; (b) Kohn, W., Sham, L. J. *Phys. Rev.* **1965**, *140*, 1133-1138.
(56) M. J. Frisch, G. W. Trucks, H. B. Schlegel, G. E. Scuseria, M. A. Robb, J. R. Cheeseman, G. Scalmani, V. Barone, G. A. Petersson, H. Nakatsuji, X. Li, M. Caricato, A. V. Marenich, J. Bloino, B. H. Janesko, R. Gomperts, B. Mennucci, H. P. Hratchian, J. V. Ortiz, A. F. Izmaylov, J. L. Sonnenberg, D. F. Williams, F. Lapparini, F. Egidi, J. Goings, B. Peng, A. Petrone, T. Henderson, D. Ranasinghe, V. G. Zakrzewski, J. Gao, N. Rega, G. Zheng, W. Liang, M. Hada, M. Ehara, K. Toyota, R. Fukuda, J. Hasegawa, M. Ishida, T. Nakajima, Y. Honda, O. Kitao, H. Nakai, T. Vreven, K. Throssell, J. A. Montgomery Jr., J. E. Peralta, F. Ogliaro, M. Bearpark, J. J. Heyd, E. N. Brothers, K. N. Kudin, V. N. Staroverov, R. Kobayashi, J. Normand, K. Raghavachari, A. P. Rendell, J. C. Burant, S. S. Iyengar, J. Tomasi, M. Cossi, J. M. Millam, M. Klene, C. Adamo, R. Cammi, J. W. Ochterski, R. L. Martin, K. Morokuma, Ö. Farkas, J. B. Foresman, D. J. Fox, Gaussian 16, Rev. C.01. Wallingford, CT, **2016**.
(57) J.-D. Chai, M. Head-Gordon, *Phys. Chem. Chem. Phys.* **2008**, *10*, 6615-6620
(58) (a) M. J. Frisch, J. A. Pople, J. S. Binkley, *J. Chem. Phys.* **1984**, *80*, 3265–3269; (b) R. Krishnan, J. S. Binkley, R. Seeger, J. A. Pople, *J. Chem. Phys.* **1980**, *72*, 650–654; (c) A. D. McLean, G. S. Chandler, *J. Chem. Phys.* **1980**, *72*, 5639–5648.
(59) (a) J. Tomasi, B. Mennucci, R. Cammi, *Chem. Rev.* **2005**, *105*, 2999–3094; (b) G. Scalmani, M. J. Frisch, *J. Chem. Phys.* **2010**, *132*, 114110.
(60) W. Humphrey, A. Dalke, K. Schulten, *J. Mol. Graph.* **1996**, *14*, 33–38.
(61) (a) L. Goerigk, A. Hansen, C. Bauer, S. Ehrlich, A. Najibi, S. Grimme, *Phys. Chem. Chem. Phys.* **2017**, *19*, 32184–32215; (b) N. Mardirossian, M. Head-Gordon, *Mol. Phys.* **2017**, *115*, 2315–2372.
(62) L. Goerigk, N. Mehta, *Aust. J. Chem.* **2019**, *72*, 563–573.
(63) H. Kruse, L. Goerigk, S. Grimme, *J. Org. Chem.* **2012**, *77*, 10824–10834.
(64) (a) D. G. Liakos, Y. Guo, F. Neese, *J. Phys. Chem. A* **2020**, *124*, 90–100; (b) M. Saitow, U. Becker, C. Riplinger, E. F. Valeev, F. Neese, *J. Chem. Phys.* **2017**, *146*, 164105.
(65) (a) F. Neese, *WIREs Comput. Mol. Sci.* **2012**, *2*, 73–78; (b) F. Neese, *WIREs Comput. Mol. Sci.* **2018**, *8*, e1327.
(66) (a) D. Rappoport, F. Furche, *J. Chem. Phys.* **2010**, *133*, 134105; (b) F. Weigend, R. Ahlrichs, *Phys. Chem. Chem. Phys.* **2005**, *7*, 3297–3305.
(67) (a) A. Hellweg, C. Hättig, S. Höfener, W. Klopper, *Theor. Chem. Acc.* **2007**, *117*, 587–597; (b) A. Hellweg, D. Rappoport, *Phys. Chem. Chem. Phys.* **2015**, *17*, 1010–1017.
(68) (a) V. Barone, M. Cossi, *J. Phys. Chem. A* **1998**, *102*, 1995–2001; (b) M. Cossi, N. Rega, G. Scalmani, V. Barone, *J. Comput. Chem.* **2003**, *24*, 669–681.
(69) J. E. Bartmess, *J. Phys. Chem.* **1994**, *98*, 6420–6424.
(70) (a) L. E. Roy, E. Jakubikova, M. G. Guthrie, E. R. Batista, *J. Phys. Chem. A* **2009**, *113*, 6745–6750; (b) D. G. Truhlar, C. J. Cramer, A. Lewis, J. A. Bumpus, *J. Chem. Educ.* **2004**, *81*, 596; (c) S. J. Konezny, M. D. Doherty, O. R. Luca, R. H. Crabtree, G. L. Soloveichik, V. S. Batista, *J. Phys. Chem. C* **2012**, *116*, 6349–6356; (d) S. Maier, B. Thapa, K. Raghavachari, *Phys. Chem. Chem. Phys.* **2020**, *22*, 4439–4452; (e) A. P. Davis, A. J. Fry, *J. Phys. Chem. A* **2010**, *114*, 12299–12304; (f) A. A. Isse, A. Gennaro, *J. Phys. Chem. B* **2010**, *114*, 7894–7899; (g) Y. Paukku, G. Hill, *J. Phys. Chem. A* **2011**, *115*, 4804–4810.
(71) (a) A. Francisco da Silva, A. João da Silva Filho, M. L. A. A. Vasconcellos, O. Luís de Santana, *Molecules* **2018**, *23*, 2129; (b) J. Ho, A. Klamt, M. L. Coote, *J. Phys. Chem. A* **2010**, *114*, 13442–13444.
(72) M. Namazian, C. Y. Lin, M. L. Coote, *J. Chem. Theory Comput.* **2010**, *6*, 2721–2725.
(73) M. Isegawa, F. Neese, D. A. Pantazis, *J. Chem. Theory Comput.* **2016**, *12*, 2272–2284.
(74) (a) S. Grimme, M. Waletzke, *J. Chem. Phys* **1999**, *111*, 5645–5655. (b) I. Lyskov, M. Kleinschmidt, C. M. Marian, *J. Chem. Phys.* **2016**, *144*, 034104. (c) A. Heil, C. M. Marian, *J. Chem. Phys.* **2017**, *147*, 194104. (d) A. Heil, M. Kleinschmidt, C. M. Marian, *J. Chem. Phys.* **2018**, *149*, 164104. (e) C. M. Marian, A. Heil, M. Kleinschmidt, *WIREs Comput. Mol.Sci.* **2019**, *9*, e1394.
(75) (a) C. Lee, W. Yang, R. G. Parr, *Phys. Rev. B* **1988**, *37*, 785–798. (b) A. D. Becke, *J. Chem. Phys.* **1993**, *98*, 1372–1377.
(76) (a) F. Weigend, M. Kattannek, R. Ahlrichs, *J. Chem. Phys.* **2009**, *130*, 164106. (b) S. Kossmann, F. Neese, *Chem. Phys. Lett.* **2009**, *481*, 240–243.
(77) F. Weigend, *J. Comput. Chem.* **2008**, *29*, 167–175.
(78) F. Neese, *J. Am. Chem. Soc.* **2006**, *128*, 10213–10222.
(79) H. Werner, W. Meyer, *J. Chem. Phys.* **1981**, *74*, 5794–5801.
(80) H. Werner, P. J. Knowles, *J. Chem. Phys.* **1985**, *82*, 5053–5063.

(81) D. A. Kreplin, P. J. Knowles, H.-J. Werner, *J. Chem. Phys.* **2020**, *152*, 074102.

(82) H.-J. Werner, P. J. Knowles, G. Knizia, F. R. Manby, M. Schütz, P. Celani, W. Györffy, D. Kats, T. Korona, R. Lindh, A. Mitrushenkov, G. Rauhut, K. R. Shamasundar, T. B. Adler, R. D. Amos, S. J. Bennie, A. Bernhardsson, A. Berning, D. L. Cooper, M. J. O. Deegan, A. J. Dobbyn, F. Eckert, E. Goll, C. Hampel, A. Hesselmann, G. Hetzer, T. Hrenar, G. Jansen, C. Köppl, S. J. R. Lee, Y. Liu, A. W. Lloyd, Q. Ma, R. A. Mata, A. J. May, S. J. McNicholas, W. Meyer, T. F. Miller III, M. E. Mura, A. Nicklass, D. P. O'Neill, P. Palmieri, D. Peng, K. Pflüger, R. Pitzer, M. Reiher, T. Shiozaki, H. Stoll, A. J. Stone, R. Tarroni, T. Thorsteinsson, M. Wang, M. Welborn, *MOLPRO, Version 2021.1, a Package of Ab Initio Programs*, **2021**.

(83) H.-J. Werner, P. J. Knowles, G. Knizia, F. R. Manby, M. Schütz, *WIREs Comput. Mol. Sci.* **2012**, *2*, 242–253.

(84) H.-J. Werner, P. J. Knowles, F. R. Manby, J. A. Black, K. Doll, A. Heßelmann, D. Kats, A. Köhn, T. Korona, D. A. Kreplin, Q. Ma, T. F. Miller, A. Mitrushchenkov, K. A. Peterson, I. Polyak, G. Rauhut, M. Sibaev, *J. Chem. Phys.* **2020**, *152*, 144107.

(85) T. H. Dunning, *J. Chem. Phys.* **1989**, *90*, 1007–1023.

(86) R. A. Kendall, T. H. Dunning, R. J. Harrison, *J. Chem. Phys.* **1992**, *96*, 6796–6806.

(87) W. Győrffy, T. Shiozaki, G. Knizia, H.-J. Werner, *J. Chem. Phys.* **2013**, *138*, 104104.

(88) B. Helmich-Paris, *J. Chem. Theory Comput.* **2019**, *15*, 4170–4179.

(89) (a) C. A. Hunter, J. K. M. Sanders, *J. Am. Chem. Soc.* **1990**, *112*, 5525-5534; (b) C. R. Martinez, B. L. Iverson, *Chem. Sci.* **2012**, *3*, 2191-2201.

(90) (a) SCALE3ABS, CrysAlisPro, Aglient Technologies Inc. Oxford and GB, **2015**. (b) Sheldrick, G. M. SADABS, Bruker AXS, Madison and USA, **2007**.

(91) (a) Clark, R. C.; Reid, J. S. *Acta Cryst. A*, **1995**, *51*, 887-897. (b) CrysAlisPro, version 171.39.37b, Agilent Technologies Inc., Oxford and GB, **2017**.

(92) Sheldrick, G. M. *Acta Cryst. A*, **2015**, *71*, 3-8.

(93) Dolomanov, O. V.; Bourhis, L. J.; Gildea, R. J.; Howard, J. A. K.; Puschmann, H. *J. Appl. Crystallogr.* **2009**, *42*, 339-341.

(94) Sheldrick, G. M. *Acta Cryst. A*, **2008**, *64*, 112-22.
